# Cobalt-Catalyzed Enantioselective Intermolecular C–H Carboamination with Racemic Allenes

**DOI:** 10.1021/jacs.6c14940

**Published:** 2026-09-07

**Authors:** Lenin Kumar Verdhi, Nicolai Cramer

**Affiliations:** Laboratory of Asymmetric Catalysis and Synthesis, Institute of Chemical Sciences and Engineering, 27218 Ecole Polytechnique Fédérale de Lausanne (EPFL) 1015, Lausanne, Switzerland

## Abstract

Enantioselective carboamination of allenes especially in conjunction with 3D-metal catalysis remains a significant challenge. We report chiral cyclopentadienyl cobalt^III^-catalyzed regio-, chemo-, and enantioselective intermolecular C–H carboamination of racemic allenyl esters with *N*-phenoxyisobutyramides. The protocol offers an efficient approach for highly selective carboamination of allenes and affords synthetically valuable chiral γ-substituted-β-enamido esters with exceptional selectivities (up to 88% yield, >99:1 er, and >20:1 *E/Z*). Operating by a kinetic resolution pathway, the transformation was additionally optimized for recovery of enantioenriched allenes with high selectivity factors of up to 138. The utility was showcased by the resolution of methyl-(*R*,*E*)-(−)-tetradeca-2,4,5-trienoate, the male bean weevil pheromone.

## Graphical abstract

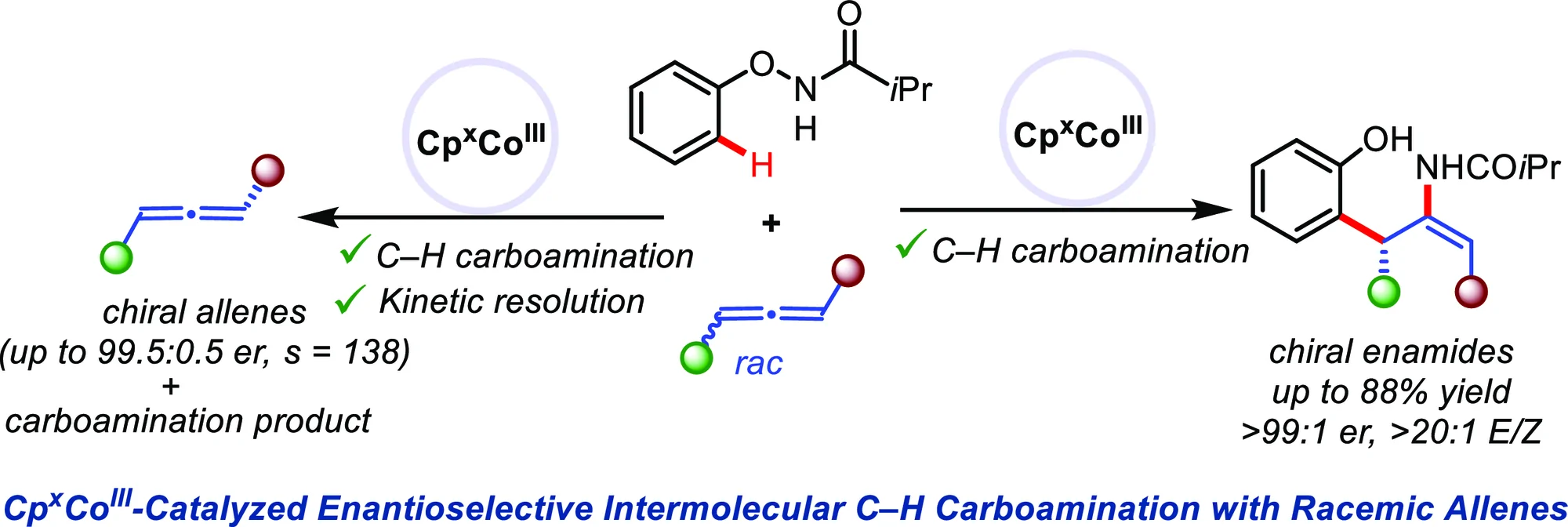

## Introduction

Carboamination is a pivotal synthetic transformation that enables the simultaneous formation of a carbon–carbon and a carbon–nitrogen bond across π-compounds, thereby generating desirable molecular complexity in a single step.^1–3^ Achieving this transformation in an intermolecular enantioselective manner through transition metal-catalyzed C–H functionalization offers an efficient and atom-economical approach, making it an attractive strategy in stereoselective synthesis.^4^ Substantial advancements have been made in enantioselective intermolecular C–H carboamination of alkenes, acrylates, conjugated 1,3-dienes, and alkynes, particularly by using rhodium-catalyzed C–H functionalization (Scheme 1B).^5^ However, related technologies for the asymmetric C–H carboamination of cumulated dienes, such as allenes, remain largely unexplored. Although allenes have been shown as competent coupling partners in C–H functionalization reactions,^6,7^ the enantioselective carboamination of allenes remains an unmet challenge. Compared to carboamination of olefins,^5^ the carboamination of allenes poses several key challenges. First, the selective addition of concurrent carbon and nitrogen fragments across the two orthogonal π-bonds of an unsymmetrical 1,3-disubstituted allene requires precise control over regio-, chemo-, and stereoselectivity (Scheme 1C). A tight control of competing processes such as annulation, proto-demetalation, or β-hydride elimination during the transformation further complicates achieving selectivity.^6^ In addition to selectivity concerns, employing racemic allenes with built-in axial chirality as coupling partners introduces two distinct mechanistic challenges. First, attack on the central carbon atom of the allene would lead to the metal-π-allyl intermediate and both enantiomers converge to forming a chiral allyl amine via a dynamic kinetic asymmetric transformation (DYKAT) (Scheme 1D, path A).^7b,8^ Alternatively, an attack on either termini of the allene would form a metal-alkenyl intermediate.^9^ In this case, a kinetic resolution process can be envisioned where the matching enantiomer of the allene may preferentially react, leaving the disfavored enantiomer untouched (Scheme 1D, path B). The formation of either metal-π-allyl or metal-alkenyl intermediates is influenced by the electronic and steric profile of the allenes, the nature of the substrate-directing group, and the choice of the metal chiral catalyst. Besides a Rh^III^-catalyzed achiral intermolecular C–H carboamination of achiral trisubstituted sulfonyl allenes with *N*-phenoxy amides resulting in allylic amines,^10^ other reactions—especially enantioselective C–H carboamination of allenes—have not been documented to date. In recent years, earth-abundant 3d-metal cobalt-catalyzed asymmetric C–H functionalizations have emerged as alternatives to precious-metal Rh^III^- and Ir^III^-based methods.^11,12^ In this respect, high-valent cyclopentadienyl Co^III^-complexes^13–15^ display remarkable selectivities that complement the reactivities of Rh^III^-complexes and have also enabled access to uncharted reactivity opportunities.^13b,13c^ Given the current dearth of C–H carboamination methods for allenes, we sought to develop cobalt-catalyzed enantioselective intermolecular C–H carboamination of racemic 1,3-disubstituted allenes. Hence, the application of chiral cyclopentadienyl cobalt-catalyzed C–H functionalizations to address previously inaccessible asymmetric C–H carboamination of allenes represents a significant research advance.

**Scheme 1:**
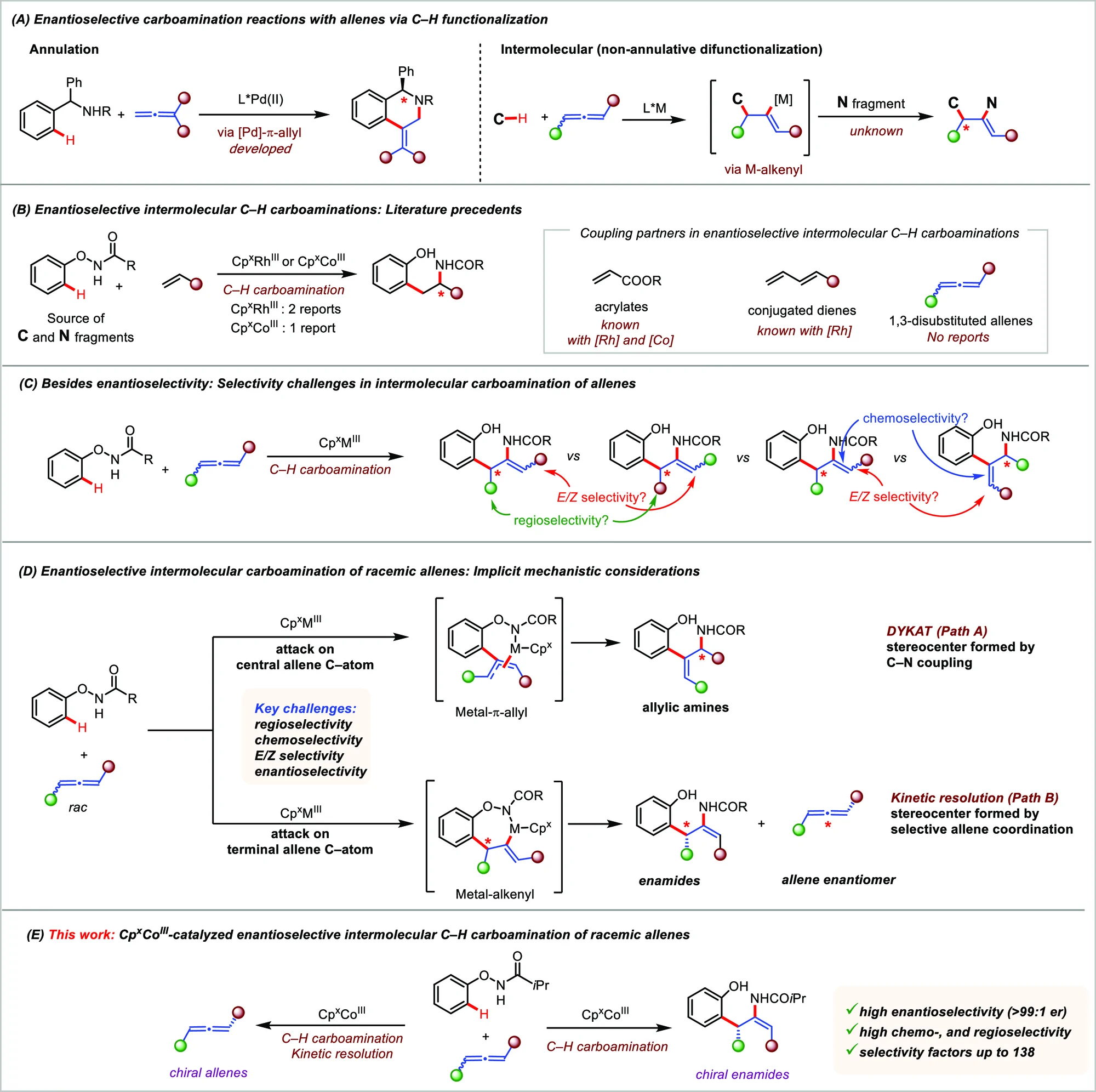
Transition Metal-Catalyzed Enantioselective C–H Carboaminations with Allenes

Herein, we report a Cp^x^Co^III^-catalyzed highly regio-, chemo-, and enantioselective intermolecular C–H carboamination of racemic allenyl esters with *N*-phenoxyisobutyramides (Scheme 1E). The developed protocol allows for rapid access to versatile chiral γ-substituted-β-enamido esters with excellent enantioselectivities (>99:1 er) and *E/Z* selectivity (>20:1). Advantageously, the resulting products can be diastereoselectively reduced under catalyst control to β-amino esters with two contiguous stereocenters. The process operating by a kinetic resolution can be optimized for allene recovery affording enantioenriched allenes with selectivity factors of up to 138.

## Results and Discussion

### Reaction Optimization

We commenced the optimization of C–H carboamination by using *N*-phenoxyisobutyramide **1a** as a source of both carbon and nitrogen fragments and *rac*-allenyl ester **2a** as model substrates (Table 1). Silver hexafluoroantimonate was used as a halide abstractor, and a combination of cesium pivalate and potassium phosphate was employed as a base. In a preliminary reaction scouting using Cp*Co­(CO)­I_2_ in TFE solvent at 23 °C afforded the carboamination product **3aa** in 36% yield as a single regioisomer (entry 1). When the reaction was performed with (Cp*RhCl_2_)_2_ as the catalyst, **3aa** was obtained in 23% yield (entry 2), indicating a reactivity preference for the cobalt catalyst. Catalyst **Co1**, featuring a disubstituted chiral Cp^x^ ligand, was completely ineffective and did not produce **3aa** (entry 3). In sharp contrast, the trisubstituted Cp^x^Co^III^ complex **Co2** (*R* = *i*Pr) yielded **3aa** in 43% yield with excellent enantioselectivity of 99:1 er (entry 4). The bulkier version **Co3** (*t*Bu instead of *i*Pr) provided **3aa** in 45% yield with an even higher of >99:1 er (entry 5). Similarly, catalyst **Co4** delivered **3aa** with an improved yield of 53%, maintaining the outstanding selectivity (entry 6). With **Co4**, switching to HFIP as a solvent increased the yield of **3aa** to 68% with >99:1 er (entry 7). Screening alternative halide scavengers AgOTf, AgBF_4_, and AgPF_6_ did not substantially affect the reaction outcome (entries 8–10). Using AgPF_6_ as a halide scavenger, a higher concentration of 0.4 M gave the yield of **3aa** to 67% (entry 11). Replacing CsOPiv by Bu_4_NOPiv improved the yield of **3aa** to 76% (entry 12), presumably due to the higher solubility of Bu_4_NOPiv. Increasing the stoichiometry of AgPF_6_ from 20 mol % to 25 mol % further improved the yield of **3aa** to 80% (entry 13). Finally, increasing the amount of allenyl ester *rac*-**2a** gave **3aa** in 87% as a single double bond isomer with >99:1 er (entry 14).

**Table 1:**
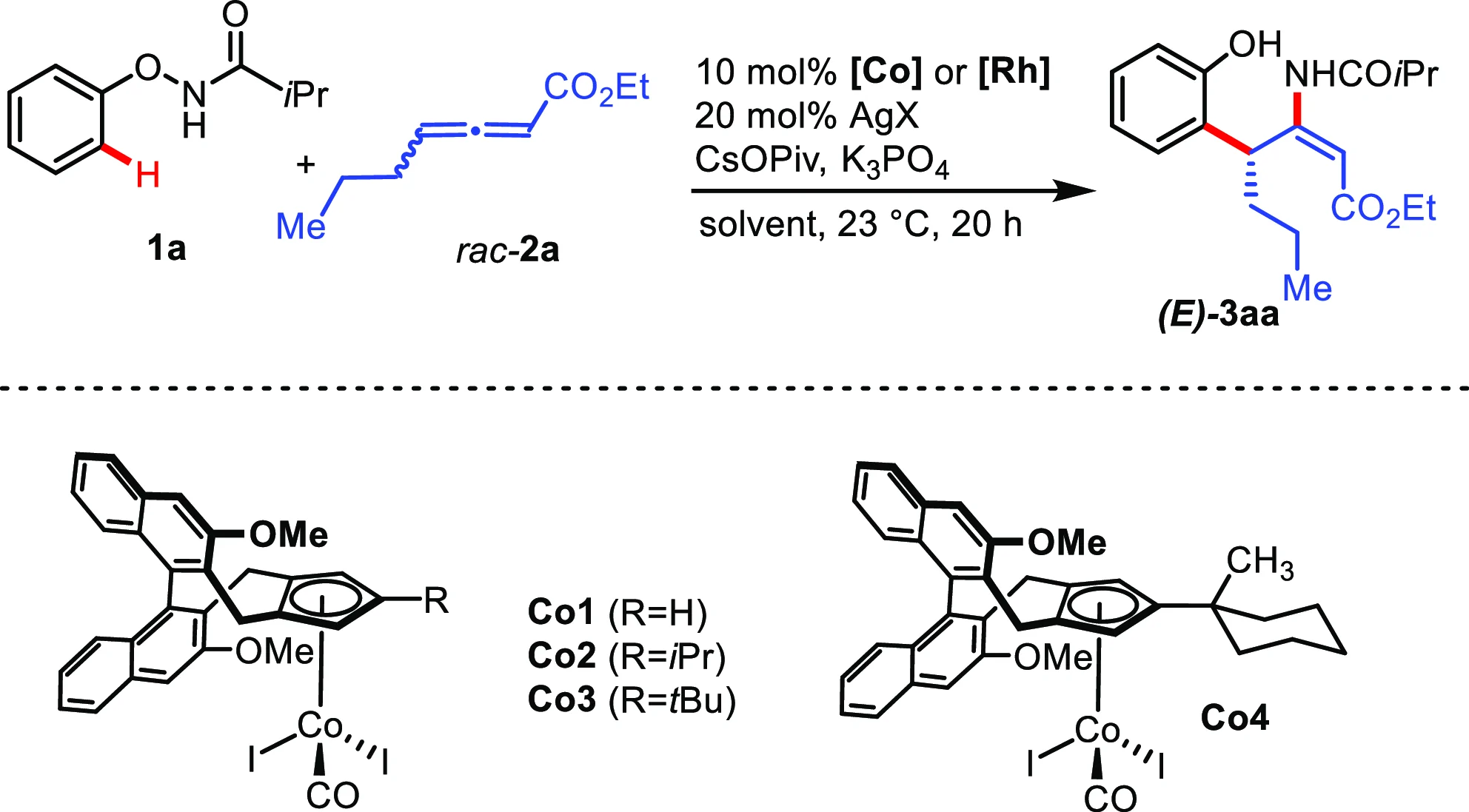
Optimization of C–H Carboamination of Allenes^a^

entry	[Co]	AgX	solvent	3aa (%)	er
1	Cp*Co	AgSbF_6_	TFE	36	-
2	Cp*Rh	AgSbF_6_	TFE	23	-
3	**Co1**	AgSbF_6_	TFE	-	-
4	**Co2**	AgSbF_6_	TFE	43	99:1
5	**Co3**	AgSbF_6_	TFE	45	99.5:0.5
6	**Co4**	AgSbF_6_	TFE	53	99.5:0.5
7	**Co4**	AgSbF_6_	HFIP	68	99.5:0.5
8	**Co4**	AgOTf	HFIP	68	99.5:0.5
9	**Co4**	AgBF_4_	HFIP	70	99.5:0.5
10	**Co4**	AgPF_6_	HFIP	71	99.5:0.5
11^b^	**Co4**	AgPF_6_	HFIP	67	99.5:0.5
12^b^,^c^	**Co4**	AgPF_6_	HFIP	76	99.5:0.5
13^b^,^c^	**Co4**	AgPF_6_ (25 mol %)	HFIP	80	99.5:0.5
14^b^,^c^,^d^	**Co4**	AgPF_6_ (25 mol %)	HFIP	87 (85)^e^	99.5:0.5

a Conditions: 0.05 mmol **1a**, 0.1 mmol **2a**, 10 mol % [**Co**], 20 mol % AgX, 0.025 mmol CsOPiv, 0.025 mmol K_3_PO_4_ in solvent (0.2 M) at 23 °C for 20 h. Yields were determined by ^1^H NMR using 1,3,5-trimethoxybenzene as an internal standard.

b 0.4 M HFIP.

c 0.025 mmol of Bu_4_NOPiv was used instead of CsOPiv.

d 0.15 mmol of **2a**.

e 2-fold scale-up, 0.1 mmol of **1a**, 0.3 mmol of **2a**, isolated yield.

### Substrate Scope

With optimized C–H carboamination conditions, the generality of the reaction was studied next (Scheme 2). As such, varying the electronic nature of substituents at the *para*-position of phenoxyisobutyramides **1x** did not affect the reactivity or selectivity of the reaction. Both electron-donating (Me, *t*Bu) and electron-withdrawing (CF_3_, CO_2_Me) groups are compatible with the transformation and delivered the corresponding carboaminated phenols **3ba**–**3fa** in good yields and enantioselectivities exceeding 99:1 er. Phenoxyisobutyramides bearing *para*-halogen substitution (**1g**–**1i**) were tolerated and furnished the corresponding products **3ga**–**3ia** with similar high enantioselectivity. Phenoxyisobutyramides bearing *meta*-substituents **1j**,**1k** were reacted with allene **2a**, affording the desired carboamination products **3ja**,**3ka** exclusively as single regioisomers. *Ortho*-fluoro-substituted phenoxyisobutyramide **1l** reacted smoothly with allene **2a** to give product **3la** in 82% yield and 99:1 er. *Ortho*-methyl-substituted phenoxyisobutyramide **1m** showed sluggish reactivity and gave the product **3ma** in reduced yield, albeit a consistent high selectivity 99:1 er. Using 3,4-dimethyl-substituted phenoxyisobutyramide **1n**, corresponding phenol **3na** was isolated in 72% yield and 99.5:0.5 er. Naphthyloxyisobutyramide **1o** gave naphthol **3oa** without compromising enantioselectivity. A fully diastereoselective carboamination of tyrosine derivative **1p** with allene **2a** proceeded successfully, and product **3pa** was obtained in 79% yield with an exquisite diastereoselectivity (99.5:0.5). Employing the opposite enantiomer of the chiral cobalt catalyst (*ent*-**Co4**), the other *dia*-**3pa** was obtained with excellent diastereoselectivity (99.5:0.5) evidencing a full catalyst control of the transformation. In general, for substrates **1a**–**1p**, the carboamination products were obtained in high yields, with high enantioselectivity (>99:1 er) and *E/Z* selectivity (>20:1). Noteworthily, switching the isobutyramide group to a carbamate reversed the *E/Z* selectivity and resulted in the *Z*-selective carboamination product as a major product. Cbz-protected phenoxyamine **1q** gave corresponding Cbz-bearing compound **3qa** with a *Z/E* ratio 3.3:1. The selectivity switch of the reaction from *E* to *Z* did not affect the enantioselectivity, and **3qa** was obtained with 99:1 er. Methyl phenoxycarbamate **1r** provided product **3ra** with a *Z/E* ratio of 3:1 and similarly high selectivity. In addition, Fmoc-carbamate **1s** reacted in an analogous manner providing Fmoc-protected enamide **3sa** (*Z/E* = 5.2:1) in high enantioselectivity. Next, we tested the reactivity of a diverse set of allenes with our C–H carboamination. Substituting the ethyl ester of the allene with either *tert*-butyl (**2b**) or benzyl ester (**2c**) had no influence on the reaction outcome. Allenamide **2d** participated in the reaction, affording **3ad** with 99.5:0.5 er. Noteworthily, allenylphosphonate **2e** underwent reaction smoothly, furnishing vinylphosphonate **3ae** in 71% yield with 98.5:1.5 er. Allenyl esters bearing methyl (**2f**), longer alkyl chain (C_8_H_17_) (**2g**), phenethyl (**2h**), and benzyl group (**2i**) substitution were compatible, providing the corresponding products **3af–3ai** consistently in high yields and enantioselectivities. Phenyl-substituted allenyl ester (**2j**) resulted in diminished yields of **3aj** with 99:1 er, likely due to steric hindrance of the phenyl group. A moderate yield was as well observed for cyclopropyl-substituted allenyl ester (**2k**), giving 40% yield and 99.5:0.5 er. Further concerning functional group compatibilities, allenyl ester containing alkyl ester (**2l**), alkoxy ether (**2m**), alkyl chloride (**2n**), and alkyl phthalimide (**2o**) functionalities at the γ-position were successfully subjected to C–H carboamination. Single-crystal X-ray crystallographic analysis of **3ao** allowed the determination of the absolute configuration to be (*R*) and the double bond geometry to be (*E*).^16a^ Allenyl ester **2p** having an additional terminal olefin moiety that potentially engages with the cyclometalated intermediate reacted exclusively at the allene functionality. No carboamination of the terminal olefin was observed.

**Scheme 2:**
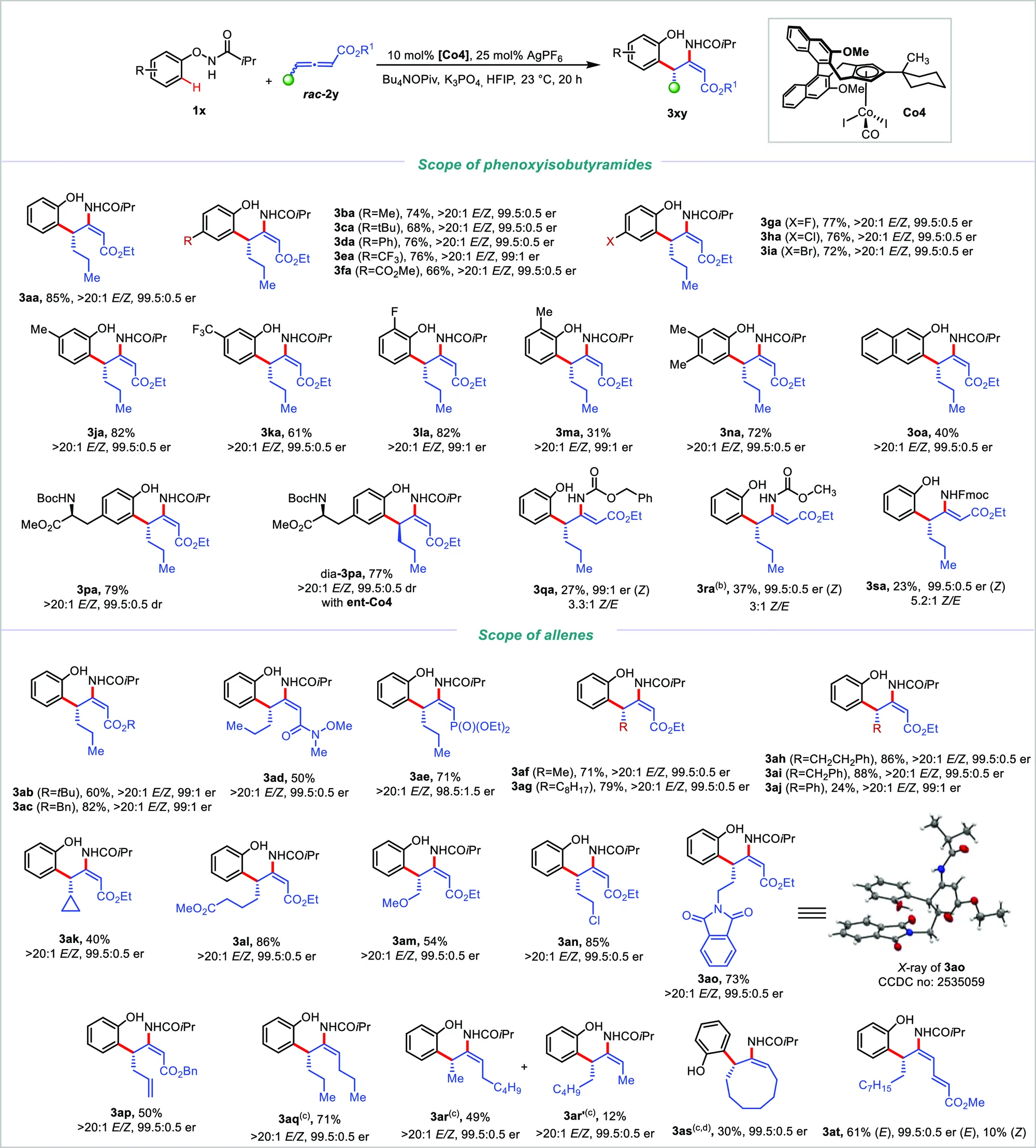
Scope for the Cp^x^Co^III^-Catalyzed Enantioselective Intermolecular Carboamination of Racemic Allenes^a^

a Conditions: 0.1 mmol **1x**, 0.3 mmol **2y**, 10 mol % **Co4**, 25 mol % AgPF_6_, 0.05 mmol Bu_4_NOPiv, 0.05 mmol K_3_PO_4_ in HFIP (0.4 M) at 23 °C for 20 h. Isolated yields.
b With 20 mol % **Co4** and 50 mol % AgPF_6_.
c With 10 mol % **Co4**, 20 mol % AgSbF_6_, 0.025 mmol CsOPiv, 0.05 mmol K_3_PO_4_ in TFE/DCE (9:1) (0.2 M) at 23 °C for 20 h.
d With 20 mol % **Co4** and 40 mol % AgSbF_6_.

Using slightly modified reaction conditions, 1,3-dipropylallene **2q** also underwent reaction, delivering the corresponding carboamination product **3aq** in 71% yield and 99.5:0.5 er. Subsequently, we investigated the unsymmetrical, dialkyl-substituted 1-methyl-3-pentyl allene (**2r**), which presents a significant challenge due to the absence of electronic bias. The reaction proceeded giving a synthetically useful 3.9:1 mixture of regioisomeric products **3ar** and **3ar′** in 61% combined yield with excellent enantioselectivity for both compounds. The major regio-isomer (**3ar**) is formed preferentially via functionalization of the less sterically hindered methyl-substituted allenic double bond. Moreover, cyclic racemic allene **2s** engaged in reaction. Finally, subjecting beetle pheromone **2t** featuring allene functionality to the reaction conditions yielded the corresponding product **3at** in 61% (*E*) yield with 99.5:0.5 er.

### Proposed Reaction Mechanism and Mechanistic Investigations

A plausible catalytic cycle for C–H carboamination of allenes is illustrated in Scheme 3. The reaction is initiated by coordination of the cobalt catalyst to the substrate nitrogen atom of **1a**. A CMD-style *ortho*-C–H activation of *N*-phenoxyisobutyramide gives cobaltacycle **I**. There are two possible scenarios leading to the observed enantioselectivity of the transformation. In pathway A, intermediate **I** strongly discriminates between the allene enantiomers. Almost exclusively, the matching enantiomer **2a** engages in coordination and subsequent migratory insertion at the most reactive terminal carbon atom of the allene, leading to Co-alkenyl intermediate **II**. The regio- as well as *E/Z*-selectivity is determined in this insertion, favoring an *E*-geometry and placement of cobalt distal to the ester. Ensuing reductive elimination and subsequent oxidative addition to N–O bond generates intermediate **III**. Finally, protonation of the cobalt–nitrogen bond releases product **3aa** and regenerates the cobalt catalyst. In this scenario, the mismatched enantiomer of **2a** enriches during the reaction. Alternatively, in situ racemization of the allene could occur under the reaction conditions. Due to continuous racemization, a suitable concentration of the reactive matching allene enantiomer is maintained throughout the process. This pathway B leads to dynamic kinetic resolution mechanism, allowing for the complete conversion of racemic allene into the carboamination product. To gain mechanistic insights on the operative allene incorporation mechanics and to determine whether carboamination proceeds via a kinetic resolution or a dynamic kinetic resolution, a small set of control experiments was conducted. When two equivalents of racemic allene **2a** were used in carboamination with phenoxyisobutyramide **1a**, we recovered 42% of unreacted allene **2a**, besides carboamination product **3aa** (40% yield and 99.5:0.5 er) after 20 h. The optical purity of recovered allene **2a** was measured at 92:8 er. Such optical enrichment of allene **2a** agrees with a kinetic resolution process pathway A. Encouraged by this result, further reaction optimization using 0.6 equiv of **1a** resulted in 40% recovery of allene **2a** with an improved selectivity of 97.5:2.5 er. In a racemization study, enantio-enriched (*S*)-**2a** was separately exposed to **[Co4]** and *ent-* **[Co4]** under standard reaction conditions in the absence of substrate **1a**. In both cases, only minimal erosion of the optical purity of (*S*)-**2a** was observed, and the allene was recovered in 65% and 57% yields, respectively. These results speak against dynamic kinetic resolution pathway B and confirm that the reaction proceeds via kinetic resolution pathway A.

**Scheme 3:**
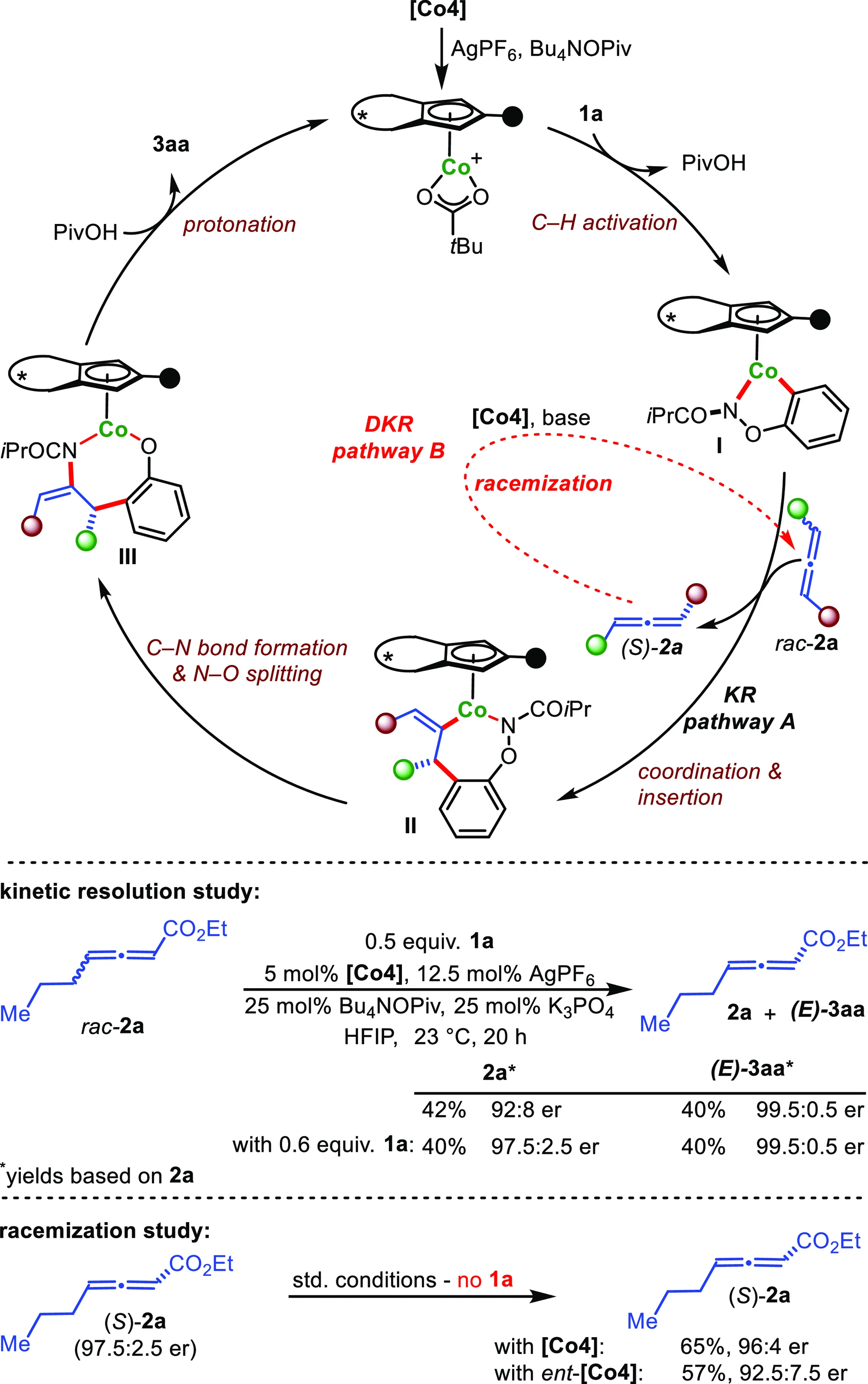
Mechanistic Rationale for the Kinetic Resolution, Dynamic Kinetic Resolution Pathways, and Control Experiments

### Kinetic Resolution of Allenes via C–H Carboamination

To take advantage of the process, we further investigated the kinetic resolution of allenes. Although methods for the kinetic resolution of allenes are reported,^17^ to our knowledge, the use of C–H functionalization technology has not been used for such purposes. Without doubt, chiral allenes serve as versatile precursors for the synthesis of complex molecular scaffolds as well as signature functional groups of numerous bioactive natural products.^18^ Owing to synthetic utility of chiral allenes, we applied our modified C–H carboamination methodology for the resolution and recovery of allenes (Scheme 4). Subjecting allenyl ethyl ester (**2a**) to kinetic resolution conditions, chiral allenyl ester (*S*)-**2a** was recovered in 40% yield with 97.5:2.5 er, which corresponds to a selectivity factor of 22. A switch to benzyl ester substrate **2c** resulted in a slight reduction in er for the recovered allene. Allene phosphonate **2e** was resolved with an excellent selectivity factor of 138. Allenyl esters bearing phenethyl (**2h**) and benzyl group (**2i**) substituents were recovered in 41% and 40% yields with 95:5 er and 96.4 er, respectively. Allene **2l** with an alkyl ester substituent was recovered in 99.5:0.5 er, corresponding to an s-factor of 116. Alkyl chloride (**2n**) and phthalimide (**2o**) substituted allenic esters were recovered with 97:3 er and 93.5:6.5 er, respectively. The absolute configuration of the recovered allenes was assigned as *S* by comparing their optical rotation data with that of the known chiral allene **2c**. To illustrate synthetic utility, the method was used to the resolve racemic allenic ester *rac*-**2t** to methyl (−)-2,4,5-tetradecatrienoate (*R*,*E*)-**2t**, the sex pheromone of the male dried bean weevil (*Acanthoscelides obtectus*).^19^ Subjecting *rac*-**2t** to the standard kinetic resolution conditions using the **Co4** catalyst afforded the unnatural enantiomer (*S,E*)-(+)-**2t** in 33% yield with an excellent selectivity of 99:1 er. Conducting the reaction with *ent*-**Co4** as the catalyst provided the natural enantiomer of the insect pheromone (*R,E*)-(−)-**2t** in 34% yield and 97.5:2.5 er. The absolute configuration of both resolved pheromones was confirmed by comparison of their optical rotations with literature values for (*R,E*)-(−)-**2t.** ^19a^

**Scheme 4:**
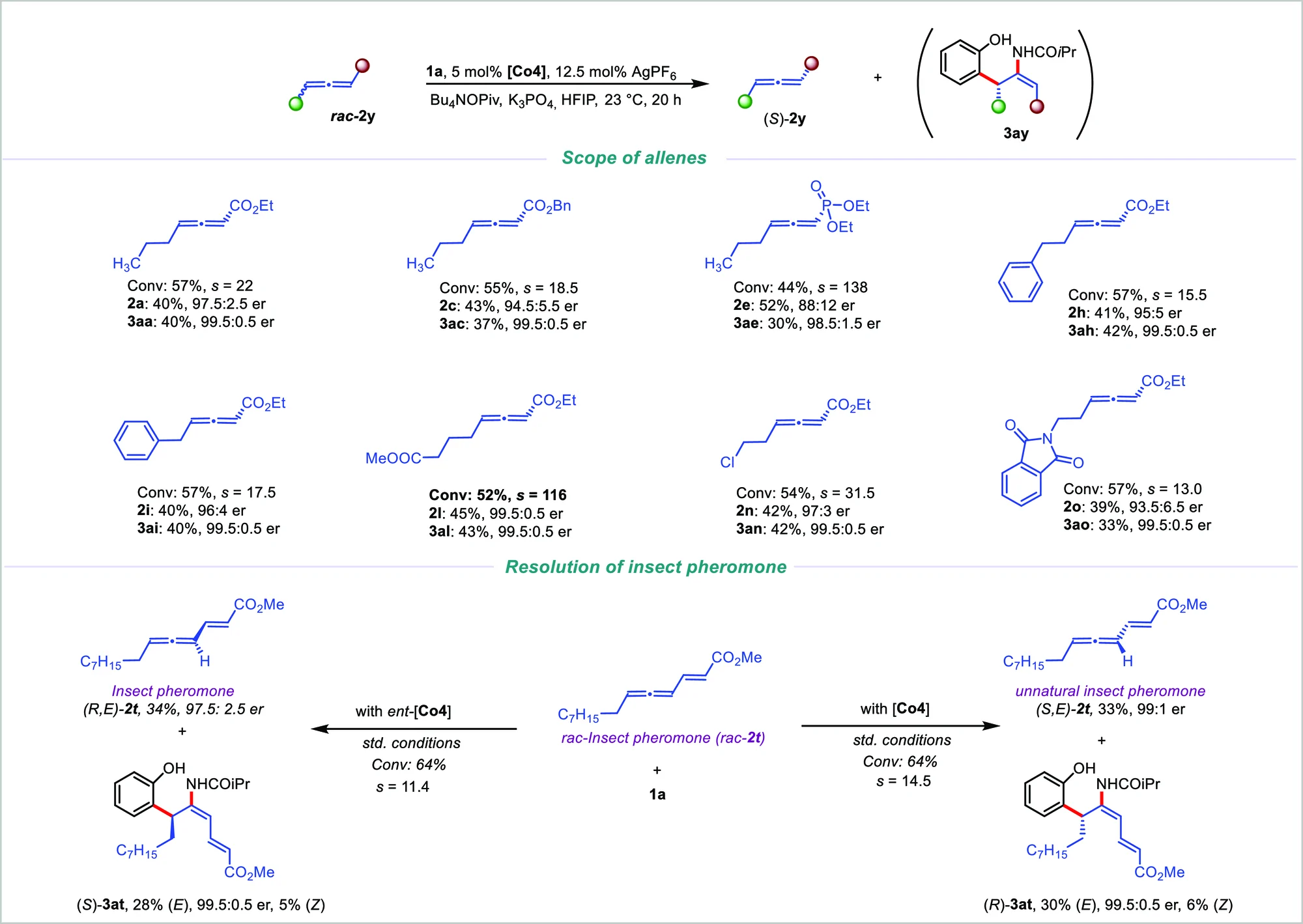
Kinetic Resolution of Racemic-Allenes by Cp^x^Co^III^-Catalyzed C–H Carboamination^a^

a Conditions: 0.2 mmol **2y**, 0.12 mmol **1a**, 5 mol % **Co4**, 12.5 mol % AgPF_6_, 0.025 mmol Bu_4_NOPiv, 0.025 mmol K_3_PO_4_ in HFIP (0.8 M) at 23 °C for 20 h. Isolated yields. Conversion was determined by crude ^1^H NMR analysis. Enantiomeric ratios were determined by chiral HPLC. Selectivity factor *s* = ln­[(1 – conv)­(1 – ee)]/ln­[(1 – conv)­(1+ ee)], ee = ee of the recovered allene.

### Product Derivatization and Synthetic Utility

Next, the synthetic utility of the carboamination products was studied. A diastereoselective hydrogenation of these β-enamido esters would provide direct access to synthetically valuable chiral β-amino acid derivatives possessing two contiguous stereocenters.^20^ Applying a rhodium-monodentate phosphoramidite hydrogenation method^21^ using Rh­(COD)_2_BF_4_ and the phosphoramidite ligand (*S*)-**5** to **3aa** gave β-amino ester **4a** in 69% yield as a single diastereomer (Scheme 5). Notably, usage of (*R*)-**5** gave an alternate diastereomer **4b** with an excellent diastereoselectivity of >20:1, evidencing a fully catalyst control of the selectivity. The efficient hydrogenation method was effective for selected substrates furnishing corresponding β-amino esters **4c**–**4e** in 71–82% yields and with full diastereoselectivities (>20:1). The absolute configuration of the newly formed stereocenter was assigned to be (*S*) by single-crystal X-ray crystallographic analysis of **4d**.^16b^ To further demonstrate synthetic practicality and to additionally take advantage of the free phenol hydroxy group formed during the transformation, an intramolecular cyclization of **3an** (99.5:0.5 er) provided 4-substituted chromane **6** in 67% yield without any erosion of enantioselectivity (Scheme 5).

**Scheme 5:**
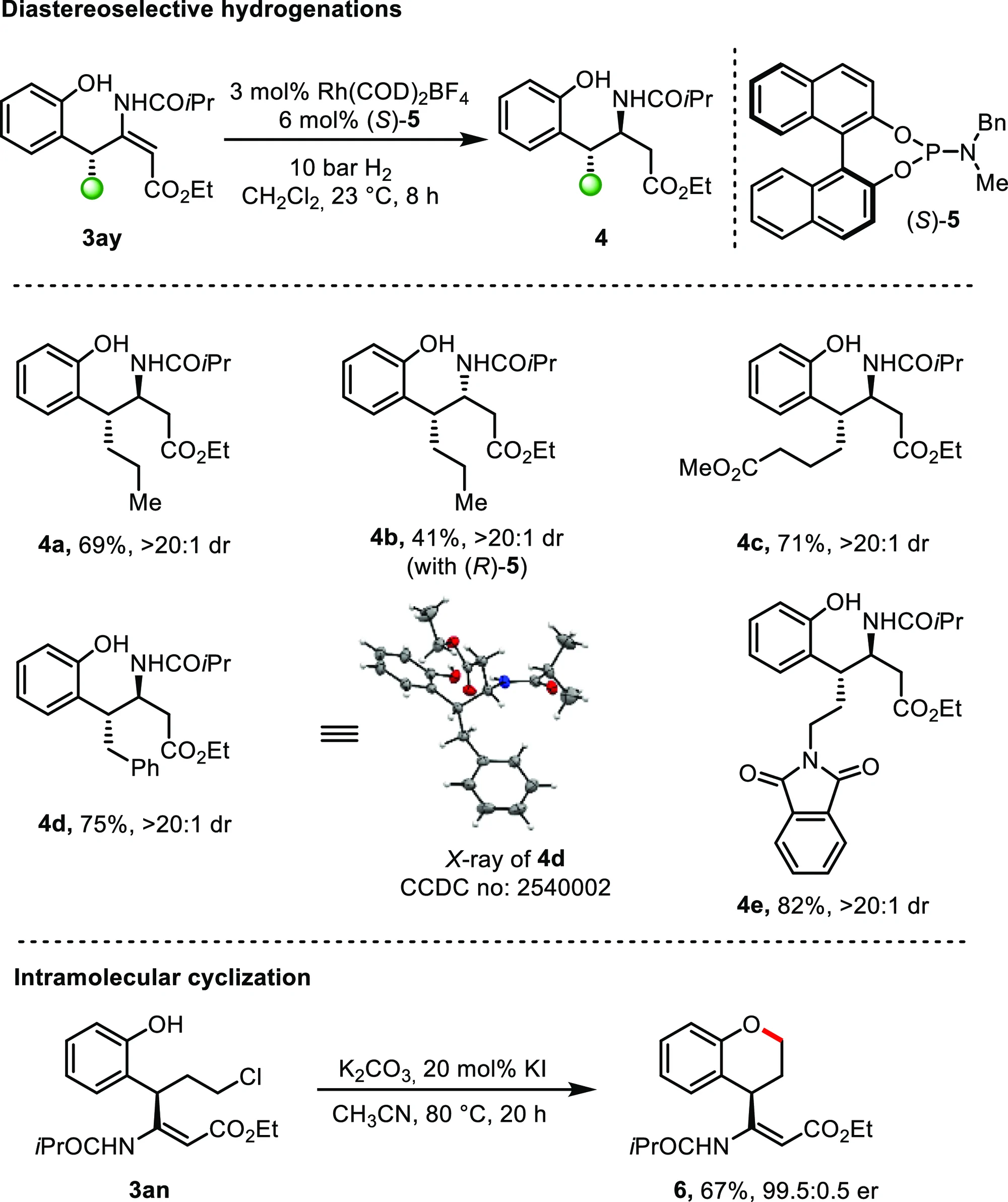
Access to β-Amino Acid Derivatives and Chromanes from Carboamination Products

## Conclusion

In summary, we have developed an efficient Cp^x^Co^III^-catalyzed highly regio-, chemo-, and enantioselective intermolecular C–H carboamination of racemic allenes. In this protocol, phenoxyisobutyramides were utilized as sources of both carbon and nitrogen fragments. A broad range of phenoxyisobutyramides with diverse electronic substituents are compatible with the carboamination process. The transformation provides direct access to synthetically important chiral γ-substituted-β-enamido esters with exceptional selectivities (>99:1 er and >20:1 *E/Z*) convertible to β-amino acid derivatives with two contiguous stereocenters. This reaction protocol is amenable to a diverse array of allenes, including allenamides, allene phosphonates, dialkyl-substituted, as well as cyclic allenes. Moreover, the reaction was extended to a kinetic resolution pathway, affording enantioenriched allenes with up to 99.5:0.5 er and s-factors up to 138. The resolution was exploited to access both enantiomers of methyl-tetradeca-trans-2,4,5-trienoate, the male bean weevil (*Acanthoscelides obtectus*) pheromone.

## Experimental Section

All experimental procedures, catalyst preparation protocols, reaction optimization details, characterization data, and stereochemical assignments are provided in the Supporting Information.

## Supplementary Material



## Notes

Synthetic procedures, characterization data for all new compounds, and crystallographic data for **3ao** and **4d** (PDF)
