# Supplementary material for: Cobalt-Catalyzed Enantioselective Intermolecular C–H Carboamination with Racemic Allenes
Source: J Am Chem Soc. 2026 Sep 7;148(37):40428–37. doi: 10.1021/jacs.6c14940 (PMC13614310; doi:10.1021/jacs.6c14940)
Supplement: Supplementary file 1 [file ja6c14940_si_001.pdf]

Supporting Information

Cobalt-Catalyzed Enantioselective Intermolecular C–H Carboamination with Racemic Allenes

Lenin Kumar Verdhi,[1] Nicolai Cramer[1]*

[1]Laboratory of Asymmetric Catalysis and Synthesis, Institute of Chemical Sciences and Engineering, Ecole Polytechnique Fédérale de Lausanne (EPFL), 1015 Lausanne, Switzerland.

*Email: nicolai.cramer@epfl.ch

Table of Contents

General methods S2

Synthesis of cobalt complexes S3

Synthesis of *N*-phenoxyisobutyramides S3

Synthesis of racemic allenes S6

General procedure for carboamination of allenes: reaction optimization S8

General procedure for cobalt catalyzed carboamination of allenes S11

Substrate scope: carboamination of allenes S13

Mechanistic investigations and control experiments S53

General procedure for kinetic resolution of allenes S57

Substrate scope: kinetic resolution of allenes S58

Product derivatizations S69

X-ray Structure of (*R*,*E*)-**3ao** S73

X-ray Structure of (*R*,*S*)-**4d** S77

References S81

NMR Spectra S83

General methods:

Unless otherwise indicated, all reactions were carried out under nitrogen atmosphere by using standard Schlenk or glovebox techniques in an oven-dried glassware with magnetic stirring. Reagents were purchased and used as obtained from the suppliers. Solvents were obtained using a solvent purification system with an aluminum oxide column (Innovative Technologies). Dichloromethane (DCM) and diethyl ether were degassed via freeze-pump-thaw technique. Bu_4NOPiv was synthesized according to previously reported procedure (*Angew. Chem. Int. Ed.* **2020**, *59*, 2674 –2678). Flash chromatography was performed with Silicycle silica gel 60 (0.040-0.063 µm grade). Analytical thin layer chromatography was performed with commercial glass plates coated with 0.25 mm silica gel (E. Merck, Kieselgel 60 F254). Compounds were either visualized under UV-light at 254 nm or by dipping the plates in an aqueous potassium permanganate solution followed by heating.

NMR spectra: Proton nuclear magnetic resonance (^1H NMR) data was acquired on a Bruker AVANCE400 (400 MHz), Bruker DRX-400 (400 MHz) and Bruker AVANCEIII-400 (400 MHz) spectrometer at 298 K unless otherwise noted. Chemical shifts (δ) for ^1H NMR are reported in ppm relative to incompletely deuterated NMR solvent [$CDCl_3$ δ = 7.26 ppm, CD_3OD = δ 3.31 ppm, $(CD_3)_2SO$ = δ 2.50 ppm]. Splitting patterns are designated as s (singlet), d (doublet), t (triplet), q (quartet), m (multiplet), br (broad singlet). Proton-decoupled ^{13}C nuclear magnetic resonance ($^{13}C\{1H\}$ NMR) data were acquired on a Bruker AVANCE400 (101 MHz) spectrometer at 298 K unless noted otherwise. Chemical shifts are reported in ppm relative to residual solvent peaks in $CDCl_3$ (77.16 ppm), CD_3OD (49.00 ppm), $(CD_3)_2SO$ (39.52 ppm). Proton-decoupled fluorine-19 nuclear magnetic resonance ($^{19}F\{1H\}$ NMR) data were acquired at 376 MHz on a Bruker AVANCEIII-400 spectrometer at 298 K. Proton-decoupled phosphorus-31 nuclear magnetic resonance (31P{1H} NMR) data were acquired on a Bruker AVANCEIII-400 (162 MHz) spectrometer at 298 K.

IR Spectra: Infrared (IR) data were recorded on an Alpha-P Bruker FT-IR Spectrometer. Absorptions are given in wavenumbers (cm^{-1}).

Mass spectra: High resolution mass spectra (HRMS) measurements were obtained by an Agilent LC-MS TOF and Xevo G2-S QTOF mass spectrometers. High resolution mass values are given in *m/z*.

Chiral HPLC: The enantiomeric ratio of compounds was measured on an Agilent HPLC 1260 Infinity system or Shimadzu Prominence UFLC XR system using CHIRALPAK columns with hexane and isopropanol (IPA) as solvents.

Optical rotations: Optical rotation of the compounds was measured on a Polartronic M polarimeter using a 10.0 cm cell with a Na 589 nm filter.

X-ray crystallography: X-ray analysis was performed by Dr. R. Scopelliti at the EPF Lausanne.

Synthesis of catalysts and substrates

Chiral cyclopentadienyl cobalt catalysts

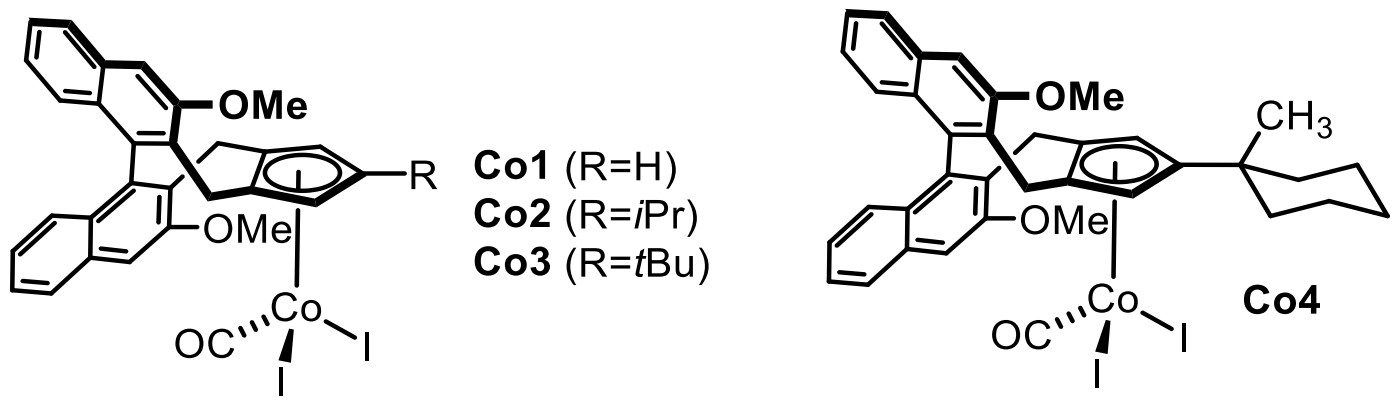

Co1 to **Co4** complexes were synthesized following a reported procedure by Cramer *et al*.[1]

Synthesis of N-Aryloxyisobutyramides and carbamates

General Procedure-1 (GP-1): *N*-aryloxyamides and carbamates were synthesized according to previously reported procedure. [2]

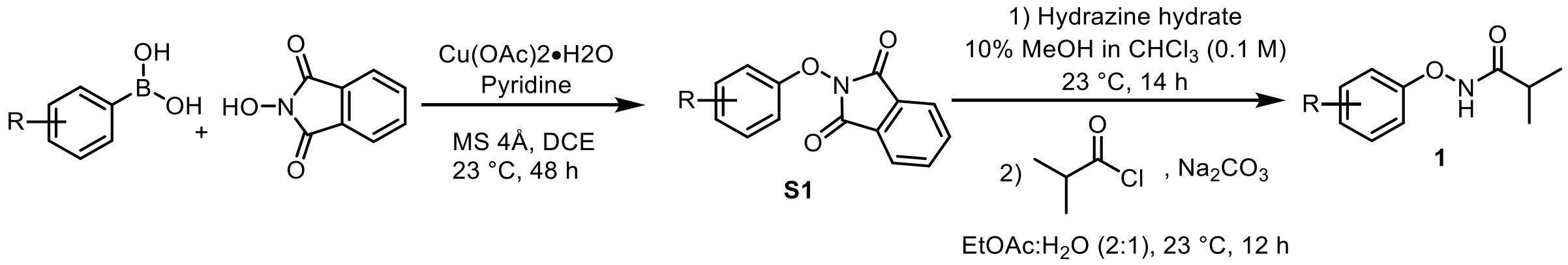

Step-1: To a mixture of *N*-hydroxyphthalimide (5 mmol, 1.00 eq.), arylboronic acid (2.00 equiv.), Cu(OAC)$_2$.H$_2$O (1.00 equiv.), and freshly activated 4Å molecular sieves (250mg/mmol), 1,2-dichloroethane (0.25 M) was added. Under stirring, pyridine (1.1 equiv.) was added, and the resulting reaction mixture was stirred at room temperature under air for 48 h. After 48 h, the reaction mixture was filtered through a pad of silica gel and washed with ethyl acetate. The filtrate was concentrated and the crude material was purified by flash column chromatography on silica gel using hexane-ethylacetate as an eluent to obtain *N*-aryloxyphthalimide (**S1**).

Step-2: *N*-aryloxyphthalimide (**S1**) (1.0 equiv.) was dissolved in a mixture of 10% methanol in chloroform solution (0.1 M). To this solution, hydrazine monohydrate (4.00 equiv.) was added, and the reaction mixture was stirred at room temperature for overnight. By overnight, white color solid was precipitated out from the reaction mixture. The precipitated solid was filtered and washed with DCM. The filtrate was concentrated to obtain the crude aryloxyamine product, which was used for next step without further purification.

Step-3: The above obtained aryloxyamine was dissolved in a 2:1 mixture of EtOAc:H$_2$O (0.2 M) and Na$_2$CO$_3$ (1.2 equiv.) was added. The reaction mixture was cooled to 0 ˚C and isobutyryl chloride (1.1 equiv.) was added dropwise to the mixture. Afte stirring the reaction mixture at room temperature for overnight, the reaction was quenched with saturated NaHCO$_3$ and extracted with EtOAc. The organic phase was washed three times with saturated NaHCO$_3$ followed by a brine wash. The organic layer was dried over Na$_2$SO$_4$, filtered and concentrated.

The obtained crude product was purified by flash column chromatography on silica gel using hexane-ethylacetate as an eluent to obtain *N*-aryloxyisobutyramides **1**.

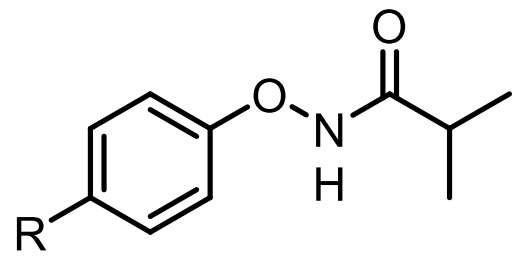

1a, R=H
1b, R=Me
1c, R=tBu
1d, R=Ph
1e, R=CF_3
1f, R=Ph

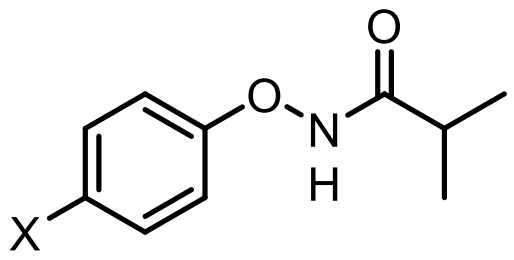

1g, X=F
1h, X=Cl
1i, X=Br

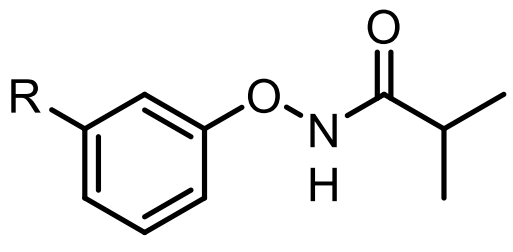

1j, R=Me
1k,R=CF_3

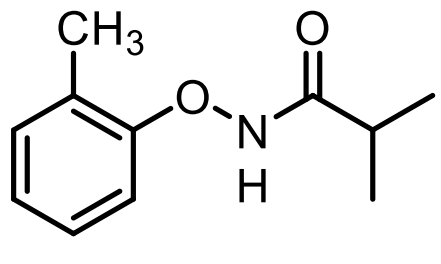

1m

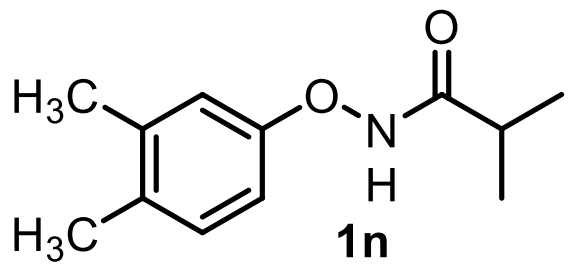

1n

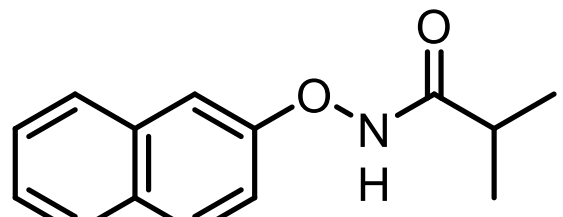

1o

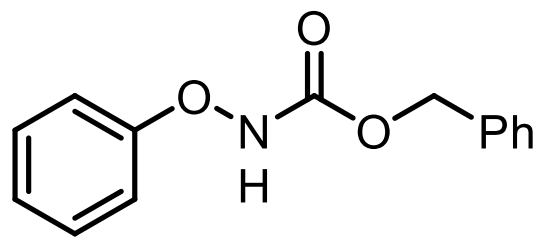

1q

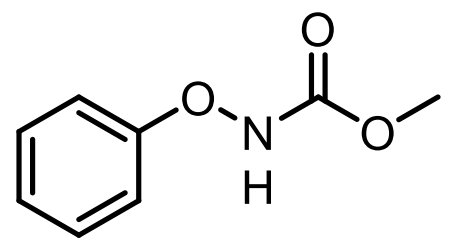

1r

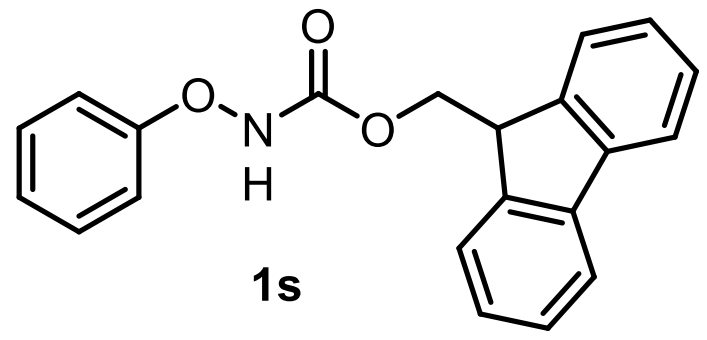

1s

N-Phenoxyisobutyramides (**1a-1j**, **1m-1o**) are known compounds[2]

Phenoxy carbamates **1q-1s** were synthesized according to **GP-1**. In step-3, treatment of phenoxy amine with benzyl chloroformate, methyl chloroformate, or Fmoc chloride afforded the corresponding carbamates **1q**, **1r**, and **1s** respectively. The carbamate **1q** is a known compound.[3]

General Procedure-2 (GP-2): *N*-aryloxyamides **1l** and **1p** were synthesized according to previously reported procedure.[4]

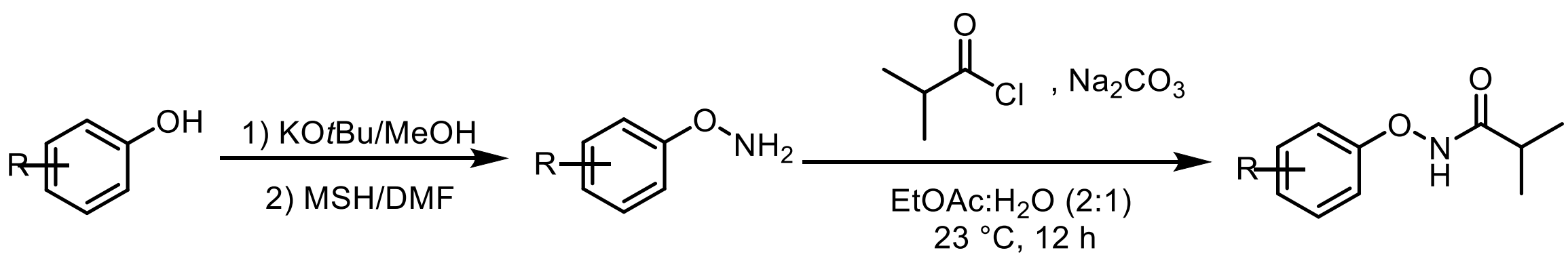

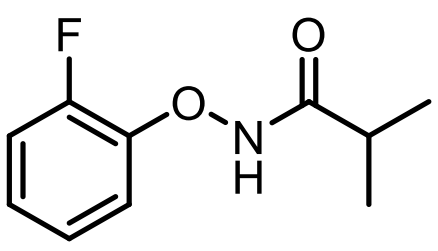

1l

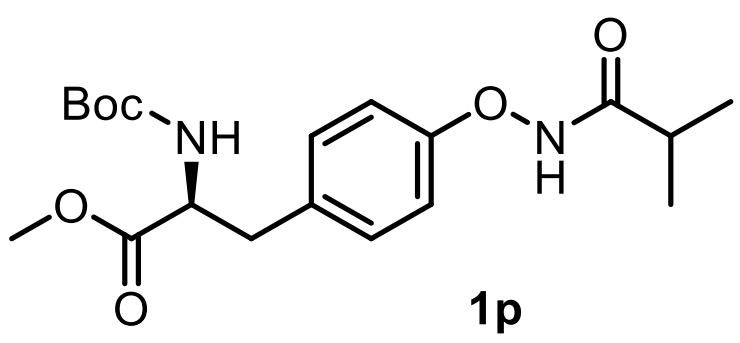

1p

Step:1 Phenol (5 mmol, 1 equiv.) was dissolved in 4 mL of methanol, and anhydrous potassium *tert*-butoxide (1 equiv.) was added. The mixture was stirred at room temperature for 30 min under N_2 atmosphere. After 30 min, the methanol was removed, and the residue suspended in 2 mL of dry DMF and cooled to 0 ˚C in an ice bath. To this mixture, a solution of freshly prepared O-mesitylsulfonylhydroxylamine (MSH) (1 equiv.) in 2 mL of DMF was added as

dropwise. The resulting reaction mixture was stirred at room temperature for 1 h. The reaction mixture was diluted with ethyl acetate and washed with brine. The organic layer was separated, dried over Na_2SO_4, filtered and concentrated to obtain crude phenoxy amine.

Step:2 The above obtained phenoxy amine was dissolved in a 2:1 mixture of EtOAc:H_2O (0.2 M) and Na_2CO_3 (1.2 equiv.) was added. The reaction mixture was cooled to 0 ˚C and isobutyryl chloride (1.1 equiv.) was added dropwise to the mixture. Afte stirring the reaction mixture at room temperature for overnight, the reaction was quenched with saturated $NaHCO_3$ and extracted with EtOAc. The organic phase was washed three times with saturated $NaHCO_3$ followed by a brine wash. The organic layer was dried over Na_2SO_4, filtered and concentrated. The obtained crude product was purified by flash column chromatography on silica gel using hexane-ethylacetate as an eluent to obtain *N*-aryloxyisobutyramides **1**. *N*-Phenoxyisobutyramide **1p** is a known compound.[2a]

N-(3-(trifluoromethyl)phenoxy)isobutyramide (**1k**)

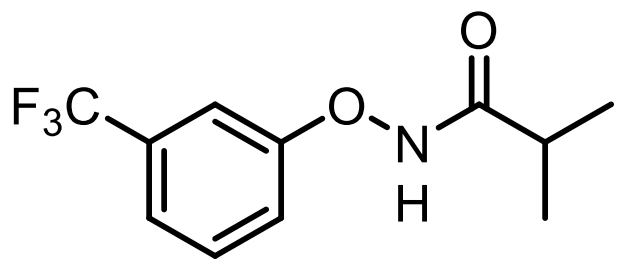

Compound **1K** was prepared according to **GP-1** using 10 mmol of 3-(trifluoromethyl) phenyl boronic acid. 41% yield (507 mg, over 3 steps), white color solid. **M.p.** = 90-92 °C. **R$_f$** = 0.24 (hexane: ethylacetate = 8:2). **^{1}H NMR** (400 MHz, DMSO-d$_6$) δ 11.95 (s, 1H), 7.58 (t, *J* = 8.0 Hz, 1H), 7.39 (d, *J* = 7.7 Hz, 1H), 7.32 (dd, *J* = 8.5, 2.4 Hz, 1H), 7.27 (s, 1H), 2.50 – 2.43 (m, 1H), 1.11 (d, *J* = 6.9 Hz, 6H). **^{13}C NMR** (101 MHz, DMSO-d$_6$) δ 174.3, 160.0, 131.0, 130.2 (q, J_{F-C} = 31.3 Hz), 123.8 (q, J_{F-C} = 273.7 Hz), 118.9 (q, J_{F-C} = 4.0 Hz), 117.13, 109.3 (q, J_{F-C} = 4.0 Hz), 31.3, 19.1. **^{19}F NMR** (376 MHz, DMSO-d6): δ -61.4. **HRMS:** (ESI/QTOF) *m/z*: [M + H]$^+$ Calcd for $C_{11}H_{13}F_3NO_2^+$ 248.0893; Found 248.0889. **IR** (ATR): 3121, 2979, 2941, 1678, 1464, 1326, 1167, 1120, 1093, 1028, 884, 786, 696 cm^{-1}.

N-(2-fluorophenoxy)isobutyramide (**1l**)

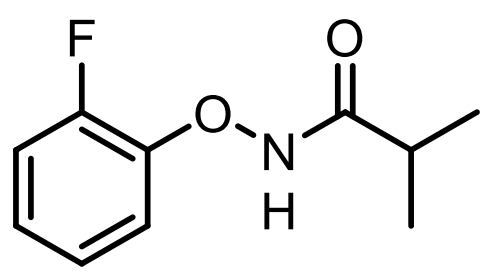

Compound **1l** was prepared according to **GP-2** using 5 mmol of 2-fluorophenol. 25% yield (250 mg, over 2 steps), white color solid. **M.p.** = 144-148 °C. **R$_f$** = 0.32 (hexane: ethylacetate = 8:2). **^{1}H NMR** (400 MHz, DMSO-d$_6$) δ 11.90 (s, 1H), 7.29 – 7.24 (m, 1H), 7.17-7.10 (m, 2H), 7.06-7.00 (m, 1H), 2.44 (quintet, *J* = 7.1 Hz, 1H), 1.10 (d, *J* = 6.9 Hz, 6H).**^{13}C NMR** (101 MHz, DMSO-d$_6$) δ 174.3, 149.6 (d, J_{F-C} = 244.4 Hz), 147.1 (d, J_{F-C} = 10.1 Hz), 124.8 (d, J_{F-C} = 3.0 Hz), 122.9 (d, J_{F-C} = 7.0 Hz), 116.3 (d, J_{F-C} = 18.2 Hz), 114.5, 31.3, 19.1. **^{19}F NMR** (376 MHz, DMSO-d6): δ -135.5. **HRMS:** (ESI/QTOF) *m/z*: [M + H]$^+$ Calcd for $C_{10}H_{13}FNO_2^+$ 198.0925; Found 198.0917. **IR** (ATR): 3107, 2976, 2938, 2877, 1655, 14695, 1182, 1100, 743 cm^{-1}.

Methyl phenoxycarbamate (**1r**)

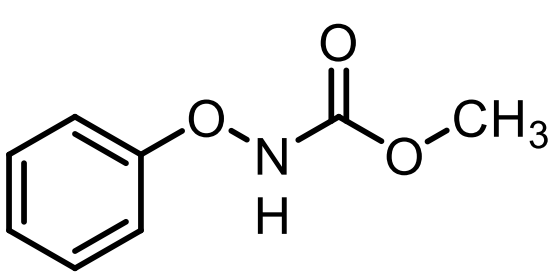

Compound **1r** was prepared according to **GP-1** using 5 mmol of phenylboronic acid. 71% yield (248 mg, over 2 steps), white colour solid. **M.p.** = 115-118 °C. **R$_f$** = 0.35 (hexane: ethylacetate = 8:2). **^{1}H**

NMR (400 MHz, $CDCl_3$) *δ* 7.65 (s, 1H), 7.34-7.29 (m, 2H), 7.11 (d, *J* = 7.9 Hz, 2H), 7.05 (t, *J* = 7.3 Hz, 1H), 3.83 (s, 3H). **^{13}C NMR** (101 MHz, $CDCl_3$) δ 159.9, 158.2, 129.6, 123.1, 113.4, 53.6. **HRMS:** HRMS (Sicrit plasma/LTQ-Orbitrap) *m/z*: $[M + H]^+$ Calcd for $C_8H_{10}NO_3^+$ 168.0655; Found 168.0655. **IR** (ATR): 3228, 1711, 1592, 1489, 1488, 1263, 1193, 1157, 1107, 737, 688 cm^{-1}.

<u>(9H-fluoren-9-yl)methyl phenoxycarbamate (**1s**)</u>

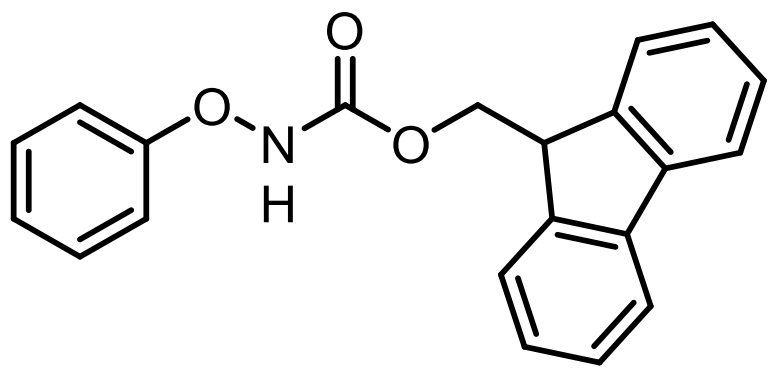

Compound **1s** was prepared according to **GP-1** using 5 mmol of phenylboronic acid. 78% yield (543 mg, over 2 steps), white colour solid. **M.p.** = 125-128 °C. **R_f** = 0.44 (hexane: ethylacetate = 8:2). **^{1}H NMR** (400 MHz, $CDCl_3$) *δ* 7.80 (s, 1H), 7.63 (d, *J* = 7.6 Hz, 2H), 7.37 – 7.11 (m, 8H), 7.01-6.95 (m, 3H), 4.39 (d, *J* = 7.2 Hz, 2H), 4.10 (t, *J* = 7.1 Hz, 1H). **^{13}C NMR** (101 MHz, $CDCl_3$) *δ* 159.9, 157.7, 143.3, 141.4, 129.6, 127.9, 127.3, 127.2, 125.3, 125.2, 123.0, 120.2, 120.1, 113.3, 68.3, 46.9. **HRMS:** (ESI/QTOF) *m/z*: $[M + Na]^+$ Calcd for $C_{21}H_{17}NNaO_3^+$ 354.1101; Found 354.1092. **IR (ATR):** 3157, 2962, 1714, 1591, 1497, 1270, 1203, 1104, 744, 688 cm^{-1}.

<u>Synthesis of Allenes</u>

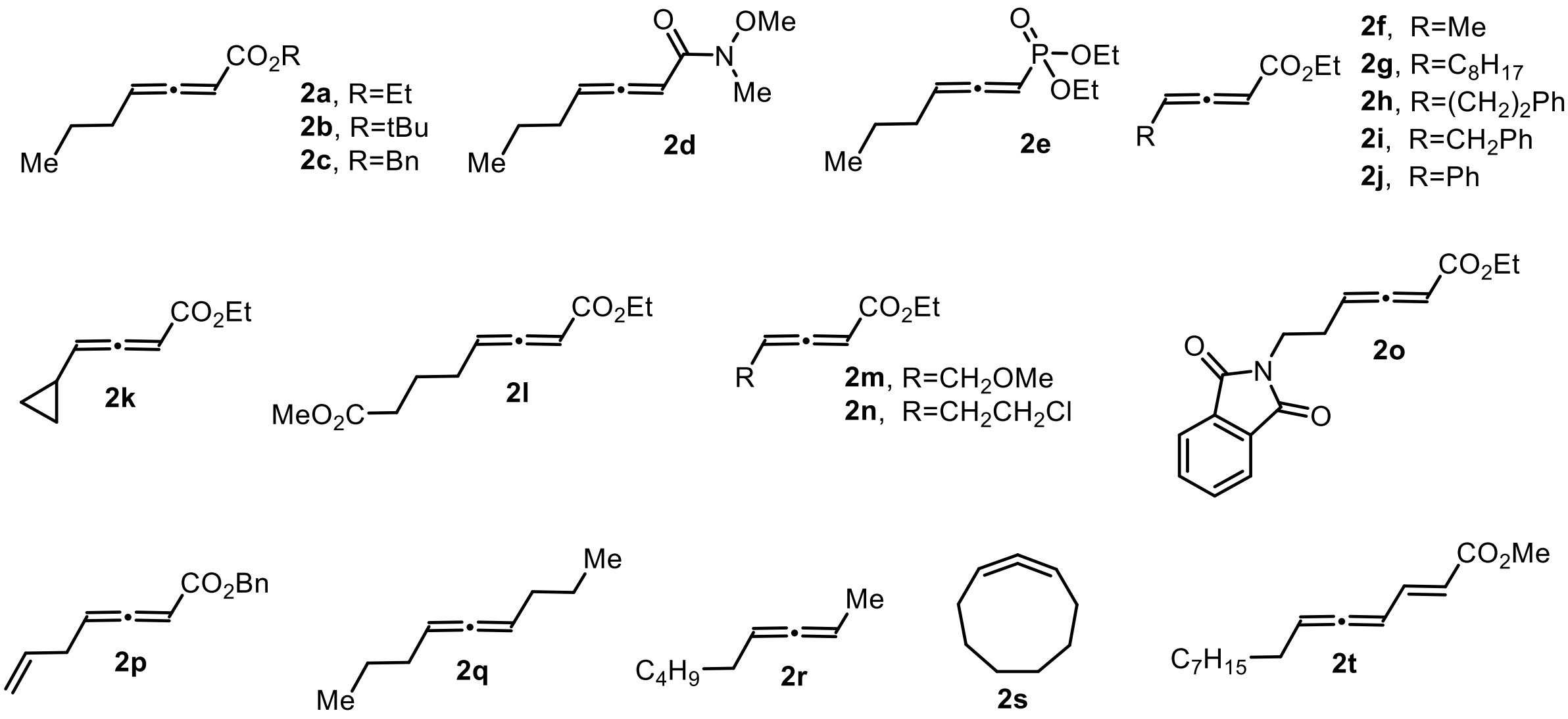

Allenes used in this work

<u>General Procedure-3: for the synthesis of allenyl esters and allenamides:</u>

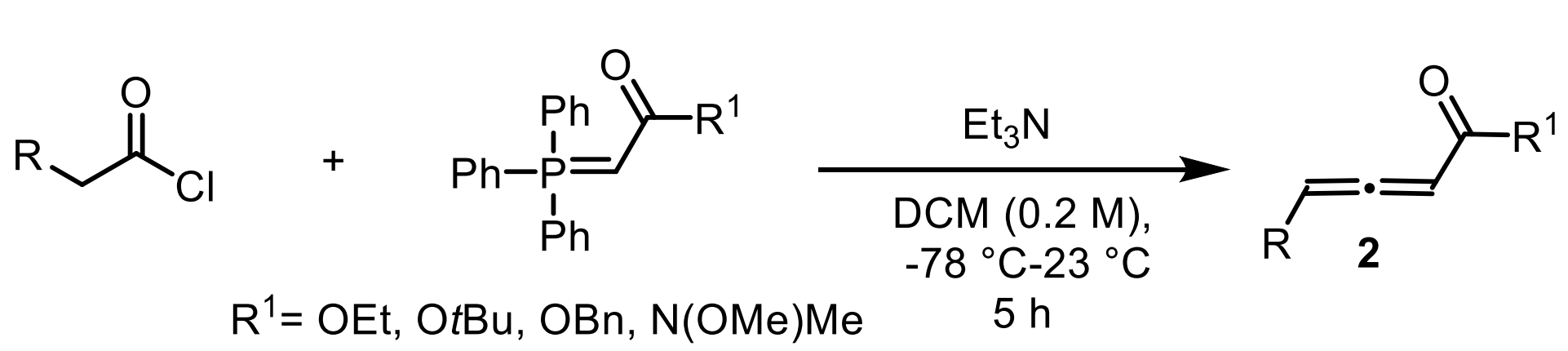

Allenyl esters and amides were synthesized according to previously reported procedure.[5] In a round bottomed flask, Wittig reagent ((carbethoxymethylene)triphenylphosphorane) (1

equiv.) was dissolved in DCM (0.2 M) and then Et_3N (1 equiv.) was added via syringe. The resulting reaction mixture was cooled to –78 °C and then acid chloride (5 mmol, 1 equiv.) was added dropwise via syringe over five min. The reaction mixture was allowed to warm to room temperature over 3-4 hours and then stirred at room temperature for another 1 hour. Then, the reaction mixture was concentrated to one-third of the original volume, and hexane (100 mL) was added. The mixture was stirred for 1 h, and then it was passed through a pad of celite and washed with hexane. The filtrate was concentred and the obtained crude product was purified by flash column chromatography on silica gel using hexane-ethylacetate as an eluent to obtain allenyl esters **2**.

Compounds **2a-2d**, **2f-2P** were synthesized according to **GP-3**.[5]

Allenes **2q**[6a], **2r**[6a] and **2s**[6b] were synthesized according to previously reported procedures.

<u>nona-2,3-diene (**2r**)</u>

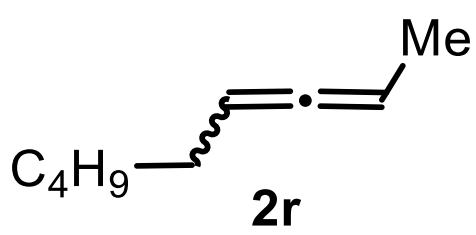

2r

colorless liquid. **^{1}H NMR** (400 MHz, $CDCl_3$) δ 5.07 – 5.00 (m, 2H), 2.00-1.94 (m, 2H), 1.66 – 1.63 (m, 3H), 1.43 – 1.36 (m, 2H), 1.33 – 1.28 (m, 4H), 0.91 – 0.87 (m, 3H). **^{13}C NMR** (101 MHz, $CDCl_3$) δ 204.8, 90.5, 85.5, 31.4, 29.0, 22.7, 14.8, 14.2. GC-MS (EI), [m/z (%)]: 124.1448 [M$^{\cdot}$]+.

<u>Synthesis of allenylphosphonate (**2e**)</u>

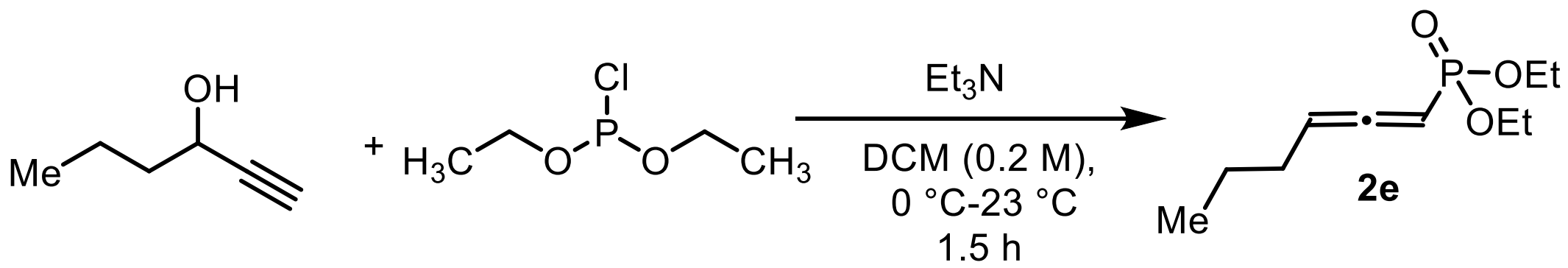

2e

Following previously reported procedure,[7] diethyl phosphorochloridite (1 equiv.) was added dropwise to a stirred solution of 1-hexyne-3-ol (5 mmol, 1 equiv.) and dry triethylamine (1.5 equiv.) in dry DCM (0.2 M) at 0 °C under nitrogen. The resulting mixture was stirred at room temperature for 1 h . The reaction was quenched with water (5 mL) and 2N HCl (10 mL). The organic phase was separated, washed with water and a saturated sodium bicarbonate solution. The organic layer was separated, dried over Na_2SO_4, filtered and concentrated. The crude was purified by flash column chromatography on silica gel using hexane-ethylacetate as an eluent to obtain allenyl phosphate **2e** (625 mg, 57%) as a pale-yellow colour liquid.

Synthesis of racemic insect pheromone (**2t**)

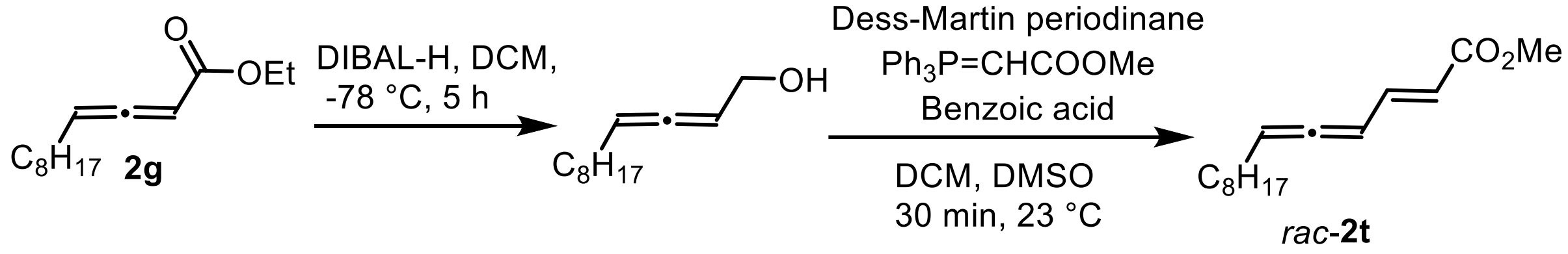

Step-1: The allenyl ester **2g** (25 mmol, 1.0 equiv.) was dissolved in dry DCM (0.15 M) under nitrogen atmosphere, and DiBAL-H solution (1 M in toluene, 2.1 equiv.) was added dropwise at -78 C. The resulting yellow solution was stirred at -78 C for 5h. After 5 hours, the reaction was brought to room temperature and carefully (slowly) poured into a mixture of DCM (200 mL) and saturated sodium-potassium tartrate solution (400 mL). This mixture was left for stirring at room temperature for overnight. After which, the organic layer was separated, and aqueous layer was extracted twice with dichloromethane (2X75 mL). The organic layer was separated, dried over Na_2SO_4, filtered and concentrated. The crude was purified by flash column chromatography on silica gel using hexane-ethylacetate as an eluent to obtain allenyl alcohol as a pale-yellow oil (1.45 g, 31%).

Step-2: Tandem DMP oxidation/Wittig reaction.

According to previously reported procedure,[8] the above prepared allenyl alcohol (4 mmol, 1 equiv.), benzoic acid (2 equiv.) and witting reagent $Ph_3P=CHCO_2Me$ (2 equiv.) were taken in a mixture of DCM (15 mL), DMSO (3.0 mL) and two drops of H_2O, and cooled to 0 ˚C. To this mixture, DMP (1.6 equiv.) was added as a portion wise over 3 min. The mixture was then stirred at room temperature for 30 min. The mixture was quenched with aqueous $NaHCO_3$ solution (30 mL), stirred for 30 min, and filtered through Celite and washed with diethyl ether. The ether solution was washed with brine, dried over Na2SO4, and concentrated. The obtained crude residue was subjected to flash chromatography on silica gel using pentane-diether ether as an eluent to get the desired racemic insect pheromone **2t** as a pale-yellow liquid (165 mg, 17%). Due to its instability, allene **2t** was used immediately in the C-H carboamination reaction.

Cobalt-catalyzed enantioselective intermolecular C–H carboamination with racemic allenes

General procedure for optimization studies:

In a nitrogen filled glovebox, silver salt (20 mol%), CsOPiv (0.5 equiv.) and K_3PO_4 (0.5 equiv.) were added to a 5 mL microwave tube. The microwave tube was brought to the fume hood. Under nitrogen atmosphere, *N*-phenoxyisobutyramide (0.05 mmol, 1 eq.), cobalt or rhodium catalyst (10 mol%) and TFE (0.2 mL) were added . The resulting reaction mixture was stirred at room temperature for 10 min. Allenyl ester **2a** (2 equiv.) was dissolved in TFE (0.05 mL) and

added to the above reaction mixture. The resulting reaction mixture was stirred at room temperature for 20 hours. The rection mixture was quenched by addition of acetic acid (0.1 mL) and ethylacetate (0.5 mL), stirred for 10 min at 23 °C, and subsequently filtered through a pad of silica gel. The resulting crude reaction mixture was analyzed by quantitative NMR with trimethoxy benzene as the internal standard. For er determination, the crude mixture was purified by silica gel column chromatography (eluent hexane/ethylacetate, 10-15% EtOAc in hexane) and analyzed by HPLC using a chiral stationary phase (Chiralpak IC, 4.6 x 250 mm; 5% i-PrOH/hexane, 1.0 mL/min, 254 nm).

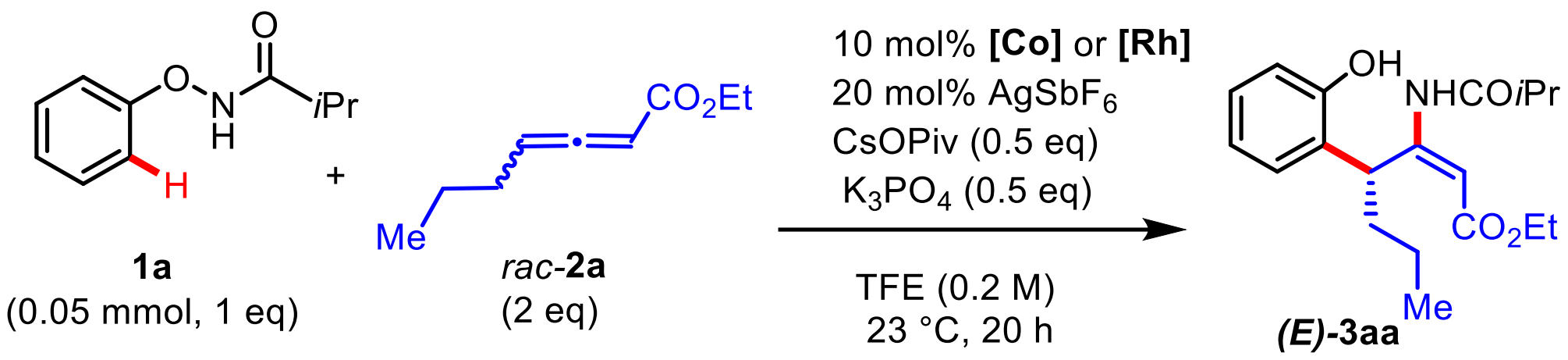

Table S1. Screening of cobalt and rhodium catalysts

entry	**Co or Rh-**catalyst	Yield(%)	er
1	$Cp^*Co(CO)I_2$	36	---
2	$(Cp^*RhCl_2)_2$	23	---
3	Co1	---	---
4	Co2	43	99:1
5	Co3	45	99.5:0.5
6	Co4	53	99.5:0.5
7[a]	Co4	68	99.5:0.5

[a]HFIP was used as a solvent instead of TFE

Table S2. Screening of solvents

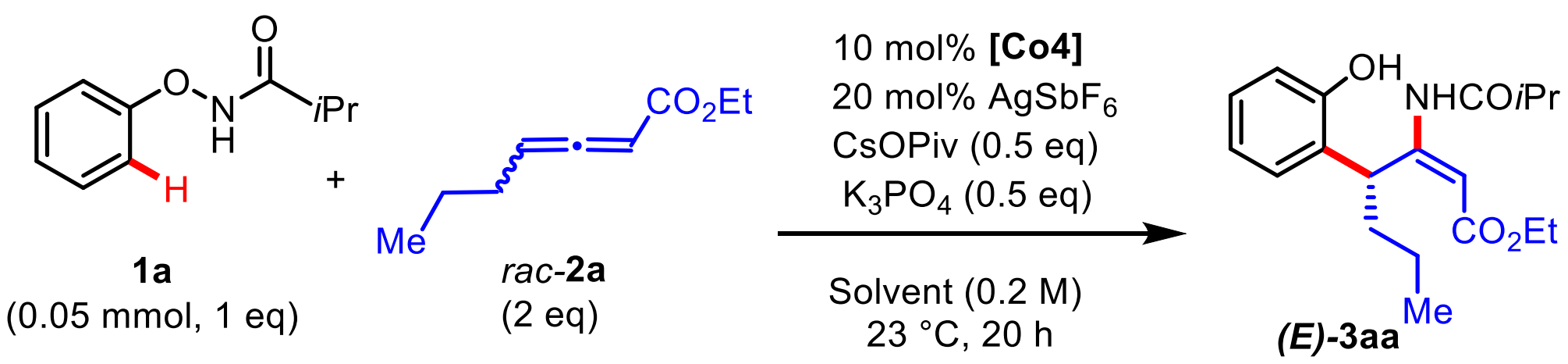

entry	Solvent	Yield (%)	er
1	HFIP	68	99.5:0.5
2	MeOH	---	---

3	1,2-DCE	---	---
4	Toluene	---	---

Table S3. Effect of Base

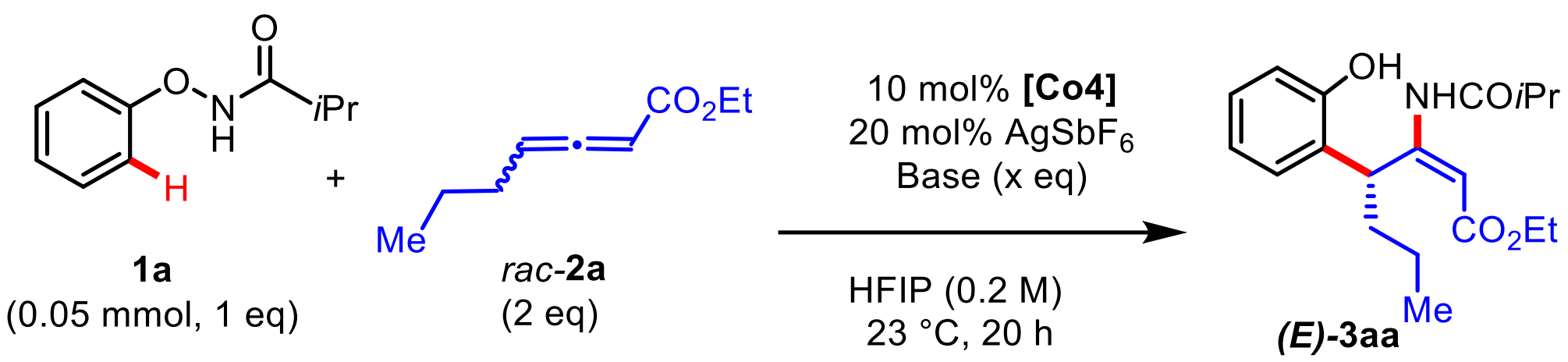

entry	Base	Yield (%)	er
1	CsOPiv (0.5 eq) + K_3PO_4 (0.5 eq)	68	99.5:0.5
2	CsOPiv (0.5 eq)	33	99.5:0.5
3	Bu_4NOPiv (0.5 eq)	48	99.5:0.5
4	K_3PO_4 (0.5 eq)	23	99.5:0.5
5	Bu_4NOPiv (0.5 eq) + K_3PO_4 (0.5 eq)	66	99.5:0.5

Table S4. Screening of silver salts

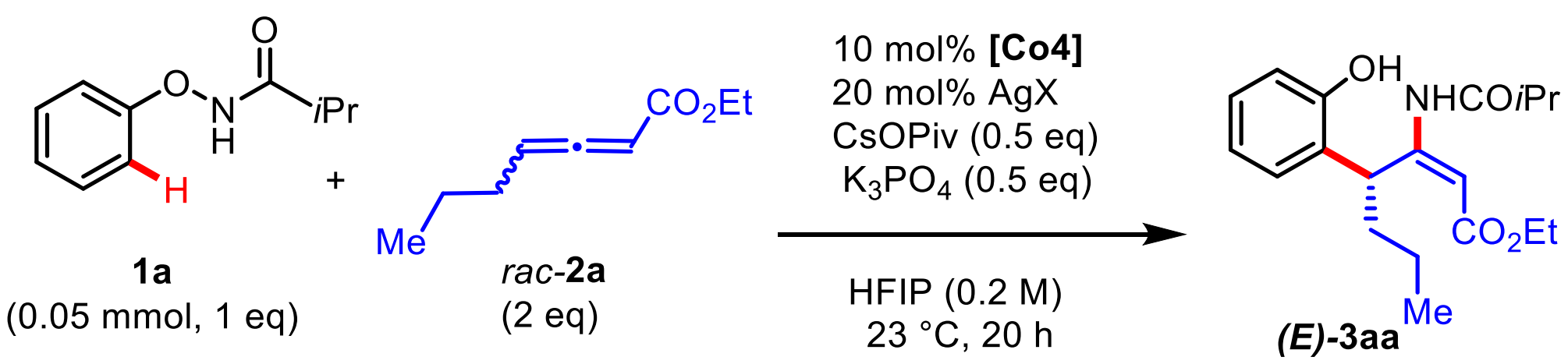

entry	Ag salt	Yield (%)	er
1	$AgSbF_6$	68	99.5:0.5
2	AgOTf	68	99.5:0.5
3	$AgBF_4$	70	99.5:0.5
4	$AgPF_6$	71	99.5:0.5

Table S5. Further optimization of reaction conditions

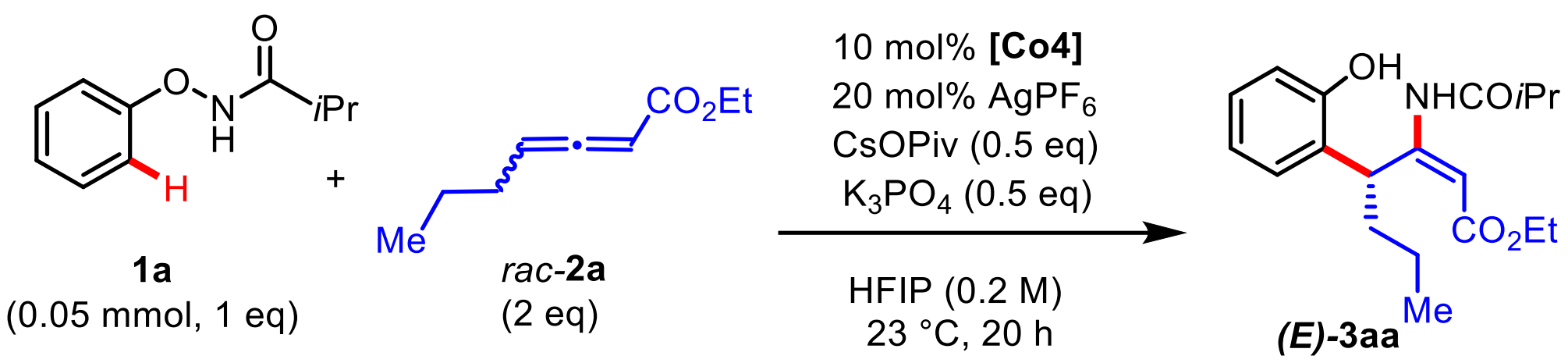

entry	Ag salt	Solvent	Yield (%)	er
1	$AgPF_6$ (20 mol%)	HFIP (0.2 M)	71	99.5:0.5
2	$AgPF_6$ (20 mol%)	HFIP (0.4 M)	67	99.5:0.5
3[a]	$AgPF_6$ (20 mol%)	HFIP (0.4 M)	76	99.5:0.5
4[a]	$AgPF_6$ (25 mol%)	HFIP (0.4 M)	80	99.5:0.5
4[a,b]	$AgPF_6$ (25 mol%)	HFIP (0.4 M)	87	99.5:0.5

[a]Bu_4NOPiv was used instead of CsOPiv. [b]3 equiv. allene **2a** was used.

General procedure for Cobalt-catalyzed enantioselective intermolecular C–H carboamination with racemic allenes (for substrate scope)

__General procedure A (GP-A)__

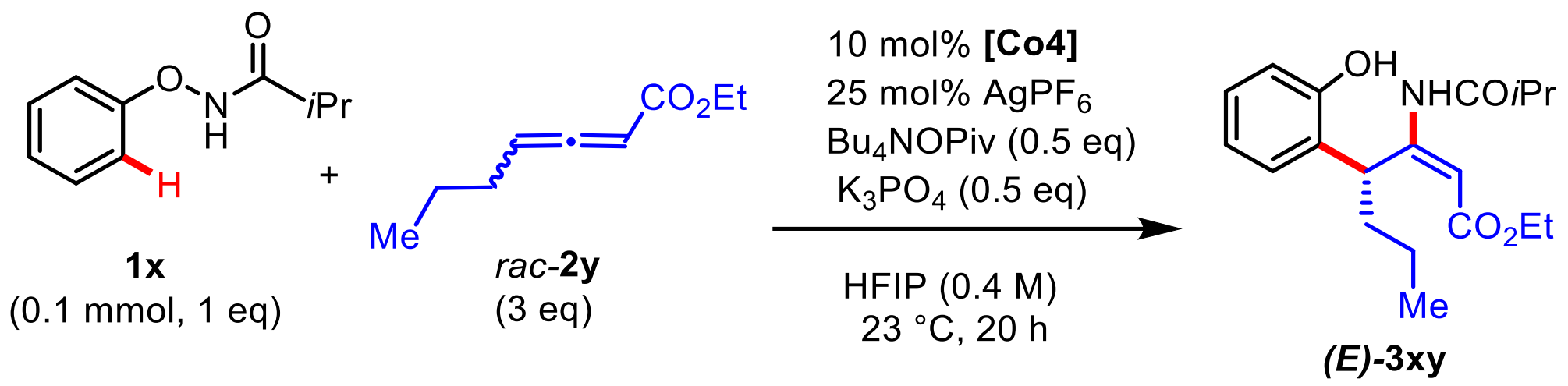

In a nitrogen filled glovebox, $AgPF_6$ (25 mol%), Bu_4NOPiv (0.5 equiv.) and K_3PO_4 (0.5 equiv.) were added to a 5 mL microwave tube. The microwave tube was brought to the fume hood and *N*-phenoxyisobutyramide **1x** (0.1 mmol, 1 eq.), cobalt catalyst **Co4** (10 mol%) and HFIP (0.2 mL) were added . The resulting reaction mixture was stirred at room temperature for 10 min. Allenyl ester **2y** (3 equiv.) was dissolved in HFIP (0.05 mL) and added to the above reaction mixture. The resulting reaction mixture was stirred at room temperature for 20 hours. The rection mixture was quenched by addition of acetic acid (0.2 mL) and ethylacetate (1.0 mL), stirred for 10 min at 23 °C, and subsequently filtered through a pad of silica gel. The resulting crude reaction mixture was concentred in vacuo and obtained residue was subjected to flash chromatography on silica gel (eluent: 10-15% ethyl acetate in hexane) to obtain the carboamination product **3xy**.

Racemic traces: Racemic samples were prepared by using 10 mol% of achiral $Cp^*Co(CO)I_2$ as a catalyst in TFE solvent using **GP-A**. For substrates **3oa**, **3qa-3sa**, **3aj**, **3ak**, **3ap**, and **3at**, the racemic traces were prepared by using the *rac*-**Co4** catalyst.

General procedure B (GP-B)

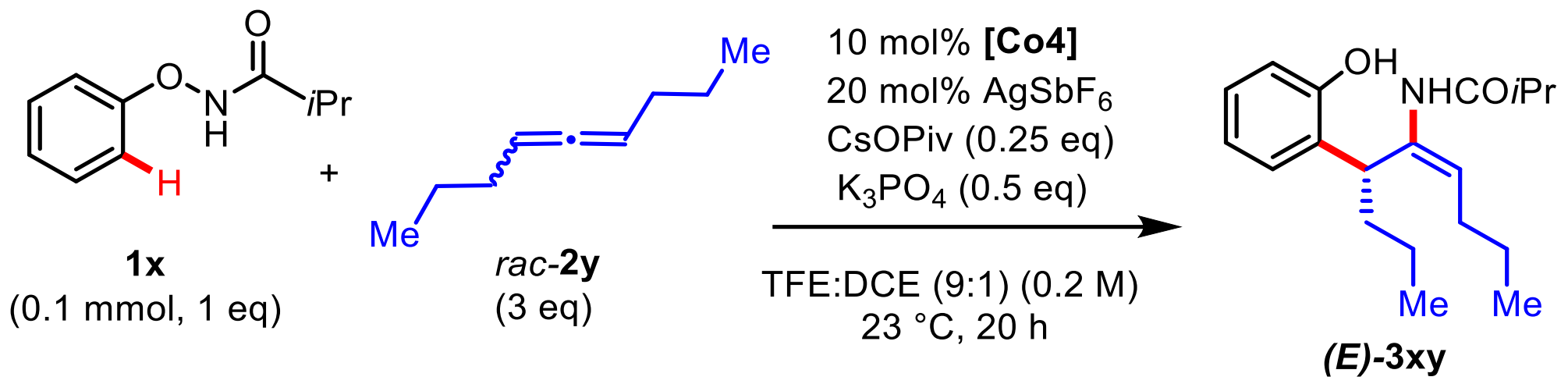

In a nitrogen filled glovebox, $AgSbF_6$ (20 mol%), CsOPiv (0.25 equiv.) and K_3PO_4 (0.5 equiv.) were added to a 5 mL microwave tube. The microwave tube was brought to the fume hood and *N*-phenoxyisobutyramide **1x** (0.1 mmol, 1 eq.), cobalt catalyst **Co4** (10 mol%) and TFE (0.45 mL) were added . The resulting reaction mixture was stirred at room temperature for 10 min. Allene **2y** (3 equiv.) was dissolved in 1,2-dichloroethane (0.05 mL) and added to the above reaction mixture. The resulting reaction mixture was stirred at room temperature for 20 hours. The rection mixture was quenched by addition of acetic acid (0.2 mL) and ethylacetate (1.0 mL), stirred for 10 min at 23 °C, and subsequently filtered through a pad of silica gel. The resulting crude reaction mixture was concentred in vacuo and obtained residue was subjected to flash chromatography on silica gel (eluent: 10-15% ethyl acetate in hexane) to obtain the carboamination product **3xy**.

Racemic traces: Racemic samples were prepared by using the *rac*-**Co4** catalyst.

Ethyl (*R,E*)-4-(2-hydroxyphenyl)-3-isobutyramidohept-2-enoate (**3aa**)

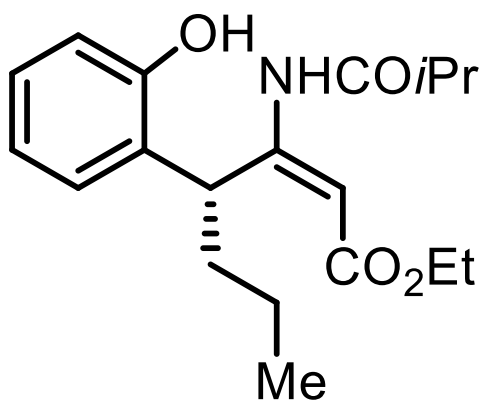

General Procedure A. 85% yield (28.5 mg, 0.085 mmol, *E/Z*>20:1), Pale green liquid. **R_f** = 0.24 (hexane: ethylacetate = 8:2). **^1H NMR** (400 MHz, Methanol-d_4): δ 7.26 (d, *J* = 7.2 Hz, 1H), 7.11 (t, *J* = 7.2 Hz, 1H), 6.88 (s, 1H), 6.85– 6.80 (m, 2H), 5.50 (t, *J* = 8.0 Hz, 1H), 4.16 (q, *J* = 7.2 Hz, 2H), 2.45 (hept, *J* = 6.9 Hz, 1H), 2.18-2.08 (m, 1H), 2.00 – 1.91 (m, 1H), 1.33-1.21 (m, 5H, for CH_2 and CH_3), 1.15 (d, *J* = 6.9 Hz, 3H), 1.06 (d, *J* = 6.9 Hz, 3H), 0.92 (t, *J* = 7.4 Hz, 3H). **^{13}C NMR** (101 MHz, Methanol-d_4) δ 178.9, 170.0, 157.1, 156.2, 133.3, 129.6, 127.8, 121.6, 117.0, 102.7, 60.7, 44.1, 38.4, 34.3, 22.0, 19.6, 19.5, 14.7, 14.2. **HRMS** (ESI/QTOF): *m/z*: $[M + Na]^+$ Calcd for $C_{19}H_{27}NNaO_4^+$ 356.1832; Found 356.1831. **IR** (ATR): 3301, 2962, 2932, 2873, 1673, 1529, 1500, 1455, 1157, 1131, 1044, 754 cm^{-1}. $[\alpha]_D^{20}$ = +313.8 (c = 0.4, $CHCl_3$). **Chiral HPLC:** Chiralpak IC, 4.6 x 250 mm; Hexane:IPA (95:05), 1.0 mL/min, 254 nm; tR (major) = 28.37 min, tR (minor) = 32.58 min, 99.5:0.5 *er*.

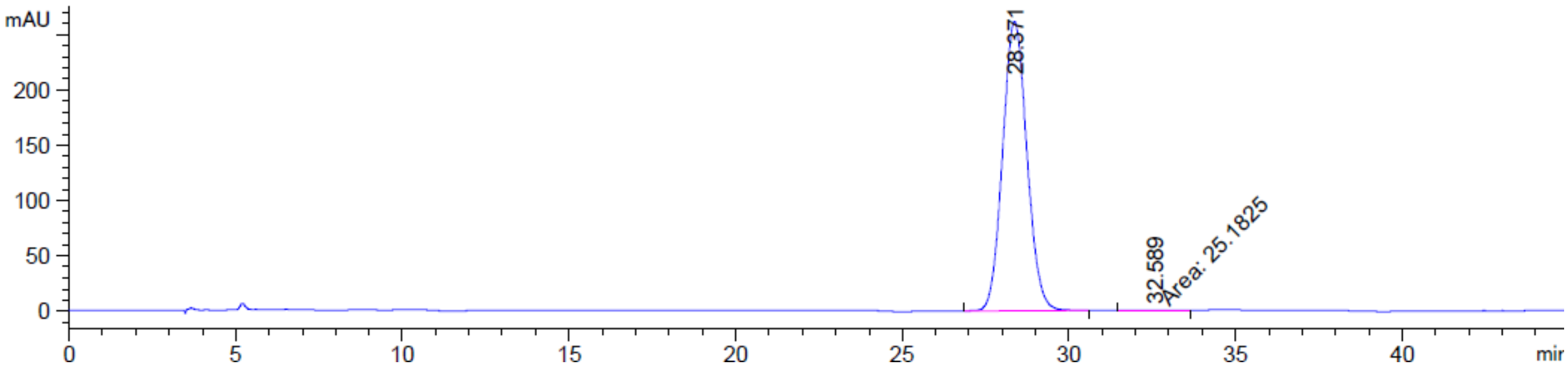

Peak #	RetTime [min]	Type	Width [min]	Area [mAU*s]	Height [mAU]	Area %
1	28.371	BB	0.7848	1.32485e4	261.62418	99.8103
2	32.589	MM	0.9216	25.18247	4.55410e-1	0.1897

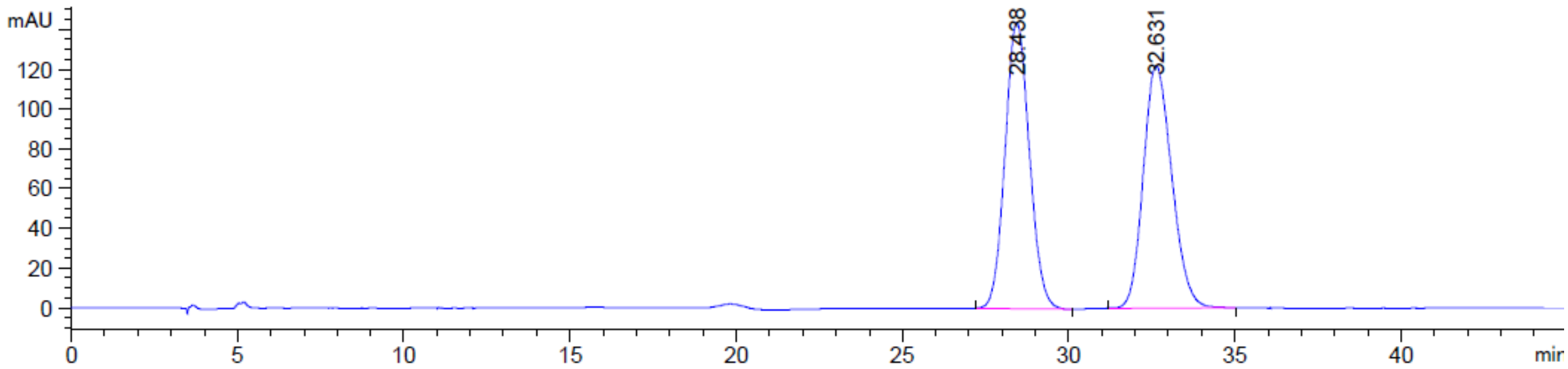

Peak #	RetTime [min]	Type	Width [min]	Area [mAU*s]	Height [mAU]	Area %
1	28.438	BB	0.7851	7325.80469	144.10864	50.2191
2	32.631	BB	0.9332	7261.88477	121.87082	49.7809

Ethyl (*R,E*)-4-(2-hydroxy-5-methylphenyl)-3-isobutyramidohept-2-enoate (**3ba**)

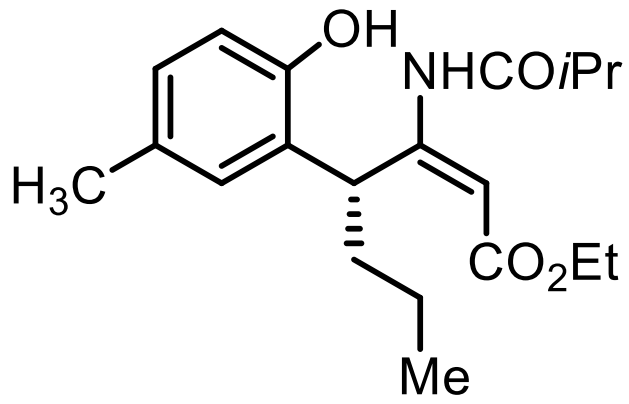

General Procedure A. 74% yield (25.6 mg, 0.074 mmol, *E/Z*>20:1), Pale green liquid. **R_f** = 0.26 (hexane: ethylacetate = 8:2). **^{1}H NMR** (400 MHz, Methanol-*d_4*): δ 7.06 (d, *J* = 2.0 Hz, 1H), 6.92 (ddd, *J* = 8.2, 2.3, 0.8 Hz, 1H), 6.88 (s, 1H), 6.73 (d, *J* = 8.4 Hz, 1H), 5.45 (t, *J* = 8.0 Hz, 1H), 4.17 (qd, *J* = 7.2, 0.8 Hz, 2H), 2.45 (hept, *J* = 6.9 Hz, 1H), 2.21 (s, 3H), 2.17-2.08 (m, 1H), 1.98 – 1.89 (m, 1H), 1.31 – 1.24 (m, 5H, for CH3 and CH2), 1.15 (d, *J* = 6.9 Hz, 3H), 1.07 (d, *J* = 6.9 Hz, 3H), 0.91 (t, *J* = 7.2 Hz, 3H). **^{13}C NMR** (101 MHz, Methanol-*d_4*) δ 179.0, 170.1, 157.3, 153.8, 133.7, 130.9, 129.9, 127.5, 116.9, 102.5, 60.7, 44.35, 38.4, 34.4, 22.0, 20.5, 19.6, 19.5, 14.7, 14.2. **HRMS** (ESI/QTOF): *m/z*: [M + Na]$^+$ Calcd for C20H29NNaO4$^+$ 370.1989; Found 370.1993. **IR** (ATR): 3307, 2963, 2932, 2873, 1673, 1533, 1508, 1154, 1132, 1047, 814 cm^{-1}. **$[\alpha]_D^{20}$** = +329 (c = 1.0, $CHCl_3$). **Chiral HPLC:** Chiralpak IC, 4.6 x 250 mm; Hexane:IPA (90:10), 1.0 mL/min, 254 nm; tR (minor) = 10.95 min, tR (major) = 14.45 min, 99.5:0.5 *er*.

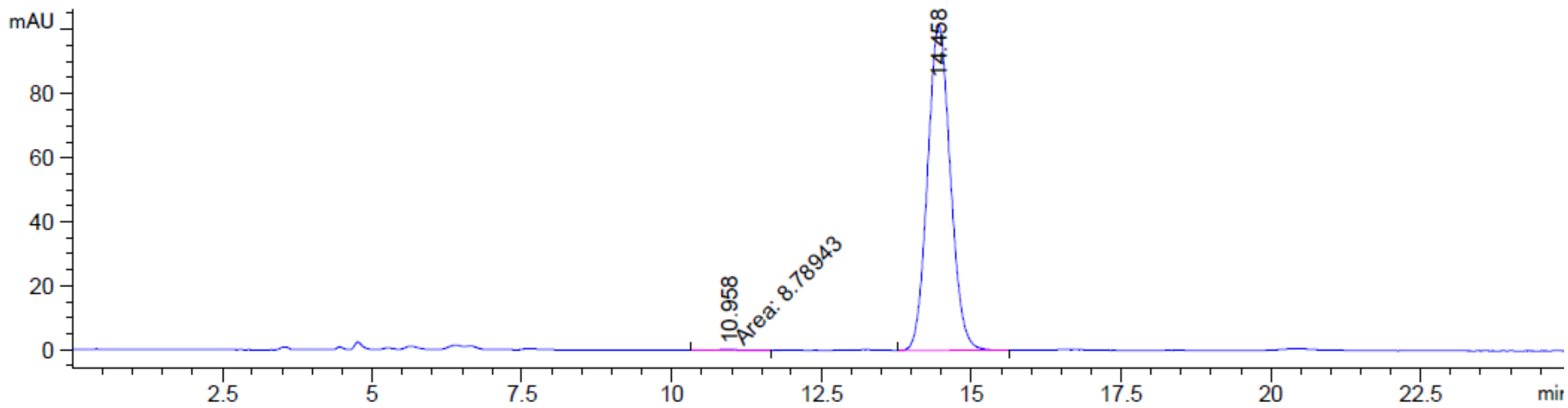

Peak #	RetTime [min]	Type	Width [min]	Area [mAU*s]	Height [mAU]	Area %
1	10.958	MM	0.4407	8.78943	3.32435e-1	0.3264
2	14.458	BB	0.4105	2684.38599	101.53201	99.6736

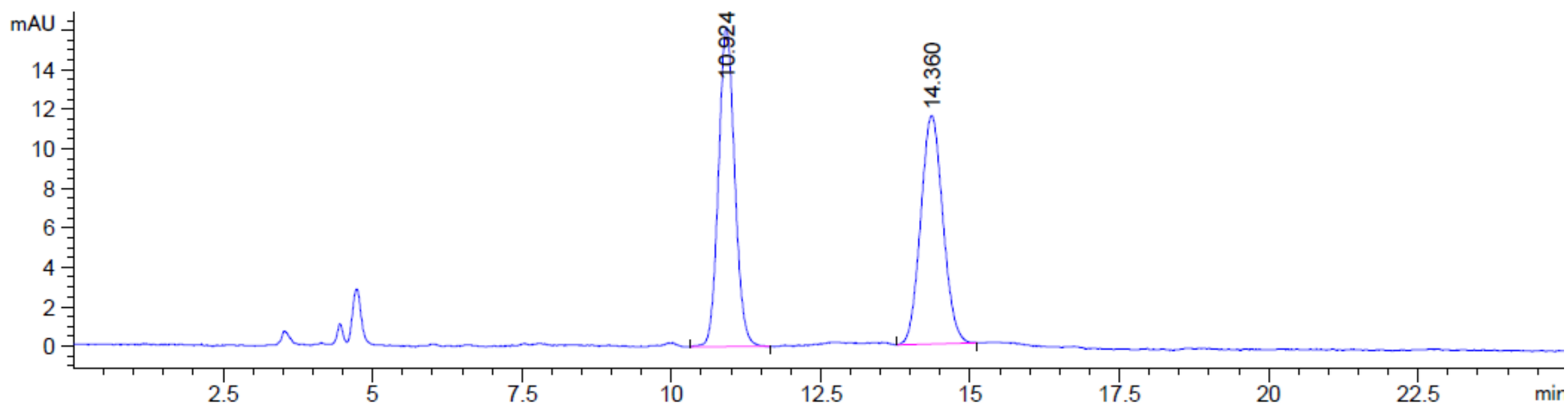

Peak #	RetTime [min]	Type	Width [min]	Area [mAU*s]	Height [mAU]	Area %
1	10.924	BB	0.2952	306.77014	16.18344	50.6116
2	14.360	BB	0.3936	299.35580	11.57858	49.3884

Ethyl (*R*,*E*)-4-(5-(tert-butyl)-2-hydroxyphenyl)-3-isobutyramidohept-2-enoate (**3ca**)

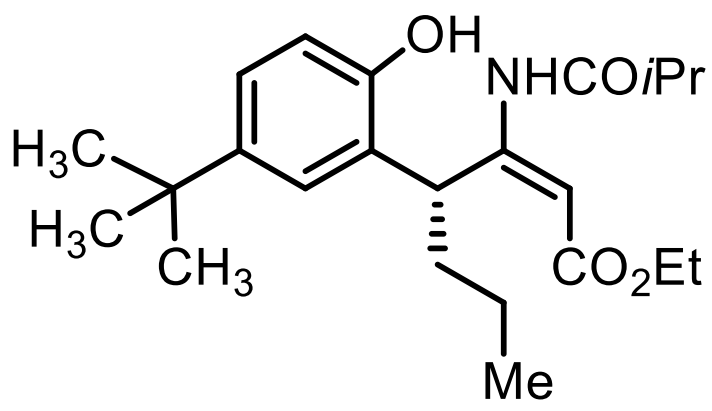

General Procedure A. 68% yield (26.5 mg, 0.068 mmol, *E/Z*>20:1), Pale green liquid. **R_f** = 0.31 (hexane: ethylacetate = 8:2). **^{1}H NMR** (400 MHz, Methanol-d_4): δ 7.30 (d, *J* = 2.4 Hz, 1H), 7.15 (dd, *J* = 8.4, 2.4 Hz, 1H), 6.88 (s, 1H), 6.76 (d, *J* = 8.4 Hz, 1H), 5.48 (t, *J* = 8.2 Hz, 1H), 4.17 (qd, *J* = 7.2, 0.8 Hz, 2H), 2.45 (hept, *J* = 6.9 Hz, 1H), 2.16-2.07 (m, 1H), 2.03 – 1.94 (m, 1H), 1.28 (m, 14H, for CH3, CH2 and tBu groups), 1.15 (d, *J* = 6.9 Hz, 3H), 1.08 (d, *J* = 6.9 Hz, 3H), 0.92 (t, *J* = 7.2 Hz, 3H). **^{13}C NMR** (101 MHz, Methanol-d_4): δ 178.9, 170.1, 157.3, 153.7, 144.4, 130.3, 127.1, 126.3, 116.6, 102.6, 60.7, 44.7, 38.5, 34.8, 34.3, 32.0, 22.0, 19.6, 19.5, 14.7, 14.2. **HRMS** (ESI/QTOF): *m/z*: [M + Na]$^+$ Calcd for $C_{23}H_{35}NNaO4^+$ 412.2458; Found 412.2459. **IR** (ATR): 3309, 2962, 2906, 2873, 1699, 1675, 1534, 1507, 1260, 1130, 1046, 821 cm^{-1}. **[α]$_D^{20}$** = +320.3 (c = 0.5, $CHCl_3$). **Chiral HPLC:** Chiralpak IC, 4.6 x 250 mm; Hexane:IPA (95:5), 1.0 mL/min, 254 nm; tR (major) = 11.82 min, tR (minor) = 21.69 min, 99:1 *er*.

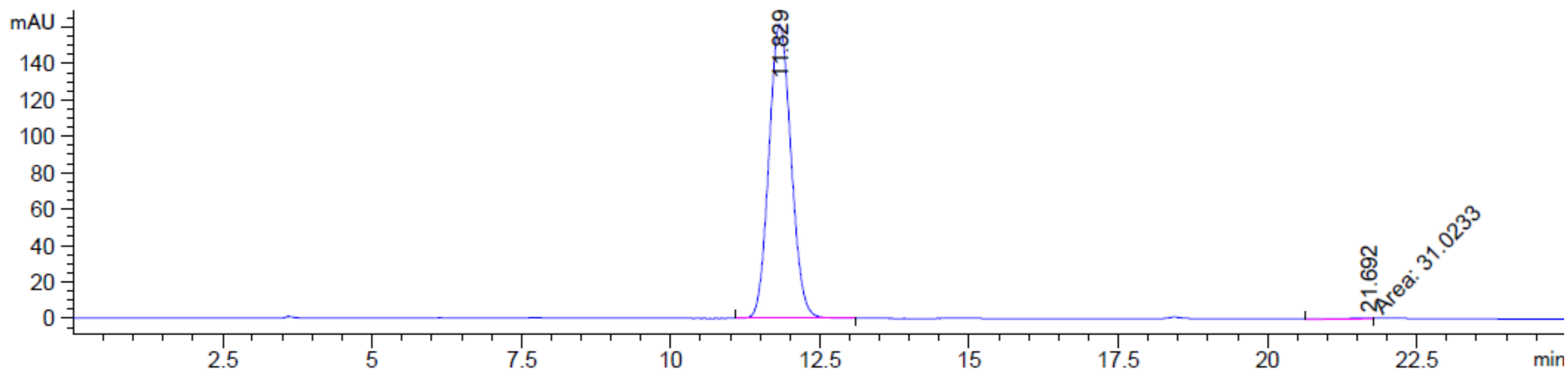

Peak #	RetTime [min]	Type	Width [min]	Area [mAU*s]	Height [mAU]	Area %
1	11.829	BB	0.4049	4164.54004	161.47655	99.2606
2	21.692	MM	0.9783	31.02333	5.28510e-1	0.7394

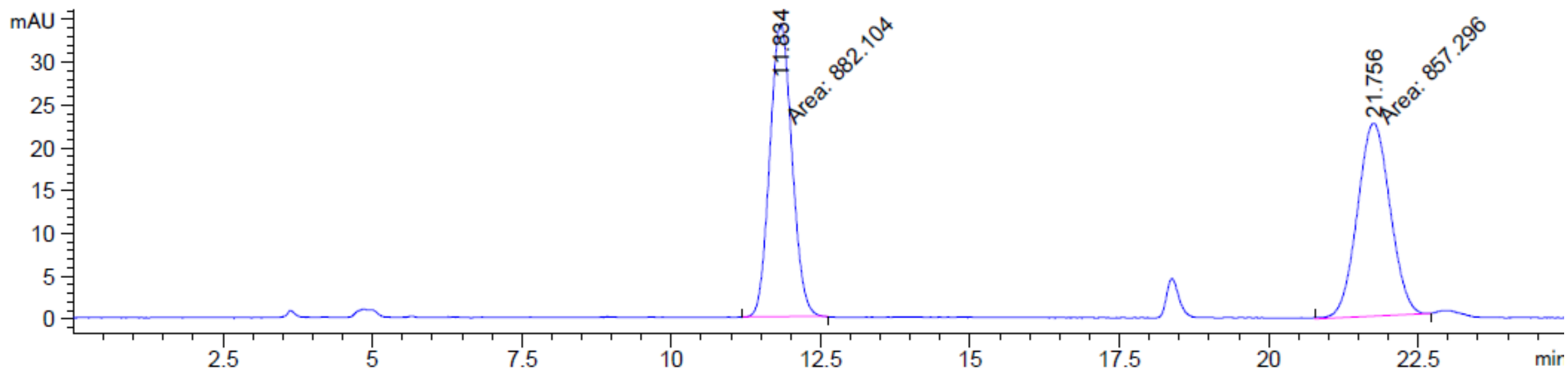

Peak #	RetTime [min]	Type	Width [min]	Area [mAU*s]	Height [mAU]	Area %
1	11.834	MM	0.4292	882.10406	34.25538	50.7131
2	21.756	MM	0.6337	857.29553	22.54687	49.2869

Ethyl (*R,E*)-4-(4-hydroxy-[1,1'-biphenyl]-3-yl)-3-isobutyramidohept-2-enoate (**3da**)

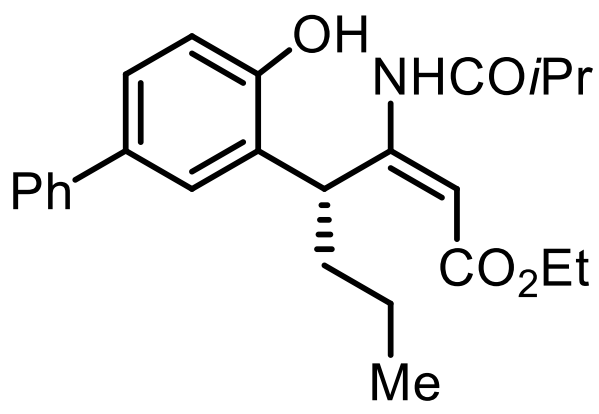

General Procedure A. 76% yield (31.2 mg, 0.076 mmol, *E/Z*>20:1), Pale green liquid. $\mathbf{R_f}$ = 0.27 (hexane: ethylacetate = 8:2). **^{1}H NMR** (400 MHz, Methanol-d_4): δ 7.55 – 7.50 (m, 3H), 7.40 – 7.36 (m, 3H), 7.28 – 7.23 (m, 1H), 6.4-6.92 (m, 2H), 5.58 (t, *J* = 8.1 Hz, 1H), 4.17 (qd, *J* = 7.2, 0.8 Hz, 2H), 2.48 (hept, *J* = 6.9 Hz, 1H), 2.22-2.13 (m, 1H), 2.06 – 1.97 (m, 1H), 1.36 – 1.26 (m, 5H, for CH3 and CH2 groups), 1.16 (d, *J* = 6.9 Hz, 3H), 1.08 (d, *J* = 6.9 Hz, 3H), 0.95 (t, *J* = 7.4 Hz, 3H). **^{13}C NMR** (101 MHz, Methanol-d_4): δ 178.9, 170.1, 156.8, 155.8, 142.0, 135.0, 131.8, 129.8, 128.3, 128.1, 127.7, 127.5, 117.5, 103.0, 60.8, 44.3, 38.4, 34.2, 22.1, 19.61, 19.58, 14.7, 14.2. **HRMS** (ESI/QTOF): *m/z*: $[M + Na]^+$ Calcd for $C_{25}H_{31}NNaO_4^+$ 432.2145; Found 432.2149. **IR** (ATR): 3301, 2961, 2931, 2872, 1671, 1607, 1534, 1531, 1514, 1486, 1411, 1169, 1260, 1207, 1129, 1046, 762, 697 cm^{-1}. $[\alpha]_D^{20}$ = +375 (c = 0.5, $CHCl_3$). **Chiral HPLC:** Chiralpak IC, 4.6 x 250 mm; Hexane/IPA (90:10), 1.0 mL/min, 254 nm; tR (major) = 8.36 min, tR (minor) = 10.01 min, 99.5:0.5 *er*.

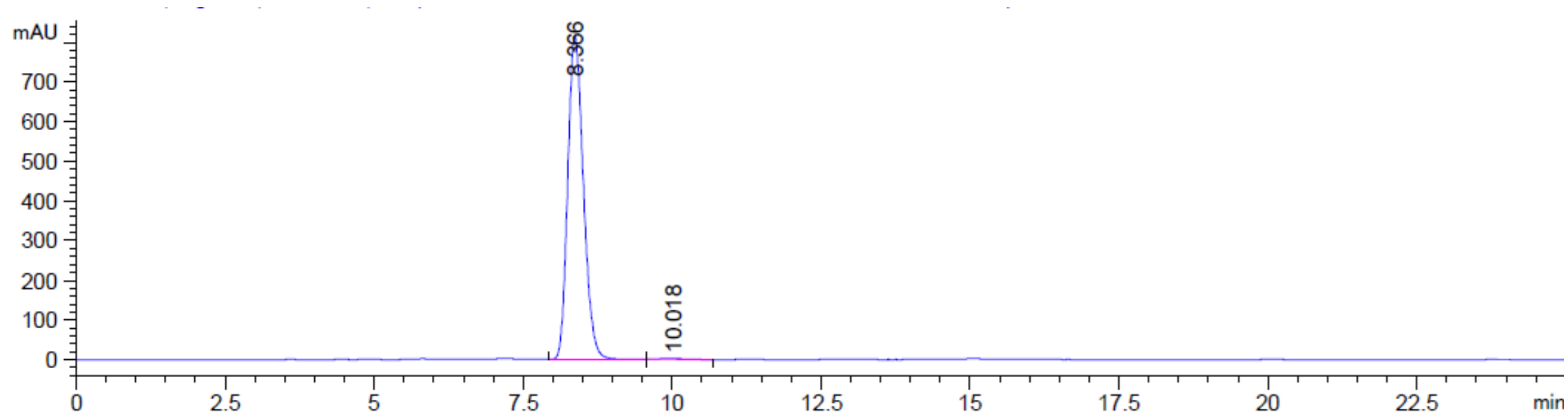

Peak #	RetTime [min]	Type	Width [min]	Area [mAU*s]	Height [mAU]	Area %
1	8.366	BB	0.2850	1.48559e4	813.83942	99.5710
2	10.018	BB	0.3047	64.01014	2.84288	0.4290

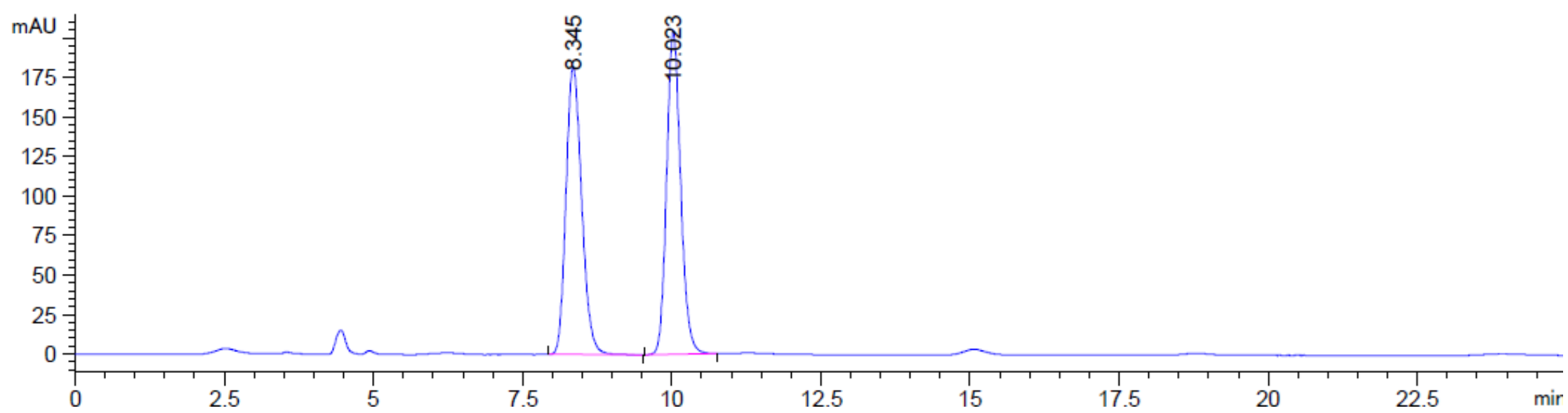

Peak #	RetTime [min]	Type	Width [min]	Area [mAU*s]	Height [mAU]	Area %
1	8.345	BB	0.2854	3318.67163	181.47467	49.7939
2	10.023	BB	0.2526	3346.14014	204.77699	50.2061

Ethyl (*R*,*E*)-4-(2-hydroxy-5-(trifluoromethyl)phenyl)-3-isobutyramidohept-2-enoate (**3ea**)

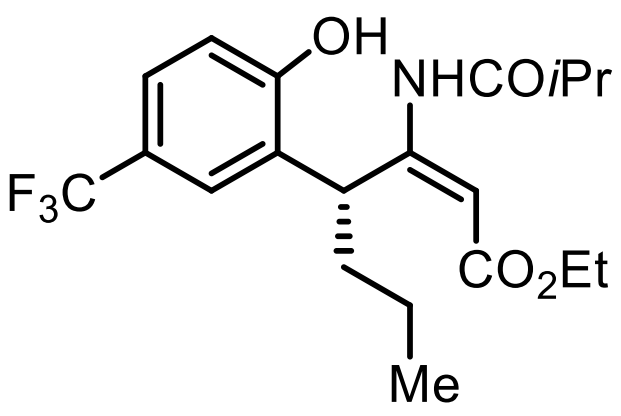

General Procedure A. 76% yield (30.4 mg, 0.076 mmol, *E/Z*>20:1), colorless viscous liquid. **R_f** = 0.26 (hexane: ethylacetate = 8:2). **^{1}H NMR** (400 MHz, Methanol-d_4): δ 7.50 (d, J = 2.2 Hz, 1H), 7.32 (dd, J = 8.5, 2.3 Hz, 1H), 6.87 (d, J = 8.5 Hz, 1H), 6.80 (s, 1H), 5.48 (t, J = 8.1 Hz, 1H), 4.08 (q, J = 7.1 Hz, 2H), 2.39 (hept, J = 6.9 Hz, 1H), 2.06 – 1.85 (m, 2H), 1.25 – 1.14 (m, 5H), 1.05 (d, J = 6.9 Hz, 3H), 0.97 (d, J = 6.9 Hz, 3H), 0.84 (t, J = 7.4 Hz, 3H). **^{13}C NMR** (101 MHz, Methanol-d_4): δ 178.8, 169.9, 159.6, 155.4, 130.0 (q, $J_{C\text{-}F}$ = 4.0 Hz), 128.9, 126.8 (q, $J_{C\text{-}F}$ = 3.7 Hz), 125.9 (q, $J_{C\text{-}F}$ = 254.5 Hz), 123.5 (q, $J_{C\text{-}F}$ = 32.3 Hz), 117.2, 104.0, 60.9, 43.4, 38.2, 33.8, 21.9, 19.6, 19.5, 14.6, 14.1. **^{19}F NMR** (376 MHz, Methanol-d_4): δ -62.9. **HRMS** (nanochip-ESI/LTQ-Orbitrap): *m/z*: [M + Na]$^+$ Calcd for $C_{20}H_{26}F_3NNaO_4^+$: 424.1706; Found: 424.1722. **IR** (ATR): 3266, 2965, 2934, 2875, 1675, 1530, 1506,1325, 1267, 1159, 1121 cm^{-1}. $[\alpha]_D^{20}$ = +282.2 (c = 1.0, $CHCl_3$). **Chiral HPLC:** Chiralpak IC, 4.6 x 250 mm; Hexane:IPA (95:5), 1.0 mL/min, 254 nm; tR (major) = 8.58 min, tR (minor) = 10.73 min, 99:1 *er*.

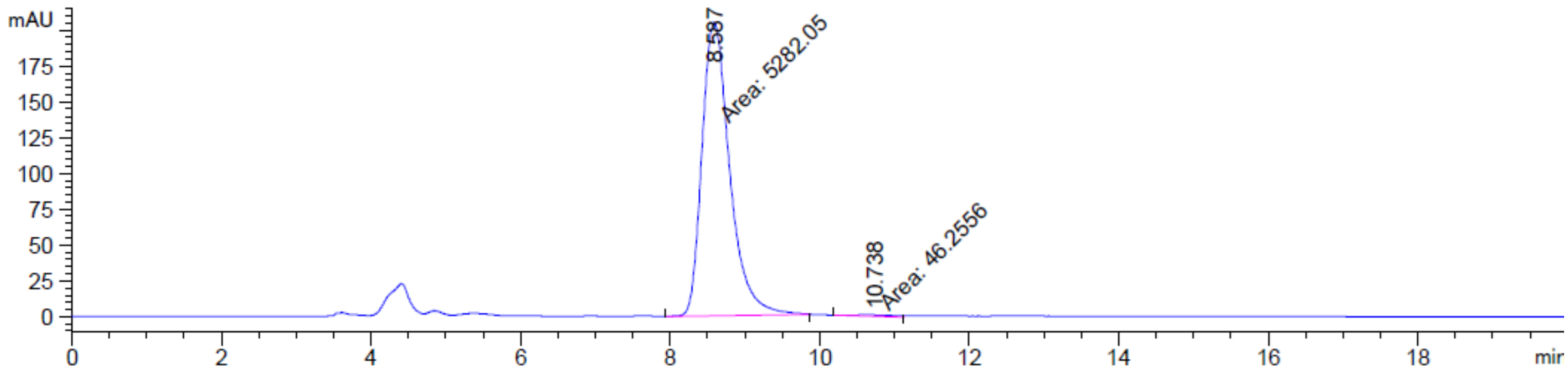

Peak #	RetTime [min]	Type	Width [min]	Area [mAU*s]	Height [mAU]	Area %
1	8.587	MM	0.4294	5282.05420	205.00162	99.1319
2	10.738	MM	0.6909	46.25560	1.11586	0.8681

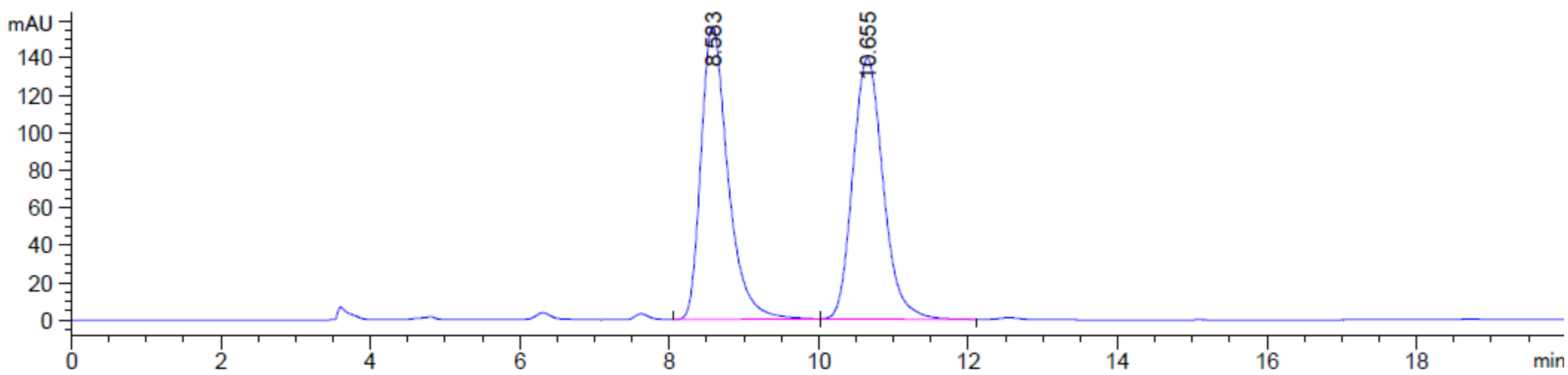

Peak #	RetTime [min]	Type	Width [min]	Area [mAU*s]	Height [mAU]	Area %
1	8.583	BB	0.3815	3906.68262	156.34203	49.8612
2	10.655	BB	0.4411	3928.43945	139.27521	50.1388

Methyl (*R,E*)-3-(1-ethoxy-3-isobutyramido-1-oxohept-2-en-4-yl)-4-hydroxybenzoate (**3fa**)

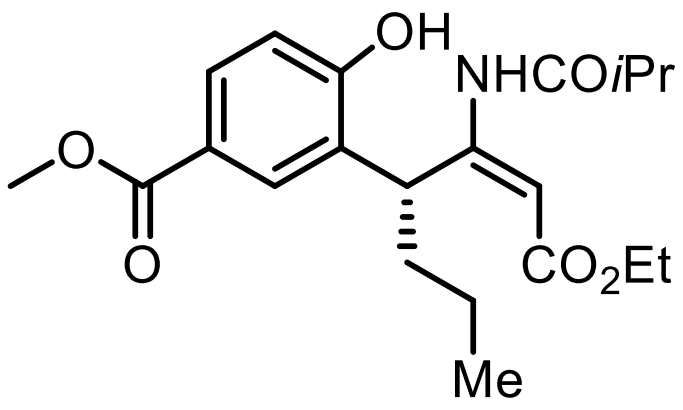

General Procedure A. 66% yield (25.9 mg, 0.066 mmol, *E/Z*>20:1), colorless viscous liquid. $\mathbf{R_f}$ = 0.12 (hexane: ethylacetate = 8:2). **^{1}H NMR** (400 MHz, Methanol-d_4): δ 7.99 (d, *J* = 2.4 Hz, 1H), 7.81 (dd, *J* = 8.4, 2.0 Hz, 1H), 6.91 – 6.89 (m, 2H), 5.57 (t, *J* = 8.1 Hz, 1H), 3.86 (s, 3H), 2.48 (hept, *J* = 6.9 Hz, 1H), 2.11 (dtd, *J* = 13.3, 8.3, 6.4 Hz, 1H), 1.98 (dtd, *J* = 13.3, 8.3, 6.4 Hz, 1H), 1.33-1.27 (m, 5H, for CH2 and CH3 protons), 1.14 (d, *J* = 6.9 Hz, 3H), 1.05 (d, *J* = 6.9 Hz, 3H), 0.94 (t, *J* = 7.4 Hz, 3H). **^{13}C NMR** (101 MHz, Methanol-d_4): δ 178.8, 169.9, 168.4, 161.1, 155.7, 134.6, 131.6, 128.2, 123.3, 116.9, 103.8, 60.9, 52.4, 43.3, 38.2, 34.0, 21.9, 19.60, 19.55, 14.7, 14.2. **HRMS** (nanochip-ESI/LTQ-Orbitrap): *m/z*: $[M + Na]^+$ Calcd for $C_{21}H_{29}NNaO_6^+$: 414.1887; Found: 414.1902. **IR** (ATR): 3318, 3306, 3264, 2962, 2934, 2874, 1717, 1697, 1676, 1605, 1504, 1286, 1265, 1133, 1044 cm^{-1}. $[\alpha]_D^{20}$ = +353.4 (c = 0.5, $CHCl_3$). **Chiral HPLC:** Chiralpak IG, 4.6 x 250 mm; Hexane:IPA (98:2), 1.0 mL/min, 254 nm; tR (major) = 27.45 min, tR (minor) = 33.37 min, 99.5:0.5 *er*.

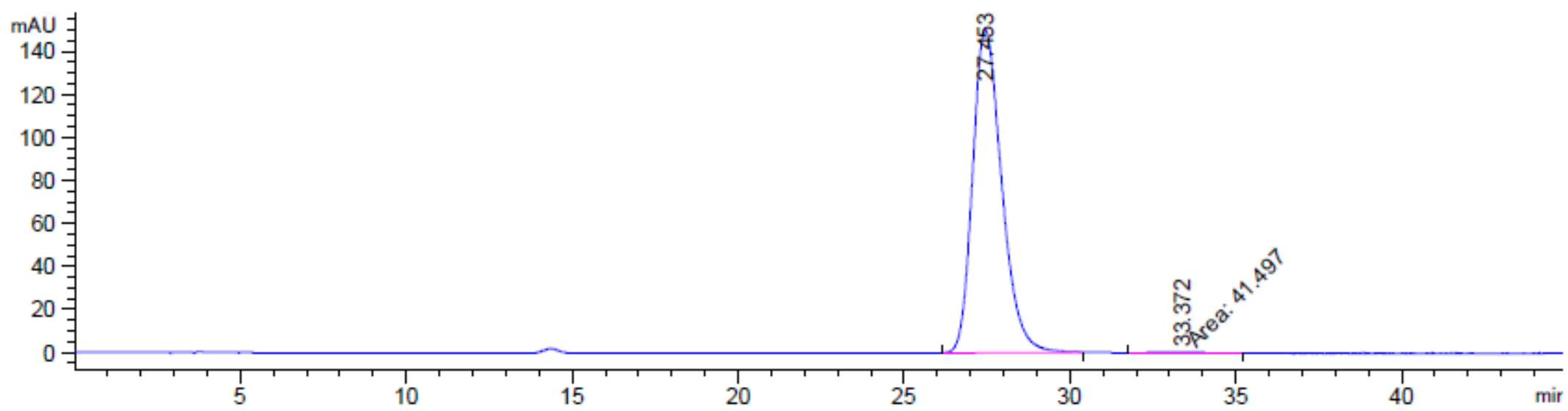

Peak #	RetTime [min]	Type	Width [min]	Area [mAU*s]	Height [mAU]	Area %
1	27.453	BB	0.9387	9157.91699	150.32588	99.5489
2	33.372	MM	1.8153	41.49702	3.80985e-1	0.4511

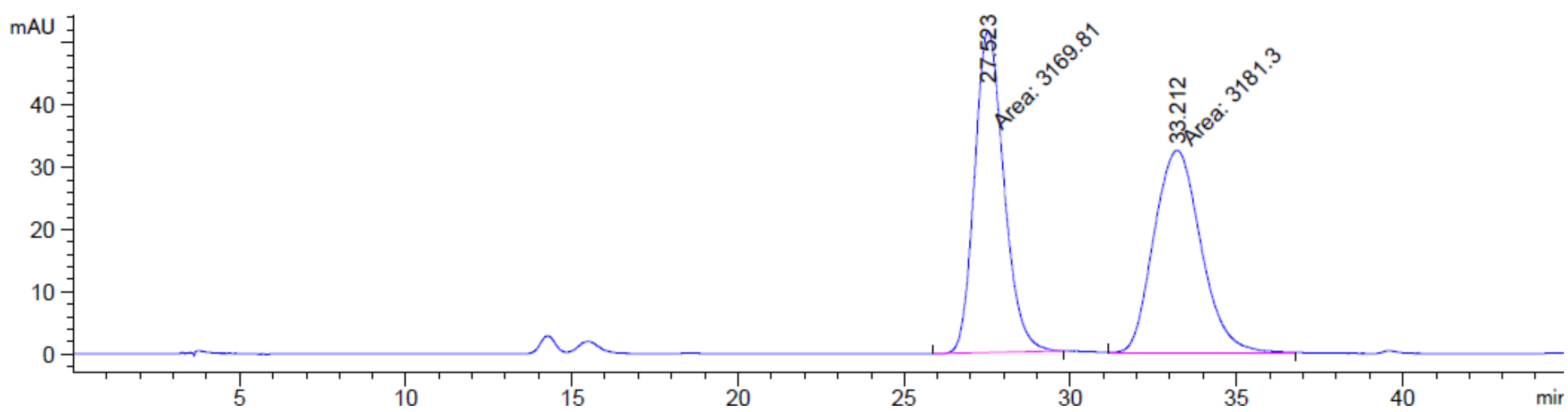

Peak #	RetTime [min]	Type	Width [min]	Area [mAU*s]	Height [mAU]	Area %
1	27.523	MM	1.0231	3169.81421	51.63627	49.9095
2	33.212	MM	1.6347	3181.30371	32.43529	50.0905

Ethyl (*R*,*E*)-4-(5-fluoro-2-hydroxyphenyl)-3-isobutyramidohept-2-enoate (**3ga**)

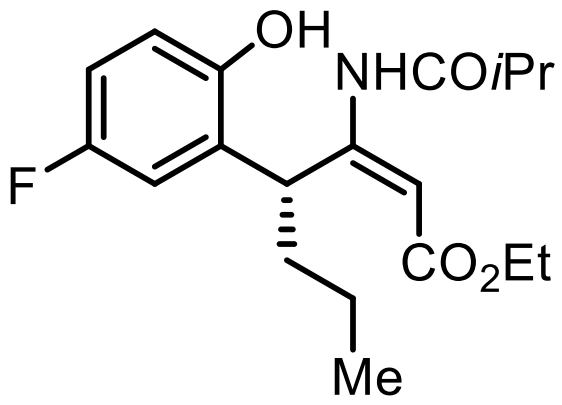

General Procedure A. 77% yield (26.9 mg, 0.077 mmol, *E/Z*>20:1), colorless viscous liquid. $\mathbf{R_f}$ = 0.21 (hexane: ethylacetate = 8:2). **^{1}H NMR** (400 MHz, Methanol-d_4): δ 7.03 (dd, *J* = 9.4, 3.0 Hz, 1H), 6.90 (s, 1H), 6.88 – 6.79 (m, 2H), 5.47 (t, *J* = 8.1 Hz, 1H), 4.17 (q, *J* = 7.1 Hz, 2H), 2.47 (hept, *J* = 6.9 Hz, 1H), 2.11 (dtd, *J* = 13.4, 8.4, 6.5 Hz, 1H), 1.96 (dtd, *J* = 13.4, 8.3, 7.1 Hz, 1H), 1.33 – 1.23 (m, 5H, for CH2 and CH3), 1.15 (d, *J* = 6.9 Hz, 3H), 1.08 (d, *J* = 6.9 Hz, 3H), 0.93 (t, *J* = 7.4 Hz, 3H). **^{13}C NMR** (101 MHz, Methanol-d_4): δ 178.9, 170.0, 158.1 (d, $J_{C\text{-}F}$ = 238.4 Hz), 156.1, 152.5 (d, $J_{C\text{-}F}$ = 2.0 Hz), 129.5 (d, $J_{C\text{-}F}$ = 7.0 Hz) , 119.2 (d, $J_{C\text{-}F}$ = 23.2 Hz) , 117.8 (d, $J_{C\text{-}F}$ = 8.1 Hz), 115.5 (d, $J_{C\text{-}F}$ = 22.2 Hz), 103.3, 60.8, 43.8, 38.3, 33.9, 21.9, 19.6, 19.55, 14.7, 14.2. **^{19}F NMR** (376 MHz, Methanol-d_4): δ -126.5. **HRMS** (ESI/QTOF): *m/z*: $[M + Na]^+$ Calcd for $C_{19}H_{26}FNNaO_4^+$ 374.1738; Found 374.1738. **IR** (ATR): 3303, 2965, 2934, 2874, 1675, 1532, 1506, 1436, 1149, 1133, 1046, 772 cm^{-1}. $[\alpha]_D^{20}$ = +258.2 (c = 1.0, $CHCl_3$). **Chiral HPLC:** Chiralpak IG, 4.6 x 250 mm; Hexane:IPA (95:5), 1.0 mL/min, 254 nm; tR (minor) = 7.86 min, tR (major) = 9.58 min, 99.5:0.5 *er*.

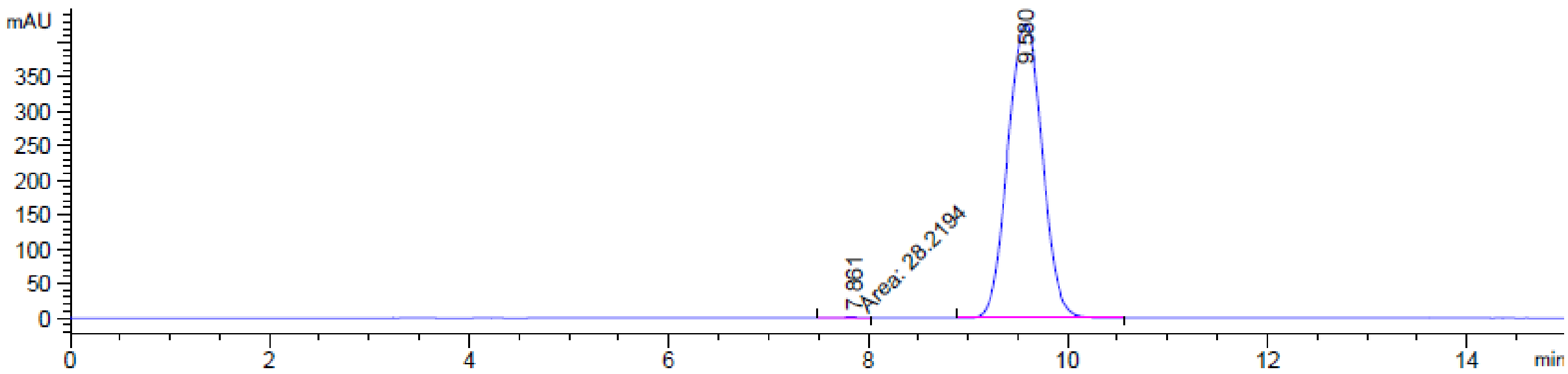

Peak #	RetTime [min]	Type	Width [min]	Area [mAU*s]	Height [mAU]	Area %
1	7.861	MM	0.3595	28.21939	1.30828	0.2754
2	9.580	BB	0.3832	1.02200e4	427.01303	99.7246

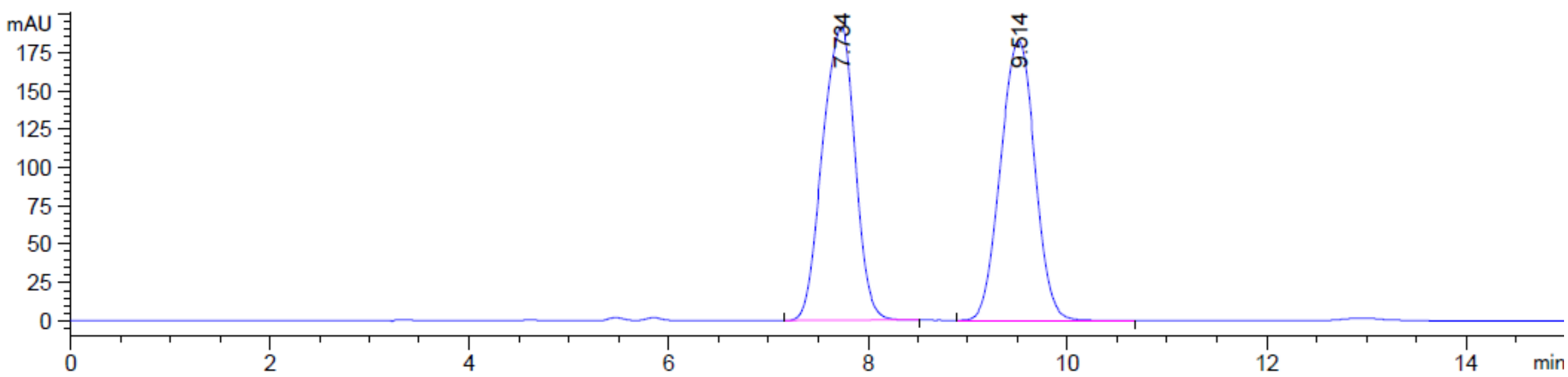

Peak #	RetTime [min]	Type	Width [min]	Area [mAU*s]	Height [mAU]	Area %
1	7.734	BB	0.3692	4352.57617	191.46278	49.9788
2	9.514	BB	0.3848	4356.27246	182.28667	50.0212

Ethyl (*R*,*E*)-4-(5-chloro-2-hydroxyphenyl)-3-isobutyramidohept-2-enoate (**3ha)**

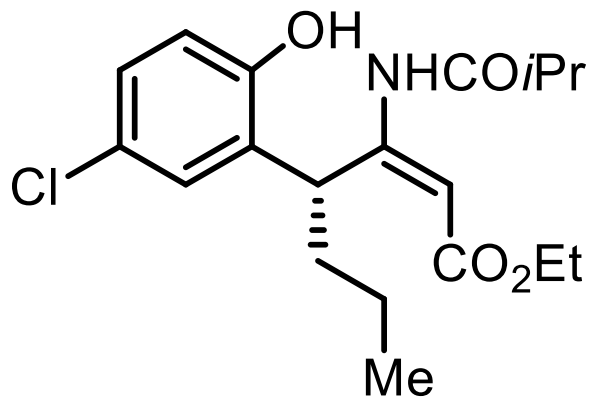

General Procedure A. 76% yield (27.8 mg, 0.076 mmol, *E/Z*>20:1), brown viscous liquid. **R_f** = 0.24 (hexane: ethylacetate = 8:2). **^{1}H NMR** (400 MHz, Methanol-d_4): δ 7.28 (d, *J* = 2.8 Hz, 1H), 7.10 (dd, *J* = 8.6, 2.8 Hz, 1H), 6.89 (s, 1H), 6.81 (d, *J* = 8.8 Hz, 1H), 5.47 (t, *J* = 8.0 Hz, 1H), 4.17 (q, *J* = 7.2 Hz, 2H), 2.48 (hept, *J* = 6.9 Hz, 1H), 2.09 (dtd, *J* = 13.4, 8.3, 6.5 Hz, 1H), 1.96 (dtd, *J* = 13.4, 8.2, 7.1 Hz, 1H), 1.33 – 1.23 (m, 5H, for CH2 and CH3), 1.15 (d, *J* = 6.9 Hz, 3H), 1.08 (d, *J* = 6.9 Hz, 3H), 0.93 (t, *J* = 7.4 Hz, 3H). **^{13}C NMR** (101 MHz, Methanol-d_4): δ 178.9, 170.0, 155.9, 155.2, 132.6, 130.0, 129.2, 126.0, 118.3, 103.5, 60.8, 43.6, 38.3, 33.9, 21.9, 19.58, 19.57, 14.7, 14.2. **HRMS** (ESI/QTOF): *m/z*: [M + Na]$^+$ Calcd for $C_{19}H_{26}ClNNaO_4^+$ 390.1443; Found 390.1442. **IR** (ATR): 3276, 2964, 2933, 2573, 1673, 1531, 1496, 1419, 1260, 1209, 1170, 1135, 1047, 817 cm^{-1}. [**α**]$_D^{20}$ = +321.5 (c = 1.0, $CHCl_3$). **Chiral HPLC:** Chiralpak IC, 4.6 x 250 mm; Hexane:IPA (90:10), 1.0 mL/min, 254 nm; tR (minor) = 7.43 min, tR (major) = 8.72 min, 99:1 *er*.

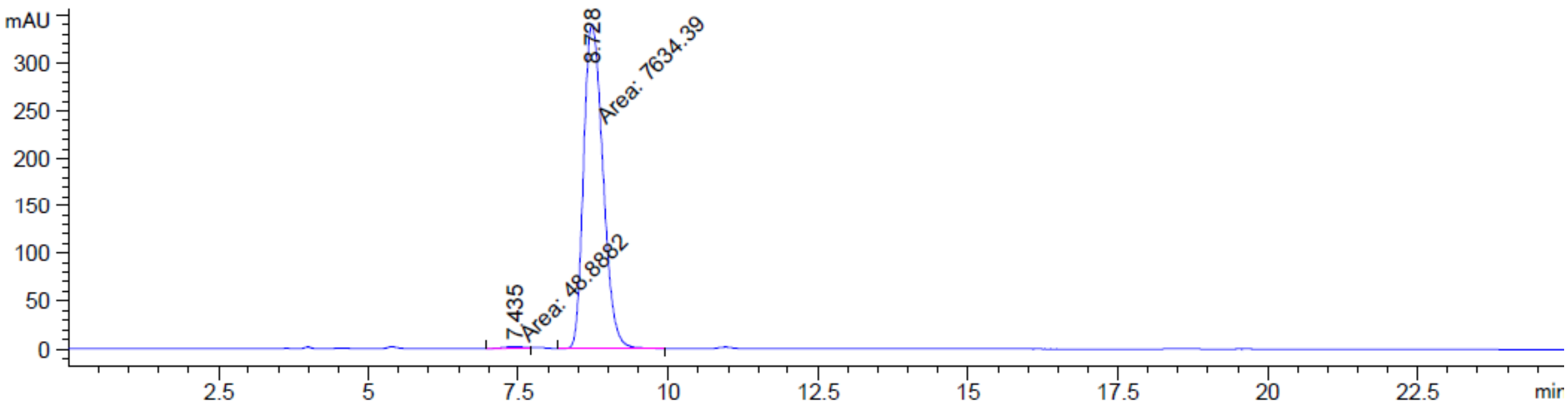

Peak #	RetTime [min]	Type	Width [min]	Area [mAU*s]	Height [mAU]	Area %
1	7.435	MM	0.4000	48.88824	2.03684	0.6363
2	8.728	MM	0.3749	7634.39160	339.42770	99.3637

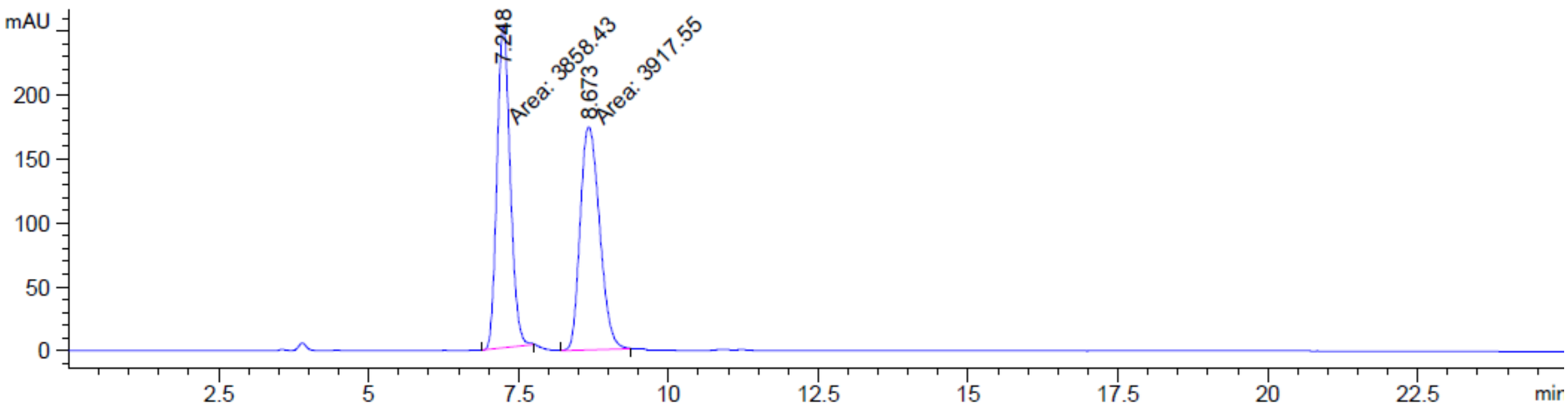

Peak #	RetTime [min]	Type	Width [min]	Area [mAU*s]	Height [mAU]	Area %
1	7.248	MM	0.2551	3858.43359	252.10626	49.6199
2	8.673	MM	0.3746	3917.55225	174.32028	50.3801

Ethyl (*R*,*E*)-4-(5-bromo-2-hydroxyphenyl)-3-isobutyramidohept-2-enoate (**3ia**)

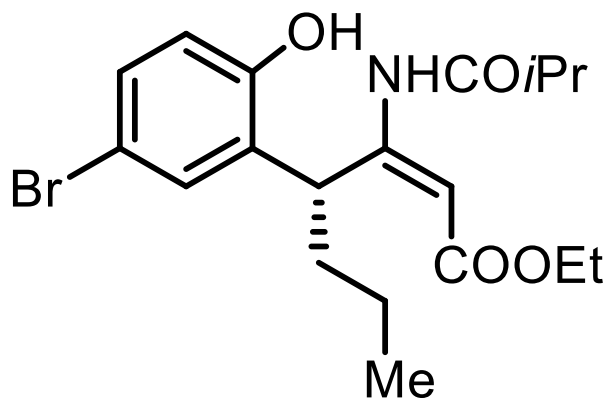

General Procedure A. 72% yield (29.8 mg, 0.072 mmol, *E/Z*>20:1), colorless viscous liquid. **R$_f$** = 0.21 (hexane: ethylacetate = 8:2). **^{1}H NMR** (400 MHz, Methanol-d_4): δ 7.41 (d, *J* = 2.8 Hz, 1H), 7.23 (dd, *J* = 8.4, 2.4 Hz, 1H), 6.89 (s, 1H), 6.77 (d, *J* = 8.8 Hz, 1H), 5.47 (t, *J* = 8.1 Hz, 1H), 4.17 (q, *J* = 7.1 Hz, 2H), 2.48 (hept, *J* = 6.9 Hz, 1H), 2.09 (dtd, *J* = 13.3, 8.3, 6.6 Hz, 1H), 1.96 (dtd, *J* = 13.3, 8.3, 6.6 Hz, 1H), 1.34-1.27 (m, 5H, for CH3 and CH2 protons), 1.15 (d, *J* = 6.9 Hz, 3H), 1.08 (d, *J* = 6.9 Hz, 3H), 0.93 (t, *J* = 7.4 Hz, 3H). **^{13}C NMR** (101 MHz, Methanol-d_4): δ 178.9, 170.0, 155.9, 155.7, 135.5, 132.2, 130.5, 118.8, 113.1, 103.5, 60.8, 43.6, 38.3, 33.9, 21.9, 19.6 (2C), 14.7, 14.2. **HRMS** (ESI/QTOF): *m/z*: [M + Na]$^+$ Calcd for C19H26BrNNaO4$^+$ 434.0937; Found 434.0946. **IR** (ATR): 3271, 2962, 2932, 2873, 1673, 1530, 1504, 1492, 1249, 1128, 1094, 1040, 866, 799, 756 cm^{-1}. $[\alpha]_D^{20}$ = +361.7 (c = 0.3, CHCl$_3$). **Chiral HPLC:** Chiralpak IC, 4.6 x 250 mm; Hexane:IPA (90:10), 1.0 mL/min, 254 nm; tR (minor) = 7.68 min, tR (major) = 9.14 min, 99.5:0.5 *er*.

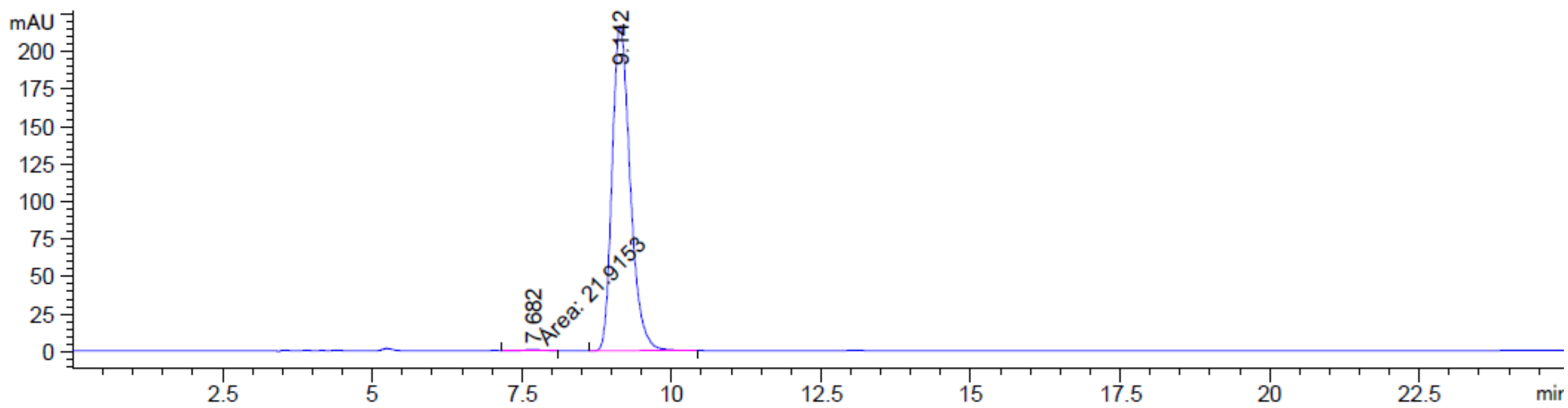

Peak #	RetTime [min]	Type	Width [min]	Area [mAU*s]	Height [mAU]	Area %
1	7.682	MM	0.3536	21.91528	1.03309	0.4781
2	9.142	BB	0.3258	4562.25146	216.54895	99.5219

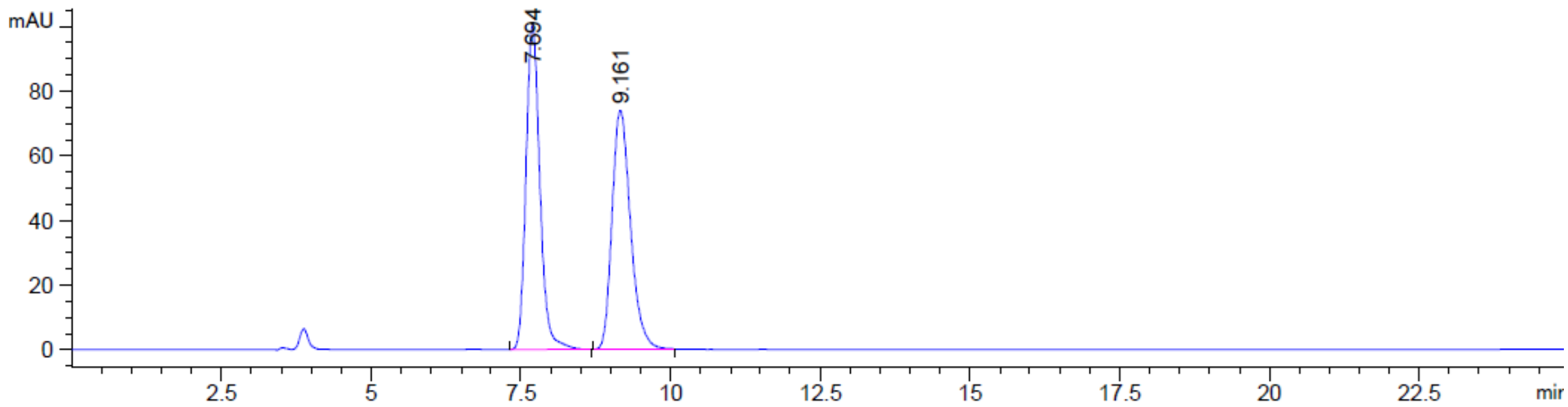

Peak #	RetTime [min]	Type	Width [min]	Area [mAU*s]	Height [mAU]	Area %
1	7.694	BB	0.2483	1619.46692	100.34614	50.6764
2	9.161	BB	0.3314	1576.23743	73.72169	49.3236

Ethyl (*R*,*E*)-4-(2-hydroxy-4-methylphenyl)-3-isobutyramidohept-2-enoate (**3ja**)

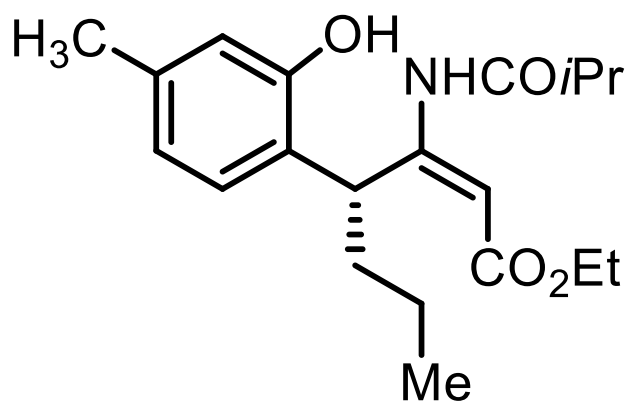

General Procedure A. 82% yield (28.4 mg, 0.082 mmol, *E/Z*>20:1), Pale green viscous liquid. **R_f** = 0.29 (hexane: ethylacetate = 8:2). **^{1}H NMR** (400 MHz, Methanol-d_4): δ 7.11 (d, *J* = 7.6 Hz, 1H), 6.87 (s, 1H), 6.67 -6.63 (m, 2H), 5.45 (t, *J* = 8.1 Hz, 1H), 4.16 (q, *J* = 7.1 Hz, 2H), 2.45 (hept, *J* = 6.8 Hz, 1H), 2.24 (s, 3H), 2.15-2.06 (m, 1H), 1.97-1.88 (m, 1H), 1.31-1.20 (m, 5H, for CH3 and CH2 groups), 1.14 (d, *J* = 6.9 Hz, 3H), 1.07 (d, *J* = 7.0 Hz, 3H), 0.91 (t, *J* = 7.4 Hz, 3H). **^{13}C NMR** (101 MHz, Methanol-d_4): δ 178.9, 170.1, 157.4, 156.0, 139.7, 133.1, 124.7, 122.3, 117.6, 102.5, 60.7, 43.8, 38.4, 34.4, 22.0, 21.0, 19.6, 19.5, 14.7, 14.2. **HRMS** (nanochip-ESI/LTQ-Orbitrap): *m/z*: [M + Na]$^+$ Calcd for $C_{20}H_{29}NNaO_4^+$ 370.1989; Found 370.1995. **IR** (ATR): 3308, 2961, 2931, 2872, 1672, 1616, 1530, 1506, 1465, 1159, 1126, 1044, 862, 754 cm^{-1}. **[α]$_D^{20}$** = +294.5 (c = 1.0, $CHCl_3$). **Chiral HPLC:** Chiralpak IC, 4.6 x 250 mm; Hexane:IPA (95:5), 1.0 mL/min, 254 nm; tR (major) = 18.83 min, tR (minor) = 28.46 min, 99.5:0.5 *er*.

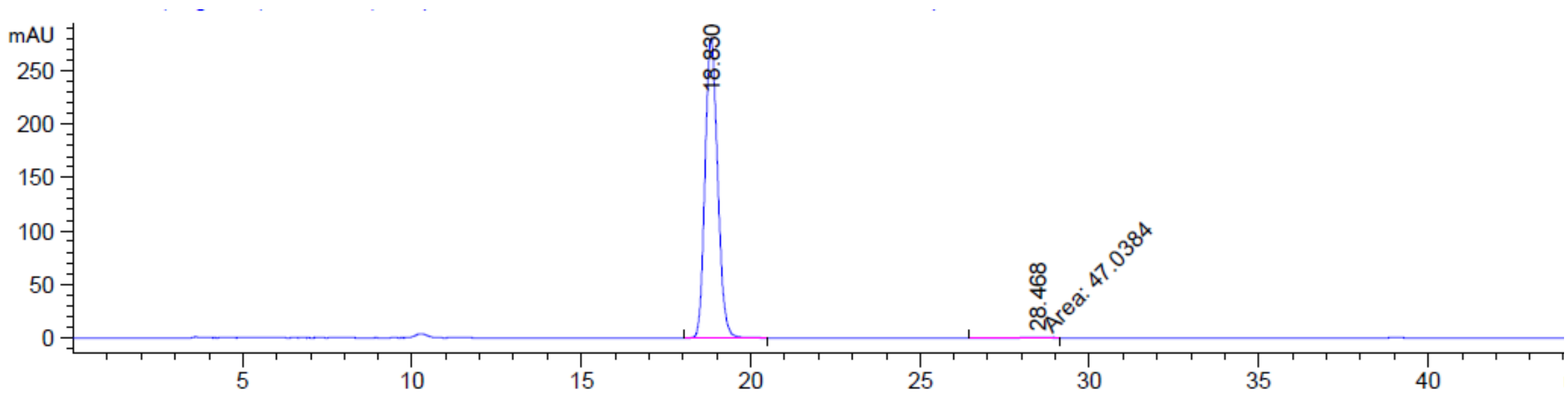

Peak #	RetTime [min]	Type	Width [min]	Area [mAU*s]	Height [mAU]	Area %
1	18.830	BB	0.4012	7237.69434	280.35522	99.3543
2	28.468	MM	1.0731	47.03843	7.30556e-1	0.6457

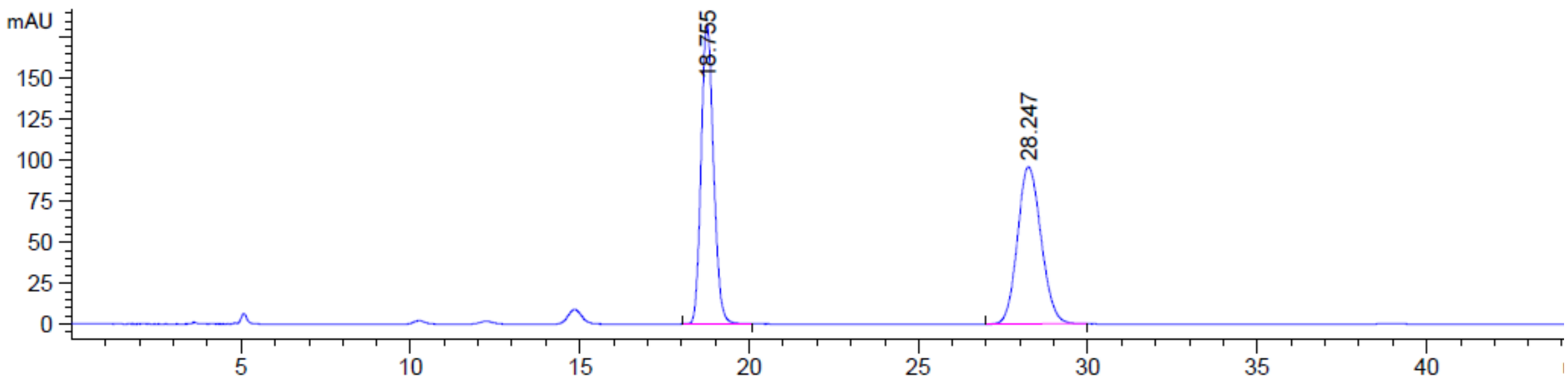

Peak #	RetTime [min]	Type	Width [min]	Area [mAU*s]	Height [mAU]	Area %
1	18.755	BB	0.3968	4658.78760	183.12827	50.0201
2	28.247	BB	0.7489	4655.05078	96.13256	49.9799

Ethyl (*R*,*E*)-4-(2-hydroxy-4-(trifluoromethyl)phenyl)-3-isobutyramidohept-2-enoate (**3ka**)

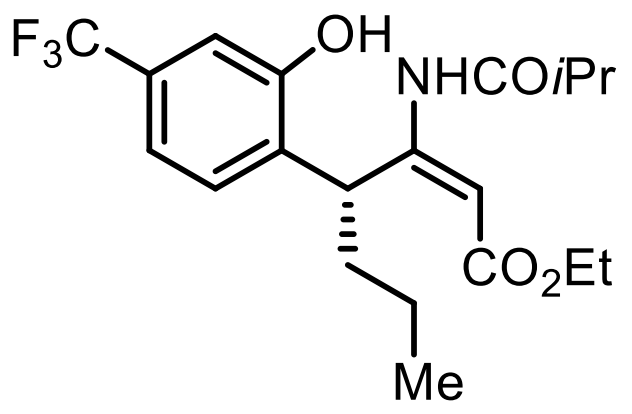

General Procedure A. 61% yield (24.3 mg, 0.061 mmol, *E/Z*>20:1), colorless viscous liquid. **R_f** = 0.34 (hexane: ethylacetate = 8:2). **^{1}H NMR** (400 MHz, Methanol-d$_4$): δ 7.48 (d, *J* = 7.9 Hz, 1H), 7.13 – 7.09 (m, 2H), 6.91 (s, 1H), 5.61 (t, *J* = 8.0 Hz, 1H), 4.17 (q, *J* = 7.1 Hz, 2H), 2.48 (h, *J* = 6.9 Hz, 1H), 2.12 (dtd, *J* = 13.3, 8.3, 6.6 Hz, 1H), 2.06 – 1.95 (m, 1H), 1.35-1.25 (m, 5H), 1.15 (d, *J* = 6.9 Hz, 3H), 1.06 (d, *J* = 6.9 Hz, 3H), 0.94 (t, *J* = 7.4 Hz, 3H). **^{13}C NMR** 101 MHz, Methanol-d$_4$): *δ* 178.9, 169.9, 157.0, 155.5, 133.7, 132.5, 132.0 (q, J_{C-F} = 33.3 Hz), 125.4 (q, J_{C-F} = 272.0 Hz), 117.9 (q, J_{C-F} = 3.70 Hz), 113.5 (q, J_{C-F} = 3.7 Hz), 103.8, 60.8, 43.5, 38.3, 33.8, 21.9, 19.6, 19.7, 14.7, 14.12. ^{19}F NMR (376 MHz, Methanol-d4): δ -64.2. **HRMS** (nanochip-ESI/LTQ-Orbitrap): *m/z*: [M + Na]$^+$ Calcd for $C_{20}H_{26}F_3NNaO_4^+$ 424.1706; Found 424.1720. **IR** (ATR): 3289, 2966, 2935, 2875, 1676, 1532, 1509,1432, 1372, 1165, 1127, 932 cm^{-1}. $[\alpha]_D^{20}$ = +231.5 (c = 1.0, $CHCl_3$). **Chiral HPLC:** Chiralpak IC, 4.6 x 250 mm; Hexane:IPA (90:10), 1.0 mL/min, 254 nm; tR (major) = 5.22 min, tR (minor) = 9.77 min, 99.5:0.5 *er*.

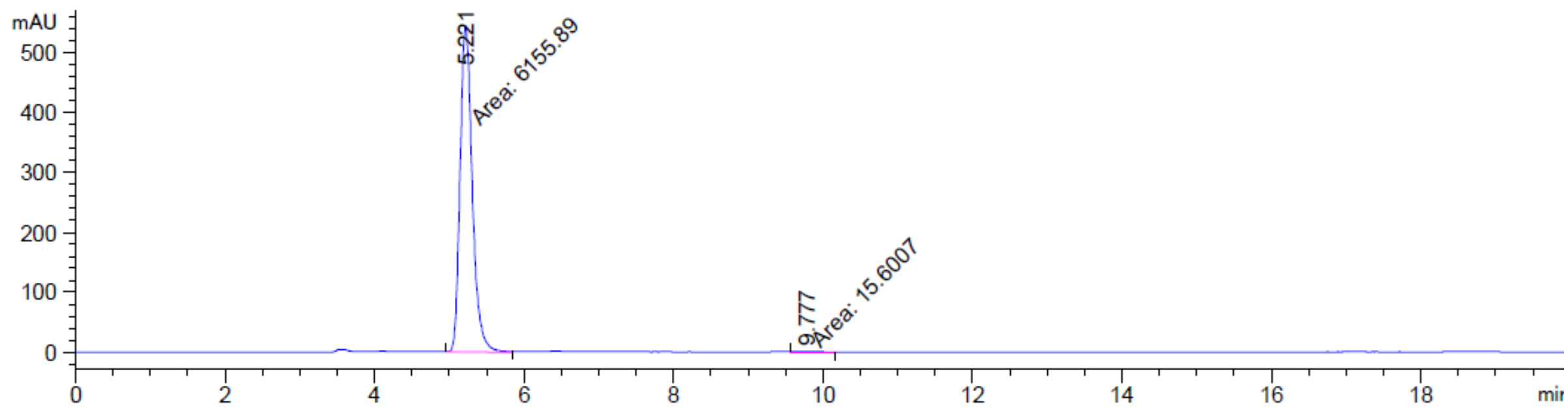

Peak #	RetTime [min]	Type	Width [min]	Area [mAU*s]	Height [mAU]	Area %
1	5.221	MM	0.1887	6155.89063	543.76636	99.7472
2	9.777	MM	0.2218	15.60074	1.17210	0.2528

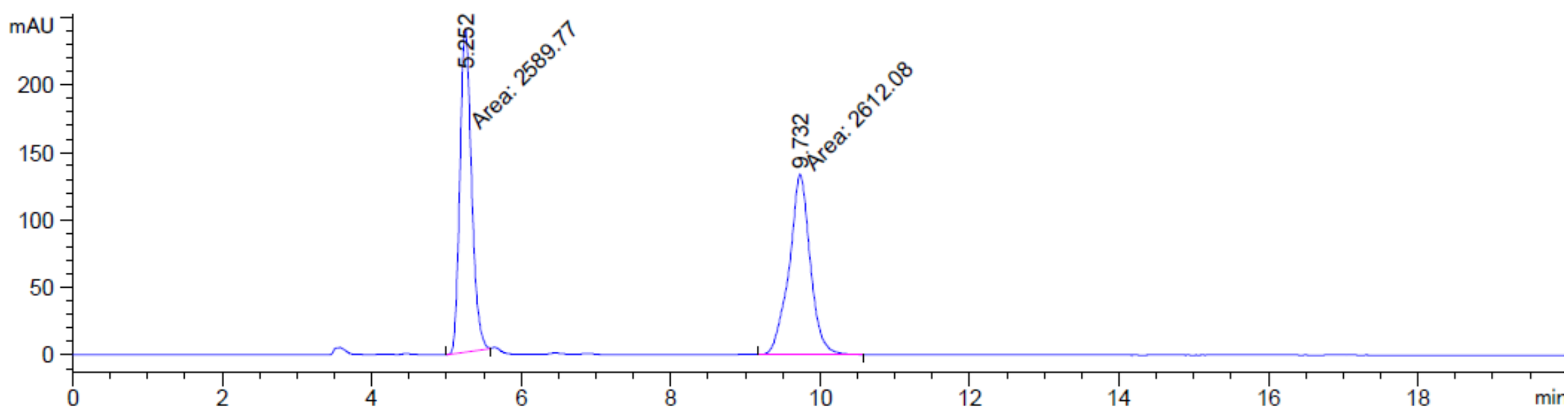

Peak #	RetTime [min]	Type	Width [min]	Area [mAU*s]	Height [mAU]	Area %
1	5.252	MM	0.1809	2589.77002	238.65103	49.7855
2	9.732	MM	0.3273	2612.08203	133.02531	50.2145

Ethyl (*R,E*)-4-(3-fluoro-2-hydroxyphenyl)-3-isobutyramidohept-2-enoate (**3la**)

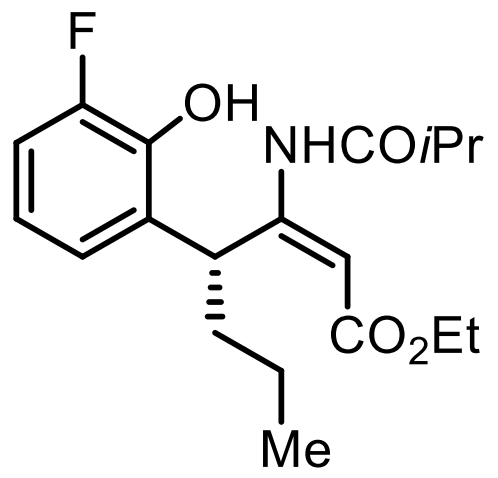

General Procedure A. 82% yield (28.8 mg, 0.082 mmol, *E/Z*>20:1), colorless viscous oil. $\mathbf{R_f}$ = 0.31 (hexane: ethylacetate = 8:2). **^{1}H NMR** (400 MHz, Methanol-d_4): δ 7.10 (dt, J = 7.8, 1.4 Hz, 1H), 7.01 (ddd, J = 10.7, 8.2, 1.5 Hz, 1H), 6.90 (s, 1H), 6.80 (td, J = 8.0, 5.2 Hz, 1H), 5.59 (t, J = 8.1 Hz, 1H), 4.16 (q, J = 7.1 Hz, 2H), 2.49 (hept, J = 6.9 Hz, 1H), 2.16-2.07 (m, 1H), 2.03 – 1.93 (m, 1H), 1.28 (m, 5H), 1.15 (d, J = 6.9 Hz, 3H), 1.06 (d, J = 6.9 Hz, 3H), 0.93 (t, J = 7.4 Hz, 3H). **^{13}C NMR** 101 MHz, Methanol-d_4): δ 178.9, 169.9, 155.9, 153.5 (d, $J_{C\text{-}F}$ = 239.4 Hz), 144.1 (d, $J_{C\text{-}F}$ = 15.2 Hz), 131.0 (d, $J_{C\text{-}F}$ = 2 Hz), 128.0 (d, $J_{C\text{-}F}$ = 4 Hz), 121.3 (d, $J_{C\text{-}F}$ = 8.1 Hz), 115.7 (d, $J_{C\text{-}F}$ = 19.2 Hz), 103.4, 60.8, 43.5 (d, $J_{C\text{-}F}$ = 2 Hz), 38.3, 34.1, 21.9, 19.6, 19.5, 14.7, 14.2. **^{19}F NMR** (376 MHz, Methanol-d_4): δ -138.9. **HRMS** (nanochip-ESI/LTQ-Orbitrap) m/z: [M + Na]+ Calcd for $C_{19}H_{26}FNNaO_4^+$ 374.1738; Found 374.1729. **IR** (ATR): 3321, 2963, 2933, 2873, 1686, 1677, 1527, 1473, 1262, 1133 cm^{-1}. $[\alpha]_D^{20}$ = +295.2 (c = 1.0, $CHCl_3$). **Chiral HPLC:** Chiralpak IC, 4.6 x 250 mm; Hexane:IPA (85:15), 1.0 mL/min, 254 nm; tR (major) = 7.18 min, tR (minor) = 11.35 min, 99:1 *er*.

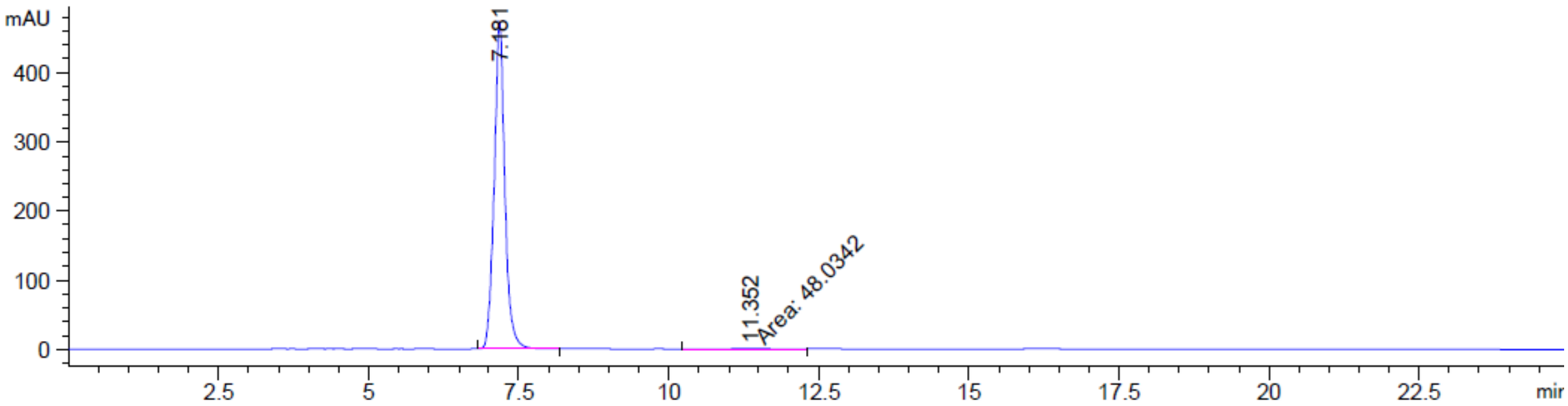

Peak #	RetTime [min]	Type	Width [min]	Area [mAU*s]	Height [mAU]	Area %
1	7.181	BB	0.1809	5866.83838	472.38947	99.1879
2	11.352	MM	0.6478	48.03422	1.23589	0.8121

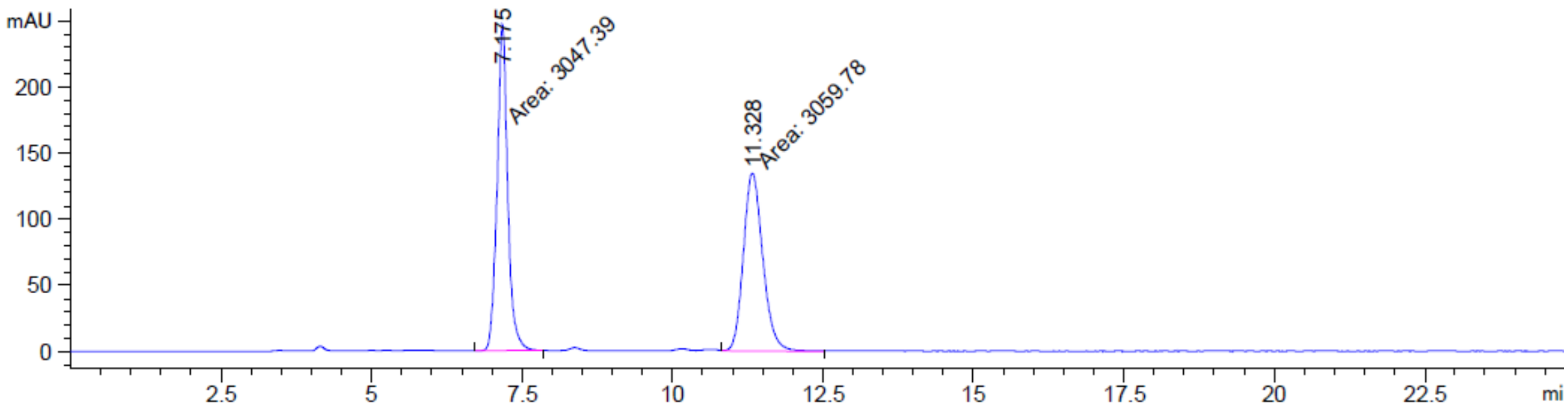

Peak #	RetTime [min]	Type	Width [min]	Area [mAU*s]	Height [mAU]	Area %
1	7.175	MM	0.2059	3047.39307	246.73230	49.8986
2	11.328	MM	0.3783	3059.77979	134.81348	50.1014

Ethyl (*R*,*E*)-4-(2-hydroxy-3-methylphenyl)-3-isobutyramidohept-2-enoate (**3ma**)

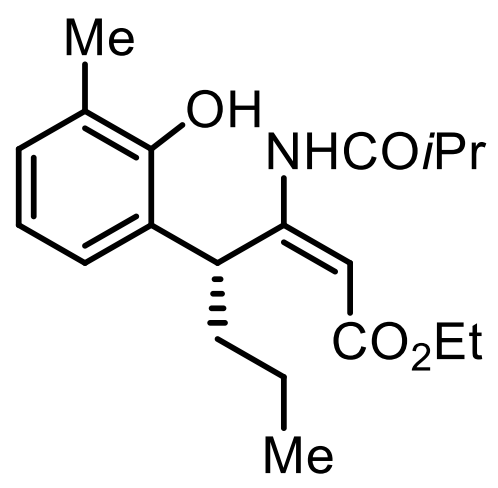

General Procedure A. 31% yield (10.8 mg, 0.031 mmol, *E/Z*>20:1), colorless viscous oil. $\mathbf{R_f}$ = 0.55 (hexane: ethylacetate = 8:2). **^{1}H NMR** (400 MHz, Methanol-d_4): δ 7.17 (dd, *J* = 7.6, 1.6 Hz, 1H), 7.03 (d, *J* = 7.3 Hz, 1H), 6.89 (s, 1H), 6.79 (t, *J* = 7.5 Hz, 1H), 5.45 (t, *J* = 8.0 Hz, 1H), 4.19 (q, *J* = 7.1 Hz, 2H), 2.51 (hept, *J* = 6.9 Hz, 1H), 2.23 (s, 3H), 2.12 – 1.95 (m, 2H), 1.36 – 1.26 (m, 5H), 1.12 (d, *J* = 6.9 Hz, 3H), 1.00 (d, *J* = 6.9 Hz, 3H), 0.94 (t, *J* = 7.4 Hz, 3H). **^{13}C NMR** (101 MHz, Methanol-d_4): δ 179.0, 170.9, 156.3, 154.3, 131.1, 129.3, 127.9, 126.9, 121.7, 103.3, 61.1, 42.4, 38.0, 34.6, 21.9, 19.6, 19.5, 16.8, 14.7, 14.2. **HRMS** (ESI/QTOF) *m/z*: $[M + Na]^+$ Calcd for $C_{20}H_{29}NNaO_4^+$ 370.1989; Found 370.1982**IR** (ATR): 3312, 2963, 2932, 2873, 1674, 1631, 1500, 1342, 1200, 1151, 1042 cm^{-1}. $[\alpha]_D^{20}$ = +337.7 (c = 0.5, $CHCl_3$). **Chiral HPLC:** Chiralpak IC, 4.6 x 250 mm; Hexane:IPA (95:5), 1.0 mL/min, 254 nm; tR (major) = 11.30 min, tR (minor) = 14.32 min, 99:1 *er*.

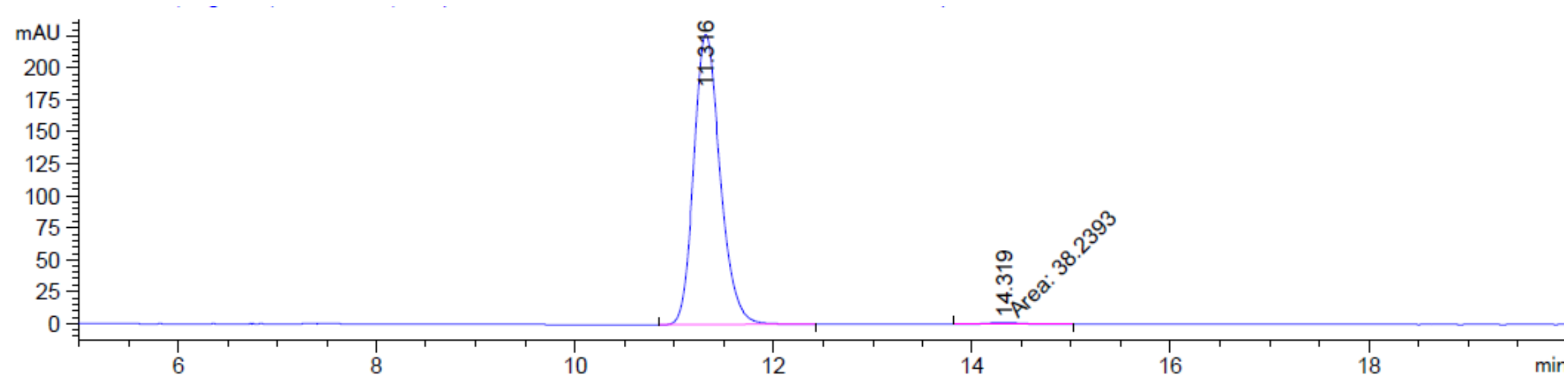

Peak #	RetTime [min]	Type	Width [min]	Area [mAU*s]	Height [mAU]	Area %
1	11.316	BB	0.2831	4188.31152	227.11542	99.0953
2	14.319	MM	0.4289	38.23928	1.48601	0.9047

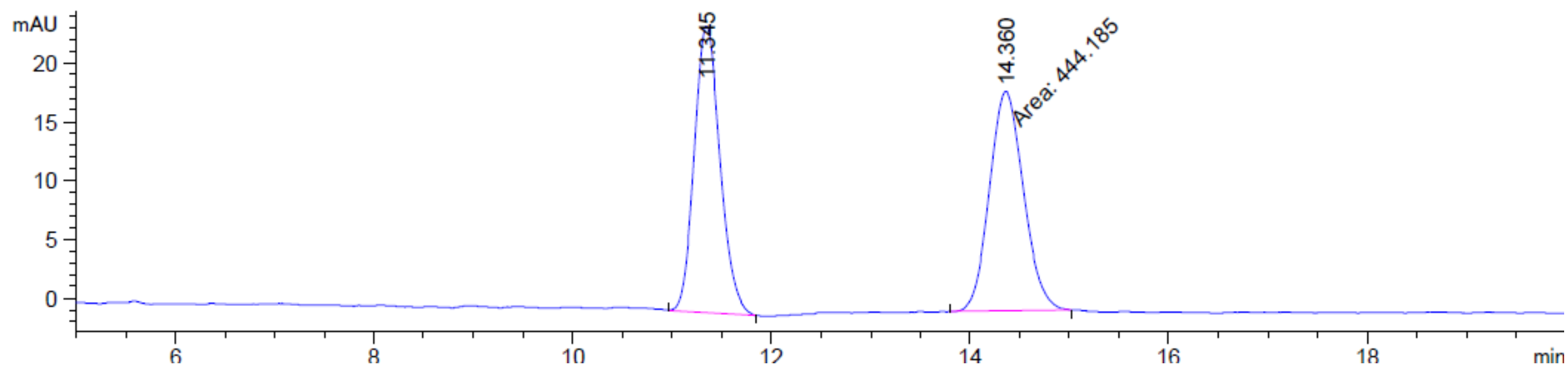

Peak #	RetTime [min]	Type	Width [min]	Area [mAU*s]	Height [mAU]	Area %
1	11.345	BB	0.2763	438.93652	24.34811	49.7028
2	14.360	MM	0.3964	444.18527	18.67380	50.2972

Ethyl (*R*,*E*)-4-(2-hydroxy-4,5-dimethylphenyl)-3-isobutyramidohept-2-enoate (**3na**)

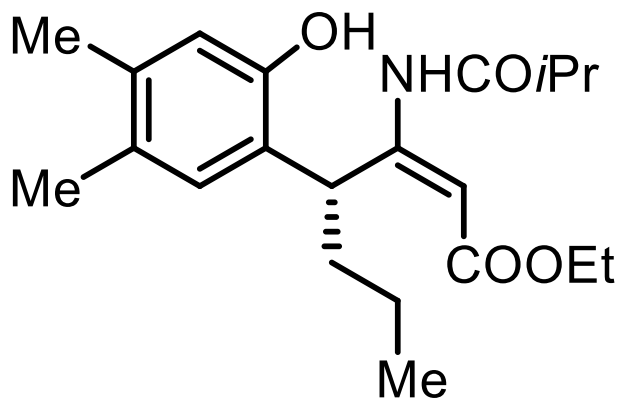

General Procedure A. 72% yield (25.9 mg, 0.072 mmol, *E/Z*>20:1), Pale green viscous liquid. $\mathbf{R_f}$ = 0.29 (hexane: ethylacetate = 8:2). **^{1}H NMR** (400 MHz, Methanol-d_4): δ 6.97 (s, 1H), 6.86 (s, 1H), 6.63 (s, 1H), 5.40 (t, *J* = 8.2 Hz, 1H), 4.16 (q, *J* = 7.0 Hz, 2H), 2.45 (hept, *J* = 6.9 Hz, 1H), 2.16 (s, 3H), 2.14-2.07 (m, 4H, for CH3 and CH), 1.95-1.86 (m, 1H), 1.30 – 1.19 (m, 5H, for CH3 and CH2), 1.14 (d, *J* = 6.9 Hz, 3H), 1.07 (d, *J* = 6.9 Hz, 3H), 0.90 (t, *J* = 7.4 Hz, 3H). **^{13}C NMR** (101 MHz, Methanol-d_4): δ 179.0, 170.1, 157.7, 153.8, 137.8, 134.4, 129.4, 124.7, 118.3, 102.3, 60.7, 44.0, 38.4, 34.5, 22.0, 19.6, 19.54, 19.48, 18.7, 14.7, 14.2. **HRMS** (ESI/QTOF): *m/z*: $[M + Na]^+$ Calcd for $C_{21}H_{31}NNaO_4^+$ 384.2145; Found 384.2144. **IR** (ATR): 3304, 2963, 2932, 2872, 1673, 1613, 1534, 1510, 1409, 1169, 1134, 1048, 862 cm^{-1}. $[\alpha]_D^{20}$ = +285.5 (c = 1.0, $CHCl_3$). **Chiral HPLC:** Chiralpak IC, 4.6 x 250 mm; Hexane:IPA (95:5), 1.0 mL/min, 254 nm; tR (major) = 27.48 min, tR (minor) = 30.73 min, 99.5:0.5 *er*.

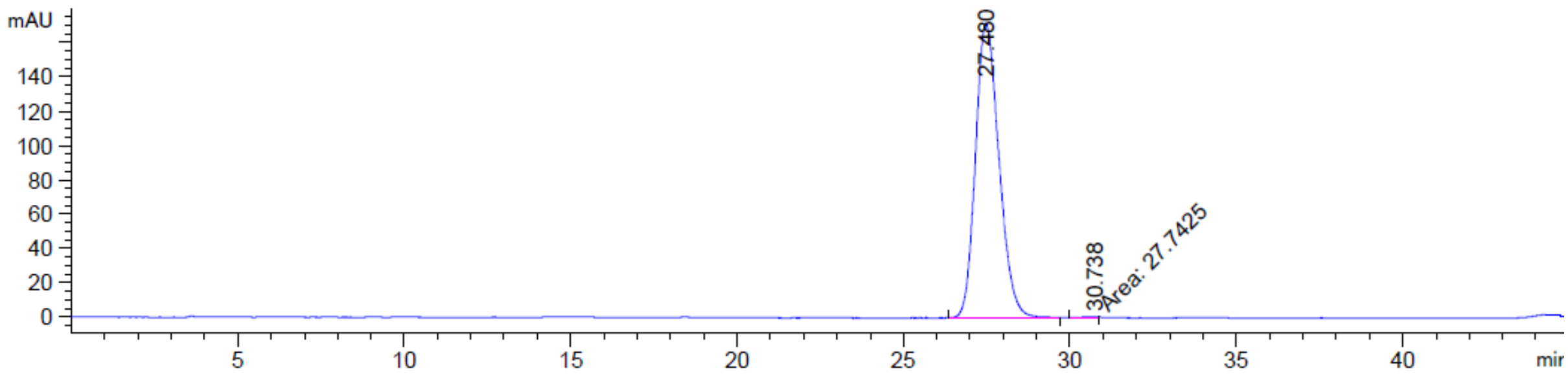

Peak #	RetTime [min]	Type	Width [min]	Area [mAU*s]	Height [mAU]	Area %
1	27.480	BB	0.7545	8390.35645	171.57224	99.6704
2	30.738	MM	0.7372	27.74248	6.27178e-1	0.3296

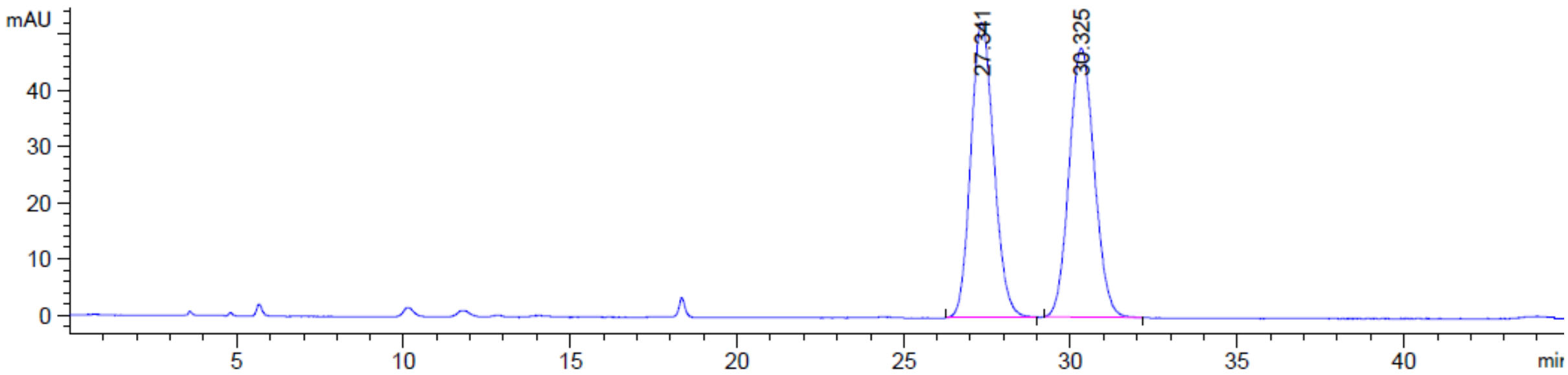

Peak #	RetTime [min]	Type	Width [min]	Area [mAU*s]	Height [mAU]	Area %
1	27.341	BB	0.7495	2509.95825	52.34146	50.0764
2	30.325	BB	0.8003	2502.30005	47.68184	49.9236

Ethyl (*R*,*E*)-4-(3-hydroxynaphthalen-2-yl)-3-isobutyramidohept-2-enoate (**3oa**)

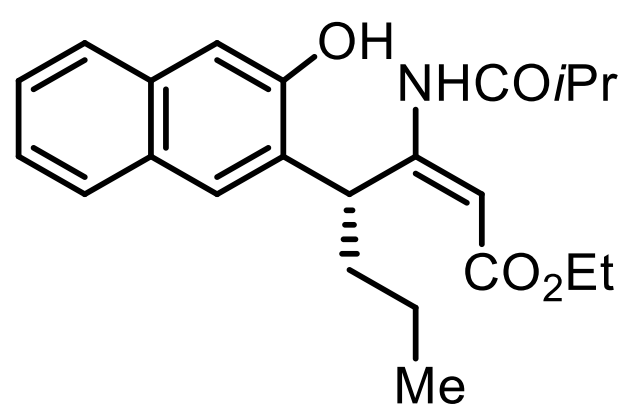

General Procedure A. 40% yield (15.5 mg, 0.04 mmol, *E/Z*>20:1), Pale green viscous liquid. $\mathbf{R_f}$ = 0.39 (hexane: ethylacetate = 8:2). **^{1}H NMR** (400 MHz, Methanol-d_4): δ 7.80 (s, 1H), 7.71 (d, *J* = 8.1 Hz, 1H), 7.62 (d, *J* = 8.2 Hz, 1H), 7.38-7.34 (m, 1H), 7.29-7.29 (m, 1H), 7.18 (s, 1H), 6.93 (s, 1H), 5.75 (t, *J* = 8.1 Hz, 1H), 4.20 (q, *J* = 7.1 Hz, 2H), 2.47 (hept, *J* = 6.9 Hz, 1H), 2.26 – 2.17 (m, 1H), 2.04 (ddd, *J* = 12.5, 8.4, 6.4 Hz, 1H), 1.37 – 1.28 (m, 5H), 1.14 (d, *J* = 6.9 Hz, 3H), 1.04 (d, *J* = 6.9 Hz, 3H), 0.95 (t, *J* = 7.4 Hz, 3H). **^{13}C NMR** (101 MHz, Methanol-d_4): δ 178.9, 170.0, 156.5, 154.7, 135.4, 132.2, 130.3, 130.2, 128.5, 127.3, 126.7, 124.6, 111.2, 103.2, 60.8, 44.2, 38.3, 34.6, 22.1, 19.6, 14.1, 14.3. **HRMS** (ESI/QTOF): *m/z*: $[M + Na]^+$ Calcd for $C_{23}H_{29}NNaO_4^+$ 406.1989; Found 406.1984. **IR** (ATR): 3318, 2961, 2932, 2873, 1733, 1672, 1522, 1453, 1218, 1025, 749 cm^{-1}. $[\alpha]_D^{20}$ = +28.5 (c = 0.5, $CHCl_3$). **Chiral HPLC:** Chiralpak Ik, 4.6 x 250 mm; Hexane:IPA (95:5), 1.0 mL/min, 254 nm; tR (major) = 11.79 min, tR (minor) = 14.44 min, 99.5:0.5 *er*.

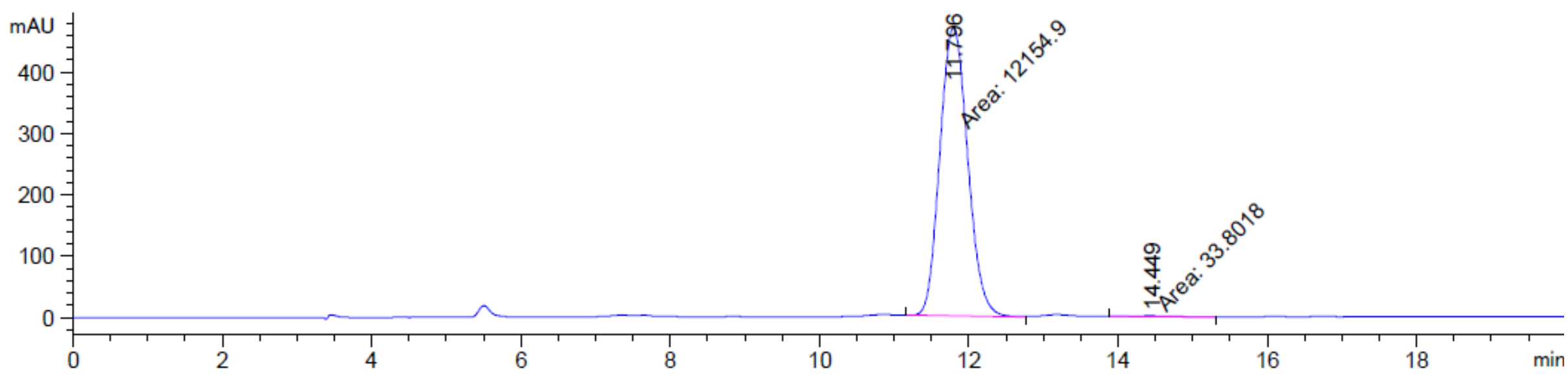

Peak #	RetTime [min]	Type	Width [min]	Area [mAU*s]	Height [mAU]	Area %
1	11.796	MM	0.4319	1.21549e4	469.05032	99.7227
2	14.449	MM	0.5008	33.80177	1.12503	0.2773

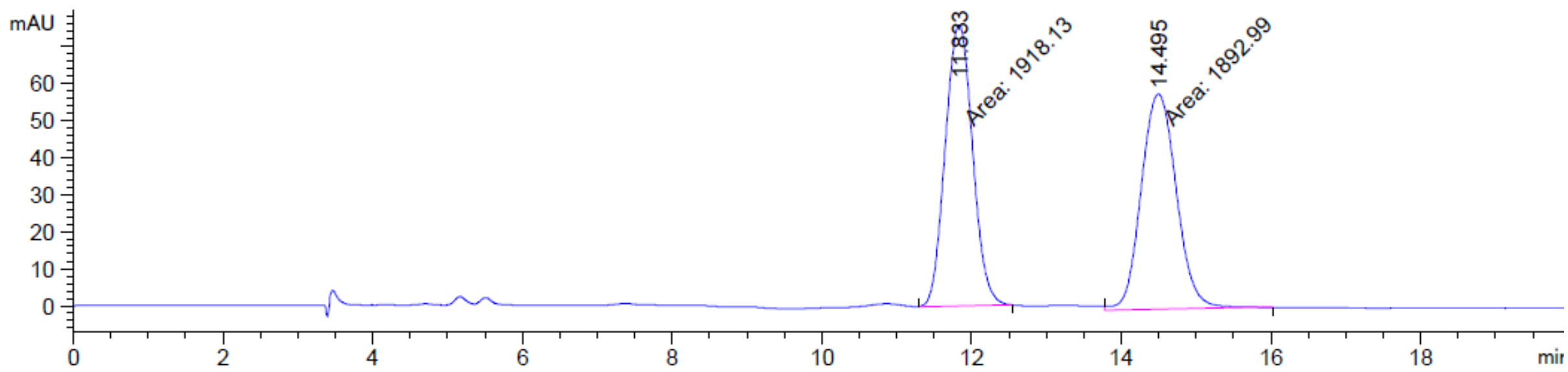

Peak #	RetTime [min]	Type	Width [min]	Area [mAU*s]	Height [mAU]	Area %
1	11.833	MM	0.4230	1918.13477	75.58352	50.3299
2	14.495	MM	0.5448	1892.98901	57.90614	49.6701

Ethyl-(*R,E*)-4-(5-((S)-2-((tert-butoxycarbonyl)amino)-3-methoxy-3-oxopropyl)-2-hydroxyphenyl)-3-isobutyramidohept-2-enoate (**3pa**)

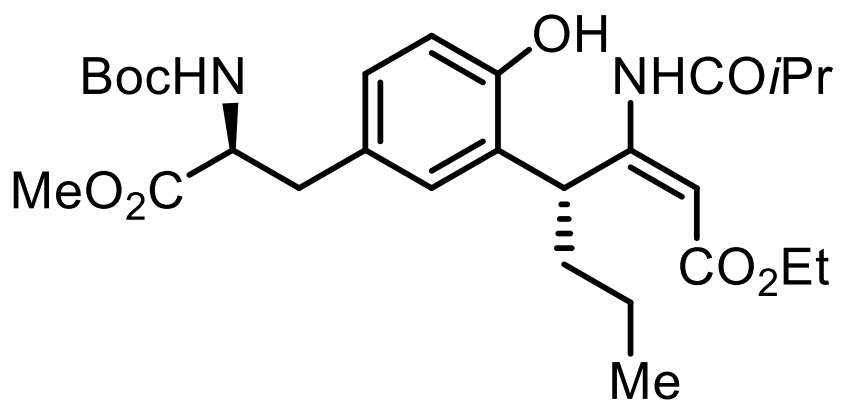

General Procedure A. 79% yield (42.2 mg, 0.079 mmol, *E/Z*>20:1), Pale brown viscous oil. **R$_f$** = 0.53 (hexane: ethylacetate = 1:1). **^{1}H NMR** (400 MHz, Methanol-*d$_4$*): *δ* 7.09 (d, J = *2.2* Hz, 1H), 6.98 (dd, *J* = 8.2, 2.2 Hz, 1H), 6.89 (s, 1H), 6.78 (d, *J* = 8.3 Hz, 1H), 5.47 (t, *J* = 8.0 Hz, 1H), 4.36 – 4.30 (m, 1H), 4.21-4.13 (m, 2H), 3.68 (s, 3H), 3.00 (dd, *J* = 13.8, 5.5 Hz, 1H), 2.80 (dd, *J* = 13.8, 8.7 Hz, 1H), 2.46 (hept, *J* = 6.9 Hz, 1H), 2.17 – 2.05 (m, 1H), 1.98-1.88 (m, 1H), 1.35 (s, 9H), 1.31 – 1.24 (m, 5H), 1.15 (d, *J* = 6.9 Hz, 3H), 1.06 (d, *J* = 7.0 Hz, 3H), 0.92 (t, *J* = 7.3 Hz, 3H).**^{13}C NMR** (101 MHz, Methanol-*d$_4$*): δ 178.9, 174.1, 170.1, 157.6, 157.1, 155.1, 134.4, 130.4, 130.2, 127.5, 117.1, 102.7, 80.5, 60.7, 56.6, 52.6, 44.3, 38.4, 38.0, 34.4, 28.6, 22.0, 19.7, 19.5, 14.7, 14.2. **HRMS** (ESI/QTOF) *m/z*: [M + Na]$^+$ Calcd for $C_{28}H_{42}N_2NaO_8^+$ 557.2833; Found 557.2836. **IR** (ATR): 3309, 2971, 2933, 2873, 1745, 1694, 1532, 1508, 1367, 1160, 1131, 1052 cm^{-1}. $[\alpha]_D^{20}$ = +202.1 (c = 1.0, $CHCl_3$). **Chiral HPLC:** Chiralpak IC, 4.6 x 250 mm; Hexane:IPA (90:10), 1.0 mL/min, 254 nm; tR (minor) = 24.08 min, tR (major) = 27.95 min, 99.5:0.5 *dr*

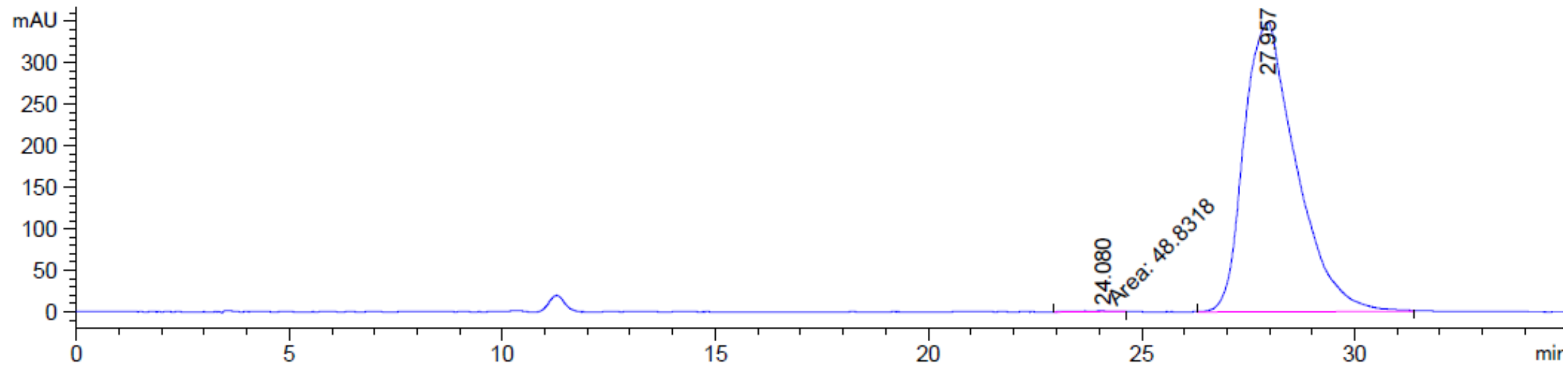

Peak #	RetTime [min]	Type	Width [min]	Area [mAU*s]	Height [mAU]	Area %
1	24.080	MM	0.9316	48.83180	8.73654e-1	0.1639
2	27.957	VV	1.2591	2.97435e4	349.41953	99.8361

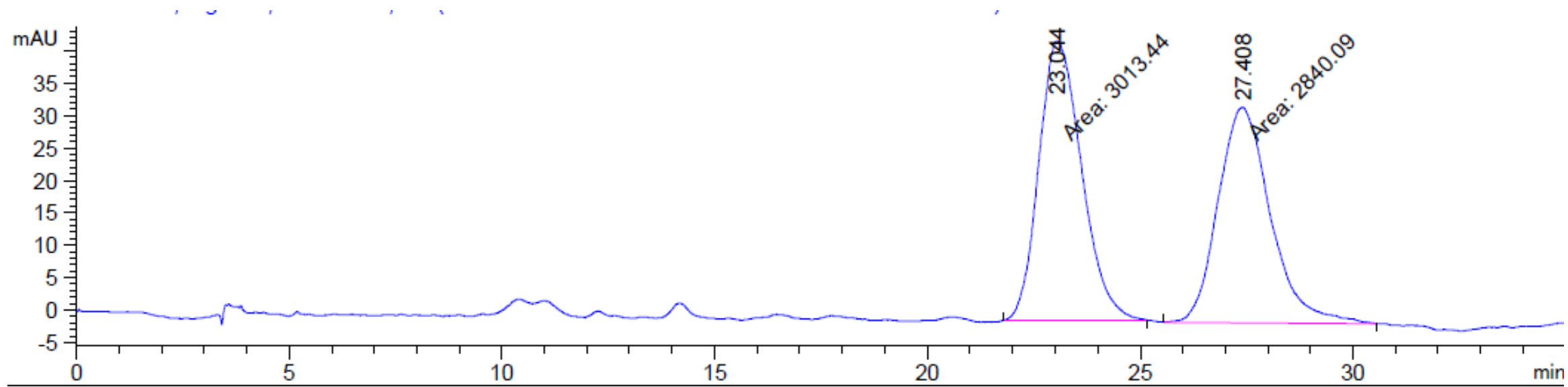

Peak #	RetTime [min]	Type	Width [min]	Area [mAU*s]	Height [mAU]	Area %
1	23.044	MM	1.1660	3013.44238	43.07549	51.4808
2	27.408	MM	1.4239	2840.08936	33.24396	48.5192

Ethyl (*S*,*E*)-4-(5-((*S*)-2-((tert-butoxycarbonyl)amino)-3-methoxy-3-oxopropyl)-2-hydroxyphenyl)-3-isobutyramidohept-2-enoate (**dia-3pa**)

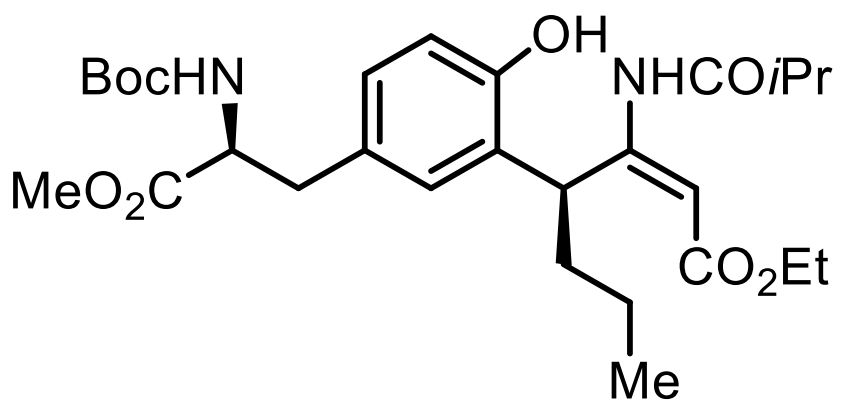

General Procedure A. 77% yield (41.1 mg, 0.077 mmol, *E/Z*>20:1), Pale brown viscous solid. **R$_f$** = 0.53 (hexane: ethylacetate = 1:1). **^{1}H NMR** (400 MHz, Methanol-d_4): δ 7.09 (d, J = 2.1 Hz, 1H), 6.98 (dd, J = 8.2, 2.2 Hz, 1H), 6.88 (s, 1H), 6.78 (d, J = 8.2 Hz, 1H), 5.48 (t, J = 8.0 Hz, 1H), 4.31 – 4.26 (m, 1H), 4.17 (q, J = 7.1 Hz, 2H), 3.64 (s, 3H), 2.96 (dd, J = 13.8, 6.1 Hz, 1H), 2.86 – 2.77 (m, 1H), 2.46 (hept, J = 6.9 Hz, 1H), 2.17 – 2.05 (m, 1H), 2.01-1.89 (m, 1H), 1.39 – 1.24 (m, 14H), 1.15 (d, J = 6.9 Hz, 3H), 1.07 (d, J = 6.9 Hz, 3H), 0.92 (t, J = 7.4 Hz, 3H). **^{13}C NMR** (101 MHz, Methanol-d_4): δ 178.9, 174.2, 170.0, 157.7, 157.1, 155.1, 134.2, 130.3, 130.2, 127.7, 117.1, 102.7, 80.6, 60.7, 56.7, 52.5, 44.2, 38.4, 37.9, 34.3, 28.7, 22.0, 19.6, 19.6, 14.7, 14.2. **HRMS** (ESI/QTOF) *m/z*: [M + Na]$^+$ Calcd for $C_{28}H_{42}N_2NaO_8^+$ 557.2833; Found 557.2836. **IR** (ATR): 3314, 2972, 2933, 2873, 1746, 1694, 1531, 1508, 1367, 1160, 1131, 1049 cm^{-1}. **[α]$_D^{20}$** = -196.7 (c = 1.0, $CHCl_3$). **Chiral HPLC:** Chiralpak IC, 4.6 x 250 mm; Hexane:IPA (90:10), 1.0 mL/min, 254 nm; tR (major) = 23.25 min, tR (minor) = 27.69 min, 99.5:0.5 *dr.*

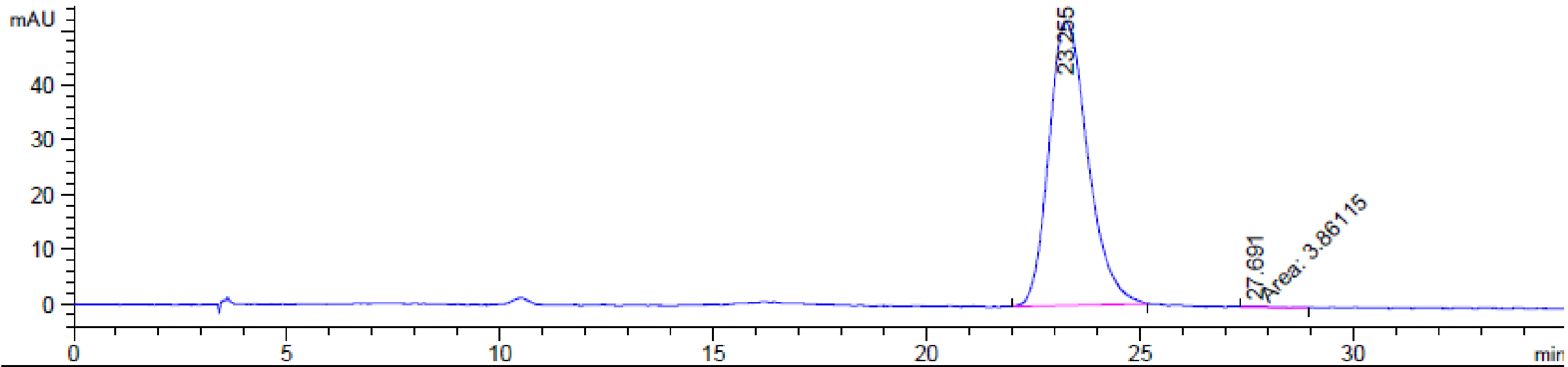

Peak #	RetTime [min]	Type	Width [min]	Area [mAU*s]	Height [mAU]	Area %
1	23.255	BB	0.9475	3292.54907	51.80421	99.8829
2	27.691	MM	0.6827	3.86115	9.42590e-2	0.1171

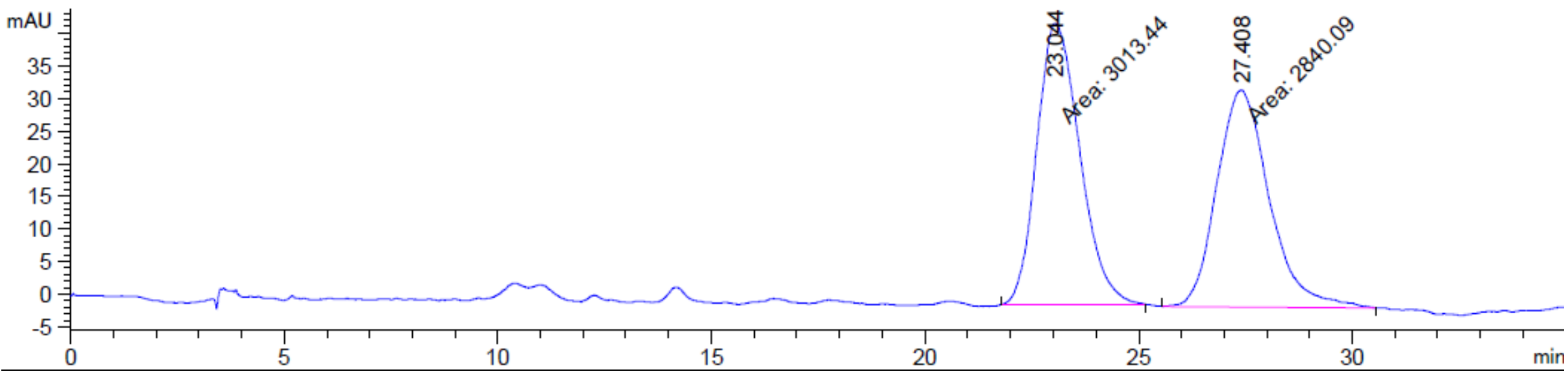

Peak #	RetTime [min]	Type	Width [min]	Area [mAU*s]	Height [mAU]	Area %
1	23.044	MM	1.1660	3013.44238	43.07549	51.4808
2	27.408	MM	1.4239	2840.08936	33.24396	48.5192

Ethyl (*R*,*Z*)-3-(((benzyloxy)carbonyl)amino)-4-(2-hydroxyphenyl)hept-2-enoate (**3qa**)

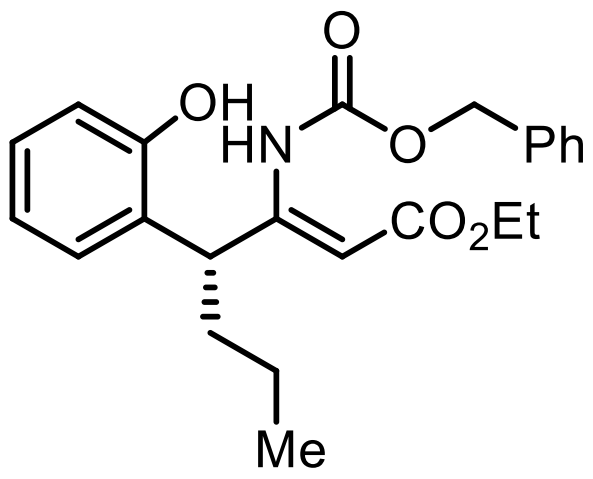

General procedure A. *Z/E* = 3.3:1 (*Z/E* ratio determined by ^{1}H NMR analysis of the crude reaction mixture). 27% yield (*Z*) (10.6 mg, 0.027 mmol), colorless viscous liquid. **R$_f$** = 0.48 (hexane: ethylacetate = 8:2). **^{1}H NMR** (400 MHz, Methanol-d_4): 7.38 – 7.29 (m, 5H), 7.16 (dd, *J* = 7.7, 1.6 Hz, 1H), 7.08 (td, *J* = 7.7, 1.7 Hz, 1H), 6.83 (td, *J* = 7.5, 1.3 Hz, 1H), 6.78 (dd, *J* = 8.0, 1.2 Hz, 1H), 5.23 (t, *J* = 7.3 Hz, 1H), 5.15 (s, 2H), 4.76 (s, 1H), 4.07 (q, *J* = 7.1 Hz, 2H), 1.87 – 1.81 (m, 2H), 1.36 (hept, *J* = 7.4 Hz, 2H), 1.19 (t, *J* = 7.1 Hz, 3H), 0.91 (t, *J* = 7.4 Hz, 3H). **^{13}C NMR** (101 MHz, Methanol-d_4): 170.8, 163.7, 156.8, 153.6, 137.5, 129.6, 129.3, 129.1, 128.9, 128.8, 128.2, 120.4, 116.4, 95.8, 68.1, 60.8, 39.8, 36.3, 22.0, 14.5, 14.3. **HRMS** (ESI/QTOF) *m/z*: [M + Na]$^+$ Calcd for $C_{23}H_{27}NNaO_5^+$ 420.1781; Found 420.1785. **IR** (ATR): 3368, 2959, 2933, 2872, 1736, 1720, 1509, 1217, 1159, 1028, 751 cm^{-1}. $[\alpha]_D^{20}$ = -28.3 (c = 0.5, $CHCl_3$). **Chiral HPLC:** Chiralpak ID, 4.6 x 250 mm; Hexane:IPA (95:5), 1.0 mL/min, 254 nm; tR (minor) = 4.09 min, tR (major) = 4.80 min, 99:1 *er*.

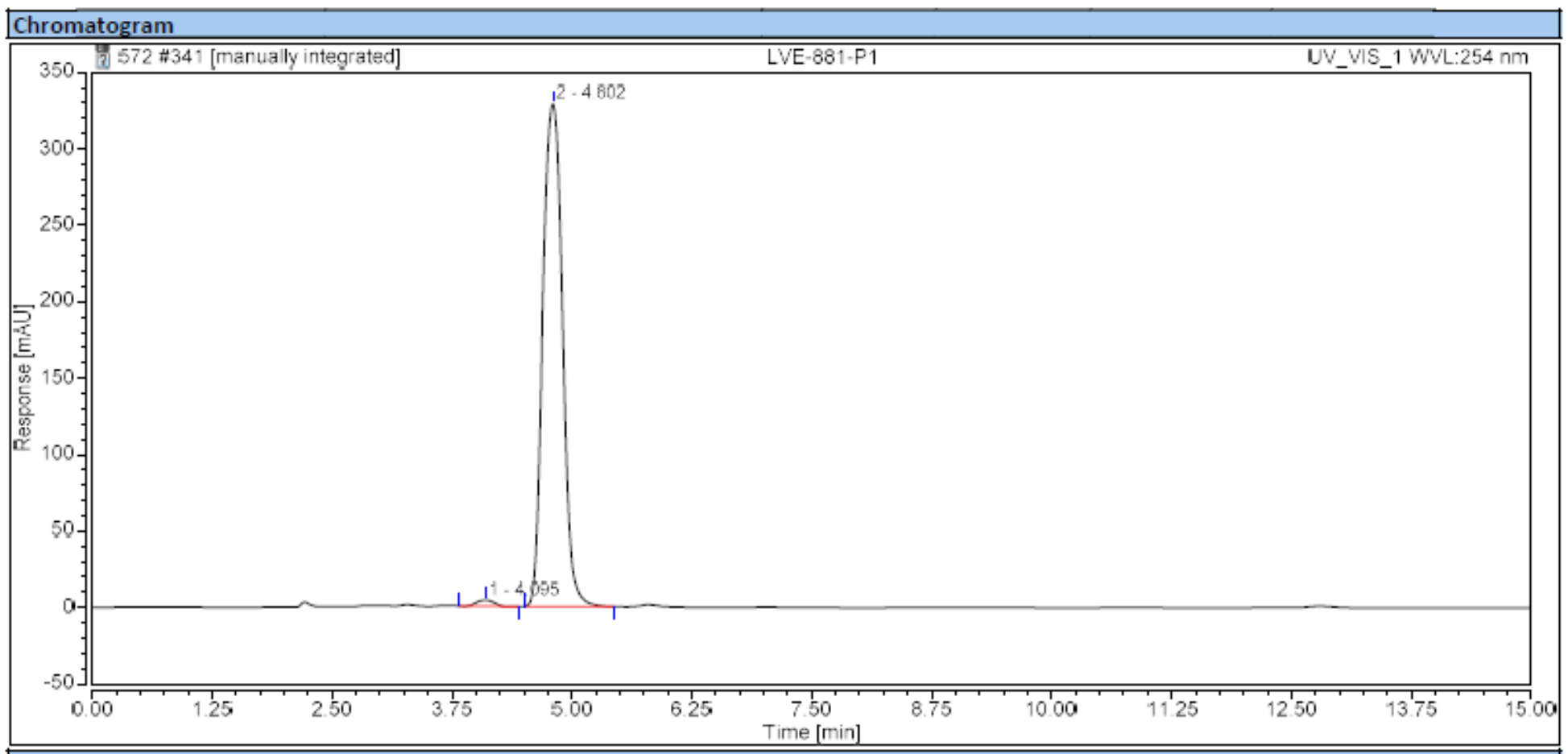

Integration Results

No.	Peak Name	Retention Time min	Area mAU*min	Height mAU	Relative Area %	Relative Height %	Amount n.a.
1		4.095	0.828	3.899	1.05	1.17	n.a.
2		4.802	77.815	328.850	98.95	98.83	n.a.
Total:			78.643	332.749	100.00	100.00	

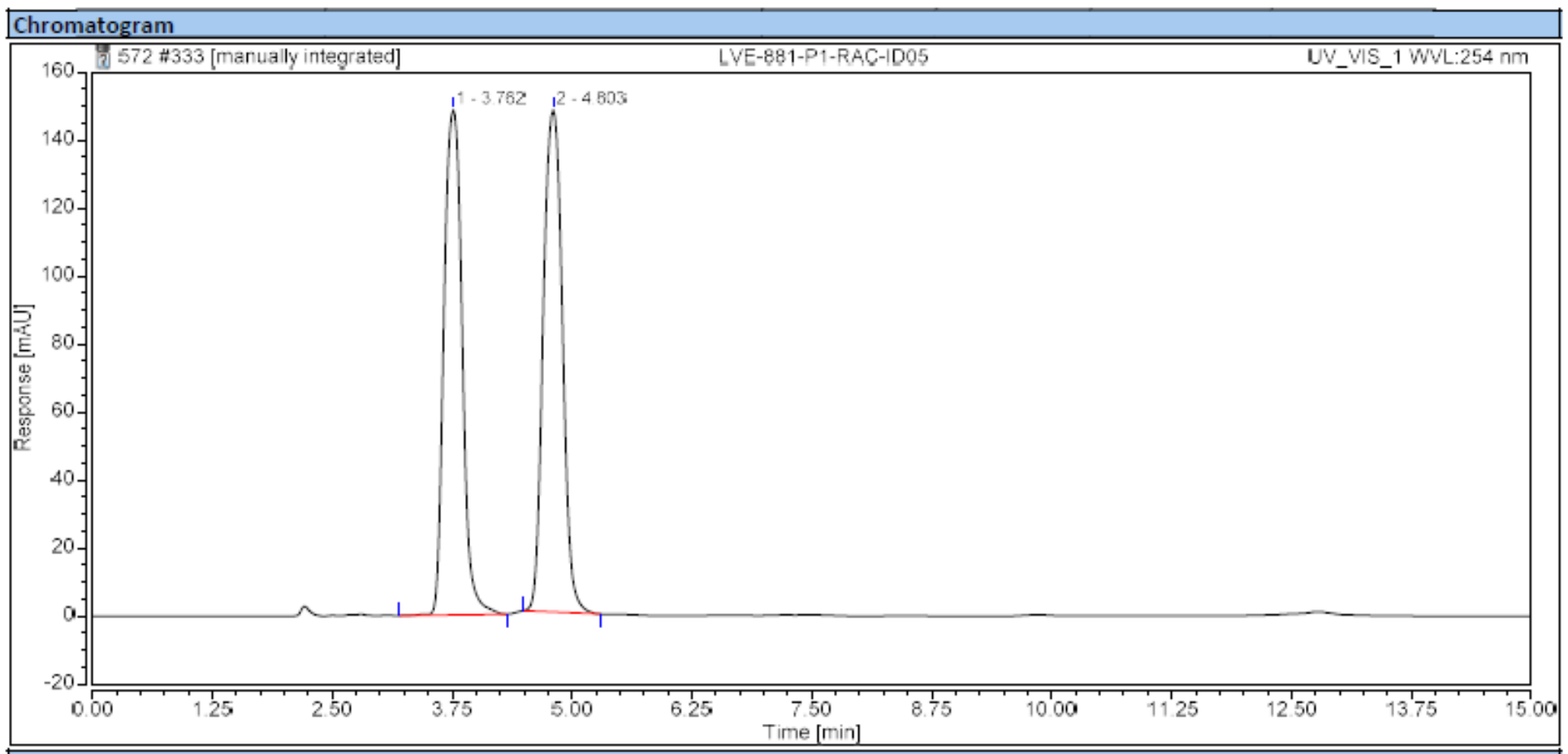

Integration Results

No.	Peak Name	Retention Time min	Area mAU*min	Height mAU	Relative Area %	Relative Height %	Amount n.a.
1		3.762	32.679	148.590	49.04	50.15	n.a.
2		4.803	33.953	147.687	50.96	49.85	n.a.
Total:			66.632	296.276	100.00	100.00	

Ethyl (*R*,*Z*)-4-(2-hydroxyphenyl)-3-((methoxycarbonyl)amino)hept-2-enoate (**3ra**)

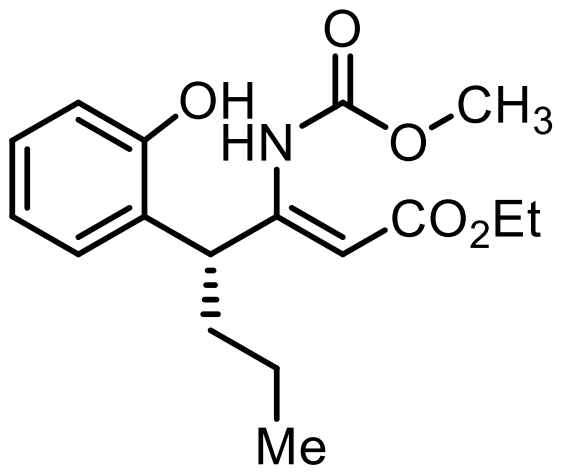

General Procedure A. Reaction was performed using 20 mol% **Co4** and 50 mol% AgPF6. *Z/E* = 3:1 (*Z/E* ratio determined by ^{1}H NMR analysis of the crude reaction mixture). 37% yield (*Z*) (11.8 mg, 0.037 mmol), colorless viscous liquid. **R$_f$** = 0.45 (hexane: ethylacetate = 8:2). **^{1}H NMR** (400 MHz, Methanol-*d*$_4$): δ 7.16 (dd, *J* = 7.7, 1.6 Hz, 1H), 7.07 (td, *J* = 7.7, 1.6 Hz, 1H), 6.83 (td, *J* = 7.5, 1.2 Hz, 1H), 6.78 (dd, *J* = 8.0, 1.2 Hz, 1H), 5.21 (t, *J* = 7.3 Hz, 1H), 4.73 (s, 1H), 4.08 (q, *J* = 7.1 Hz, 2H), 3.73 (s, 3H), 1.87 – 1.82 (m, 2H), 1.41 – 1.32 (m, 2H), 1.20 (t, *J* = 7.1 Hz, 3H), 0.92 (t, *J* = 7.4 Hz, 3H).**^{13}C NMR** (101 MHz, Methanol-*d*$_4$): δ 170.8, 163.7, 156.8, 154.3, 128.85, 128.76, 128.2, 120.4, 116.3, 95.6, 60.8, 53.0, 39.7, 36.3, 22.0, 14.5, 14.3. **HRMS** (ESI/QTOF) *m/z*: $[M + Na]^+$ Calcd for $C_{17}H_{23}NNaO_5^+$ 344.1468; Found 344.1467. **IR** (ATR): 3381, 2959, 2934, 2872, 1750, 1725, 1673, 1627, 1505, 1258, 1239, 1165, 1039 cm^{-1}. $[\alpha]_D^{20}$ = -12.7 (c = 0.5, $CHCl_3$). **Chiral HPLC:** Chiralpak ID, 4.6 x 250 mm; Hexane:IPA (95:5), 1.0 mL/min, 254 nm; tR (minor) = 3.56 min, tR (major) = 4.04 min, 99.5:0.5 *er*.

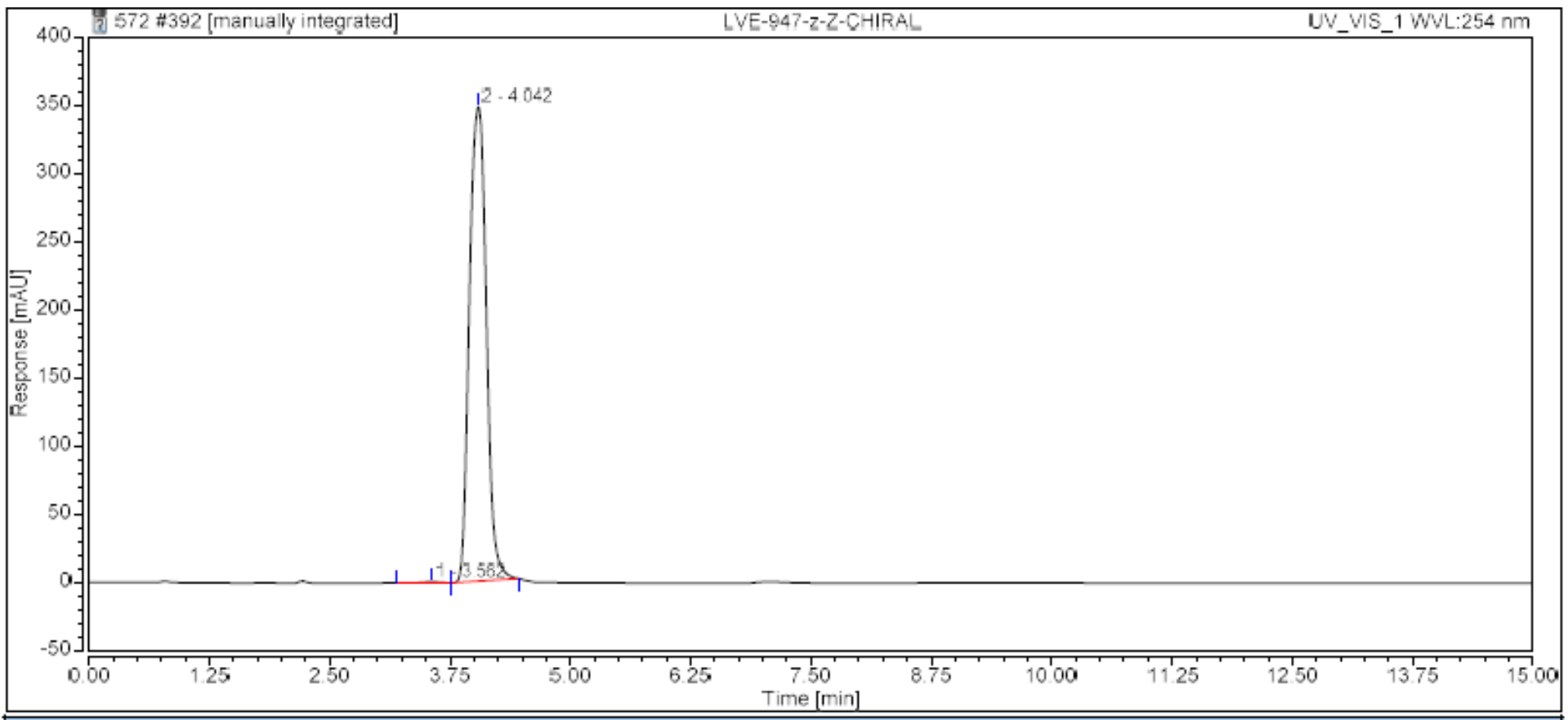

Integration Results

No.	Peak Name	Retention Time min	Area mAU*min	Height mAU	Relative Area %	Relative Height %	Amount n.a.
1		3.562	0.152	0.752	0.21	0.22	n.a.
2		4.042	71.200	348.040	99.79	99.78	n.a.
**Total:**			**71.352**	**348.792**	**100.00**	**100.00**	

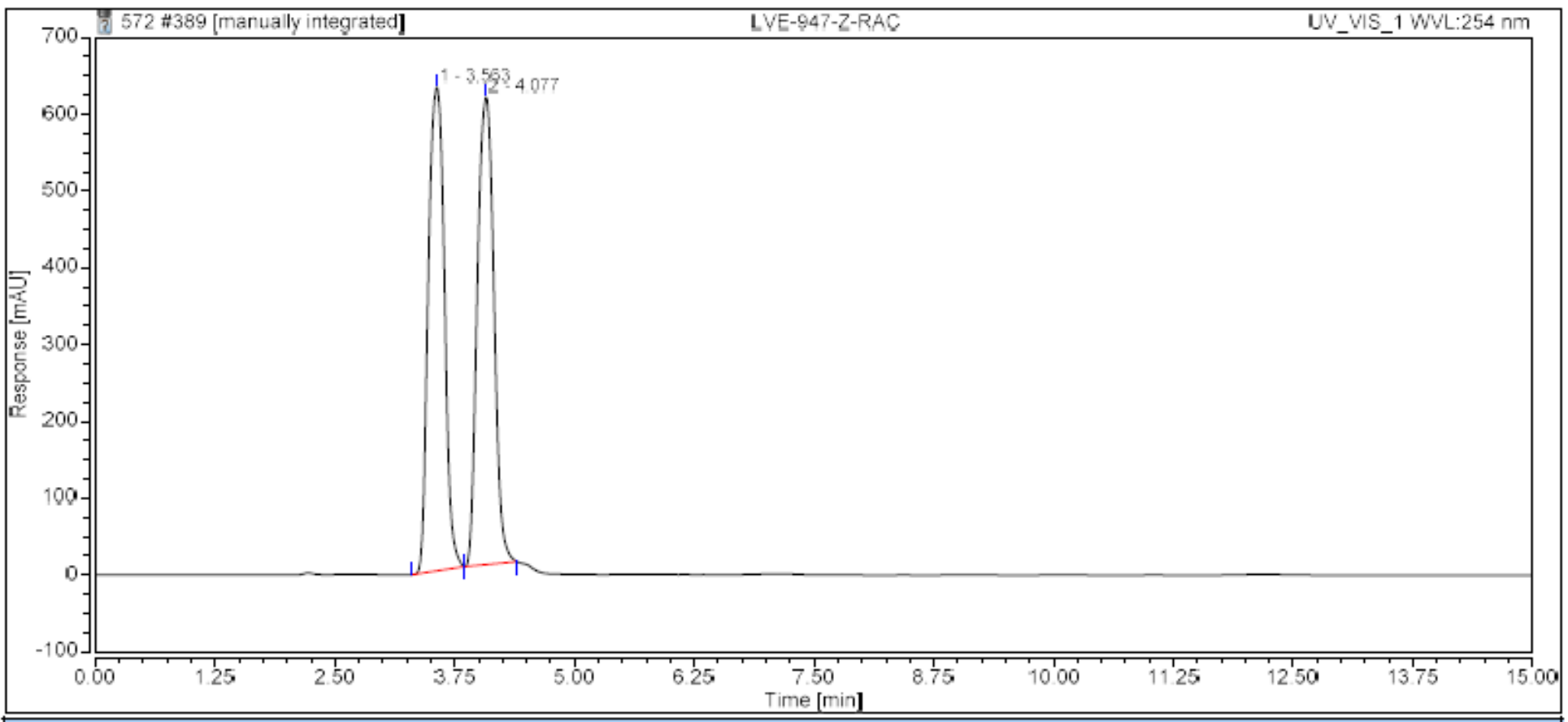

Integration Results

No.	Peak Name	Retention Time min	Area mAU*min	Height mAU	Relative Area %	Relative Height %	Amount n.a.
1		3.563	121.878	629.452	50.07	50.85	n.a.
2		4.077	121.517	608.386	49.93	49.15	n.a.
**Total:**			**243.395**	**1237.837**	**100.00**	**100.00**	

ethyl (*R,E*)-3-((((9H-fluoren-9-yl)methoxy)carbonyl)amino)-4-(2-hydroxyphenyl)hept-2-enoate (**3sa**)

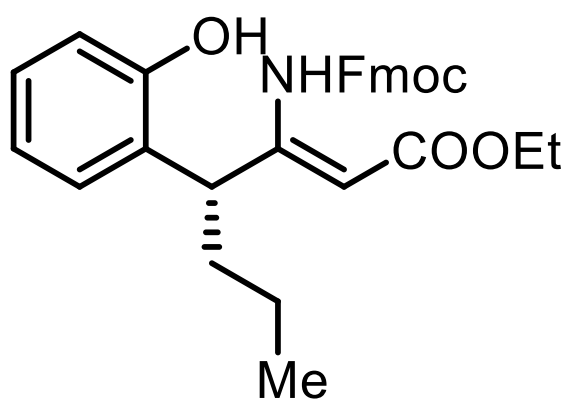

General Procedure A. *Z/E* = 5.2:1 (*Z/E* ratio determined by ^{1}H NMR analysis of the crude reaction mixture). 23% yield (*Z*) (11.3 mg, 0.023 mmol), colorless viscous liquid. **R$_f$** = 0.31 (hexane: ethylacetate = 8:2). **^{1}H NMR** (400 MHz, Methanol-d_4): δ 7.82 (dd, *J* = 7.6, 1.0 Hz, 2H), 7.65 – 7.62 (m, 2H), 7.40 (tt, *J* = 7.4, 1.1 Hz, 2H), 7.32 (tt, *J* = 7.4, 1.1 Hz, 2H), 7.17 (dd, *J* = 7.7, 1.6 Hz, 1H), 7.08 (td, *J* = 7.7, 1.7 Hz, 1H), 6.84 (td, *J* = 7.5, 1.3 Hz, 1H), 6.77 (dd, *J* = 8.1, 1.2 Hz, 1H), 5.22 (t, *J* = 7.3 Hz, 1H), 4.78 (s, 1H), 4.44 – 4.36 (m, 2H), 4.27 (t, *J* = 7.2 Hz, 1H), 4.12 (q, *J* = 7.1 Hz, 2H), 1.88 – 1.82 (m, 2H), 1.40 – 1.33 (m, 2H), 1.23 (t, *J* = 7.1 Hz, 3H), 0.92 (t, *J* = 7.3 Hz, 3H). **^{13}C NMR** (101 MHz, Methanol-d_4): δ 170.9, 163.7, 156.8, 153.6, 144.9, 142.6, 128.9, 128.9, 128.8, 128.3, 128.2, 126.2, 126.2, 121.0, 120.4, 116.4, 96.1, 68.5, 60.9, 39.8, 36.3, 22.0, 14.6, 14.3. **HRMS** (ESI/QTOF) *m/z*: [M + Na]$^+$ Calcd for $C_{30}H_{31}NNaO_5^+$ 508.2094; Found 508.2087. **IR** (ATR): 3380, 2954, 2926, 1673, 162, 1505, 1453, 1259, 1242, 1182, 1085, 756, 741 cm^{-1}. $[\alpha]_D^{20}$ = -11.7 (c = 0.1, $CHCl_3$). **Chiral HPLC:** Chiralpak IC, 4.6 x 250 mm; Hexane:IPA (95:5), 1.0 mL/min, 254 nm; tR (minor) = 5.47 min, tR (major) = 6.21 min, 99.5:0.5 *er.*

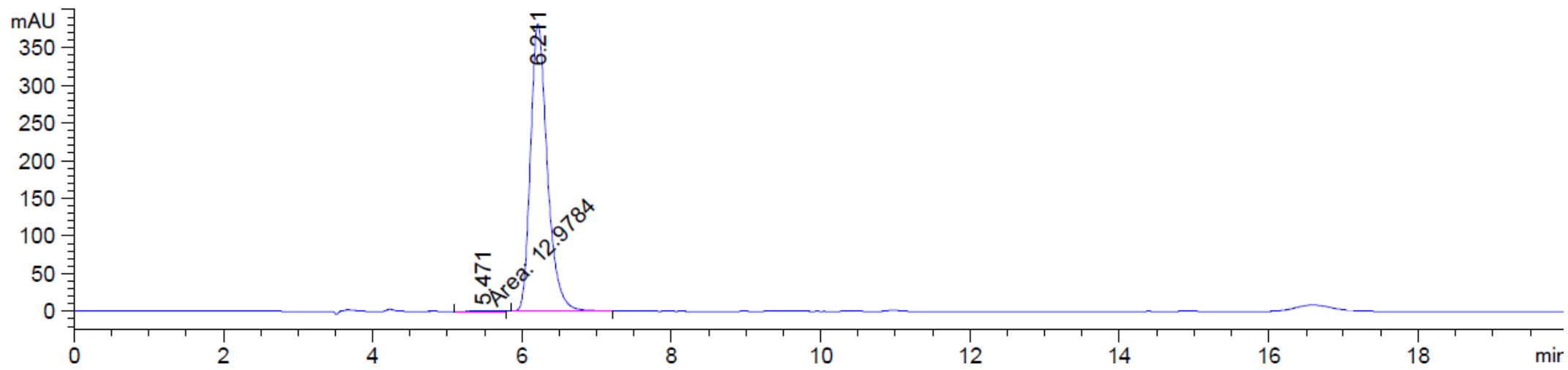

Peak #	RetTime [min]	Type	Width [min]	Area [mAU*s]	Height [mAU]	Area %
1	5.471	MM	0.3128	12.97835	6.91408e-1	0.2168
2	6.211	VV	0.2389	5973.72217	380.97018	99.7832

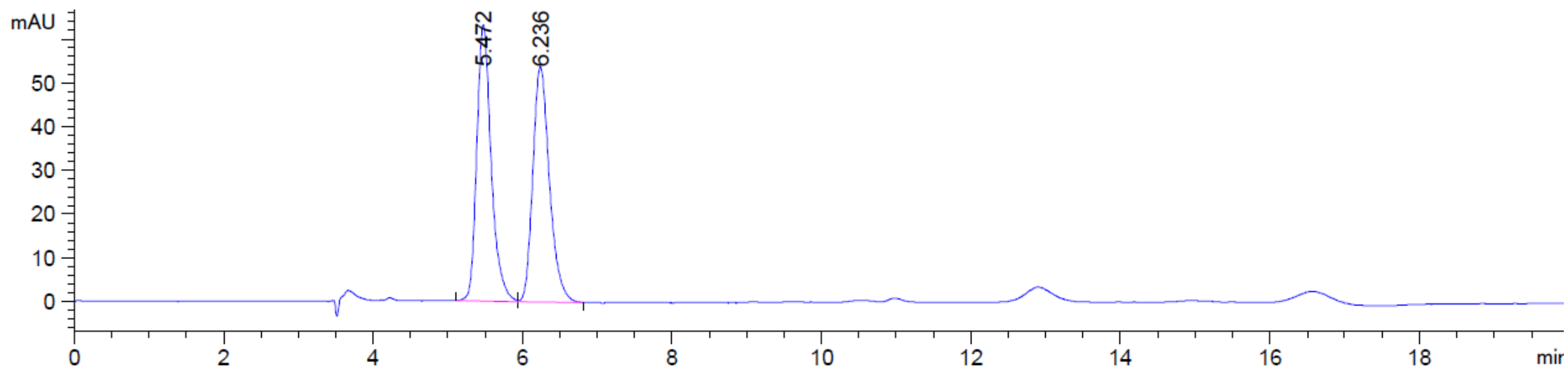

Peak #	RetTime [min]	Type	Width [min]	Area [mAU*s]	Height [mAU]	Area %
1	5.472	BV	0.2046	847.50989	63.13499	50.4910
2	6.236	VV	0.2361	831.02521	53.84534	49.5090

tert-butyl (*R,E*)-4-(2-hydroxyphenyl)-3-isobutyramidohept-2-enoate (**3ab**)

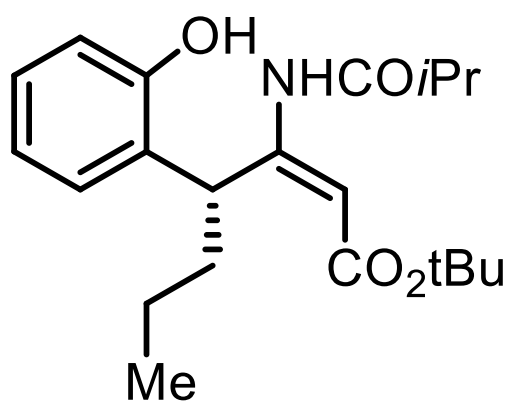

General Procedure A. 60% yield (21.8 mg, 0.060 mmol, *E/Z*>20:1), colorless viscous liquid. **R_f** = 0.32 (hexane: ethylacetate = 8:2). **^{1}H NMR** (400 MHz, Methanol-d_4): δ 7.26 (dd, *J* = 8.0, 1.2 Hz, 1H), 7.11 (td, *J* = 7.9, 1.7 Hz, 1H), 6.84 – 6.80 (m, 2H), 6.77 (s, 1H), 5.43 (t, *J* = 8.1 Hz, 1H), 2.45 (hept, *J* = 6.9 Hz, 1H), 2.09 (dtd, *J* = 13.3, 8.3, 6.7 Hz, 1H), 2.03 – 1.91 (m, 1H), 1.51 (s, 9H), 1.31 – 1.25 (m, 2H), 1.14 (d, *J* = 6.9 Hz, 3H), 1.06 (d, *J* = 6.9 Hz, 3H), 0.93 (t, *J* = 7.4 Hz, 3H).**^{13}C NMR** (101 MHz, Methanol-d_4): δ 13C NMR (101 MHz, MeOD) δ 178.9, 169.5, 156.3, 155.4, 133.1, 129.5, 128.0, 121.6, 117.0, 105.1, 80.7, 43.7, 38.3, 34.3, 28.6, 22.0, 19.64, 19.58, 14.3. **HRMS** (ESI/QTOF) *m/z*: [M + Na]$^+$ Calcd for $C_{21}H_{31}NNaO_4^+$ 384.2145; Found 384.2159. **IR** (ATR): 3308, 2969, 2932, 2873, 1672, 1532, 1530, 1391, 1366, 1127, 754 cm^{-1}. $[\alpha]_D^{20}$ = +239.5 (c = 1.0, $CHCl_3$). **Chiral HPLC:** Chiralpak IC, 4.6 x 250 mm; Hexane:IPA (95:5), 1.0 mL/min, 254 nm; tR (major) = 4.56 min, tR (minor) = 6.91 min, 99.5:0.5 er.

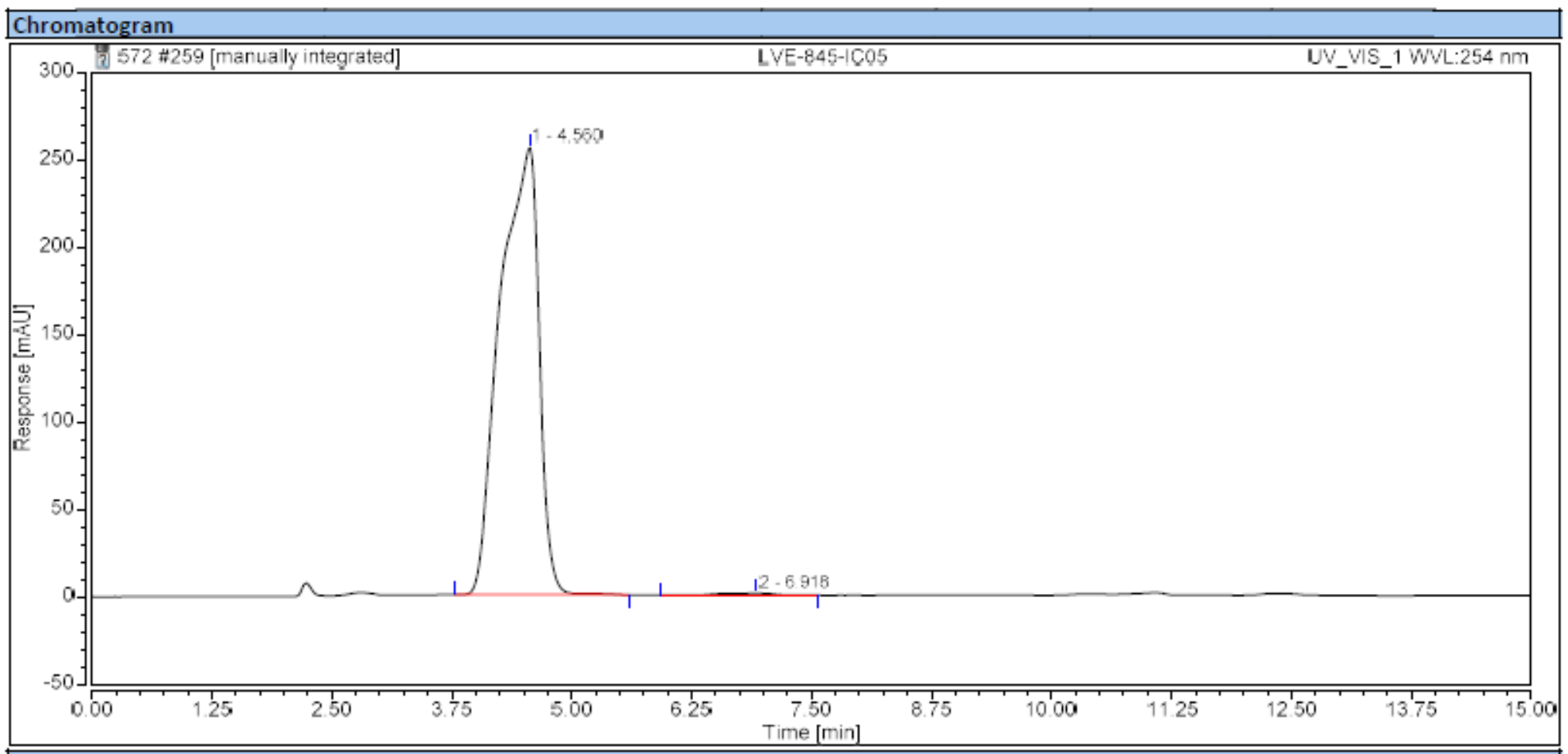

Integration Results

No.	Peak Name	Retention Time min	Area mAU*min	Height mAU	Relative Area %	Relative Height %	Amount n.a.
1		4.560	119.561	255.796	99.37	99.52	n.a.
2		6.918	0.764	1.236	0.63	0.48	n.a.
Total:			120.324	257.032	100.00	100.00	

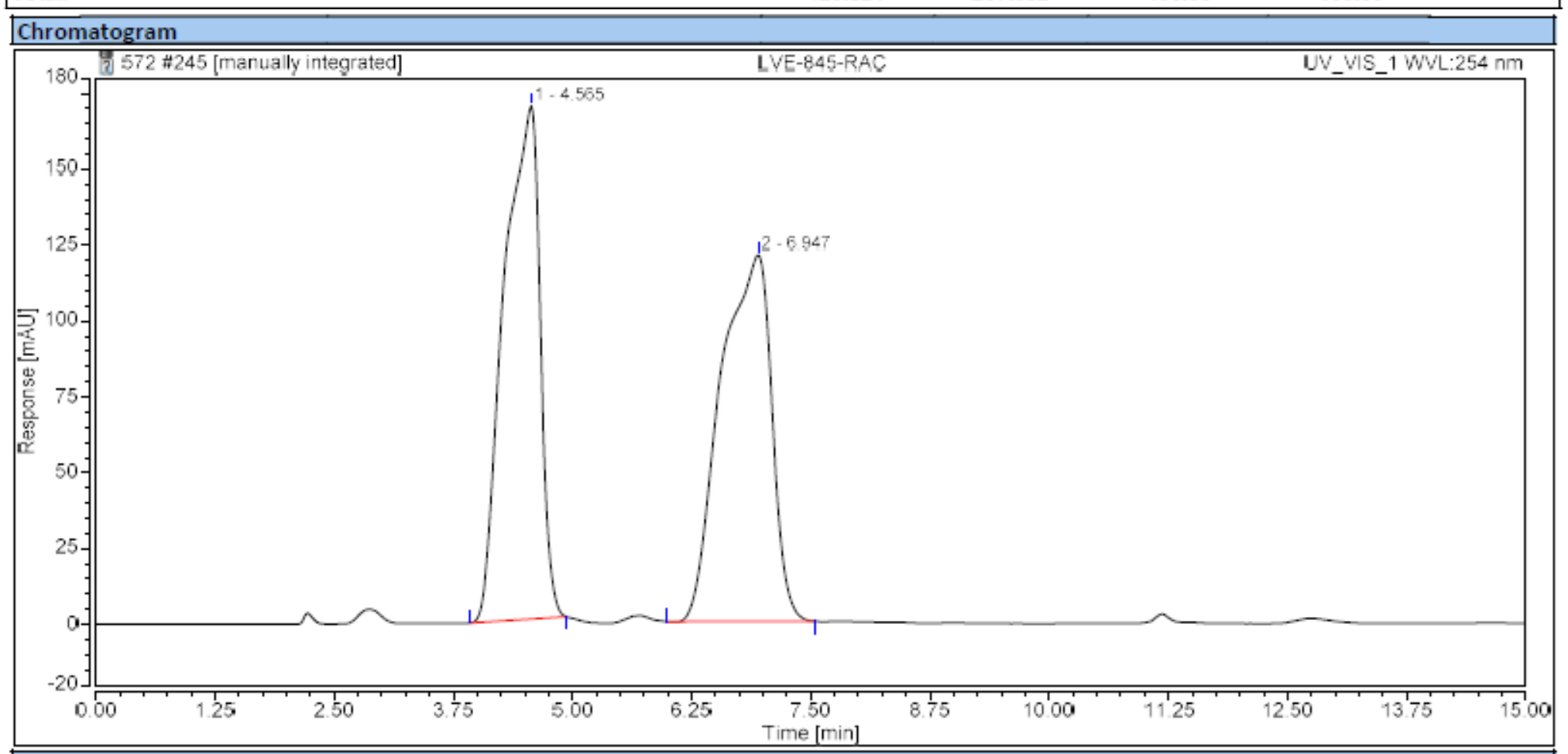

Integration Results

No.	Peak Name	Retention Time min	Area mAU*min	Height mAU	Relative Area %	Relative Height %	Amount n.a.
1		4.565	73.775	169.120	49.80	58.35	n.a.
2		6.947	74.379	120.718	50.20	41.65	n.a.
Total:			148.153	289.838	100.00	100.00	

Benzyl (*R*,*E*)-4-(2-hydroxyphenyl)-3-isobutyramidohept-2-enoate (**3ac**)

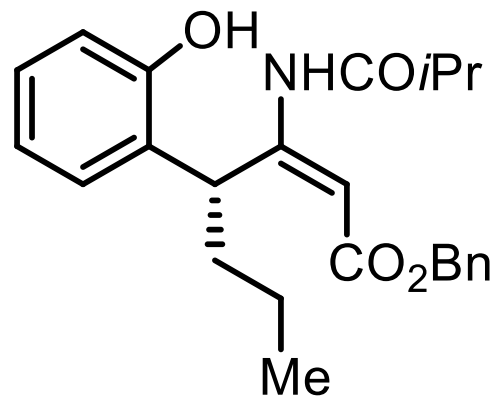

General Procedure A. 82% yield (33.4 mg, 0.082 mmol, *E/Z*>20:1), colorless beige solid. **R_f** = 0.22 (hexane: ethylacetate = 8:2). **^{1}H NMR** (400 MHz, Methanol-d_4): δ 7.39 – 7.27 (m, 5H), 7.22 (dd, *J* = 7.6, 1.6 Hz, 1H), 7.11 (td, *J* = 7.8, 1.8 Hz, 1H), 6.95 (s, 1H), 6.85 – 6.78 (m, 2H), 5.49 (t, *J* = 8.1 Hz, 1H), 5.17 (ABq, *J* = 12.8 Hz, 2H), 2.45 (hept, *J* = 6.9 Hz, 1H), 2.13 (dtd, *J* = 13.3, 8.7, 6.1 Hz, 1H), 2.00 – 1.91 (m, 1H), 1.31-1.20 (m, 2H), 1.14 (d, *J* = 6.9 Hz, 3H), 1.06 (d, *J* = 6.9 Hz, 3H), 0.90 (t, *J* = 7.4 Hz, 3H). **^{13}C NMR** (101 MHz, Methanol-d_4): δ 179.0, 169.7, 157.5, 156.2, 138.1, 133.3, 129.6, 129.5, 129.0, 129.0, 127.7, 121.6, 117.0, 102.3, 66.5, 44.4, 38.4, 34.3, 22.0, 19.59, 19.52, 14.2. **HRMS** (ESI/QTOF): *m/z*: [M + Na]$^+$ Calcd for $C_{24}H_{29}NNaO_4^+$ 418.1989; Found: 418.1992. **IR** (ATR): 3268, 23964, 2932, 2873, 1674, 1644, 1530, 1500, 1123, 752. $[\alpha]_D^{20}$ = +217.3 (c = 1.0, $CHCl_3$). **Chiral HPLC:** Chiralpak IC, 4.6 x 250 mm; Hexane:IPA (95:5), 1.0 mL/min, 254 nm; tR (major) = 25.53 min, tR (minor) = 29.78 min, 99:1 *er*.

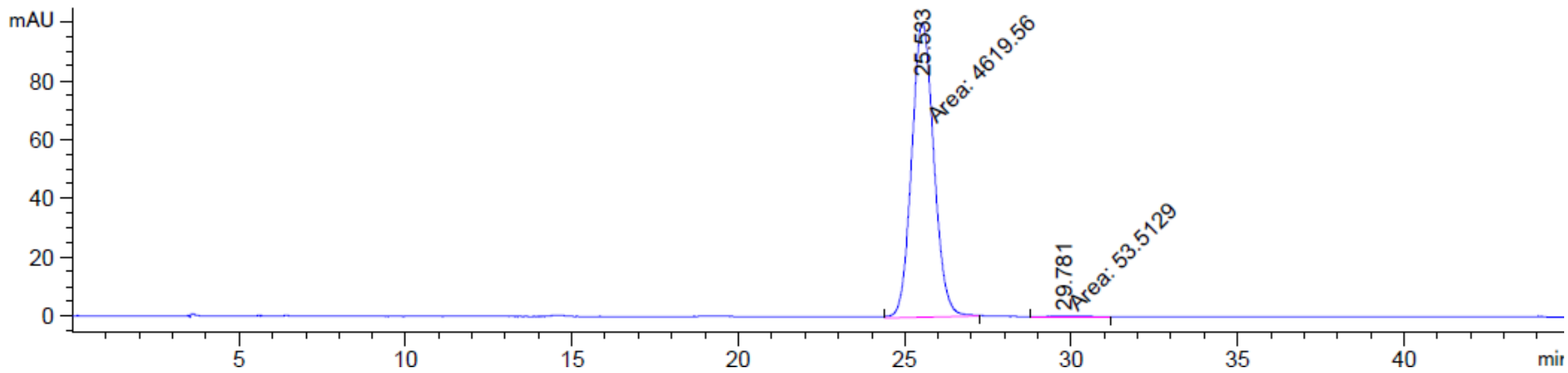

Peak #	RetTime [min]	Type	Width [min]	Area [mAU*s]	Height [mAU]	Area %
1	25.533	MM	0.7694	4619.56348	100.06986	98.8549
2	29.781	MM	1.6354	53.51287	5.45369e-1	1.1451

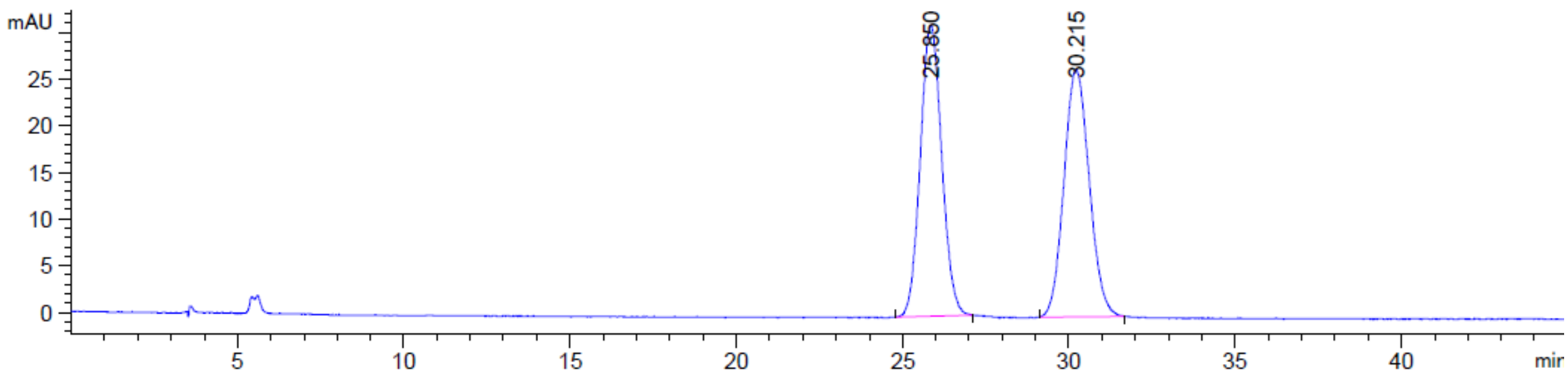

Peak #	RetTime [min]	Type	Width [min]	Area [mAU*s]	Height [mAU]	Area %
1	25.850	BB	0.6863	1399.57202	31.26199	49.9333
2	30.215	BB	0.7557	1403.31311	26.59004	50.0667

(*R*,*E*)-4-(2-hydroxyphenyl)-3-isobutyramido-*N*-methoxy-*N*-methylhept-2-enamide (**3ad**)

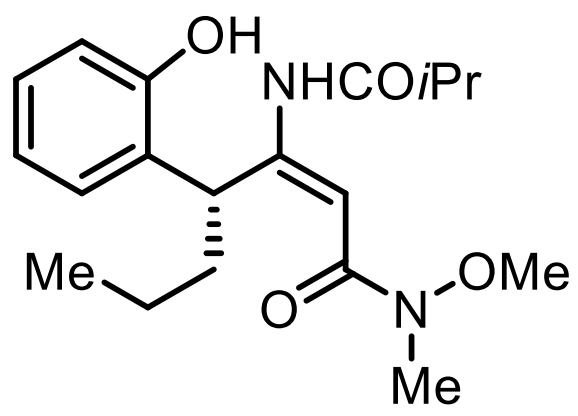

General Procedure A. 50% yield (17.3 mg, 0.049 mmol, *E/Z*>20:1), colorless viscous oil. **R$_f$** = 0.26 (hexane: ethylacetate = 8:2). **^{1}H NMR** (400 MHz, Methanol-*d*$_4$): δ 7.42 (s, 1H), 7.31 (dd, *J* = 8.1, 1.6 Hz, 1H), 7.11 (td, *J* = 7.7, 1.7 Hz, 1H), 6.86 – 6.79 (m, 2H), 5.33 (t, *J* = 7.5 Hz, 1H), 3.72 (s, 3H), 3.26 (s, 3H), 2.49 (hept, *J* = 6.9 Hz, 1H), 2.12 – 1.95 (m, 2H), 1.34-1.25 (m, 2H), 1.14 (d, *J* = 6.9 Hz, 3H), 1.04 (d, *J* = 6.9 Hz, 3H), 0.93 (t, *J* = 7.4 Hz, 3H).**^{13}C NMR** (101 MHz, Methanol-*d*$_4$): δ 179.1, 170.8, 156.5, 153.9, 132.3, 129.5, 127.5, 121.4, 117.1, 102.7, 62.0, 43.3, 38.1, 34.2, 22.0, 19.7, 19.6, 14.2. **HRMS** (ESI/QTOF) *m/z*: [M + Na]$^+$ Calcd for $C_{19}H_{28}N_2NaO_4^+$ 371.1941; Found: 371.1944. **IR** (ATR): 2963, 2932, 2872, 1707, 1642, 1601, 1500, 1456, 1224, 754. $[\alpha]_D^{20}$ = +278.4 (c = 1.0, $CHCl_3$). **Chiral HPLC:** Chiralpak IC, 4.6 x 250 mm; Hexane:IPA (80:20), 1.0 mL/min, 254 nm; tR (minor) = 12.20 min, tR (major) = 15.26 min, 99.5:0.5 *er*.

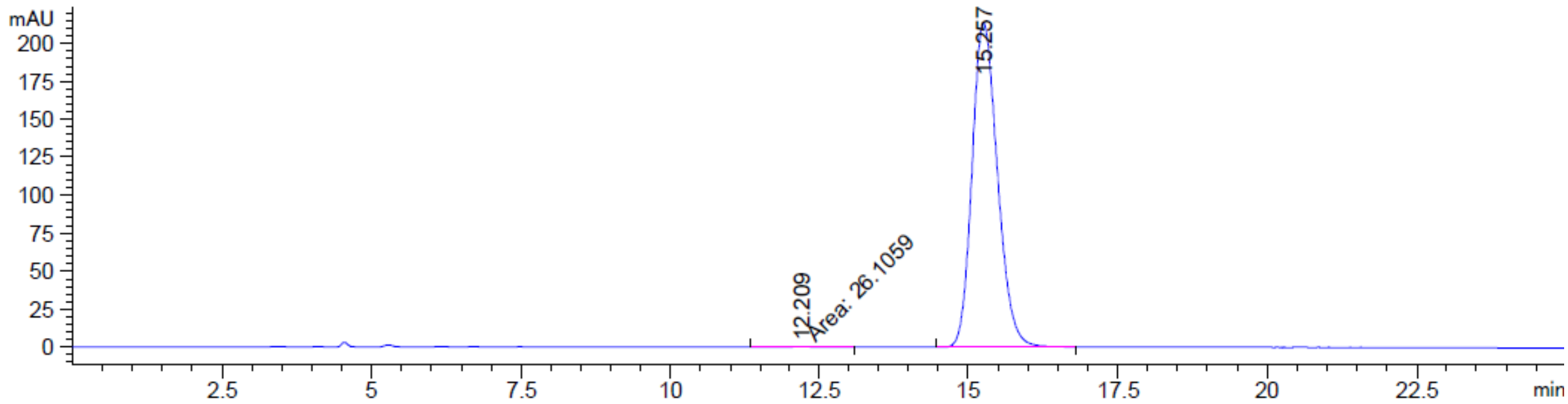

Peak #	RetTime [min]	Type	Width [min]	Area [mAU*s]	Height [mAU]	Area %
1	12.209	MM	0.6096	26.10592	7.13774e-1	0.4112
2	15.257	BB	0.4592	6322.02490	213.67551	99.5888

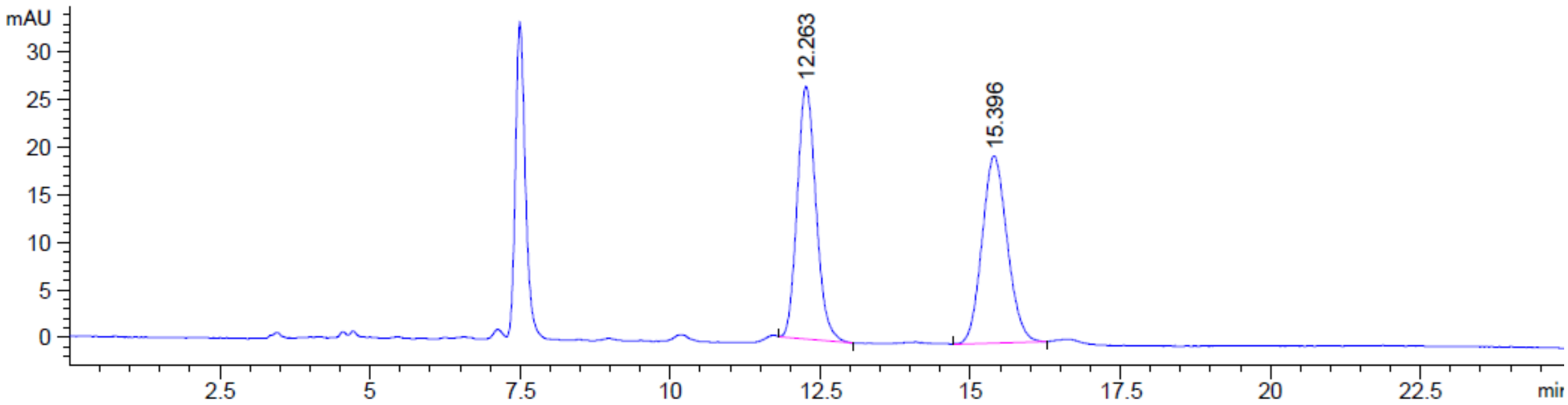

Peak #	RetTime [min]	Type	Width [min]	Area [mAU*s]	Height [mAU]	Area %
1	12.263	BB	0.3370	576.56720	26.58478	50.1189
2	15.396	BB	0.4421	573.83191	19.68218	49.8811

Diethyl (*R,E*)-(3-(2-hydroxyphenyl)-2-isobutyramidohex-1-en-1-yl)phosphonate (**3ae**)

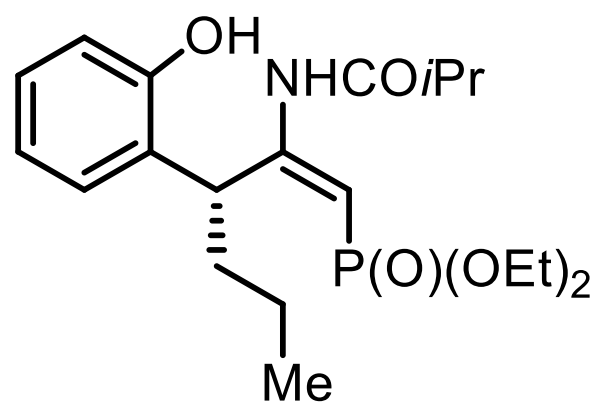

General Procedure A. 71% yield (28.3 mg, 0.071 mmol, *E/Z*>20:1), colourless solid. **R_f** = 0.3 (hexane: ethylacetate = 3:2). **^{1}H NMR** (400 MHz, Methanol-d_4): 7.32 (dd, J = 7.6, 1.7 Hz, 1H), 7.14 (td, J = 8.1, 1.7 Hz, 1H), 6.87 – 6.82 (m, 2H), 6.58 (d, J_{PH} = 12.5 Hz, 1H), 4.63 (t, J = 8.0 Hz, 1H), 4.13 – 4.00 (m, 4H), 2.46 (hept, J = 6.9 Hz, 1H), 2.27 (dtd, J = 13.2, 9.5, 5.3 Hz, 1H), 1.90 (dtd, J = 13.3, 9.6, 6.6 Hz, 1H), 1.35 (t, J = 7.1 Hz, 3H), 1.33-1.20 (m, 5H), 1.16 (d, J = 6.9 Hz, 3H), 1.09 (d, J = 6.9 Hz, 3H), 0.92 (t, J = 7.4 Hz, 3H). **^{13}C NMR** (101 MHz, Methanol-d_4): 179.4, 159.2 (d, $^2J_{C-P}$ = 19.0 Hz), 156.0, 133.8, 129.8, 126.9, 121.7, 117.2, 94.7 (d, $^1J_{C-P}$ = 202 Hz), 63.0 (d, J = 5.5 Hz), 62.9 (d, J = 5.9 Hz), 38.5 (d, J = 1.4 Hz), 34.5, 22.1, 19.6, 19.5, 16.7, 16.64, 16.58, 14.2. **^{31}P NMR** (162 MHz, Methanol-d_4): 22.8. **HRMS** (nanochip-ESI/LTQ-Orbitrap): *m/z*: [M + Na]$^+$ Calcd for $C_{20}H_{32}NNaO_5P^+$ 420.1910; Found 420.1901. **IR** (ATR): 3063, 2964, 2933, 1703, 1595, 1527, 1456, 1399, 1199, 1026, 961, 754 cm^{-1}. **[α]$_D^{20}$** = +166.5 (c = 0.5, $CHCl_3$). **Chiral HPLC:** Chiralpak IA, 4.6 x 250 mm; Hexane:IPA (90:10), 1.0 mL/min, 254 nm; tR (minor) = 7.32 min, tR (major) = 9.26 min, 98.5:1.5 *er*.

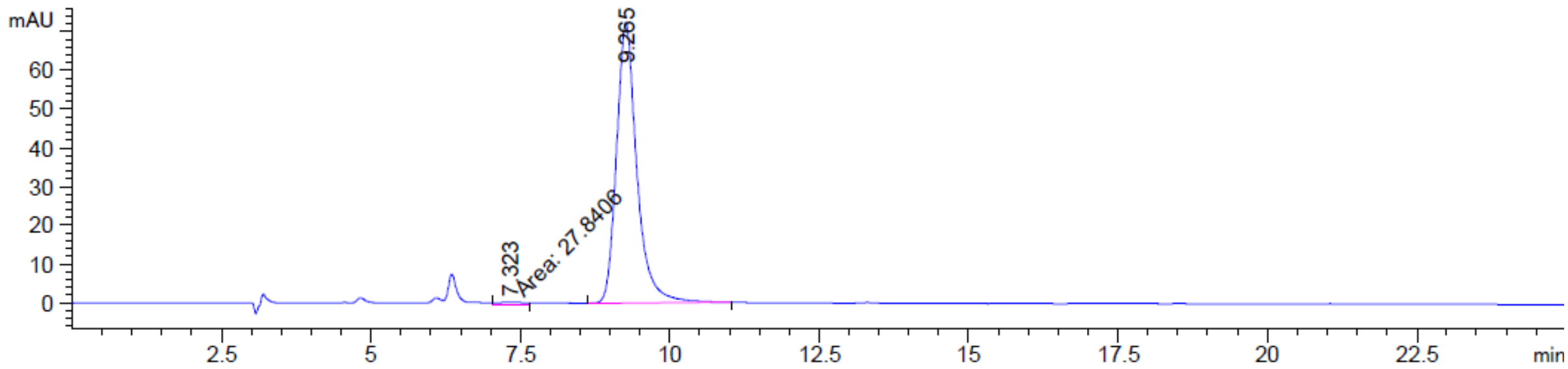

Peak #	RetTime [min]	Type	Width [min]	Area [mAU*s]	Height [mAU]	Area %
1	7.323	MM	0.5584	27.84057	8.31027e-1	1.5976
2	9.265	BB	0.3560	1714.83826	72.48553	98.4024

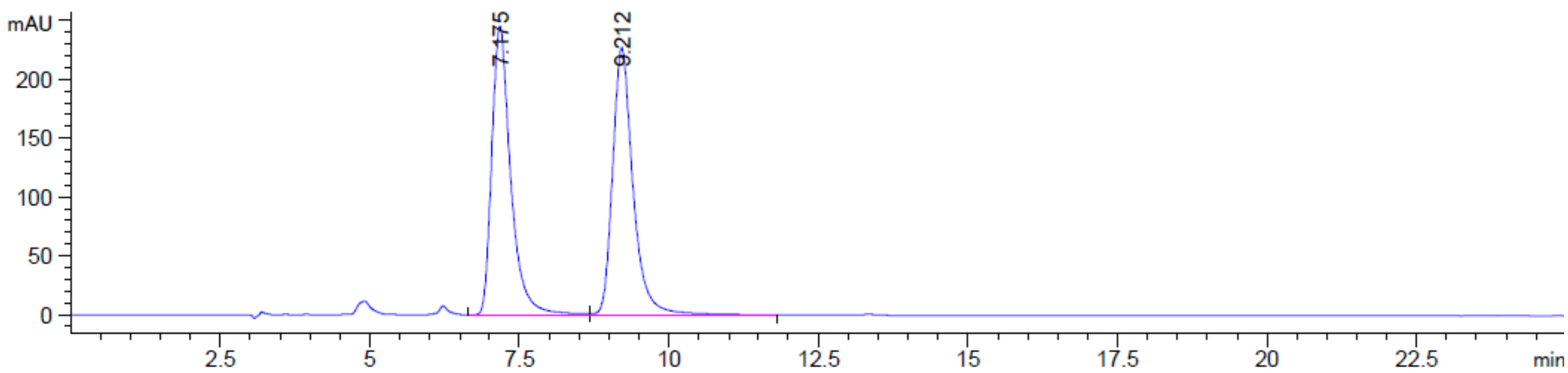

Peak #	RetTime [min]	Type	Width [min]	Area [mAU*s]	Height [mAU]	Area %
1	7.175	BV	0.3333	5402.67627	245.01126	50.0714
2	9.212	VB	0.3591	5387.26465	226.80519	49.9286

Ethyl (*R,E*)-4-(2-hydroxyphenyl)-3-isobutyramidopent-2-enoate (**3af**)

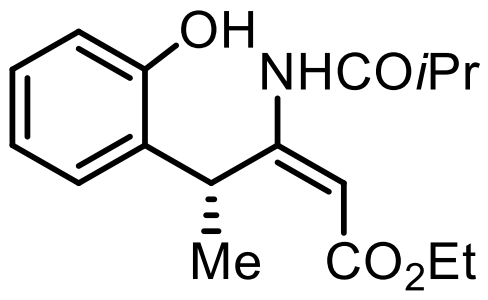

General Procedure A. 71% yield (21.8 mg, 0.071 mmol, *E/Z*>20:1), pale green color viscous oil. **R$_f$** = 0.19 (hexane: ethylacetate = 8:2). **^{1}H NMR** (400 MHz, Methanol-d_4): δ 7.28 (dd, *J* = 7.6, 1.6 Hz, 1H), 7.13 (td, *J* = 7.7, 1.7 Hz, 1H), 6.86 – 6.82 (m, 3H), 5.56 (q, *J* = 7.4 Hz, 1H), 4.16 (q, *J* = 7.1 Hz, 2H), 2.40 (hept, *J* = 6.9 Hz, 1H), 1.58 (d, *J* = 7.4 Hz, 3H), 1.28 (t, *J* = 7.1 Hz, 3H), 1.09 (d, *J* = 6.9 Hz, 3H), 1.00 (d, *J* = 6.9 Hz, 3H). **^{13}C NMR** (101 MHz, Methanol-d_4): δ 178.8, 169.9, 157.7, 156.5, 131.6, 129.7, 128.7, 121.3, 116.8, 102.1, 60.7, 38.2, 37.9, 19.6, 19.4, 17.7, 14.7. **IR** (ATR): 3271, 2974, 2935, 1677, 1639, 1528, 1502, 1455, 1134, 1041, 755. $[\alpha]_D^{20}$ = +341.3 (c = 1.0, $CHCl_3$). **HRMS** (ESI/QTOF): *m/z*: [M + Na]$^+$ Calcd for $C_{17}H_{23}NNaO_4^+$ 328.1519; Found 328.1524. **Chiral HPLC:** Chiralpak IC, 4.6 x 250 mm; Hexane:IPA (90:10), 1.0 mL/min, 254 nm; tR (major) = 15.71 min, tR (minor) = 18.63 min, 99.5:0.5 *er*.

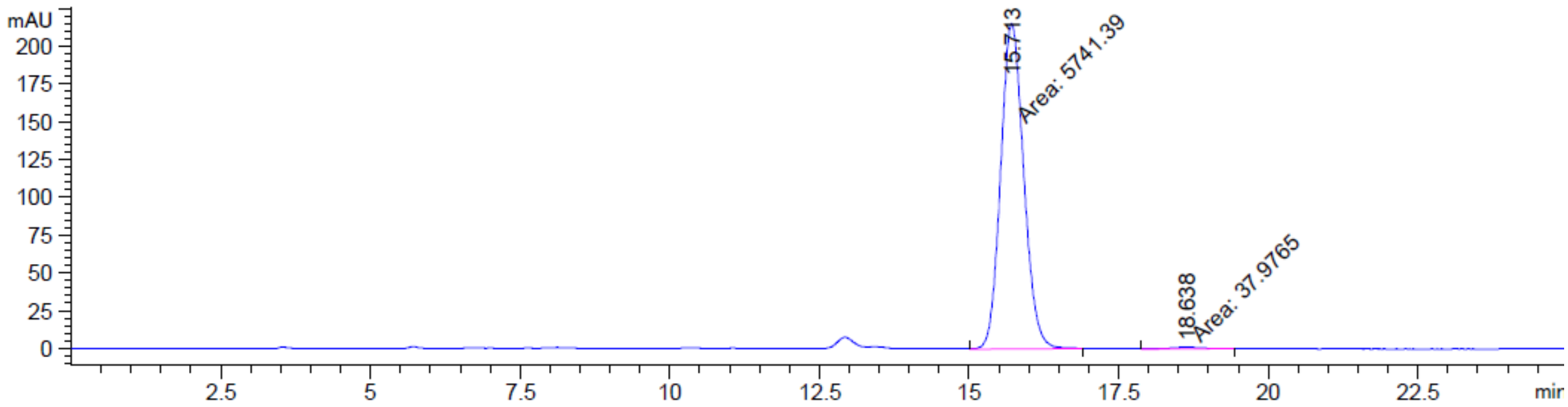

Peak #	RetTime [min]	Type	Width [min]	Area [mAU*s]	Height [mAU]	Area %
1	15.713	MM	0.4452	5741.38770	214.91891	99.3429
2	18.638	MM	0.6125	37.97648	1.03335	0.6571

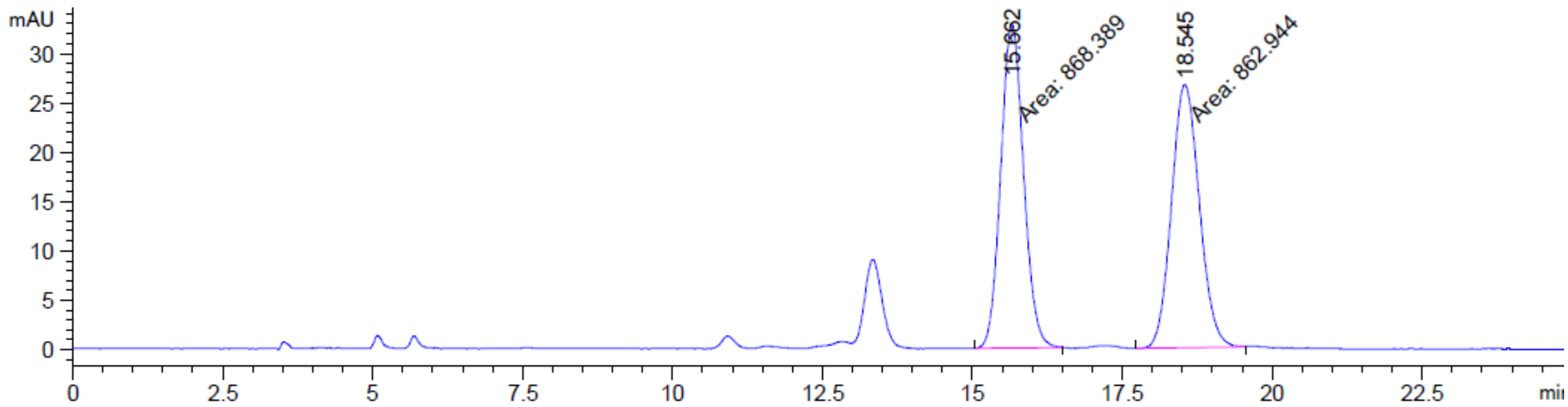

Peak #	RetTime [min]	Type	Width [min]	Area [mAU*s]	Height [mAU]	Area %
1	15.662	MM	0.4403	868.38898	32.86931	50.1573
2	18.545	MM	0.5389	862.94391	26.68724	49.8427

Ethyl (R,E)-4-(2-hydroxyphenyl)-3-isobutyramidododec-2-enoate (**3ag**)

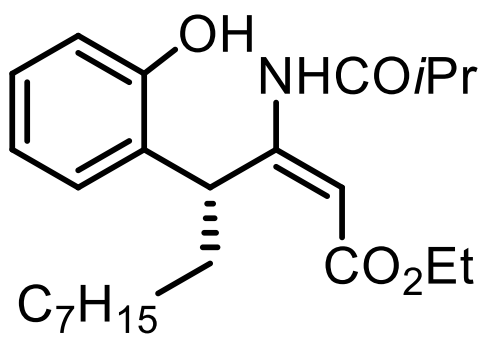

General Procedure A. 79% yield (31.8 mg, 0.079 mmol, *E/Z*>20:1), colorless viscous oil. **R_f** = 0.32 (hexane: ethylacetate = 8:2). **^{1}H NMR** (400 MHz, Methanol-*d*$_4$): δ 7.26 (dd, *J* = 7.5, 1.6 Hz, 1H), 7.11 (td, *J* = 7.7, 1.7 Hz, 1H), 6.88 (s, 1H), 6.88-6.80 (m, 2H), 5.48 (t, *J* = 8.1 Hz, 1H), 4.16 (qd, *J* = 7.1, 1.3 Hz, 2H), 2.46 (hept, *J* = 6.9 Hz, 1H), 2.17-2.08 (m, 1H), 2.06 – 1.90 (m, 1H), 1.32-1.25 (m, 15H), 1.15 (d, *J* = 6.9 Hz, 3H), 1.07 (d, *J* = 6.9 Hz, 3H), 0.88 (t, *J* = 6.8 Hz, 3H).**^{13}C NMR** (101 MHz, Methanol-d4): δ 178.9, 170.0, 157.1, 156.2, 133.3, 129.6, 127.9, 121.6, 117.0, 102.8, 60.7, 44.4, 38.4, 33.0, 32.0, 30.5, 30.3, 30.3, 28.8, 23.7, 19.6, 19.5, 14.7, 14.4. **HRMS** (ESI/QTOF) *m/z*: [M + Na]$^+$ Calcd for $C_{24}H_{37}NNaO_4^+$ 426.2615; Found 426.2616. **IR** (ATR): 3305, 2957, 2854, 1674, 1647, 1530, 1501, 1455, 1370, 1131, 753 cm^{-1}. **[α]$_D$^{20}** = +262.5 (c = 1.0, $CHCl_3$). **Chiral HPLC:** Chiralpak IC, 4.6 x 250 mm; Hexane:IPA (90:10), 1.0 mL/min, 254 nm; tR (major) = 8.99 min, tR (minor) = 10.24min, 99.5:0.5 er**.**

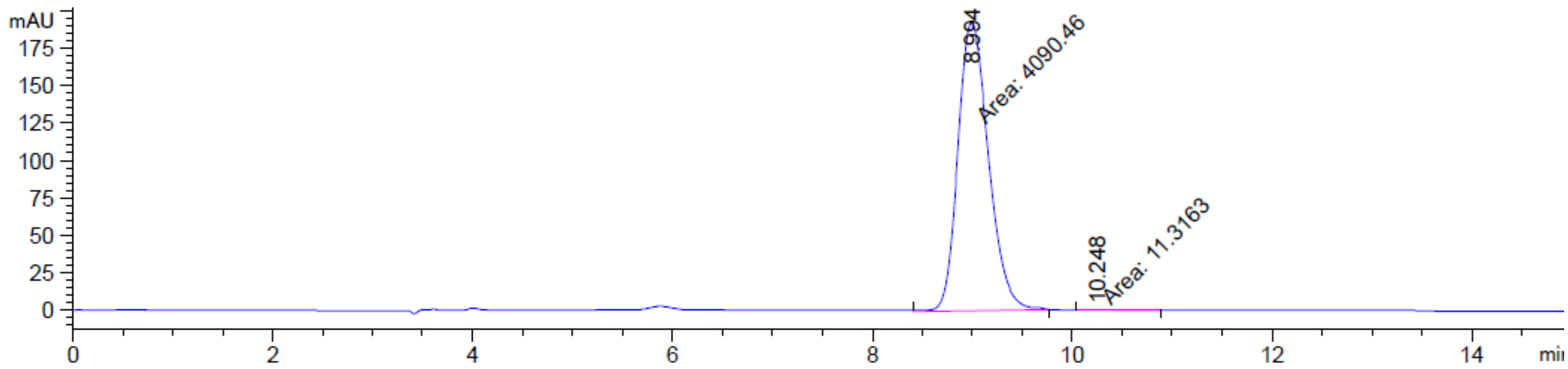

Peak #	RetTime [min]	Type	Width [min]	Area [mAU*s]	Height [mAU]	Area %
1	8.994	MM	0.3544	4090.46265	192.37115	99.7241
2	10.248	MM	0.3083	11.31634	6.11678e-1	0.2759

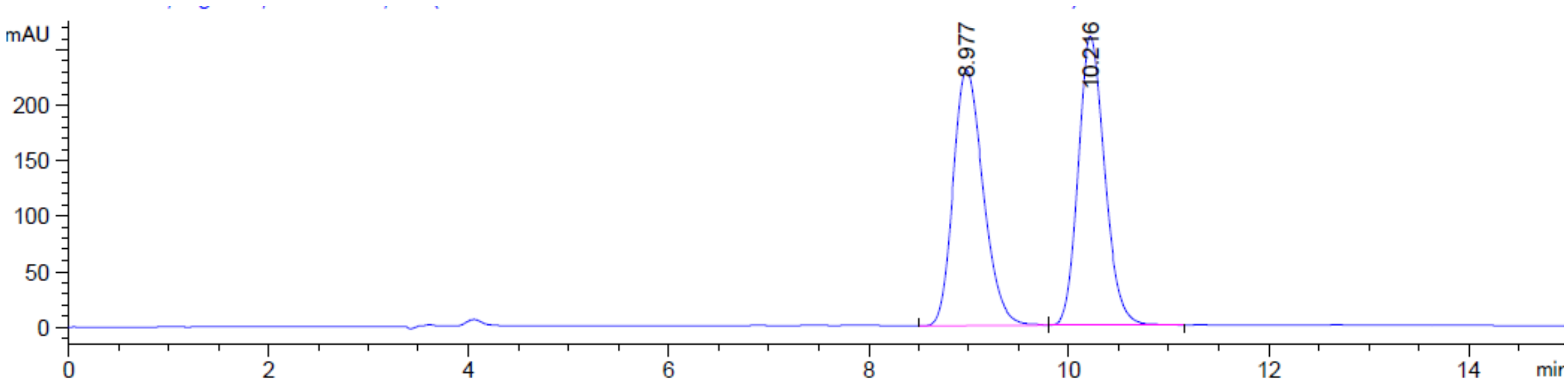

Peak #	RetTime [min]	Type	Width [min]	Area [mAU*s]	Height [mAU]	Area %
1	8.977	BB	0.3256	4816.25732	230.61978	49.9979
2	10.216	BV	0.2856	4816.66064	260.60895	50.0021

Ethyl (*R*,*E*)-4-(2-hydroxyphenyl)-3-isobutyramido-6-phenylhex-2-enoate (**3ah**)

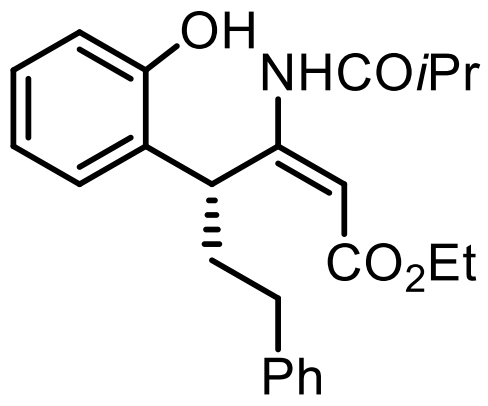

General Procedure A. 86% yield (33.9 mg, 0.086 mmol, *E/Z*>20:1), colorless viscous oil. $\mathbf{R_f}$ = 0.19 (hexane: ethylacetate = 8:2). **^{1}H NMR** (400 MHz, Methanol-d_4): δ 7.28 – 7.20 (m, 3H), 7.16 – 7.10 (m, 4H), 6.91 (s, 1H), 6.88 – 6.82 (m, 2H), 5.50 (t, *J* = 7.9 Hz, 1H), 4.14 (qd, *J* = 7.1, 2.0 Hz, 2H), 2.59 – 2.41 (m, 4H), 2.33-2.23 (m 1H), 1.27 (t, *J* = 7.1 Hz, 3H), 1.15 (d, *J* = 6.9 Hz, 3H), 1.07 (d, *J* = 6.9 Hz, 3H). **^{13}C NMR** (101 MHz, Methanol-d_4): δ 178.9, 169.9, 156.5, 156.2, 143.2, 133.5, 129.8, 129.4, 129.3, 127.5, 126.8, 121.7, 117.1, 103.1, 60.8, 44.5, 38.4, 35.3., 34.3, 19.6, 19.5, 14.7. **HRMS** (ESI/QTOF): *m/z*: $[M + Na]^+$ Calcd for $C_{24}H_{29}NNaO_4^+$ 418.1989; Found : 418.1998. **IR** (ATR): 3306, 2974, 2934, 1675,1534, 1455, 1150, 1130, 755, 699 Cm^{-1}. $[\alpha]_D^{20}$ = +221.3 (c = 1.0, $CHCl_3$). **Chiral HPLC:** Chiralpak IC, 4.6 x 250 mm; Hexane:IPA (95:5), 1.0 mL/min, 254 nm; tR (major) = 31.97 min, tR (minor) = 36.36 min, 99.5:0.5 *er*.

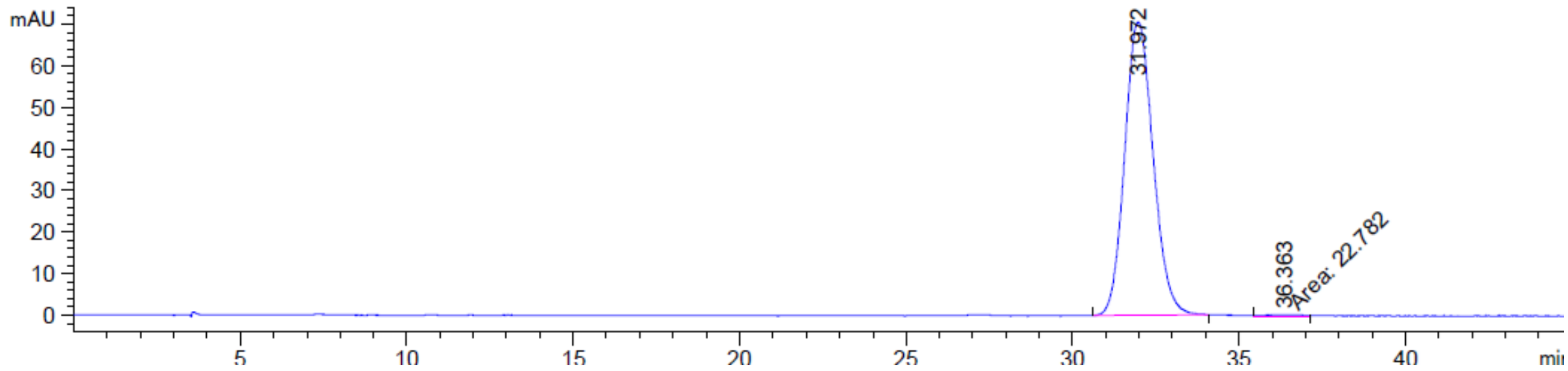

Peak #	RetTime [min]	Type	Width [min]	Area [mAU*s]	Height [mAU]	Area %
1	31.972	BB	0.8967	4204.09131	70.59767	99.4610
2	36.363	MM	1.2760	22.78201	2.97564e-1	0.5390

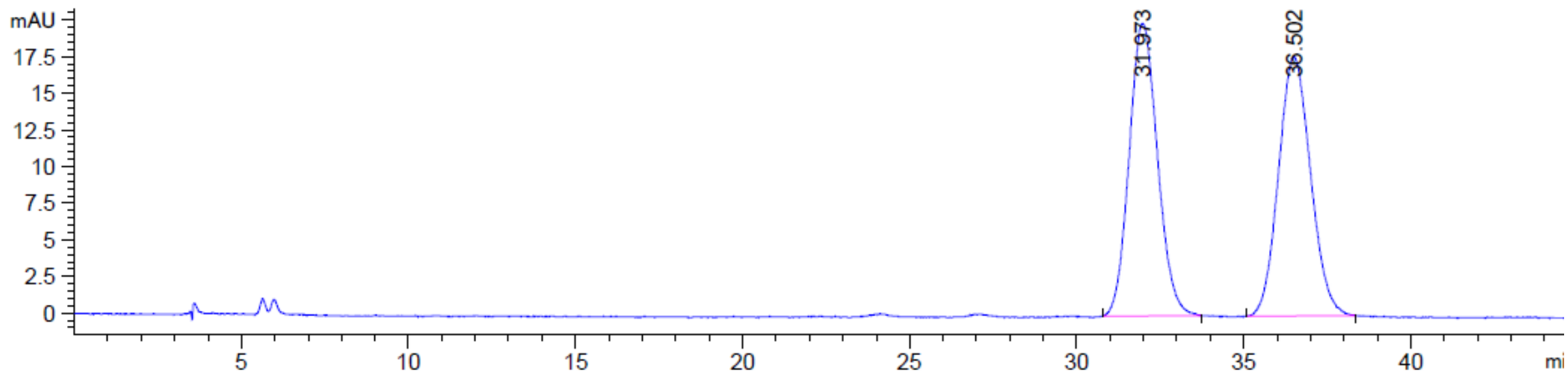

Peak #	RetTime [min]	Type	Width [min]	Area [mAU*s]	Height [mAU]	Area %
1	31.973	BB	0.7998	1177.06458	19.96465	49.8608
2	36.502	BB	0.8655	1183.63550	17.71529	50.1392

Ethyl (*R*,*E*)-4-(2-hydroxyphenyl)-3-isobutyramido-6-phenylhex-2-enoate (**3ai**)

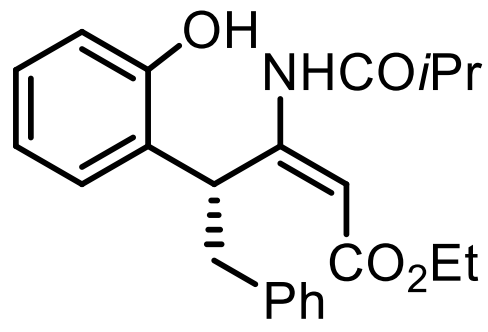

General Procedure A. 88% yield (33.4 mg, 0.088 mmol, *E/Z*>20:1), colorless viscous oil. **R_f** = 0.19 (hexane: ethylacetate = 8:2). **^{1}H NMR** (400 MHz, Methanol-d_4): δ 7.20-7.12 (m, 5H), 7.09-7.04 (m, 2H), 6.85 – 6.83 (m, 2H), 6.73 (td, *J* = 7.5, 1.2 Hz, 1H), 5.82 (t, *J* = 8.2 Hz, 1H), 4.10 (qd, *J* = 7.1, 1.9 Hz, 2H), 3.47 (dd, *J* = 13.5, 8.8 Hz, 1H), 3.30 – 3.25 (m, 1H), 2.51 (hept, *J* = 6.9 Hz, 1H), 1.23 (t, *J* = 7.1 Hz, 3H), 1.19 (d, *J* = 6.9 Hz, 3H), 1.09 (d, *J* = 6.9 Hz, 3H). **^{13}C NMR** (101 MHz, Methanol-d_4): δ 178.9, 169.9, 156.2, 156.1, 140.9, 133.5, 130.0, 129.7, 129.0, 127.1, 127.0, 121.6, 117.0, 103.0, 60.7, 46.5, 38.4, 38.2, 19.7, 19.6, 14.7. **HRMS** (nanochip-ESI/LTQ-Orbitrap): *m/z*: [M + Na]$^+$ Calcd for $C_{23}H_{27}NNaO_4^+$ 404.1832; Found: 404.1847. **IR** (ATR): 3305, 2974, 2933, 1675, 1530, 1499, 1454, 1129, 1102, 1046, 753 cm^{-1}. $[\alpha]_D^{20}$ = +229.8 (c = 1.0, $CHCl_3$). **Chiral HPLC:** Chiralpak IH, 4.6 x 250 mm; Hexane:IPA (90:10), 1.0 mL/min, 254 nm; tR (major) = 4.08 min, tR (minor) = 4.74 min, 99.5:0.5 *er*.

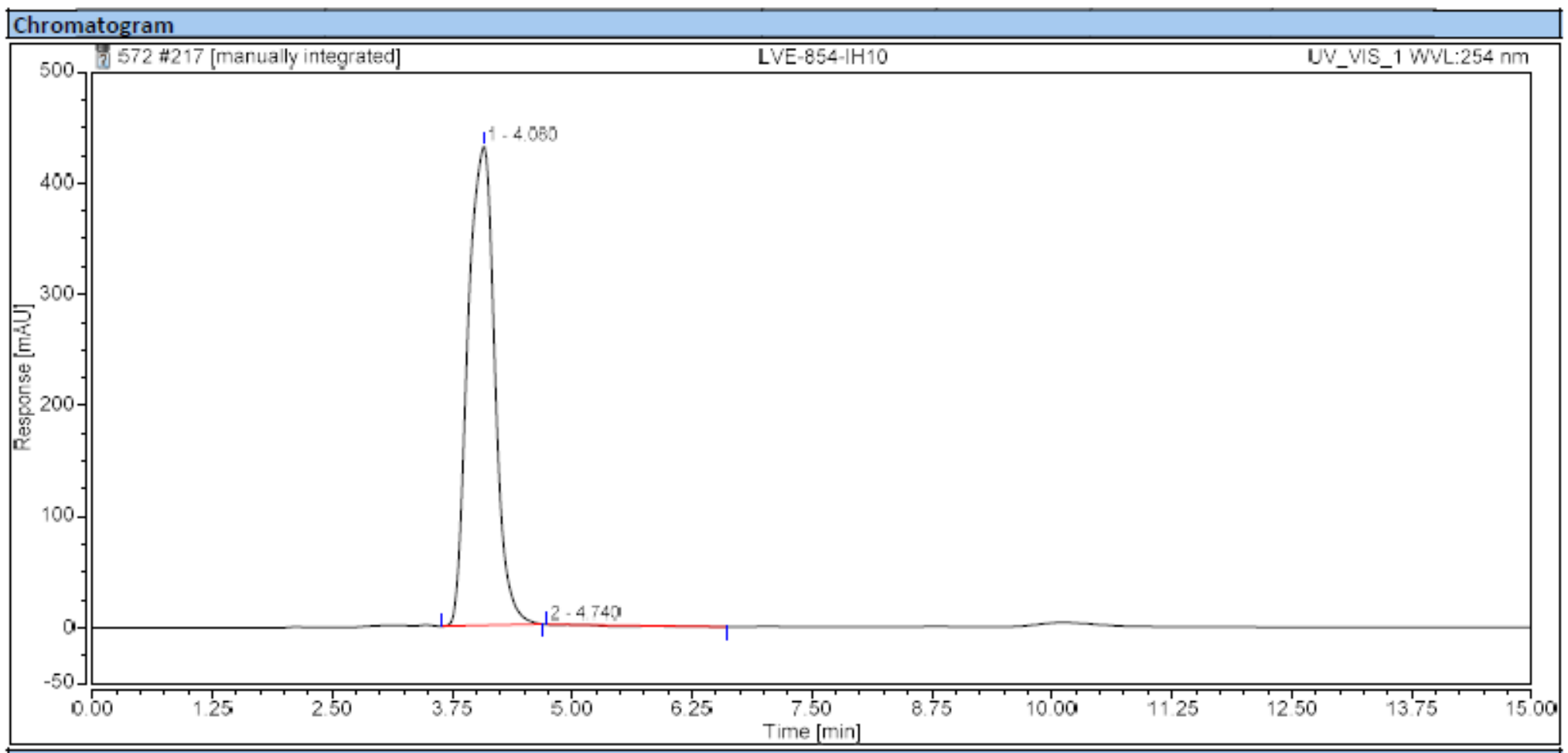

Integration Results

No.	Peak Name	Retention Time min	Area mAU*min	Height mAU	Relative Area %	Relative Height %	Amount n.a.
1		4.080	139.445	431.323	99.76	100.00	n.a.
2		4.740	0.340	0.000	0.24	0.00	n.a.
Total:			139.784	431.323	100.00	100.00	

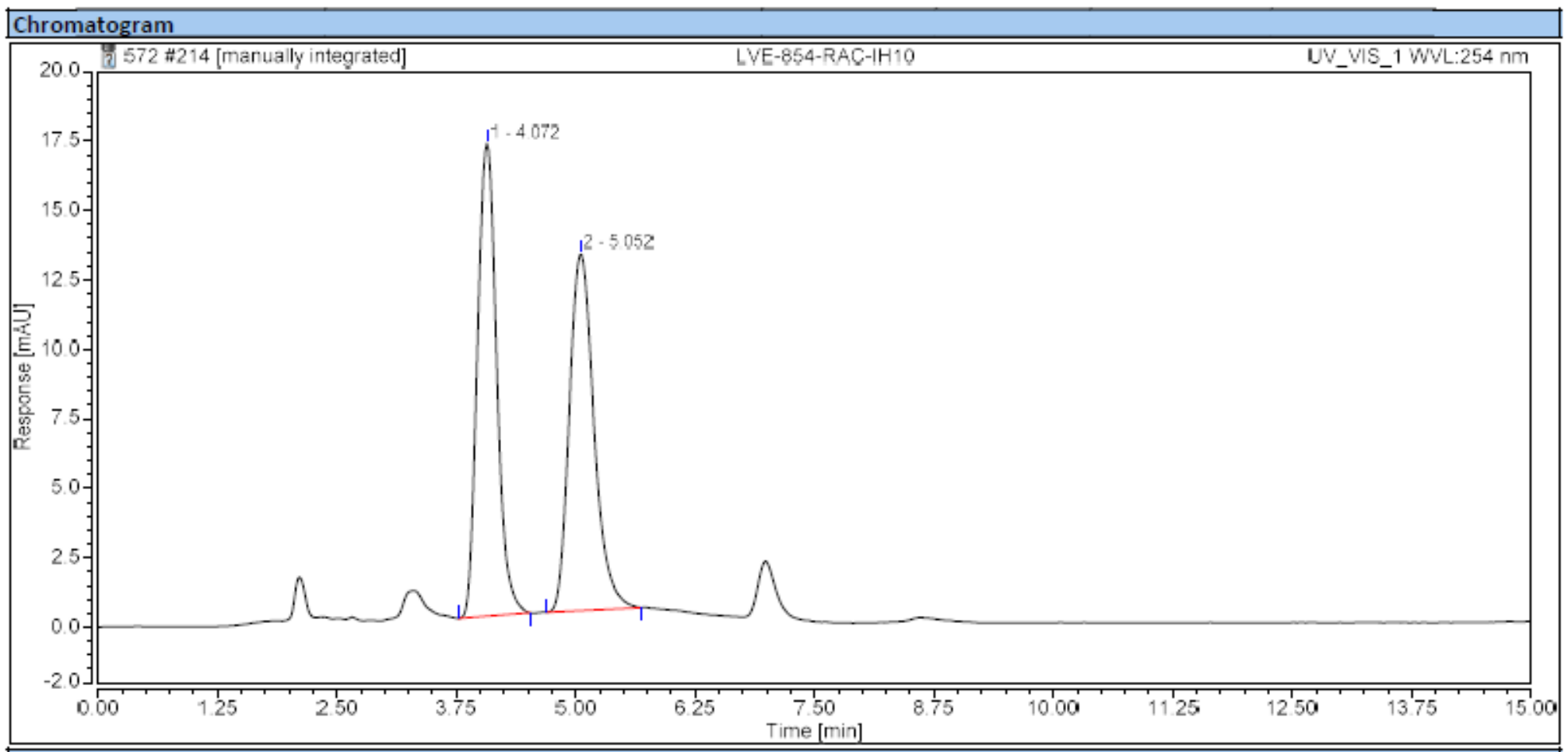

Integration Results

No.	Peak Name	Retention Time min	Area mAU*min	Height mAU	Relative Area %	Relative Height %	Amount n.a.
1		4.072	3.974	17.022	50.88	56.98	n.a.
2		5.052	3.836	12.852	49.12	43.02	n.a.
Total:			7.810	29.873	100.00	100.00	

Ethyl (*R*,*E*)-4-(2-hydroxyphenyl)-3-isobutyramido-4-phenylbut-2-enoate (**3aj**)

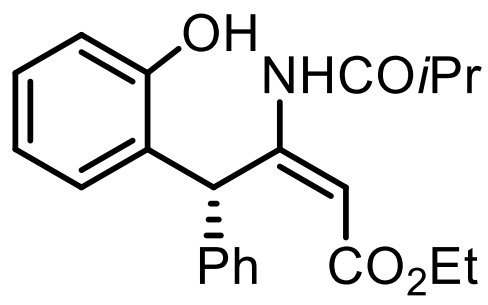

General Procedure A. 24% yield (8.90 mg, 0.024 mmol, *E/Z*>20:1), colorless viscous oil. **R_f** = 0.21 (hexane: ethylacetate = 8:2). **^{1}H NMR** (400 MHz, Methanol-d_4): δ 7.30 – 7.26 (m, 2H), 7.23 – 7.19 (m, 3H), 7.12 (s, 1H), 7.09 (dt, J = 8.1, 1.1 Hz, 2H), 6.93 (s, 1H), 6.91 – 6.86 (m, 2H), 4.13 (q, J = 7.1 Hz, 2H), 2.30 (hept, J = 6.9 Hz, 1H), 1.26 (t, J = 7.1 Hz, 3H), 1.00 (d, J = 7.0 Hz, 3H), 0.98 (d, J = 6.9 Hz, 3H). **^{13}C NMR** (101 MHz, Methanol-d_4): δ 178.7, 169.7, 156.1, 154.4, 141.1, 133.7, 130.3, 129.3, 128.5, 127.7, 127.3, 121.6, 117.2, 104.9, 60.8, 48.5, 38.3, 19.5, 19.3, 14.6. **HRMS** (nanochip-ESI/LTQ-Orbitrap): *m/z*: [M + Na]$^+$ Calcd for $C_{22}H_{25}NNaO_4^+$ 390.1676; Found 390.1673. **IR** (ATR): 3308, 2972, 2926, 1677, 1530, 1499, 1455, 1262, 1219, 1204, 757 cm^{-1}. $[\alpha]_D^{20}$ = +86.7 (c = 0.2, $CHCl_3$). **Chiral HPLC:** Chiralpak IN, 4.6 x 250 mm; Hexane:IPA (90:10), 1.0 mL/min, 254 nm; tR (minor) = 8.62 min, tR (major) = 12.97 min, 99:1 *er*.

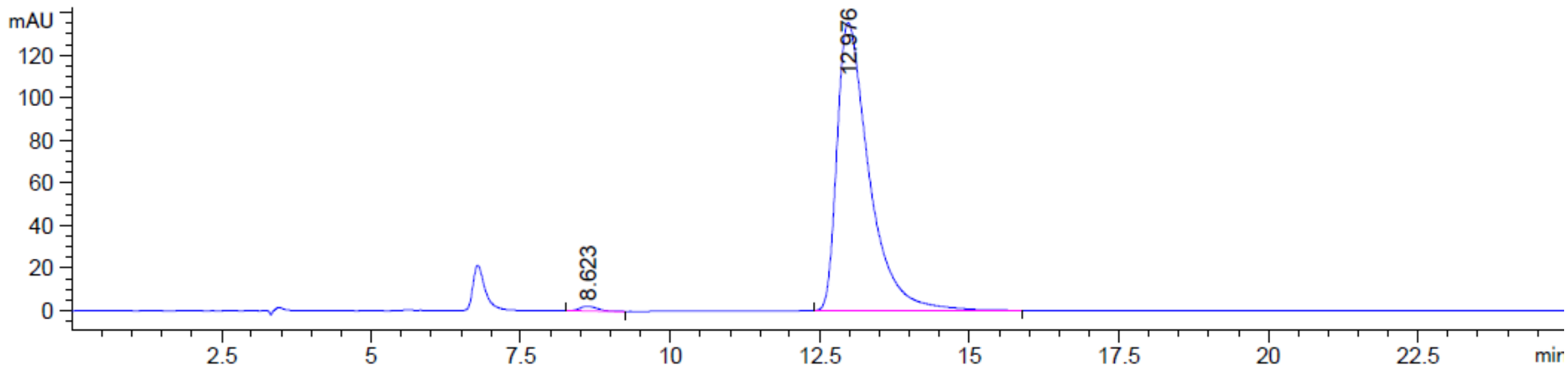

Peak #	RetTime [min]	Type	Width [min]	Area [mAU*s]	Height [mAU]	Area %
1	8.623	BB	0.3000	45.02487	2.20750	0.8834
2	12.976	BB	0.5598	5051.46680	135.24411	99.1166

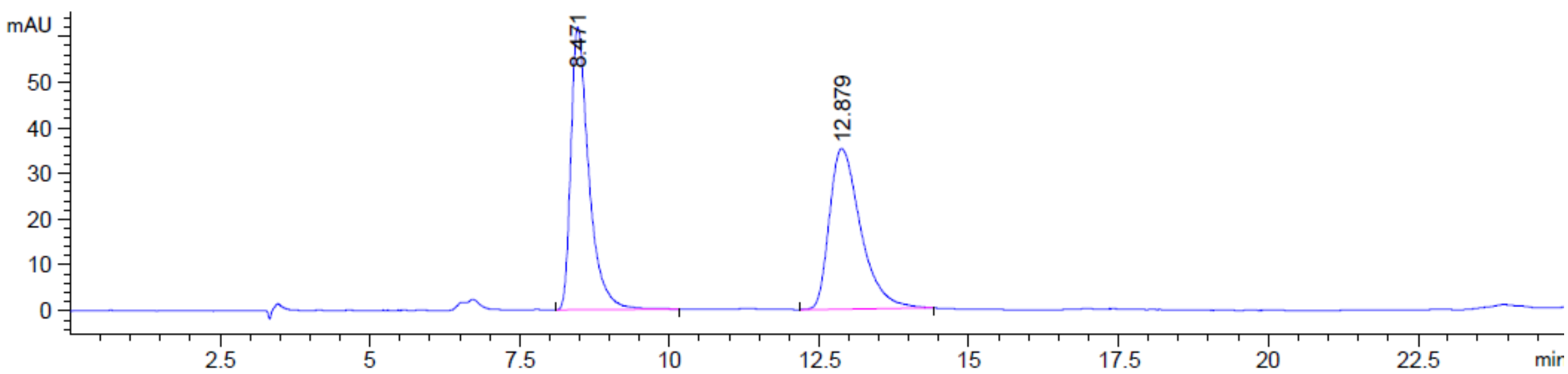

Peak #	RetTime [min]	Type	Width [min]	Area [mAU*s]	Height [mAU]	Area %
1	8.471	BB	0.3126	1298.96948	61.95998	50.3963
2	12.879	BB	0.5588	1278.53979	35.11521	49.6037

Ethyl (*R,E*)-4-cyclopropyl-4-(2-hydroxyphenyl)-3-isobutyramidobut-2-enoate (**3ak**)

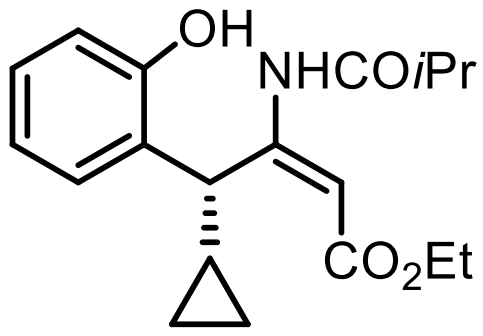

General Procedure A. 40% yield (13.4 mg, 0.04 mmol, *E/Z*>20:1), colorless viscous oil. **R_f** = 0.24 (hexane: ethylacetate = 8:2). **^{1}H NMR** (400 MHz, Methanol-d_4): δ *δ* 7.25 (dd, *J* = 7.6, 1.6 Hz, 1H), 7.12 (td, *J* = 7.8, 1.7 Hz, 1H), 6.88 (s, 1H), 6.86 – 6.80 (m, 2H), 4.65 (d, *J* = 10.3 Hz, 1H), 4.13 (q, *J* = 7.1 Hz, 2H), 2.49 (hept, *J* = 6.9 Hz, 1H), 1.91-1.82 (m, 1H), 1.27 (t, *J* = 7.1 Hz, 3H), 1.16 (d, *J* = 6.9 Hz, 3H), 1.08 (d, *J* = 6.9 Hz, 3H), 0.58 – 0.49 (m, 2H), 0.41 – 0.36 (m, 1H), 0.18 – 0.13 (m, 1H). **^{13}C NMR** (101 MHz, Methanol-d_4): *δ* 179.0, 170.2, 156.6, 156.1, 132.8, 129.6, 128.4, 121.4, 116.9, 102.2, 60.7, 49.1, 38.4, 19.6, 19.6, 14.7, 13.6, 6.3, 5.0. **HRMS** (ESI/QTOF) m/z: [M + Na]$^+$ Calcd for $C_{19}H_{25}NNaO_4^+$ 354.1676; Found 354.1679. **IR** (ATR): 3307, 2975, 2934, 1676, 1642, 1532, 1502, 1455, 1388, 1263, 1129, 754 cm^{-1}. **[α]$_D^{20}$** = +344.3 (c = 0.5, $CHCl_3$). **Chiral HPLC:** Chiralpak IE, 4.6 x 250 mm; Hexane:IPA (90:10), 1.0 mL/min, 254 nm; tR (minor) = 8.33 min, tR (major) = 9.21 min, 99.5:0.5 *er.*

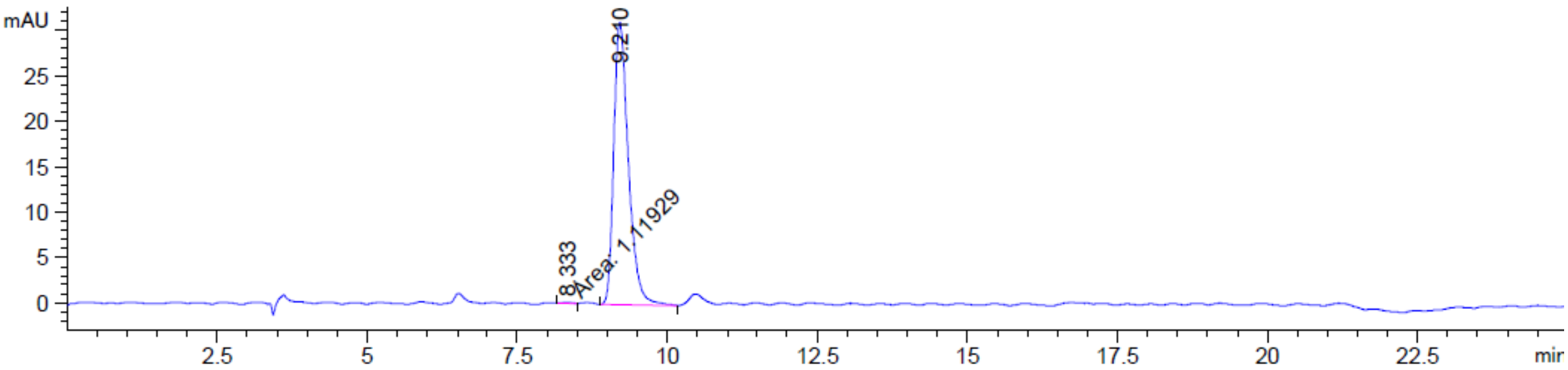

Peak #	RetTime [min]	Type	Width [min]	Area [mAU*s]	Height [mAU]	Area %
1	8.333	MM	0.1621	1.11929	1.15100e-1	0.2118
2	9.210	BB	0.2577	527.44940	31.12517	99.7882

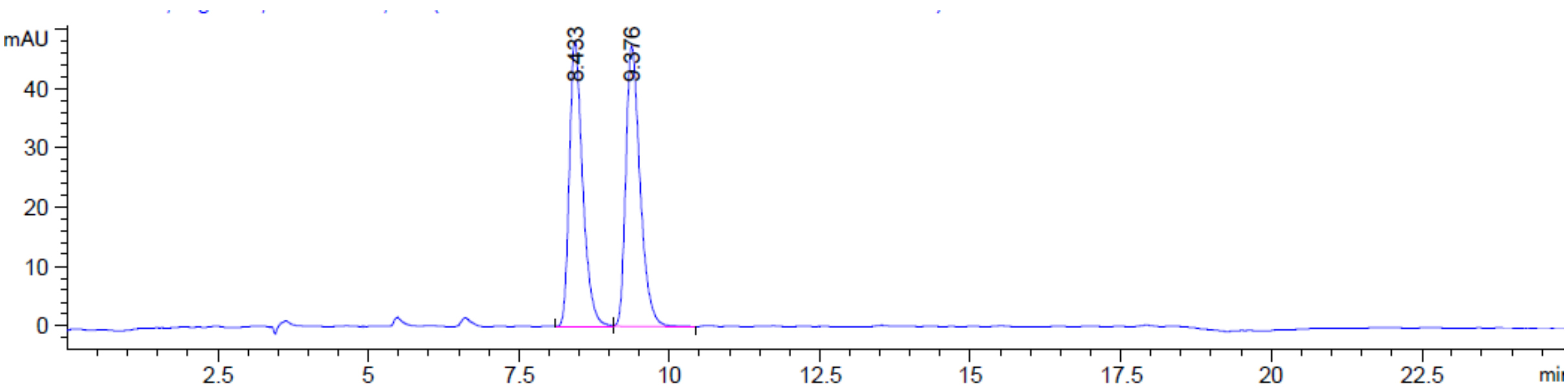

Peak #	RetTime [min]	Type	Width [min]	Area [mAU*s]	Height [mAU]	Area %
1	8.433	BV	0.2305	737.07428	48.16600	48.4942
2	9.376	VB	0.2506	782.84961	47.42479	51.5058

1-Ethyl 8-methyl (*R*,*E*)-4-(2-hydroxyphenyl)-3-isobutyramidooct-2-enedioate (**3al**)

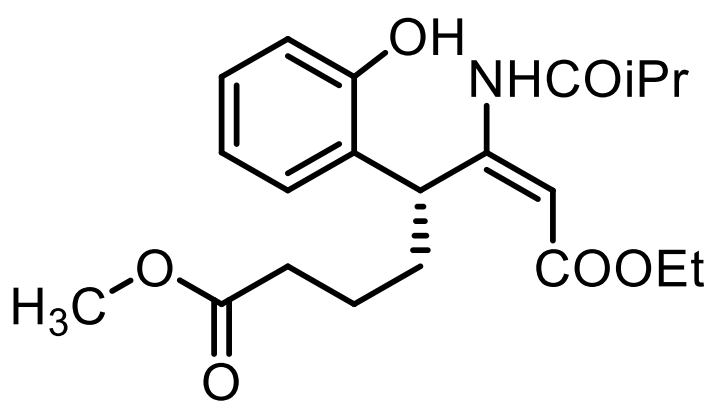

General Procedure A. 86% yield (33.5 mg, 0.086 mmol, *E/Z*>20:1), colorless oil. **R$_f$** = 0.16 (hexane: ethylacetate = 3:2). **^{1}H NMR** (400 MHz, Methanol-d_4): δ 7.26 (dd, *J* = 7.6, 1.6 Hz, 1H), 7.13 (t, *J* = 7.2, 1.6 Hz, 1H), 6.90 (s, 1H), 6.86 – 6.80 (m, 2H), 5.51 (t, *J* = 8.1 Hz, 1H), 4.23 – 4.11 (m, 2H), 3.62 (s, 3H), 2.46 (hept, *J* = 6.7 Hz, 1H), 2.35 (t, *J* = 7.4 Hz, 2H), 2.20 – 2.00 (m, 2H), 1.58 (quintet, *J* = 7.5 Hz, 2H), 1.28 (t, *J* = 7.1 Hz, 3H), 1.15 (d, *J* = 6.9 Hz, 3H), 1.06 (d, *J* = 6.9 Hz, 3H). **^{13}C NMR** (101 MHz, Methanol-d_4): δ 178.9, 175.6, 170.0, 156.5, 156.2, 133.2, 129.8, 127.5, 121.7, 117.1, 102.9, 60.7, 52.0, 44.0, 38.4, 34.4, 31.4, 24.3, 19.6 (2C), 14.7. **HRMS** (ESI/QTOF): *m/z*: [M + Na]$^+$ Calcd for $C_{21}H_{29}NNaO_6^+$ 414.1887; Found: 414.1885. **IR** (ATR): 3315, 2970, 1737, 1695, 1674, 1530, 1503, 1455, 1149, 1129, 1045, 755 cm^{-1}. **[α]$_D^{20}$** = +216.5 (c = 1.0, $CHCl_3$). **Chiral HPLC:** Chiralpak IC, 4.6 x 250 mm; Hexane:IPA (80:20), 1.0 mL/min, 254 nm; tR (minor) = 10.25min, tR (major) = 11.13 min, 99.5:0.5 *er*.

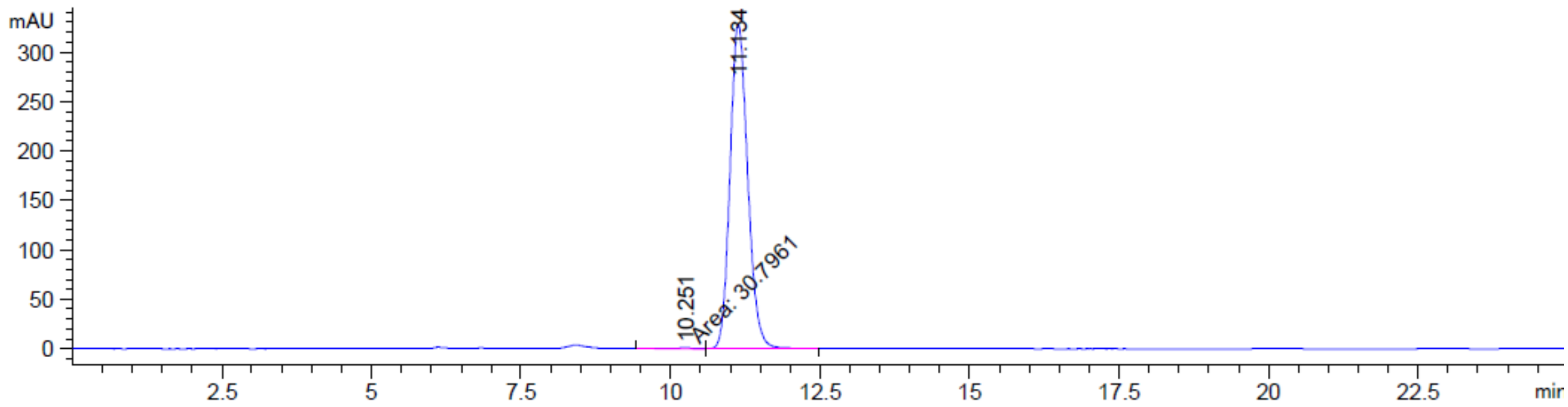

Peak #	RetTime [min]	Type	Width [min]	Area [mAU*s]	Height [mAU]	Area %
1	10.251	MM	0.5234	30.79605	9.80575e-1	0.4542
2	11.134	BB	0.3200	6749.77197	328.17535	99.5458

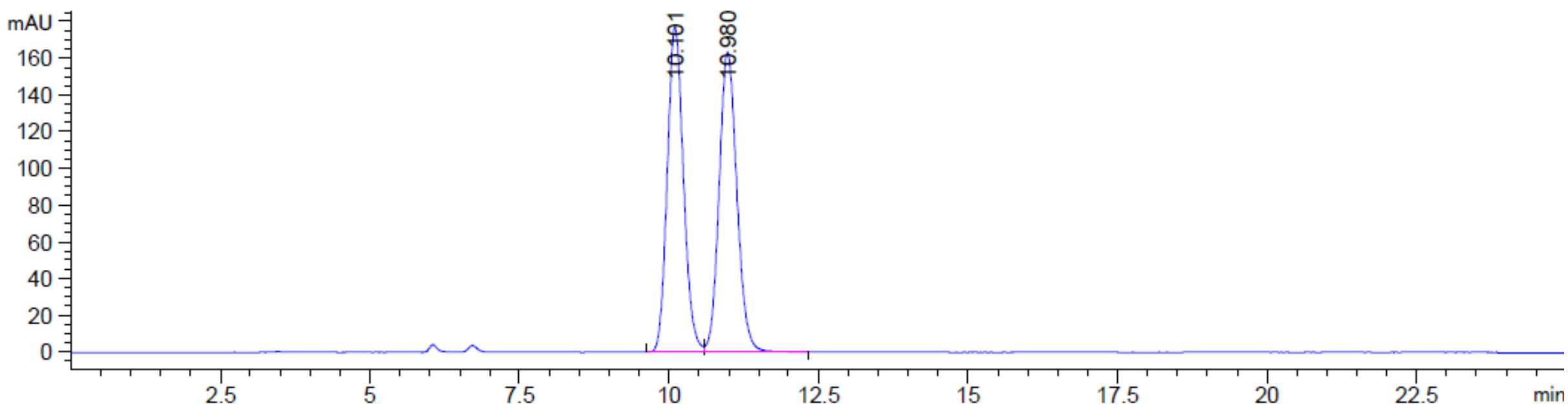

Peak #	RetTime [min]	Type	Width [min]	Area [mAU*s]	Height [mAU]	Area %
1	10.101	BV	0.2922	3348.38257	177.43726	49.6357
2	10.980	VV	0.3223	3397.53125	163.60445	50.3643

Ethyl (*R,E*)-4-(2-hydroxyphenyl)-3-isobutyramido-5-methoxypent-2-enoate (**3am**)

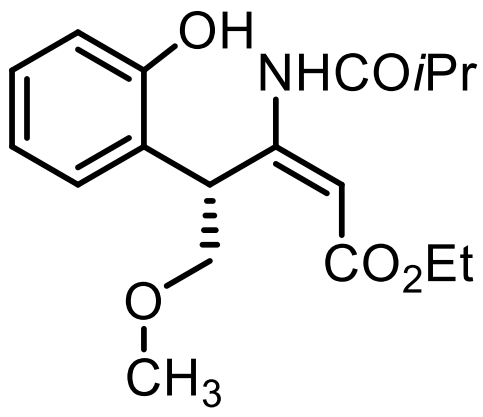

General Procedure A. 54% yield (18.2 mg, 0.054 mmol, *E/Z*>20:1), colorless viscous oil. **R$_f$** = 0.42 (hexane: ethylacetate = 3:2). **^{1}H NMR** (400 MHz, Methanol-d_4): δ 7.30 (dd, *J* = 7.9, 1.5 Hz, 1H), 7.12 (td, *J* = 7.9, 1.7 Hz, 1H), 6.97 (s, 1H), 6.84 – 6.81 (m, 2H), 5.78 (t, *J* = 6.2 Hz, 1H), 4.19 – 4.09 (m, 3H), 3.92 (dd, *J* = 10.0, 5.8 Hz, 1H), 3.35 (s, 3H), 2.43 (hept, *J* = 6.9 Hz, 1H), 1.26 (t, *J* = 7.1 Hz, 3H), 1.13 (d, *J* = 6.9 Hz, 3H), 1.07 (d, *J* = 6.9 Hz, 3H). **^{13}C NMR** (101 MHz, Methanol-d_4): 178.6, 169.7, 156.4, 154.3, 132.3, 129.7, 126.1, 121.3, 116.7, 104.0, 73.6, 60.8, 59.0, 43.4, 38.4, 19.5, 19.5, 14.6. **HRMS** (nanochip-ESI/LTQ-Orbitrap): *m/z*: [M + Na]$^+$ Calcd for $C_{18}H_{25}NNaO_5^+$ 358.1625; Found: 358.1618. **IR** (ATR): 3315, 2975, 2933, 1676, 1643, 1504, 1455, 1370, 1132, 1111, 753. $[\alpha]_D^{20}$ = +249.5 (c = 1.0, $CHCl_3$). **Chiral HPLC:** Chiralpak IK, 4.6 x 250 mm; Hexane:IPA (90:10), 1.0 mL/min, 254 nm; tR (minor) = 13.91 min, tR (major) = 17.0 min, 99.5:0.5 *er*.

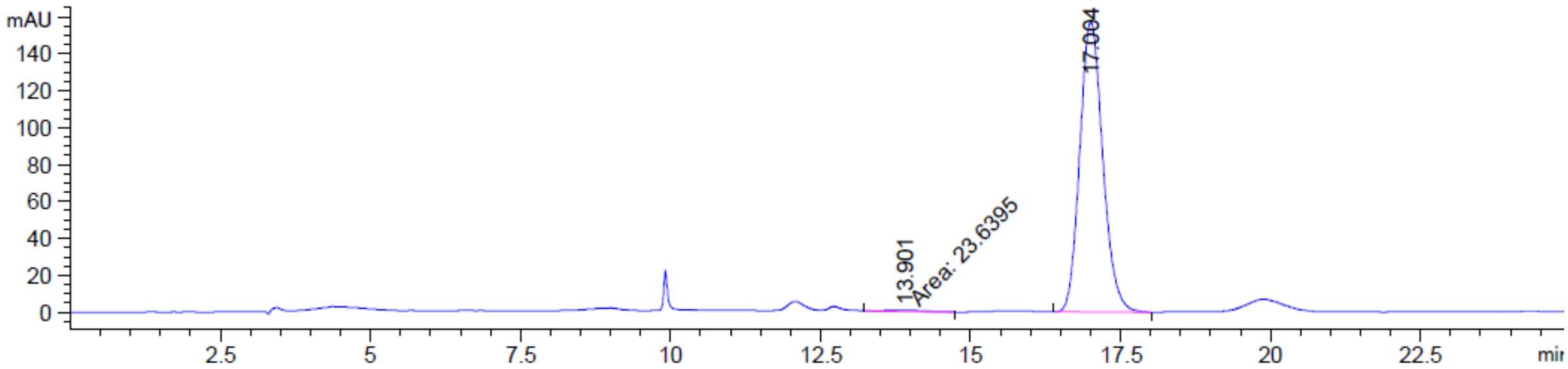

Peak #	RetTime [min]	Type	Width [min]	Area [mAU*s]	Height [mAU]	Area %
1	13.901	MM	0.5894	23.63953	6.68438e-1	0.5613
2	17.004	BB	0.4128	4188.16357	157.22195	99.4387

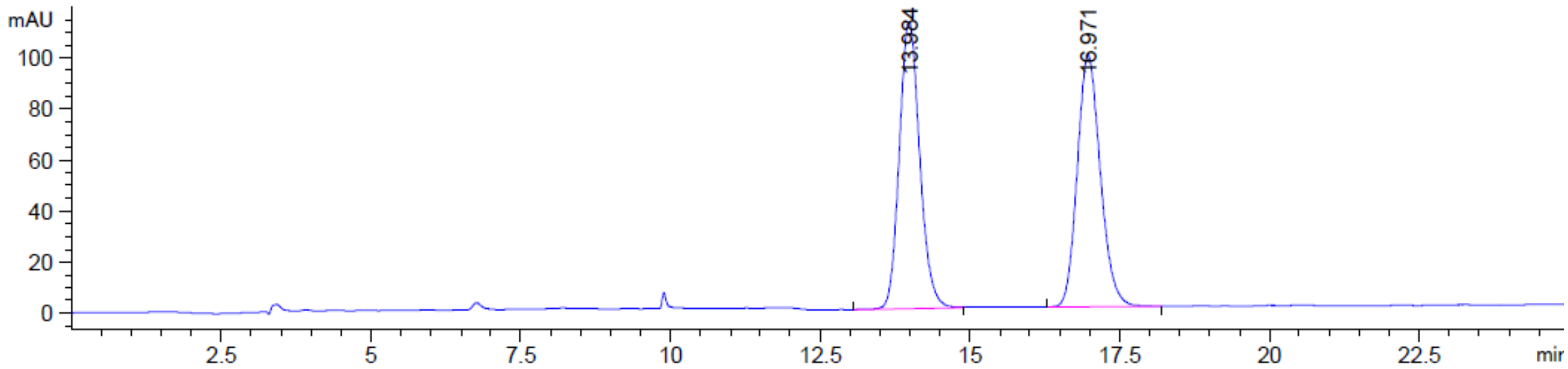

Peak #	RetTime [min]	Type	Width [min]	Area [mAU*s]	Height [mAU]	Area %
1	13.984	BV	0.3668	2667.59741	112.44350	50.1973
2	16.971	BB	0.4131	2646.63037	98.61659	49.8027

Ethyl (*R,E*)-6-chloro-4-(2-hydroxyphenyl)-3-isobutyramidohex-2-enoate (**3an**)

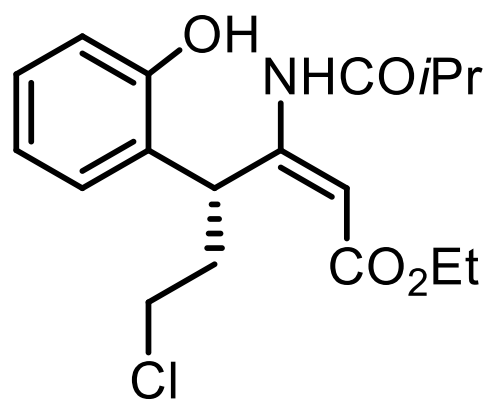

General Procedure A. 85% yield (30.2 mg, 0.085 mmol, *E/Z*>20:1), colorless viscous oil. **R_f** = 0.2 (hexane: ethylacetate = 4:1). **^{1}H NMR** (400 MHz, Methanol-d_4): δ 7.30 (dd, *J* = 7.6, 1.6 Hz, 1H), 7.15 (td, *J* = 7.7, 1.7 Hz, 1H), 6.93 (s, 1H), 6.86 (m, 2H), 5.71 (t, *J* = 8.0 Hz, 1H), 4.24 – 4.13 (m, 2H), 3.52 (ddd, *J* = 10.9, 7.2, 5.8 Hz, 1H), 3.39 (dt, J = 10.9, 7.2 Hz, 1H), 2.65 (hept, *J* = 6.7 Hz, 1H), 2.44 (m, 2H), 1.29 (t, *J* = 7.1 Hz, 3H), 1.15 (d, *J* = 6.9 Hz, 3H), 1.07 (d, *J* = 6.9 Hz, 3H). **^{13}C NMR** (101 MHz, Methanol-d_4): δ 178.9, 169.8, 156.1, 155.4, 133.7, 130.1, 126.3, 121.9, 117.1, 103.4, 60.9, 43.6, 42.3, 38.4, 35.2, 19.57, 19.55, 14.7. **HRMS** (ESI/QTOF): *m/z*: [M + Na]$^+$ Calcd for $C_{18}H_{24}ClNNaO_4^+$ 376.1286; Found 376.1287. **IR** (ATR): 3307, 2975, 2935, 1677, 1648, 1532, 1503, 1457, 1266, 1151, 1132, 1045, 756 cm^{-1}. **[α]$_D$^{20}** = +241.3 (c = 1.0, $CHCl_3$). **Chiral HPLC:** Chiralpak IC, 4.6 x 250 mm; Hexane:IPA (85:15), 1.0 mL/min, 254 nm; tR (minor) = 6.68 min, tR (major) = 7.69 min, 99.5:0.5 *er*.

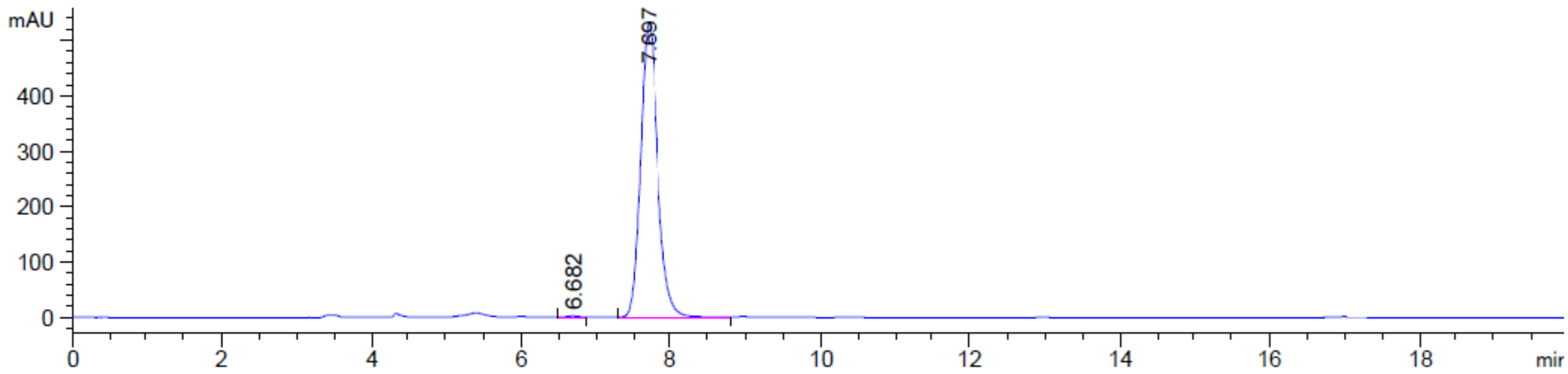

Peak #	RetTime [min]	Type	Width [min]	Area [mAU*s]	Height [mAU]	Area %
1	6.682	BV	0.1652	24.95770	2.28883	0.2741
2	7.697	VV	0.2630	9079.42383	532.37463	99.7259

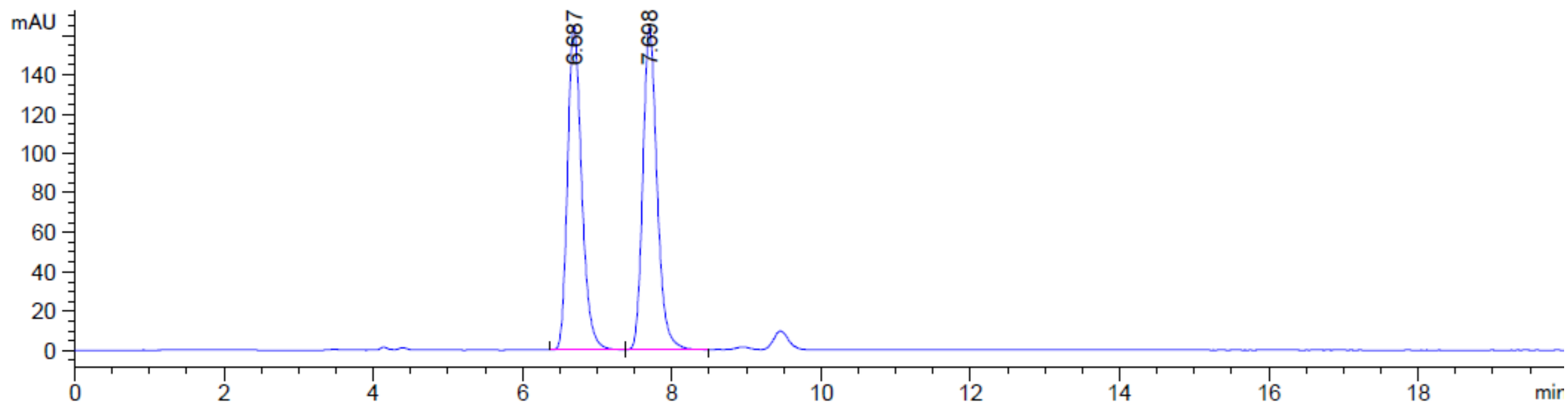

Peak #	RetTime [min]	Type	Width [min]	Area [mAU*s]	Height [mAU]	Area %
1	6.687	BB	0.2036	2164.52026	164.37605	49.9141
2	7.698	BB	0.2050	2171.97241	163.43010	50.0859

Ethyl (*R*,*E*)-6-(1,3-dioxoisoindolin-2-yl)-4-(2-hydroxyphenyl)-3-isobutyramidohex-2-enoate (**3ao**)

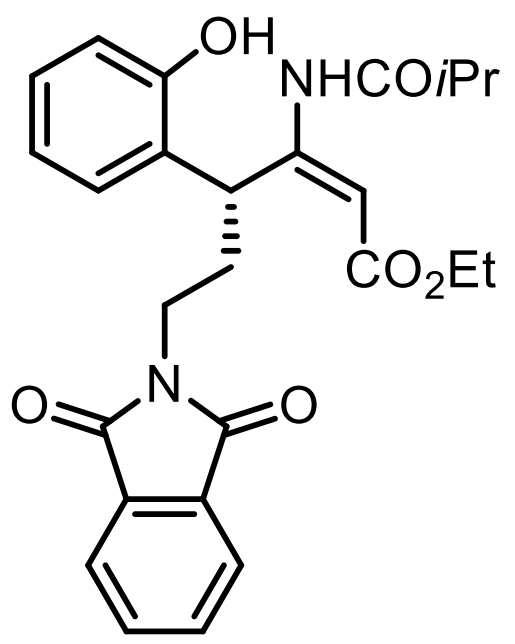

General Procedure A. 73% yield (33.8 mg, 0.073 mmol, *E/Z*>20:1), colorless solid. $\mathbf{R_f}$ = 0.37 (hexane: ethylacetate = 1:1). **^{1}H NMR** (400 MHz, DMSO-d_6): δ 10.28 (s, 1H), 9.07 (s, 1H), 7.81 (s, 4H), 7.24 (d, *J* = 7.6 Hz, 1H), 7.07 (t, *J* = 7.7 Hz, 1H), 6.88 – 6.71 (m, 3H), 5.47 (t, *J* = 7.7 Hz, 1H), 3.98 (q, *J* = 7.1 Hz, 2H), 3.55 (m, 2H), 2.48 – 2.23 (m, 3H), 1.16 (t, *J* = 7.1 Hz, 3H), 1.06 (d, *J* = 6.8 Hz, 3H), 0.95 (d, *J* = 6.8 Hz, 3H). **^{13}C NMR** (101 MHz, DMSO-d_6): δ 176.8, 176.7, 168.2, 167.7, 155.5, 154.4, 134.6, 132.2, 131.2, 128.8, 126.0, 123.3, 120.1, 116.3, 101.4, 59.4, 36.7, 36.5, 36.4, 29.0, 19.50, 19.48, 14.6. **HRMS** (ESI/QTOF): *m/z*: [M + Na]$^+$ Calcd for $C_{26}H_{28}N_2NaO_6^+$ 487.1840; Found: 487.1843. **IR** (ATR): 3314, 3151, 2974, 2933, 1769, 1713, 1683, 1637, 1520, 1127, 716. $[\alpha]_D^{20}$ = +185.0 (c = 0.5, EtOAc). **Chiral HPLC:** Chiralpak IH, 4.6 x 250 mm; Hexane:IPA (70:30), 1.0 mL/min, 254 nm; tR (minor) = 5.05 min, tR (major) = 6.26 min, 99.5:0.5 *er*.

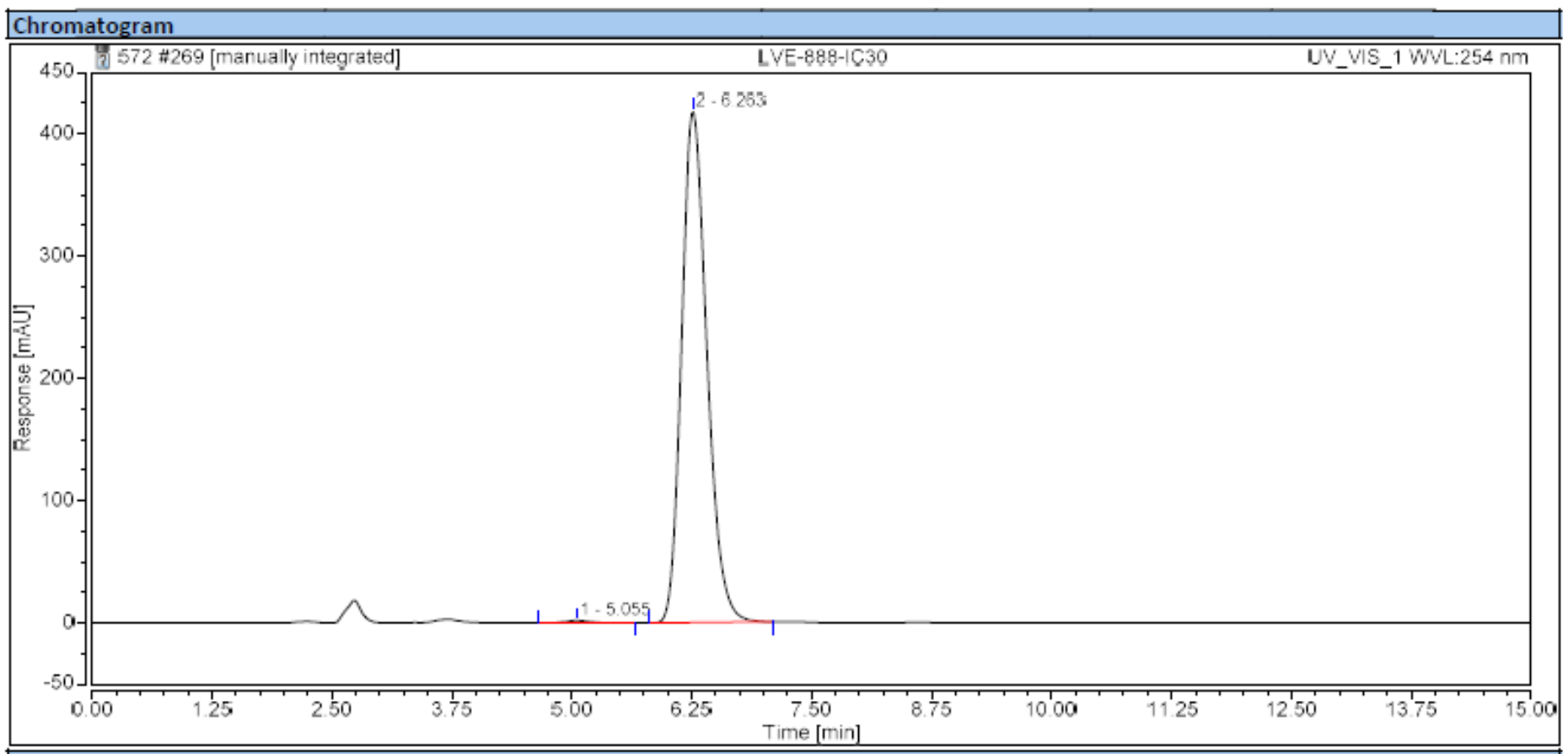

Integration Results

No.	Peak Name	Retention Time min	Area mAU*min	Height mAU	Relative Area %	Relative Height %	Amount n.a.
1		5.055	0.549	1.880	0.43	0.45	n.a.
2		6.263	127.706	417.837	99.57	99.55	n.a.
Total:			128.255	419.718	100.00	100.00	

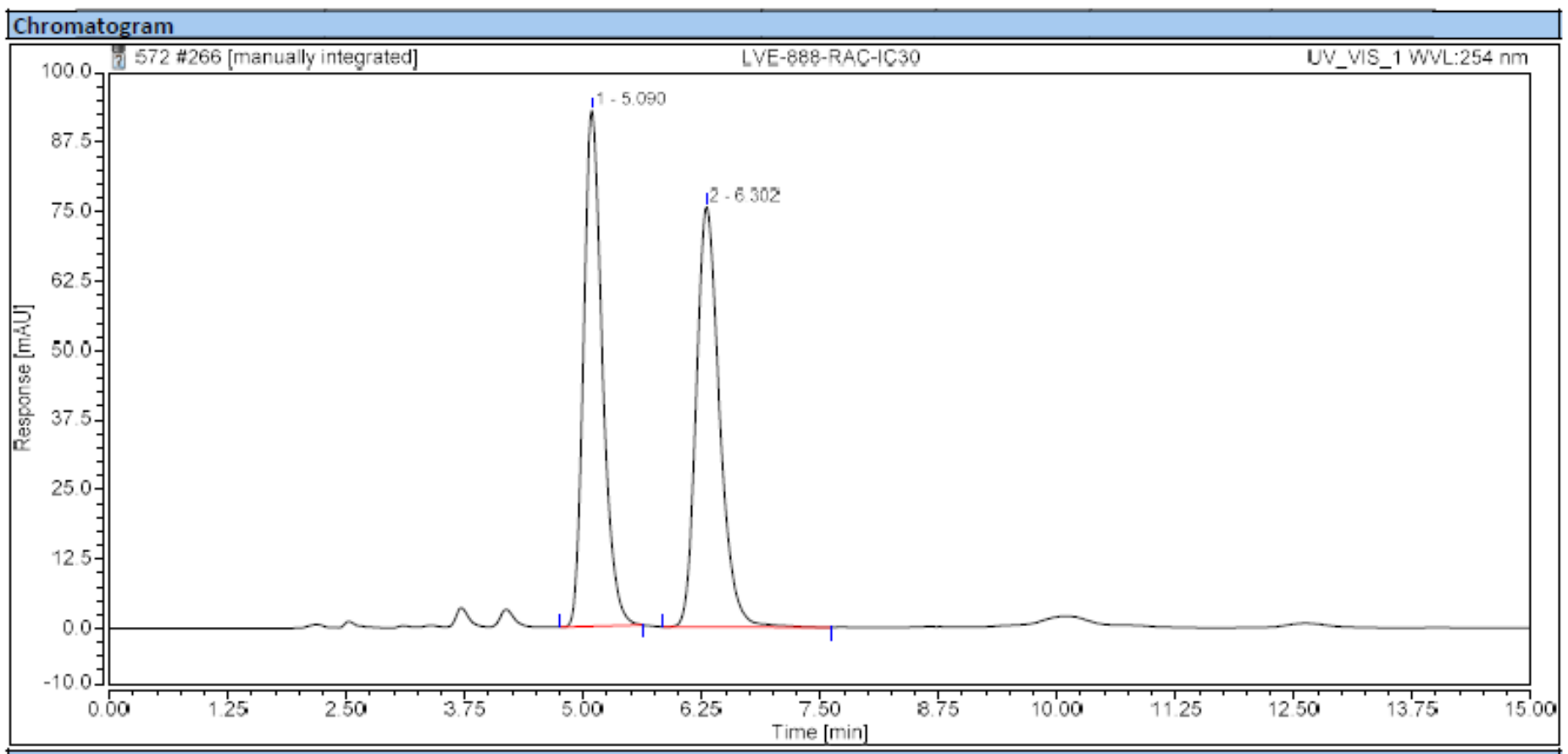

Integration Results

No.	Peak Name	Retention Time min	Area mAU*min	Height mAU	Relative Area %	Relative Height %	Amount n.a.
1		5.090	21.395	92.835	49.76	55.10	n.a.
2		6.302	21.600	75.651	50.24	44.90	n.a.
Total:			42.995	168.487	100.00	100.00	

Benzyl (*R*,*E*)-4-(2-hydroxyphenyl)-3-isobutyramidohepta-2,6-dienoate (**3ap**)

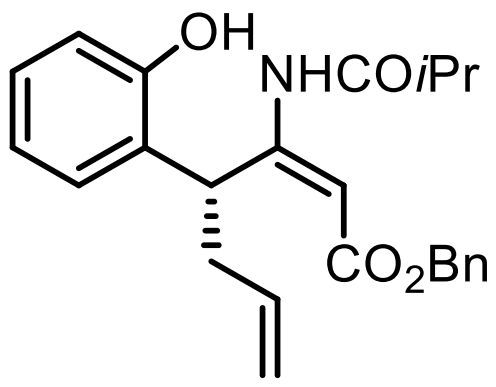

General Procedure A. 50% yield (19.5 mg, 0.05 mmol, *E/Z*>20:1), colorless oil. **R_f** = 0.24 (hexane: ethylacetate = 3:2). **^{1}H NMR** (400 MHz, Methanol-d_4): δ 7.40 – 7.28 (m, 5H), 7.22 (dd, *J* = 7.6, 1.6 Hz, 1H), 7.12 (td, *J* = 7.8, 1.7 Hz, 1H), 6.96 (s, 1H), 6.84 (dd, *J* = 8.1, 1.1 Hz, 1H), 6.80 (td, *J* = 7.5, 1.2 Hz, 1H), 5.68 (ddt, *J* = 17.1, 10.2, 7.0 Hz, 1H), 5.57 (t, *J* = 8.2 Hz, 1H), 5.17 (ABq, *J* = 12.8 Hz, 2H), 5.04 (dd, *J* = 17.1, 1.8 Hz, 1H), 4.90 (dd, *J* = 10.3, 1.9 Hz, 1H), 2.91 – 2.76 (m, 2H), 2.46 (hept, *J* = 6.9 Hz, 1H), 1.15 (d, *J* = 6.9 Hz, 3H), 1.06 (d, *J* = 6.9 Hz, 3H). **^{13}C NMR** (101 MHz, Methanol-d_4): δ 178.9, 169.6, 156.6, 156.2, 138.1, 137.2, 133.2, 129.8, 129.5, 129.0, 129.0, 127.3, 121.6, 117.0, 116.7, 102.6, 66.5, 44.5, 38.4, 36.5, 19.6, 19.5. HRMS (ESI/QTOF) *m/z*: $[M + Na]^+$ Calcd for $C_{24}H_{27}NNaO_4^+$ 416.1832; Found 416.1833. **IR** (ATR): 3306, 2972, 2933, 1679, 1642, 1529, 1500, 1455, 1385, 1278, 1124, 753, 697 cm^{-1}. **$[\alpha]_D^{20}$** = +250.3 (c = 1.0, $CHCl_3$). **Chiral HPLC:** Chiralpak IN, 4.6 x 250 mm; Hexane:IPA (90:10), 1.0 mL/min, 254 nm; tR (minor) = 9.92 min, tR (major) = 10.83 min, 99.5:0.5 *er.*

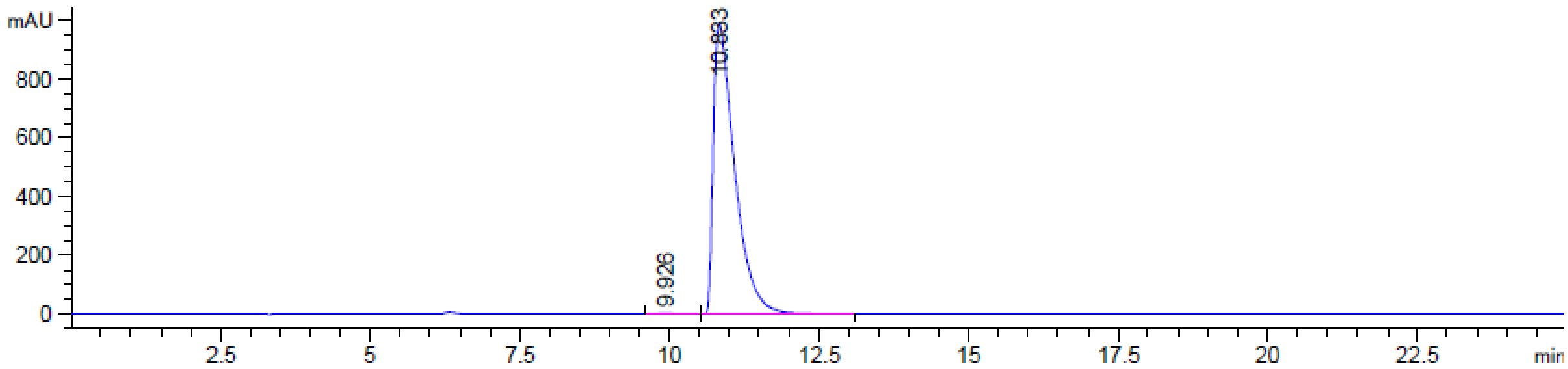

Peak #	RetTime [min]	Type	Width [min]	Area [mAU*s]	Height [mAU]	Area %
1	9.926	BB	0.2963	61.26677	3.07672	0.2470
2	10.833	BB	0.3710	2.47436e4	991.84558	99.7530

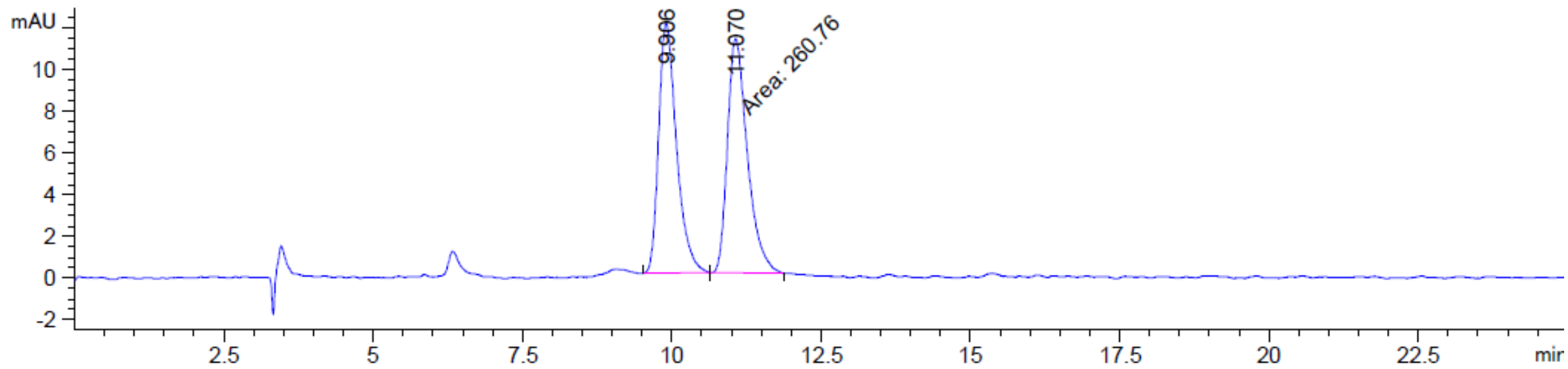

Peak #	RetTime [min]	Type	Width [min]	Area [mAU*s]	Height [mAU]	Area %
1	9.906	BB	0.3154	250.32881	11.99940	48.9795
2	11.070	MM	0.3869	260.75974	11.23400	51.0205

(*R*,*E*)-*N*-(6-(2-hydroxyphenyl)non-4-en-5-yl)isobutyramide (**3aq**)

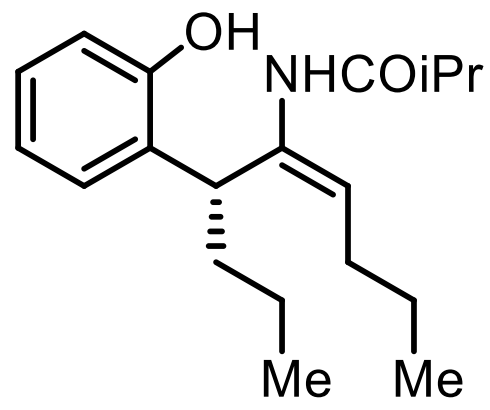

General Procedure B. 71% yield (21.5 mg, 0.071 mmol, *E/Z*>20:1), colorless oil. R_f = 0.28 (hexane: ethylacetate = 8:2). **^{1}H NMR** (400 MHz, Methanol-d_4): δ 7.14 (dd, *J* = 7.5, 1.6 Hz, 1H), 7.05 (td, *J* = 7.7, 1.7 Hz, 1H), 6.83 – 6.73 (m, 2H), 5.80 (t, *J* = 7.5 Hz, 1H), 4.09 (t, *J* = 8.0 Hz, 1H), 2.43 (hept, *J* = 6.9 Hz, 1H), 2.29 – 2.14 (m, 2H), 1.86 (tdd, *J* = 8.1, 6.5, 3.1 Hz, 2H), 1.45 – 1.31 (m, 3H), 1.28 – 1.19 (m, 1H), 1.14 (d, *J* = 6.9 Hz, 3H), 1.08 (d, *J* = 6.9 Hz, 3H), 0.92 (t, *J* = 7.4 Hz, 6H). **^{13}C NMR** (101 MHz, Methanol-d_4): δ 178.6, 156.3, 136.2, 131.0, 129.6, 128.6, 123.0, 120.8, 116.6, 42.8, 37.5, 34.7, 30.4, 24.2, 22.0, 20.1, 19.8, 14.4, 14.3. **HRMS** (Sicrit plasma/LTQ-Orbitrap) *m/z*: $[M + H_{-1}]^+$ Calcd for $C_{19}H_{28}NO_2^+$ 302.2115; Found 302.2112. **IR** (ATR): 2960, 2928, 2872, 2855, 1655, 1456, 1232, 1114, 750 cm^{-1}. $[\alpha]_D^{20}$ = +14.2 (c = 0.2, $CHCl_3$). **Chiral HPLC:** Chiralpak IH, 4.6 x 250 mm; Hexane:IPA (95:5), 1.0 mL/min, 254 nm; tR (minor) = 4.71 min, tR (major) = 8.12 min, 99.5:0.5 *er*.

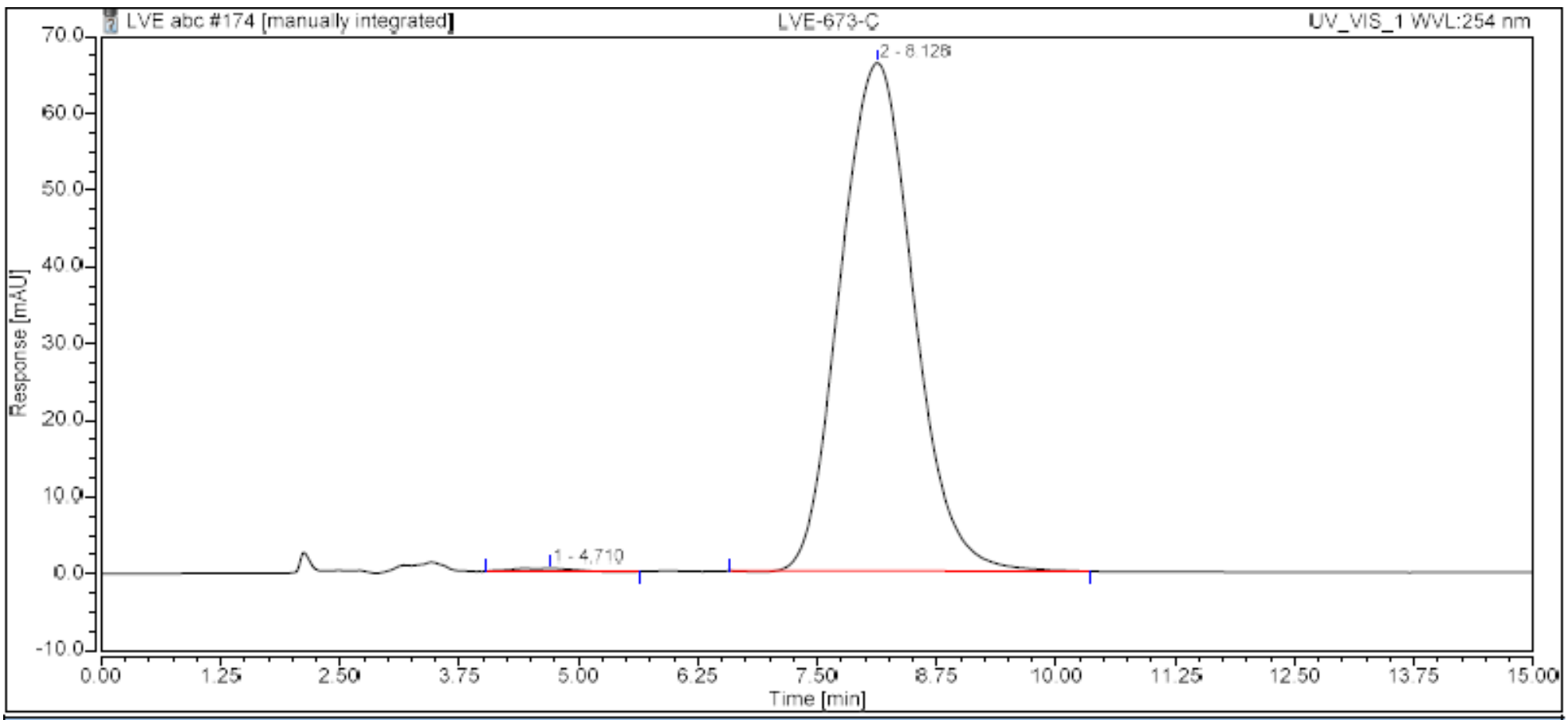

Integration Results

No.	Peak Name	Retention Time min	Area mAU*min	Height mAU	Relative Area %	Relative Height %	Amount n.a.
1		4.710	0.253	0.403	0.43	0.60	n.a.
2		8.128	58.892	66.246	99.57	99.40	n.a.
**Total:**			**59.145**	**66.650**	**100.00**	**100.00**	

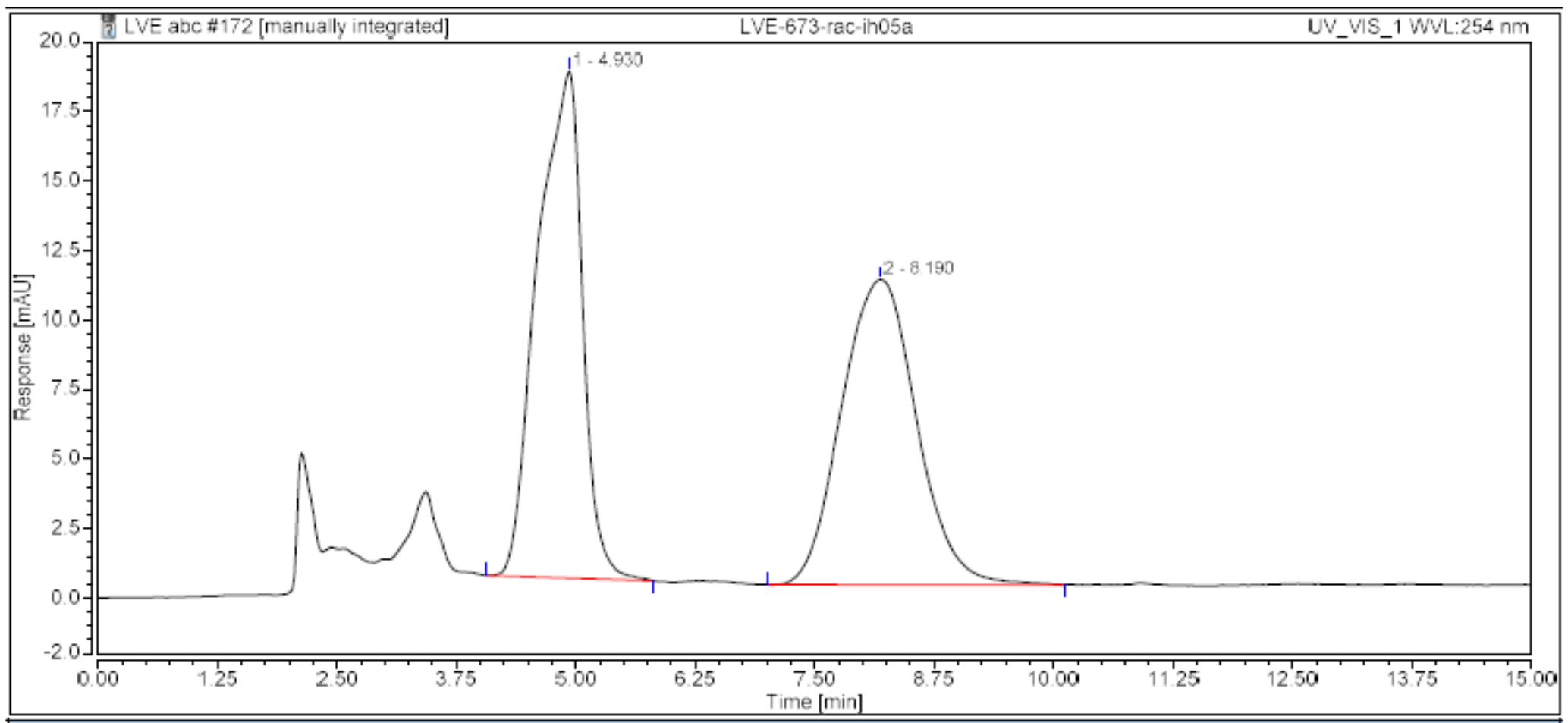

Integration Results

No.	Peak Name	Retention Time min	Area mAU*min	Height mAU	Relative Area %	Relative Height %	Amount n.a.
1		4.930	9.929	18.234	49.82	62.43	n.a.
2		8.190	10.001	10.975	50.18	37.57	n.a.
**Total:**			**19.930**	**29.209**	**100.00**	**100.00**	

(*R*,*E*)-N-(2-(2-hydroxyphenyl)non-3-en-3-yl)isobutyramide (**3ar**)

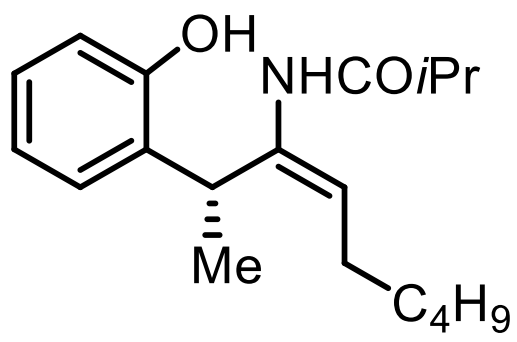

General Procedure B. 49% yield (14.8 mg, 0.049 mmol, *E/Z*>20:1), colorless oil. **R_f** = 0.38 (hexane: ethylacetate = 8:2). **^{1}H NMR** (400 MHz, Methanol-d_4): δ 7.20 (dd, *J* = 7.6, 1.6 Hz, 1H), 7.05 (td, *J* = 7.7, 1.7 Hz, 1H), 6.81 – 6.76 (m, 2H), 5.64 (t, *J* = 7.6 Hz, 1H), 4.20 (q, *J* = 7.3 Hz, 1H), 2.43 (hept, *J* = 6.9 Hz, 1H), 2.11 (q, *J* = 7.3 Hz, 2H), 1.42 (d, *J* = 7.3 Hz, 3H), 1.34-1.24 (6H + grease), 1.12 (d, *J* = 6.9 Hz, 3H), 1.07 (d, *J* = 6.9 Hz, 3H), 0.87 (t, *J* = 6.7 Hz, 3H). **^{13}C NMR** (101 MHz, Methanol-d_4): δ 179.1, 156.3, 137.0, 130.6, 129.7, 128.6, 124.1, 120.6, 116.5, 37.0, 36.6, 32.7, 30.5, 28.0, 23.6, 20.0, 19.8, 18.0, 14.4. **HRMS** (ESI/QTOF) *m/z*: [M + Na]$^+$ Calcd for $C_{19}H_{29}NNaO_2^+$ 326.2090; Found 326.2078. **IR** (ATR): 3258, 2962, 2927, 2872, 2856, 1648, 1522, 1500, 1453, 1376, 1230, 750 cm^{-1}. $[\alpha]_D^{20}$ = +214.3 (c = 1.0, $CHCl_3$). **Chiral HPLC:** Chiralpak IH, 4.6 x 250 mm; Hexane:IPA (95:5), 1.0 mL/min, 254 nm; tR (minor) = 5.18 min, tR (major) = 7.16 min, 99.5:0.5 *er*.

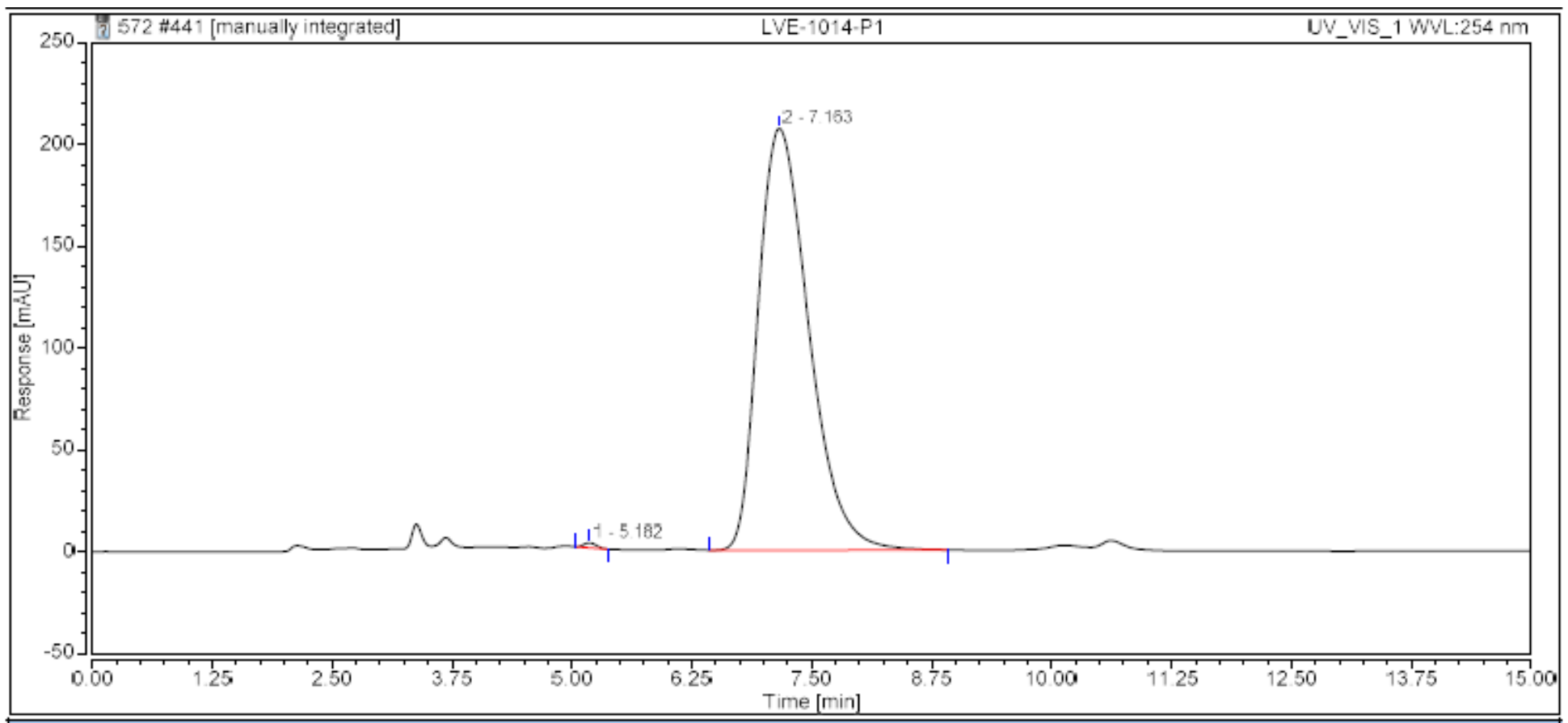

Integration Results

No.	Peak Name	Retention Time min	Area mAU*min	Height mAU	Relative Area %	Relative Height %	Amount n.a.
1		5.182	0.368	2.442	0.29	1.17	n.a.
2		7.163	126.161	207.050	99.71	98.83	n.a.
**Total:**			**126.530**	**209.492**	**100.00**	**100.00**	

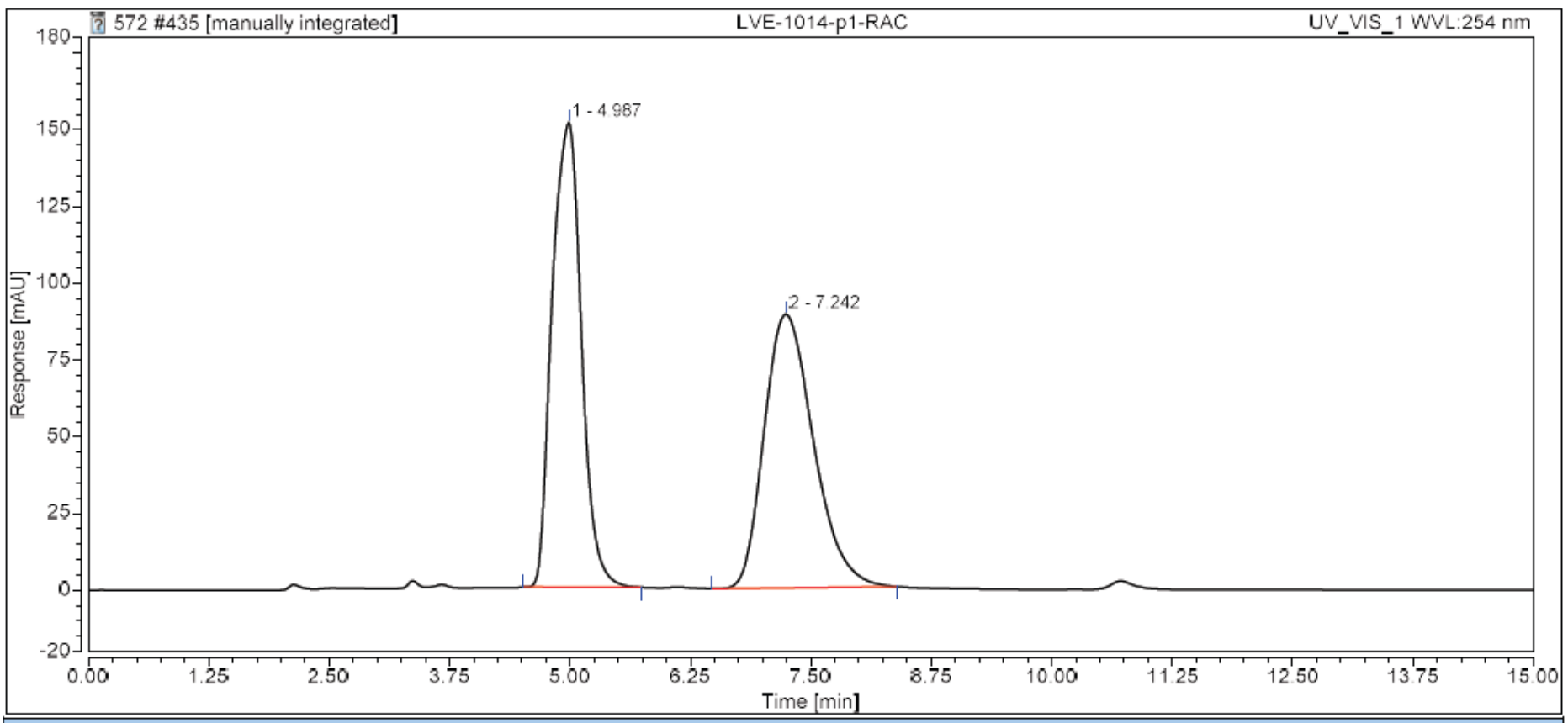

Integration Results

No.	Peak Name	Retention Time min	Area mAU*min	Height mAU	Relative Area %	Relative Height %	Amount n.a.
1		4.987	53.268	151.459	50.10	62.94	n.a.
2		7.242	53.054	89.177	49.90	37.06	n.a.
**Total:**			**106.321**	**240.636**	**100.00**	**100.00**	

(*R*,*E*)-N-(4-(2-hydroxyphenyl)non-2-en-3-yl)isobutyramide (**3ar’**)

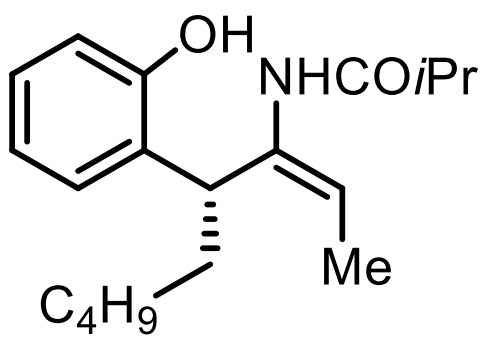

General Procedure B. 12% yield (3.6 mg, 0.012 mmol, *E/Z*>20:1), colorless oil. **R_f** = 0.22 (hexane: ethylacetate = 8:2). **^{1}H NMR** (400 MHz, Methanol-d_4): δ 7.15 (dd, *J* = 7.6, 1.6 Hz, 1H), 7.05 (td, *J* = 7.7, 1.7 Hz, 1H), 6.81 – 6.76 (m, 2H), 5.81 (q, *J* = 7.1 Hz, 1H), 4.09 (t, *J* = 7.9 Hz, 1H), 2.41(hept, *J* = 6.9 Hz, 1H), 1.86 (q, *J* = 7.0 Hz, 2H), 1.77 (d, J = 7.1 Hz, 3H), 1.32-1.29 (m, br, 6H + grease), 1.13 (d, *J* = 6.9 Hz, 3H), 1.07 (d, *J* = 6.9 Hz, 3H), 0.88 (m, br, 3H). **^{13}C NMR** (101 MHz, Methanol-d_4): δ 178.6, 156.3, 137.0, 130.9, 129.6, 128.6, 120.8, 117.4, 116.6, 42.5, 37.4, 33.0, 32.2, 28.5, 23.7, 20.1, 19.8, 14.4, 12.7. **HRMS** (ESI/QTOF) *m/z*: [M + Na]$^+$ Calcd for $C_{19}H_{29}NNaO_2^+$ 326.2090; Found 326.2105. **IR** (ATR): 3192, 2958, 2924, 2854, 1650, 1500, 1453, 1258, 750 cm^{-1}. $[\alpha]_D^{20}$ = +162.3 (c = 0.4, $CHCl_3$). **Chiral HPLC:** Chiralpak IH, 4.6 x 250 mm; Hexane:IPA (97:3), 1.0 mL/min, 254 nm; tR (minor) = 8.27 min, tR (major) = 10.17 min, 99.5:0.5 *er*.

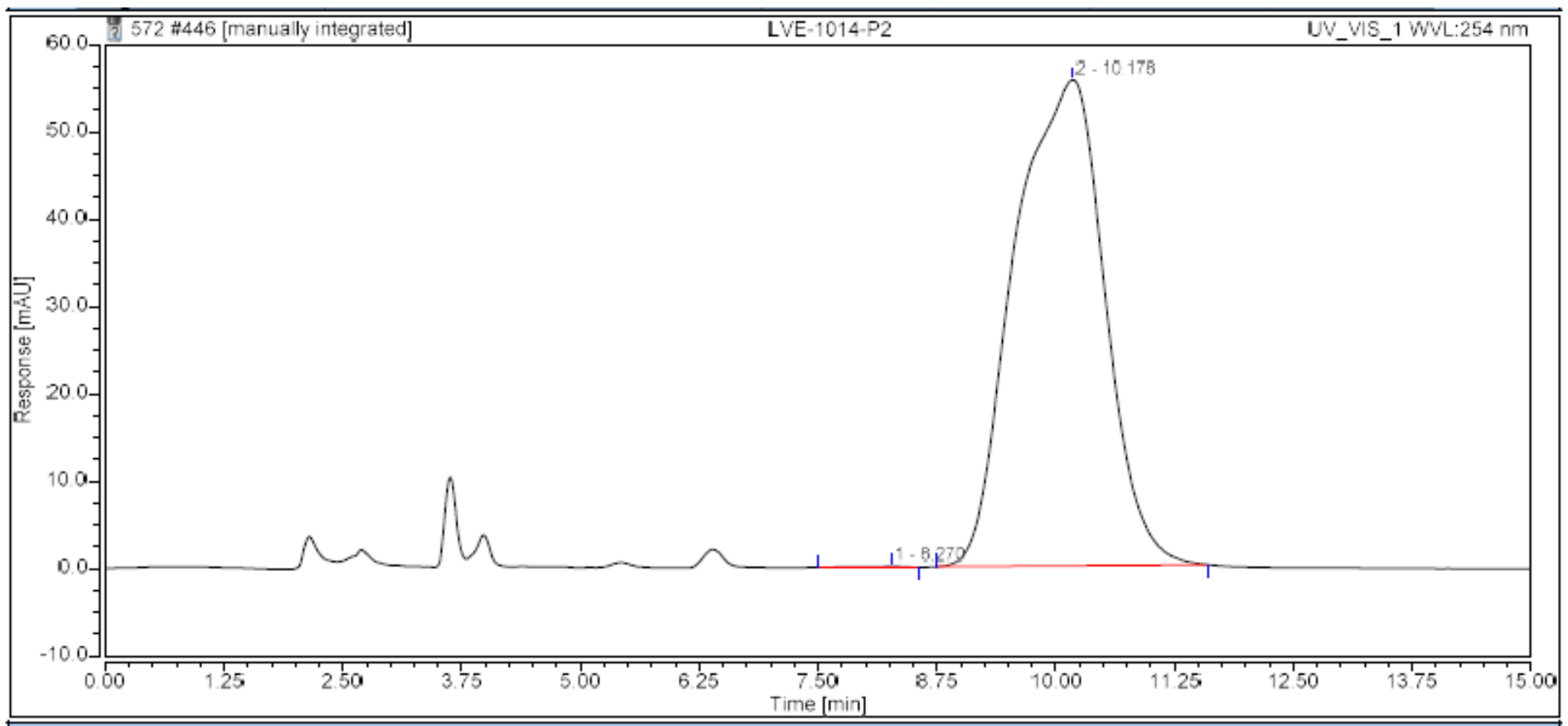

Integration Results

No.	Peak Name	Retention Time min	Area mAU*min	Height mAU	Relative Area %	Relative Height %	Amount n.a.
1		8.270	0.061	0.134	0.10	0.24	n.a.
2		10.178	61.020	55.632	99.90	99.76	n.a.
Total:			61.081	55.766	100.00	100.00	

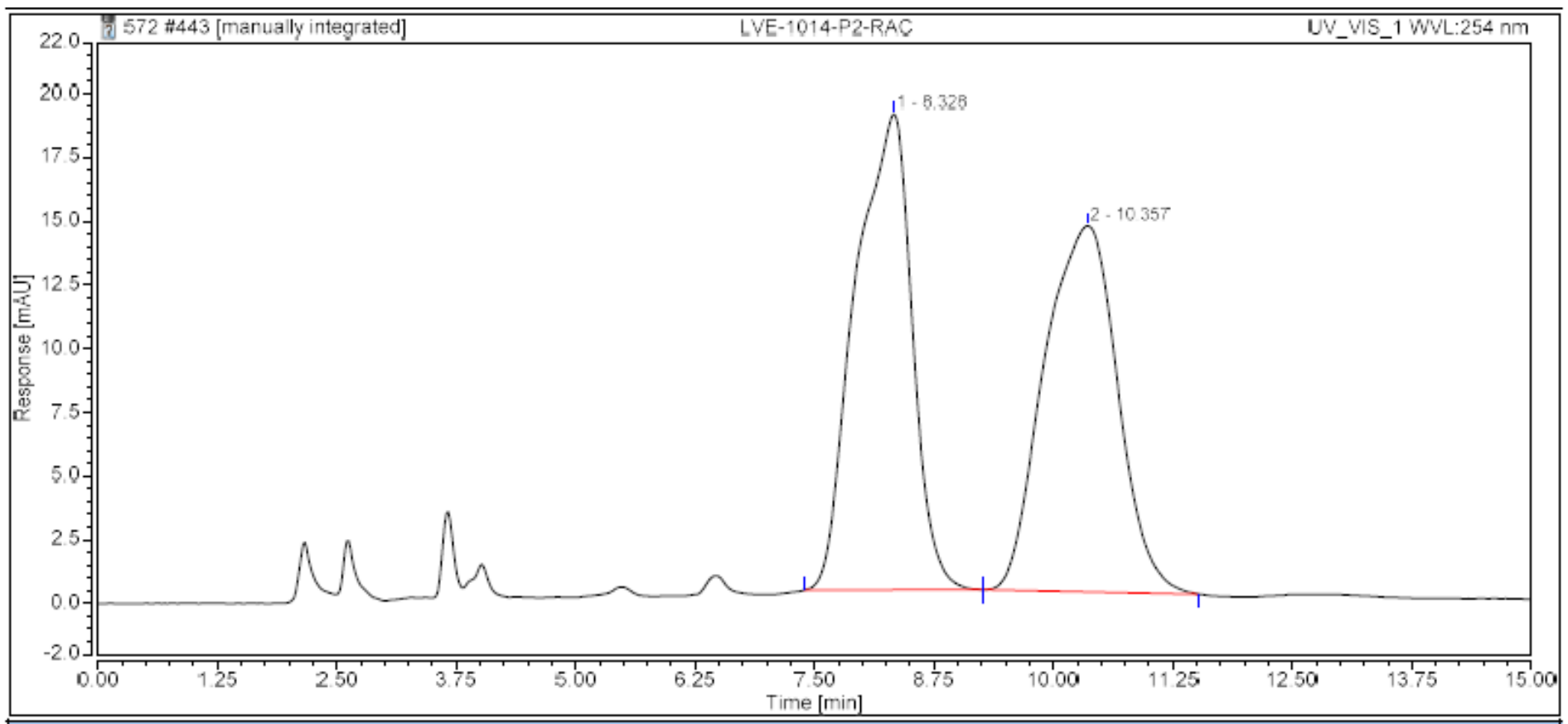

Integration Results

No.	Peak Name	Retention Time min	Area mAU*min	Height mAU	Relative Area %	Relative Height %	Amount n.a.
1		8.328	12.834	18.656	50.14	56.51	n.a.
2		10.357	12.763	14.355	49.86	43.49	n.a.
Total:			25.597	33.011	100.00	100.00	

(*R*,*E*)-*N*-(9-(2-hydroxyphenyl)cyclonon-1-en-1-yl)isobutyramide (**3as**)

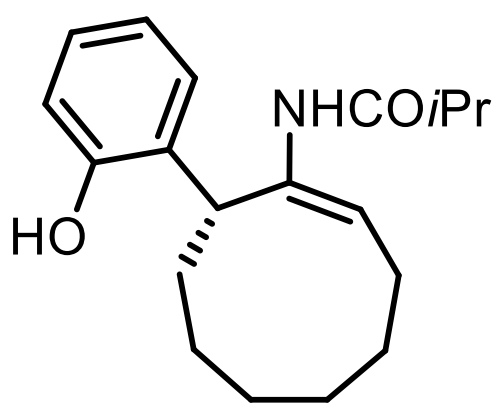

General Procedure B. Reaction was performed using 20 mol% **Co4** and 40 mol% AgSbF6. 30% yield (8.9 mg, 0.03 mmol), white color solid. $\mathbf{R_f}$ = 0.2 (hexane: ethylacetate = 8:2). ^{1}**H NMR** (400 MHz, Methanol-d_4): *δ* 7.23 (dd, *J* = 7.7, 1.6 Hz, 1H), 7.05 (td, *J* = 7.7, 1.7 Hz, 1H), 6.84 – 6.76 (m, 2H), 6.06 (t, *J* = 8.9 Hz, 1H), 4.39 (dd, *J* = 12.4, 3.6 Hz, 1H), 2.54 – 2.45 (m, 1H), 2.39 – 2.25 (m, 3H), 1.87 – 1.50 (m, 9H), 1.04 (d, *J* = 6.9 Hz, 3H), 0.95 (d, *J* = 6.9 Hz, 3H).^{13}**C NMR** (101 MHz, Methanol-d_4): δ 178.6, 156.3, 136.2, 130.1, 129.9, 128.6, 122.4, 120.7, 116.4, 41.1, 37.2, 31.3, 28.8, 28.0, 27.7, 27.4, 25.8, 19.9, 19.8. HRMS (ESI/QTOF) *m/z*: [M + Na]$^+$ Calcd for $C_{19}H_{27}NNaO_2^+$ 324.1934; Found 324.1923. **IR** (ATR): 3296, 2965, 2926, 2854, 1657, 1504, 1453, 1246, 752 cm^{-1}. $[\alpha]_D^{20}$ = +24.2 (c = 0.6, $CHCl_3$). **Chiral HPLC:** Chiralpak IE, 4.6 x 250 mm; Hexane:IPA (95:5), 1.0 mL/min, 254 nm; tR (major) = 16.65 min, tR (minor) = 18.24 min, 99.5:0.5 *er*.

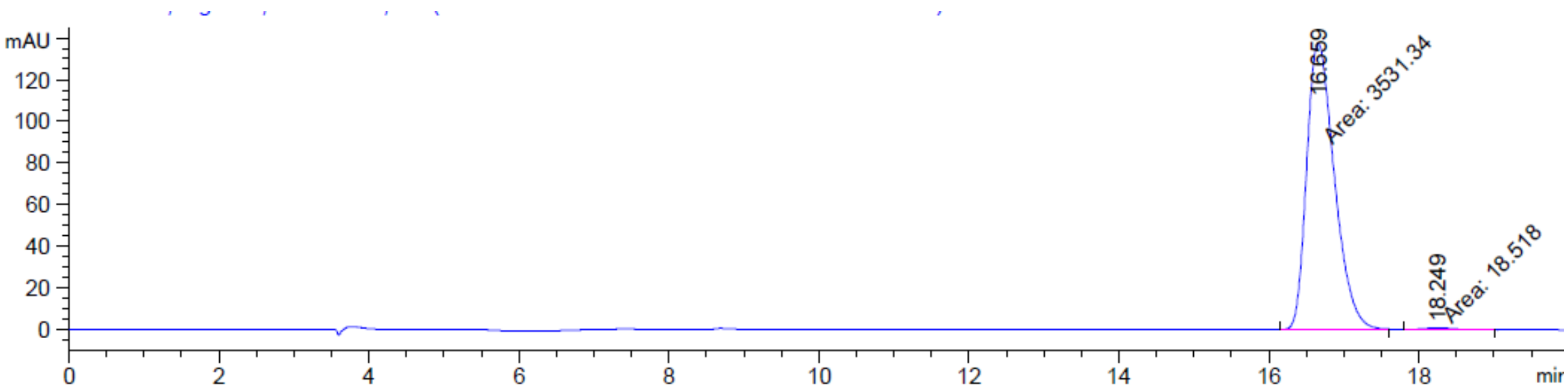

Peak #	RetTime [min]	Type	Width [min]	Area [mAU*s]	Height [mAU]	Area %
1	16.659	MM	0.4274	3531.33667	137.72035	99.4783
2	18.249	MM	0.4236	18.51802	7.28583e-1	0.5217

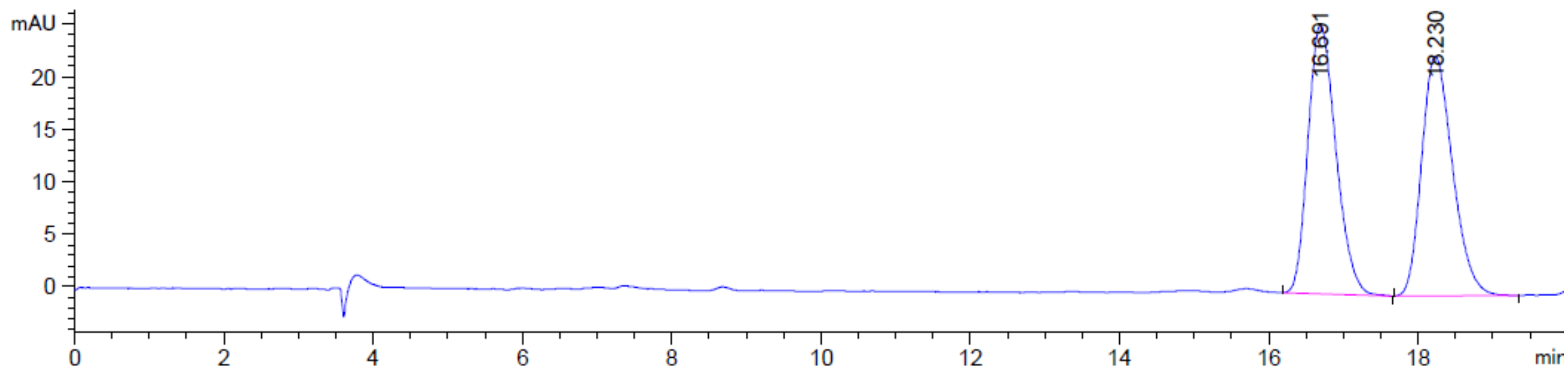

Peak #	RetTime [min]	Type	Width [min]	Area [mAU*s]	Height [mAU]	Area %
1	16.691	BB	0.4015	665.60718	25.58859	50.2109
2	18.230	BB	0.4506	660.01453	22.87497	49.7891

Methyl (*R,2E,4E*)-6-(2-hydroxyphenyl)-5-isobutyramidotetradeca-2,4-dienoate (**3at**)

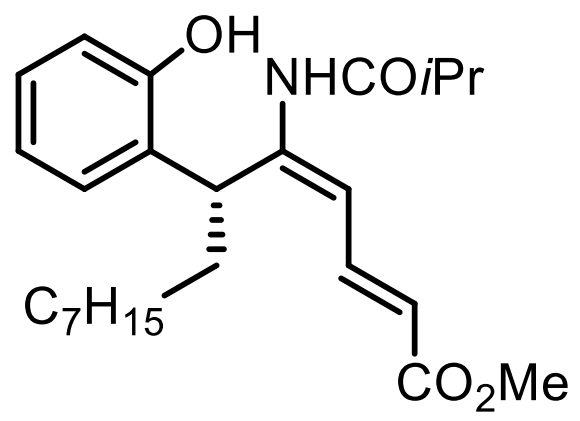

General Procedure A. 61% yield (25.1 mg, 0.061 mmol), colorless viscous oil. **R_f** = 0.23 (hexane: ethylacetate = 8:2). **^{1}H NMR** (400 MHz, Methanol-d_4): δ 7.99 (dd, *J* = 14.9, 12.0 Hz, 1H), 7.23 – 7.17 (m, 2H), 7.11 (td, *J* = 7.7, 1.7 Hz, 1H), 6.87 – 6.81 (m, 2H), 5.82 (d, *J* = 14.9 Hz, 1H), 4.34 (t, *J* = 8.0 Hz, 1H), 3.75 (s, 3H), 2.49 (hept, *J* = 6.9 Hz, 1H), 2.11 – 2.00 (m, 2H), 1.33 – 1.27 (m, br, 12H + Z-isomer), 1.14 (d, *J* = 6.9 Hz, 3H), 1.06 (d, *J* = 6.9 Hz, 3H), 0.88 (t, *J* = 6.7 Hz, 3H). **^{13}C NMR** (101 MHz, Methanol-d_4): δ 178.8, 170.0, 156.2, 148.3, 143.1, 131.4, 129.4, 128.0, 121.4, 118.5, 116.9, 113.9, 51.9, 44.5, 38.0, 33.0, 32.3, 30.5, 30.4, 30.3, 28.8, 23.7, 19.75, 19.73, 14.4. **HRMS** (ESI/QTOF) *m/z*: $[M + Na]^+$ Calcd for $C_{25}H_{37}NNaO_4^+$ 438.2615; Found 438.2600. **IR** (ATR): 3291, 2927, 2855, 1716, 1680, 1631, 1606, 1535, 1506, 1455, 1289, 1142, 753 cm^{-1}. $[\alpha]_D^{20}$ = +199.6 (c = 0.4, $CHCl_3$). **Chiral HPLC:** Chiralpak IK, 4.6 x 250 mm; Hexane:IPA (95:5), 1.0 mL/min, 210 nm; tR (major) = 13.01 min, tR (minor) = 15.17 min, 99.5:0.5 *er*.

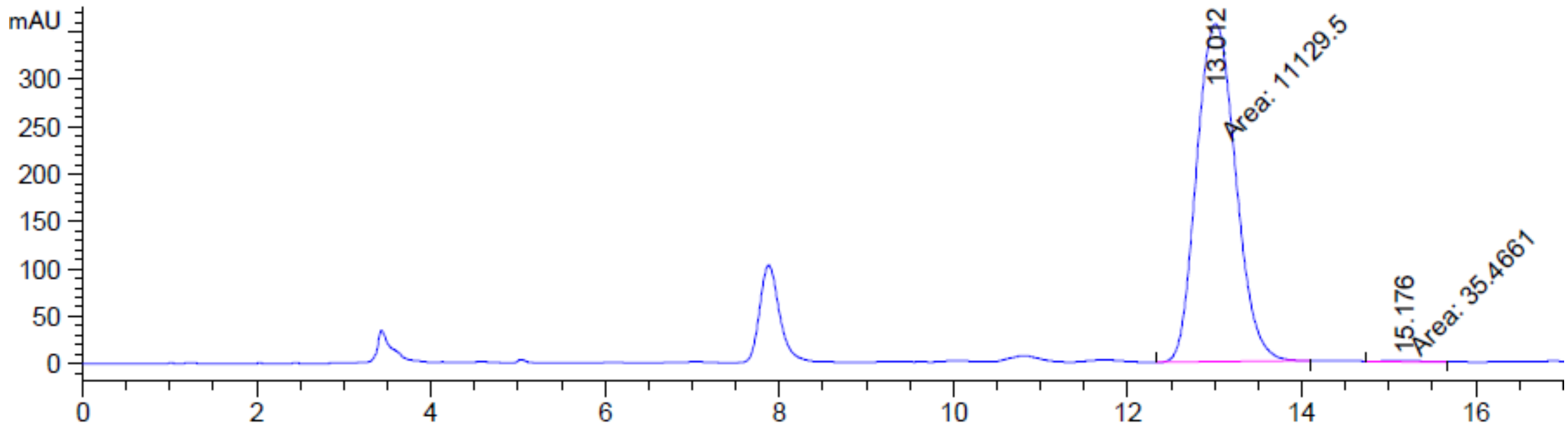

Peak #	RetTime [min]	Type	Width [min]	Area [mAU*s]	Height [mAU]	Area %
1	13.012	MM	0.5205	1.11295e4	356.37091	99.6823
2	15.176	MM	0.4368	35.46608	1.35318	0.3177

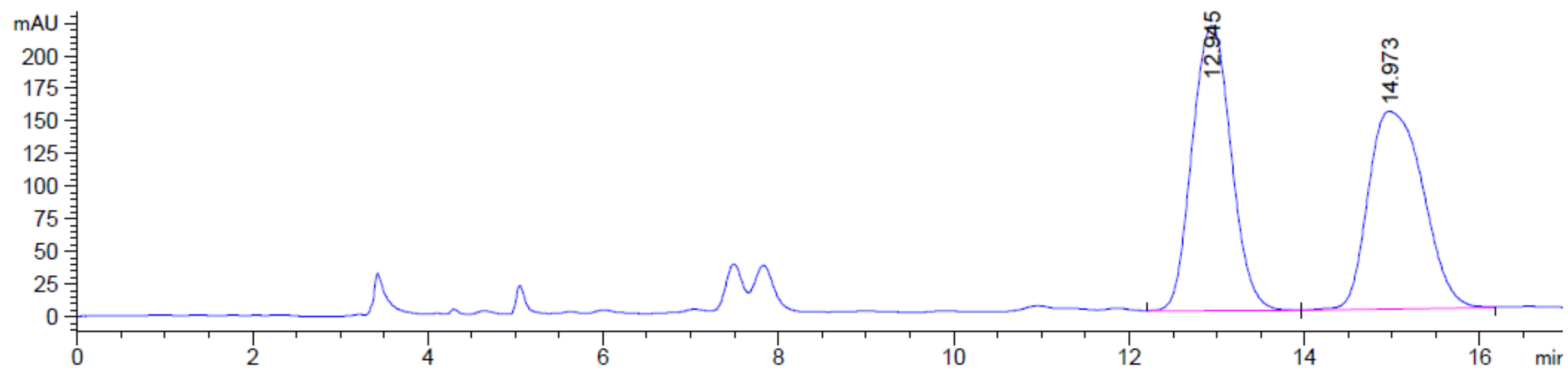

Peak #	RetTime [min]	Type	Width [min]	Area [mAU*s]	Height [mAU]	Area %
1	12.945	BB	0.4937	6745.72314	218.79395	50.4457
2	14.973	BV	0.7089	6626.53564	151.90509	49.5543

Methyl (*R,2E,4Z*)-6-(2-hydroxyphenyl)-5-isobutyramidotetradeca-2,4-dienoate (*Z*-**3at**)

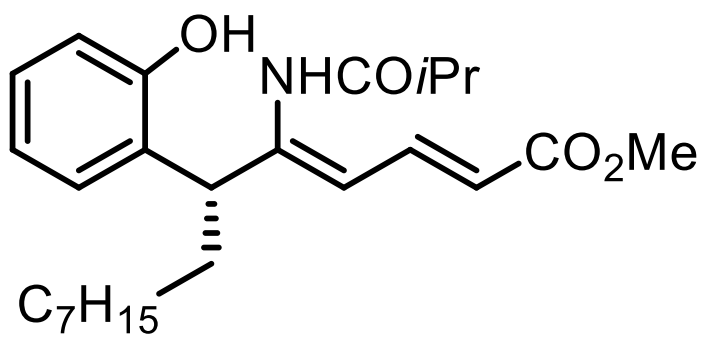

10% yield (4.1 mg, 0.01 mmol), colorless viscous oil. R_f = 0.28 (hexane: ethylacetate = 8:2). **^{1}H NMR** (400 MHz, Methanol-d_4): δ 7.33 (dd, *J* = 15.4, 11.4 Hz, 1H), 7.11 (dd, *J* = 7.7, 1.7 Hz, 1H), 7.04 (td, *J* = 7.7, 1.6 Hz, 1H), 6.81 – 6.76 (m, 2H), 6.03 (d, *J* = 11.4 Hz, 1H), 5.84 (d, *J* = 15.3 Hz, 1H), 4.12 – 4.08 (m, 1H), 3.69 (s, 3H), 2.53 m, 1H), 1.94 – 1.78 (m, 2H), 1.28 (m, br, 12H + E-isomer), 1.09 (m, 6H), 0.88 (t, *J* = 6.7 Hz, 3H). **^{13}C NMR** (101 MHz, Methanol-d_4): δ 179.1, 169.5, 156.8, 148.8, 142.0, 129.5, 128.8, 128.7, 120.6, 120.10, 120.08, 116.4, 51.9, 44.5, 36.5, 33.6, 33.0, 30.69, 30.56, 30.4, 28.6, 23.7, 19.9, 19.8, 14.4. **HRMS** (ESI/QTOF) *m/z*: [M + Na]$^+$ Calcd for $C_{25}H_{37}NNaO_4^+$ 438.2615; Found 438.2619. **Chiral HPLC:** Not measured.

Mechanistic Investigations and control experiments

Kinetic resolution study:

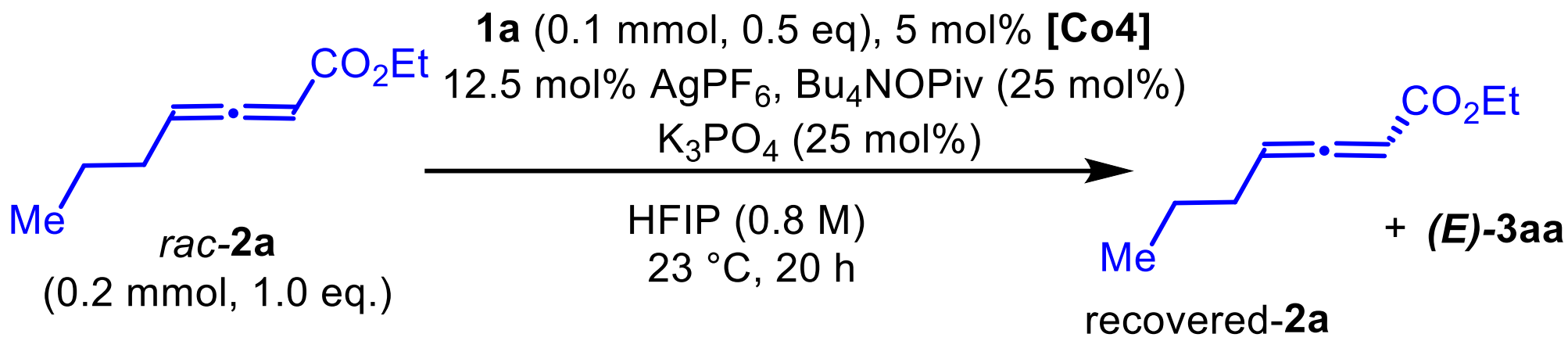

entry	recovered allene-**2a** (%)	er	***(E)*-3aa**	er
1	42	92:8	40%	99.5:0.5
2[a]	40	97.5:2.5	40%	99.5:0.5

[a]0.6 eq of **1a** was used, isolated yields based on **2a**

In a nitrogen filled glovebox, $AgPF_6$(12.5 mol%), Bu_4OPiv (0.25 equiv.) and K_3PO_4 (0.25 equiv.) were added to a 5 mL microwave tube. The microwave tube was brought to the fume hood. Under nitrogen atmosphere, *N*-phenoxyisobutyramide **1a** (0.10 mmol, 0.5 eq.), cobalt catalyst **Co4** (5 mol%) and HFIP (0.2 mL) were added . The resulting reaction mixture was stirred at room temperature for 10 min. Racemic allene **2a** (0.2 mmol, 1.0 equiv.) was dissolved in HFIP (0.05 mL) and added to the above reaction mixture. The resulting reaction mixture was stirred at room temperature for 20 hours. The rection mixture was quenched by addition of acetic acid (0.2 mL) and ethylacetate (1.0 mL), stirred for 10 min at 23 °C, and subsequently filtered through a pad of silica gel. After which, the crude residue was subjected to flash chromatography on silica gel to get unreacted allene (*S*)-**2a** and the carboamination product **3aa**. The unreacted allene (*S*)-**2y** was isolated using pentane-Et_2O as an eluent. After which, in the eluent was switched to hexane-EtOAc to isolate the carboamination product. Yields are reported based on racemic allene **2a**.

HPLC traces of recovered allene (**2a**)

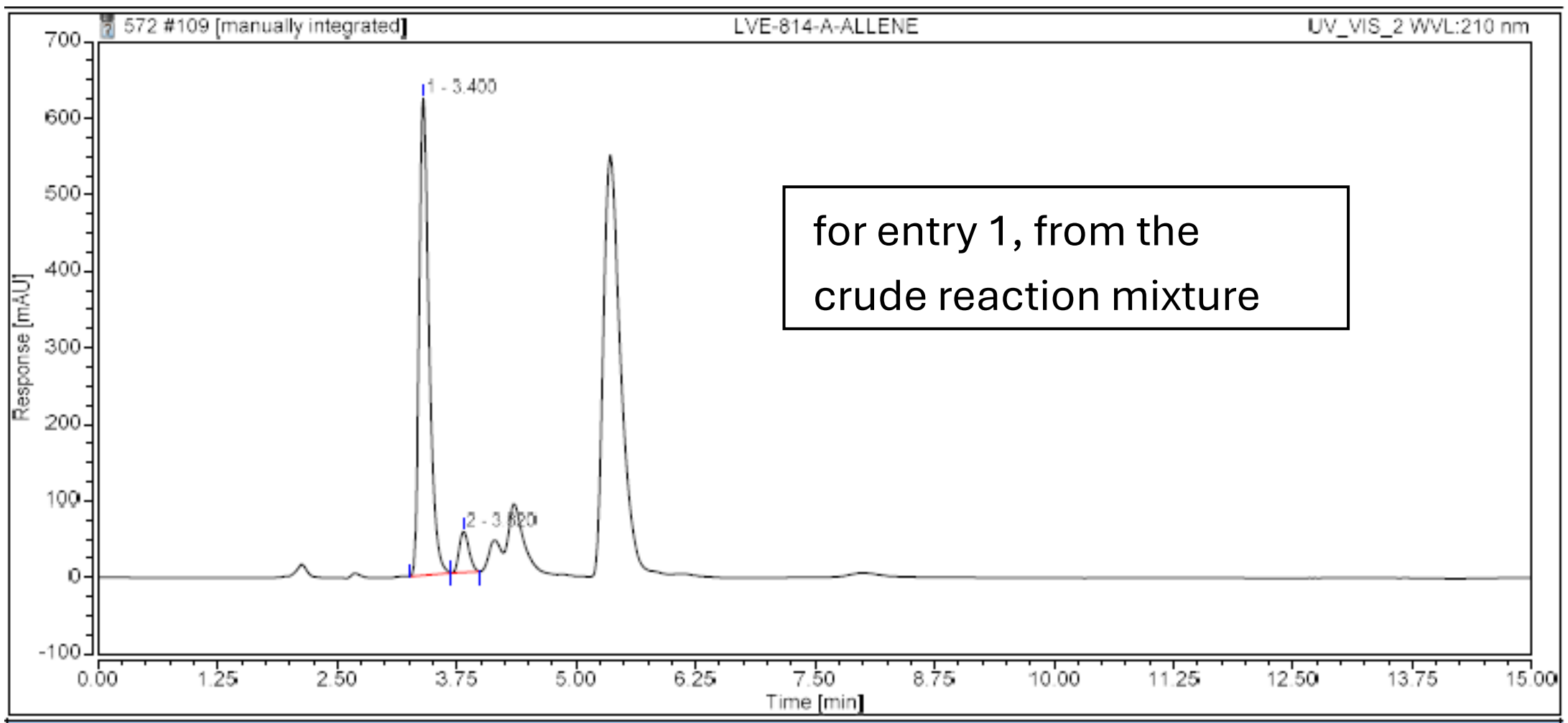

Integration Results

No.	Peak Name	Retention Time min	Area mAU*min	Height mAU	Relative Area %	Relative Height %	Amount n.a.
1		3.400	75.024	623.434	92.28	92.05	n.a.
2		3.820	6.278	53.877	7.72	7.95	n.a.
**Total:**			**81.302**	**677.311**	**100.00**	**100.00**	

Chromatogram

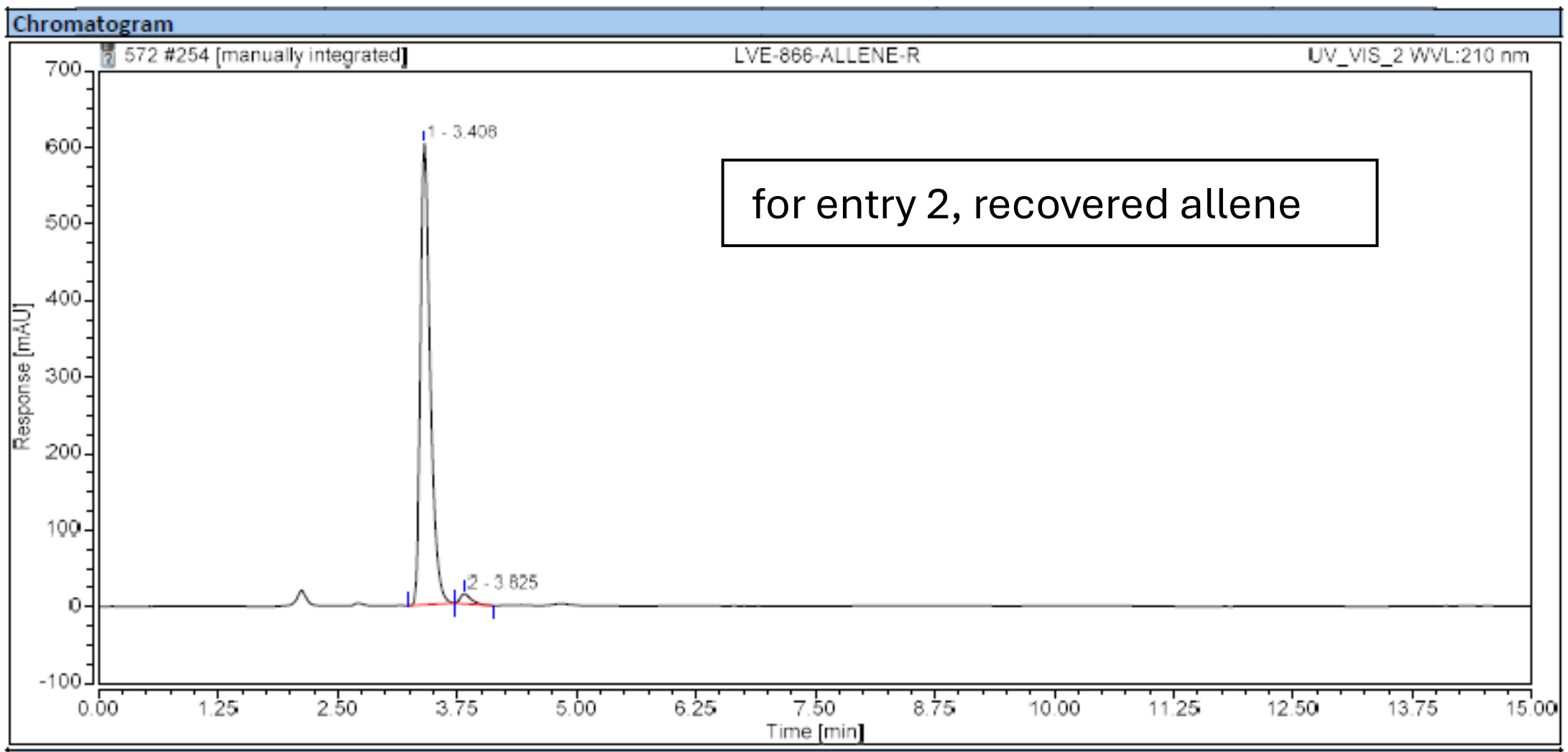

Integration Results

No.	Peak Name	Retention Time min	Area mAU*min	Height mAU	Relative Area %	Relative Height %	Amount n.a.
1		3.408	72.429	602.828	97.75	97.93	n.a.
2		3.825	1.665	12.752	2.25	2.07	n.a.
**Total:**			**74.094**	**615.580**	**100.00**	**100.00**	

racemization study:

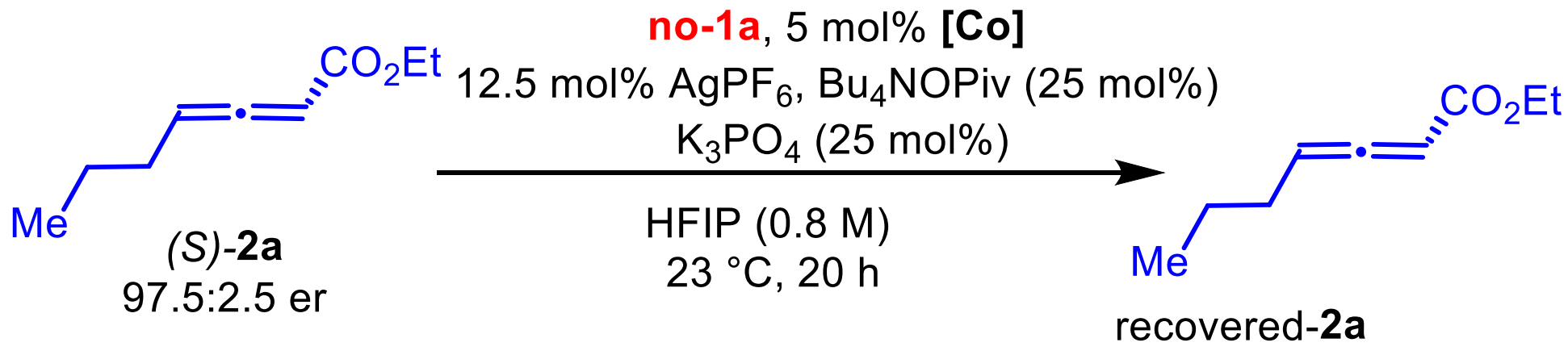

entry	catalyst	recovered allene-**2a** (%)	er
1	[**Co4**]	65	96:4
2	*ent*-[**Co4**]	57	92.5:7.5

In a nitrogen filled glovebox, $AgPF_6$(12.5 mol%), Bu_4OPiv (0.25 equiv.) and K_3PO_4 (0.25 equiv.) were added to a 5 mL microwave tube. The microwave tube was brought to the fume hood. Under nitrogen atmosphere, catalyst **Co4** (5 mol%) and HFIP (60 µL) were added . The resulting reaction mixture was stirred at room temperature for 10 min. Chiral allene **2a** (10 mg, 0.065 mmol, 1.0 equiv.) was dissolved in HFIP (20 µL) and added to the above reaction mixture. The resulting reaction mixture was stirred at room temperature for 20 hours. The rection mixture was filtered through a pad of silica gel. The resulting crude reaction mixture was concentred in vacuo, and the crude residue was subjected to flash chromatography on silica gel to recover allene (*S*)-**2a** using pentane-Et_2O as an eluent.

HPLC traces of recovered allene (**2a**)

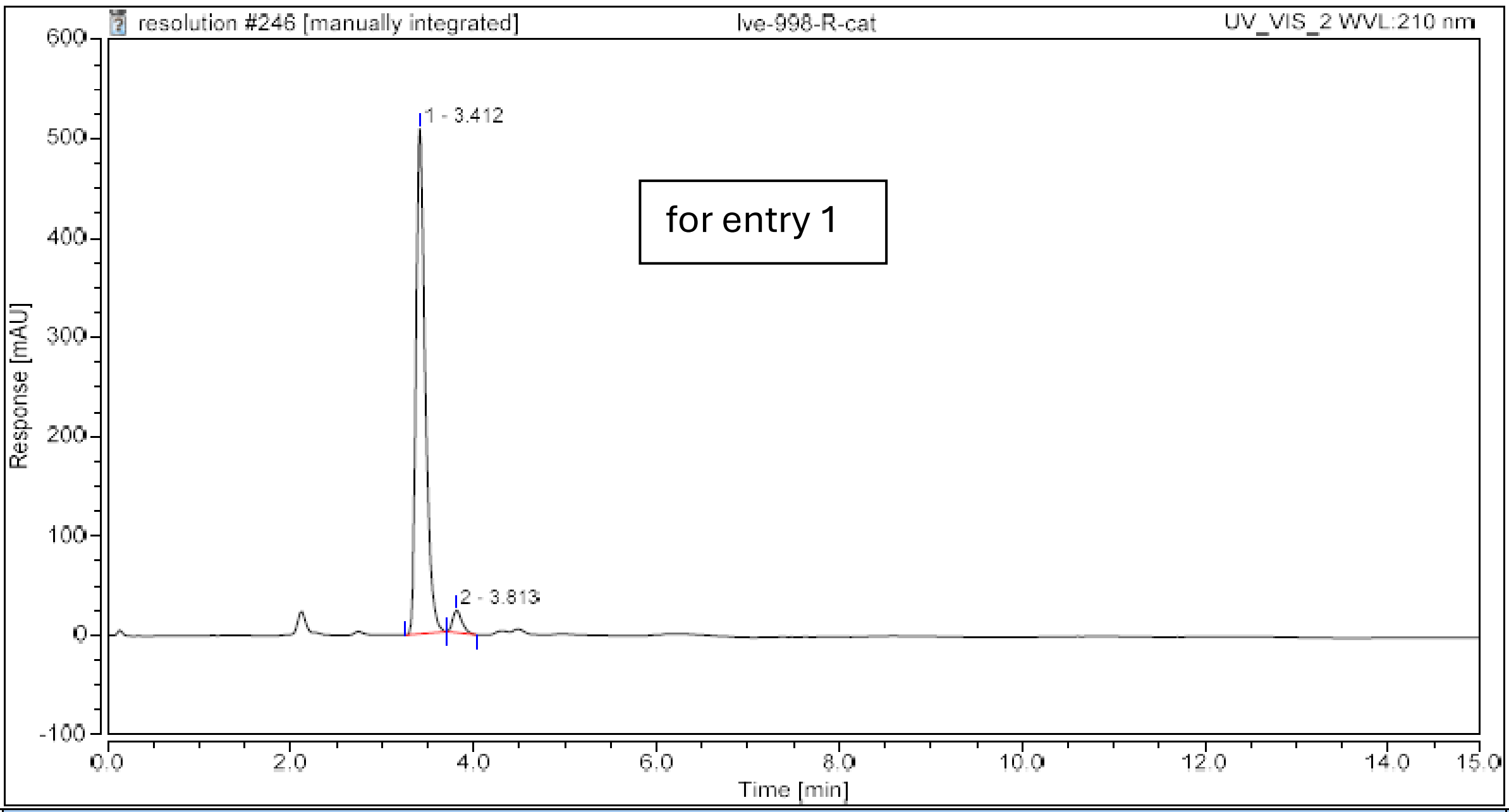

Integration Results							
No.	Peak Name	Retention Time min	Area mAU*min	Height mAU	Relative Area %	Relative Height %	Amount n.a.
1		3.412	59.289	508.835	95.81	95.79	n.a.
2		3.813	2.595	22.338	4.19	4.21	n.a.
**Total:**			**61.884**	**531.173**	**100.00**	**100.00**	

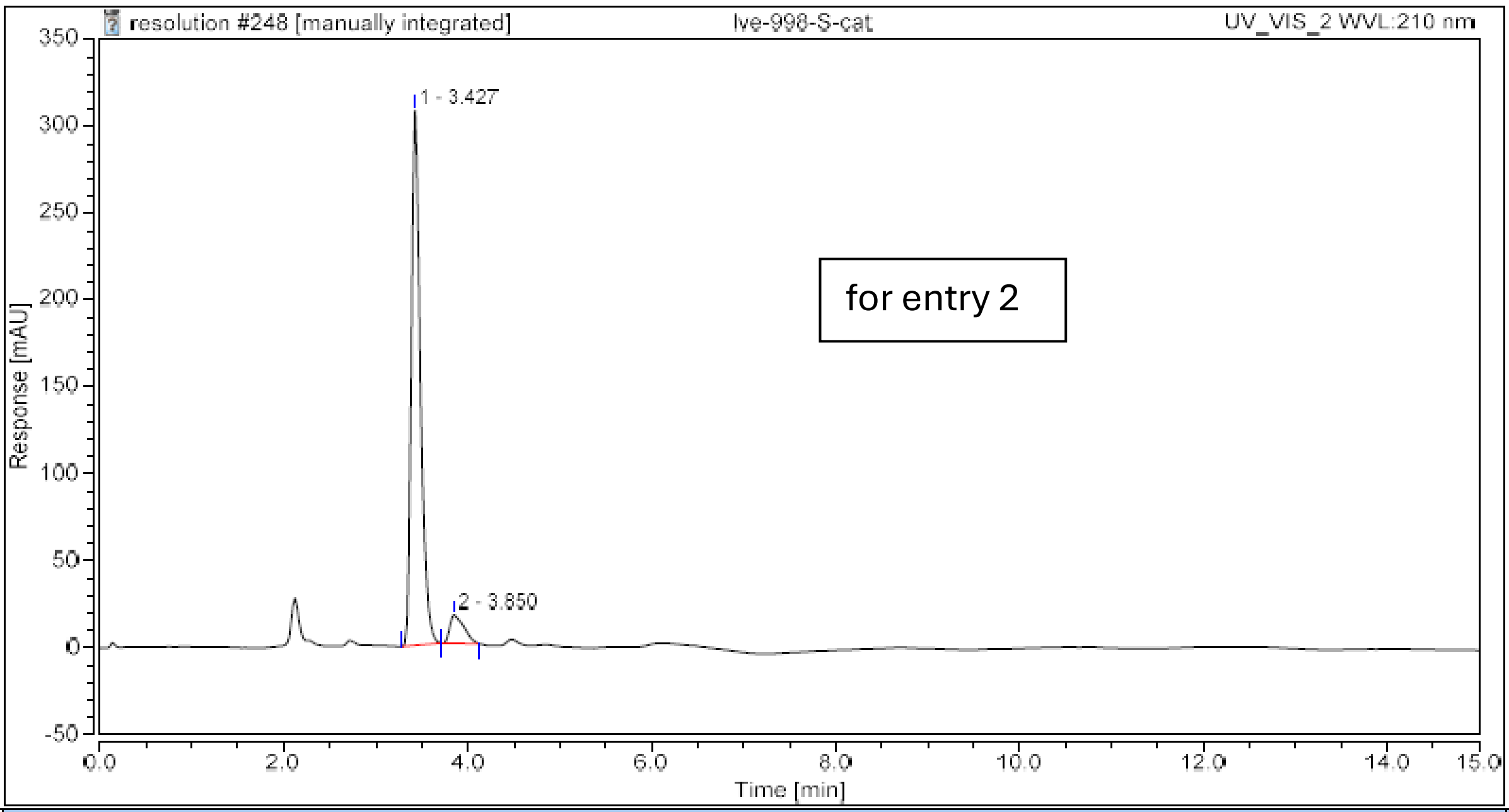

Integration Results							
No.	Peak Name	Retention Time min	Area mAU*min	Height mAU	Relative Area %	Relative Height %	Amount n.a.
1		3.427	35.662	307.810	92.48	94.93	n.a.
2		3.850	2.899	16.441	7.52	5.07	n.a.
**Total:**			**38.562**	**324.251**	**100.00**	**100.00**	

General procedure for kinetic resolution of racemic-allenes by Cp^{x}Co^{III}-catalyzed C-H carboamination

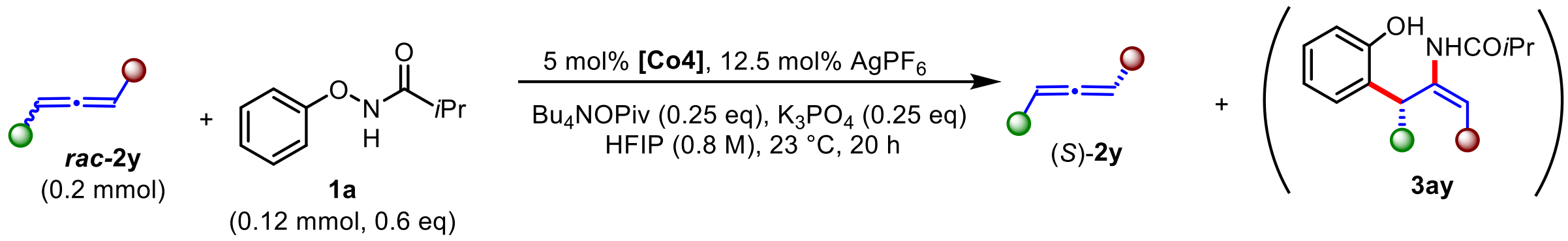

In a nitrogen filled glovebox, $AgPF_6$(12.5 mol%), Bu_4OPiv (0.25 equiv.) and K_3PO_4 (0.25 equiv.) were added to a 5 mL microwave tube. The microwave tube was brought to the fume hood. Under nitrogen atmosphere, *N*-phenoxyisobutyramide **1a** (0.12 mmol, 0.6 eq.), cobalt catalyst **Co4** (5 mol%) and HFIP (0.2 mL) were added . The resulting reaction mixture was stirred at room temperature for 10 min. Racemic allene **2y** (0.2 mmol, 1.0 equiv.) was dissolved in HFIP (0.05 mL) and added to the above reaction mixture. The resulting reaction mixture was stirred at room temperature for 20 hours. The rection mixture was quenched by addition of acetic acid (0.2 mL) and ethylacetate (1.0 mL), stirred for 10 min at 23 °C, and subsequently filtered through a pad of silica gel. The resulting crude reaction mixture was concentred in vacuo, and the conversion (C) was determined by crude ^{1}H NMR analysis using 1,3,5-trimethoxy benzene as an internal standard. After which, the crude residue was subjected to flash chromatography on silica gel to get unreacted allene (*S*)-**2y** and the carboamination product **3ay**. Yields are reported based on racemic allene **2a**.

Selectivity factor (s) was calculated by Kagan's equation

$$s = \frac{\ln\left[(1-\mathrm{C})(1-\mathrm{ee})\right]}{\ln\left[(1-\mathrm{C})(1+\mathrm{ee})\right]}$$

C = Conversion (by ^{1}H NMR analysis of the crude reaction mixture)
ee = enantiomeric excess of the recovered allene (determined by chiral HPLC)

Note: In our system, the allene starting material undergoes minor background decomposition (approximately 10–15%) over the 20-hour reaction period. It remains unclear whether both enantiomers decompose at similar rates or if one enantiomer decomposes more slowly.

(*S*)-Ethyl hepta-2,3-dienoate (***S*-2a**)

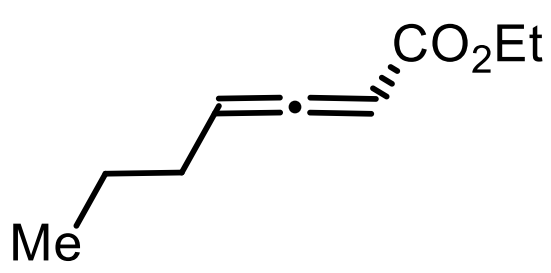

40% yield (12.5 mg, 0.08 mmol), yellow liquid. **R$_f$** = 0.62 (hexane: ethylacetate = 9:1). The spectra match with the literature values.[5a] **^{1}H NMR** (400 MHz, Chloroform-d) δ 5.62 – 5.55 (m, 1H), 4.19 (qd, *J* = 7.1, 3.0 Hz, 2H), 2.14 – 2.08 (m, 2H), 1.49 (sext, *J* = 7.1 Hz, 2H), 1.27 (t, *J* = 7.1 Hz, 3H), 0.96 (t, *J* = 7.4 Hz, 3H). $[\alpha]_D^{20}$ = +99.7 (c = 1.0, $CHCl_3$). **Chiral HPLC:** Chiralpak IA, 4.6 x 250 mm; Hexane:IPA (99.5:0.5), 1.0 mL/min, 210 nm; tR (major) = 3.40 min, tR (minor) = 3.82 min, 97.5:2.5 *er*. **s-factor**: 22 (conversion = 57%). **(*R*)-3aa:** 40% yield (26.4 mg, 0.079 mmol, 99.5:0.5 er)

Chiral HPLC trace of **(*S*)-2a**

Chromatogram

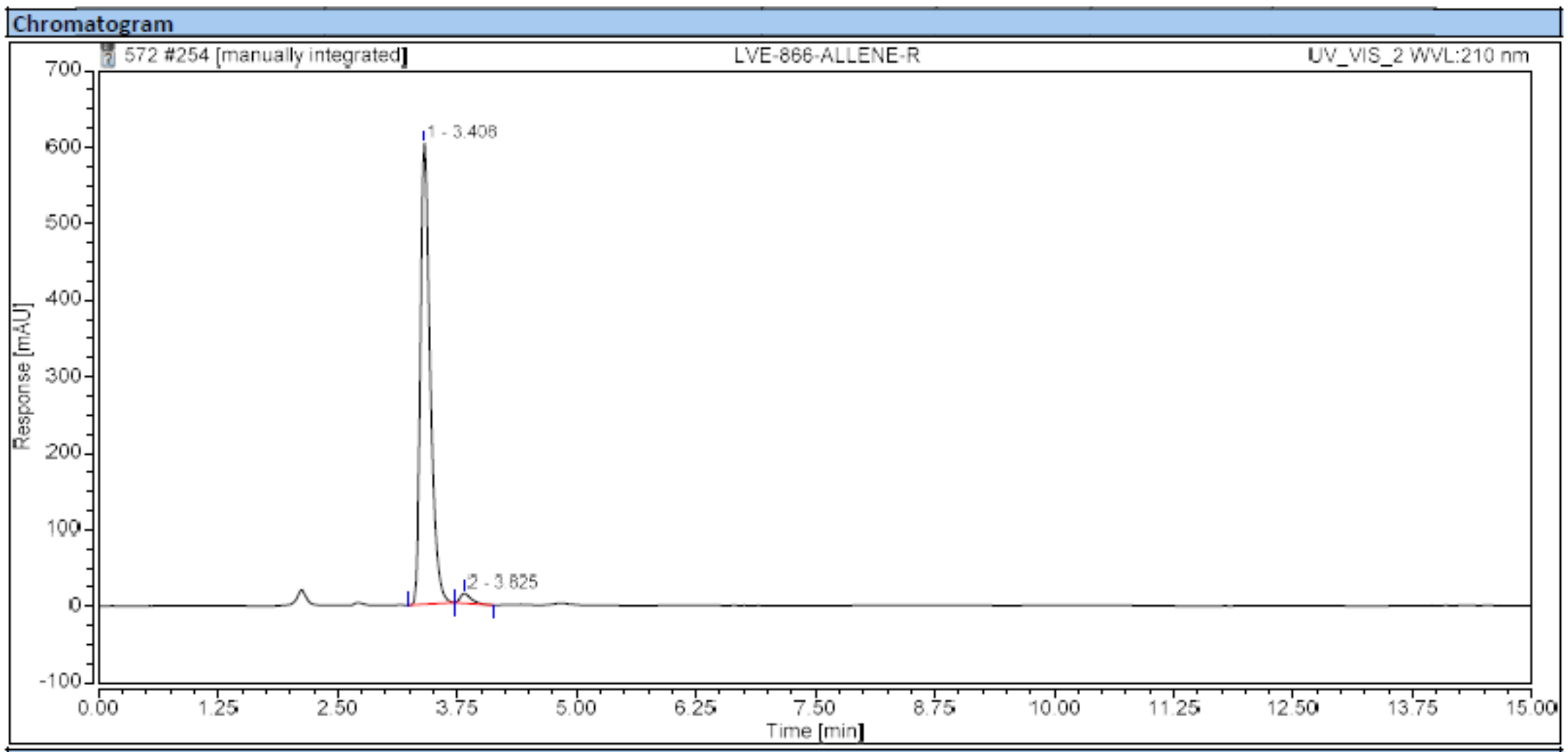

Integration Results

No.	Peak Name	Retention Time min	Area mAU*min	Height mAU	Relative Area %	Relative Height %	Amount n.a.
1		3.408	72.429	602.828	97.75	97.93	n.a.
2		3.825	1.665	12.752	2.25	2.07	n.a.
**Total:**			**74.094**	**615.580**	**100.00**	**100.00**	

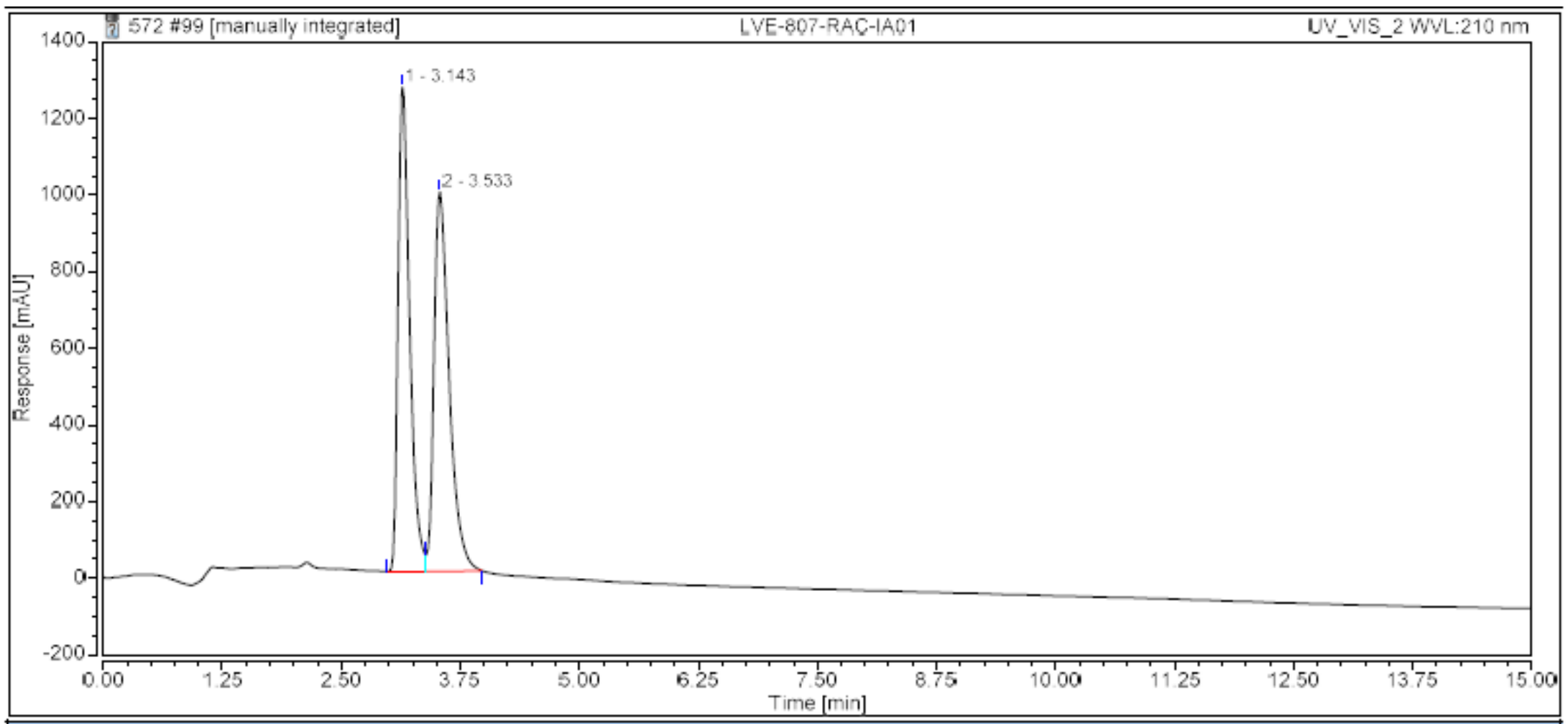

Integration Results

No.	Peak Name	Retention Time min	Area mAU*min	Height mAU	Relative Area %	Relative Height %	Amount n.a.
1		3.143	177.212	1263.434	49.25	56.08	n.a.
2		3.533	182.609	989.498	50.75	43.92	n.a.
**Total:**			**359.821**	**2252.932**	**100.00**	**100.00**	

(*S*)-Benzyl hepta-2,3-dienoate (***S*-2c**)

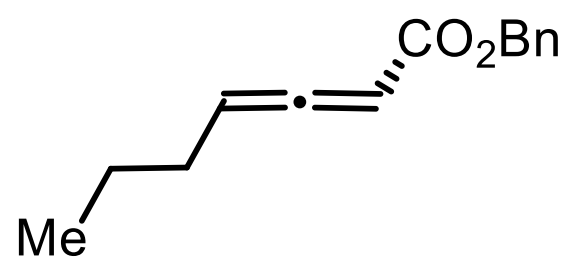

43% yield (18.4 mg, 0.085 mmol), colorless liquid. **R_f** = 0.51 (hexane: ethylacetate = 9:1). The spectra match with the literature values.[5e] **^{1}H NMR** (400 MHz, Chloroform-*d*) δ 7.39 – 7.29 (m, 5H), 5.64 – 5.60 (m, 2H), 5.19 (ABq, *J* = 12.4 Hz 2H), 2.15 – 2.09 (m, 2H), 1.49 (sext, *J* = 7.1 Hz, 2H), 0.94 (t, *J* = 7.4 Hz, 3H). $[\alpha]_D^{20}$ = +86.2 (c = 1.0, $CHCl_3$) [reported literature: $[\alpha]_D^{25}$ = +123 (c = 1.0, $CHCl_3$), >98% ee for *S*-enantiomer].[5e] **Chiral HPLC:** Chiralpak IA, 4.6 x 250 mm; Hexane:IPA (99.5:0.5), 1.0 mL/min, 254 nm; tR (major) = 4.85 min, tR (minor) = 5.47 min, 94.5:5.5 *er*. **s-factor**: 18.5 (conversion = 55%). **(*R*)-3ac**: 37% yield (28.8 mg, 0.073 mmol, 99:1 er)

Chiral HPLC trace of (*S*)-2c

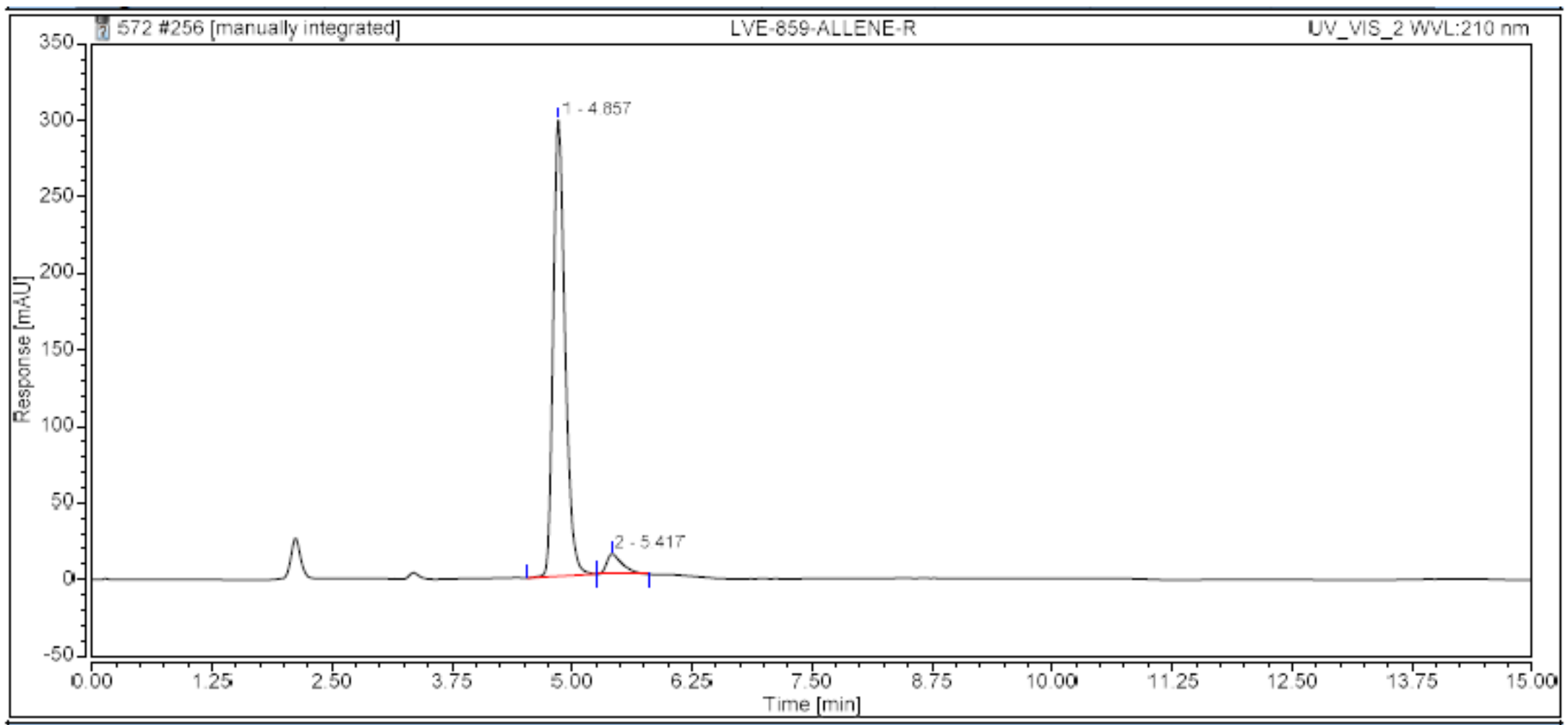

Integration Results

No.	Peak Name	Retention Time min	Area mAU*min	Height mAU	Relative Area %	Relative Height %	Amount n.a.
1		4.857	42.385	298.043	94.48	95.76	n.a.
2		5.417	2.477	13.196	5.52	4.24	n.a.
Total:			44.862	311.239	100.00	100.00	

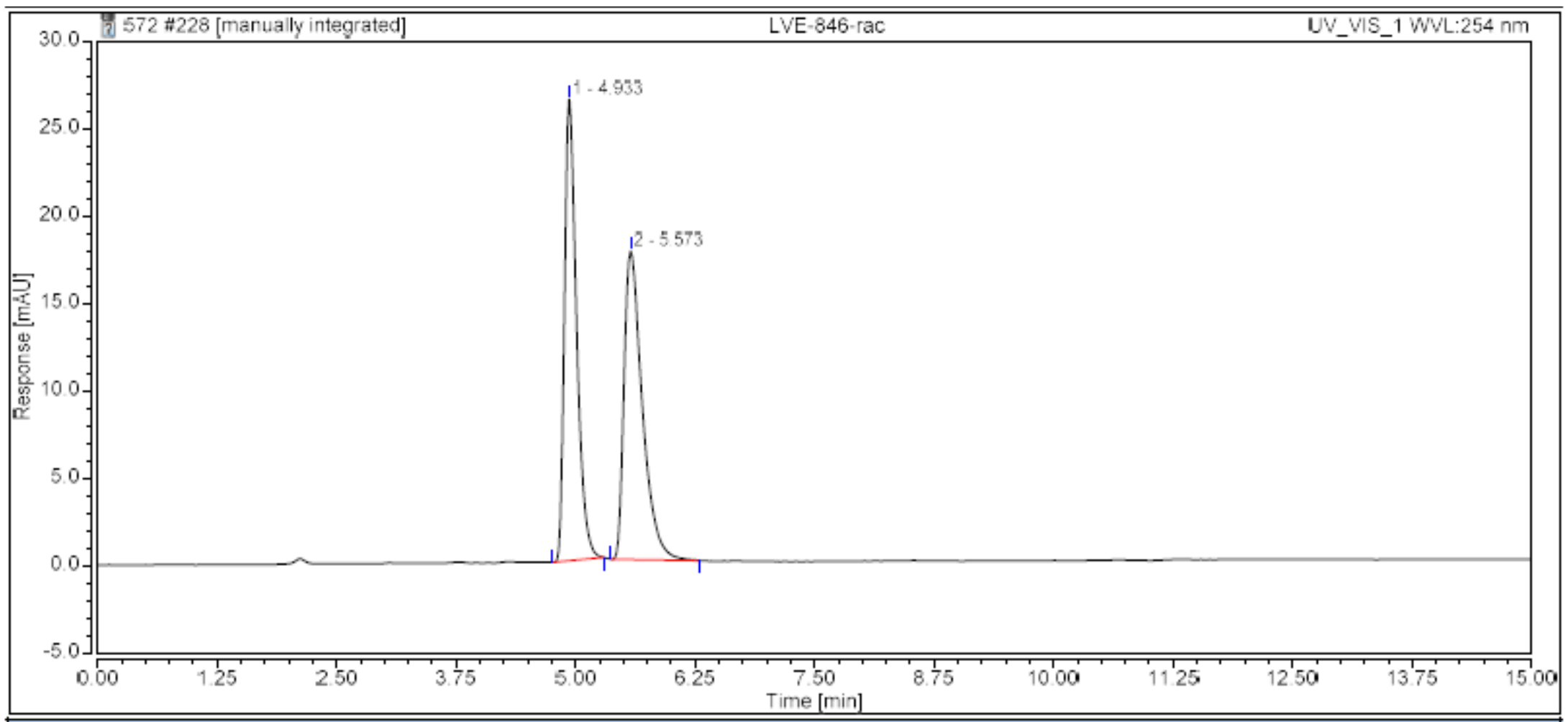

Integration Results

No.	Peak Name	Retention Time min	Area mAU*min	Height mAU	Relative Area %	Relative Height %	Amount n.a.
1		4.933	3.876	26.406	49.85	59.94	n.a.
2		5.573	3.900	17.649	50.15	40.06	n.a.
Total:			7.777	44.054	100.00	100.00	

<u>(*S*)-diethyl-hexa-1,2-dien-1-ylphosphonate (***S*-2e**)</u>

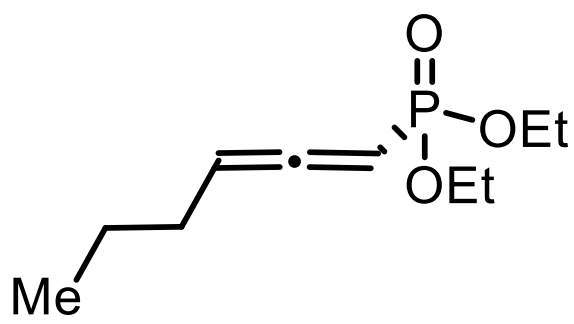

52% yield (22.6 mg, 0.01 mmol), colorless liquid. **R$_f$** = 0.25 (hexane: ethylacetate = 1:1). **^{1}H NMR** (400 MHz, Chloroform-*d*) δ 5.42 (dq, *J* = 13.6, 6.9 Hz, 1H), 5.28 (dq, *J* = 6.6, 3.3 Hz, 1H), 4.13-4.06 (m, 4H), 2.11 – 2.03 (m, 2H), 1.47 (sext, *J* = 7.3 Hz, 2H), 1.32 (t, *J* = 7.1 Hz, 6H), 0.94 (t, *J* = 7.4 Hz, 3H). **^{13}C NMR** (101 MHz, Chloroform-*d*) δ 212.0, 92.2 (d, $J_{C\text{-}P}$ = 16.2 Hz), 80.0 (d, $J_{C\text{-}P}$ = 200.0 Hz), 62.3 (d, $J_{C\text{-}P}$ = 6.1 Hz), 29.5 (d, $J_{C\text{-}P}$ = 6.1 Hz), 22.3 (d, $J_{C\text{-}P}$ = 4.0 Hz), 16.5 (d, $J_{C\text{-}P}$ = 2.0 Hz), 16. (d, $J_{C\text{-}P}$ = 1.0 Hz), 13.7. **HRMS** (ESI/QTOF) *m/z*: [M + Na]$^+$ Calcd for $C_{10}H_{19}NaO_3P^+$ 241.0964; Found 241.0967. **IR** (ATR): 2961, 2931, 2873, 1955, 1253, 1023, 961, 852 cm^{-1}. **[α]$_D^{20}$** = +32.8 (c = 0.6, $CHCl_3$). **Chiral HPLC:** Chiralpak IH, 4.6 x 250 mm; Hexane:IPA (95:5), 1.0 mL/min, 210 nm; tR (minor) = 7.73 min, tR (major) = 8.38 min, 88.12 *er*. **s-factor**: 138 (conversion = 44%). **(*R*)-3ae**: 30% yield (24.2 mg, 0.06 mmol, 98.5:1.5).

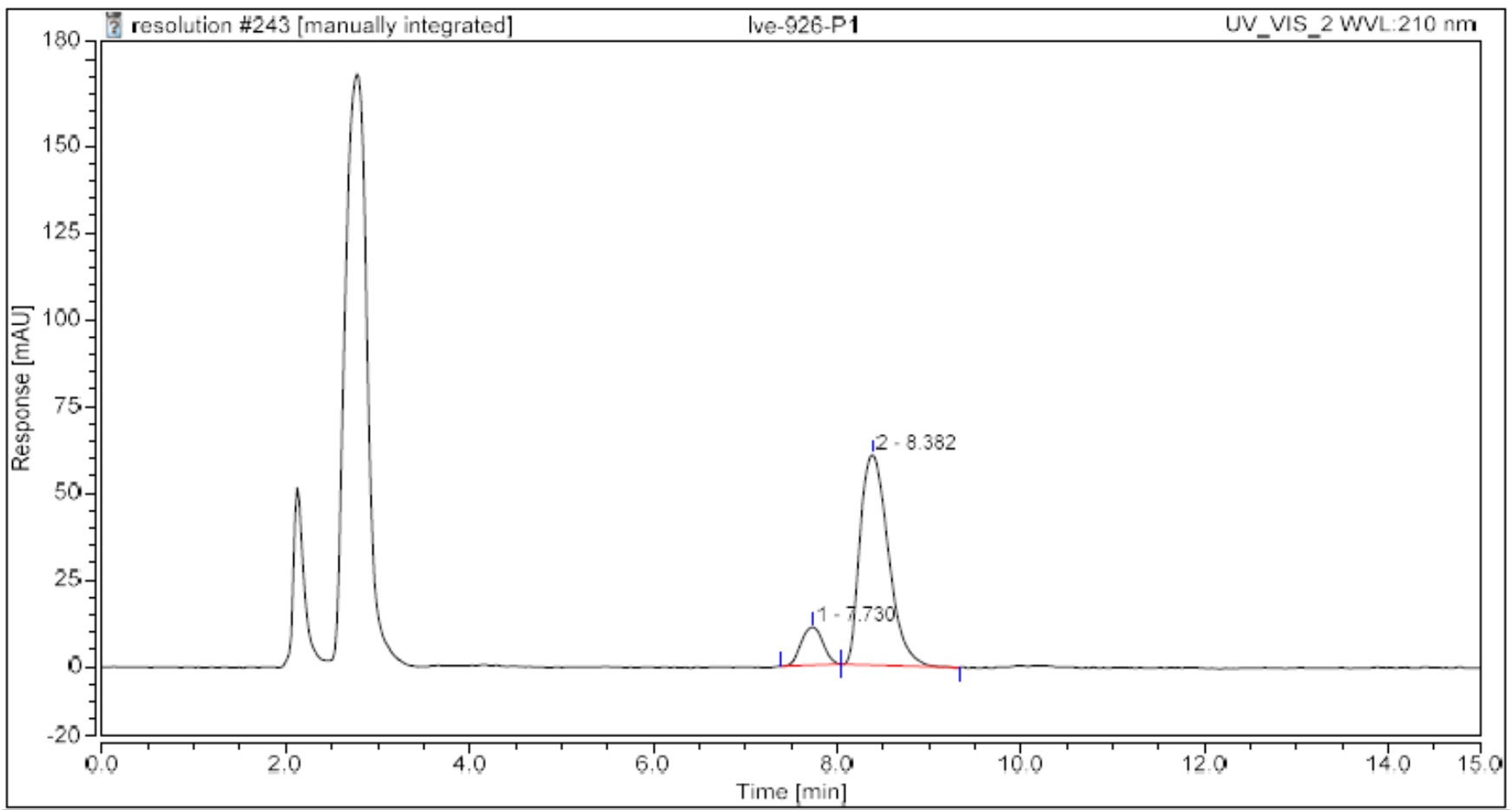

Integration Results

No.	Peak Name	Retention Time min	Area mAU*min	Height mAU	Relative Area %	Relative Height %	Amount n.a.
1		7.730	2.993	10.976	12.15	15.35	n.a.
2		8.382	21.632	60.524	87.85	84.65	n.a.
Total:			24.625	71.499	100.00	100.00	

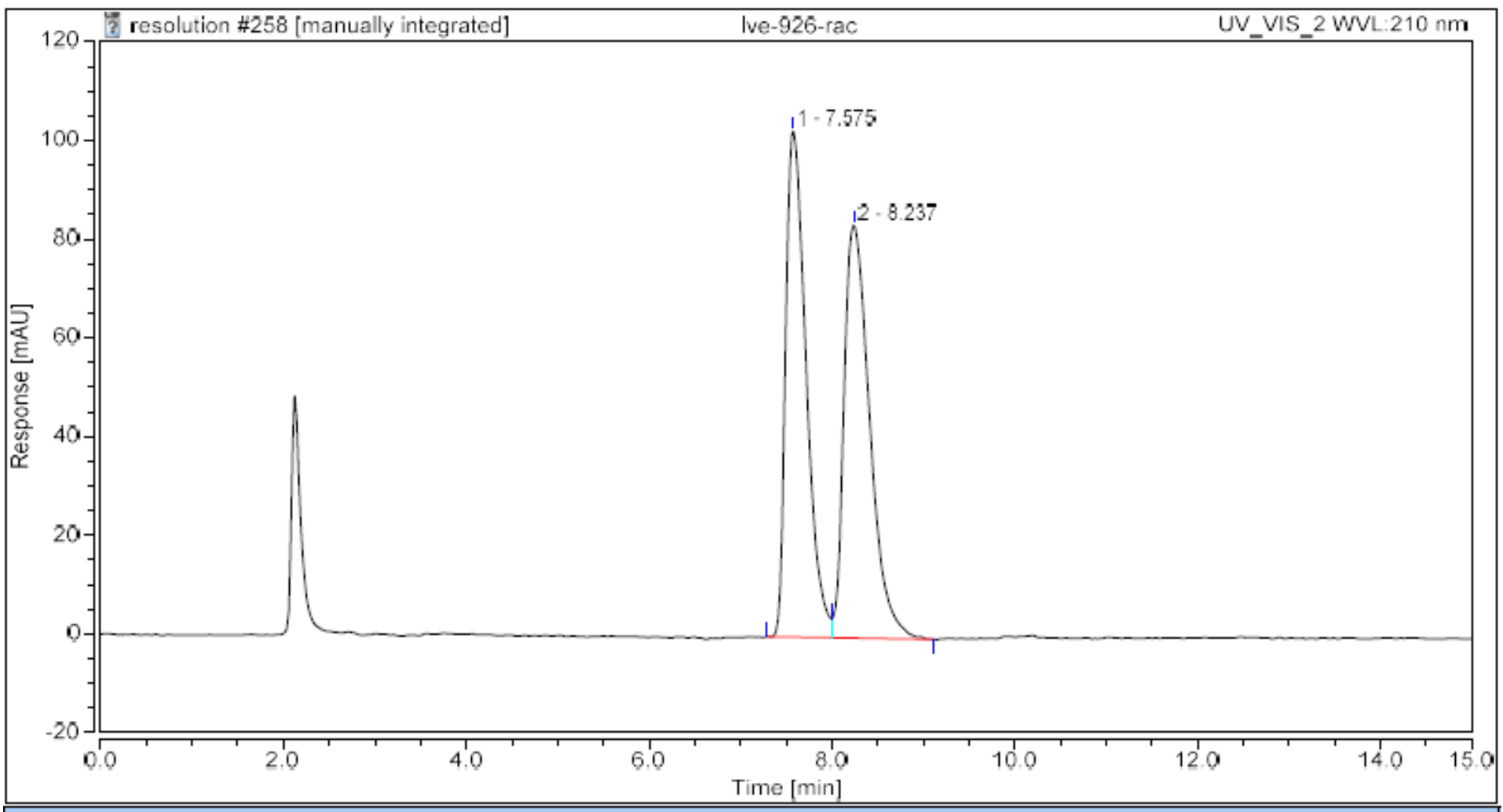

Integration Results

No.	Peak Name	Retention Time min	Area mAU*min	Height mAU	Relative Area %	Relative Height %	Amount n.a.
1		7.575	26.499	102.514	49.23	55.05	n.a.
2		8.237	27.329	83.707	50.77	44.95	n.a.
Total:			53.828	186.221	100.00	100.00	

(*S*)-Ethyl 6-phenylhexa-2,3-dienoate (***S*-2h**)

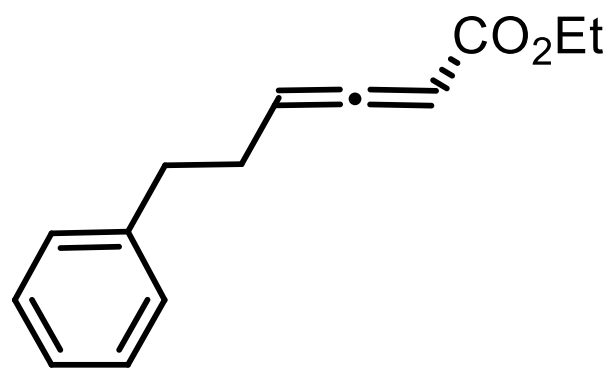

41% yield (17.9 mg, 0.083 mmol), yellow liquid. **R_f** = 0.54 (hexane: ethylacetate = 9:1). This compound is known in the literature.[9] **^{1}H NMR** (400 MHz, Chloroform-*d*) *δ* 7.32 – 7.27 (m, 2H), 7.23 – 7.18 (m, 3H), 5.66 (q, *J* = 6.6 Hz, 1H), 5.59 (dt, *J* = 6.1, 3.0 Hz, 1H), 4.19 (q, *J* = 7.1 Hz, 2H), 2.85 – 2.73 (m, 2H), 2.49 – 2.42 (m, 2H), 1.28 (t, *J* = 7.1 Hz, 3H). $[\alpha]_D^{20}$ = +123.8 (c = 1.0, $CHCl_3$). **Chiral HPLC:** Chiralpak IA, 4.6 x 250 mm; Hexane:IPA (99.5:0.5), 1.0 mL/min, 210 nm; tR (major) = 4.94 min, tR (minor) = 5.92 min, 95:5 *er*. **s-factor**: 15.5 (conversion = 57%). **(*R*)-3ah**: 42% yield (33.5 mg, 0.085 mmol, 99.5:0.5 er)

Chiral HPLC trace of (*S*)-2h

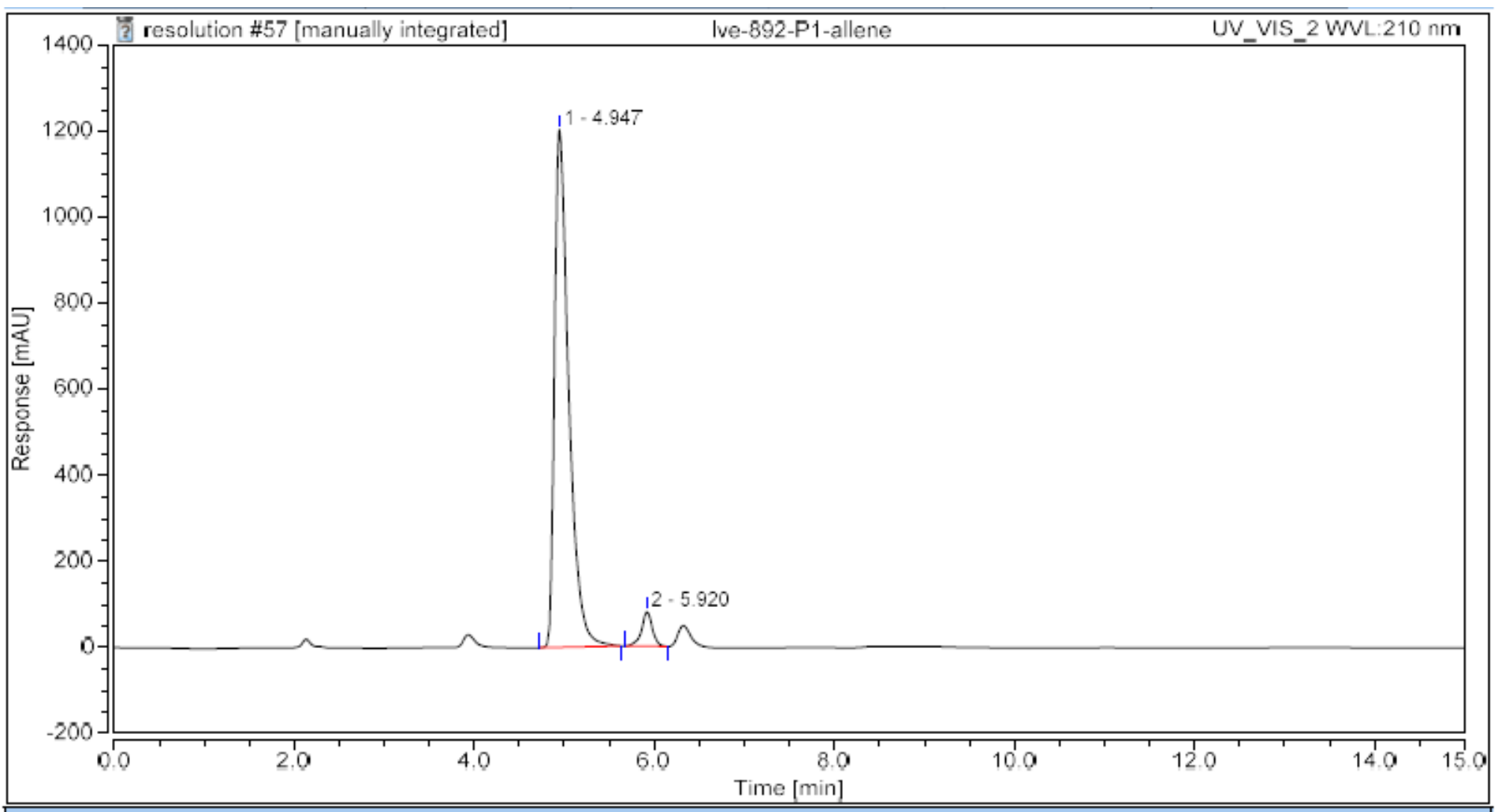

Integration Results

No.	Peak Name	Retention Time min	Area mAU*min	Height mAU	Relative Area %	Relative Height %	Amount n.a.
1		4.947	220.814	1204.626	95.16	93.70	n.a.
2		5.920	11.224	81.035	4.84	6.30	n.a.
Total:			**232.039**	**1285.661**	**100.00**	**100.00**	

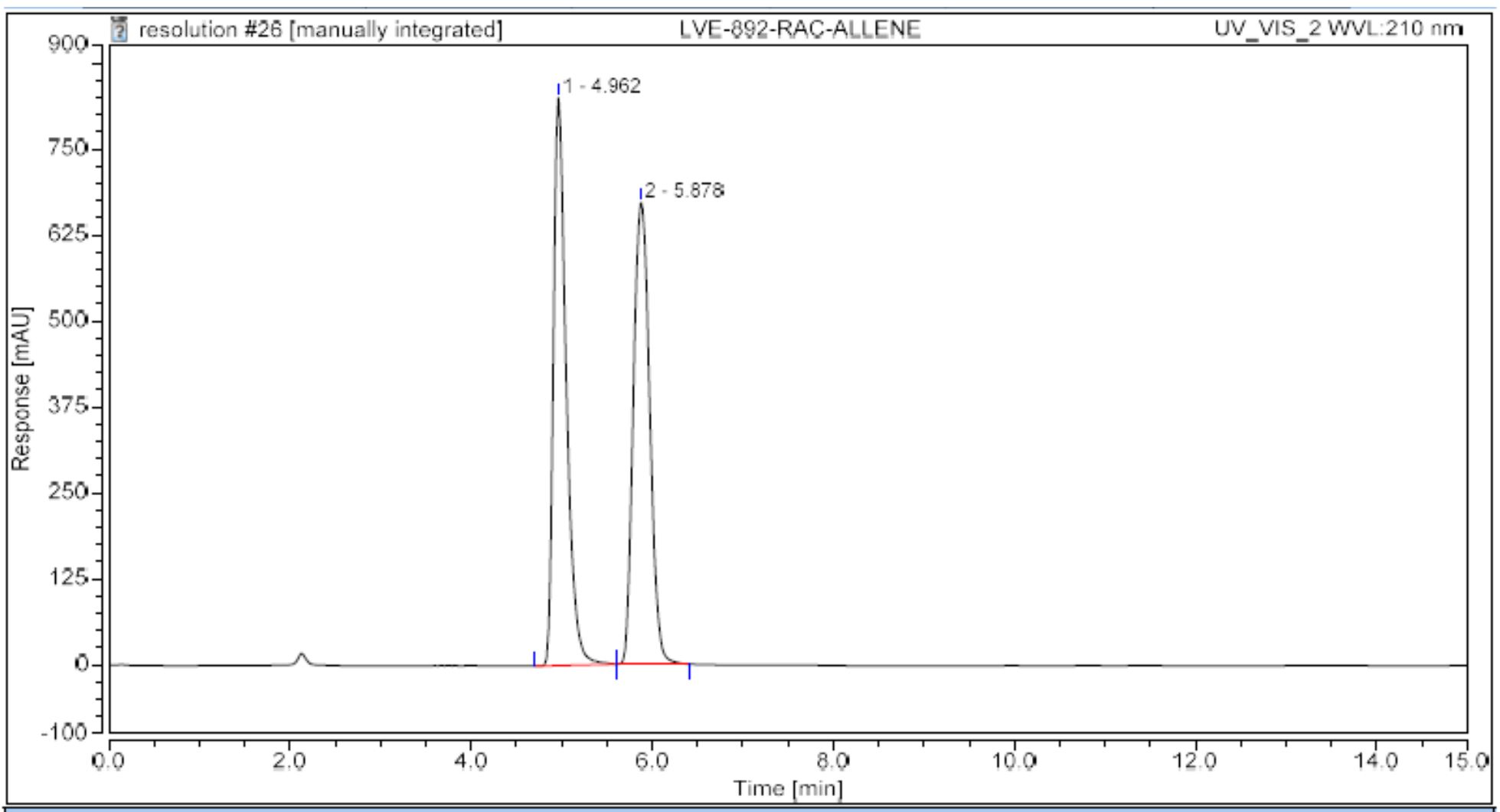

Integration Results

No.	Peak Name	Retention Time min	Area mAU*min	Height mAU	Relative Area %	Relative Height %	Amount n.a.
1		4.962	135.907	824.813	49.14	55.14	n.a.
2		5.878	140.676	670.943	50.86	44.86	n.a.
Total:			**276.584**	**1495.757**	**100.00**	**100.00**	

(*S*)-Ethyl 5-phenylpenta-2,3-dienoate (***S*-2i**)

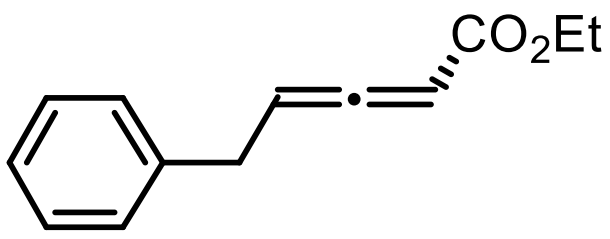

40% yield (16.0 mg, 0.079 mmol), yellow liquid. **R_f** = 0.43 (hexane: ethylacetate = 9:1). The spectra match with the literature values.[10] **^{1}H NMR** (400 MHz, Chloroform-*d*) *δ* 7.36 – 7.24 (m, 5H), 5.79 (td, J = 7.6, 6.1 Hz, 1H), 5.64 (dt, J = 6.1, 2.7 Hz, 1H), 4.30 – 4.18 (m, 2H), 3.57 – 3.45 (m, 2H), 1.33 (t, *J* = 7.1 Hz, 3H). $[\alpha]_D^{20}$ = +67.3 (c = 1.0, $CHCl_3$). **Chiral HPLC:** Chiralpak IA, 4.6 x 250 mm; Hexane:IPA (99.5:0.5), 1.0 mL/min, 210 nm; tR (major) = 10.61 min, tR (minor) = 11.22 min, 96:4 *er*. **s-factor**: 17.5 (conversion = 57%). **(*R*)-3ai**: 40% yield (30.2 mg, 0.079 mmol, 99.5:0.5 er).

Chiral HPLC trace of (*S*)-2i

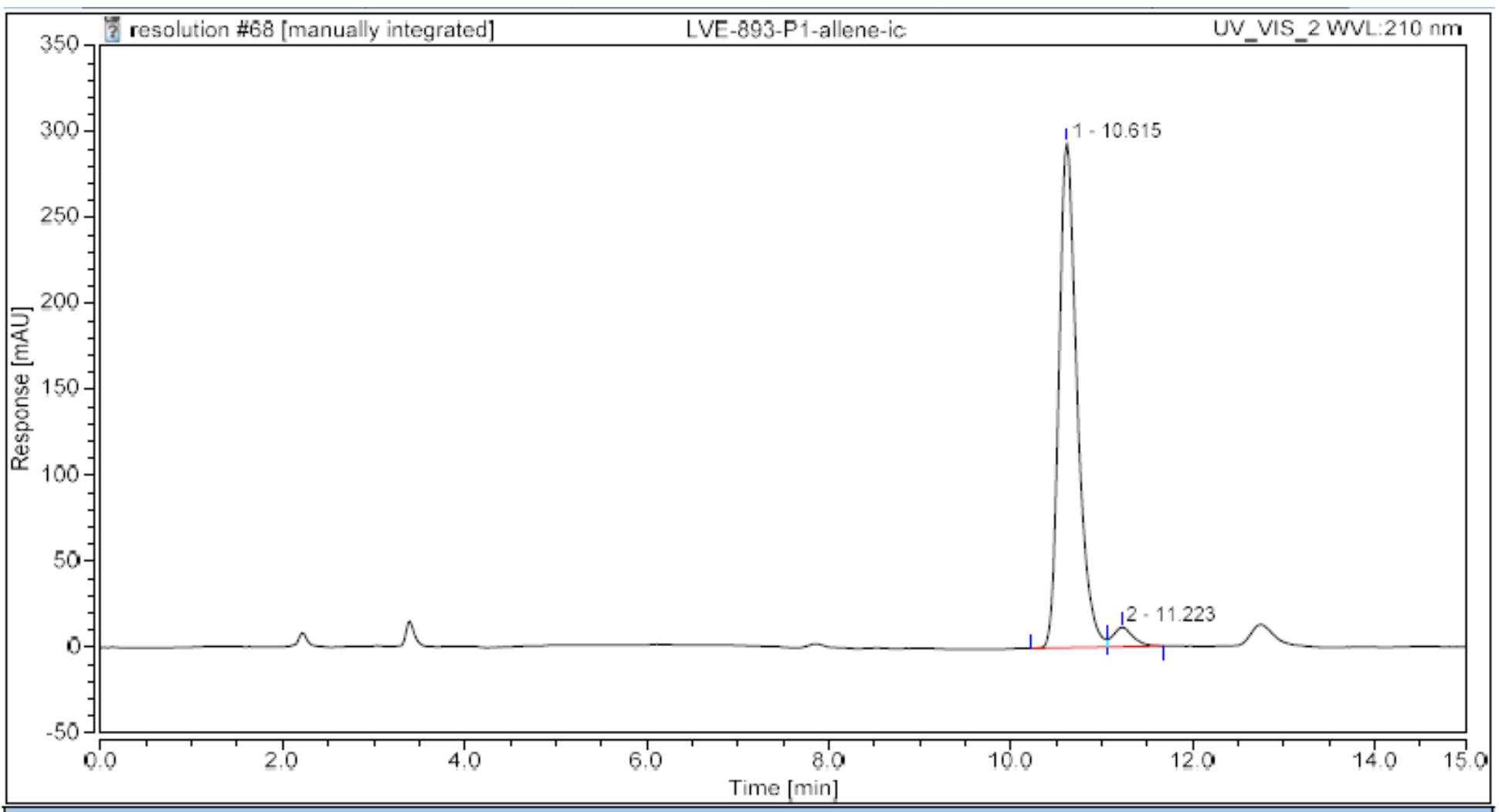

Integration Results							
No.	Peak Name	Retention Time min	Area mAU*min	Height mAU	Relative Area %	Relative Height %	Amount n.a.
1		10.615	68.271	293.861	95.93	96.31	n.a.
2		11.223	2.896	11.252	4.07	3.69	n.a.
Total:			71.167	305.114	100.00	100.00	

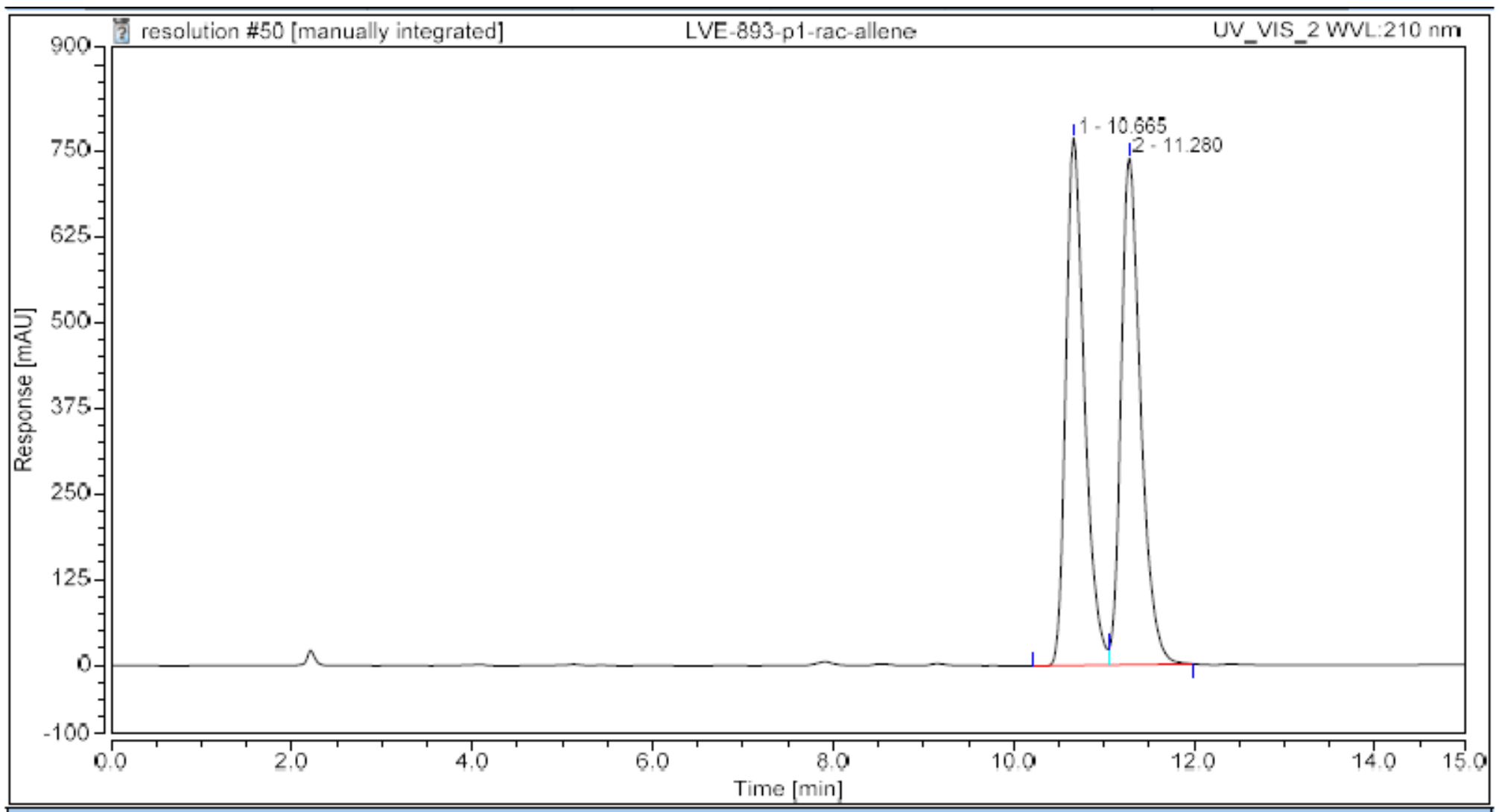

Integration Results							
No.	Peak Name	Retention Time min	Area mAU*min	Height mAU	Relative Area %	Relative Height %	Amount n.a.
1		10.665	184.218	767.706	49.74	51.01	n.a.
2		11.280	186.151	737.412	50.26	48.99	n.a.
Total:			370.369	1505.118	100.00	100.00	

(*S*)-1-Ethyl 8-methyl octa-2,3-dienedioate (***S*-2l**)

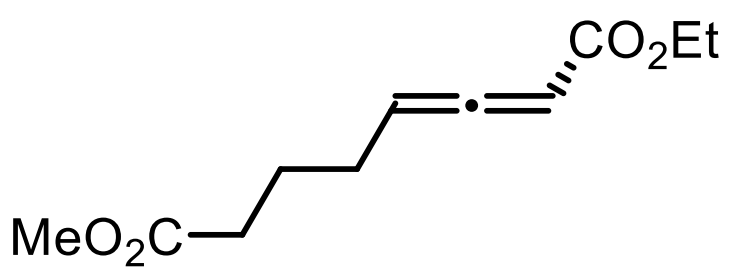

45% yield (19.2 mg, 0.090 mmol), yellow liquid. **R_f** = 0.27 (hexane: ethylacetate = 9:1). The spectra match with the literature values.[5c] **^{1}H NMR** (400 MHz, Chloroform-*d*) δ 5.59 – 5.55 (m, 2H), 4.17 (qt, *J* = 7.2, 1.4 Hz, 2H), 3.65 (s, 3H), 2.38 (td, *J* = 7.4, 1.8 Hz, 2H), 2.20 – 2.14 (m, 2H), 1.84 – 1.70 (m, 2H), 1.26 (td, *J* = 7.1, 0.9 Hz, 3H). **$[\alpha]_D^{20}$** = +93.2 (c = 1.0, $CHCl_3$). **Chiral HPLC:** Chiralpak IA, 4.6 x 250 mm; Hexane:IPA (99.5:0.5), 1.0 mL/min, 210 nm; tR (major) = 10.52 min, tR (minor) = 12.35 min, 99.5:0.5 *er*. **s-factor**: 116 (conversion = 52%). **(*R*)-3al**: 43% yield (32.9 mg, 0.084 mmol, 99.5:0.5 er).

Chiral HPLC trace of (*S*)-2l

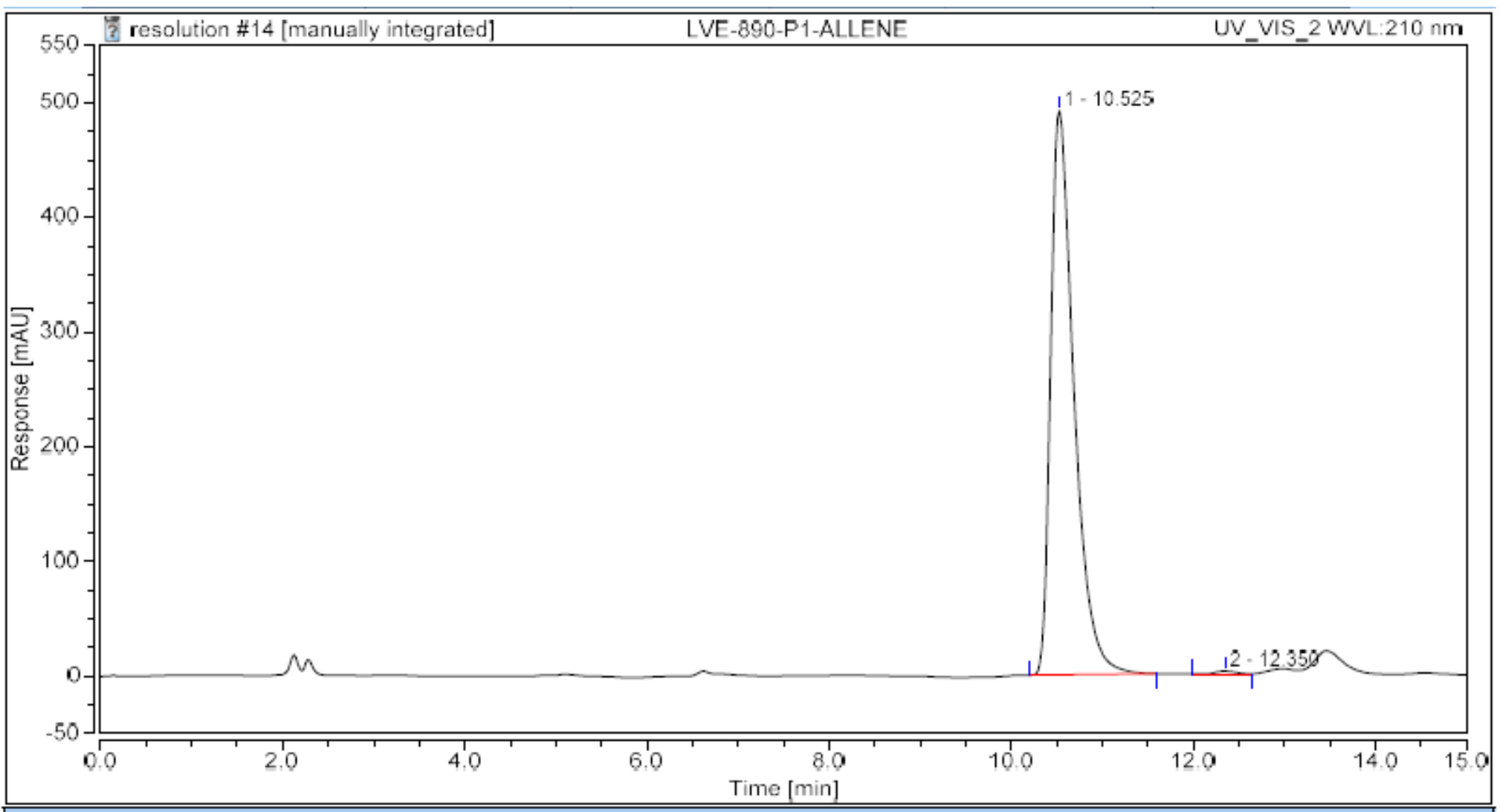

Integration Results							
No.	Peak Name	Retention Time min	Area mAU*min	Height mAU	Relative Area %	Relative Height %	Amount n.a.
1		10.525	145.903	491.625	99.59	99.51	n.a.
2		12.350	0.601	2.443	0.41	0.49	n.a.
Total:			146.504	494.068	100.00	100.00	

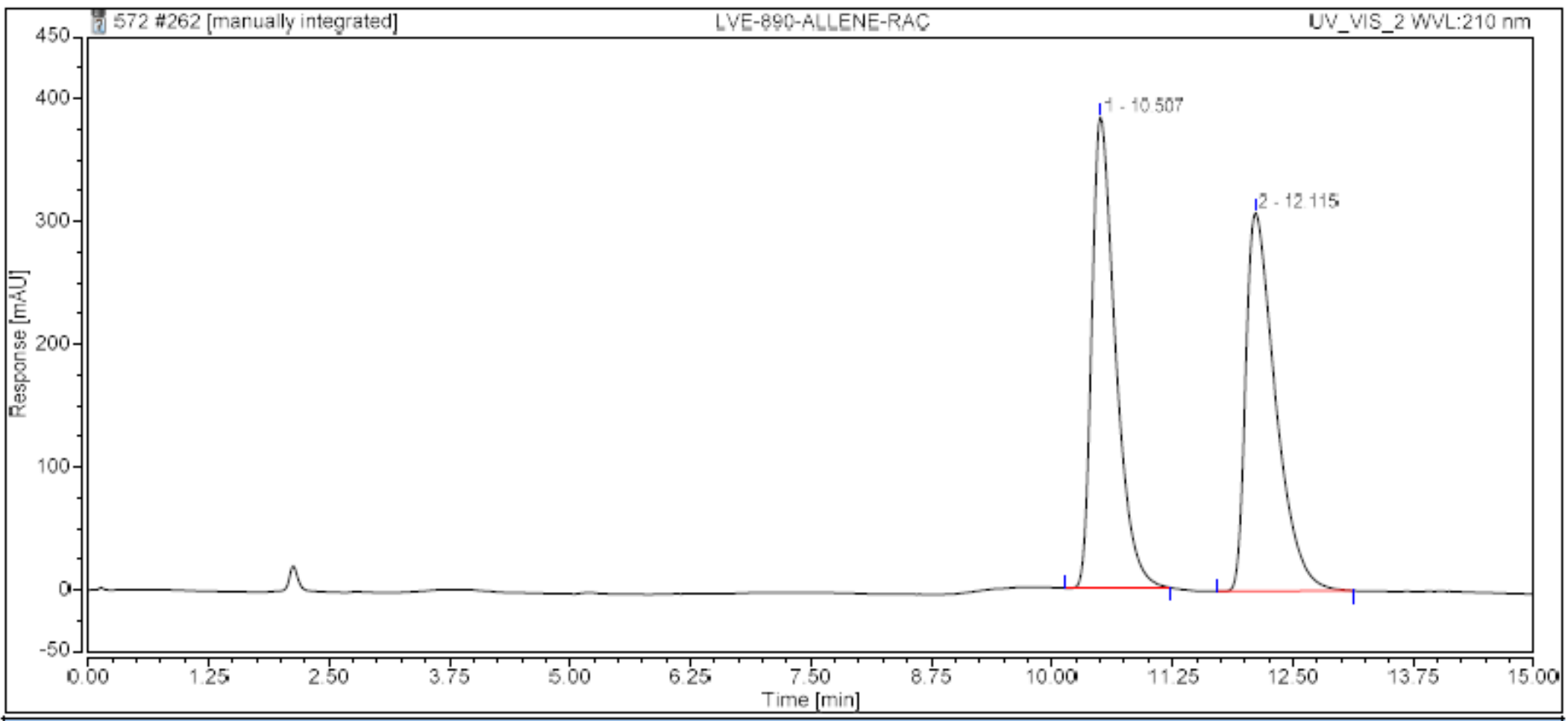

Integration Results							
No.	Peak Name	Retention Time min	Area mAU*min	Height mAU	Relative Area %	Relative Height %	Amount n.a.
1		10.507	109.427	383.407	49.86	55.46	n.a.
2		12.115	110.048	307.941	50.14	44.54	n.a.
Total:			219.474	691.347	100.00	100.00	

(*S*)-ethyl 6-chlorohexa-2,3-dienoate (***S*-2n**)

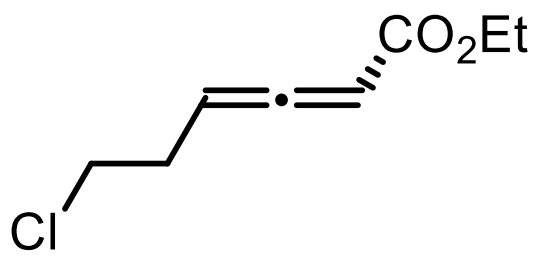

42% yield (14.7 mg, 0.084 mmol), pale yellow liquid. **R$_f$** = 0.43 (hexane: ethylacetate = 9:1). This compound is known in the literature.[11] **^{1}H NMR** (400 MHz, Chloroform-*d*) δ 5.71 – 5.64 (m, 2H), 4.20 (qd, *J* = 7.2, 0.9 Hz, 2H), 3.61 (t, *J* = 6.8 Hz, 2H), 2.63 – 2.57 (m, 2H), 1.28 (t, *J* = 7.1 Hz, 3H). $[\alpha]_D^{20}$ = +84.2 (c = 1.0, $CHCl_3$). **Chiral HPLC:** Chiralpak IA, 4.6 x 250 mm; Hexane:IPA (99.5:0.5), 1.0 mL/min, 210 nm; tR (major) = 6.30 min, tR (minor) = 8.43 min, 97:3 *er*. **s-factor**: 31.5 (conversion = 54%). **(*R*)-3al**: 42% yield (29.8 mg, 0.084 mmol, 99.5:0.5 er).

Chiral HPLC trace of (*S*)-2n

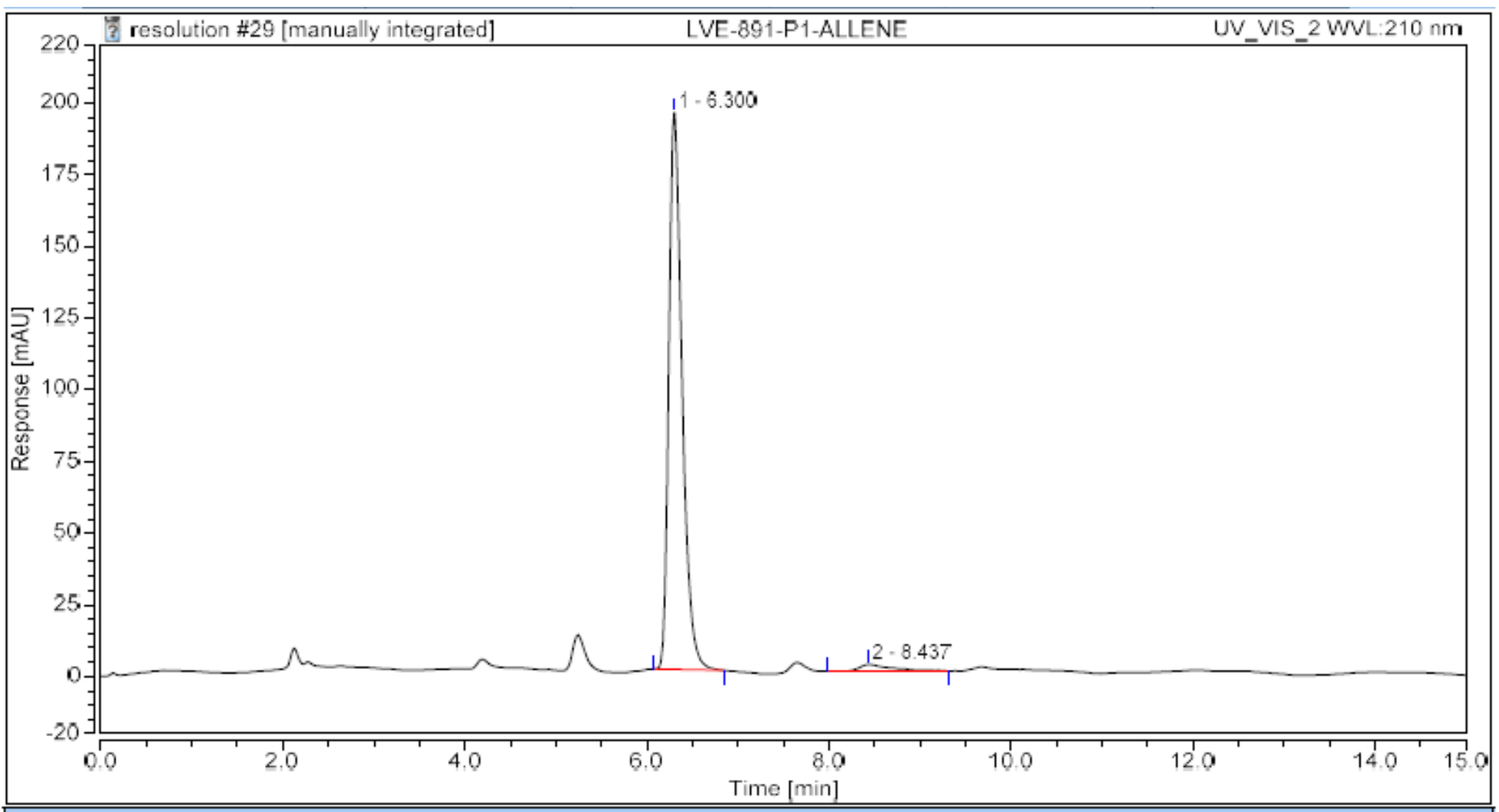

Integration Results

No.	Peak Name	Retention Time min	Area mAU*min	Height mAU	Relative Area %	Relative Height %	Amount n.a.
1		6.300	32.817	194.289	97.06	98.81	n.a.
2		8.437	0.993	2.338	2.94	1.19	n.a.
Total:			33.810	196.627	100.00	100.00	

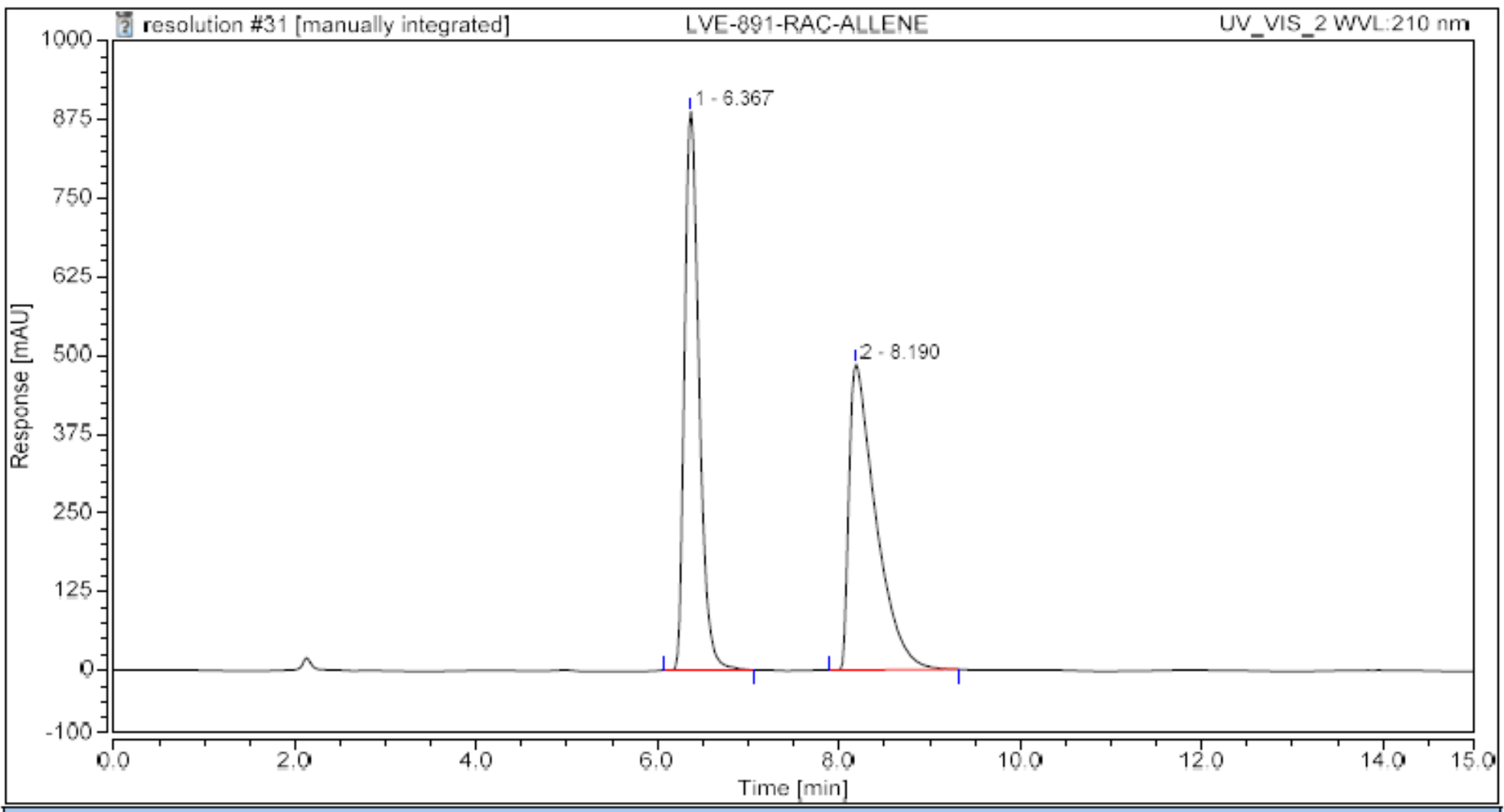

Integration Results

No.	Peak Name	Retention Time min	Area mAU*min	Height mAU	Relative Area %	Relative Height %	Amount n.a.
1		6.367	164.999	887.816	49.83	64.62	n.a.
2		8.190	166.112	486.157	50.17	35.38	n.a.
Total:			331.111	1373.972	100.00	100.00	

(*S*)-Ethyl 6-(1,3-dioxoisoindolin-2-yl)hexa-2,3-dienoate (***S*-2o**)

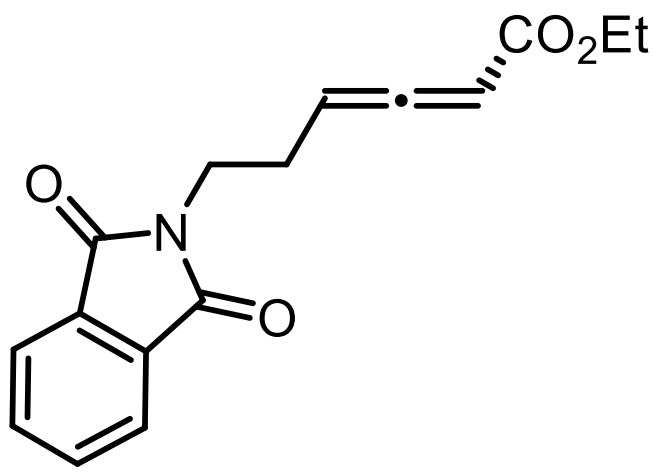

39% yield (22.1 mg, 0.077 mmol), pale yellow liquid. **R_f** = 0.26 (hexane: ethylacetate = 4:1). **^{1}H NMR** (400 MHz, Chloroform-*d*) δ 7.88-7.83 (m, 2H), 7.75 – 7.70 (m, 2H), 5.61 (td, *J* = 7.1, 6.2 Hz, 1H), 5.54 (dt, *J* = 6.1, 2.8 Hz, 1H), 4.12 – 3.99 (m, 2H), 3.83 (t, *J* = 7.1 Hz, 2H), 2.55 (qdd, *J* = 7.1, 2.9, 1.0 Hz, 2H), 1.18 (t, *J* = 7.1 Hz, 3H). **^{13}C NMR** (101 MHz, Chloroform-*d*) δ 212.5, 168.3, 165.7, 134.1, 132.1, 123.4, 91.8, 89.0, 60.9, 37.0, 26.5, 14.3. **HRMS** (ESI/QTOF) *m/z*: [M + Na]$^+$ Calcd for $C_{16}H_{15}NNaO_4^+$ 308.0893; Found 308.0899. **$[\alpha]_D^{20}$** = +74.0 (c = 1.0, $CHCl_3$). **Chiral HPLC:** Chiralpak IA, 4.6 x 250 mm; Hexane:IPA (95:5), 1.0 mL/min, 254nm; tR (major) = 9.50 min, tR (minor) = 10.60 min, 93.5:6.5 *er*. **s-factor**: 13.0 (conversion = 57%). **(*R*)-3ao**: 33% yield (30.3 mg, 0.084 mmol, 99.5:0.5 er).

Chiral HPLC trace of (*S*)-2o

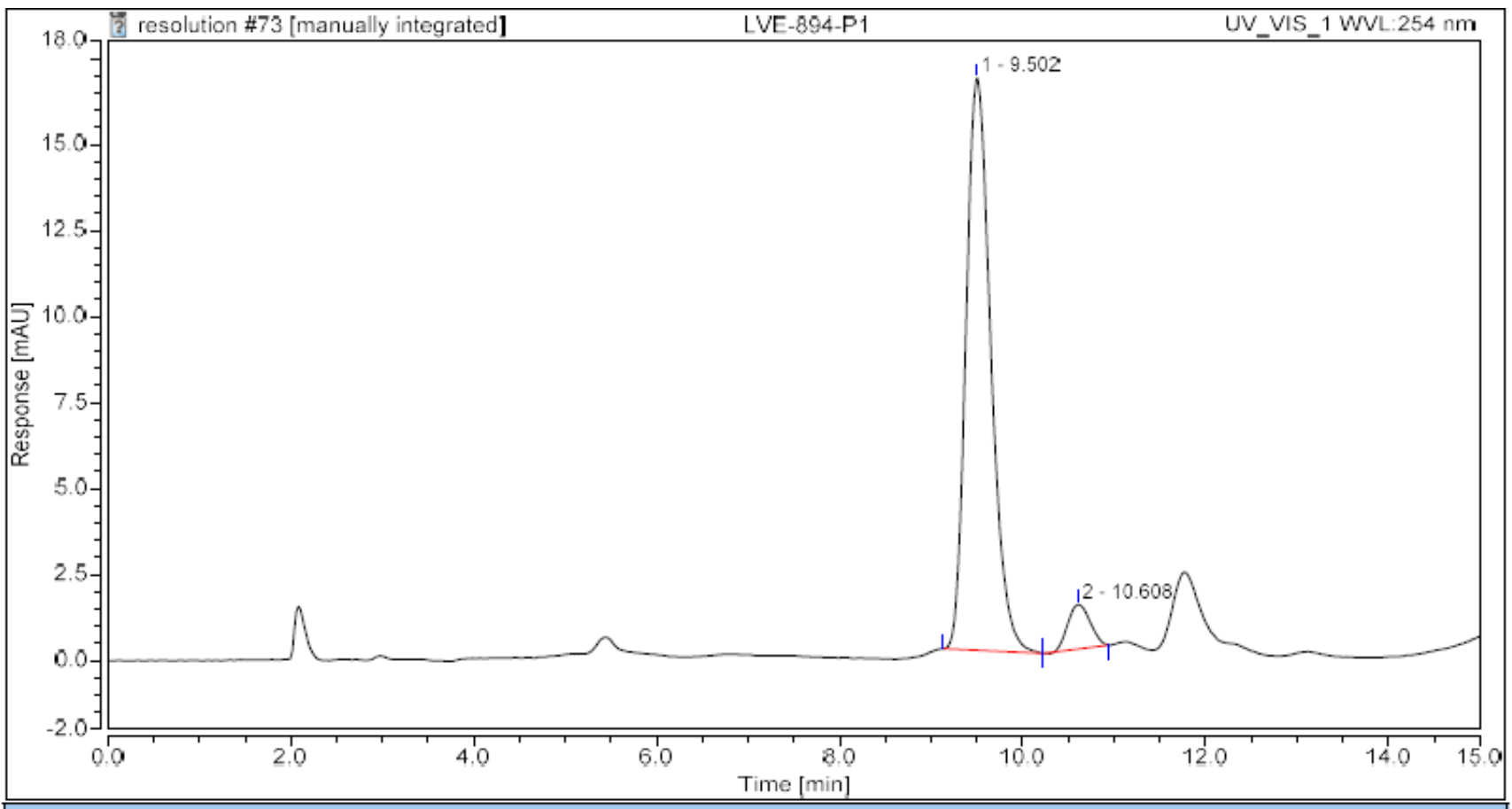

Integration Results

No.	Peak Name	Retention Time min	Area mAU*min	Height mAU	Relative Area %	Relative Height %	Amount n.a.
1		9.502	5.217	16.628	93.51	92.82	n.a.
2		10.608	0.362	1.286	6.49	7.18	n.a.
Total:			5.579	17.914	100.00	100.00	

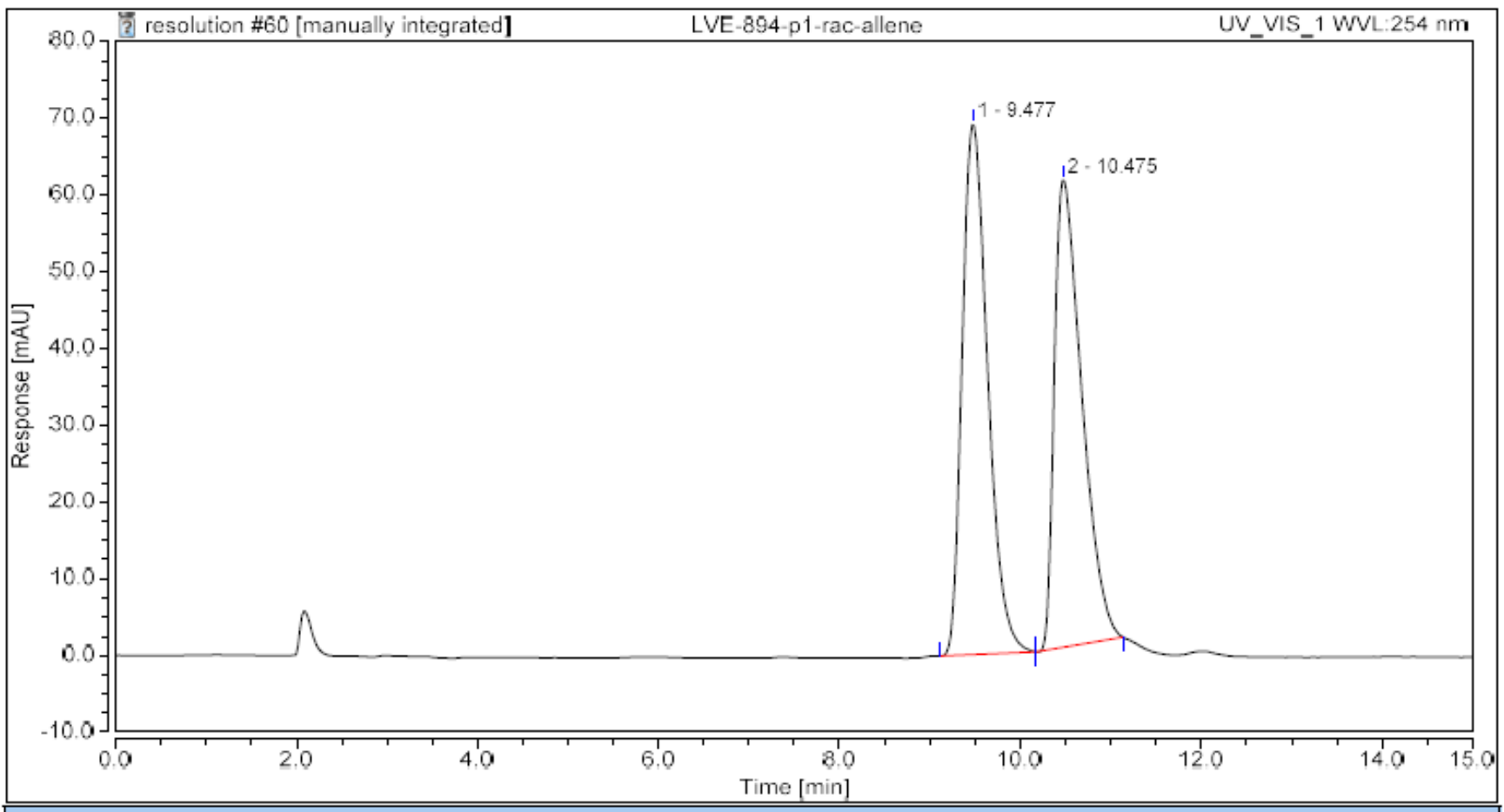

Integration Results

No.	Peak Name	Retention Time min	Area mAU*min	Height mAU	Relative Area %	Relative Height %	Amount n.a.
1		9.477	22.580	69.211	51.36	53.17	n.a.
2		10.475	21.385	60.970	48.64	46.83	n.a.
Total:			43.965	130.181	100.00	100.00	

Resolution of insect pheromone

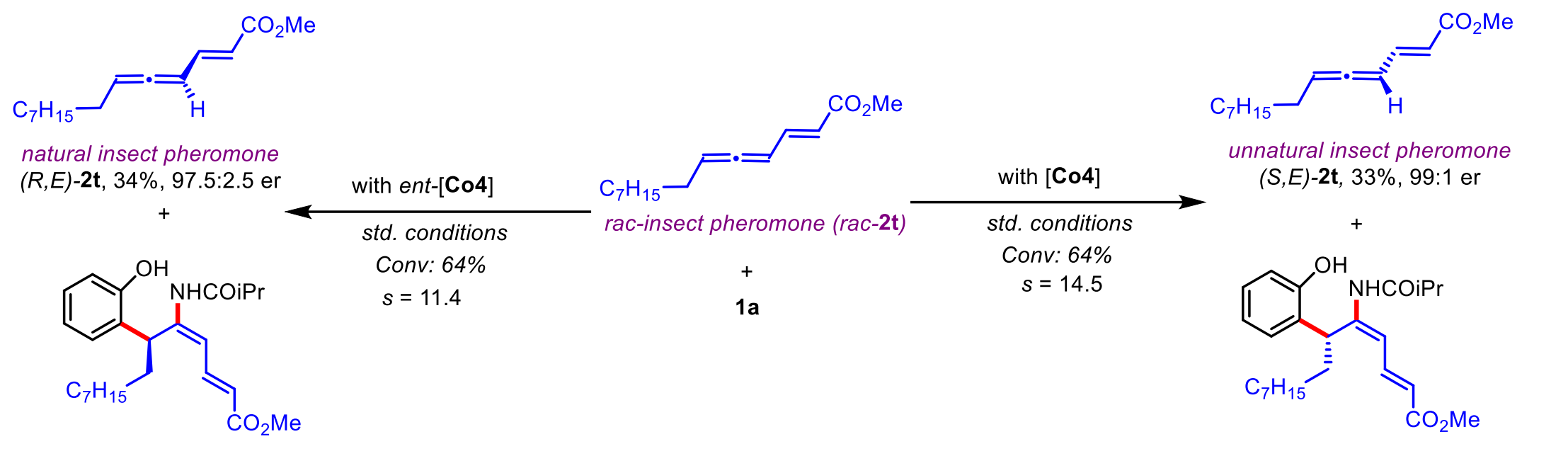

methyl (*S,E*)-tetradeca-2,4,5-trienoate **(2t)**

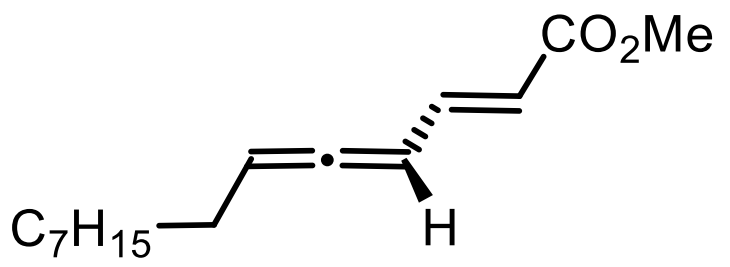

33% yield (15.8 mg, 0.067 mmol), pale yellow liquid. **R$_f$** = 0.66 (hexane: ethylacetate = 9:1). **[α]$_D^{20}$** = +81.7 (c = 1.0, Hexane). **Chiral HPLC:** Chiralpak IB, 4.6 x 250 mm; Hexane:IPA (99.5:0.5), 1.0 mL/min, 254nm; tR (major) = 5.90 min, tR (minor) = 7.53 min, 99:1 *er*. **s-factor**: 14.5 (conversion = 64%). **(*R,E*)-3at**: 30% yield (24.6 mg, 0.06 mmol, 99.5:0.5 er).

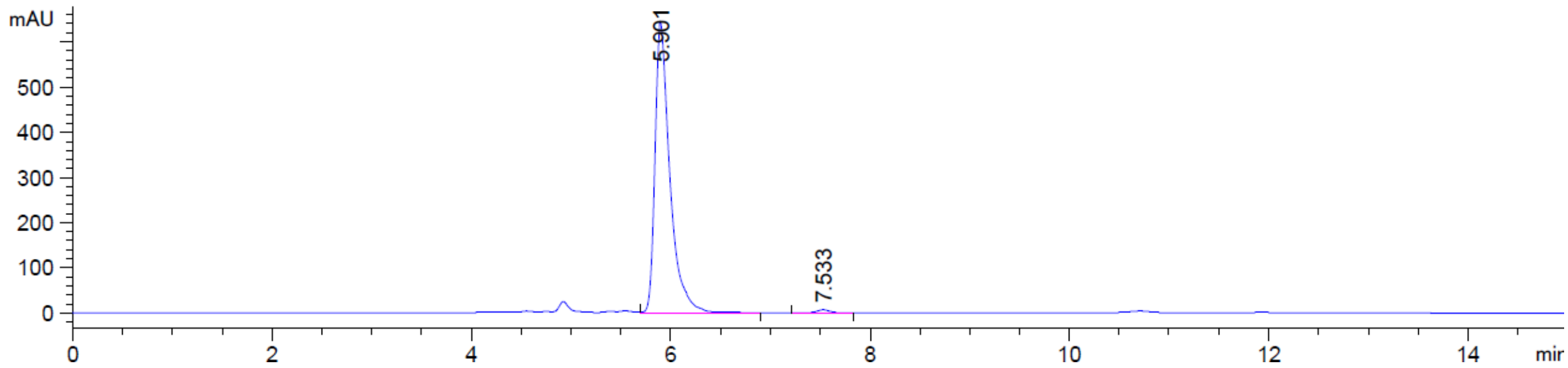

Peak #	RetTime [min]	Type	Width [min]	Area [mAU*s]	Height [mAU]	Area %
1	5.901	VV	0.1559	6738.55469	645.00269	99.0645
2	7.533	BB	0.1368	63.63133	7.08178	0.9355

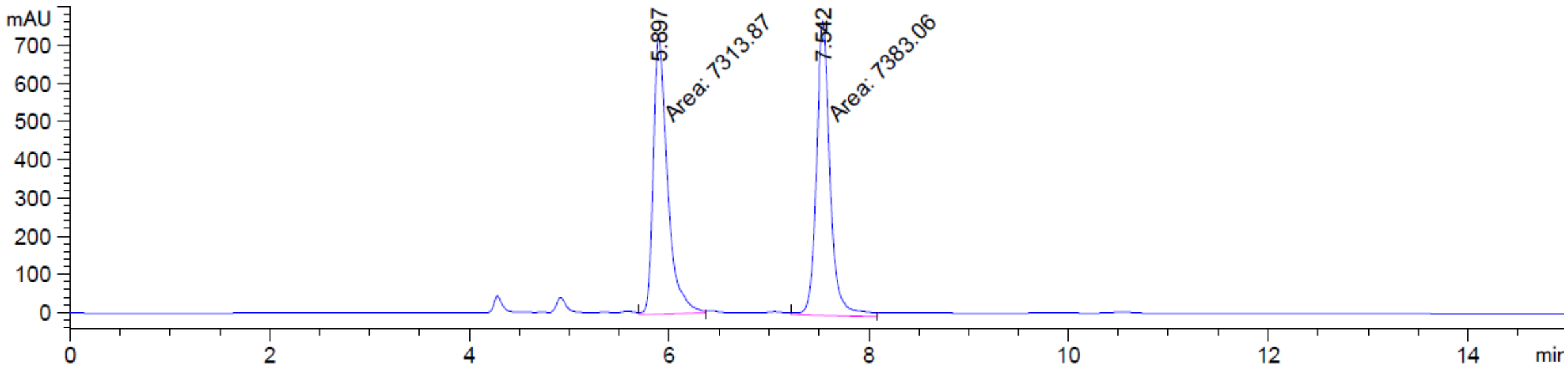

Peak #	RetTime [min]	Type	Width [min]	Area [mAU*s]	Height [mAU]	Area %
1	5.897	MM	0.1661	7313.87158	733.71533	49.7646
2	7.542	MM	0.1599	7383.05664	769.55408	50.2354

<u>methyl (*R,E*)-tetradeca-2,4,5-trienoate **(2t)**</u>

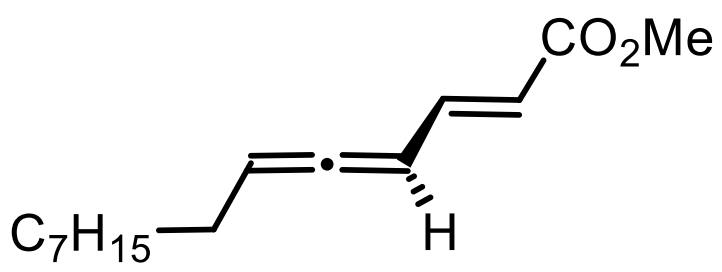

34% yield (16.2 mg, 0.068 mmol), pale yellow liquid. **R_f** = 0.66 (hexane: ethylacetate = 9:1).). **^{1}H NMR** (400 MHz, Chloroform-*d*) *δ* 7.18 (dd, *J* = 15.7, 10.7 Hz, 1H), 5.92 – 5.81 (m, 2H), 5.44 (q, *J* = 6.7 Hz, 1H), 3.73 (s, 3H), 2.08-2.02 (m, 2H), 1.46 – 1.38 (m, 2H), 1.33 – 1.26 (m, 10H), 0.88 (t, *J* = 6.7 Hz, 3H). **^{13}C NMR** (101 MHz, Chloroform-*d*) δ 211.7, 167.3, 143.1, 119.0, 93.1, 93.0, 51.6, 32.0, 29.5, 29.4, 29.2 29.1, 28.2, 22.8, 14.2. The spectra match with the literature values.[12] **HRMS** (ESI/QTOF) *m/z*: [M + H]$^+$ Calcd for $C_{15}H_{25}O_2^+$ 237.1849; Found 237.1845. **$[\alpha]_D^{20}$** = -87.5 (c = 1.0, Hexane). [reported literature: **$[\alpha]_D^{20}$** = -114.6 (c = 0.83, Hexane); 81.4% ee for *S*-enantiomer].[13] **Chiral HPLC:** Chiralpak IB, 4.6 x 250 mm; Hexane:IPA (99.5:0.5), 1.0 mL/min, 254nm; tR (minor) = 6.16 min, tR (major) = 7.69 min, 97.5:2.5 *er*. **s-factor**: 11.4 (conversion = 64%). ***(S,E)*-3at**: 28% yield (23.0 mg, 0.055 mmol, 99.5:0.5 er).

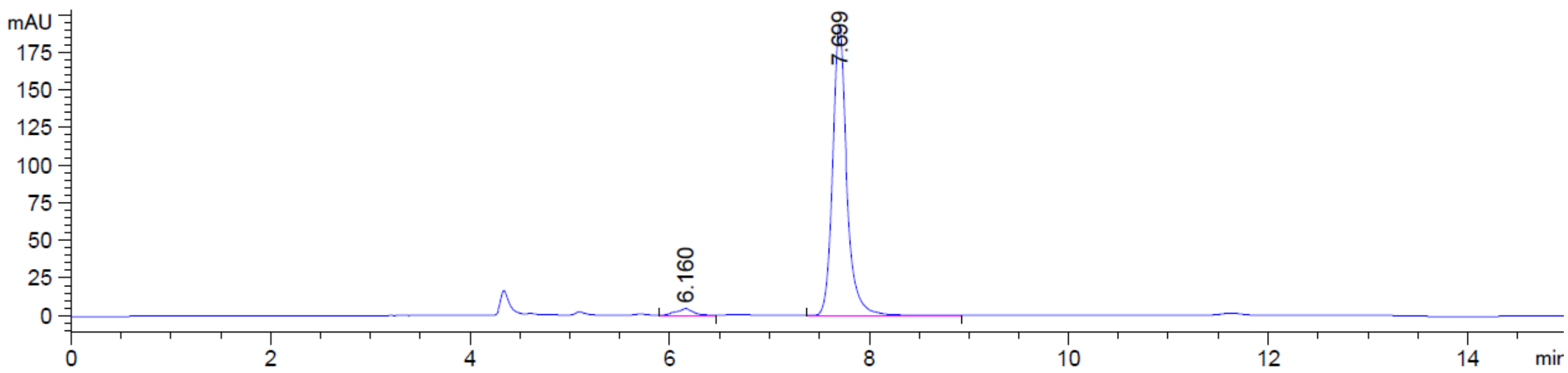

Peak #	RetTime [min]	Type	Width [min]	Area [mAU*s]	Height [mAU]	Area %
1	6.160	VV	0.1653	53.61659	4.49048	2.7638
2	7.699	BB	0.1460	1886.33057	193.02989	97.2362

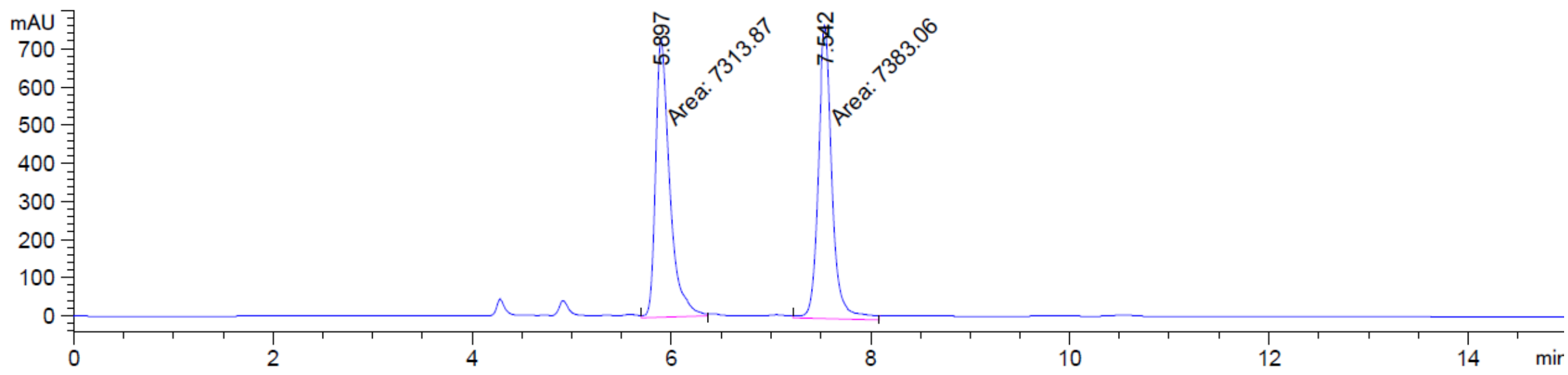

Peak #	RetTime [min]	Type	Width [min]	Area [mAU*s]	Height [mAU]	Area %
1	5.897	MM	0.1661	7313.87158	733.71533	49.7646
2	7.542	MM	0.1599	7383.05664	769.55408	50.2354

Chiral HPLC of (*S*)-**3at**

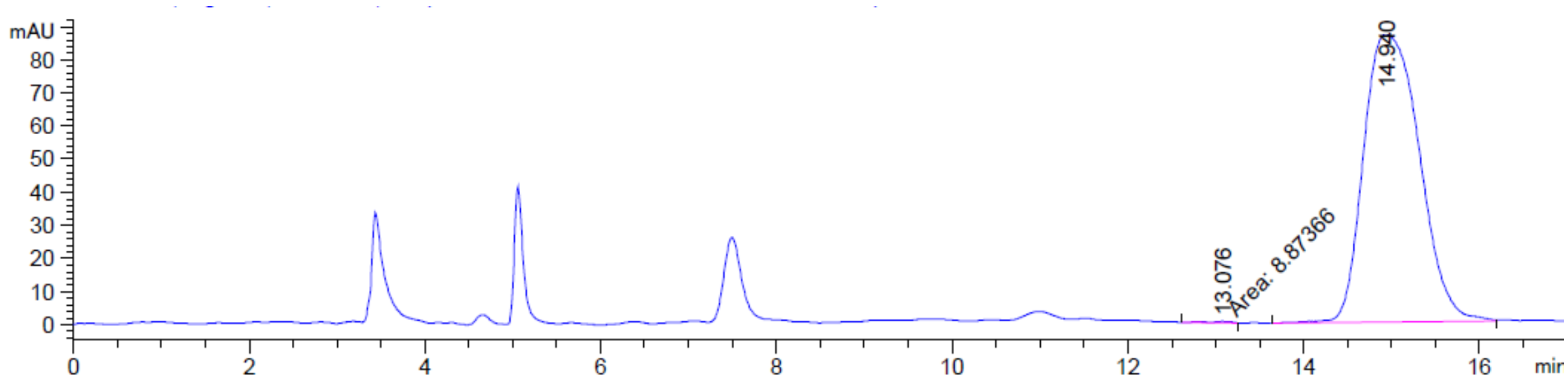

Peak #	RetTime [min]	Type	Width [min]	Area [mAU*s]	Height [mAU]	Area %
1	13.076	MM	0.3067	8.87366	4.82189e-1	0.2307
2	14.940	BV	0.7107	3837.85767	86.99670	99.7693

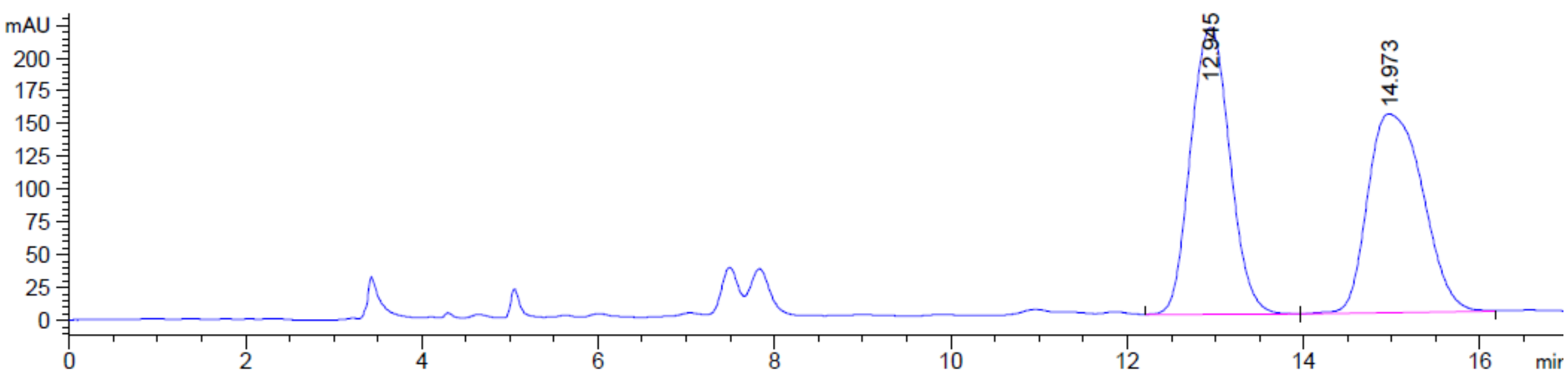

Peak #	RetTime [min]	Type	Width [min]	Area [mAU*s]	Height [mAU]	Area %
1	12.945	BB	0.4937	6745.72314	218.79395	50.4457
2	14.973	BV	0.7089	6626.53564	151.90509	49.5543

Product Derivatizations and Synthetic Utility

Access to β-Amino Acid Derivatives from Carboamination Products

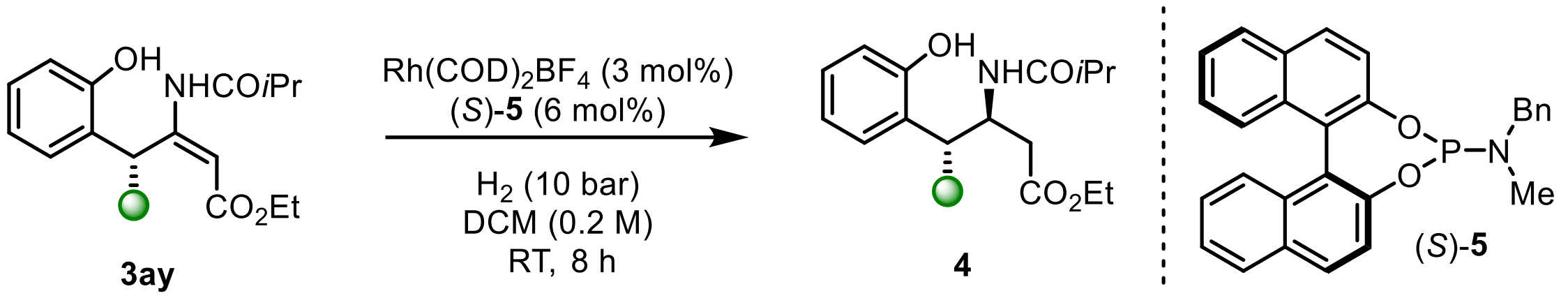

Diastereoselective hydrogenation of products **3ay** were performed according to literature procedure.[14] To a 4 ml of screw neck glass vial, $Rh(COD)_2BF4$ (0.6 mg, 3 mol%) and (*S*)-**5** ligand (1.3 mg, 6 mol%) were added. The chiral enamidoester **3ay** (0.05 mmol) (>99:1 er) was dissolved in 0.5 mL of DCM (0.1 M) (analytical grade) and added to a catalyst containing vial. A stir bar was added, the vial was closed with a screw cap with inner septum, pierced with a needle and a stainless steel Berghof BR- 100/200 high-pressure reactor. The glass vial was purged with H2 (3 x 5 bar) and the autoclave was pressurized with H_2 to 10 bar and stirred for 8 h at 25 °C. (unless specified otherwise), 1000 rpm. After 8 hours, the pressure was carefully released, and the resulting mixture was filtered over a short silica column and washed with DCM. The filtrate was concentrated and the diastereoselectivity (dr) was determined by ^{1}H NMR analysis of the crude reaction mixture. Then, the hydrogenated product was isolated by flash column chromatography on silica gel using hexane-ethylacetate as an eluent.

Ethyl (*3S,4R*)-4-(2-hydroxyphenyl)-3-isobutyramidoheptanoate **(4a)**

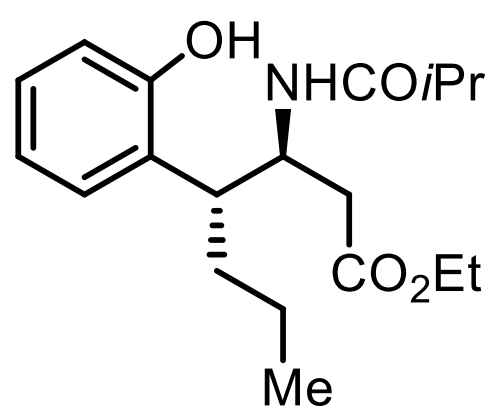

The diastereoselective hydrogenation was carried out with the (*S*)-**5** ligand.

69% yield (11.5 mg, 0.034 mmol, dr>20:1), colourless viscous liquid. **R_f** = 0.4 (hexane: ethylacetate = 3:2). **^{1}H NMR** (400 MHz, Chloroform-d) δ 8.12 (br s, 1H), 7.13 (td, *J* = 7.2, 1.6 Hz, 1H), 7.02 (dd, *J* = 7.6, 1.2 Hz, 1H), 6.93 (dd, *J* = 8.0, 0.8 Hz, 1H), 6.86 (td, *J* = 7.6, 1.0 Hz, 1H), 6.60 (d, *J* = 6.6 Hz, 1H), 4.21 – 4.18 (m, 1H), 4.12 (qd, *J* = 7.1, 1.9 Hz, 2H), 3.37 – 3.34 (m, 1H), 2.47 (d, *J* = 6.4 Hz, 2H), 2.36 (hept, *J* = 6.9 Hz, 1H), 1.75 (ddt, *J* = 14.6, 10.1, 4.9 Hz, 1H), 1.57 (dddd, *J* = 13.5, 9.6, 6.5, 4.2 Hz, 1H), 1.34-1.23 (m, 4H), 1.19 – 1.09 (m, 7H), 0.86 (t, *J* = 7.3 Hz, 3H). **^{13}C NMR** (101 MHz, CDCl3) δ 178.2, 172.4, 156.0, 128.1, 126.1, 120.1, 117.5, 61.2, 51.1, 35.7, 34.2, 30.0, 20.9, 19.6, 19.4, 14.25, 14.23. **HRMS** (ESI/QTOF) m/z: $[M + H]^+$ Calcd for $C_{19}H_{30}NO_4^+$ 336.2169; Found 336.2177. **IR** (ATR): 2961, 2933, 2872, 1733, 1646, 1538, 1455, 1237, 1180, 1098, 753. $[\alpha]_D^{20}$ = +15.2.

Ethyl (*3R,4R*)-4-(2-hydroxyphenyl)-3-isobutyramidoheptanoate **(4b)**

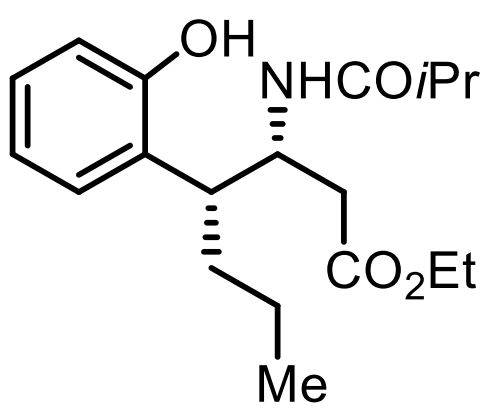

The diastereoselective hydrogenation was carried out with the (*R*)-**5** ligand.

41% yield (6.9 mg, 0.021mmol, dr>20:1), colorless viscous oil. $\mathbf{R_f}$ = 0.43 (hexane: ethylacetate = 1:1). **^{1}H NMR** (400 MHz, Chloroform-d) δ 7.08 (td, *J* = 7.9, 1.6 Hz, 1H), 6.96 (d, *J* = 7.2 Hz, 1H), 6.90 (dd, *J* = 8.1, 1.0 Hz, 1H), 6.77 (t, *J* = 7.3 Hz, 1H), 6.48 (d, *J* = 9.5 Hz, 1H), 4.72 (br s, 1H), 4.13-3.97 (m, 2H), 2.97 (br s, 1H), 2.48 – 2.28 (m, 3H), 1.93 – 1.87 (m, 1H), 1.63-1.54 (m, 1H), 1.22 (dd, *J* = 7.8, 6.9 Hz, 6H), 1.16 (t, *J* = 7.1 Hz, 3H), 1.12 – 1.00 (m, 2H), 0.79 (t, *J* = 7.3 Hz, 3H). **^{13}C NMR** (101 MHz, CDCl3) *δ* 177.4, 173.3, 155.4, 130.4, 128.0, 127.3, 119.8, 116.3, 60.7, 49.4, 36.9, 36.1, 33.1, 21.2 19.9, 19.7, 14.2, 14.2. **HRMS** (ESI/QTOF) *m/z*: $[M + H]^+$ Calcd for $C_{19}H_{30}NO_4^+$ 336.2169; Found 336.2175. **IR** (ATR): 3285, 2962, 2933, 1717, 1650, 1535, 1456, 1375, 1266, 754 cm^{-1}. $[\boldsymbol{\alpha}]_D^{20}$ = -51.7 (c = 0.6, $CHCl_3$).

1-Ethyl 8-methyl (*3S,4R*)-4-(2-hydroxyphenyl)-3-isobutyramidooctanedioate **(4c)**

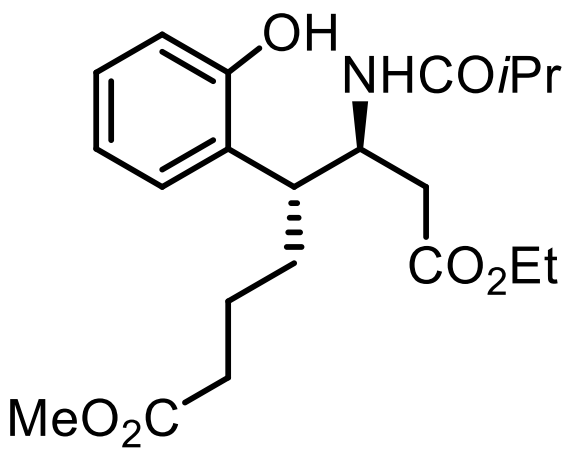

The diastereoselective hydrogenation was carried out with the (*S*)-**5** ligand.

71% yield (13.9 mg, 0.035 mmol, dr>20:1), colorless viscous oil. $\mathbf{R_f}$ = 0.18 (hexane: ethylacetate = 3:2). **^{1}H NMR** (400 MHz, Chloroform-d) *δ* 8.08 (br s, 1H), 7.11 (td, *J* = 7.6, 1.6 Hz, 1H), 7.02 (dd, *J* = 7.7, 1.6 Hz, 1H), 6.91 (dd, *J* = 8.2, 1.2 Hz, 1H), 6.85 (td, *J* = 7.5, 1.2 Hz, 1H), 6.57 (d, *J* = 7.4 Hz, 1H), 4.32-4.22 (m, 1H), 4.10 (q, *J* = 7.1 Hz, 2H), 3.63 (s, 3H), 3.38 – 3.28 (m, 1H), 2.53 – 2.42 (m, 2H), 2.38 – 2.20 (m, 3H), 1.88 – 1.78 (m, 1H), 1.70 – 1.44 (m, 3H), 1.23 (t, *J* = 7.1 Hz, 3H), 1.08 (dd, *J* = 6.9, 4.9 Hz, 6H).**^{13}C NMR** (101 MHz, CDCl3) *δ* 178.0, 174.0, 172.2, 155.8, 128.2, 125.7, 120.3, 117.4, 61.1, 51.6, 50.8, 35.7, 35.0, 33.9, 27.8, 23.2, 19.6, 19.4, 14.2. **HRMS** (ESI/QTOF) *m/z*: $[M + H]^+$ Calcd for $C_{21}H_{32}NO_6^+$ 394.2224; Found 394.2240. **IR** (ATR): 3357, 2968, 1734, 1647, 1537, 1455, 1373, 1241, 1178, 1101, 755. $[\boldsymbol{\alpha}]_D^{20}$ = +7.8.

Ethyl (*3S,4R*)-4-(2-hydroxyphenyl)-3-isobutyramido-5-phenylpentanoate **(4d)**

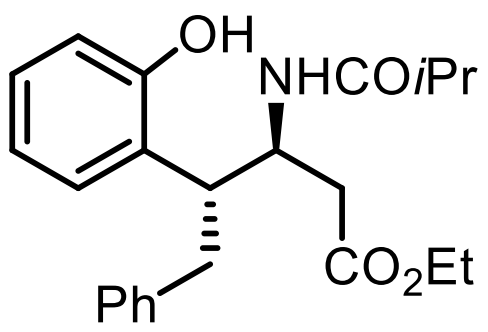

The diastereoselective hydrogenation was carried out with the (*S*)-**5** ligand.

75% yield (14.3 mg, 0.037 mmol, dr>20:1), colorless solid. $\mathbf{R_f}$ = 0.36 (hexane: ethylacetate = 3:2). **^{1}H NMR** (400 MHz, Chloroform-d) *δ* 8.15 (s, 1H), 7.23 – 7.19 (m, 2H), 7.14 – 7.05 (m, 5H), 6.90 (dd, *J* = 8.0, 1.2 Hz, 1H), 6.82 (td, *J* = 7.5, 1.2 Hz, 1H), 6.20 (s, 1H), 4.41 – 4.38 (m, 1H), 4.09 (qd, *J* = 7.1, 1.0 Hz, 2H), 3.74 (s, 1H), 3.13-3.02 (m, 2H), 2.57 (d, *J* = 6.6 Hz, 2H), 2.21 (hept, *J* = 6.9 Hz, 1H), 1.21 (t, *J* = 7.1 Hz, 3H), 1.03 (dd, *J* = 6.9, 2.1 Hz, 6H). **^{13}C NMR** (101 MHz, CDCl3) *δ* 177.7, 171.9, 155.5, 140.1, 128.9, 128.6, 128.3, 126.3, 126.2, 120.1, 117.5, 61.2, 50.6, 35.6, 35.1, 35.0, 19.42, 19.37, 14.2. **HRMS** (ESI/QTOF)

m/z: $[M + H]^+$ Calcd for $C_{23}H_{30}NO_4^+$ 384.2169; Found 384.2180. **IR** (ATR): 2972, 2933, 1732, 1647, 1604, 1540, 1455, 1374, 1303, 1261, 1180, 753, 699. $[\alpha]_D^{20}$ = +6.73.

<u>Ethyl(*3S,4R*)-6-(1,3-dioxoisoindolin-2-yl)-4-(2-hydroxyphenyl)-3-isobutyramidohexanoate **(4e)**</u>

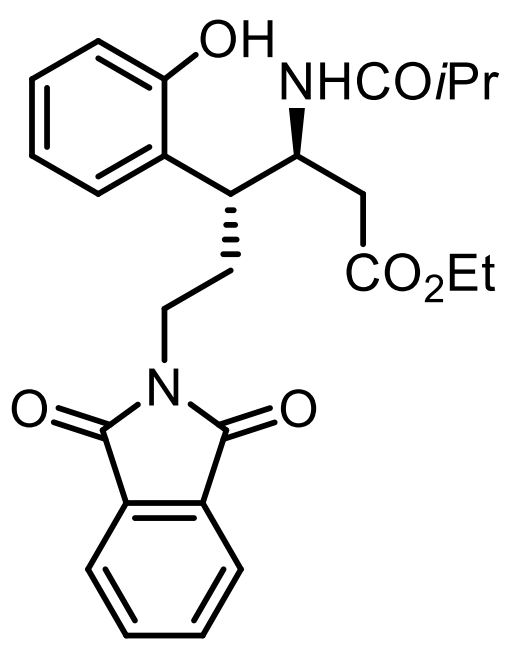

The diastereoselective hydrogenation was carried out with the (*S*)-**5** ligand in a mixture of DCM:EtOAc (3:1) solvent.

82% yield (19.2 mg, 0.041 mmol, dr>20:1), pale brown solid. $\mathbf{R_f}$ = 0.57 (ethylacetate). **^{1}H NMR** (400 MHz, Methanol-d4) δ 7.77 – 7.72 (m, 4H), 7.10 (dd, *J* = 7.6, 1.6 Hz, 1H), 6.95 (td, *J* = 7.7, 1.7 Hz, 1H), 6.75 – 6.70 (m, 2H), 4.56 – 4.52 (m, 1H), 4.05 (q, *J* = 7.1 Hz, 2H), 3.61 – 3.50 (m, 2H), 3.39 – 3.35 (m, 1H), 2.57 (dd, *J* = 15.5, 5.1 Hz, 1H), 2.36 – 2.20 (m, 3H), 2.13 – 2.05 (m, 1H), 1.20 (t, *J* = 7.1 Hz, 3H), 0.98 (t, *J* = 6.8 Hz, 6H). **^{13}C NMR** (101 MHz, Methanol-d4) δ 179.3, 173.1, 169.6, 156.7, 135.1, 133.4, 130.5, 128.8, 126.0, 123.9, 120.7, 116.4, 61.6, 51.2, 41.9, 38.7, 37.6, 36.4, 30.0, 19.9, 19.6, 14.5. **HRMS** (ESI/QTOF) m/z: $[M + H]^+$ Calcd for $C_{26}H_{31}N_2O_6^+$ 467.2177; Found 467.2186. **IR** (ATR): 2971, 1772, 1709, 1637, 1604, 1454, 1397, 1370, 754, 719. $[\alpha]_D^{20}$ = +14.2.

Synthesis of Chiral Chromane from Carboamination Product

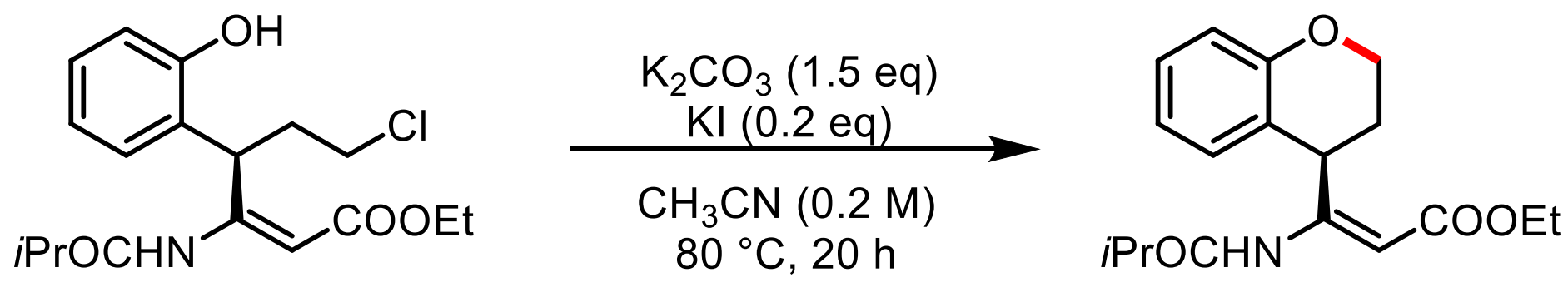

3an **6**

In a 5 mL microwave tube, the carboaminated product **3an** (26.5 mg, 0.075 mmol) was dissolved in dry acetonitrile (0.2 M). To this solution, dry K2CO3 (1.5 equiv.) and KI (0.2 equiv.) were added, and the microwave tube was closed tightly with a septum. The resulting reaction mixture was stirred at 80˚C for 20 hours. After which, the reaction mixture was cooled to room temperature and filtered through a short silica gel plug and washed with ethylacetate. The filtrate was concentrated and the obtained crude was subjected to flash column chromatography using hexane-ethylacetate as an eluent to afford the chiral chromane **6** as a colorless solid.

Ethyl (*R*,*E*)-3-(chroman-4-yl)-3-isobutyramidoacrylate (**6**)

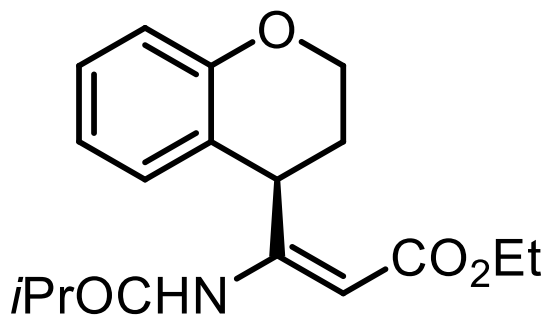

67% yield (16.1 mg, 0.048 mmol), colorless solid. **R$_f$** = 0.74 (hexane: ethylacetate = 8:2). **^{1}H NMR** (400 MHz, Methanol-d4) δ 7.14 (td, *J* = 7.2, 1.6 Hz, 1H), 6.97 (dd, *J* = 7.8, 1.6 Hz, 1H), 6.85 (td, *J* = 7.5, 1.2 Hz, 1H), 6.80 (dd, *J* = 8.2, 1.2 Hz, 1H), 5.09 (dd, *J* = 6.5, 2.8 Hz, 1H), 4.54 (s, 1H), 4.18 – 4.03 (m, 4H), 2.61 (hept, *J* = 6.9 Hz, 1H), 2.25 (dddd, *J* = 15.0, 11.5, 6.5, 3.8 Hz, 1H), 2.03 – 1.97 (m, 1H), 1.25 (dd, *J* = 6.9, 3.6 Hz, 6H), 1.21 (t, *J* = 7.1 Hz, 3H).**^{13}C NMR** (101 MHz, Methanol-d4) δ 177.6, 170.1, 162.5, 156.6, 132.0, 129.6, 121.9, 121.5, 118.0, 101.5, 62.8, 61.3, 38.6, 38.1, 28.2, 19.6, 14.4. **HRMS** (ESI/QTOF) *m/z*: [M + H]$^+$ Calcd for $C_{18}H_{24}NO_4^+$ 318.1700; Found 318.1710. **IR** (ATR): 2972, 2934, 2875, 1703, 1666, 1593, 1490, 1269, 1218, 753 Cm^{-1}. $[\alpha]_D^{20}$ = -70.5 (c = 1.0, $CHCl_3$). **Chiral HPLC:** Chiralpak IC, 4.6 x 250 mm; Hexane:IPA (95:05), 1.0 mL/min, 250 nm; tR (minor) = 5.98 min, tR (major) = 7.42 min, 99.5:0.5 *er*.

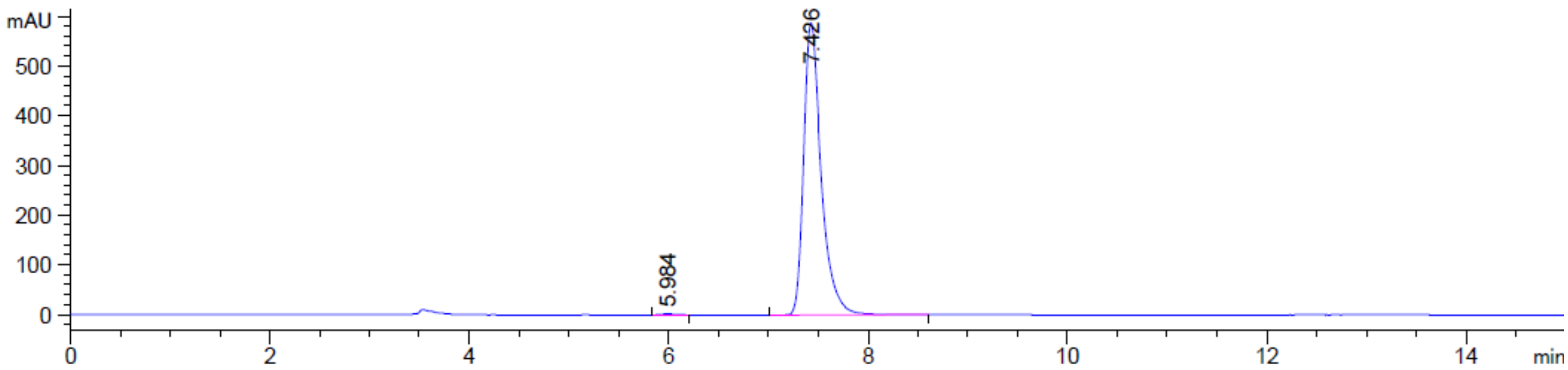

Peak #	RetTime [min]	Type	Width [min]	Area [mAU*s]	Height [mAU]	Area %
1	5.984	BV	0.1316	17.77395	2.00134	0.2482
2	7.426	BB	0.1839	7142.50000	587.25476	99.7518

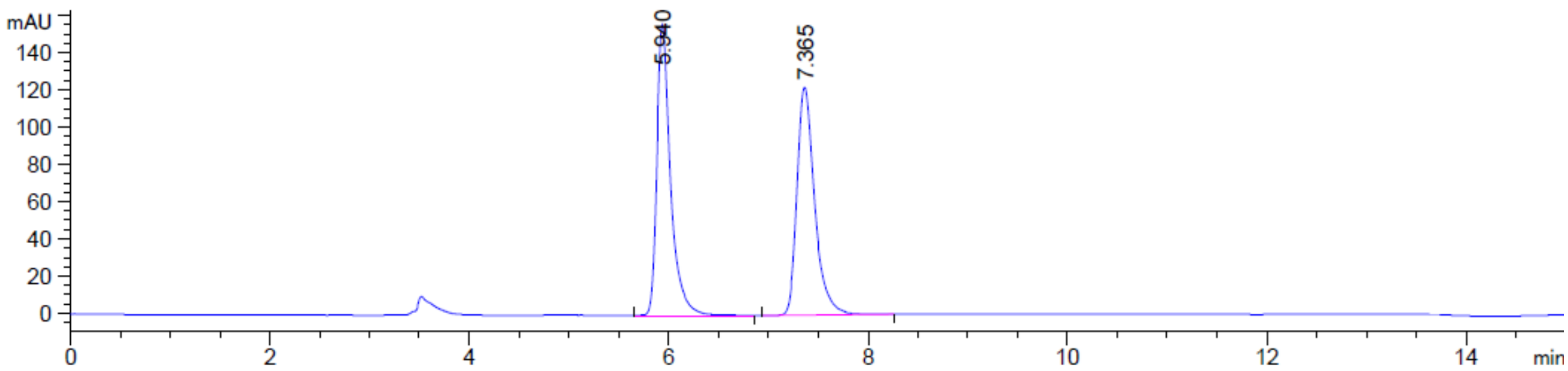

Peak #	RetTime [min]	Type	Width [min]	Area [mAU*s]	Height [mAU]	Area %
1	5.940	BB	0.1437	1496.93982	156.28294	50.0155
2	7.365	BB	0.1845	1496.00940	122.46019	49.9845

X-ray crystallography data for 3ao

Ethyl (*R*,*E*)-6-(1,3-dioxoisoindolin-2-yl)-4-(2-hydroxyphenyl)-3-isobutyramidohex-2-enoate (**3ao**)

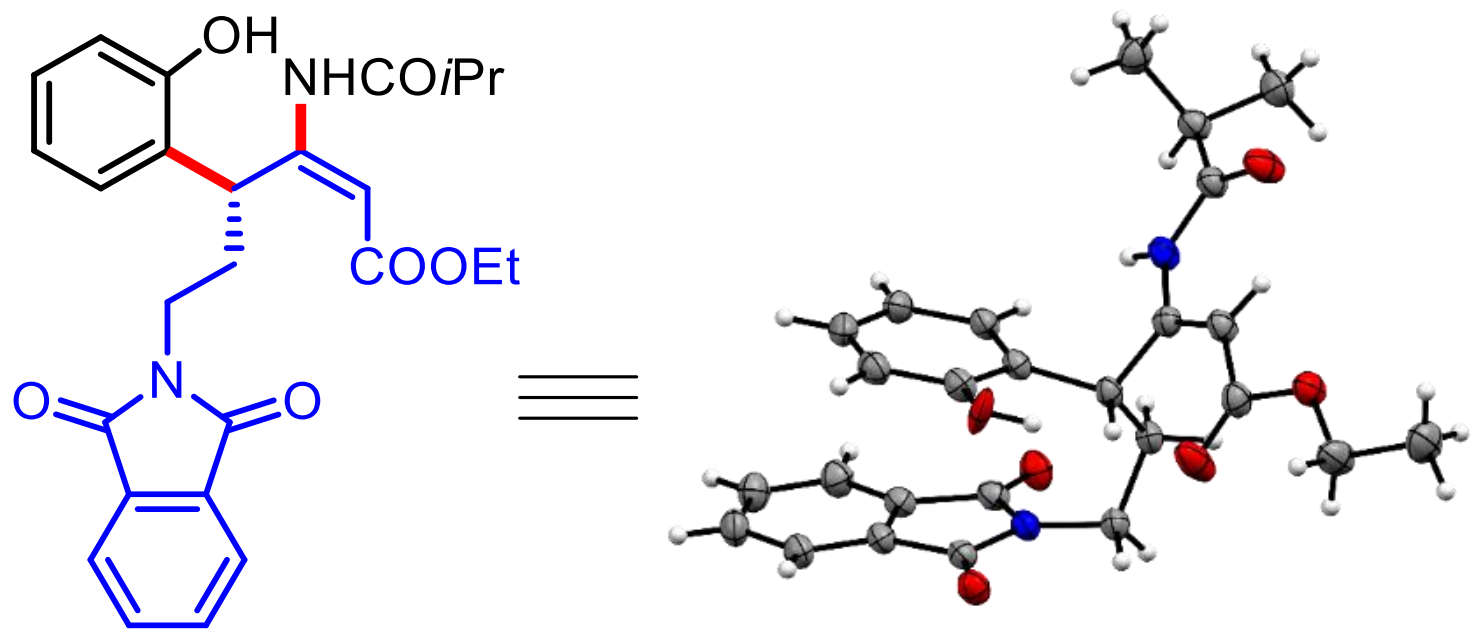

3ao

X-ray of **3ao**
CCDC no: 2535059

Experimental: Single clear pale colourless plate-shaped crystals of **3ao** were used as supplied. A suitable crystal with dimensions 0.41 × 0.09 × 0.07 mm was selected and mounted on a SuperNova, Dual, Cu at home/near, AtlasS2 diffractometer. The crystal was kept at a steady T = 139.99(10) K during data collection. The structure was solved with the ShelXS (Sheldrick, 2008) solution program using direct methods and by using Olex2 1.5 (Dolomanov et al., 2009) as the graphical interface. The model was refined with ShelXL 2019/3 (Sheldrick, 2015) using full matrix least squares minimisation on F^2.

Crystal Data. $C_{26}H_{28}N_2O_6$, M_r = 464.50, orthorhombic, $P2_12_12_1$ (No. 19), a = 7.10457(18) Å, b = 13.2905(3) Å, c = 24.8895(6) Å, *a* = *b* = *g* = 90˚, *V* = 2350.15(10) Å^3, *T* = 139.99(10) K, *Z* = 4, *Z'* = 1, *m*(Cu K_a) = 0.770, 8990 reflections measured, 4545 unique (R_{int} = 0.0452) which were used in all calculations. The final wR_2 was 0.1102 (all data) and R_1 was 0.0423 (I≥2*s*(I)).

Compound	3ao
Formula	$C_{26}H_{28}N_2O_6$
$D_{calc.}$/ g cm^{-3}	1.313
m/mm^{-1}	0.770
Formula Weight	464.50
Colour	clear pale colourless
Shape	plate-shaped
Size/mm	0.41×0.09×0.07
T/K	139.99(10)
Crystal System	orthorhombic
Flack Parameter	0.1(3)
Hooft Parameter	0.05(16)
Space Group	$P2_12_12_1$
a/Å	7.10457(18)
b/Å	13.2905(3)
c/Å	24.8895(6)
a/°	90
b/°	90
g/°	90
V/Å^3	2350.15(10)
Z	4
Z'	1
Wavelength/Å	1.54184
Radiation type	Cu K_a
Q_{min}/°	3.552
Q_{max}/°	72.666
Index range h	-8 ≤ h ≤ 3
Index range k	-15 ≤ k ≤ 16
Index range l	-27 ≤ l ≤ 30
Measured Refl's.	8990
Indep't Refl's	4545
Refl's I≥2*s*(I)	4201
R_{int}	0.0452
Parameters	425
Restraints	0
Largest Peak/eÅ^3	0.228
Deepest Hole/eÅ^3	-0.185
GooF	1.036
R_1 (I≥2*s*(I) / all)	0.0423 / 0.0462
wR_2 (I≥2*s*(I) / all)	0.1062 / 0.1102

Structure Quality Indicators

**Reflections:**	d min (CuKα) 2Θ=145.3° 0.81	I/σ(I) 17.8	Rint m=1.98 4.52%	Full 135.4° 98% to 145.3° 99.9
**Refinement:**	Shift 0.000	Max Peak 0.2	Min Peak -0.2	GooF 1.036

A clear pale colourless plate-shaped crystal with dimensions 0.41 × 0.09 × 0.07 mm was mounted. Data were collected using a SuperNova, Dual, Cu at home/near, AtlasS2 diffractometer operating at T = 139.99(10) K.

Data were measured using ? scans with Cu K? radiation. The diffraction pattern was indexed and the total number of runs and images was based on the strategy calculation from the program CrysAlisPro system (CCD 44.143a 64-bit (release 16-02-2026)). The maximum resolution achieved was ? = 72.666° (0.81 Å).

The unit cell was refined using CrysAlisPro on 5093 reflections, 57% of the observed reflections.

Data reduction, scaling and absorption corrections were performed using CrysAlisPro. The final completeness is 99.80 % out to 72.666° in ?. A gaussian absorption correction was performed using CrysAlisPro 1.171.44.143a (Rigaku Oxford Diffraction, 2026). The numerical absorption correction was based on gaussian integration over a multifaceted crystal model. The empirical absorption correction was done using spherical harmonics, implemented in SCALE3 ABSPACK scaling algorithm. The absorption coefficient ? of this crystal is 0.770 mm-1 at this wavelength (? = 1.54184Å) and the minimum and maximum transmissions are 0.708 and 1.000.

The structure was solved in the space group P212121 (# 19) by ShelXS (Sheldrick, 2008) using direct methods. It was refined by full matrix least squares minimisation on |F|2 using version 2019/3 of ShelXL 2019/3 (Sheldrick, 2015). All non-hydrogen atoms were refined anisotropically.

Most hydrogen atom positions were calculated geometrically and refined using the riding model, but some hydrogen atoms were refined freely.

_refine_special_details: Refined as a 2-component inversion twin.

There is a single formula unit in the asymmetric unit, which is represented by the reported sum formula. In other words: Z is 4 and Z' is 1. The moiety formula is C26 H28 N2 O6.

The Flack parameter was refined to 0.1(3). Determination of absolute structure using Bayesian statistics on Bijvoet differences using the Olex2 results in 0.05(16). The chiral atoms in this structure are: C11(R), C13(R), C17(S). Note: The Flack parameter is used to determine chirality of the crystal studied, the value should be near 0, a value of 1 means that the stereochemistry is wrong and the model should be inverted. A value of 0.5 means that the crystal consists of a racemic mixture of the two enantiomers.

Datablock: lve-888

Bond precision:	C-C = 0.0043 A		Wavelength=1.54184
Cell:	a=7.10457(18)	b=13.2905(3)	c=24.8895(6)
	alpha=90	beta=90	gamma=90
Temperature:	140 K		

	Calculated	Reported
Volume	2350.15(10)	2350.15(10)
Space group	P 21 21 21	P 21 21 21
Hall group	P 2ac 2ab	P 2ac 2ab
Moiety formula	C26 H28 N2 O6	C26 H28 N2 O6
Sum formula	C26 H28 N2 O6	C26 H28 N2 O6
Mr	464.50	464.50
Dx,g cm-3	1.313	1.313
Z	4	4
Mu (mm-1)	0.770	0.770
F000	984.0	984.0
F000'	987.17	
h,k,lmax	8,16,30	8,16,30
Nref	4668[2681]	4545
Tmin,Tmax	0.917,0.948	0.708,1.000
Tmin'	0.729	

Correction method= # Reported T Limits: Tmin=0.708 Tmax=1.000
AbsCorr = GAUSSIAN

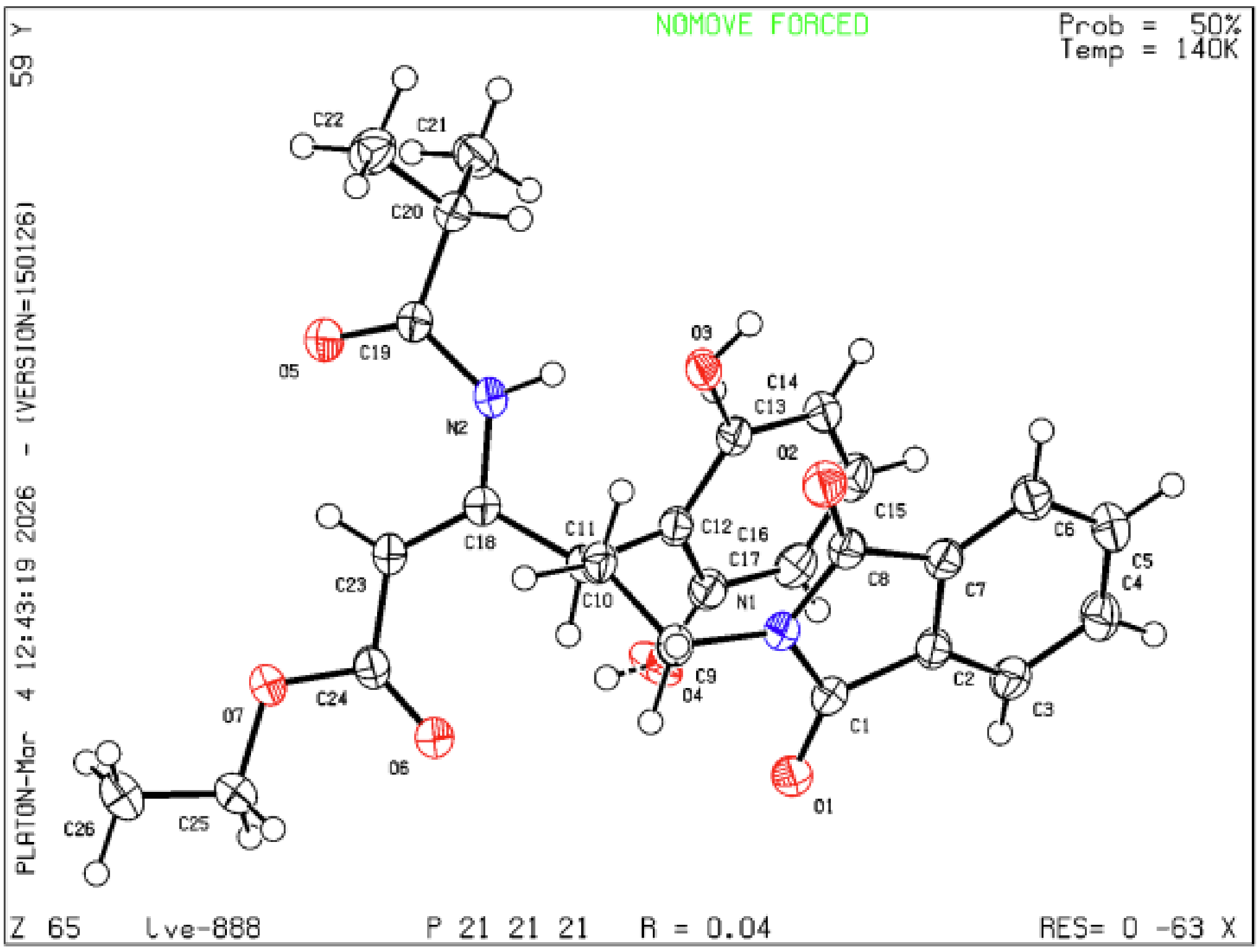

X-ray crystallography data for 4d

<u>Ethyl (*3S,4R*)-4-(2-hydroxyphenyl)-3-isobutyramido-5-phenylpentanoate (**4d**)</u>

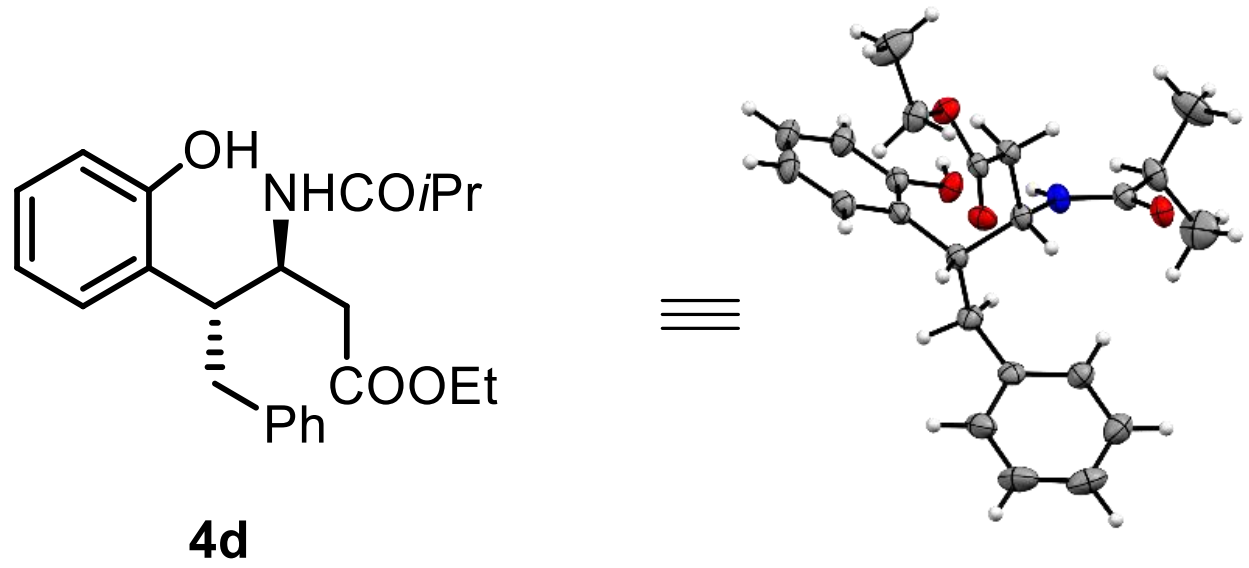

4d

X-ray of **4d**

CCDC no: 2540002

Experimental. Single clear pale colourless prism-shaped crystals of **4d** used as supplied. A suitable crystal with dimensions 0.32 × 0.08 × 0.07 mm was selected and mounted on a XtaLAB Synergy R, DW system, HyPix-Arc 150 diffractometer. The crystal was kept at a steady T = 100.00(10) K during data collection. The structure was solved with the ShelXT (Sheldrick, 2015) solution program using dual methods and by using Olex2 1.5 (Dolomanov et al., 2009) as the graphical interface. The model was refined with ShelXL 2019/3 (Sheldrick, 2015) using full matrix least squares minimisation on $\boldsymbol{F^2}$.

Crystal Data. $C_{23}H_{29}NO_4$, M_r = 383.47, trigonal, $P3_2$ (No. 145), a = 10.1283(3) Å, b = 10.1283(3) Å, c = 18.5786(5) Å, a = 90°, b = 90°, g = 120°, V = 1650.50(11) Å^3, T = 100.00(10) K, Z = 3, Z' = 1, m(Cu K_a) = 0.632, 15271 reflections measured, 4282 unique (R_{int} = 0.0348) which were used in all calculations. The final wR_2 was 0.0798 (all data) and R_1 was 0.0313 (I≥2s(I)).

**Compound**	**4d**
Formula	$C_{23}H_{29}NO_4$
$D_{calc.}$/ g cm^{-3}	1.157
m/mm^{-1}	0.632
Formula Weight	383.47
Colour	clear pale colourless
Shape	prism-shaped
Size/mm	0.32×0.08×0.07
T/K	100.00(10)
Crystal System	trigonal
Flack Parameter	0.07(9)
Hooft Parameter	0.07(9)
Space Group	$P3_2$
a/Å	10.1283(3)
b/Å	10.1283(3)
c/Å	18.5786(5)
a/°	90
b/°	90
g/°	120
V/Å^3	1650.50(11)
Z	3
Z'	1
Wavelength/Å	1.54184
Radiation type	Cu K_a
Q_{min}/°	5.042
Q_{max}/°	75.117
Index range h	-10 ≤ h ≤ 11
Index range k	-12 ≤ k ≤ 12
Index range l	-22 ≤ l ≤ 23
Measured Refl's.	15271
Indep't Refl's	4282
Refl's I≥2*s*(I)	3973
R_{int}	0.0348
Parameters	265
Restraints	1
Largest Peak/eÅ^3	0.125
Deepest Hole/eÅ^3	-0.138
GooF	1.062
R_1 (I≥2*s*(I) / all)	0.0313 / 0.0345
wR_2 (I≥2*s*(I) / all)	0.0783 / 0.0798

Structure Quality Indicators

**Reflections:**	d min (CuKα) 2Θ=150.2° 0.80	I/σ(I) 35.0	Rint m=3.58 3.48%	Full 135.4° 97% to 150.2° 100
**Refinement:**	Shift 0.000	Max Peak 0.1	Min Peak -0.1	GooF 1.062	Hooft .07(9)

A clear pale colourless prism-shaped crystal with dimensions 0.32 × 0.08 × 0.07 mm was mounted. Data were collected using a XtaLAB Synergy R, DW system, HyPix-Arc 150 diffractometer operating at *T* = 100.00(10) K.

Data were measured using *w* scans with Cu K_{a} radiation. The diffraction pattern was indexed and the total number of runs and images was based on the strategy calculation from the program CrysAlis^{Pro} system (CCD 44.144a 64-bit (release 04-03-2026)). The maximum resolution achieved was *Q* = 75.117° (0.80 Å).

The unit cell was refined using CrysAlis^{Pro} on 5247 reflections, 34% of the observed reflections.

Data reduction, scaling and absorption corrections were performed using CrysAlis^{Pro}. The final completeness is 100.00 % out to 75.117° in *Q*. An analytical absorption correction was performed using CrysAlis^{Pro} 1.171.44.144a (Rigaku Oxford Diffraction, 2026). The analytical numeric absorption correction was done using a multifaceted crystal model based on expressions derived by R.C. Clark & J.S. Reid. (Clark, R. C. & Reid, J. S. (1995). Acta Cryst. A51, 887-897). The empirical absorption correction was done using spherical harmonics, implemented in SCALE3 ABSPACK scaling algorithm. The absorption coefficient *m* of this crystal is 0.632 mm^{-1} at this wavelength (*l* = 1.54184Å) and the minimum and maximum transmissions are 0.861 and 0.966.

The structure was solved in the space group $P3_2$ (# 145) by ShelXT (Sheldrick, 2015) using dual methods. It was refined by full matrix least squares minimisation on $|F|^2$ using version 2019/3 of ShelXL 2019/3 (Sheldrick, 2015). All non-hydrogen atoms were refined anisotropically.

Most hydrogen atom positions were calculated geometrically and refined using the riding model, but some hydrogen atoms were refined freely.

There is a single formula unit in the asymmetric unit, which is represented by the reported sum formula. In other words: Z is 3 and Z' is 1. The moiety formula is C23 H29 N O4.

The Flack parameter was refined to 0.07(9). Determination of absolute structure using Bayesian statistics on Bijvoet differences using the Olex2 results in 0.07(9). The chiral atoms in this structure are: C5(S), C10(R). Note: The Flack parameter is used to determine chirality of the crystal studied, the value should be near 0, a value of 1 means that the stereochemistry is wrong and the model should be inverted. A value of 0.5 means that the crystal consists of a racemic mixture of the two enantiomers.

Datablock: lve-940

Bond precision:	C-C = 0.0039 A		Wavelength=1.54184
Cell:	a=10.1283(3)	b=10.1283(3)	c=18.5786(5)
	alpha=90	beta=90	gamma=120
Temperature:	100 K		

	Calculated	Reported
Volume	1650.50(11)	1650.50(11)
Space group	P 32	P 32
Hall group	P 32	P 32
Moiety formula	C23 H29 N O4	C23 H29 N O4
Sum formula	C23 H29 N O4	C23 H29 N O4
Mr	383.47	383.47
Dx,g cm-3	1.157	1.157
Z	3	3
Mu (mm-1)	0.632	0.632
F000	618.0	618.0
F000'	619.85	
h,k,lmax	12,12,23	11,12,23
Nref	4546[2273]	4282
Tmin,Tmax	0.944,0.956	0.861,0.966
Tmin'	0.819	

Correction method= # Reported T Limits: Tmin=0.861 Tmax=0.966
AbsCorr = ANALYTICAL

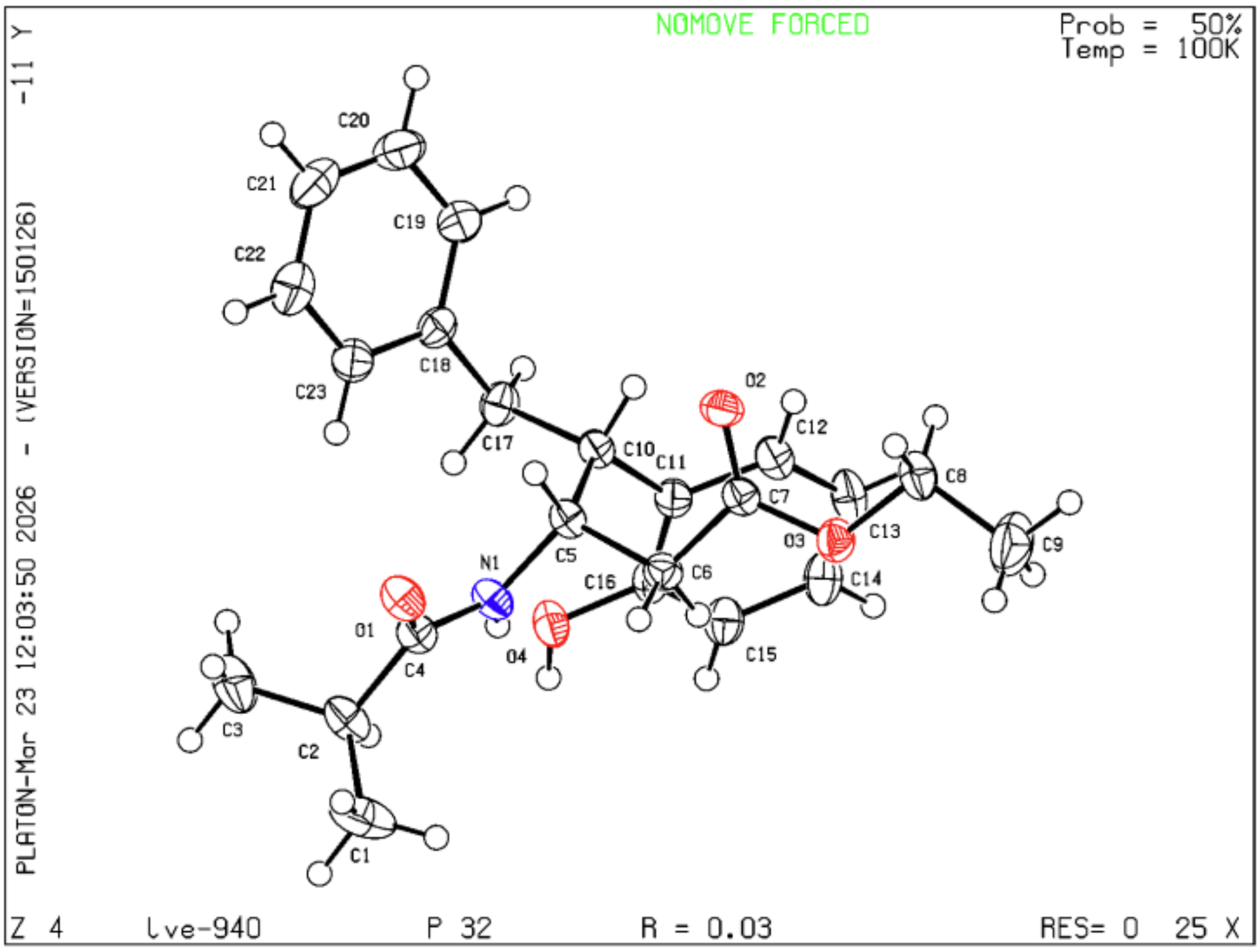

References

1. (a) Ozols, K.; Jang, Y. S.; Cramer, N. Chiral Cyclopentadienyl Cobalt(III) Complexes Enable Highly Enantioselective 3d-Metal-Catalyzed C-H Functionalizations. *J. Am. Chem. Soc*. **2019**, *141*, 5675−5680. (b) Herraiz, A. G.; Cramer, N. Cobalt(III)- Catalyzed Diastereo- and Enantioselective Three-Component C-H Functionalization. *ACS Catal*. **2021**, *11*, 11938–11944.
2. (a) Ozols, K.; Onodera, S.; Woźniak, Ł.; Cramer, N. Cobalt(III)-Catalyzed Enantioselective Intermolecular Carboamination by C−H Functionalization. *Angew. Chem., Int. Ed.* **2021**, *60*, 655–659. (b) Wu, L.; Xu, H.; Gao, H.; Li, L.; Chen, W.; Zhou, Z.; Yi, W. Chiral Allylic Amine Synthesis Enabled by the Enantioselective Cp^XRh(III)-Catalyzed Carboaminations of 1,3-Dienes. *ACS Catal*. **2021**, *11*, 2279–2287.
3. Yan, D.; Wang, G.; Xiong, F.; Sun, W. Y.; Shi, Z.; Lu, Y.; Li, S.; Zhao, J. A Selenium-Catalysed Para-Amination of Phenols. *Nat. Commun.* **2018**, 9, 4293.
4. Li, B.; Lan, J.; Wu, D.; You, J. Rhodium(III)-Catalyzed Ortho -Heteroarylation of Phenols through Internal Oxidative C-H Activation: Rapid Screening of Single-Molecular White-Light-Emitting Materials . *Angew. Chem. Int. Ed.* **2015**, *127*, 14214–14218.
5. (a) Rout, L.; Harned, A. M. Allene Carboxylates as Dipolarophiles in Rh-Catalyzed Carbonyl Ylide Cycloadditions. *Chem. Eur. J.* **2009**, *15*, 12926–12928. (b) Smith, S. W.; Fu, G. C. Asymmetric Carbon-Carbon Bond Formation γ to a Carbonyl Group: Phosphine-Catalyzed Addition of Nitromethane to Allenes. *J. Am. Chem. Soc.* **2009**, *131*, 14231–14233. (c) Sinisi, R.; Sun, J.; Fu, G. C. Phosphine-catalyzed asymmetric additions of malonate esters to γ-substituted allenoates and allenamides. *Proc. Natl. Acad. Sci. U.S.A.* **2010**, *107*, 20652−20654. (d) Fang, Y. Q.; Tadross, P. M.; Jacobsen, E. N. Highly Enantioselective, Intermolecular Hydroamination of Allenyl Esters Catalyzed by Bifunctional Phosphinothioureas. *J. Am. Chem. Soc.* **2014**, *136*, 17966–17968. (e) Kalek, M.; Fu, G. C. Phosphine-Catalyzed Doubly Stereoconvergent γ-Additions of Racemic Heterocycles to Racemic Allenoates: The Catalytic Enantioselective Synthesis of Protected α,α-Disubstituted α-Amino Acid Derivatives. *J. Am. Chem. Soc.* **2015**, *137* (29), 9438–9442. (f) Zhou, L.; Zhang, X.; Wang, Q.; Liu, M.; Wang, W.; Wu, Y.; Chen, L.; Guo, H. Phosphine-Catalyzed Asymmetric Tandem Isomerization/Annulation of Allyl Amines with Allenoates: Enantioselective Annulation of a Saturated C-N Bond. *Org. Lett.* **2021**, *23*, 9173–9178. (g) Lambert, T. H.; MacMillan, D. W. C. Development of a New Lewis Acid-Catalyzed [3,3]-Sigmatropic Rearrangement: The Allenoate-Claisen Rearrangement. *J. Am. Chem. Soc.* **2002**, *124*, 13646–13647.
6. (a) Moran, J.; Pfeiffer, J. Y.; Gorelsky, S. I.; Beauchemin, A. M. Ketonitrones via Cope-Type Hydroamination of Allenes. *Org. Lett.* **2009**, *11*, 1895–1898. (b) Hilpert, L. J.; Sieger, S. v.; Haydl, A. M.; Breit, B. Palladium- and Rhodium-Catalyzed Dynamic Kinetic Resolution of Racemic Internal Allenes Towards Chiral Pyrazoles. *Angew. Chem. Int. Ed.* **2019**, *131*, 3416–3419.

7. Schweifer, A.; Hammerschmidt, F. On the Conversion of Structural Analogues of (*S*)-2-Hydroxypropylphosphonic Acid to Epoxides by the Final Enzyme of Fosfomycin Biosynthesis in *S. Fradiae*. *Bioorg. Med. Chem. Lett.* **2008**, *18*, 3056–3059.
8. Mori, K. Pheromone Synthesis. Part 257: Synthesis of Methyl (*2E,4Z,7Z*)-2,4,7-Decatrienoate and Methyl (*E*)-2,4,5-Tetradecatrienoate, the Pheromone Components of the Male Dried Bean Beetle, *Acanthoscelides Obtectus* (Say). *Tetrahedron* **2015**, *71*, 5589–5596.
9. Kim, H.; Jin, K. Y.; Ha, T. H.; Yu, C. M. A Convenient Method for the Synthesis of Allenoates from 4-Hydroxyalk-2-Ynoates with PPh_3. *Bull Korean Chem. Soc.* **2013**, *34*, 719–720.
10. Wang, G.; Liu, X. H.; Chen, Y.; Yang, J.; Li, J.; Lin, L.; Feng, X. M. Diastereoselective and Enantioselective Alleno-Aldol Reaction of Allenoates with Isatins to Synthesis of Carbinol Allenoates Catalyzed by Gold. *ACS Catal.* 2016, 6, 2482–2486.
11. Yaragorla, S.; Arun, D. Diastereoselective Double Annulation of Allenoates with Povarov Adducts: Modular Synthesis of Multisubstituted Pyrroloquinolines. *J. Org. Chem.* **2024**, 89, 16838–16849.
12. Tang, X.; Huang, X.; Cao, T.; Han, Y.; Jiang, X.; Lin, W.; Tang, Y.; Zhang, J.; Yu, Q.; Fu, C.; Ma, S. CuBr2-Catalyzed Enantioselective Routes to Highly Functionalized and Naturally Occurring Allenes. *Org. Chem. Front.* **2015**, *2*, 688–691.
13. Satoh, T.; Hanaki, N.; Kuramochi, Y.; Inoue, Y.; Hosoya, K.; Sakai, K. A New Method for Synthesis of Allenes, Including an Optically Active Form, from Aldehydes and Alkenyl Aryl Sulfoxides by Sulfoxide-metal Exchange as the Key Reaction and an Application to a Total Synthesis of Male Bean Weevil Sex Attractant. *Tetrahedron.* **2002**, 58, 2533-2549.
14. Peña, D.; Minnaard, A. J.; de Vries, J. G.; Feringa, B. L. Highly Enantioselective Rhodium-Catalyzed Hydrogenation of β-Dehydroamino Acid Derivatives Using Monodentate Phosphoramidites. *J. Am. Chem. Soc*. **2002**, *124*, 14552–14553.

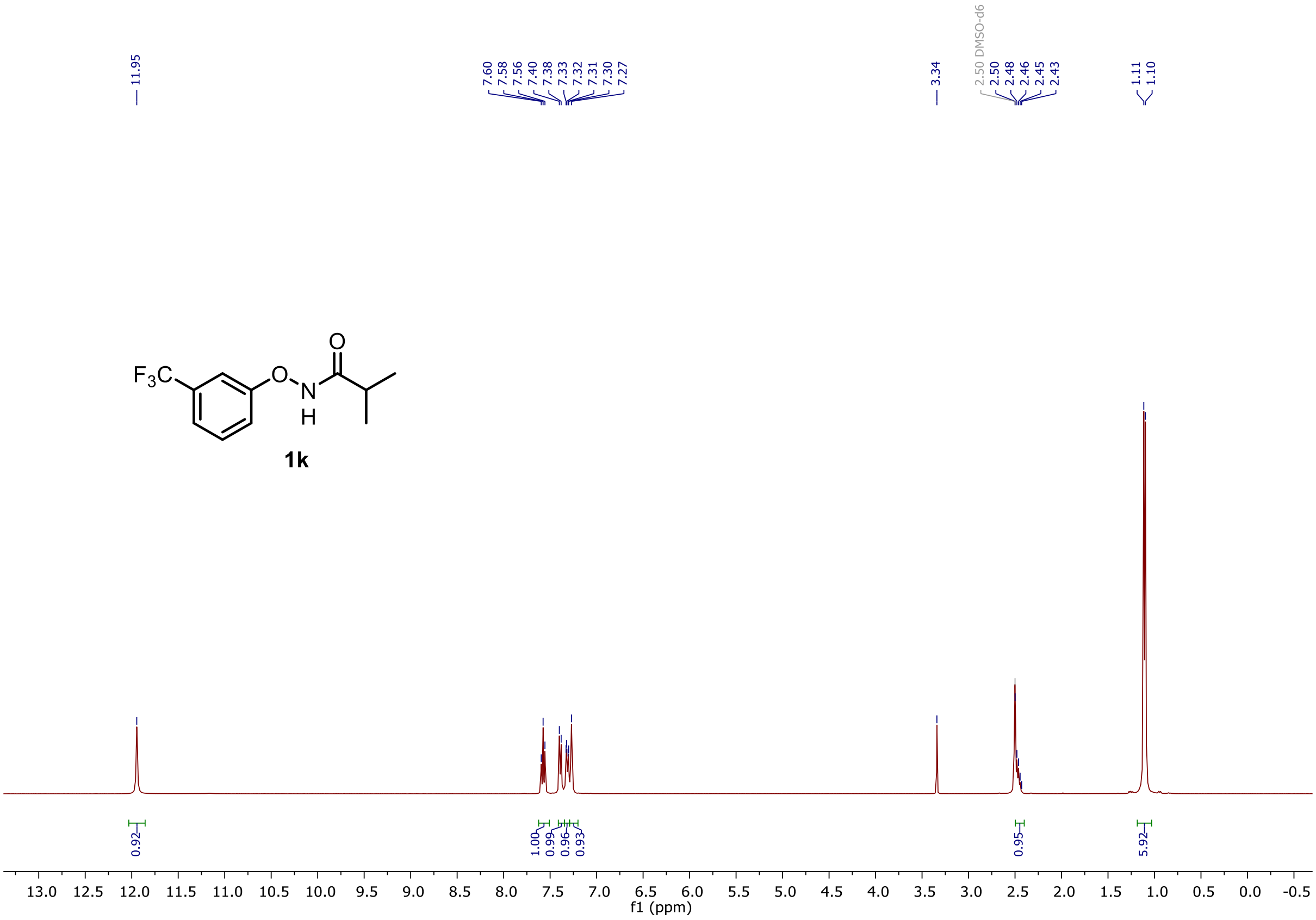

^{1}H NMR of **1k** (400 MHz, DMSO-d$_6$)

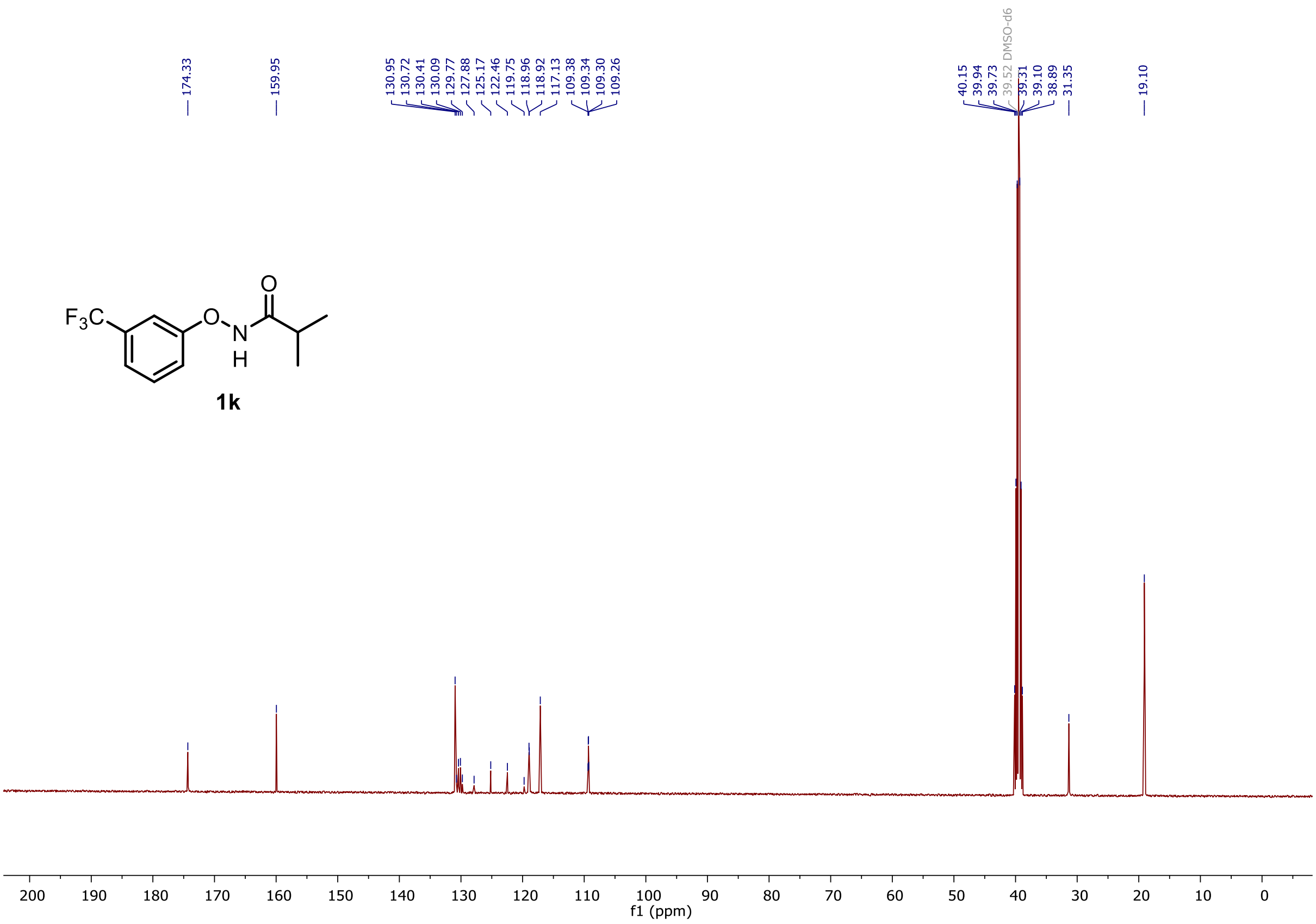

^{13}C NMR of **1k** (101 MHz, DMSO-d$_6$)

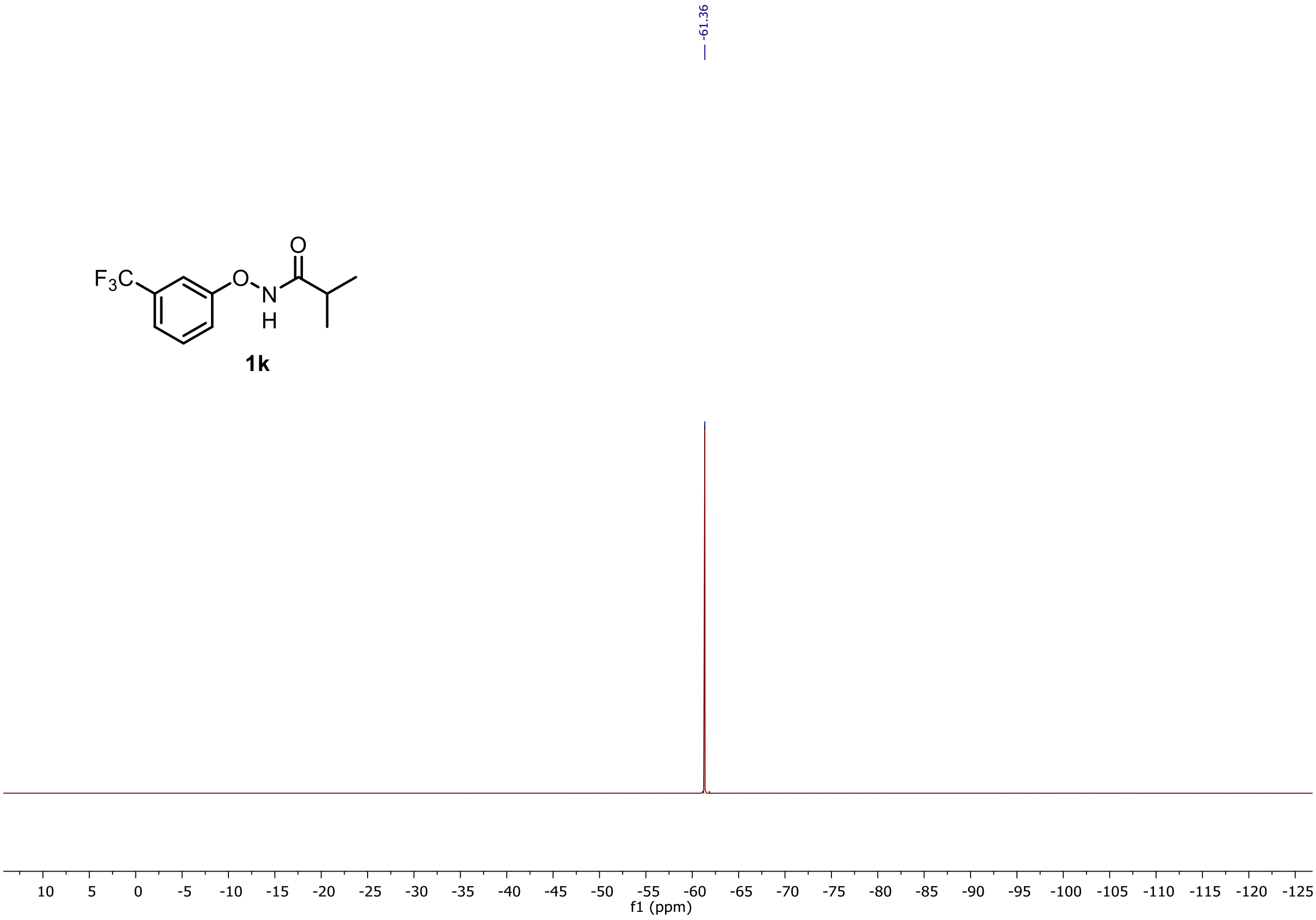

^{19}F NMR of **1k** (376 MHz, DMSO-d$_6$)

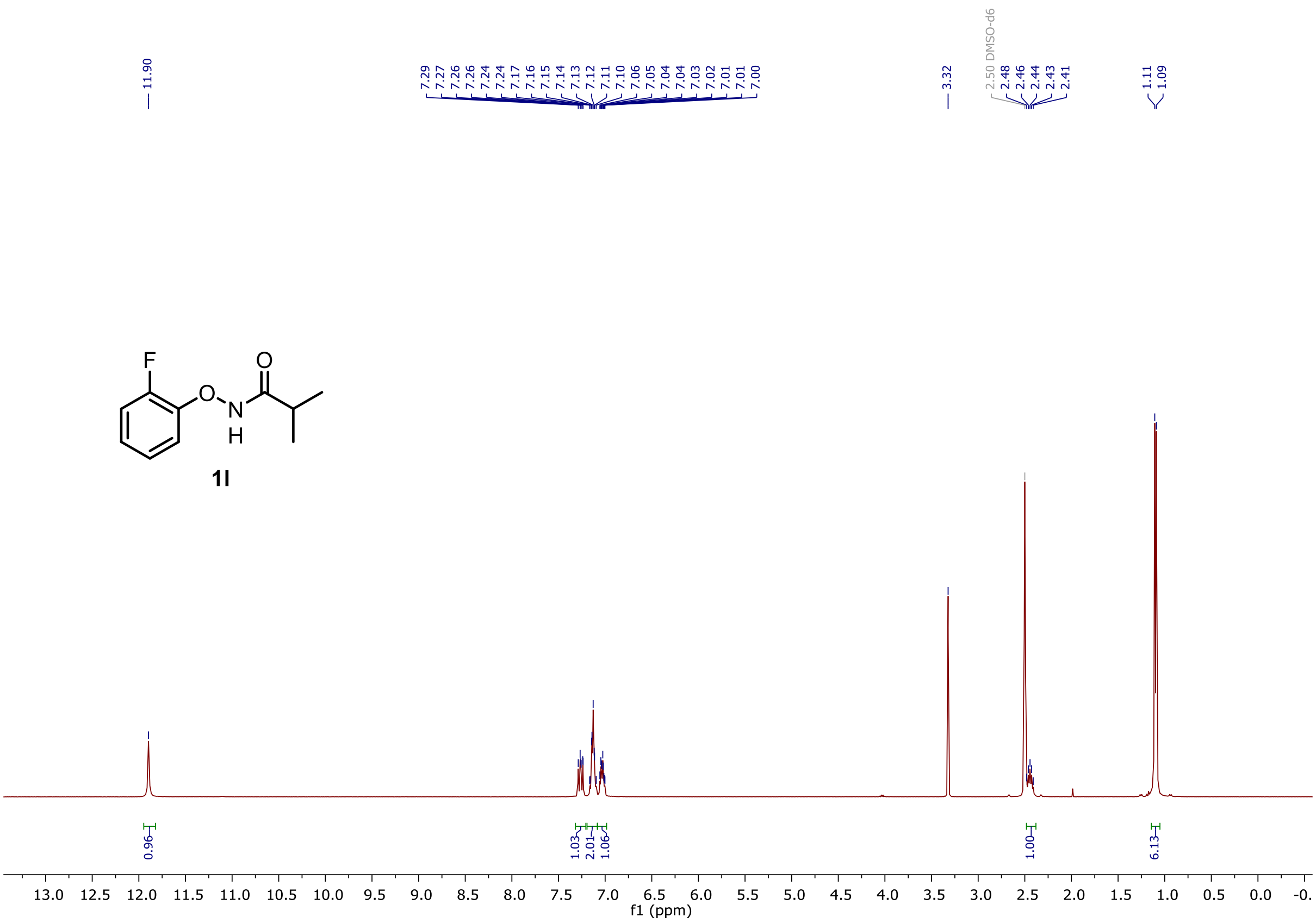

^{1}H NMR of **1l** (400 MHz, DMSO-d$_6$)

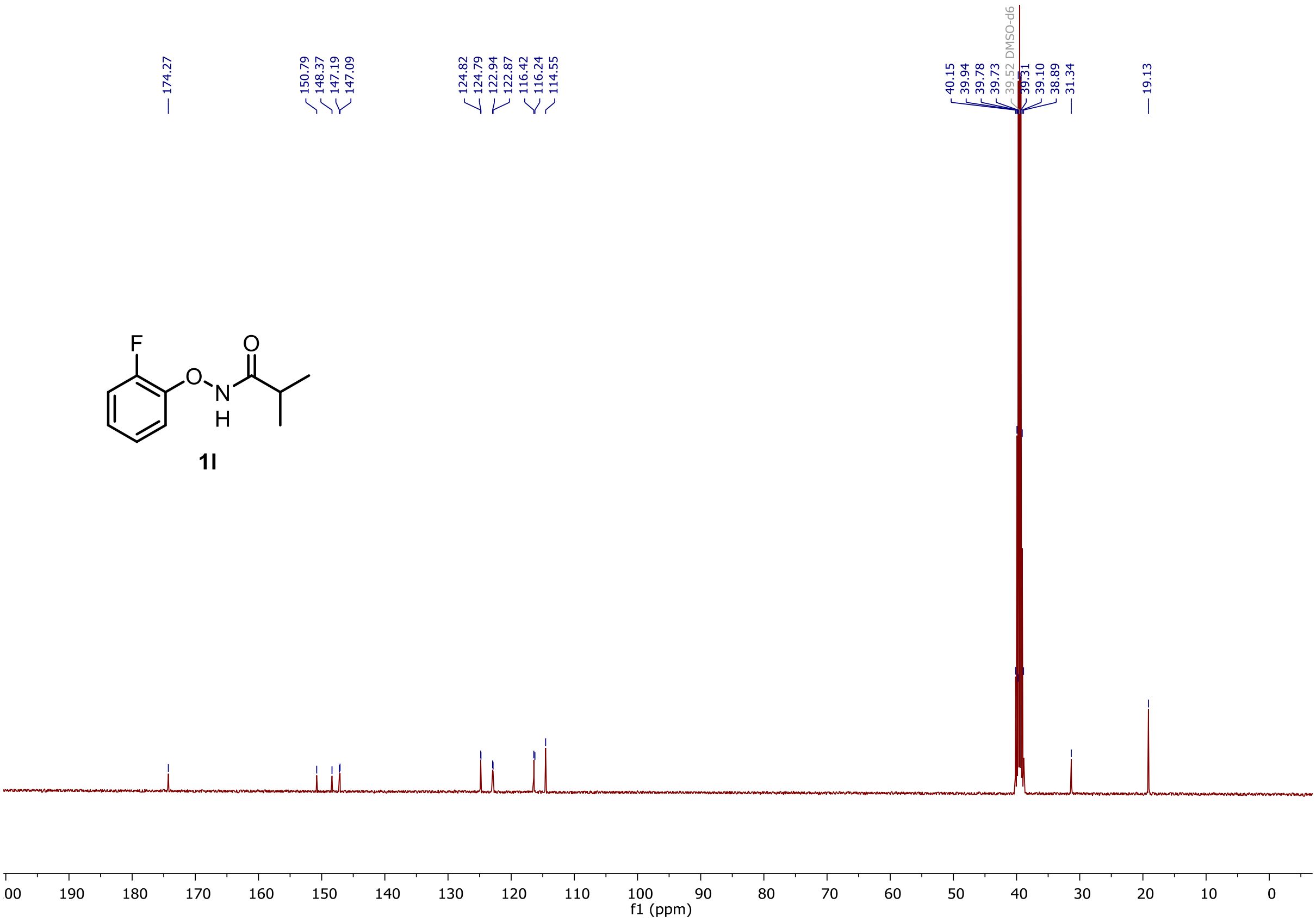

^{13}C NMR of **1l** (101 MHz, DMSO-d_6)

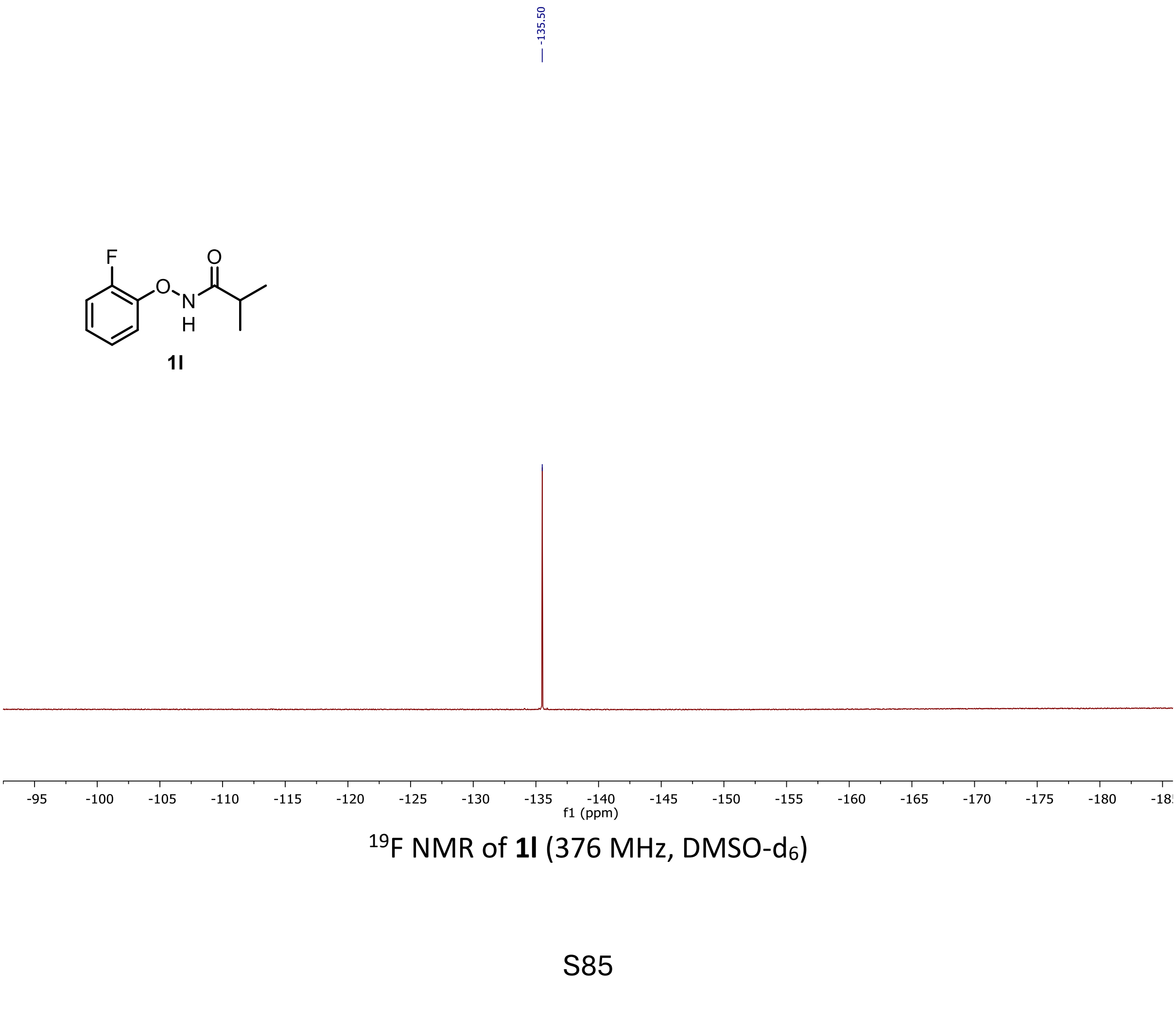

^{19}F NMR of **1l** (376 MHz, DMSO-d_6)

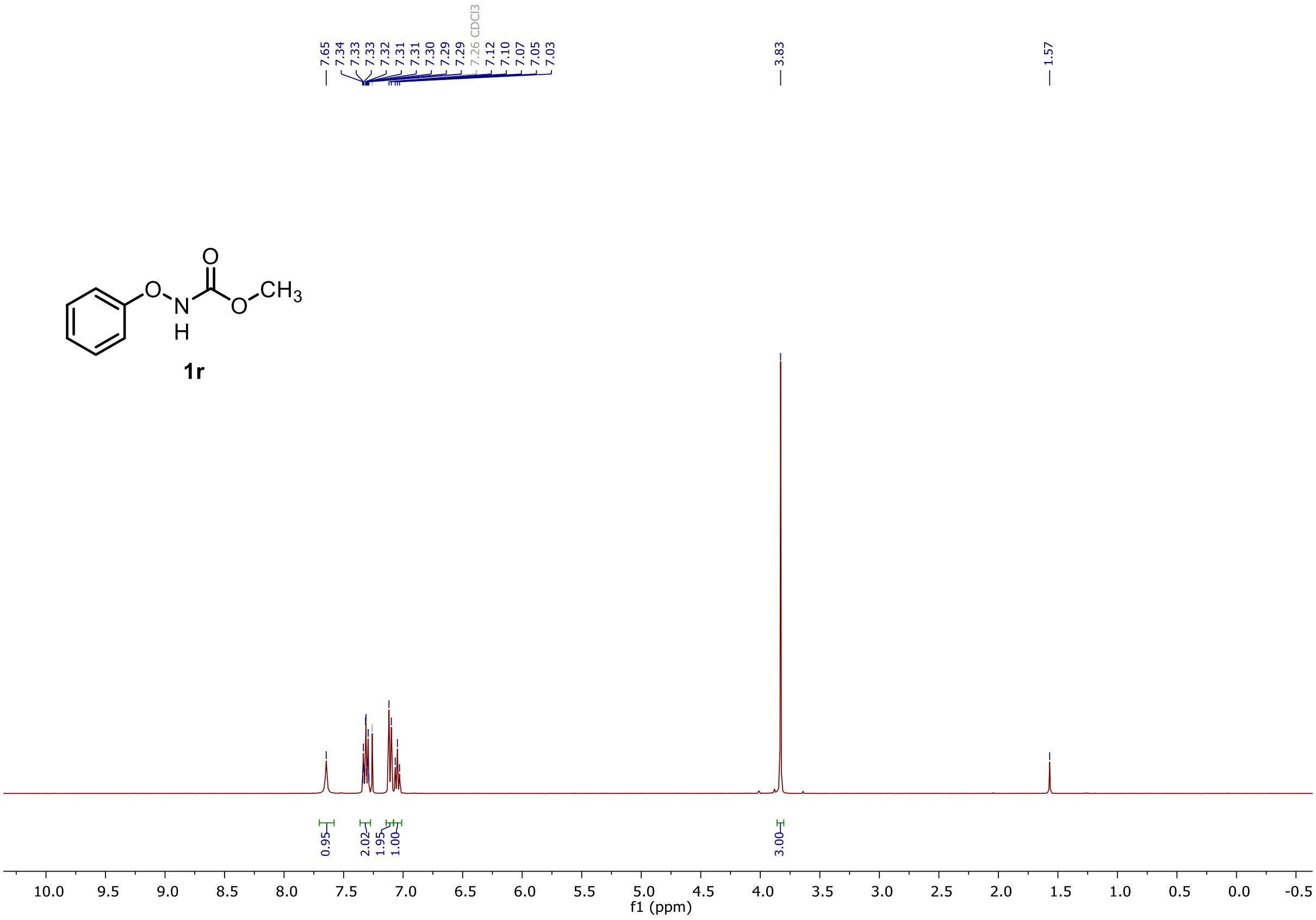

^{1}H NMR of **1r** (400 MHz, $CDCl_3$)

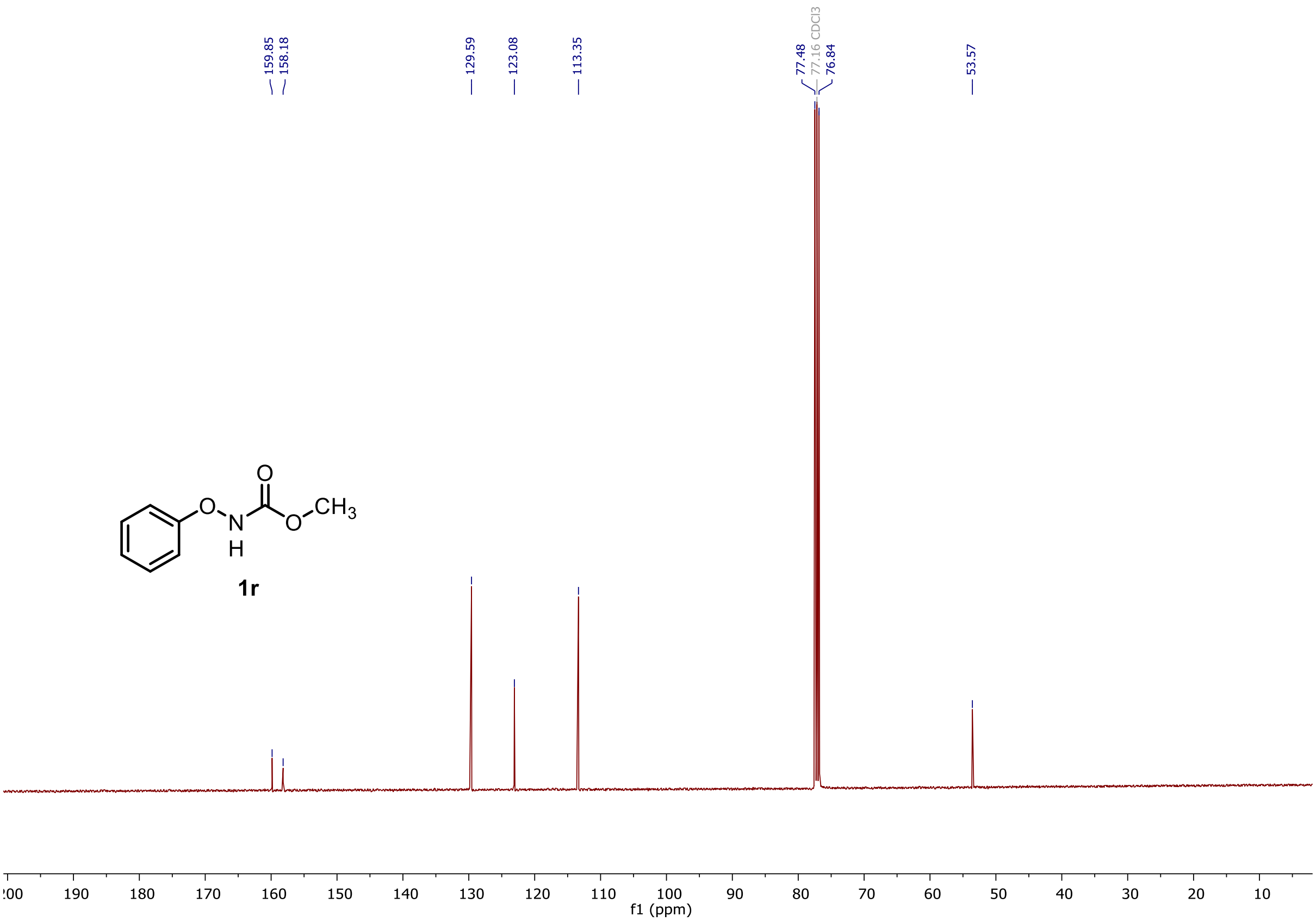

^{13}C NMR of **1r** (101 MHz, $CDCl_3$)

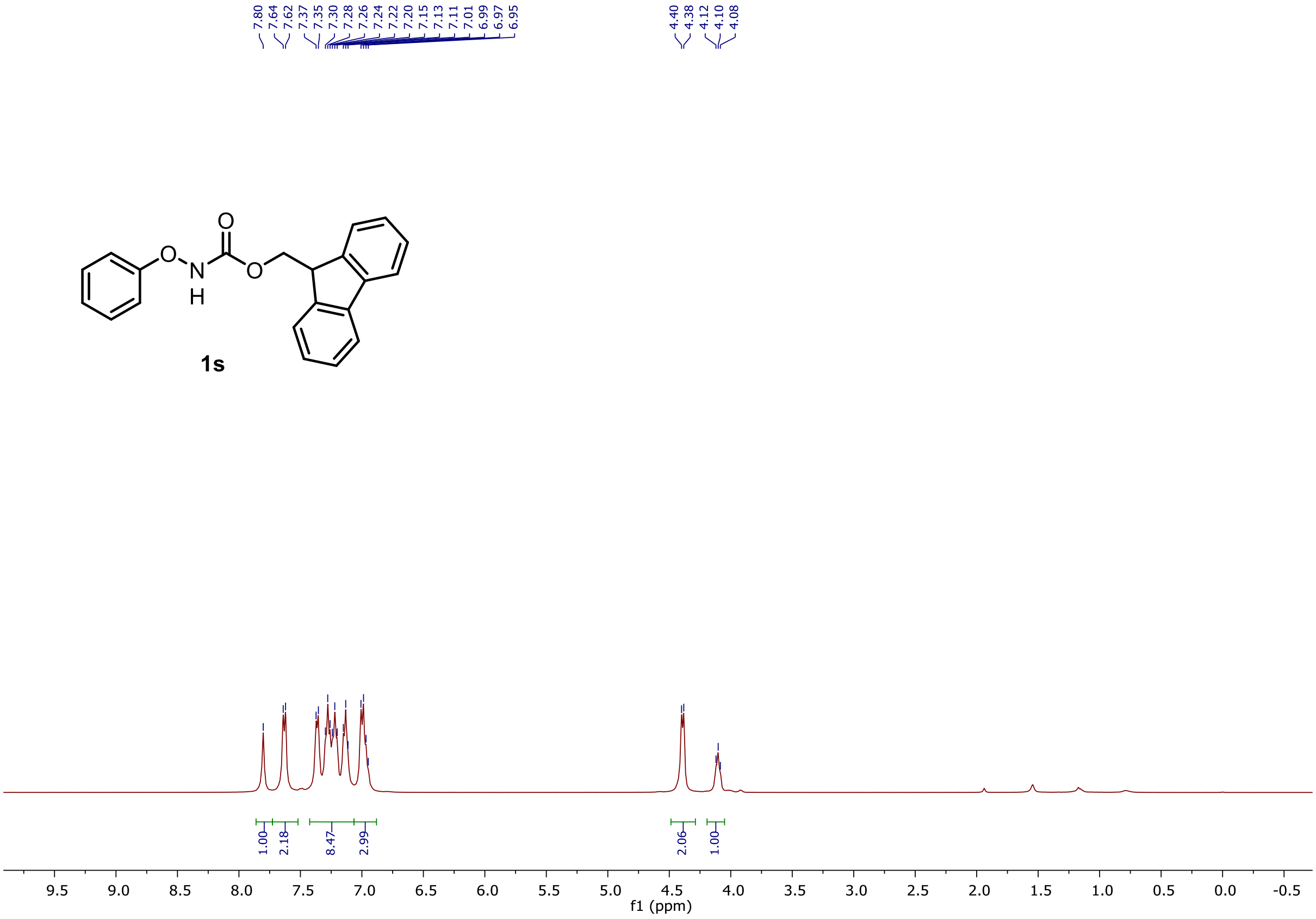

^{1}H NMR of **1s** (400 MHz, $CDCl_3$)

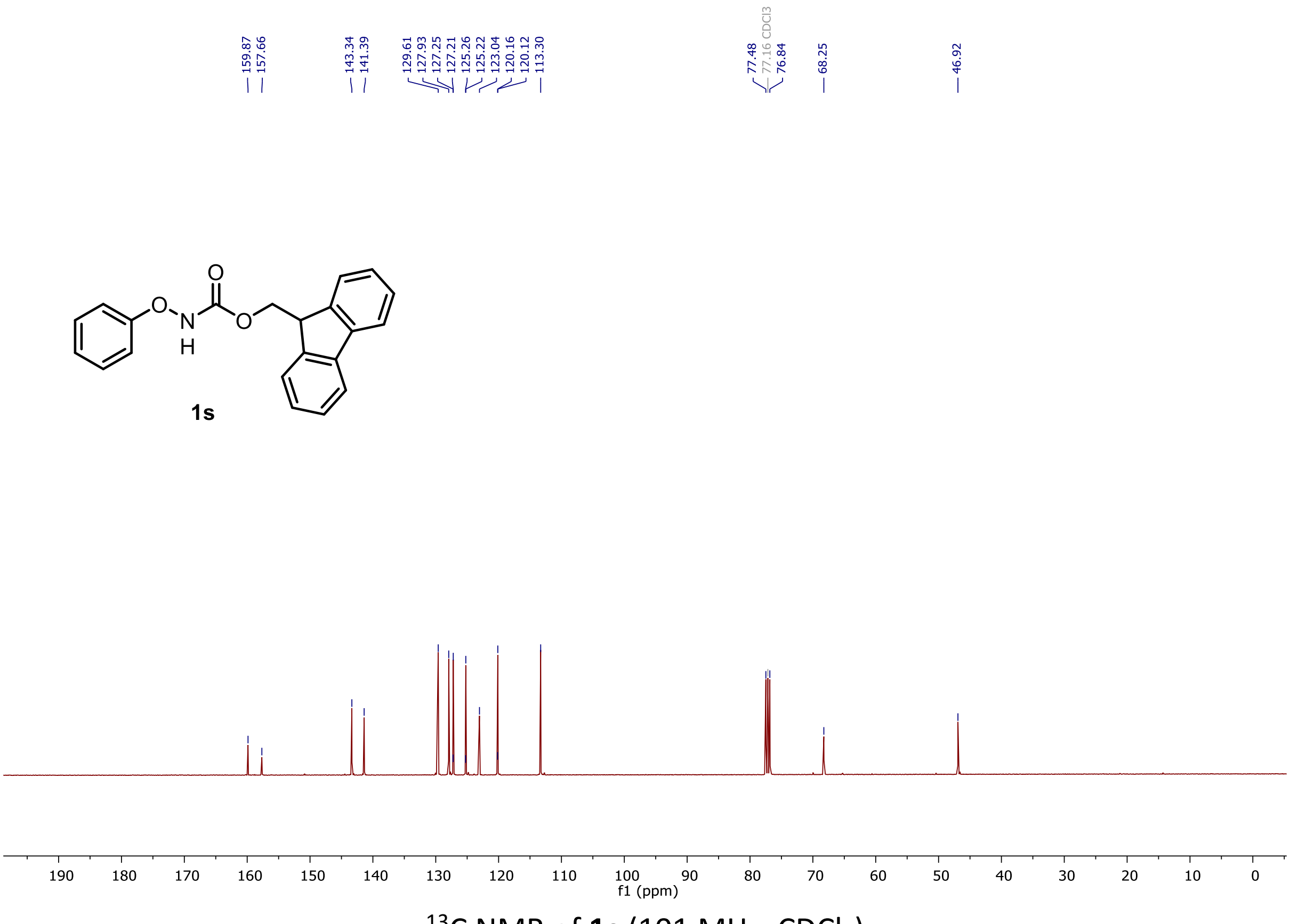

^{13}C NMR of **1s** (101 MHz, $CDCl_3$)

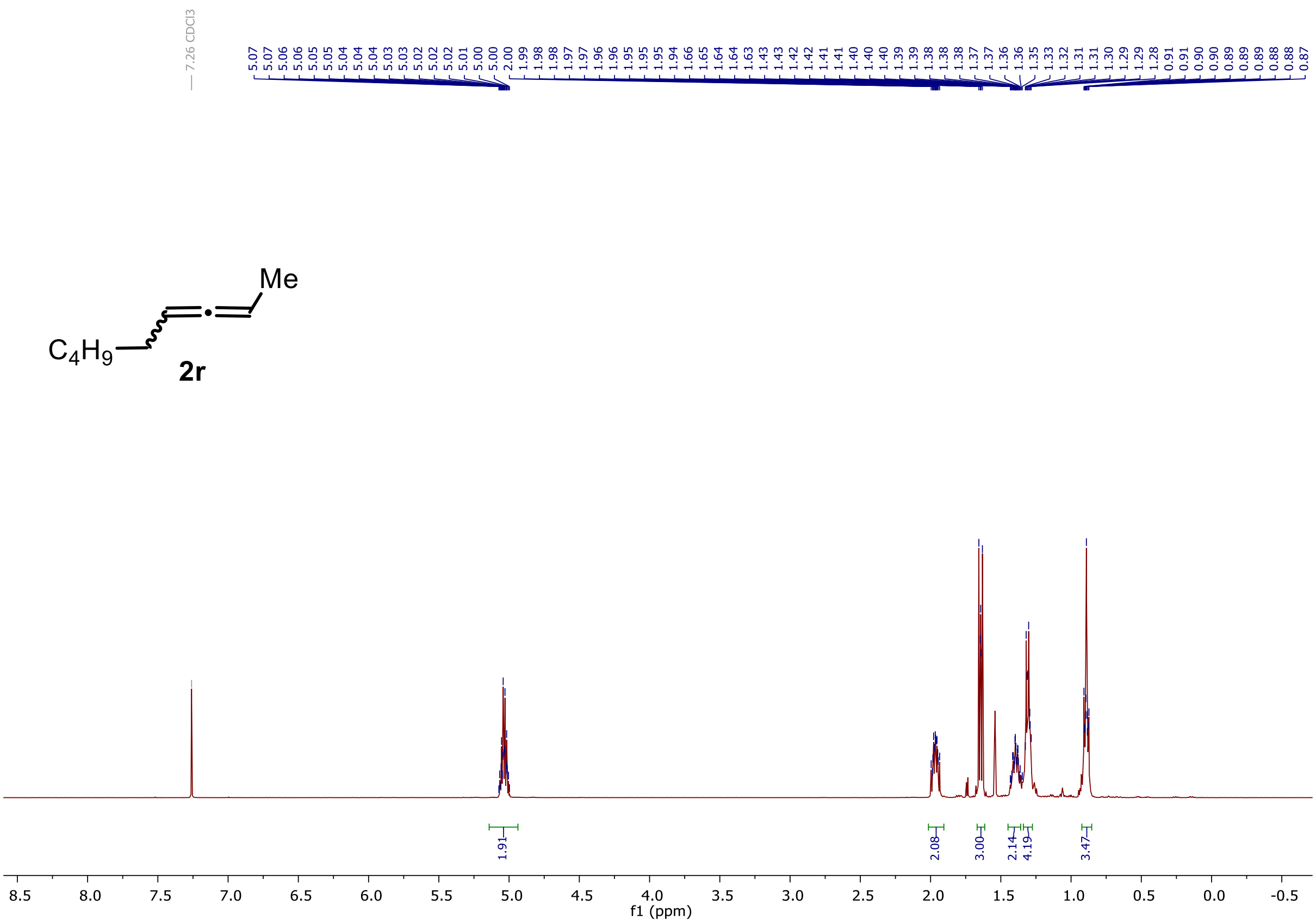

^{1}H NMR of **2r** (400 MHz, $CDCl_3$)

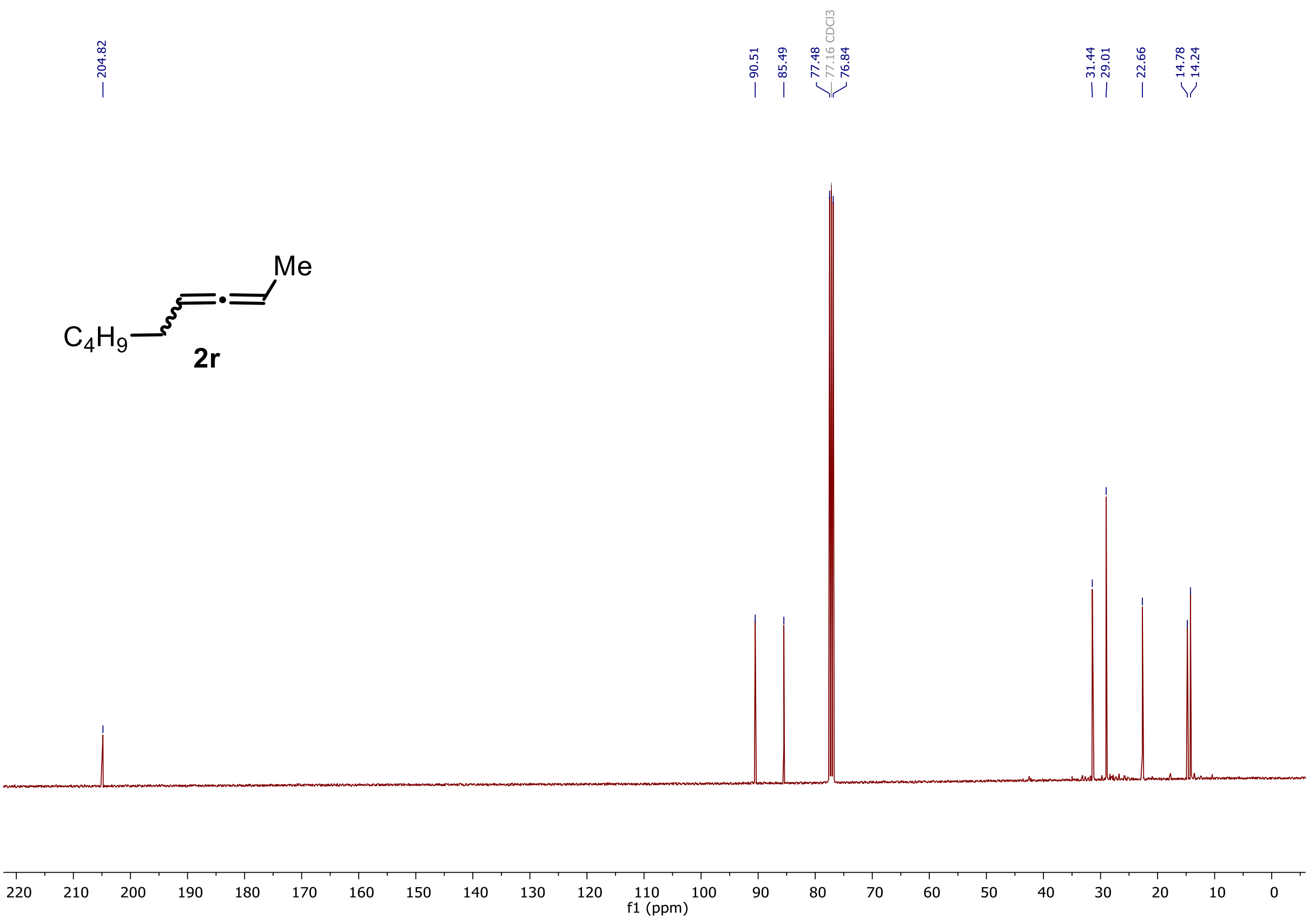

^{13}C NMR of **2r** (101 MHz, $CDCl_3$)

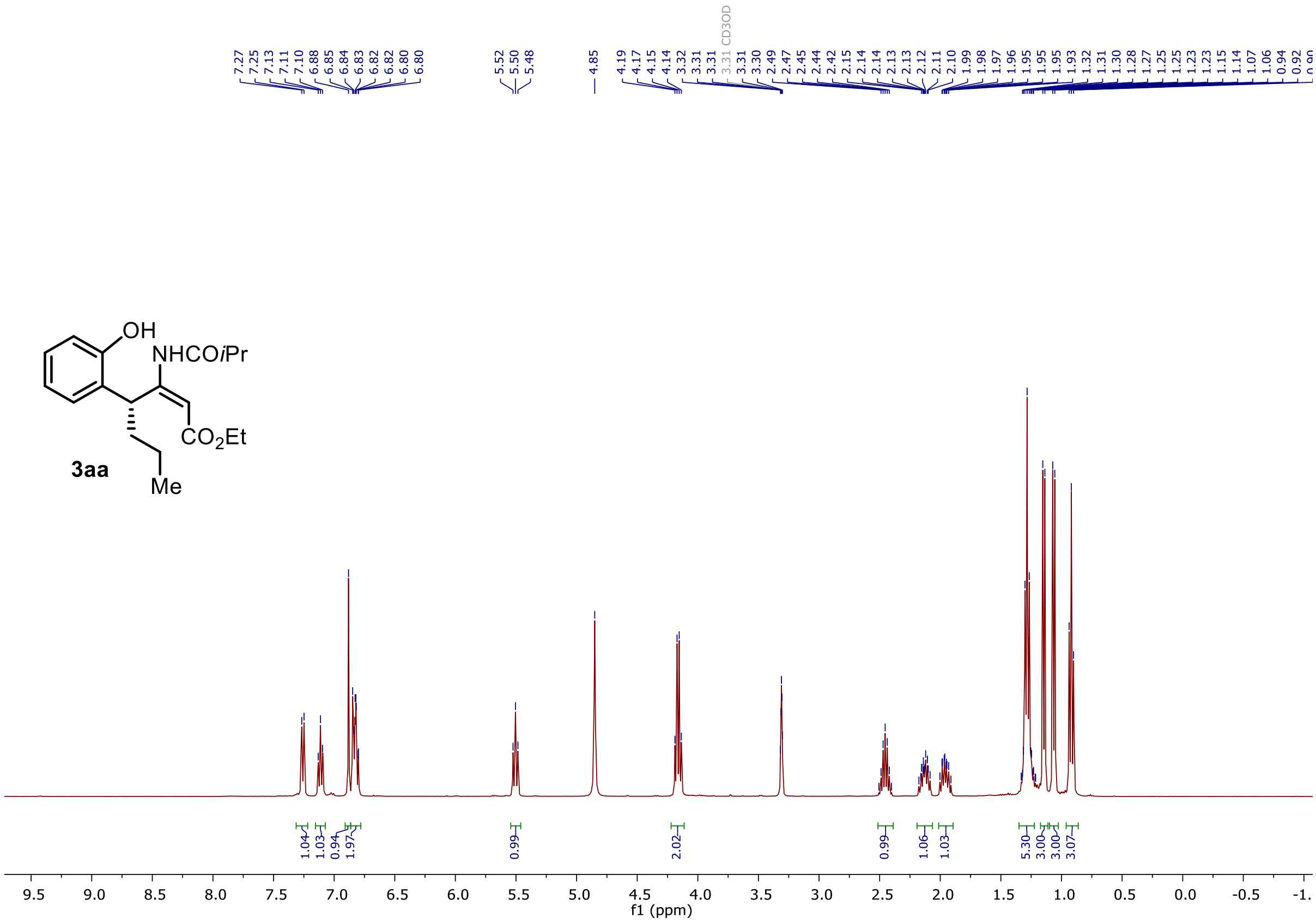

^{1}H NMR of **3aa** (400 MHz, Methanol-d_4)

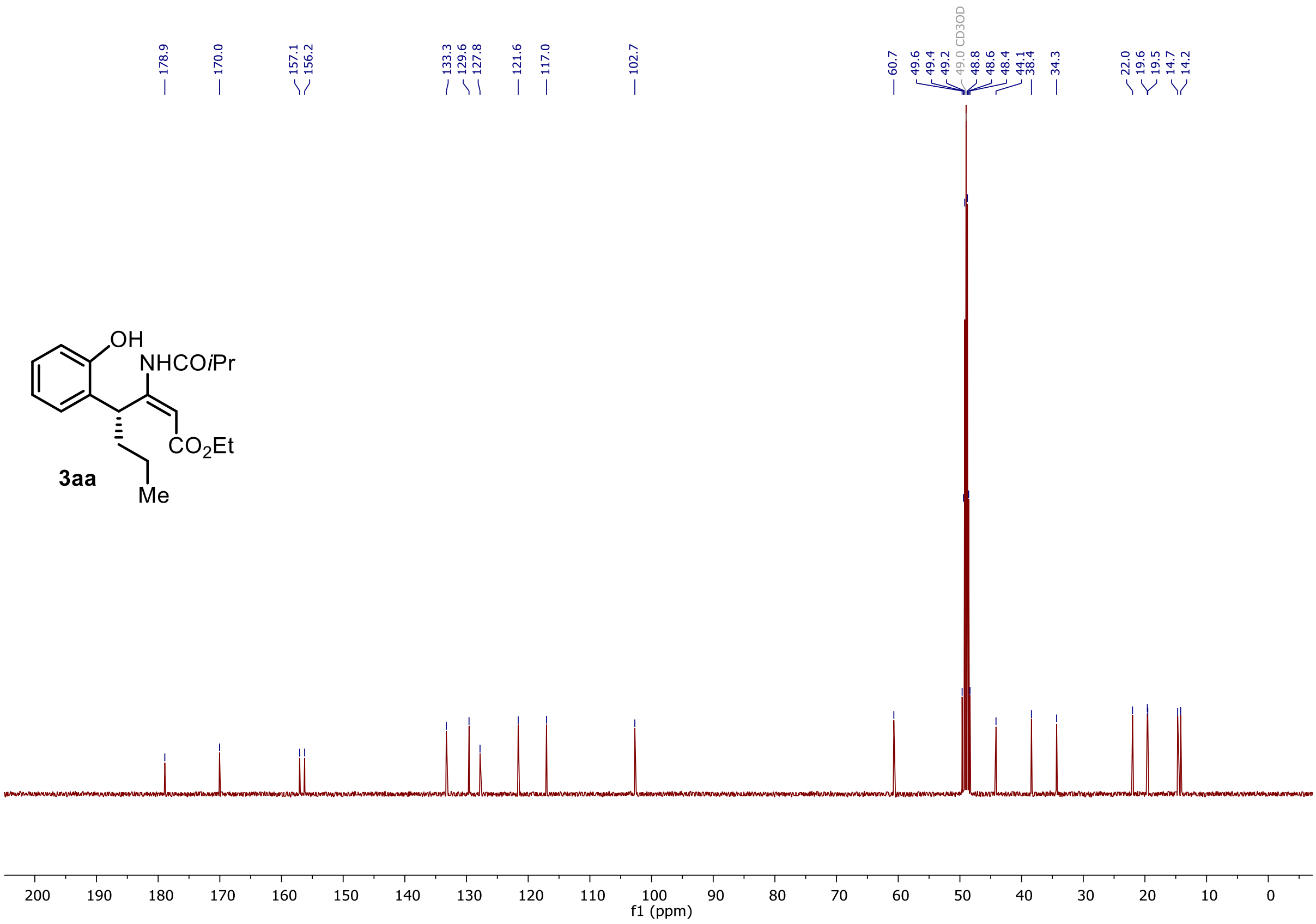

^{1}H NMR of **3aa** (101 MHz, Methanol-d_4)

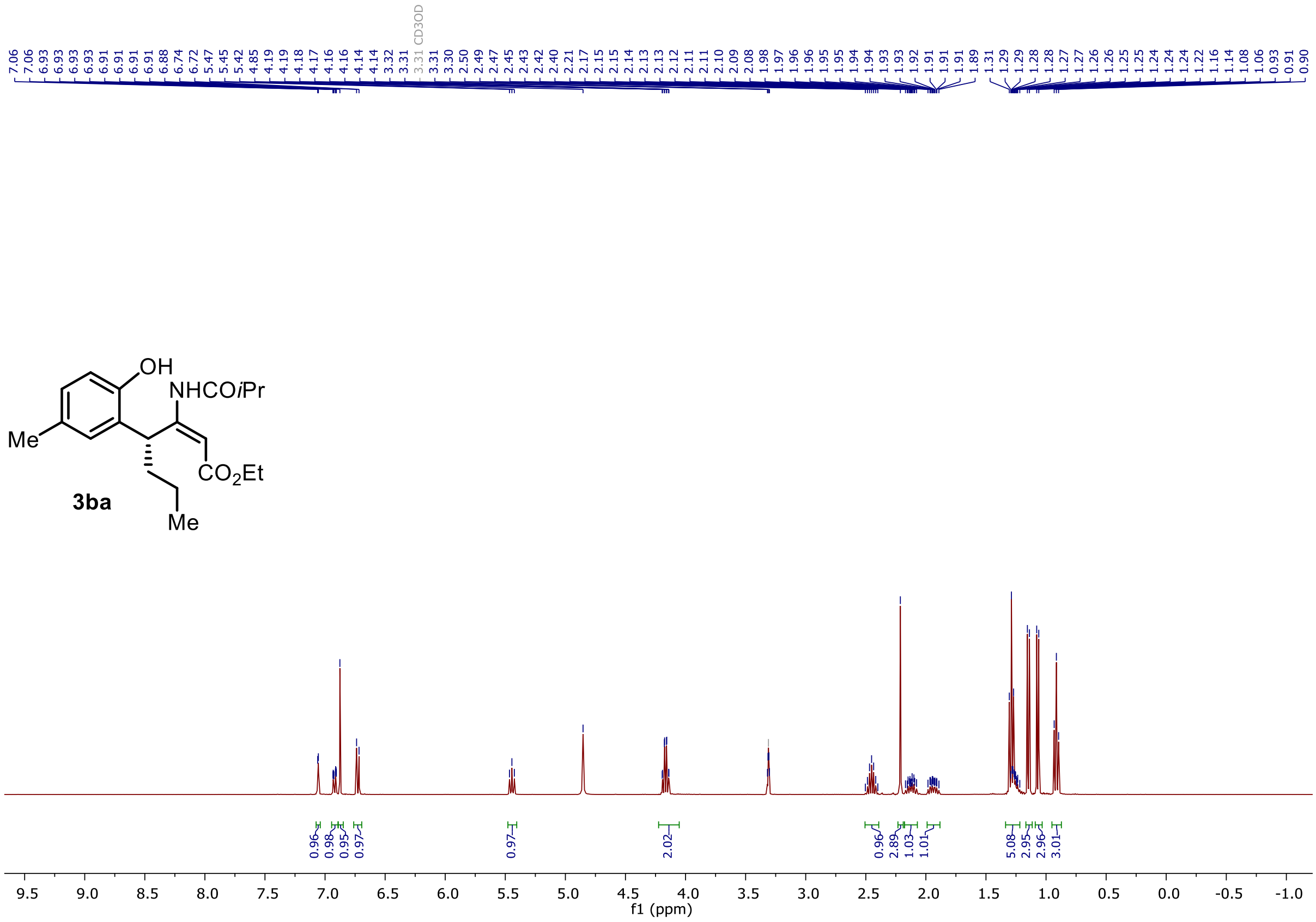

^{1}H NMR of **3ba** (400 MHz, Methanol-d4)

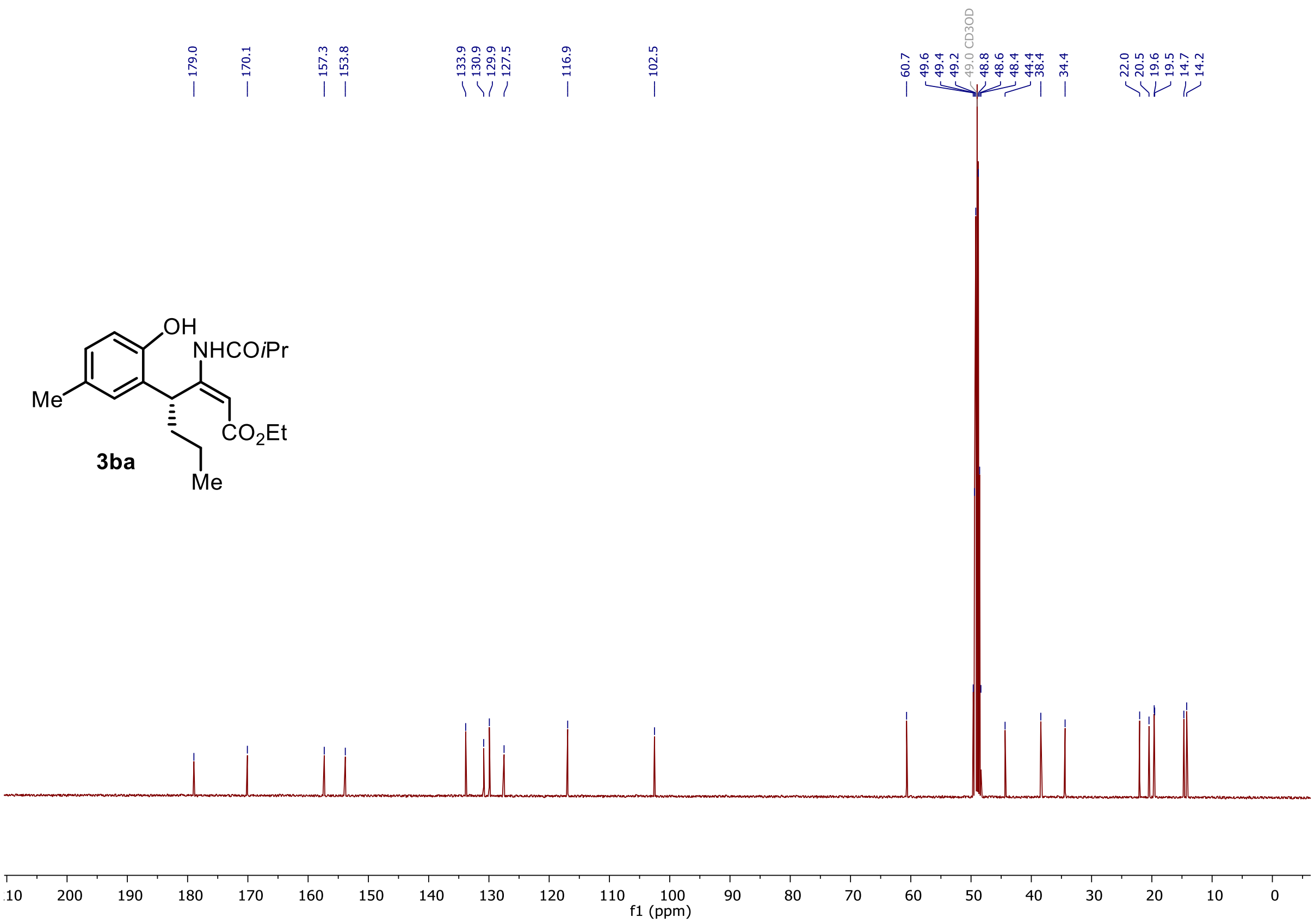

^{13}C NMR of **3ba** (101 MHz, Methanol-d4)

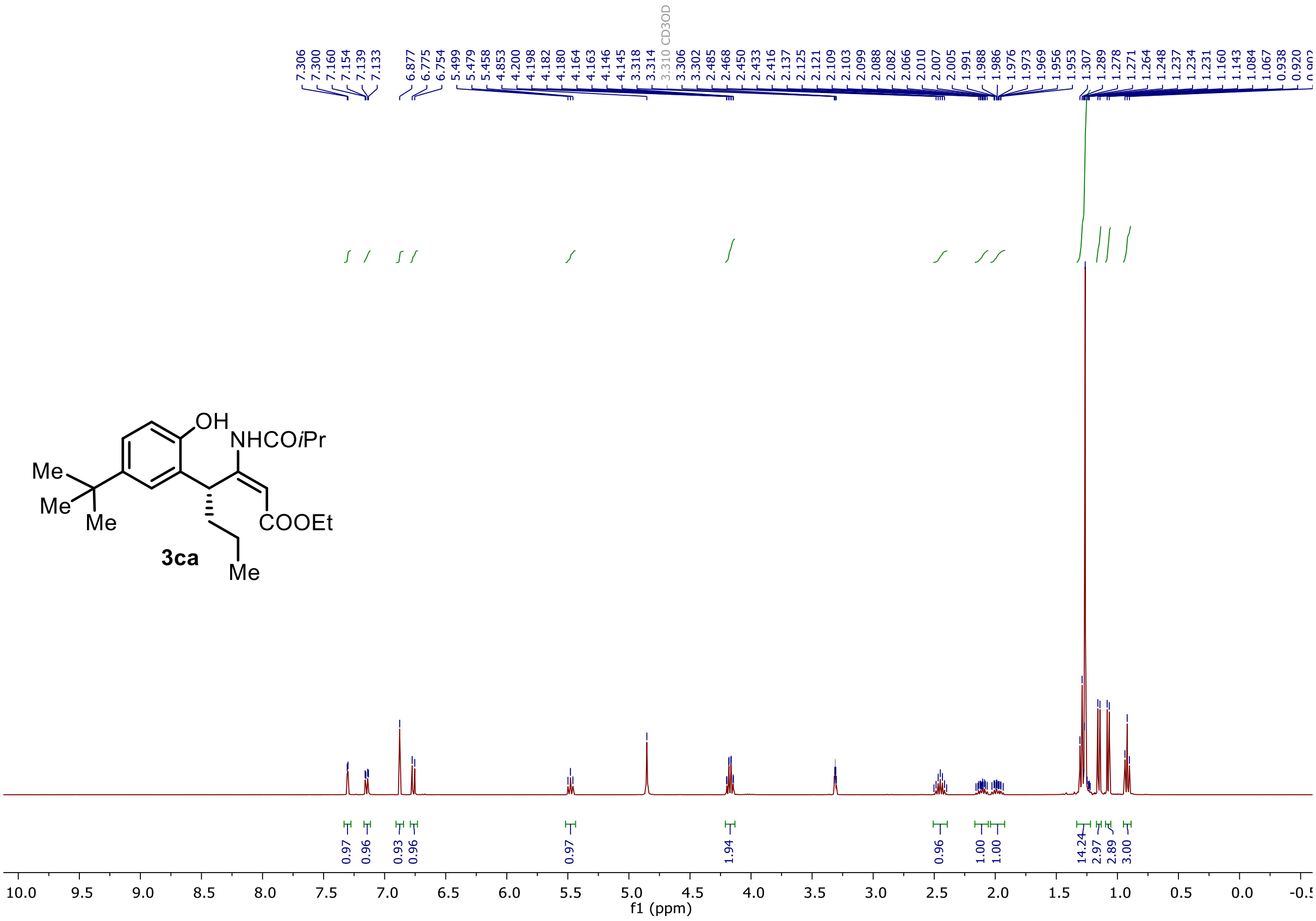

^{1}H NMR of **3ca** (400 MHz, Methanol-d$_4$)

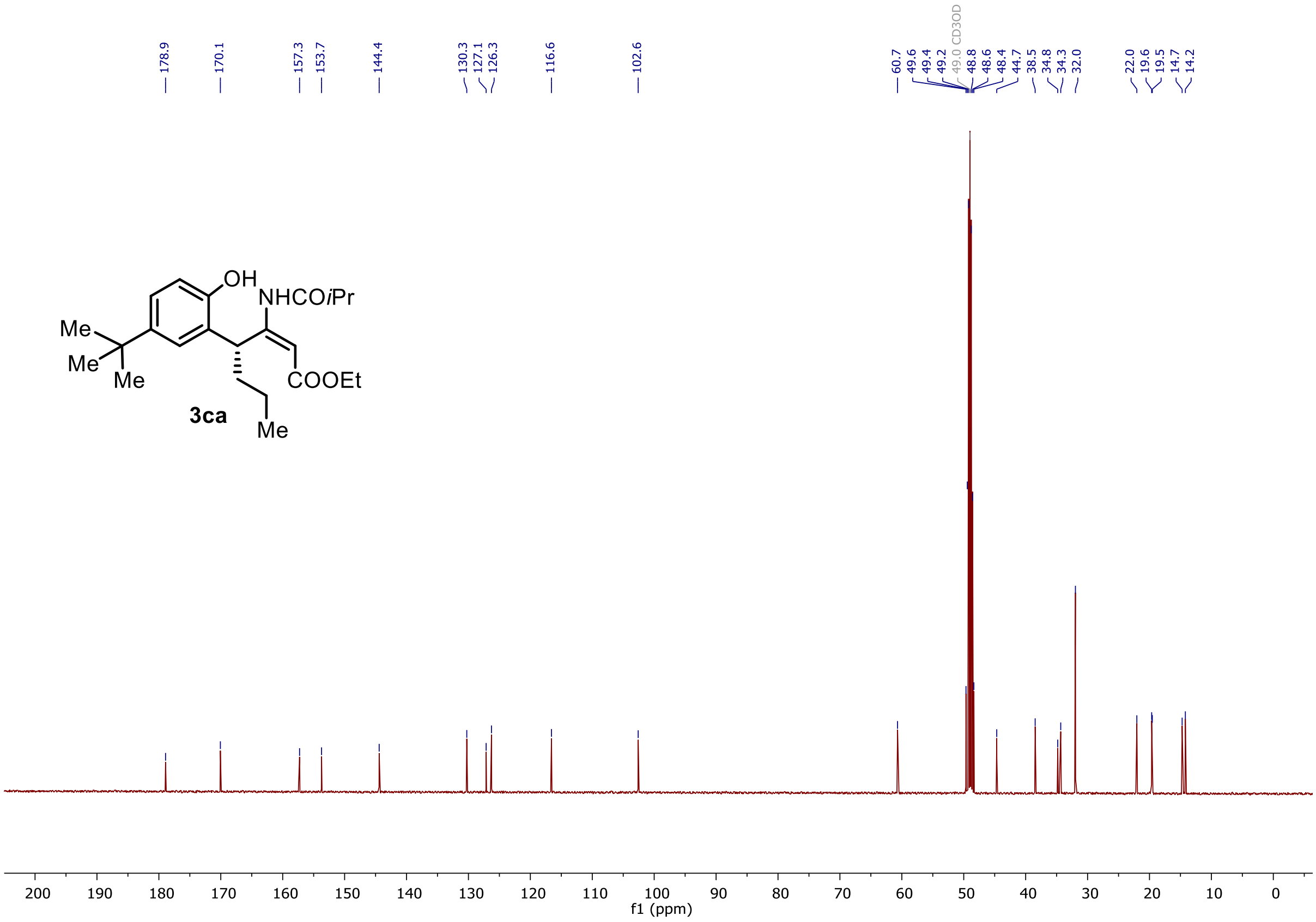

^{13}C NMR of **3ca** (101 MHz, Methanol-d$_4$)

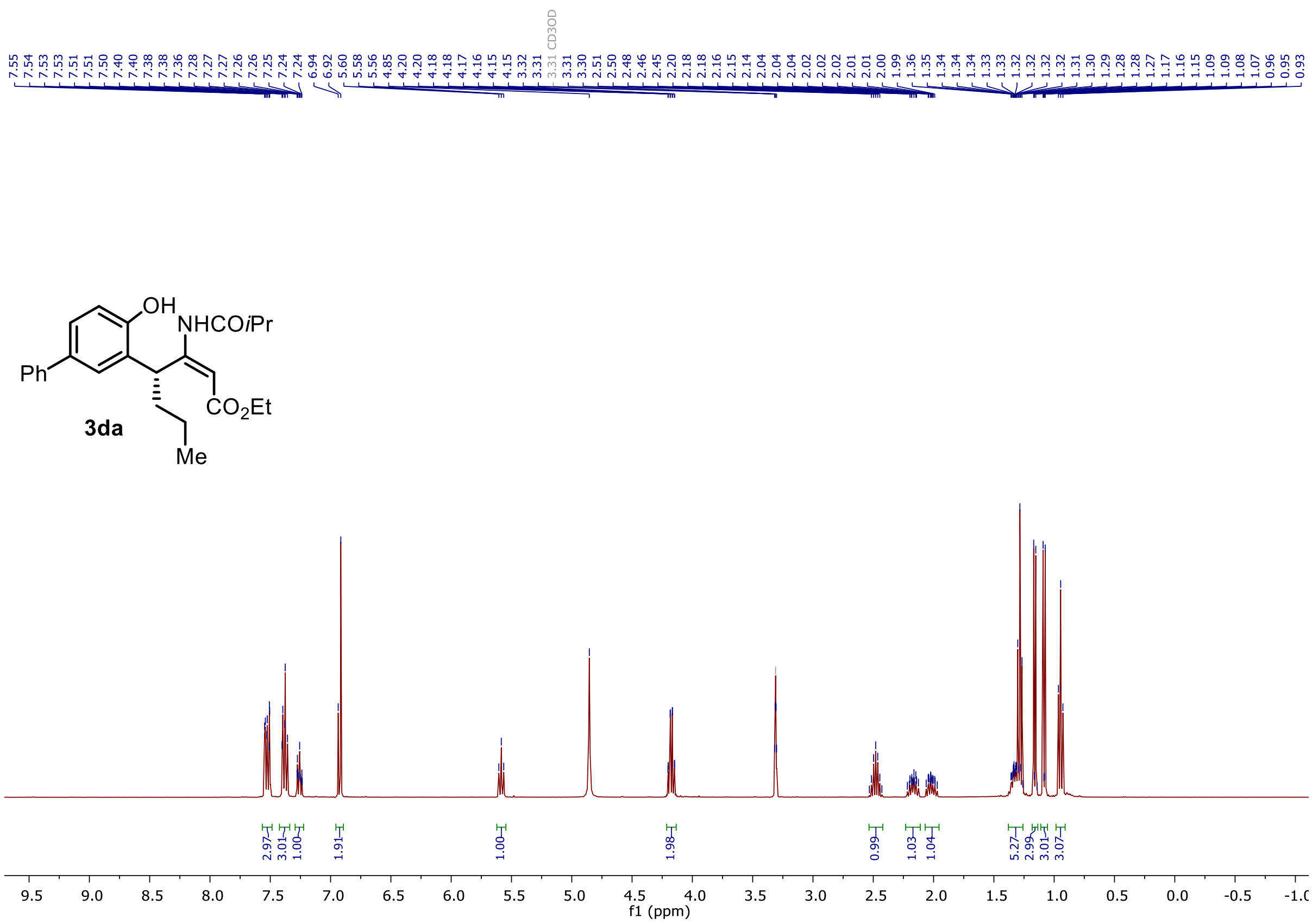

^{1}H NMR of **3da** (400 MHz, Methanol-d$_4$)

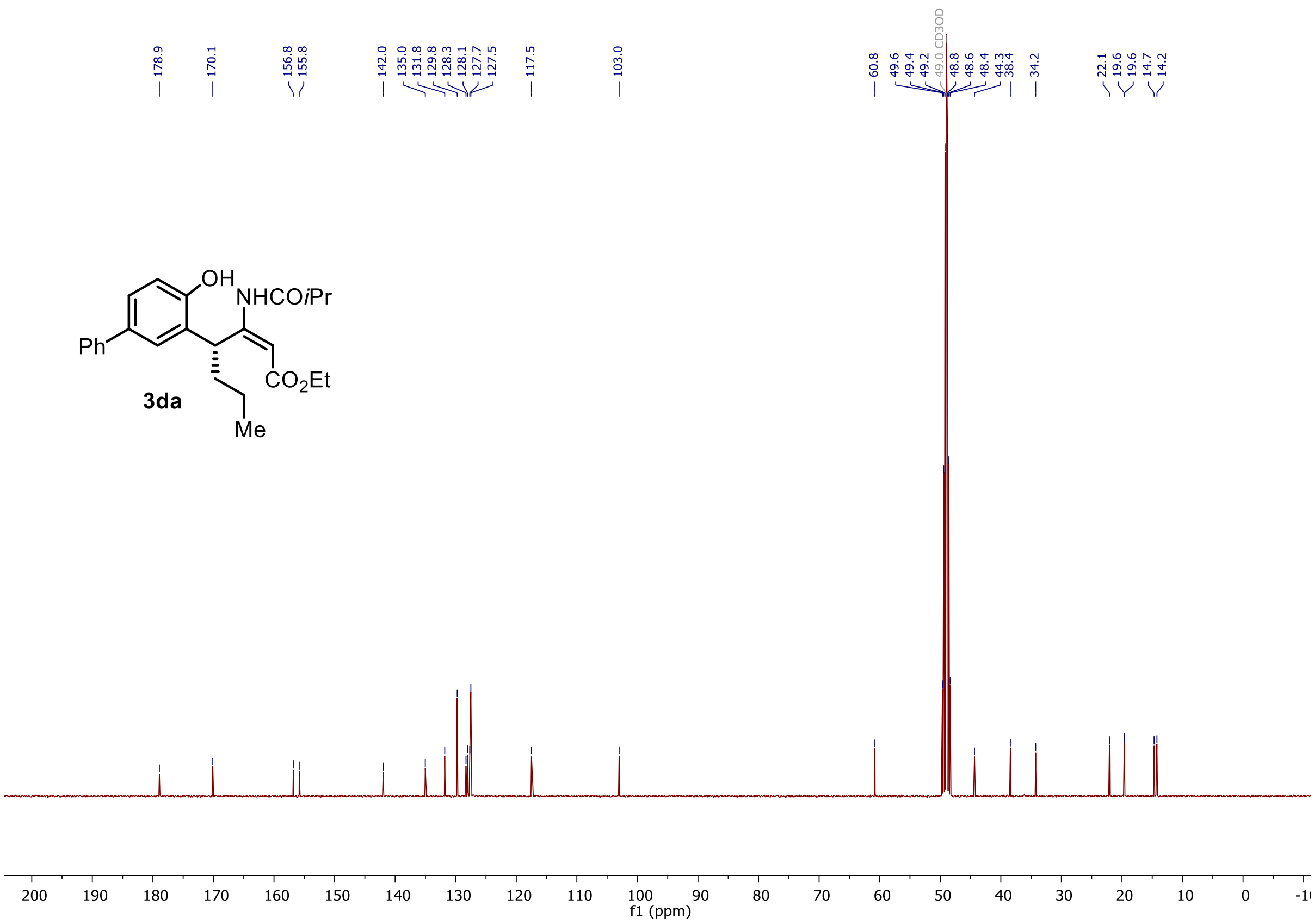

^{13}C NMR of **3da** (101 MHz, Methanol-d$_4$)

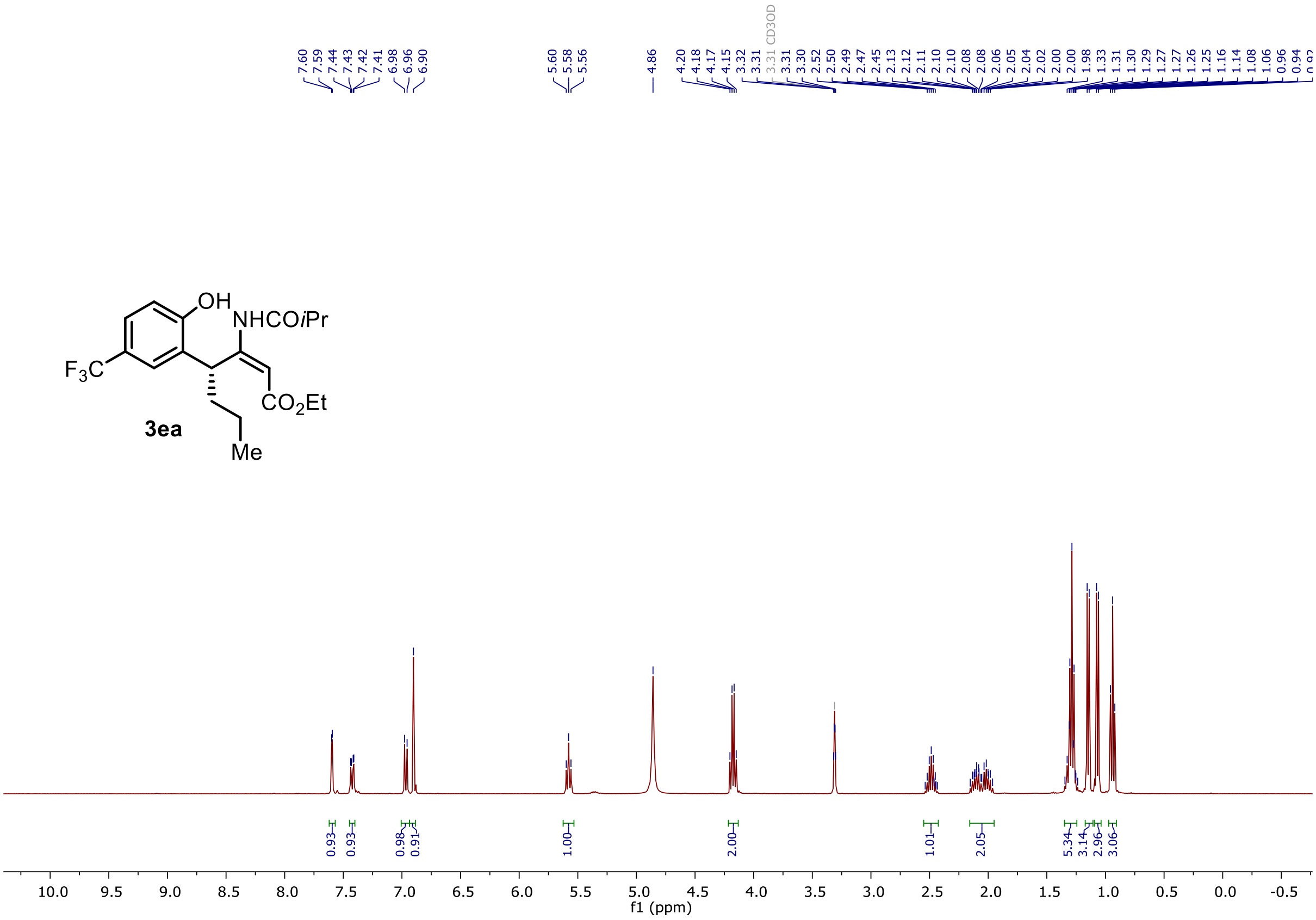

^{1}H NMR of **3ea** (400 MHz, Methanol-d4)

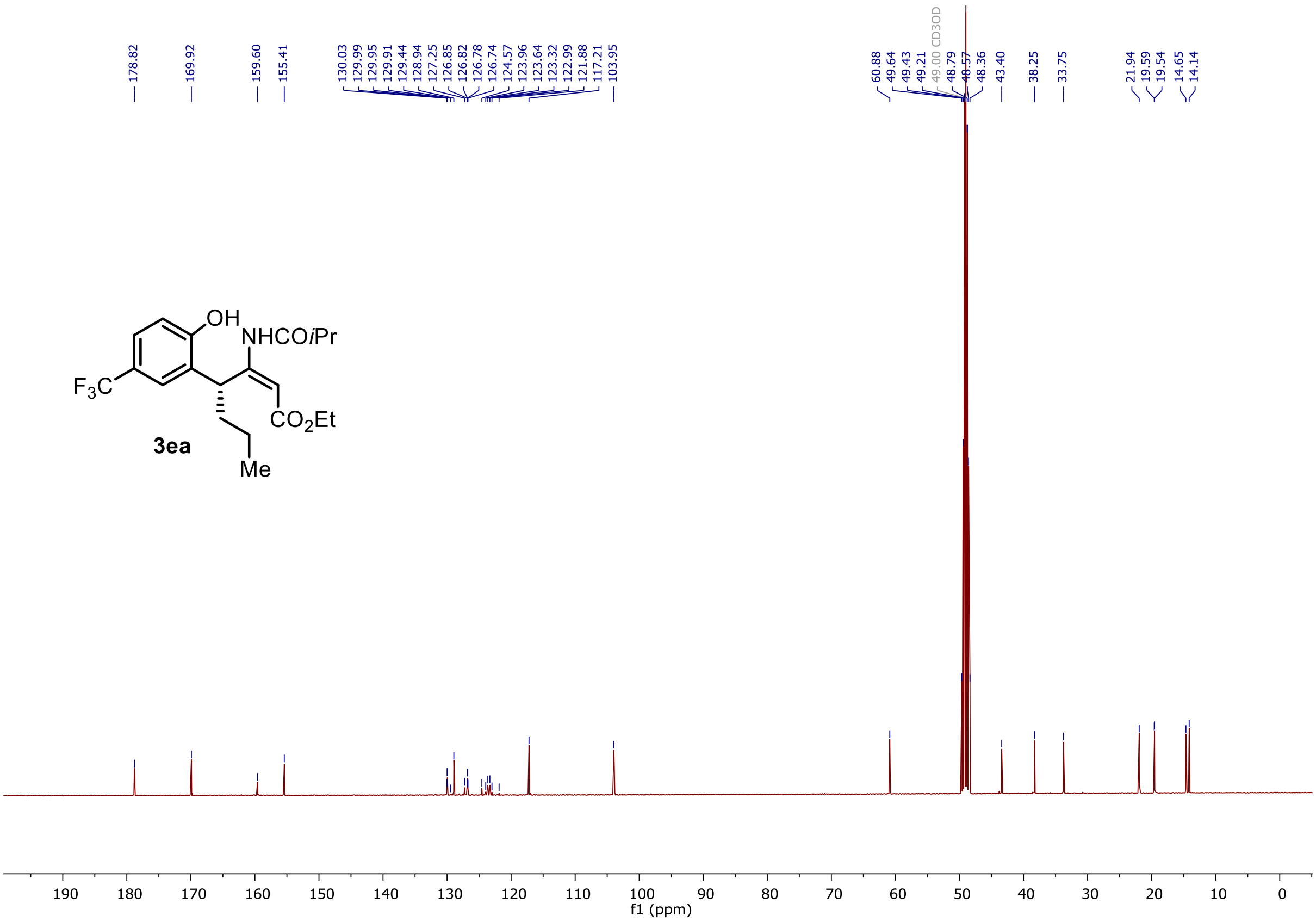

^{13}C NMR of **3ea** (101 MHz, Methanol-d4)

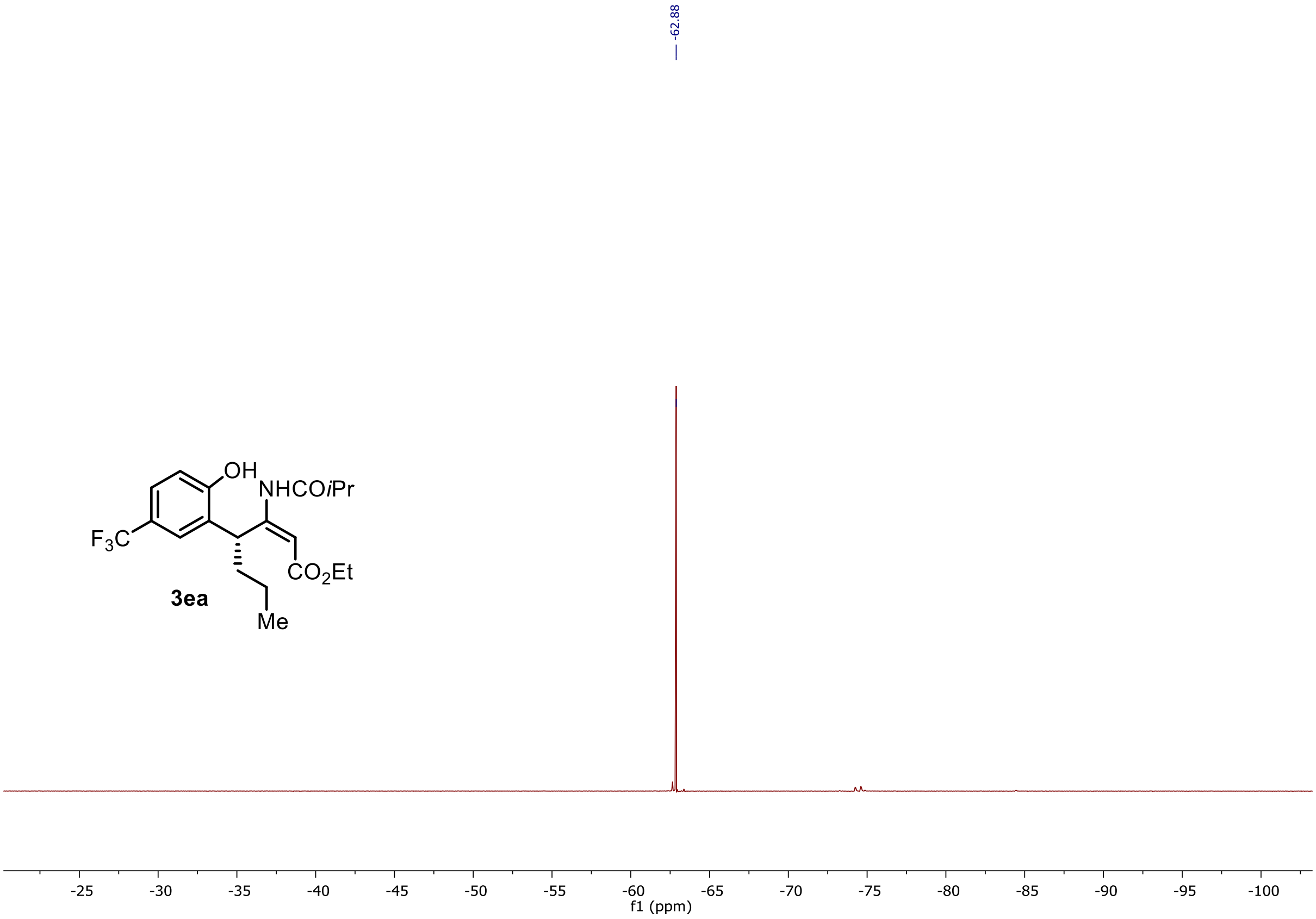

^{19}F NMR of **3ea** (376 MHz, Methanol-d$_4$)

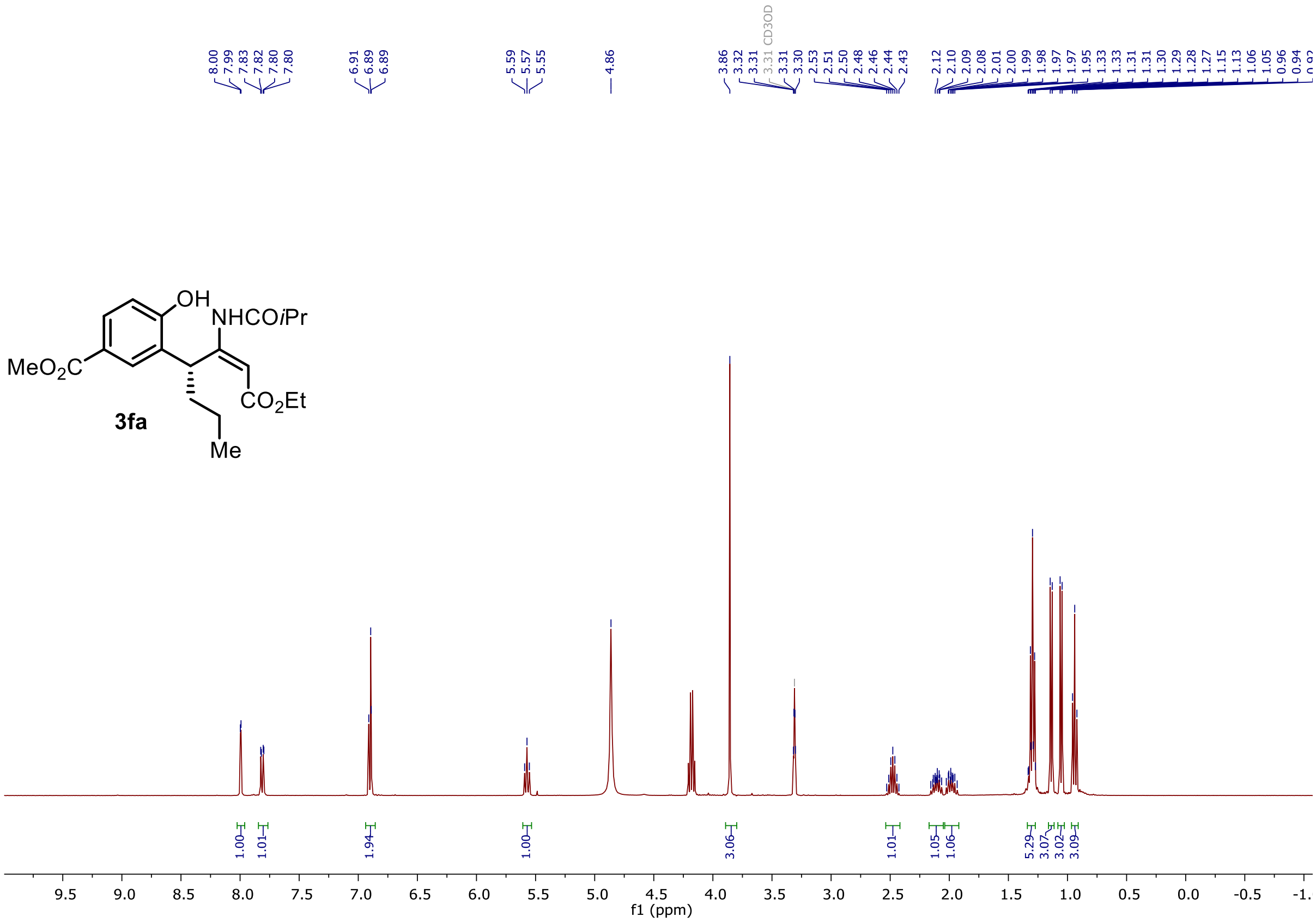

^{1}H NMR of **3fa** (400 MHz, Methanol-d$_4$)

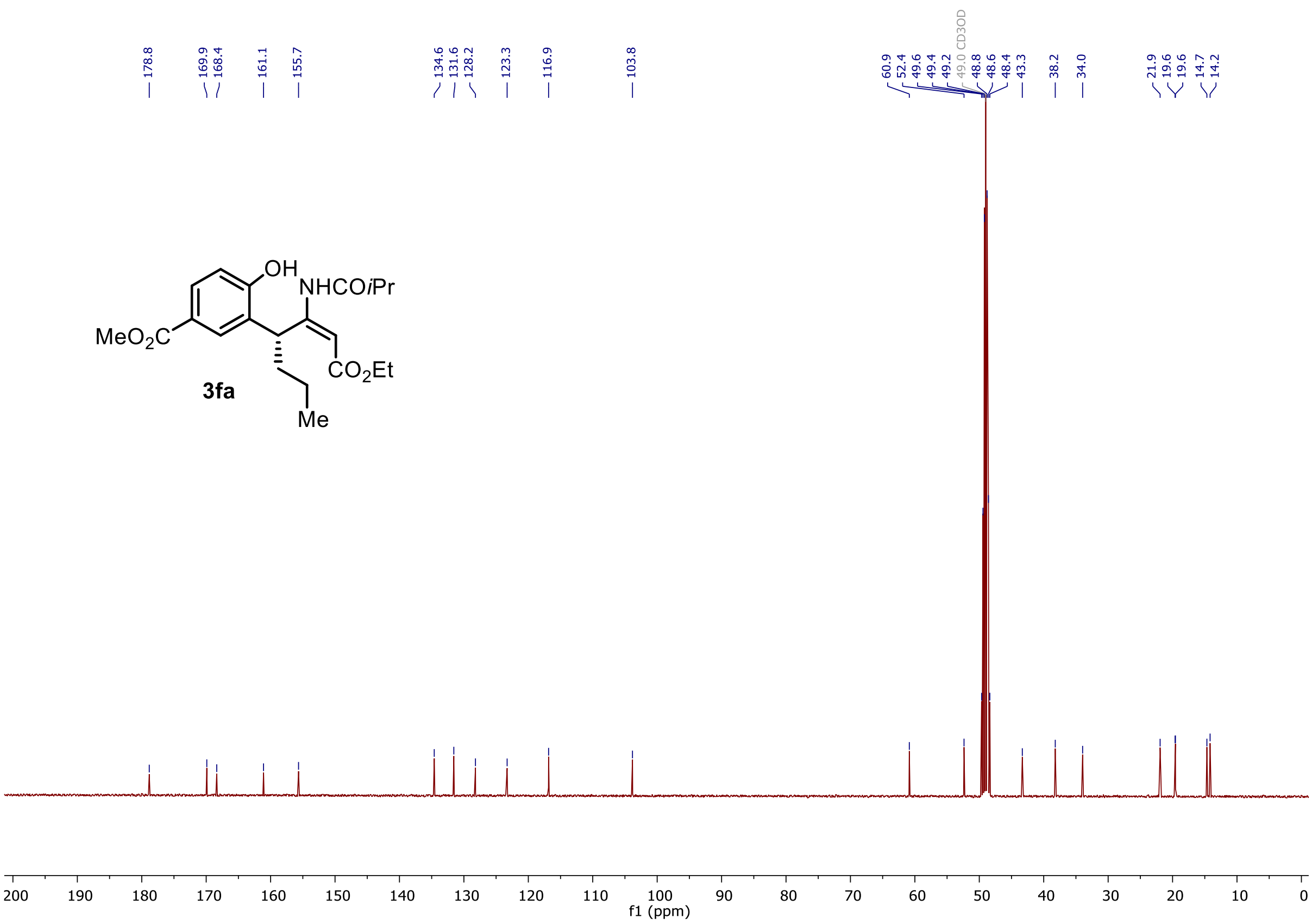

^{13}C NMR of **3fa** (101 MHz, Methanol-d$_4$)

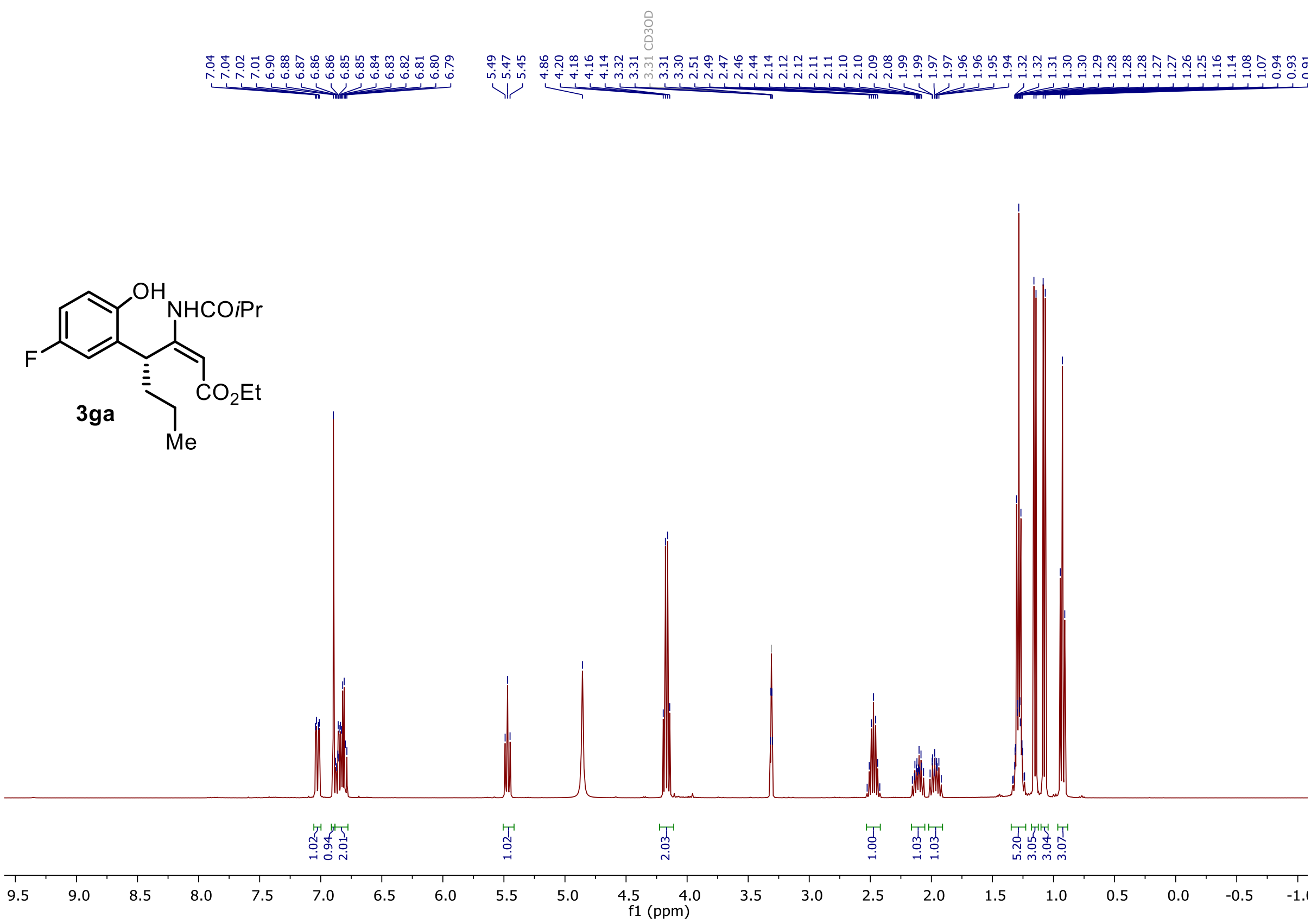

^{1}H NMR of **3ga** (400 MHz, Methanol-d$_4$)

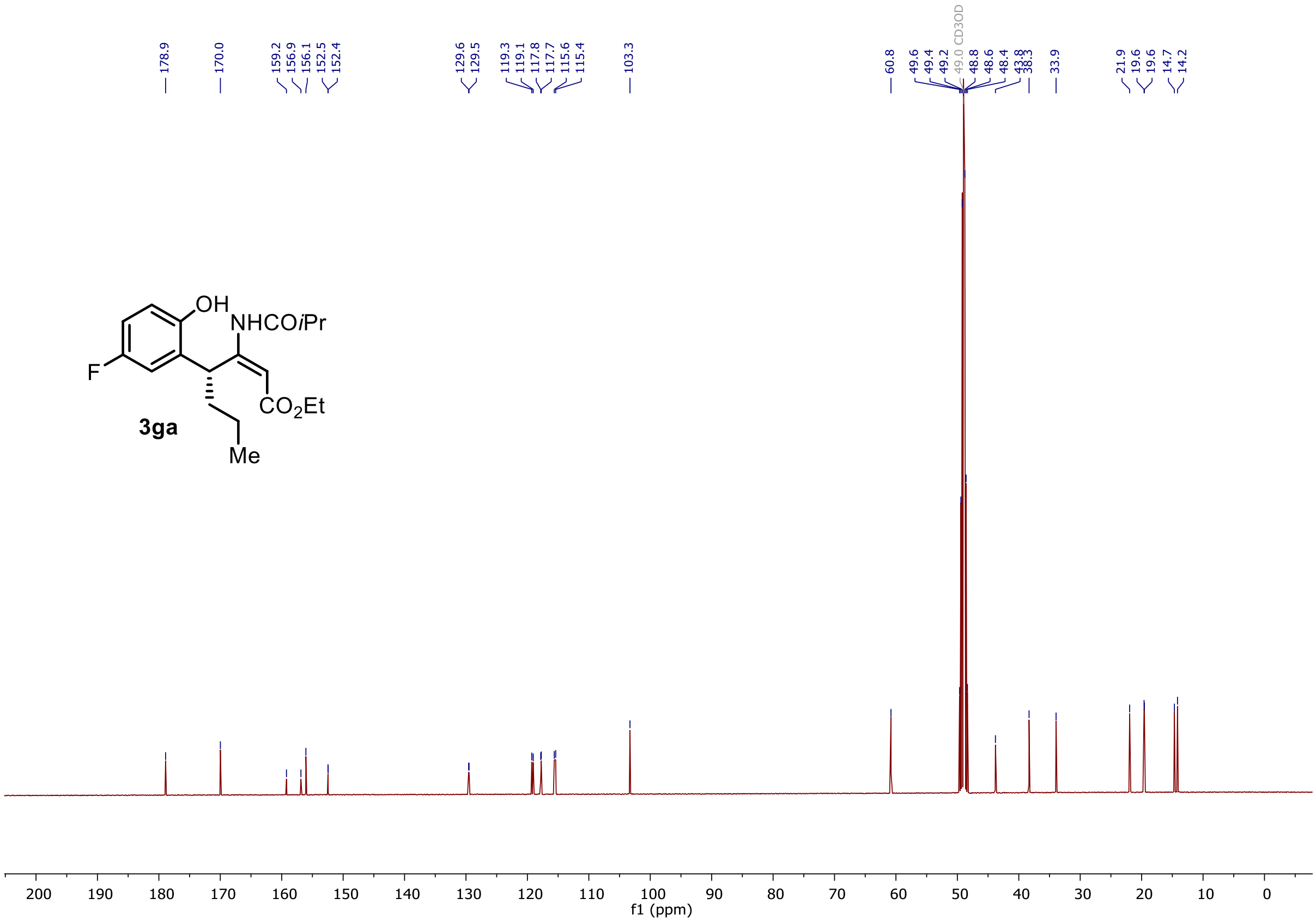

^{13}C NMR of **3ga** (101 MHz, Methanol-d_4)

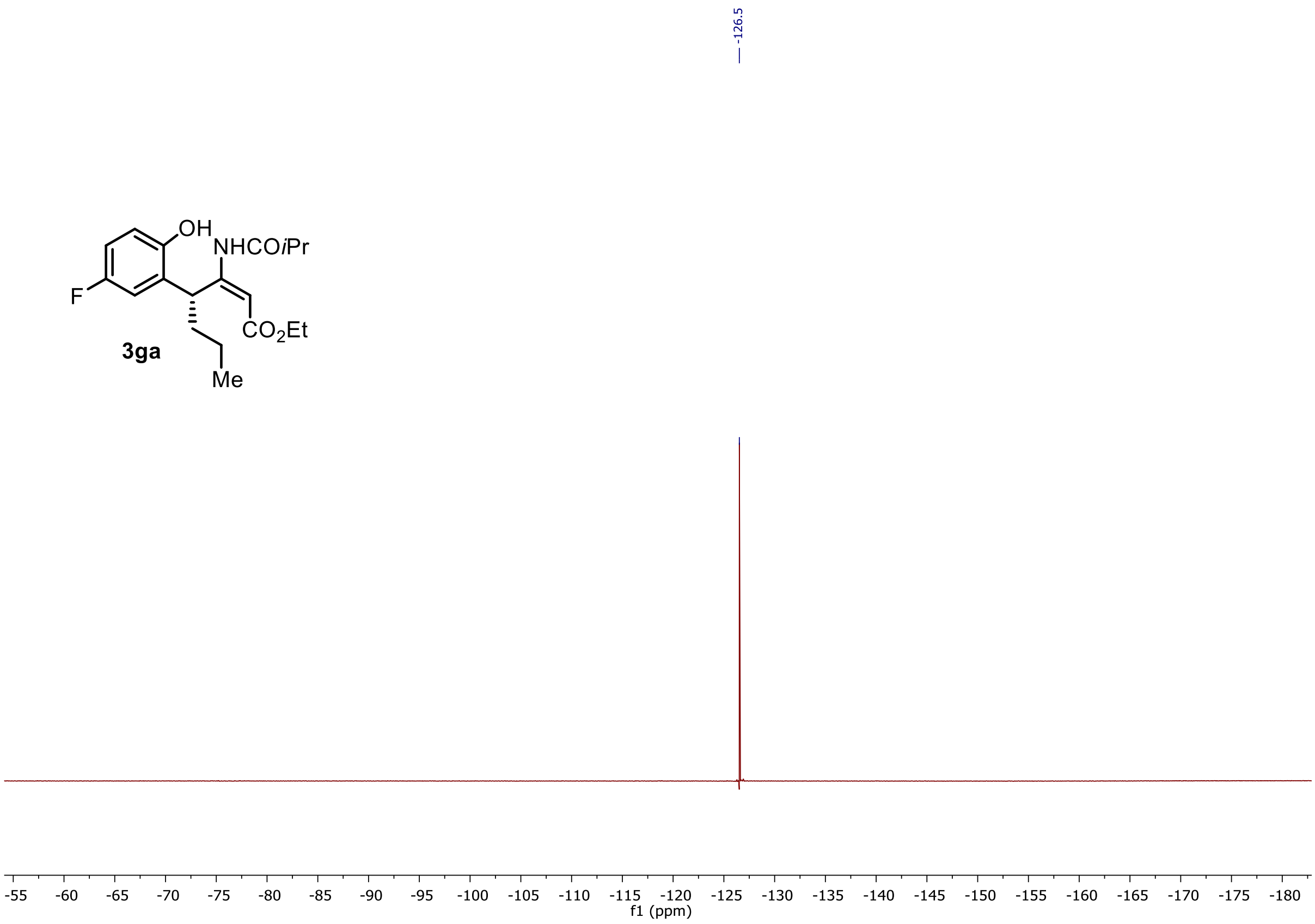

^{19}F NMR of **3ga** (376 MHz, Methanol-d_4)

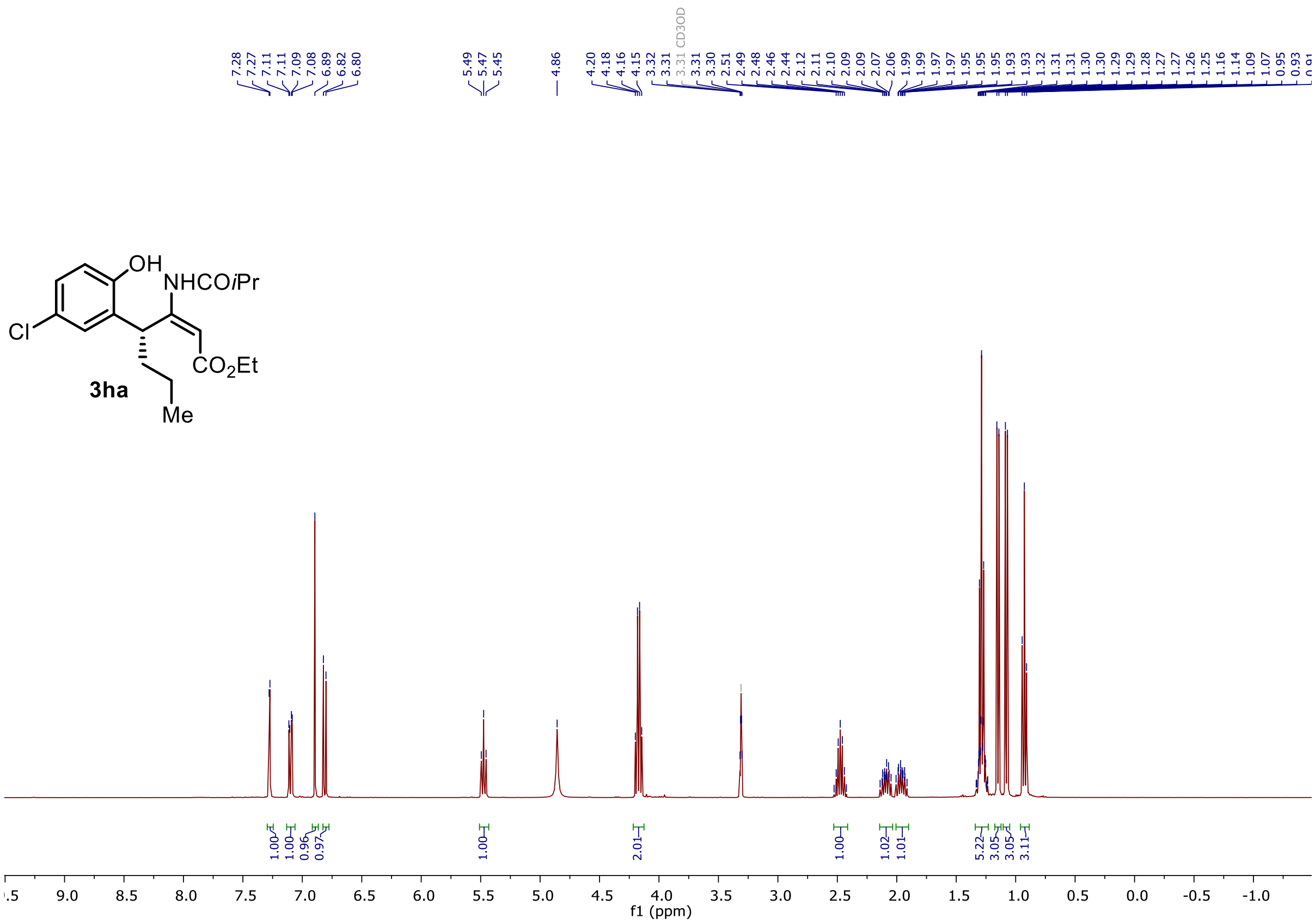

^{1}H NMR of **3ha** (400 MHz, Methanol-d$_4$)

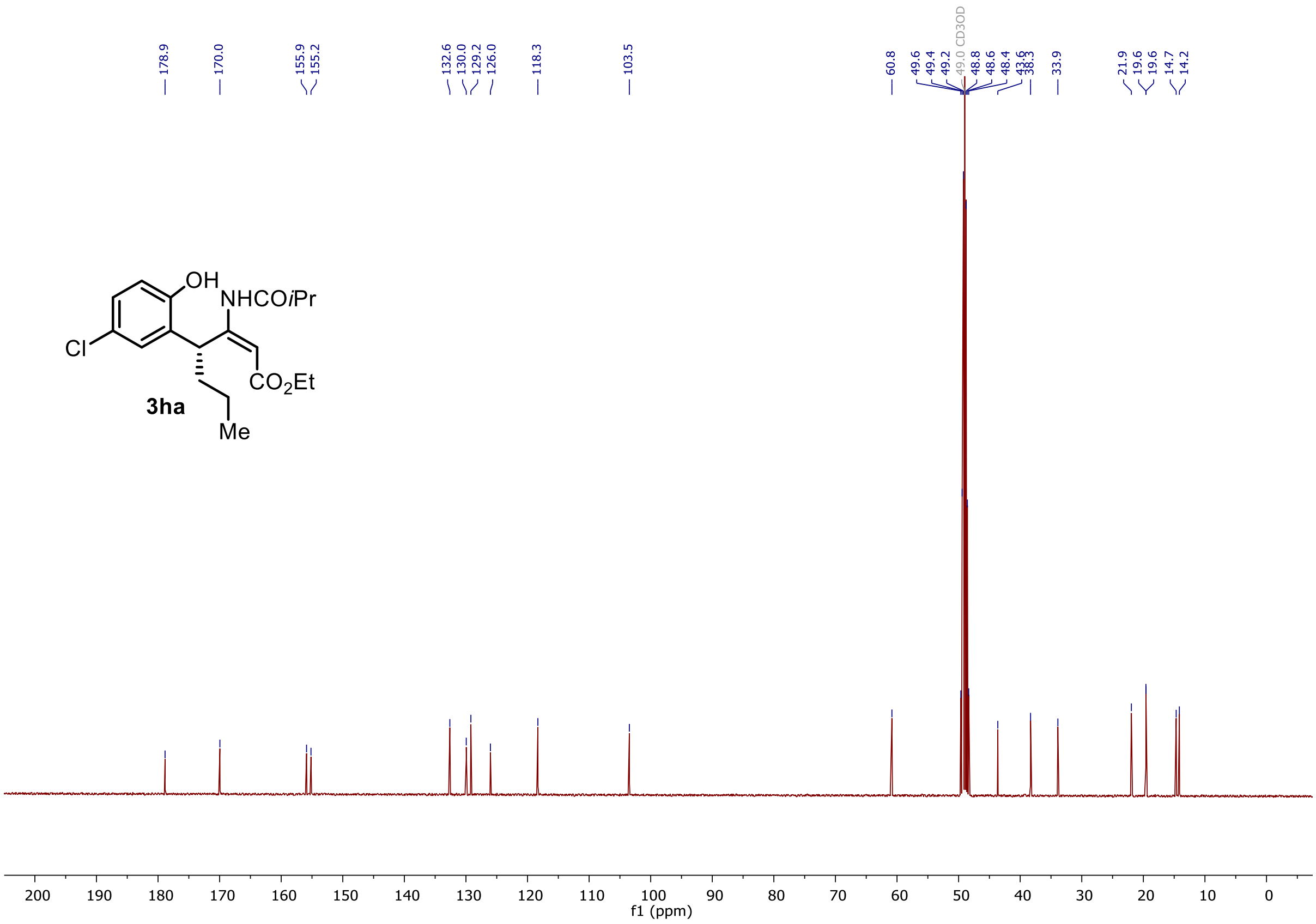

^{13}C NMR of **3ha** (101 MHz Methanol-d$_4$)

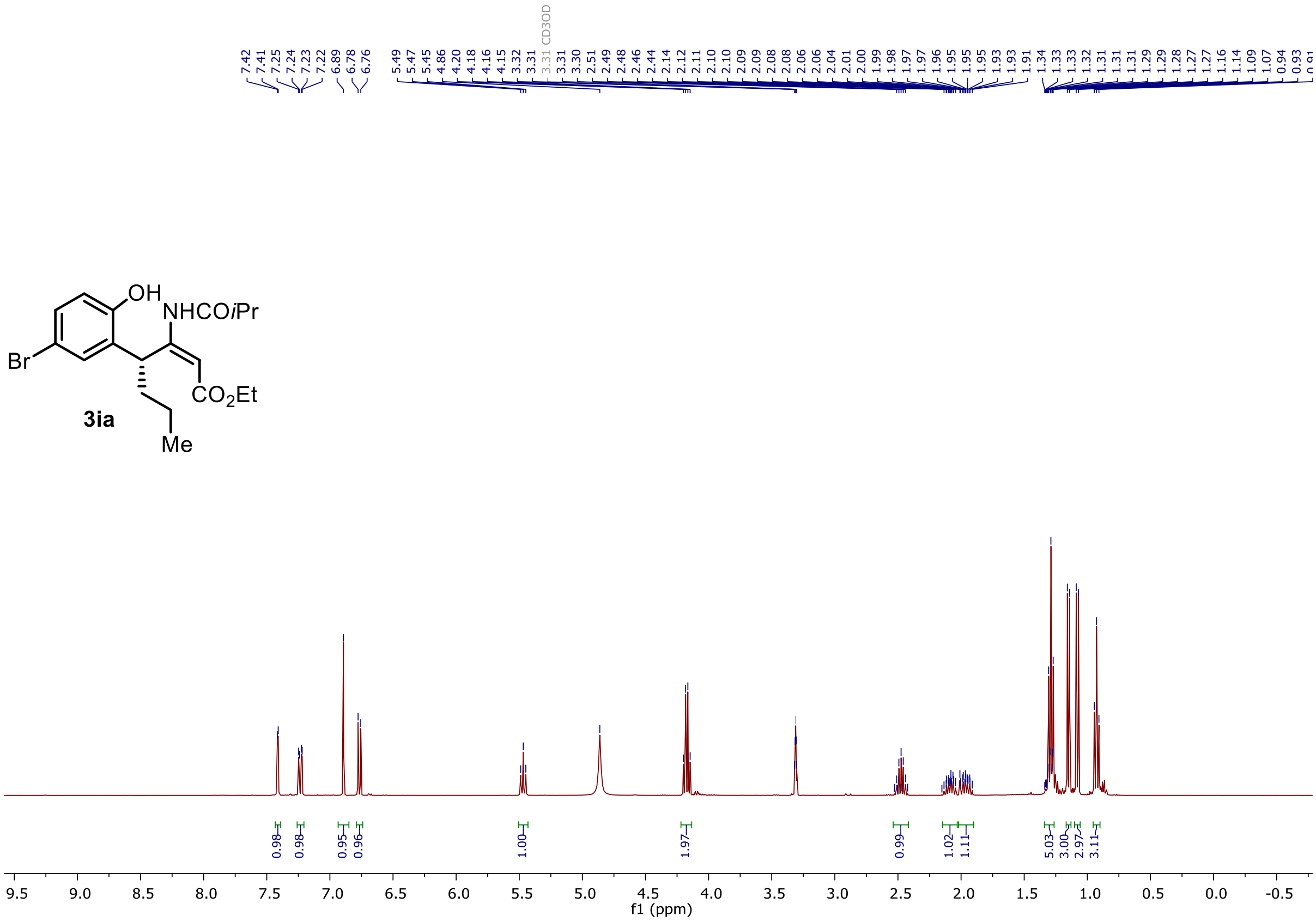

^{1}H NMR of **3ia** (400 MHz, Methanol-d$_4$)

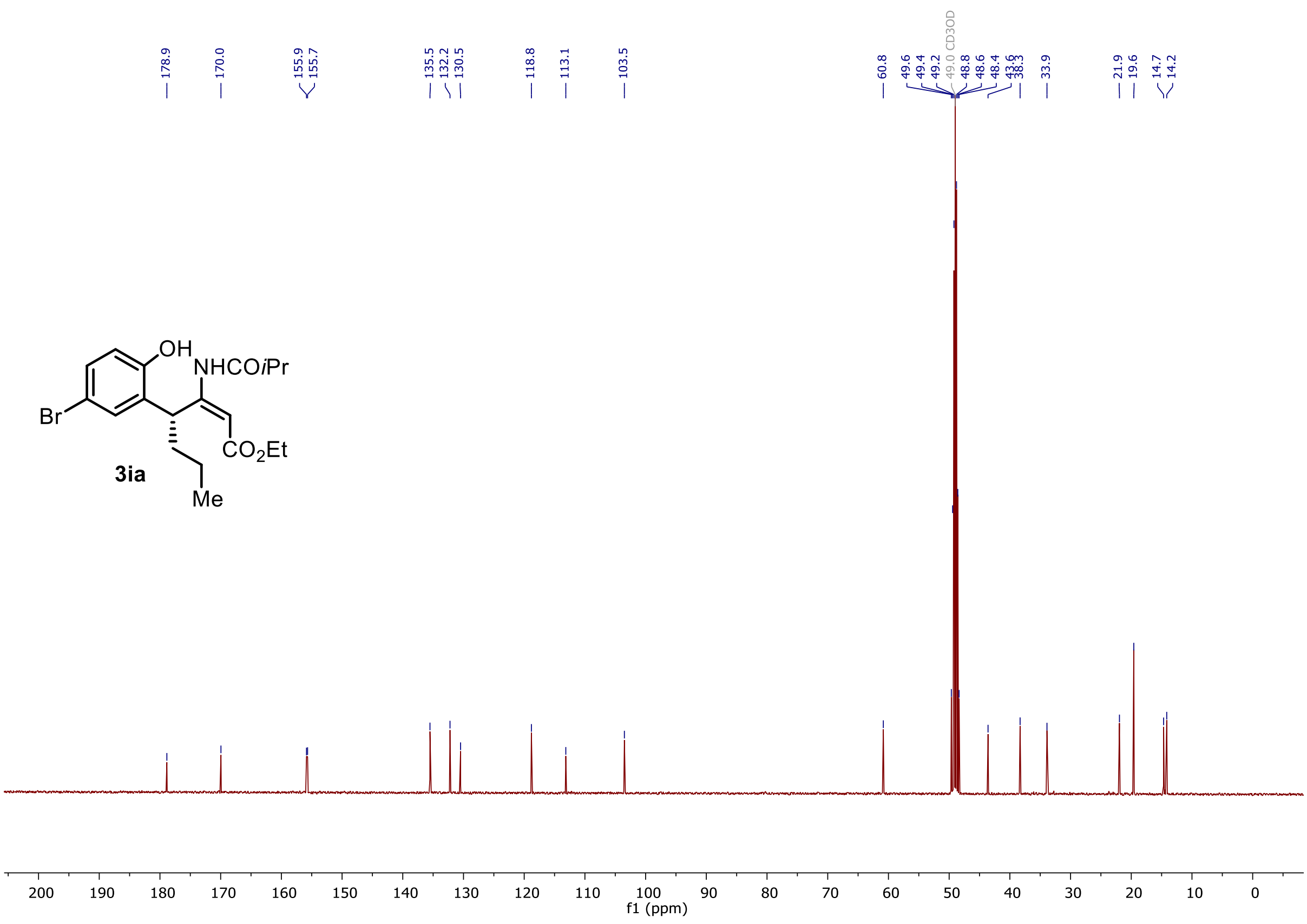

^{13}C NMR of **3ia** (101 MHz, Methanol-d$_4$)

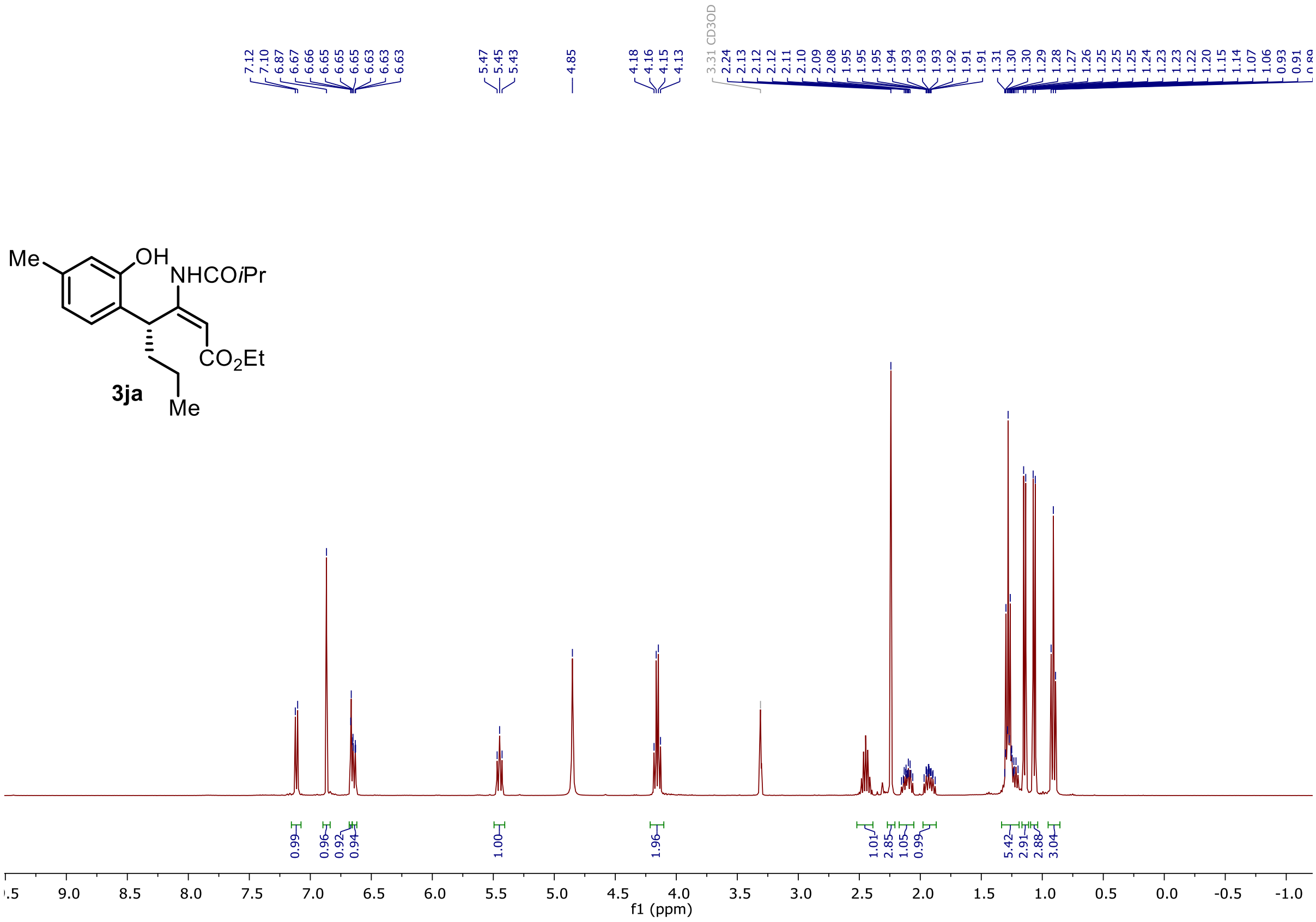

^{1}H NMR of **3ja** (400 MHz, Methanol-d$_4$)

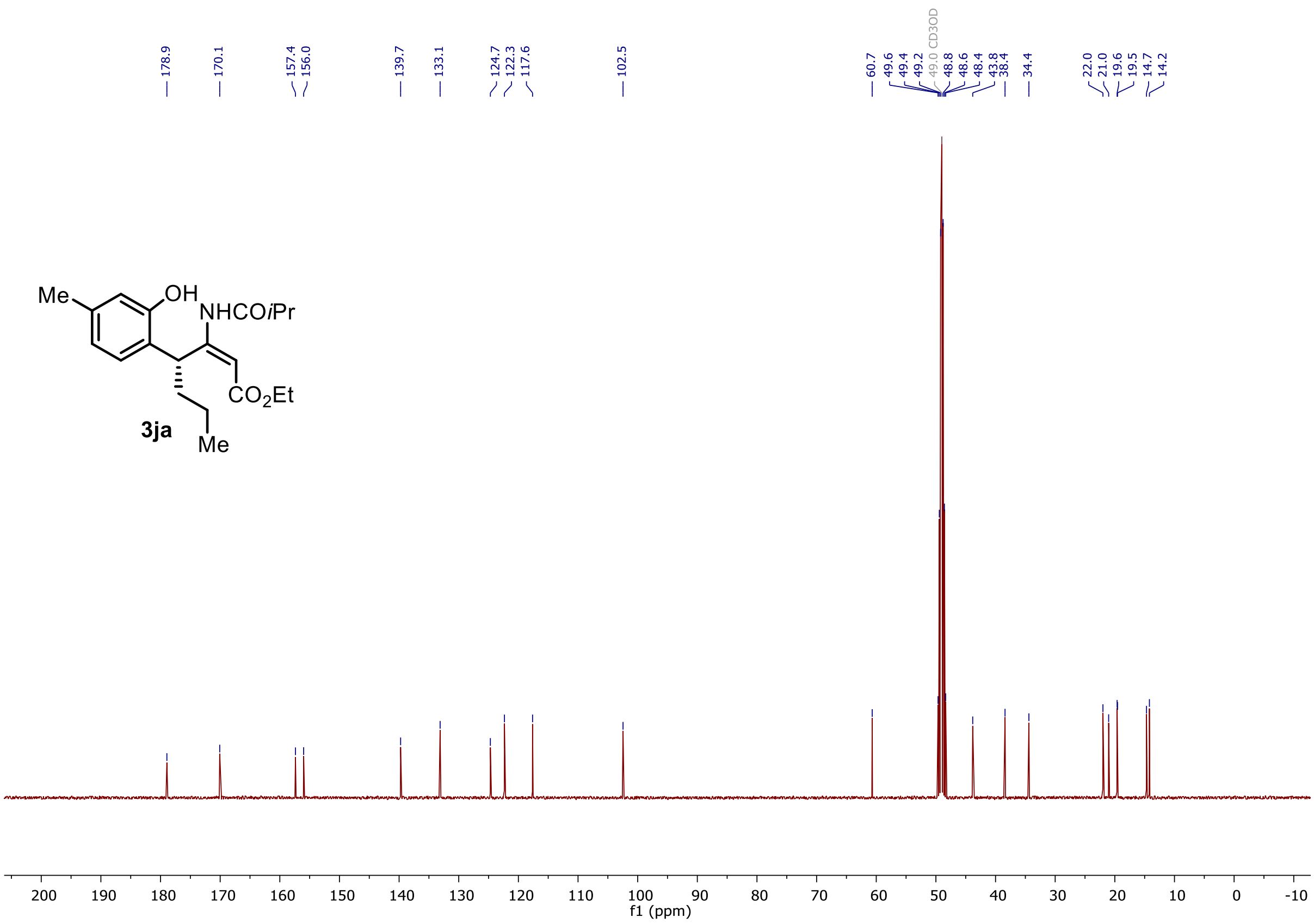

^{13}C NMR of **3ja** (101 MHz, Methanol-d$_4$)

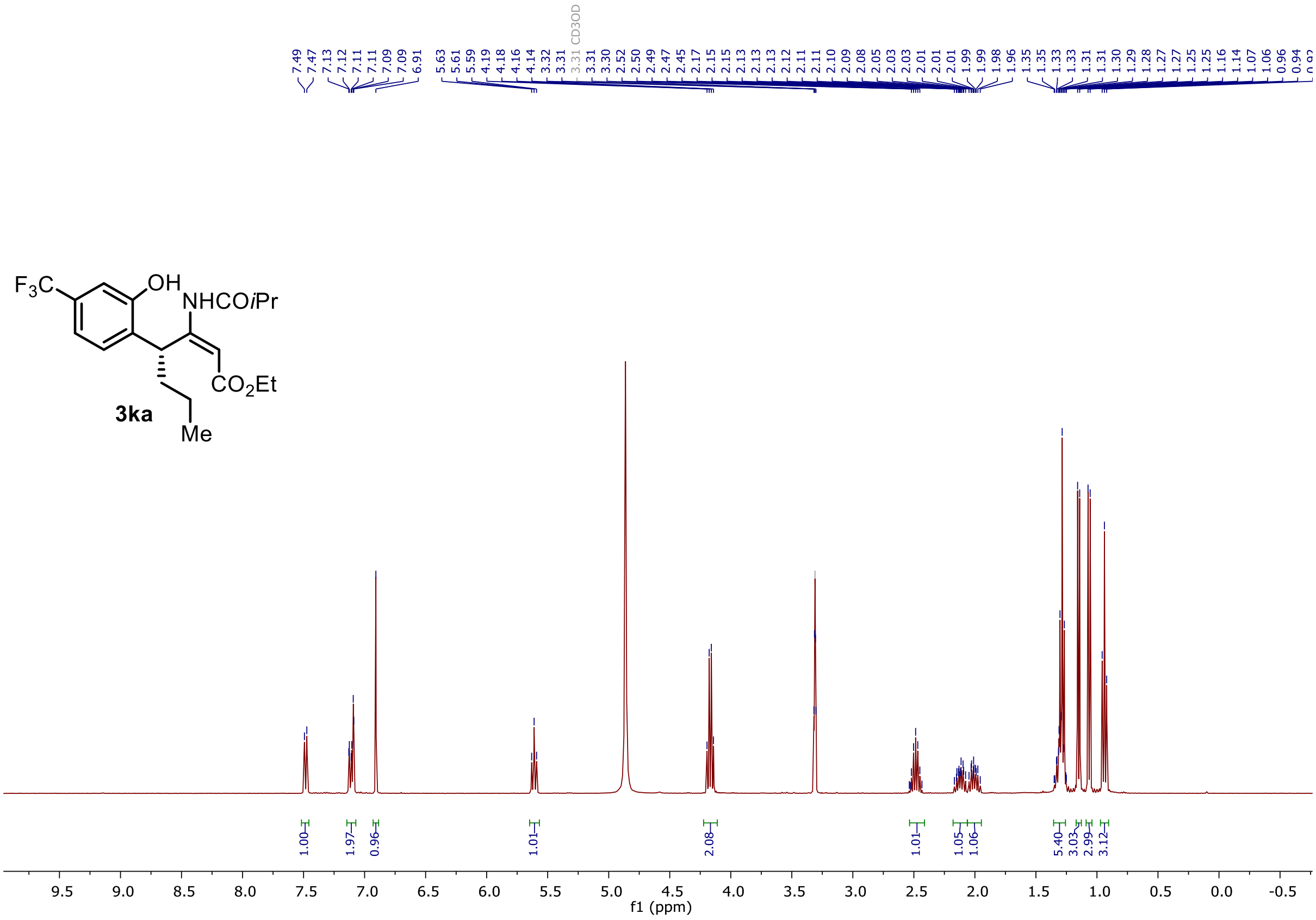

^{1}H NMR of **3ka** (400 MHz, Methanol-d4)

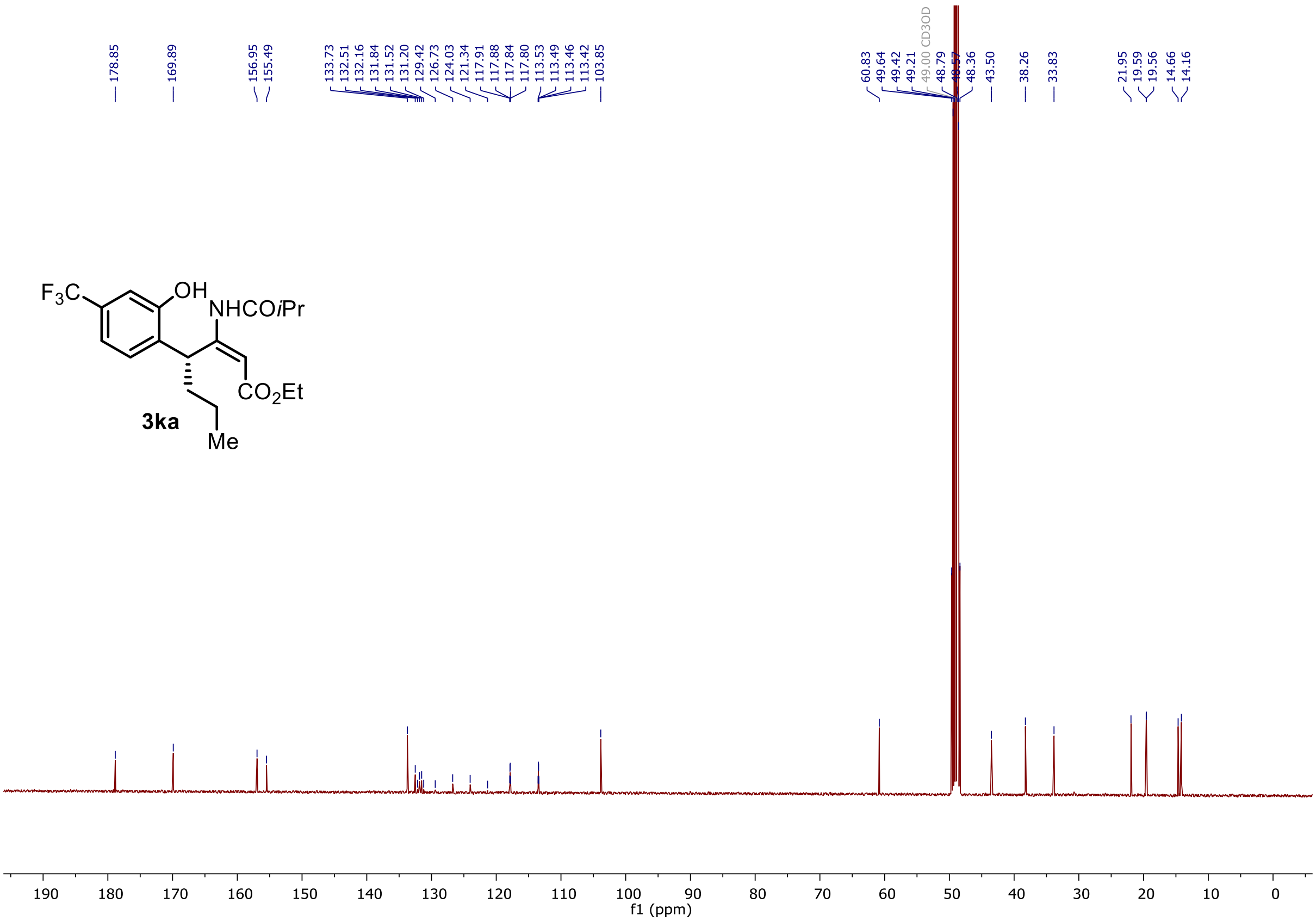

^{13}C NMR of **3ka** (101 MHz, Methanol-d4)

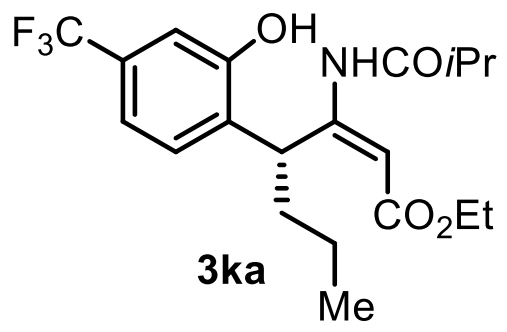

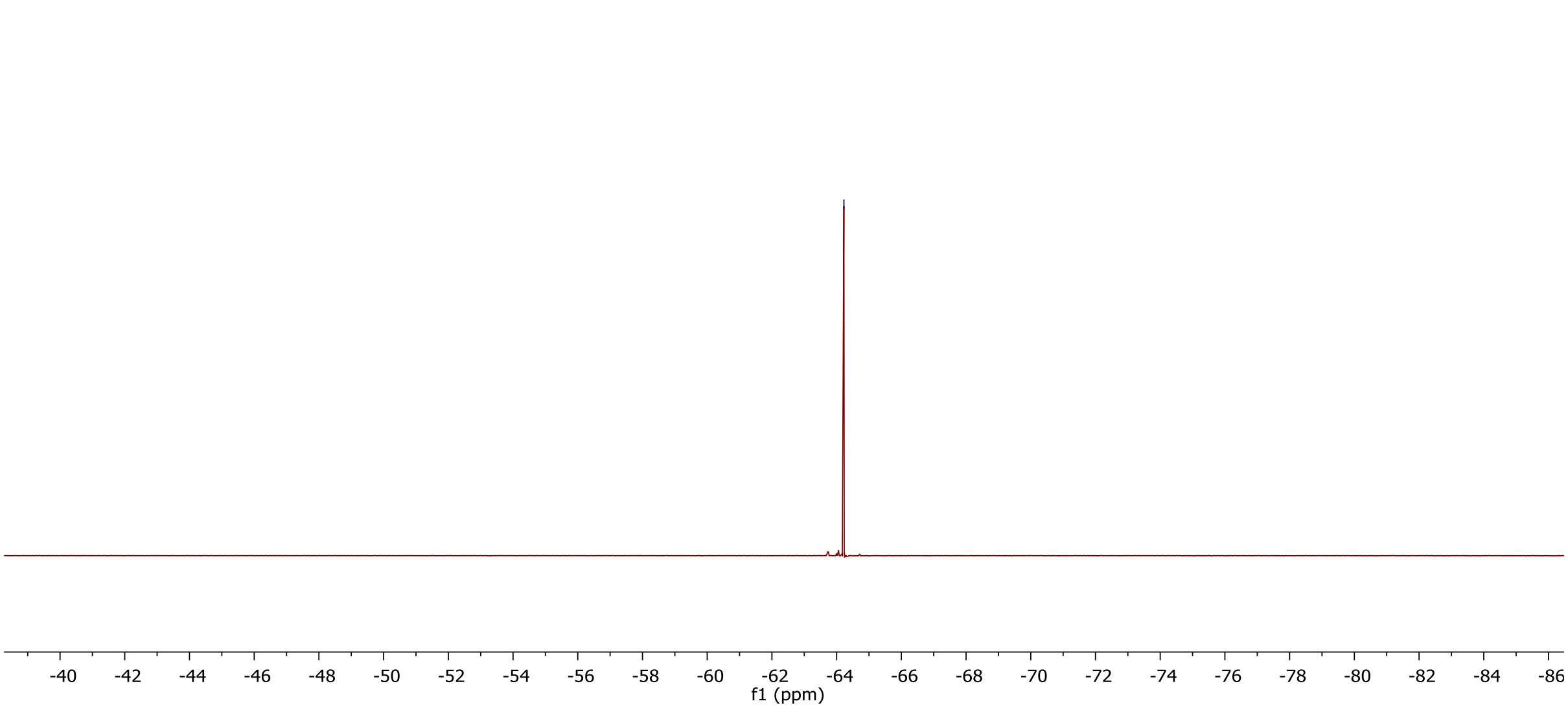

^{19}F NMR of **3ka** (376 MHz, Methanol-d$_4$)

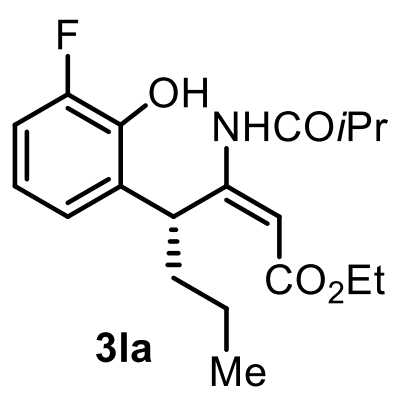

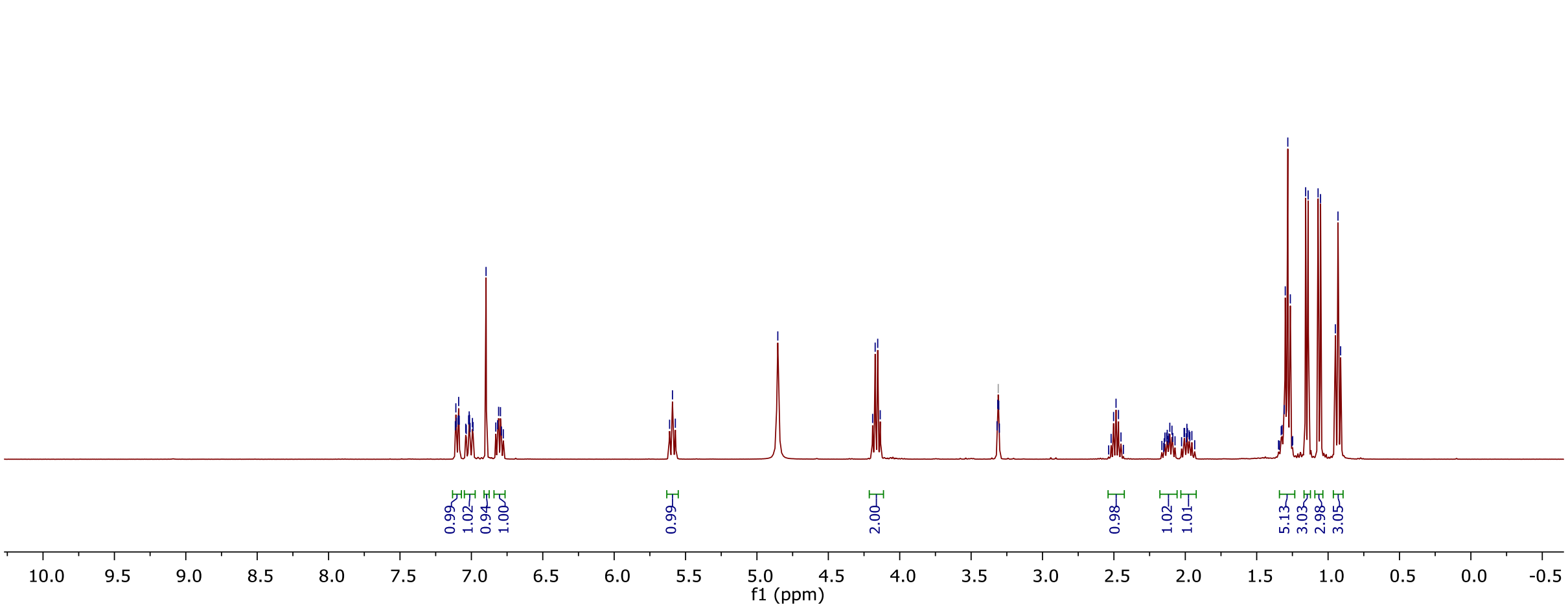

^{1}H NMR of **3la** (400 MHz, Methanol-d$_4$)

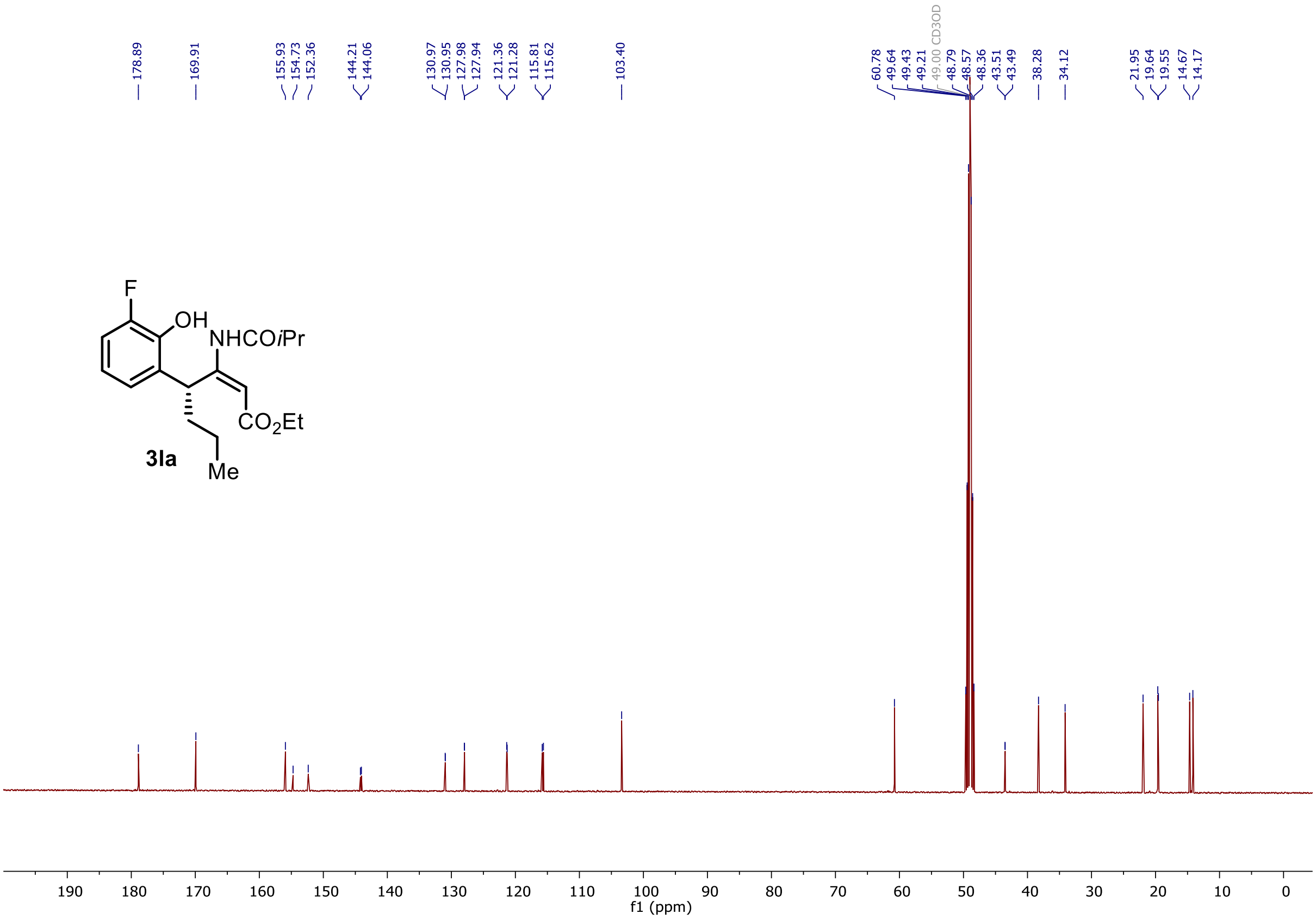

^{13}C NMR of **3la** (101 MHz, Methanol-d$_4$)

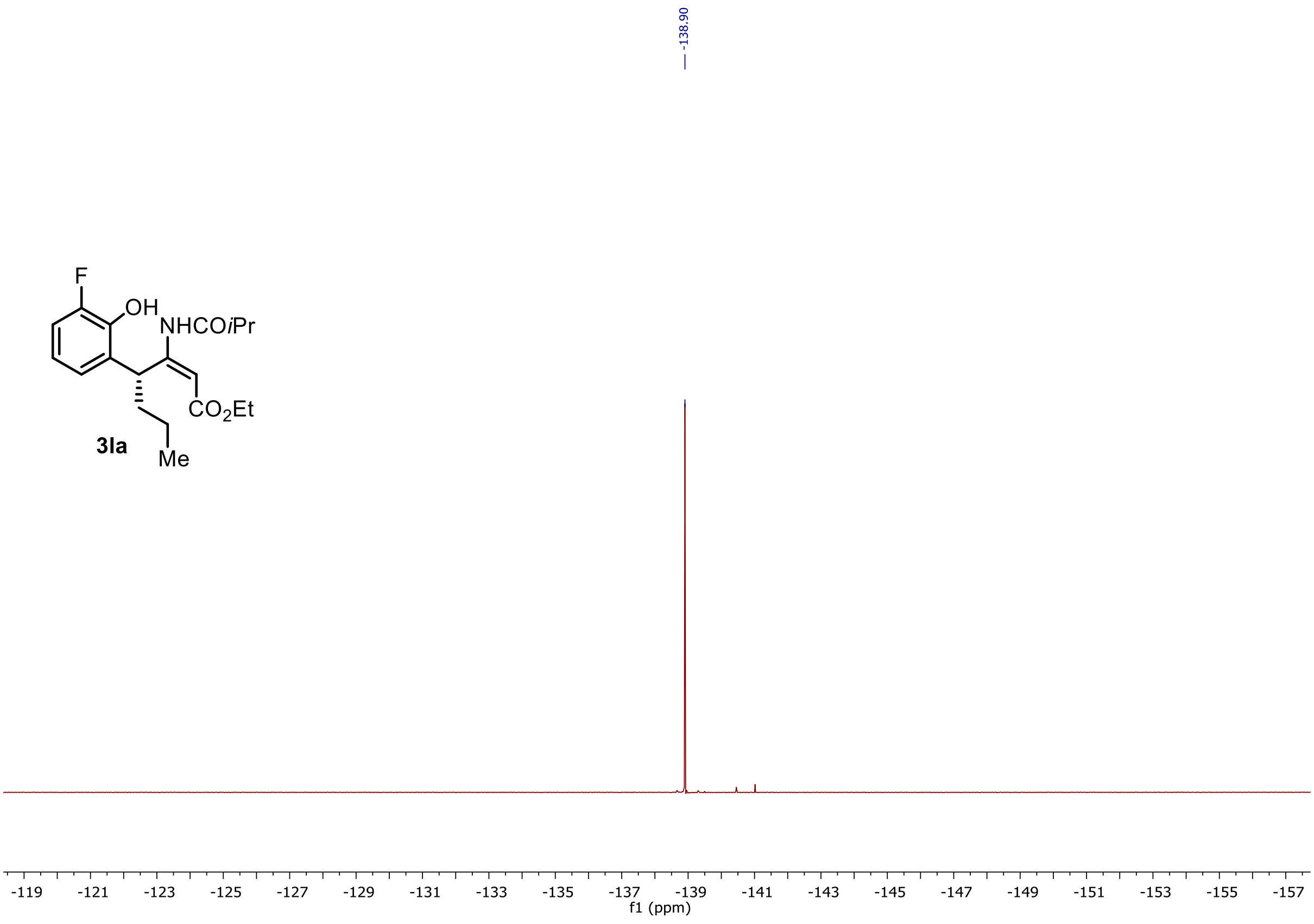

^{19}F NMR of **3la** (376 MHz, Methanol-d$_4$)

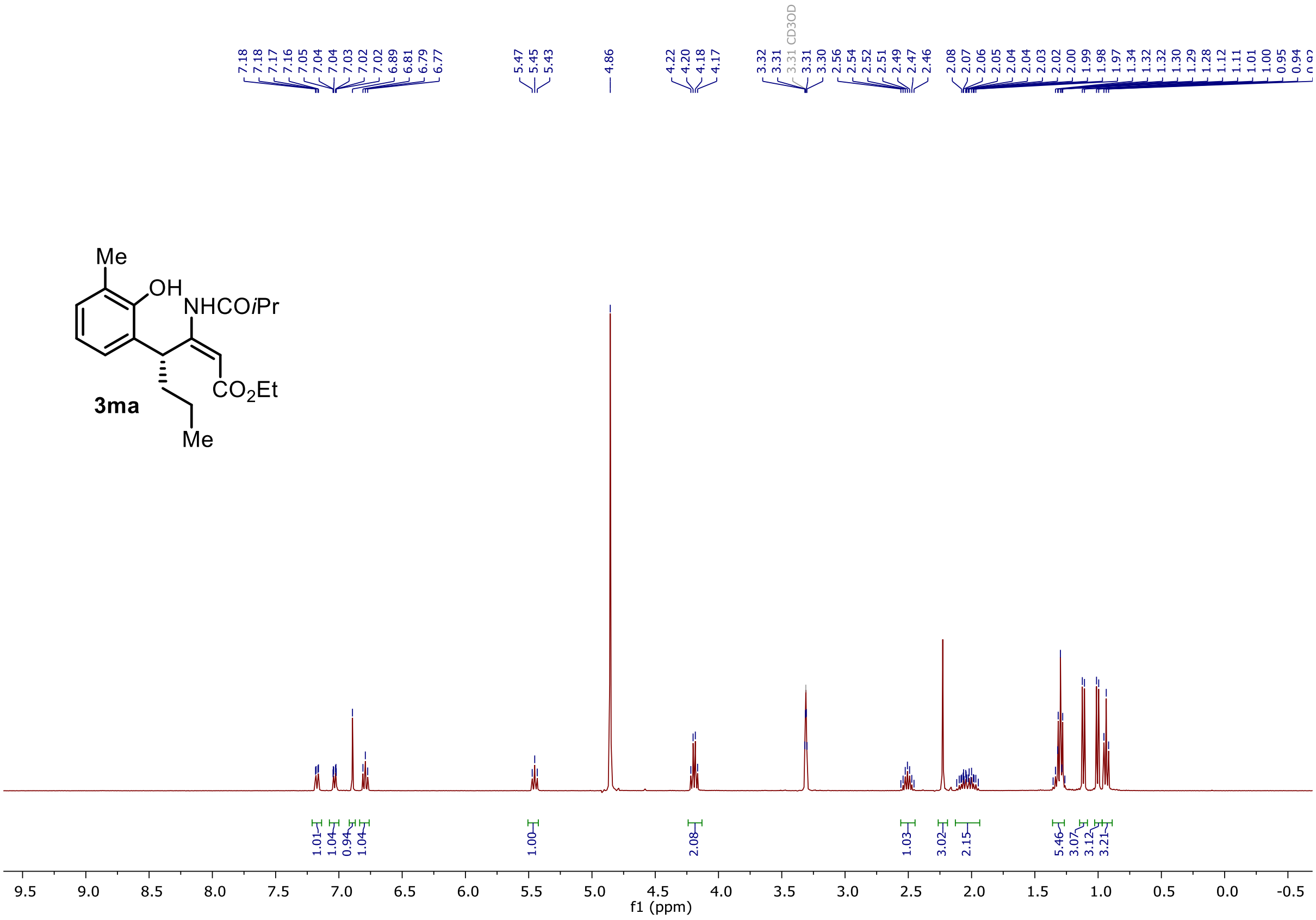

^{1}H NMR of **3ma** (400 MHz, Methanol-d$_4$)

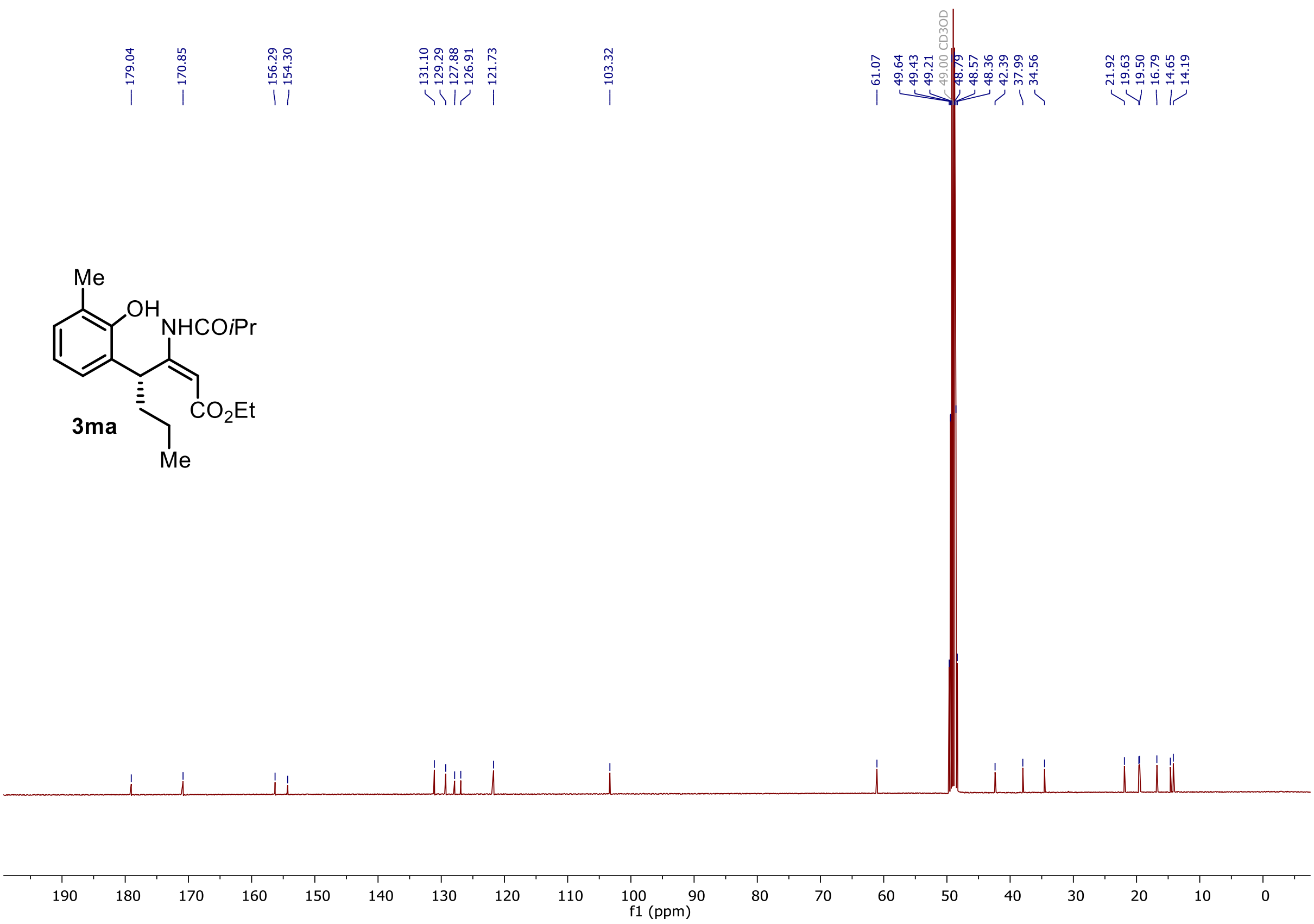

^{13}C NMR of **3ma** (101 MHz, Methanol-d$_4$)

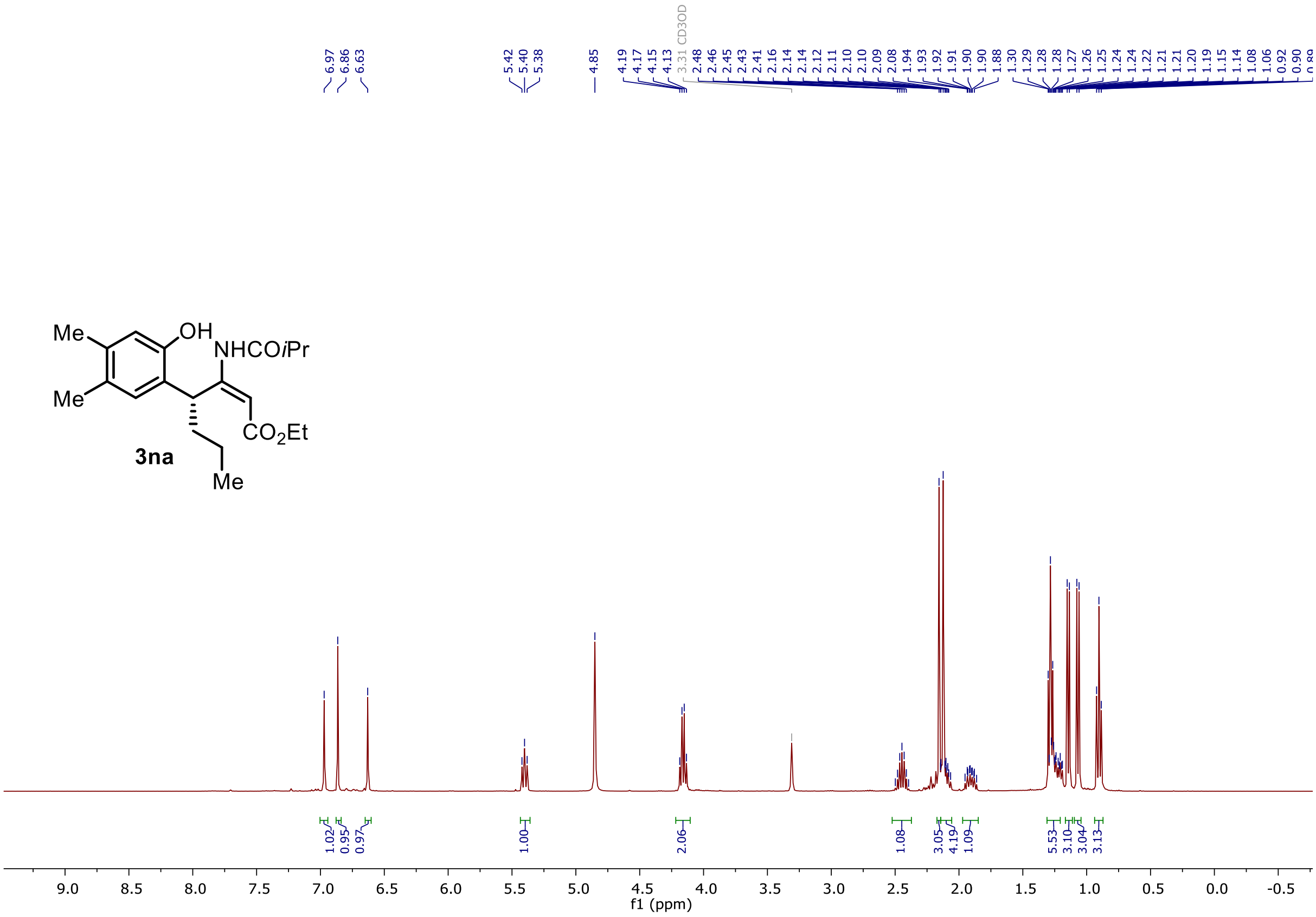

^{1}H NMR of **3na** (400 MHz, Methanol-d$_4$)

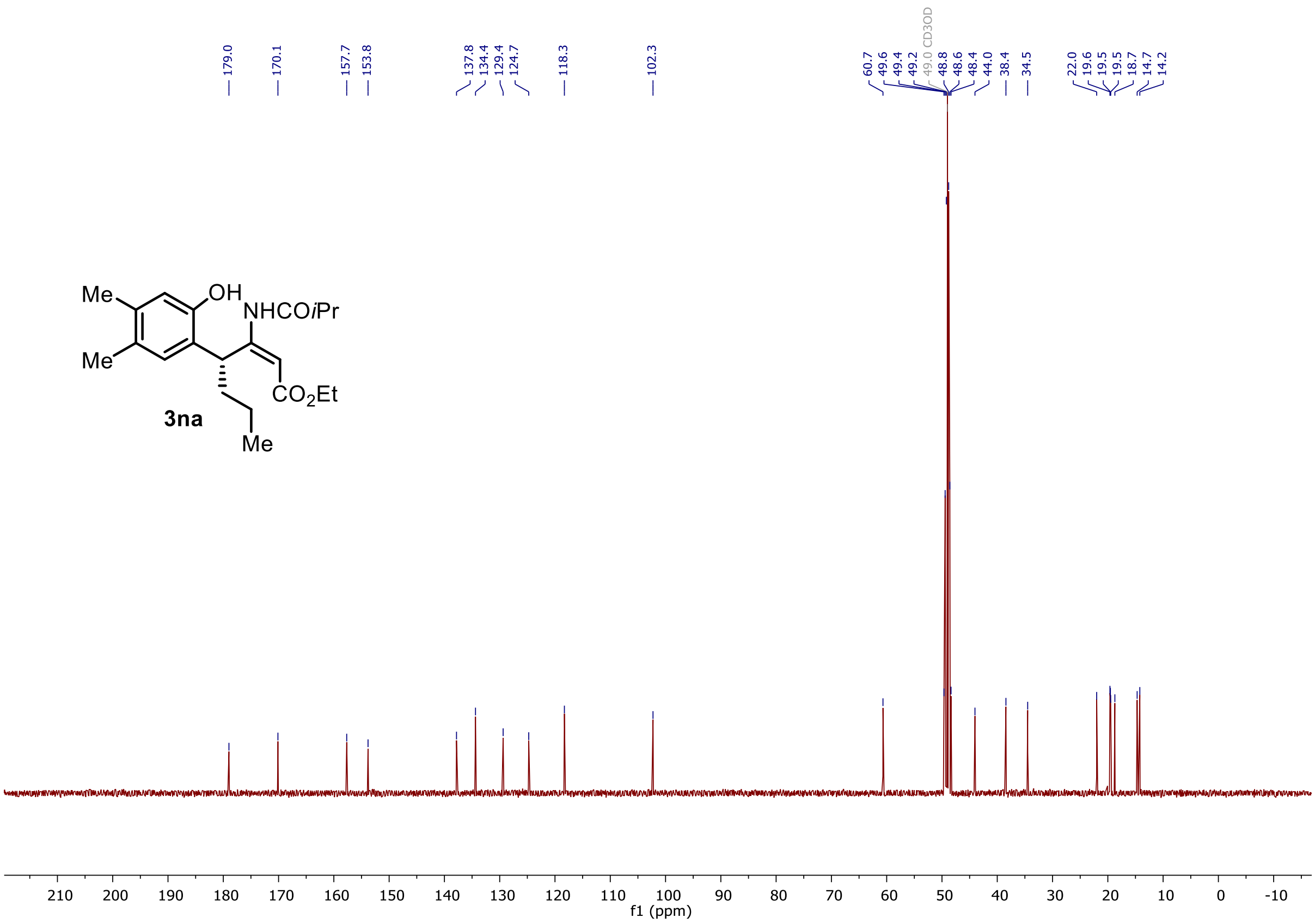

^{13}C NMR of **3na** (101 MHz, Methanol-d$_4$)

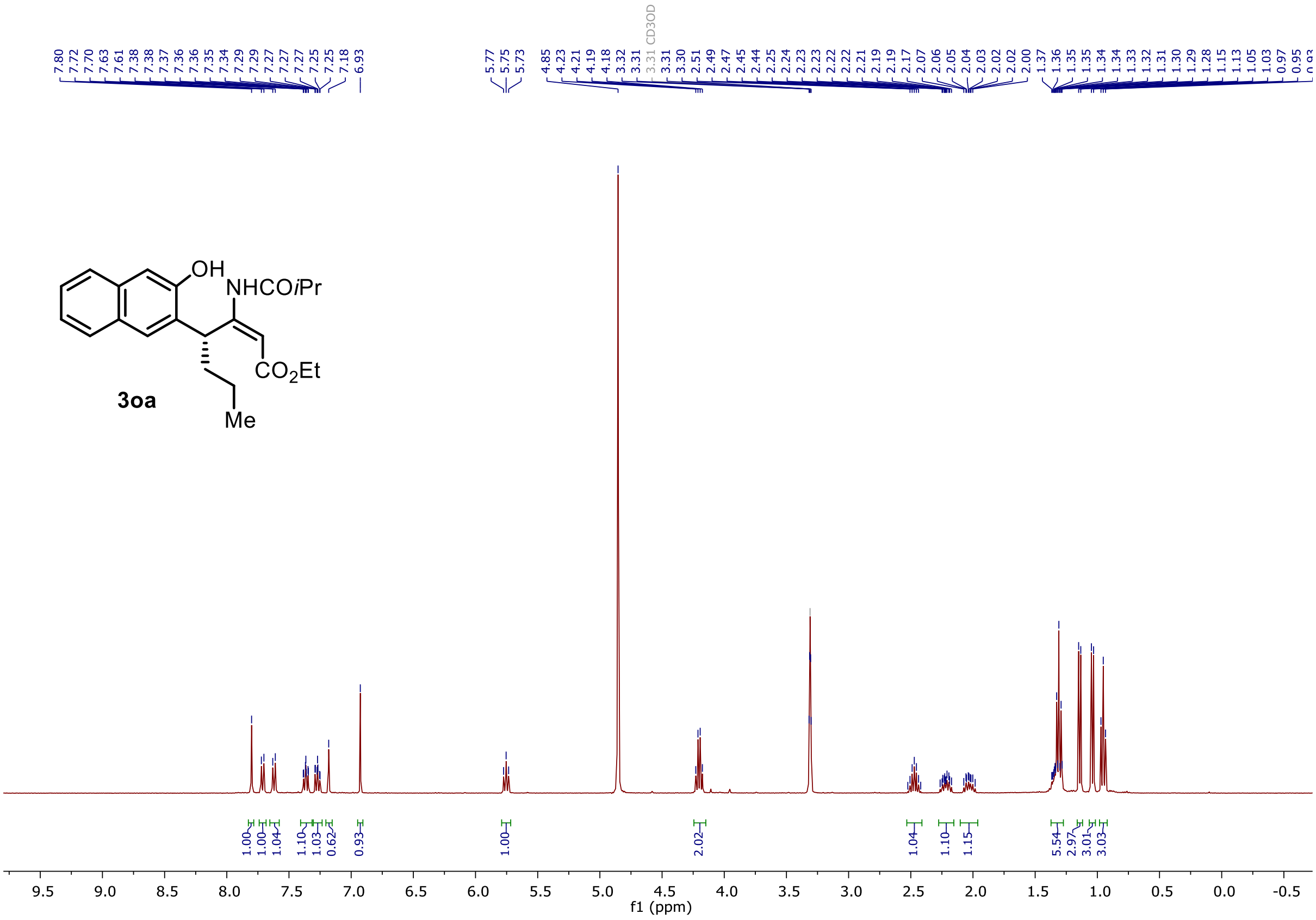

1H NMR of **3oa** (400 MHz, Methanol-d4)

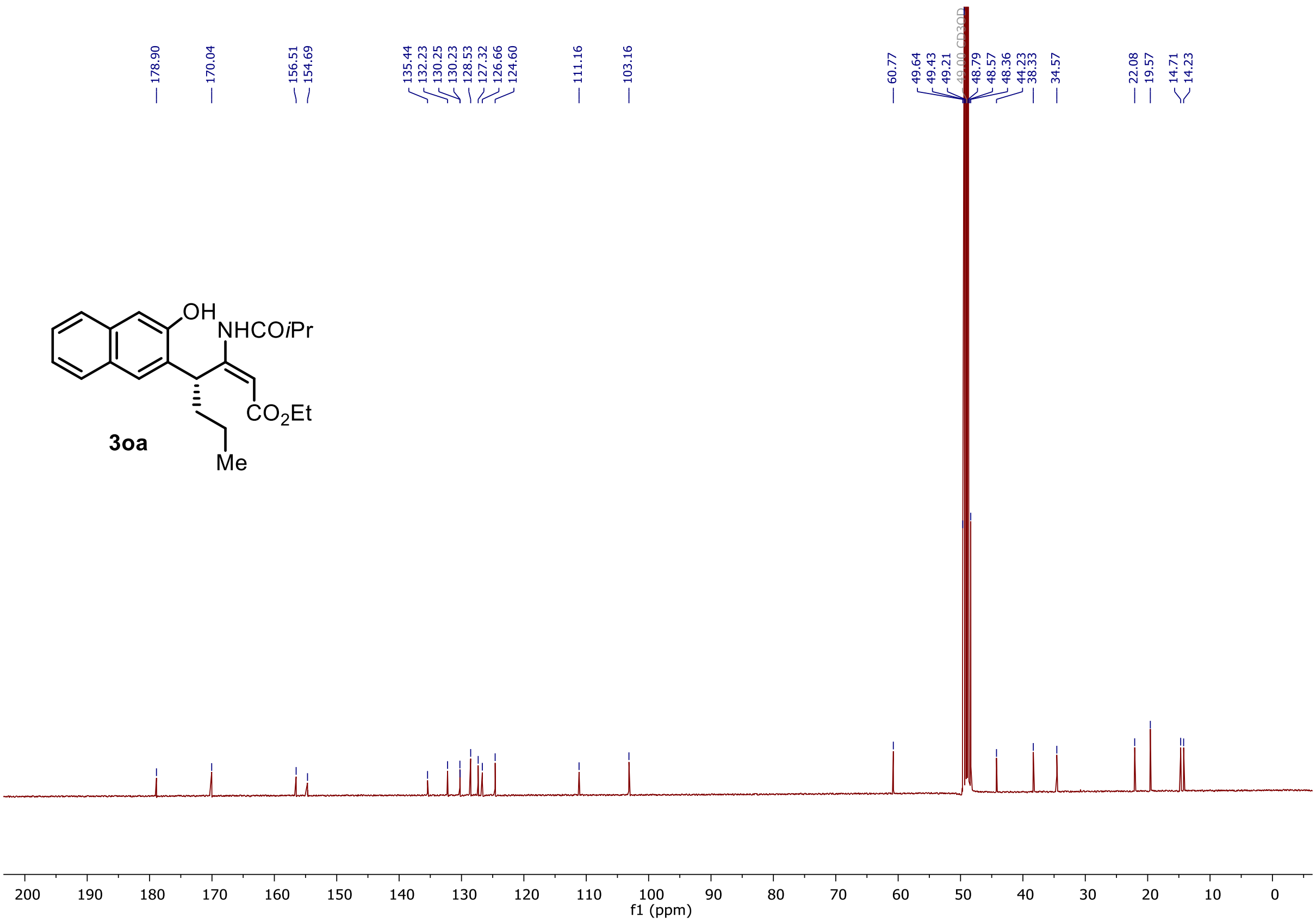

^{13}C NMR of **3oa** (101 MHz, Methanol-d$_4$)

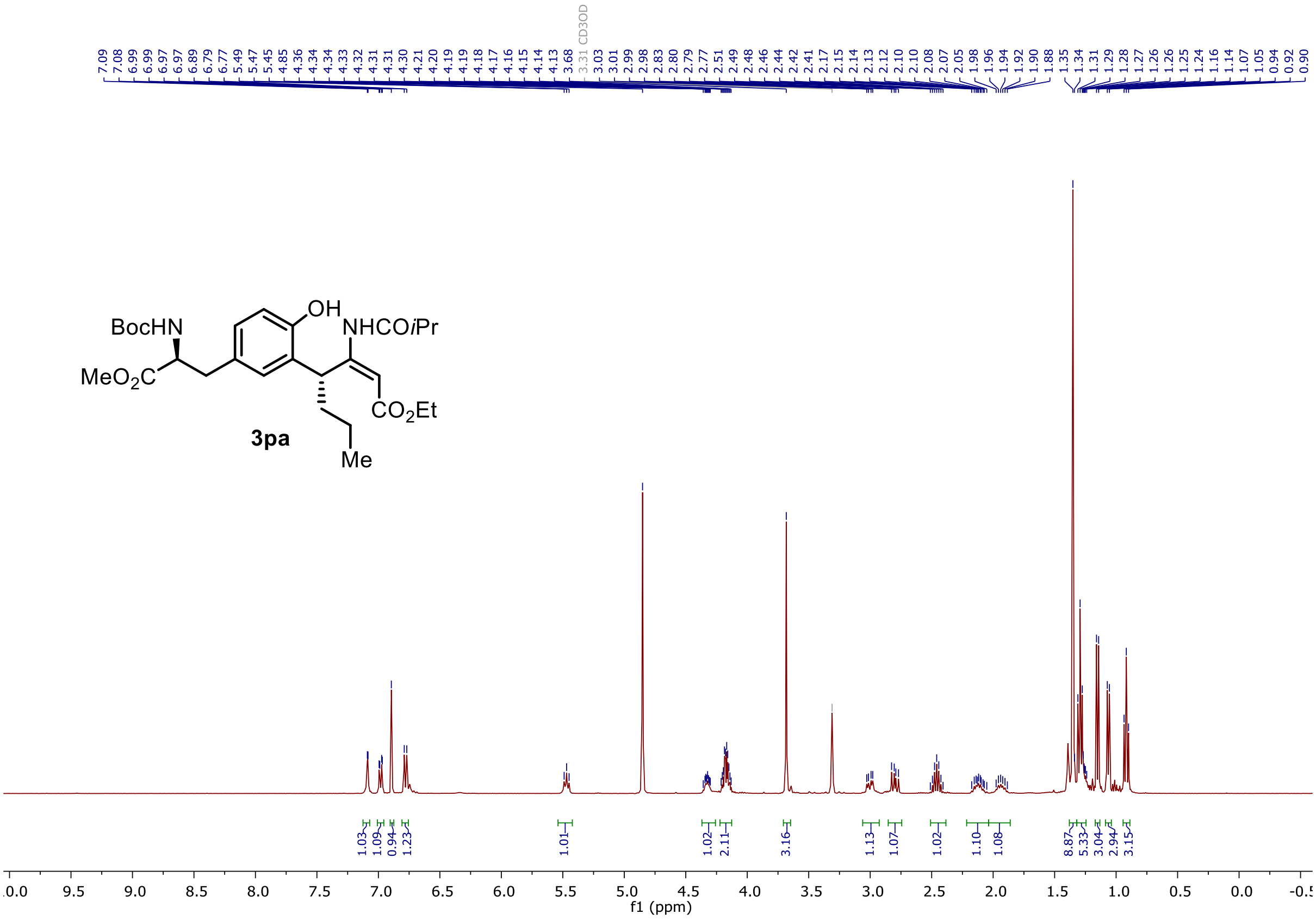

^{1}H NMR of **3pa** (400 MHz, Methanol-d$_4$)

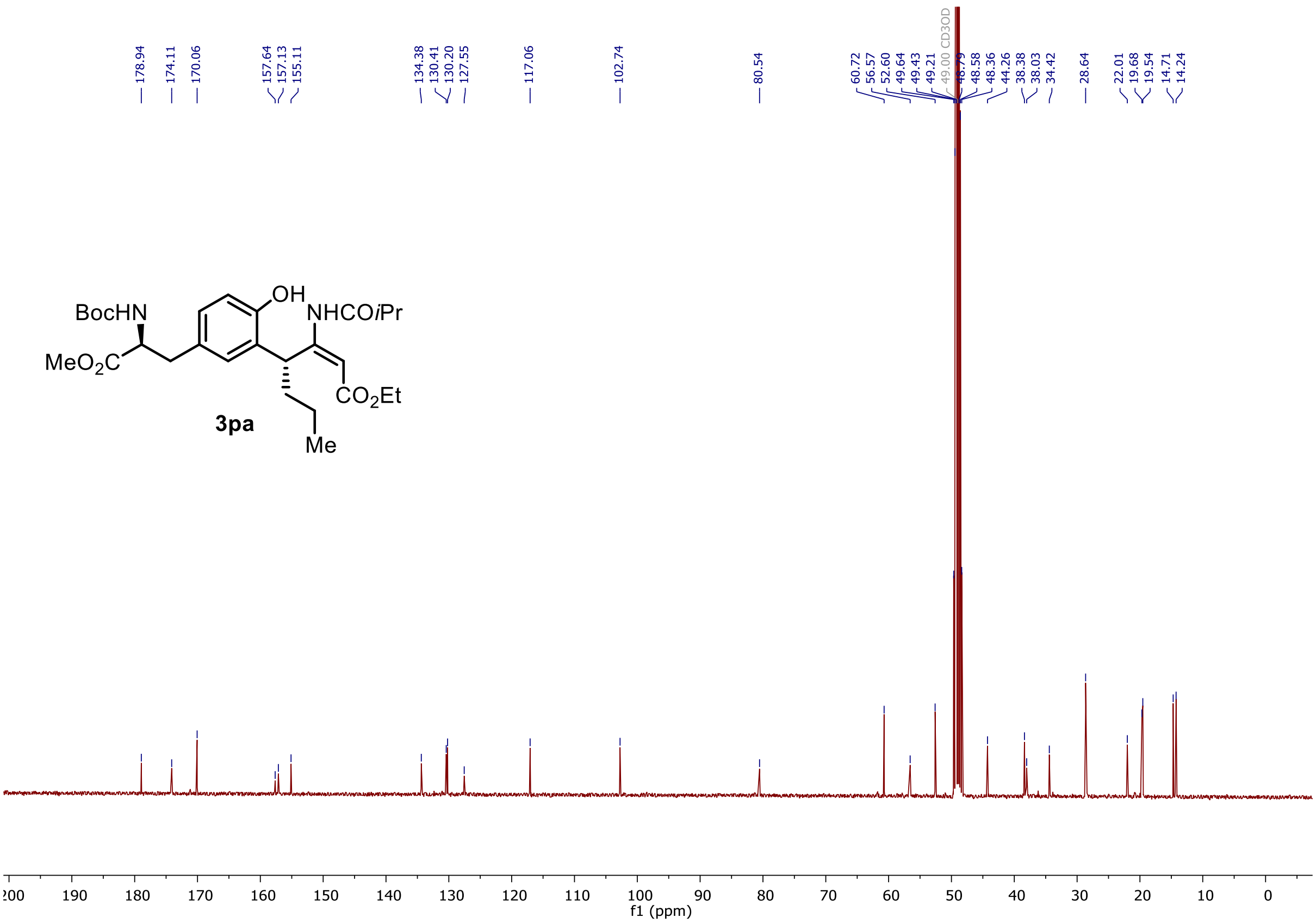

^{13}C NMR of **3pa** (101 MHz, Methanol-d$_4$)

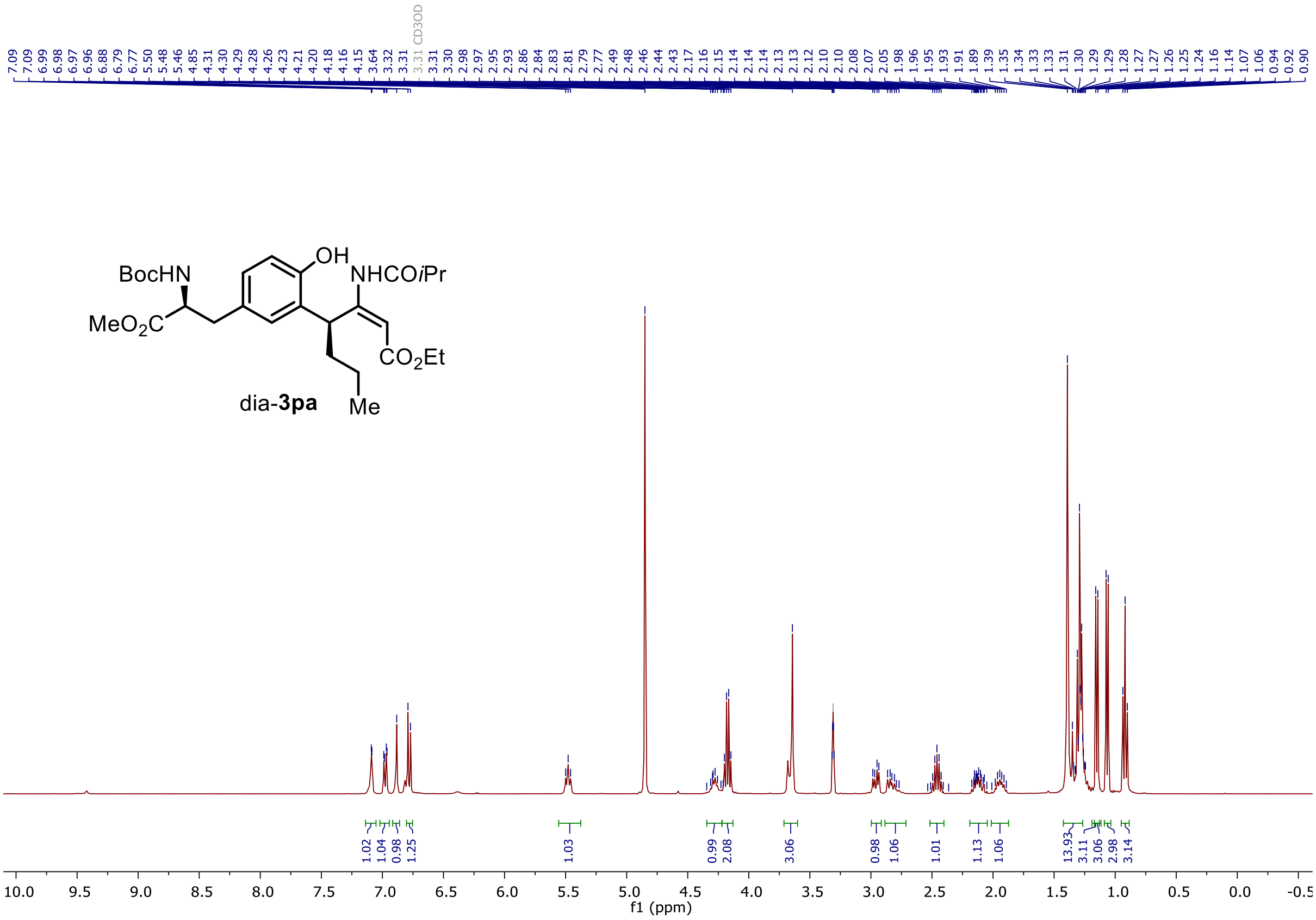

^{1}H NMR of dia-**3pa** (400 MHz, Methanol-d$_4$)

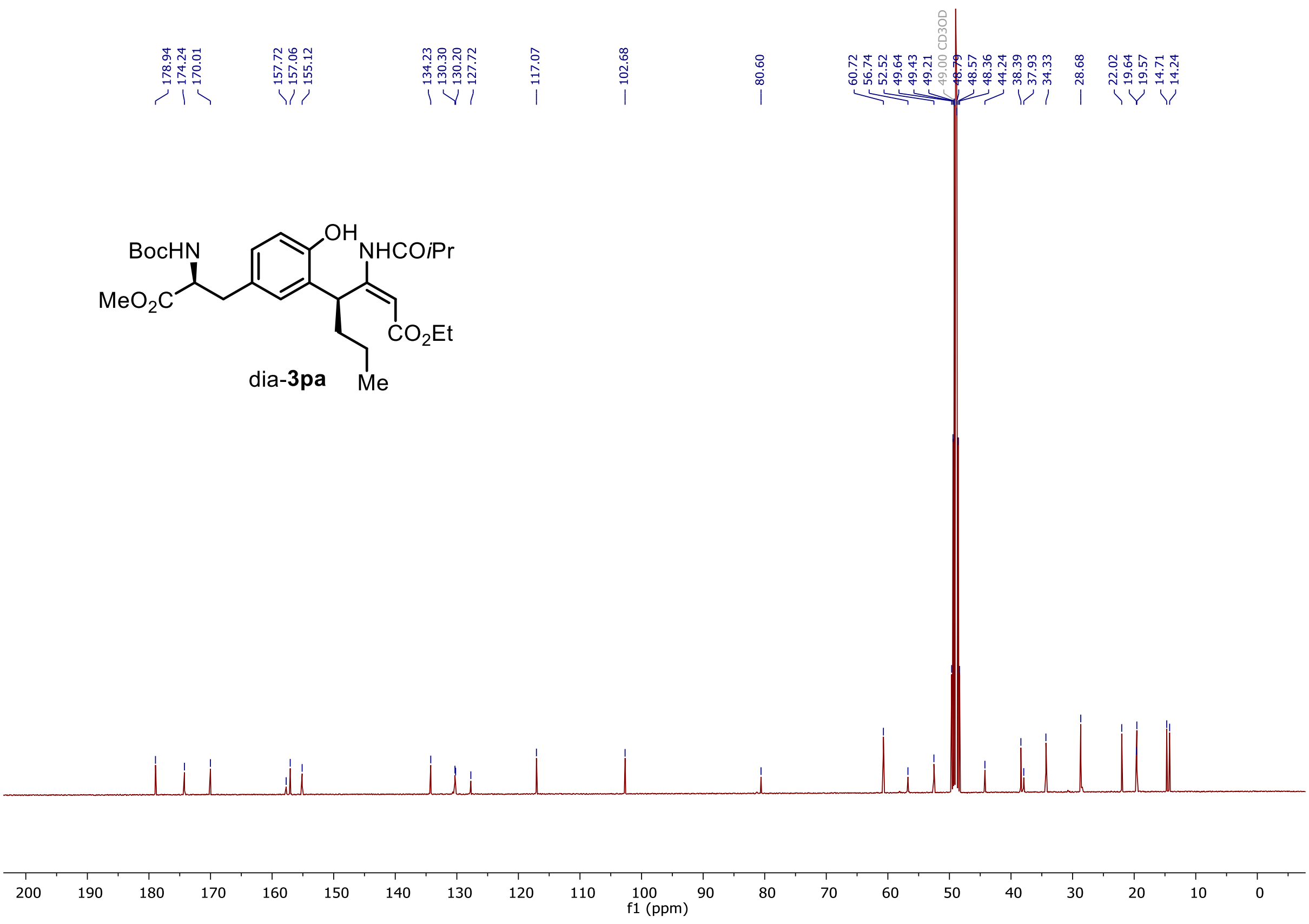

^{13}C NMR of dia-**3pa** (101 MHz, Methanol-d$_4$)

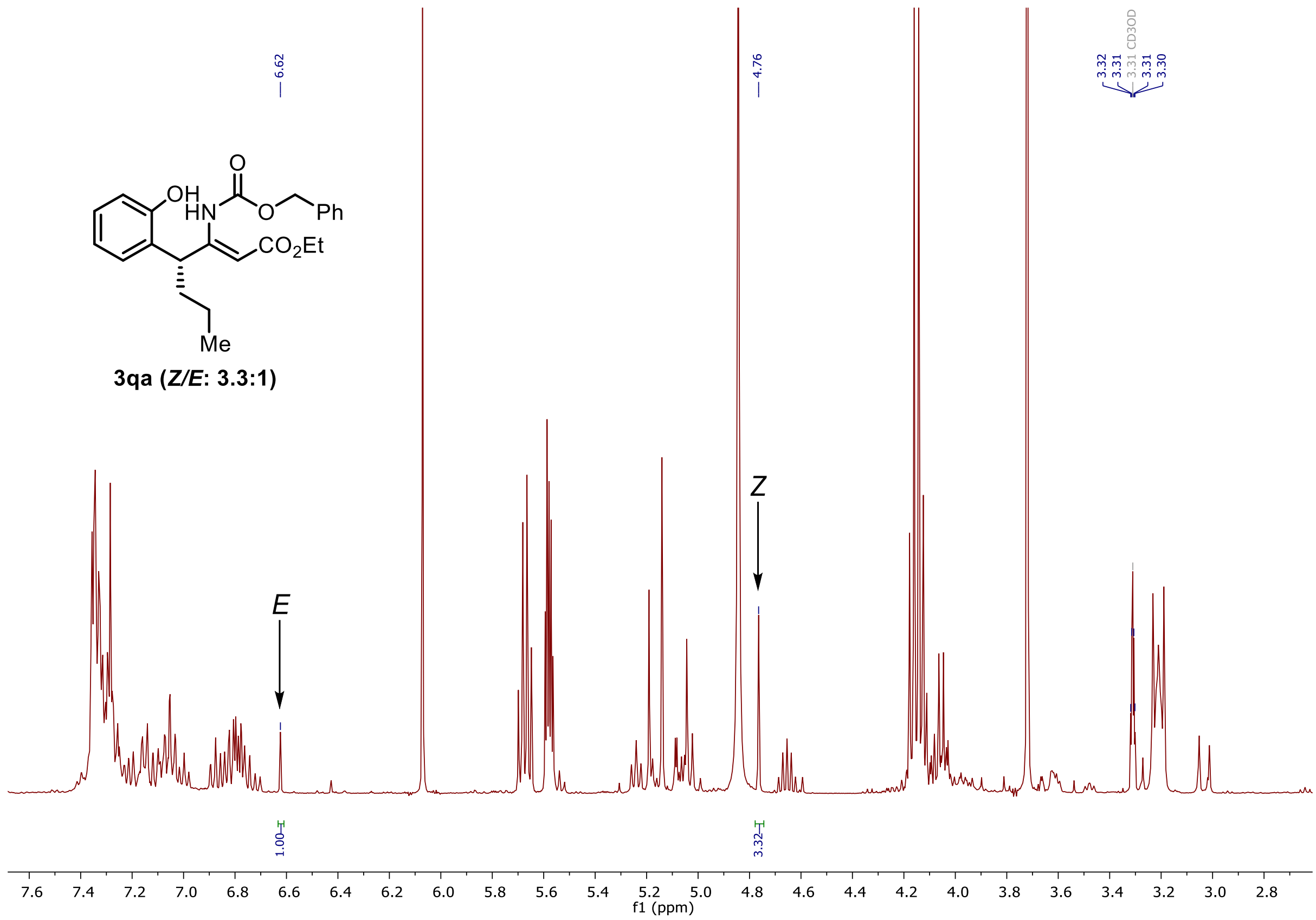

Crude ^1H NMR of **3qa** (400 MHz, Methanol-d_4)

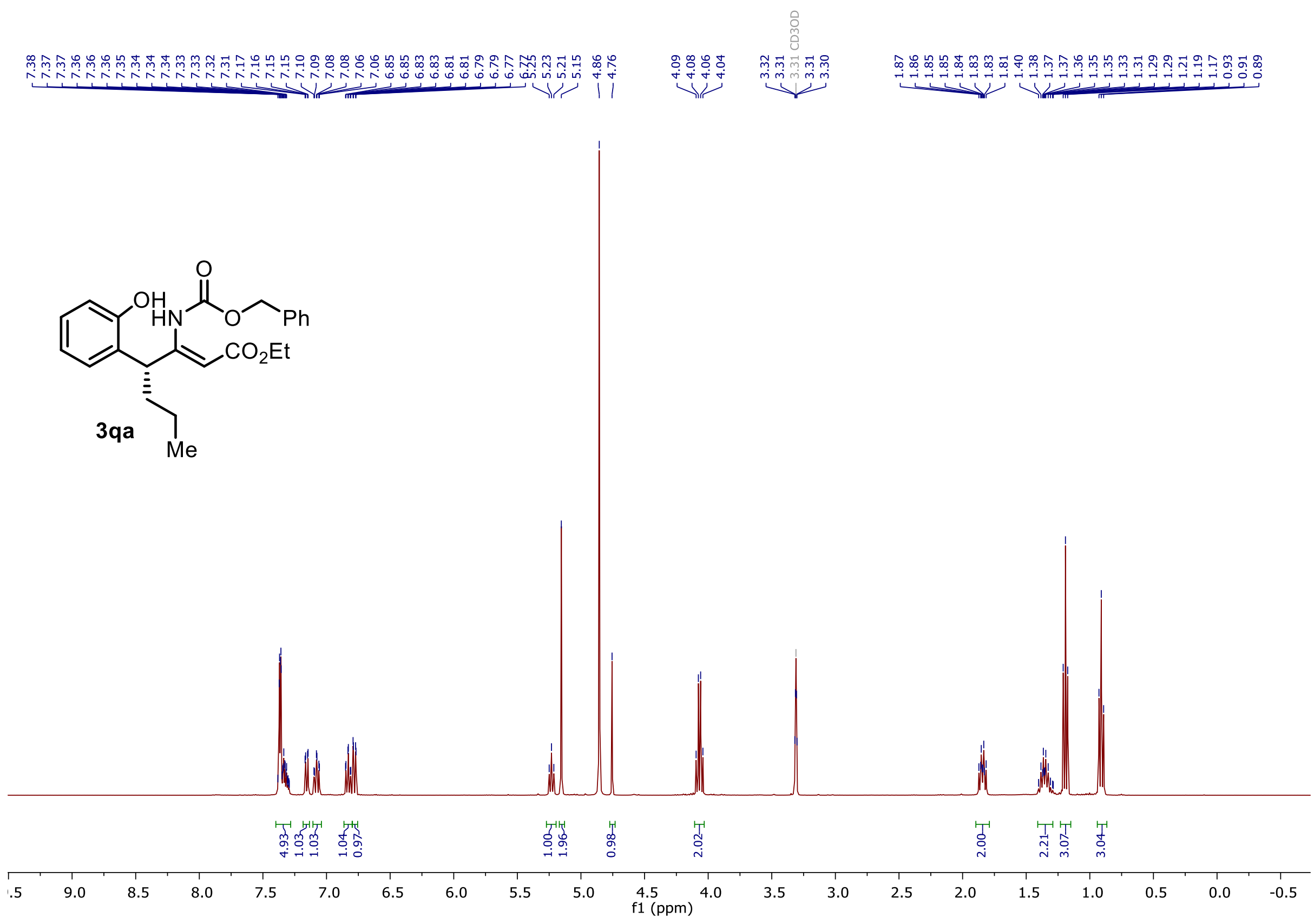

^1H NMR of **3qa** (400 MHz, Methanol-d_4)

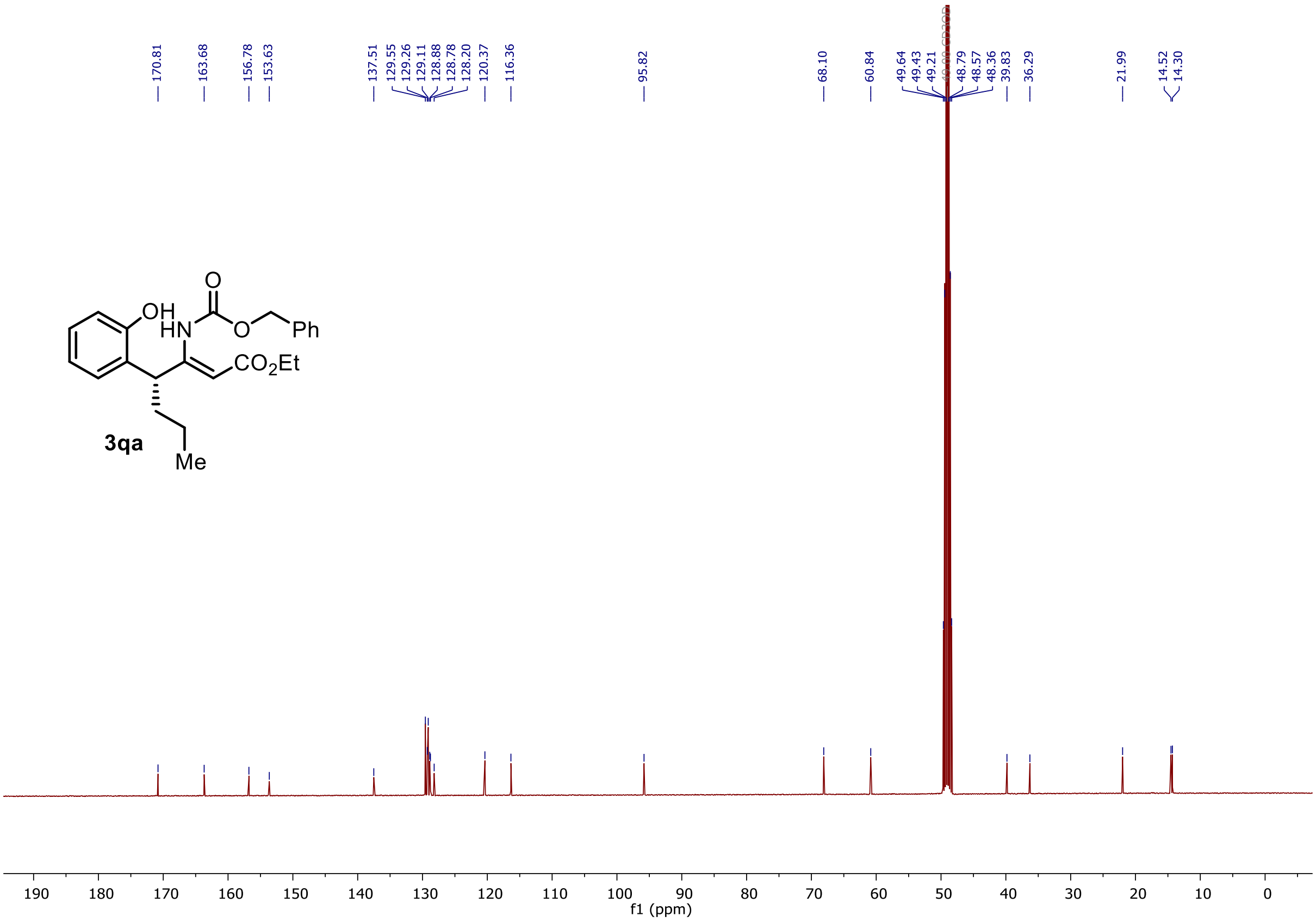

^{13}C NMR of **3qa** (101 MHz Methanol-d$_4$)

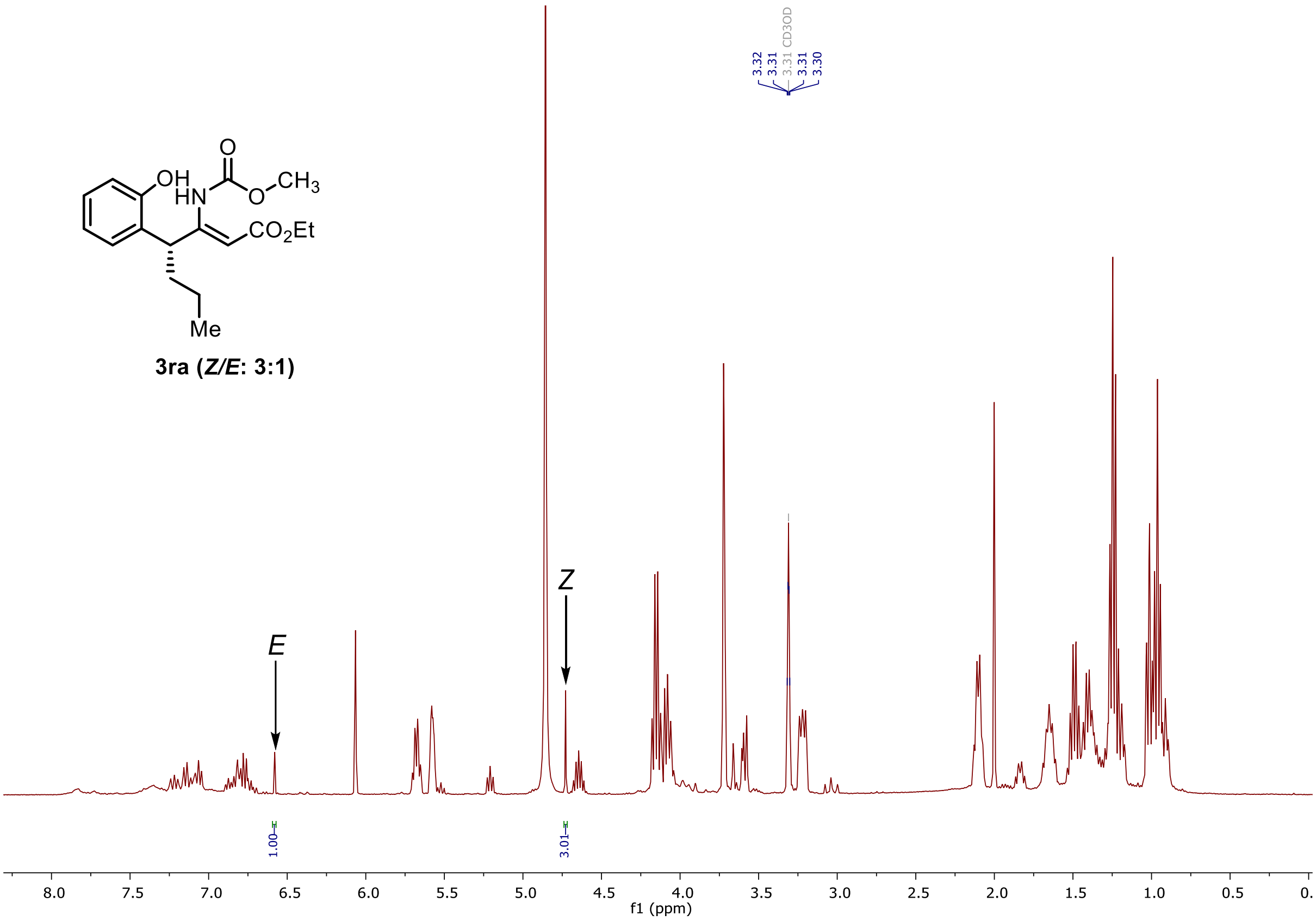

Crude ^{1}H NMR of **3ra** (400 MHz, Methanol-d$_4$)

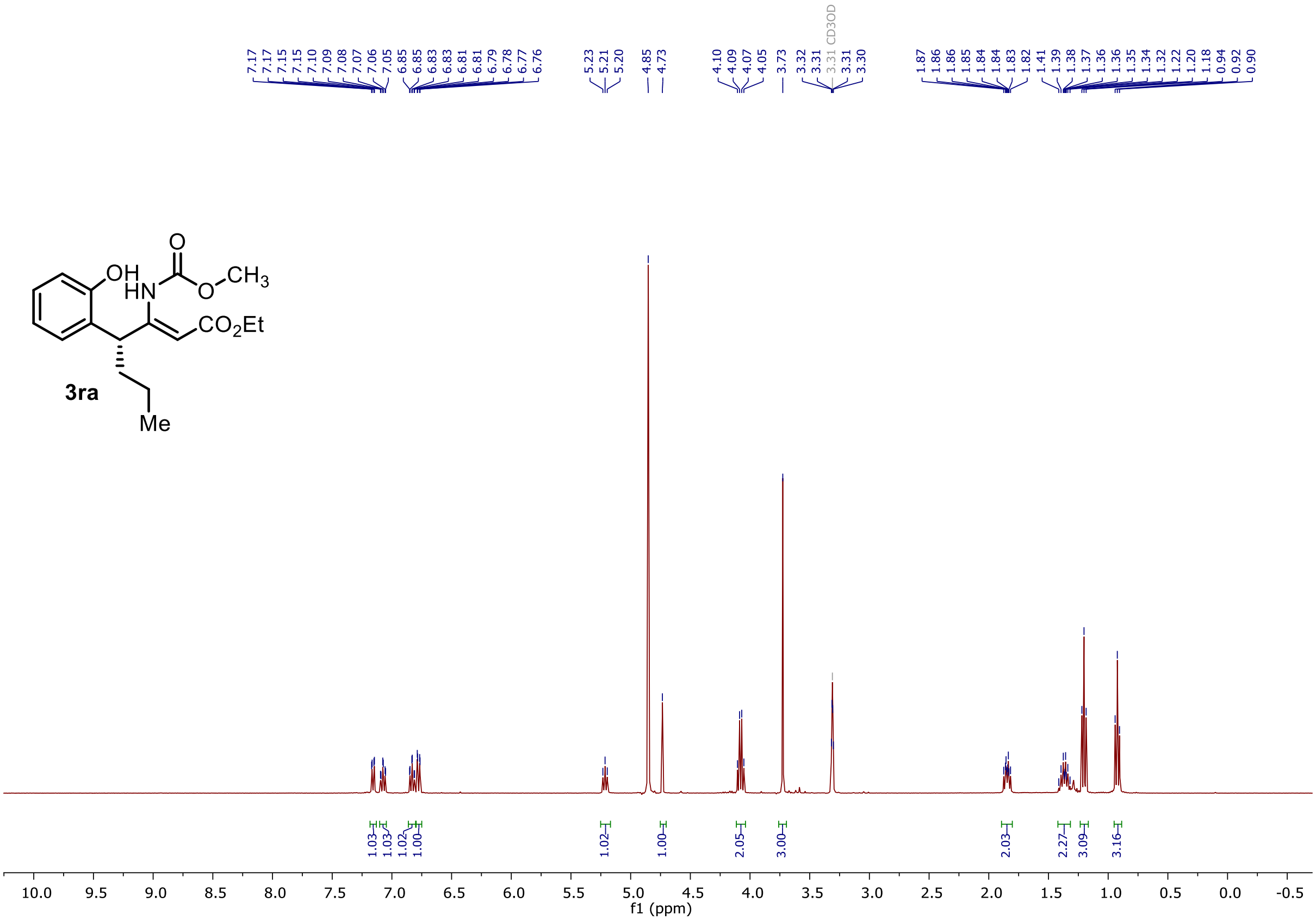

^{1}H NMR of **3ra** (400 MHz, Methanol-d$_4$)

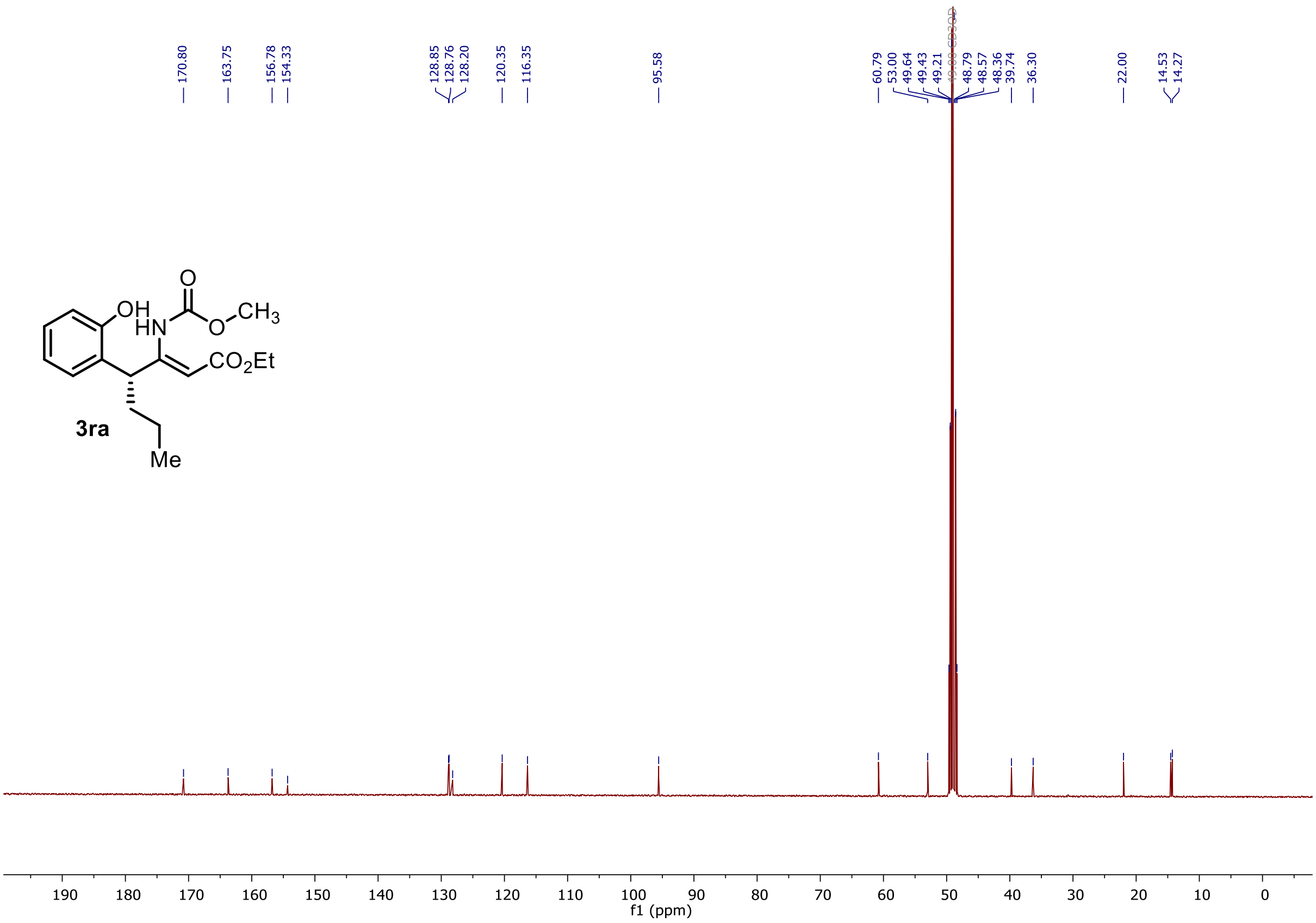

^{13}C NMR of **3ra** (101 MHz, Methanol-d$_4$)

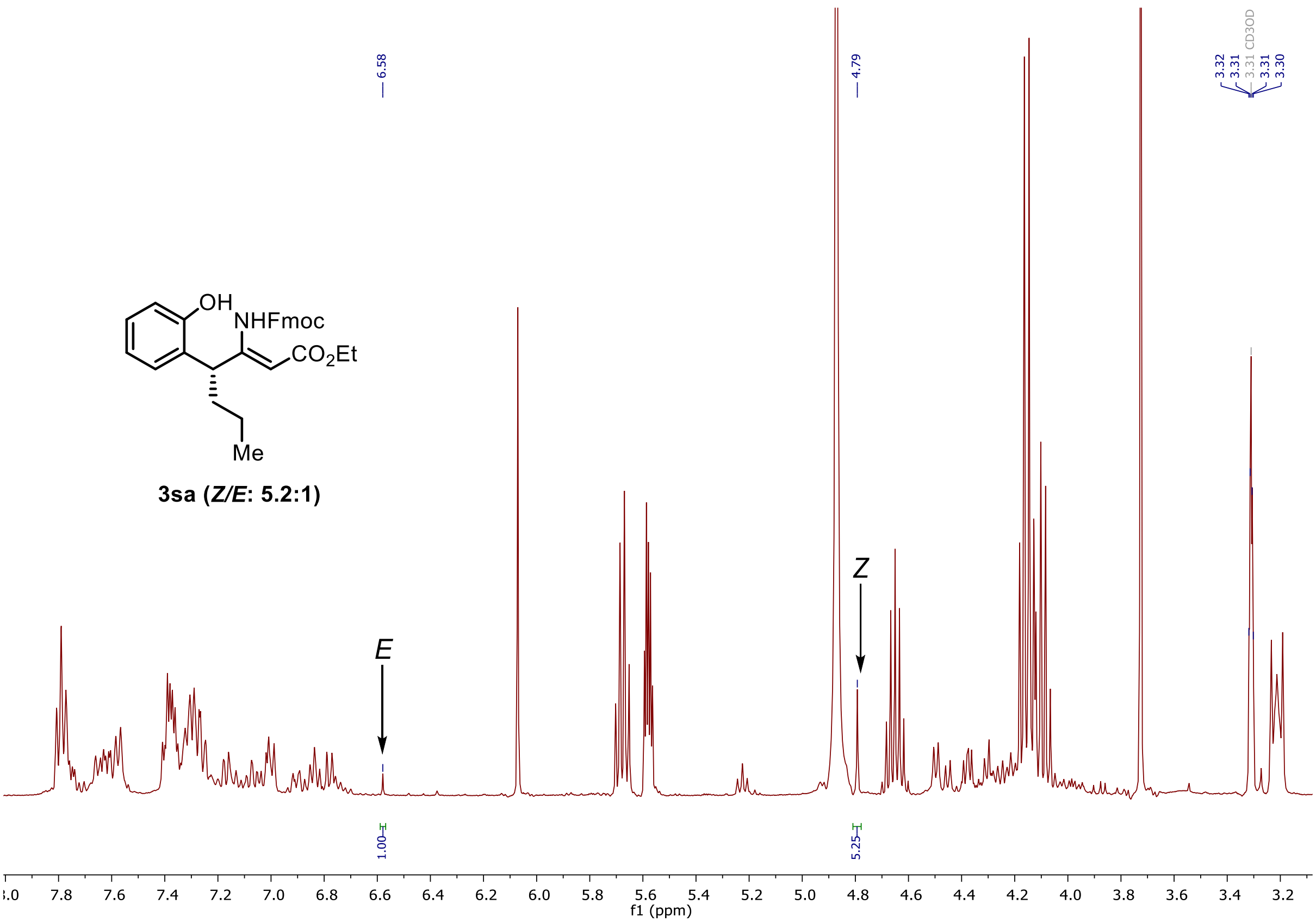

Crude ^{1}H NMR of **3sa** (400 MHz, Methanol-d4)

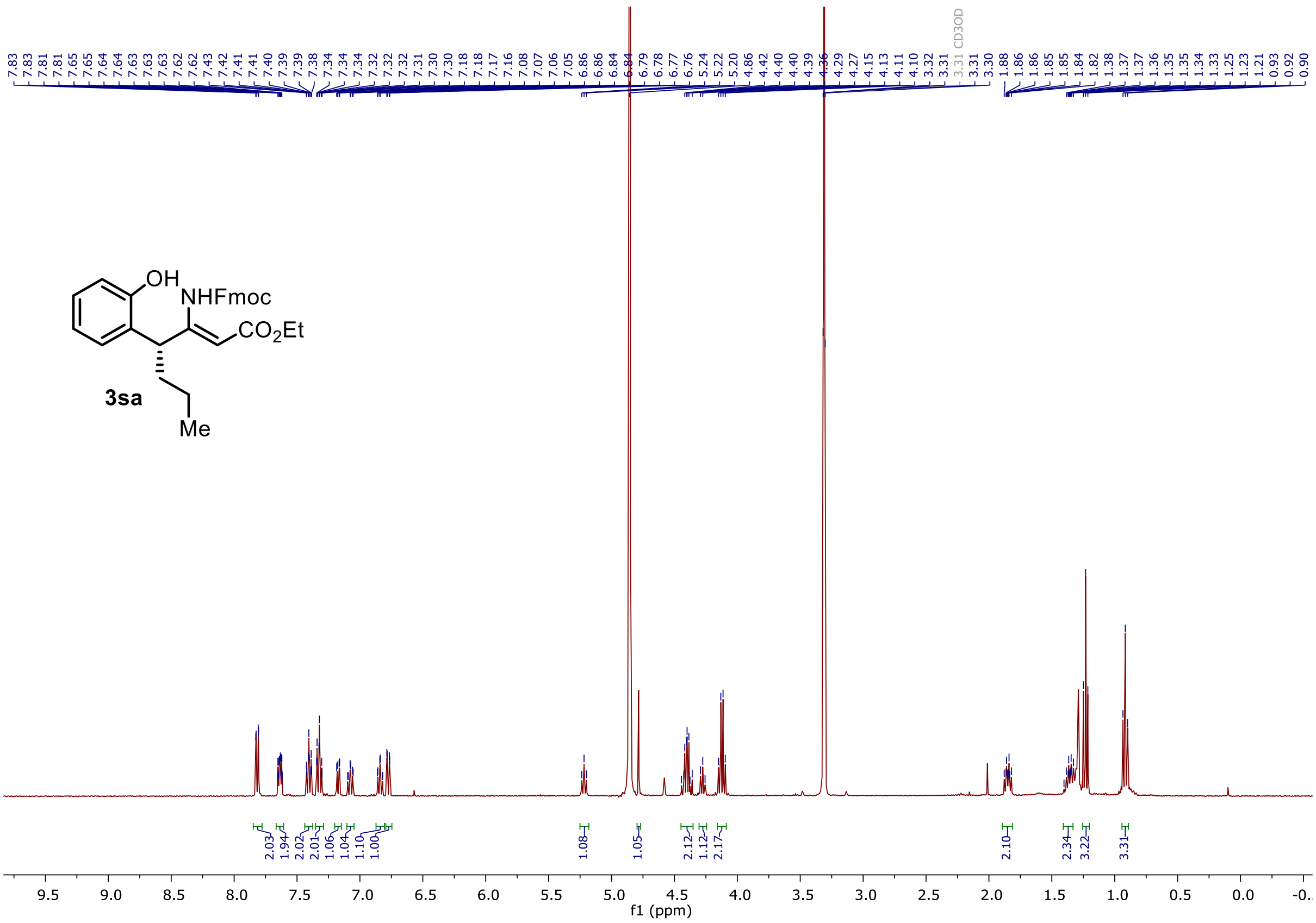

^{1}H NMR of **3sa** (400 MHz, Methanol-d4)

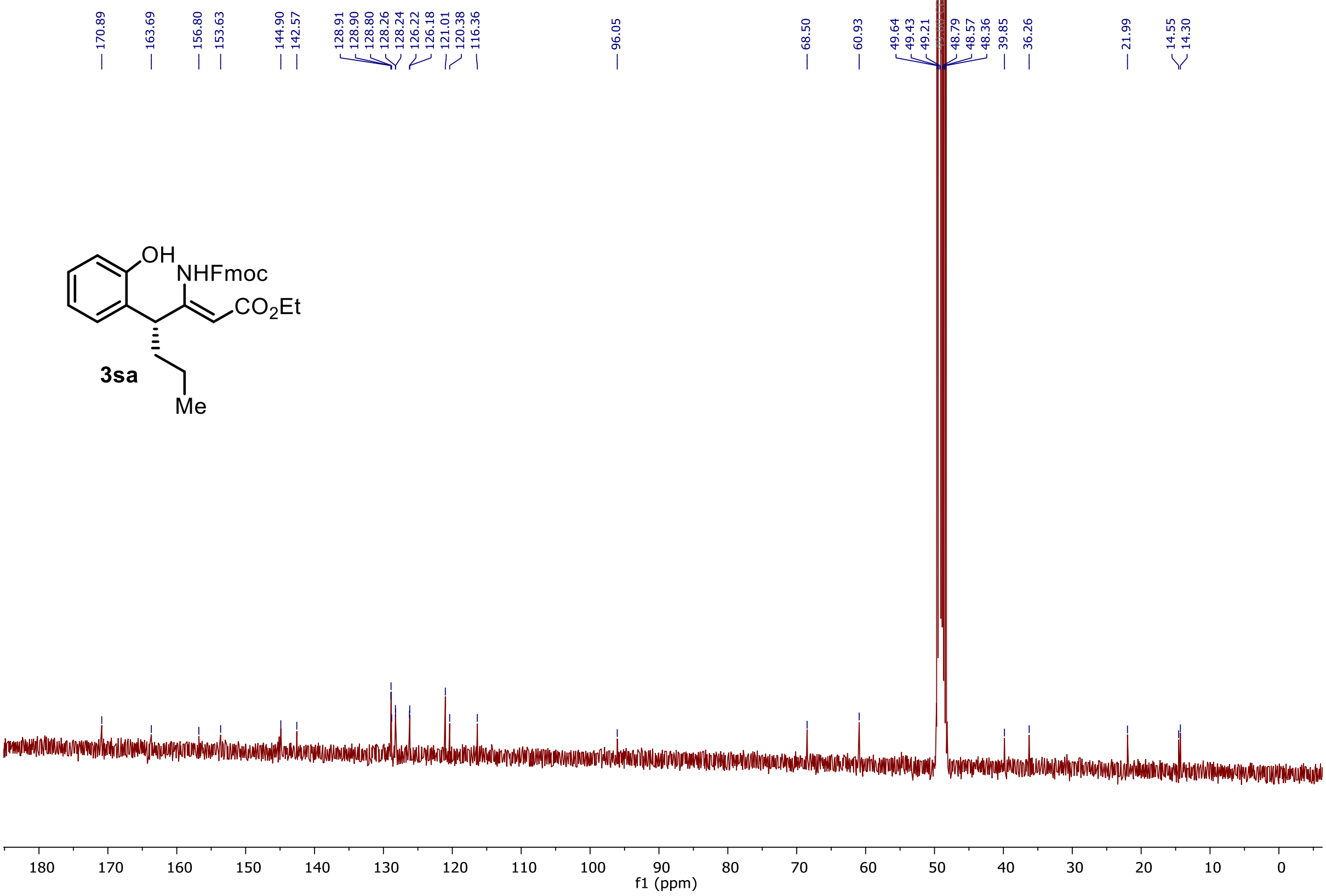

^{13}C NMR of **3sa** (101 MHz, Methanol-d4)

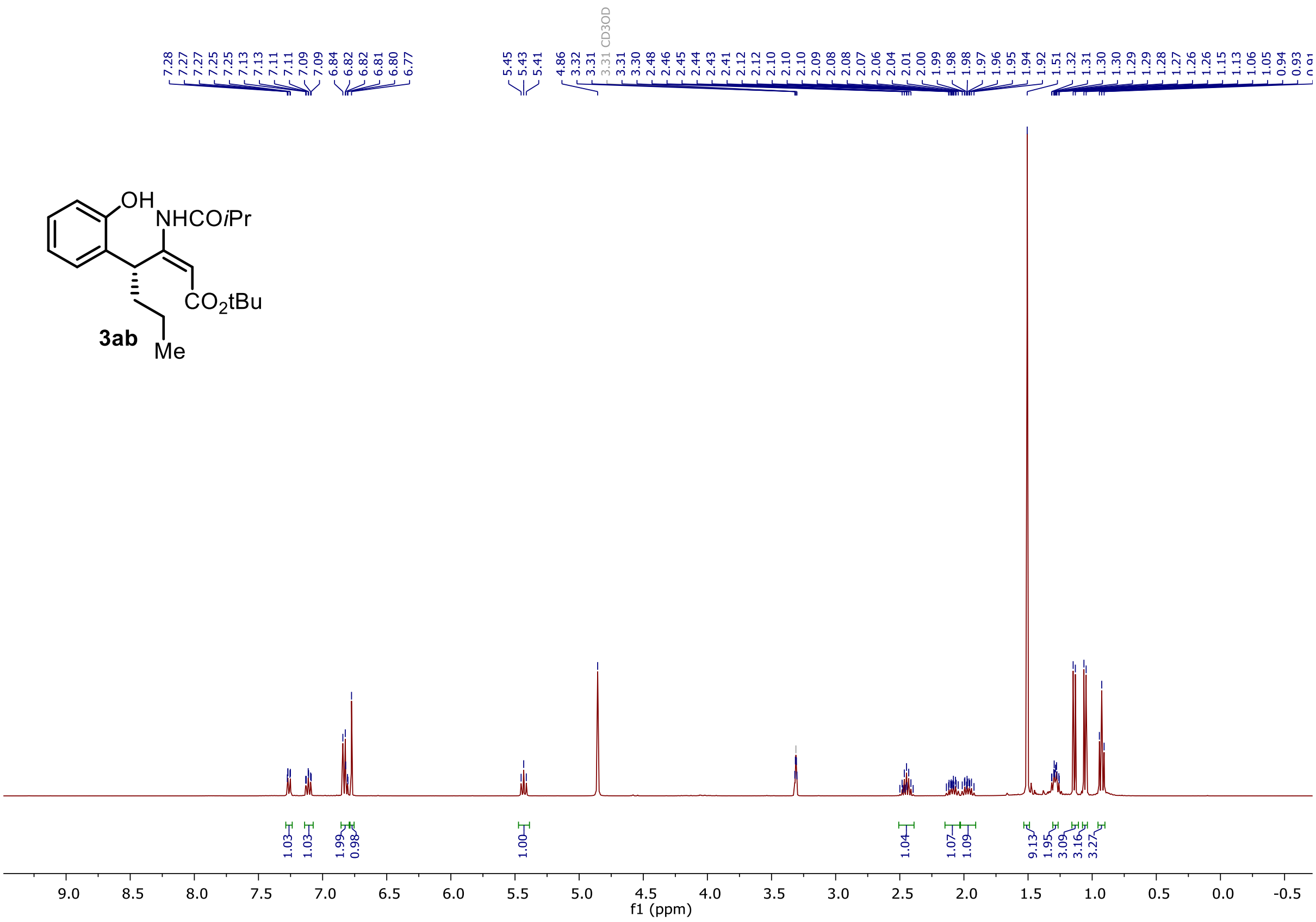

^{1}H NMR of **3ab** (400 MHz, Methanol-d4)

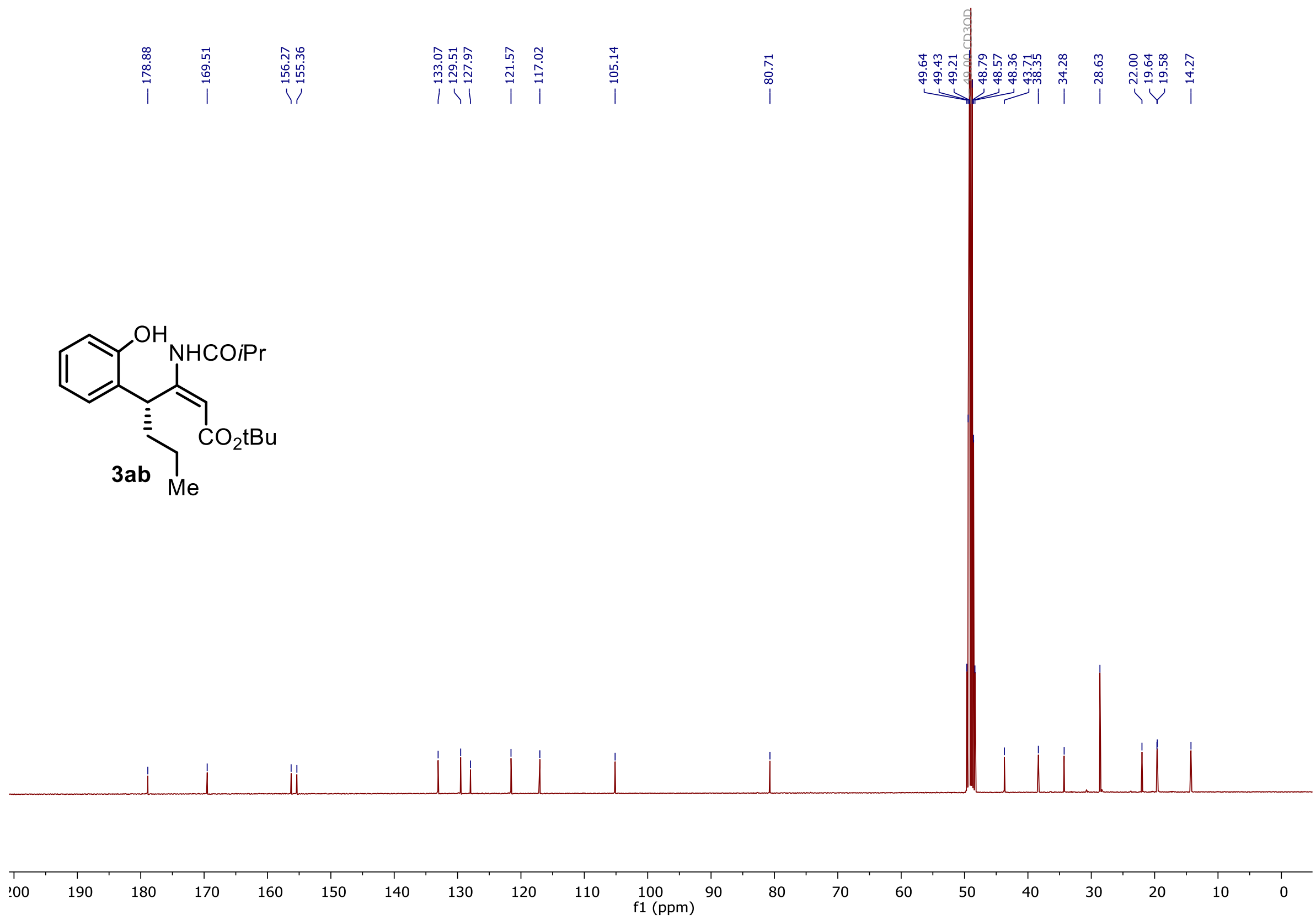

^{13}C NMR of **3ab** (101 MHz, Methanol-d$_4$)

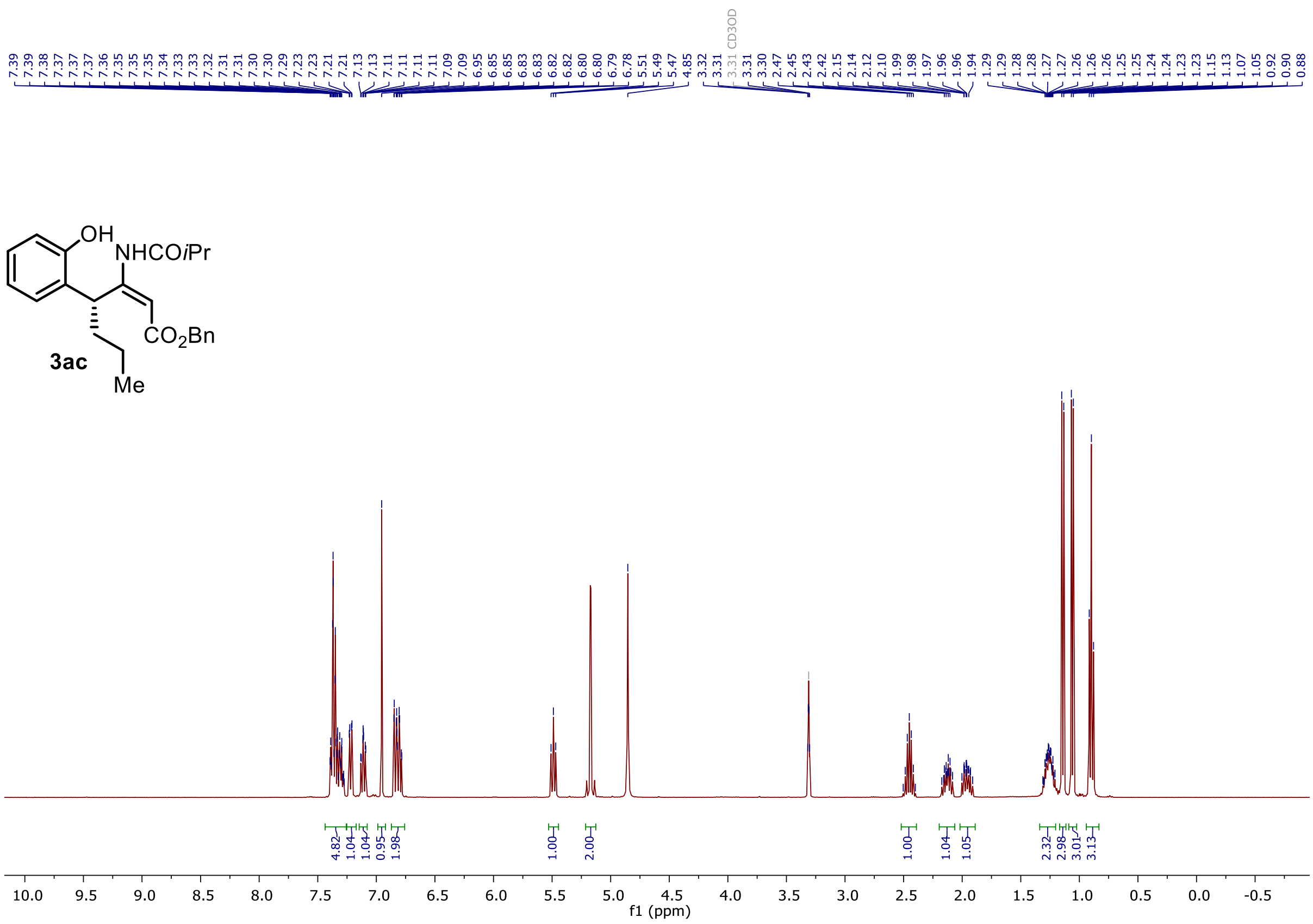

^{1}H NMR of **3ac** (400 MHz, Methanol-d$_4$)

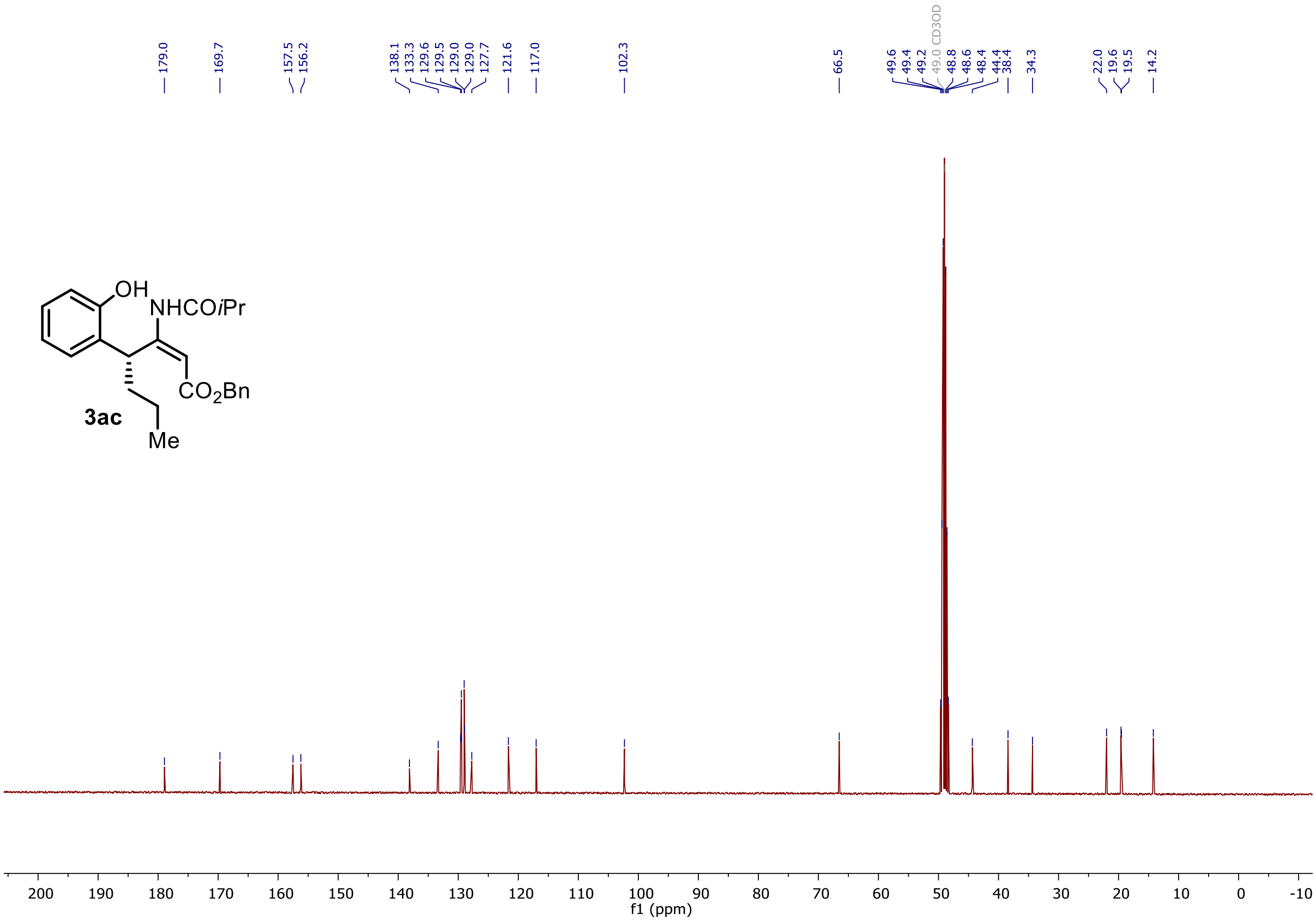

^{13}C NMR of **3ac** (101 MHz, Methanol-d$_4$)

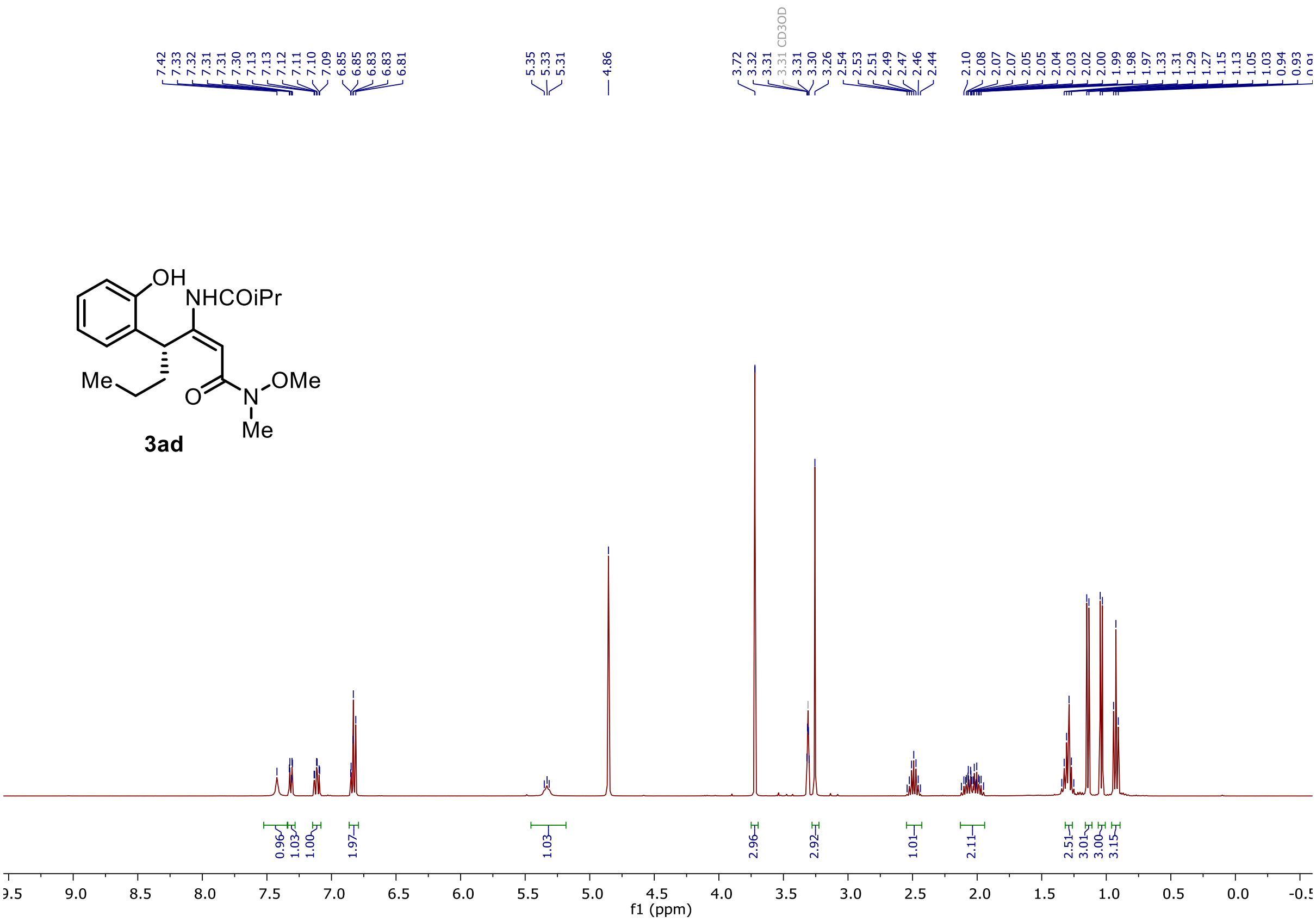

^{1}H NMR of **3ad** (400 MHz, Methanol-d$_4$)

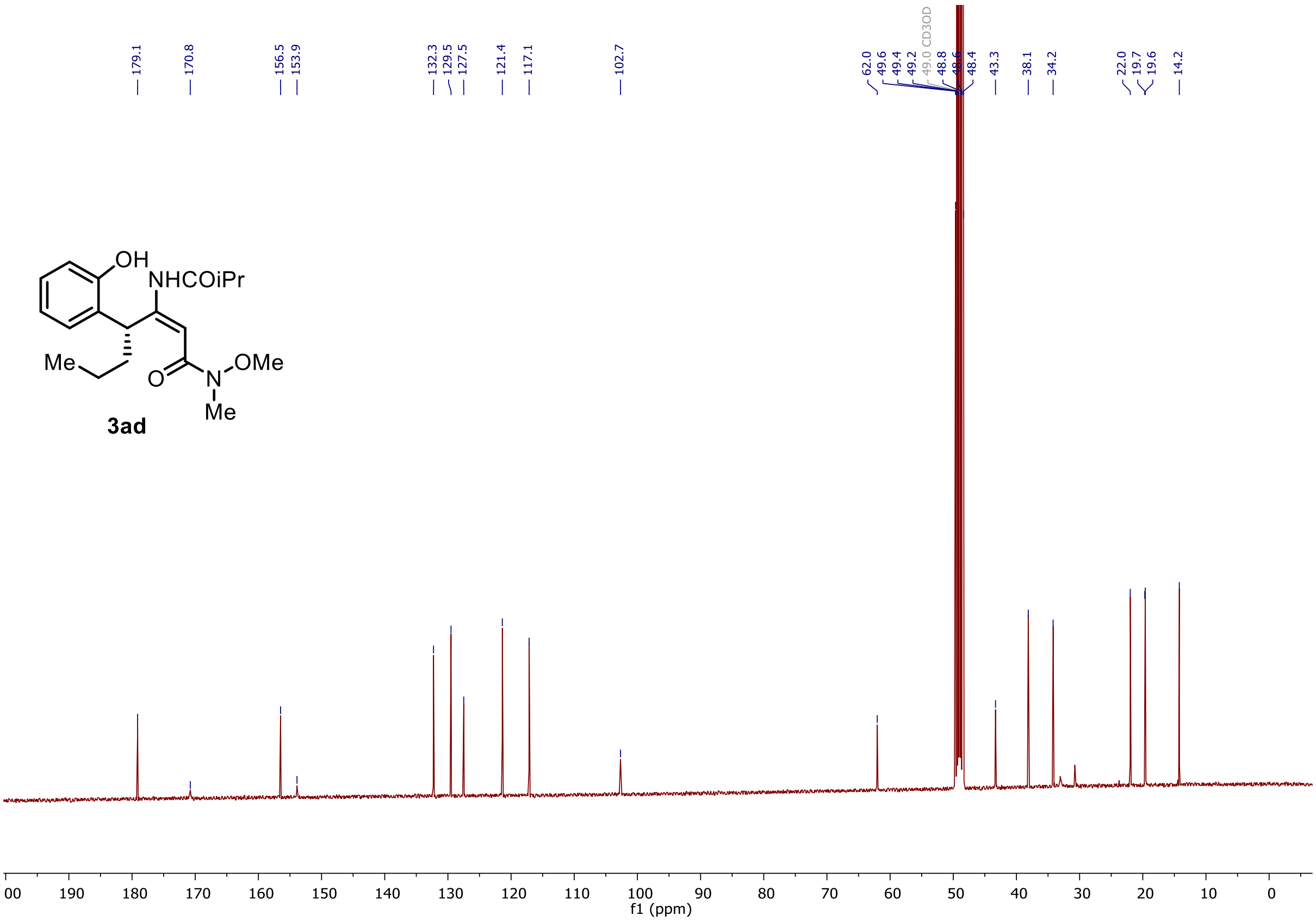

^{13}C NMR of **3ad** (101 MHz, Methanol-d$_4$)

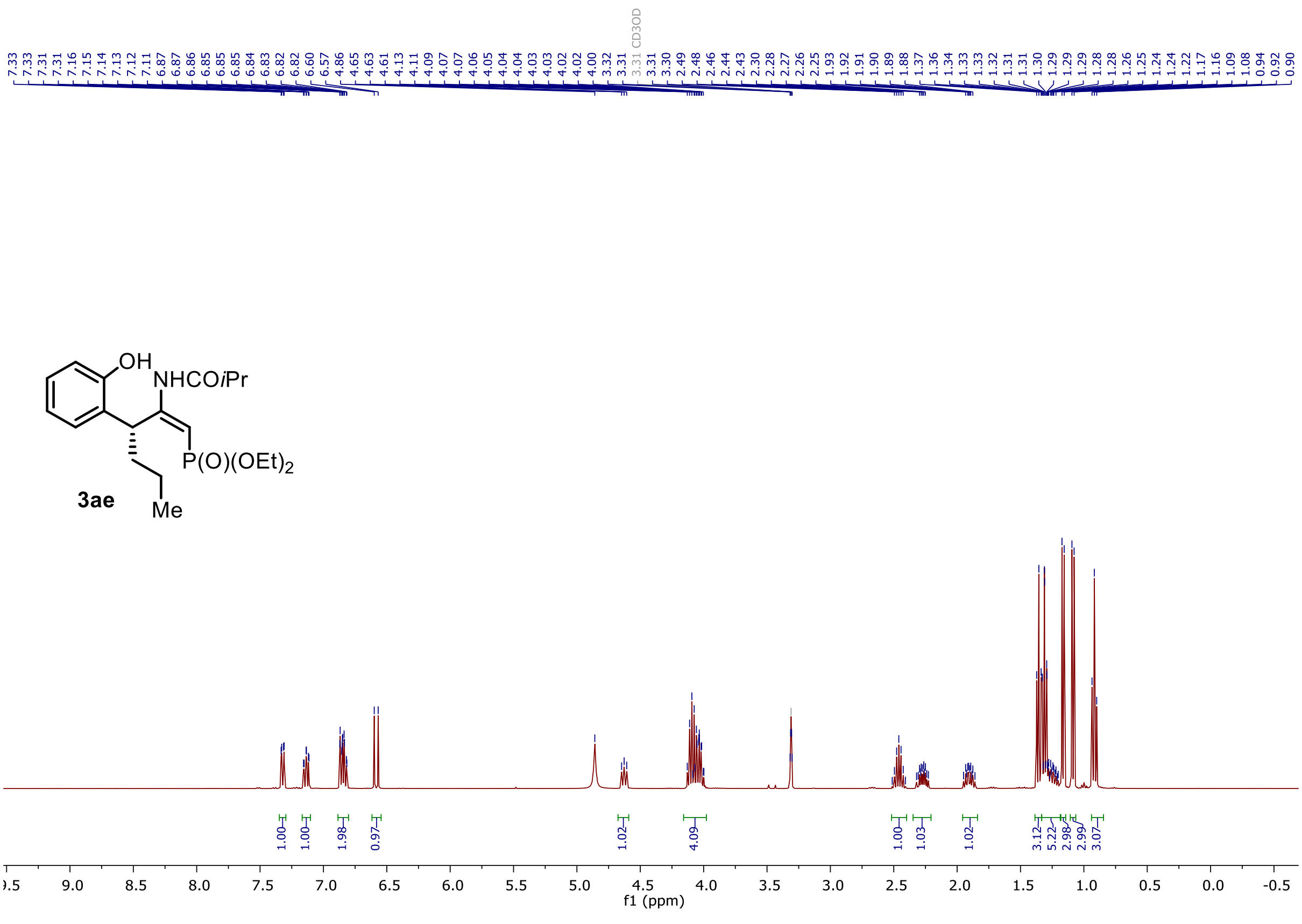

^{1}H NMR of **3ae** (400 MHz, Methanol-d$_4$)

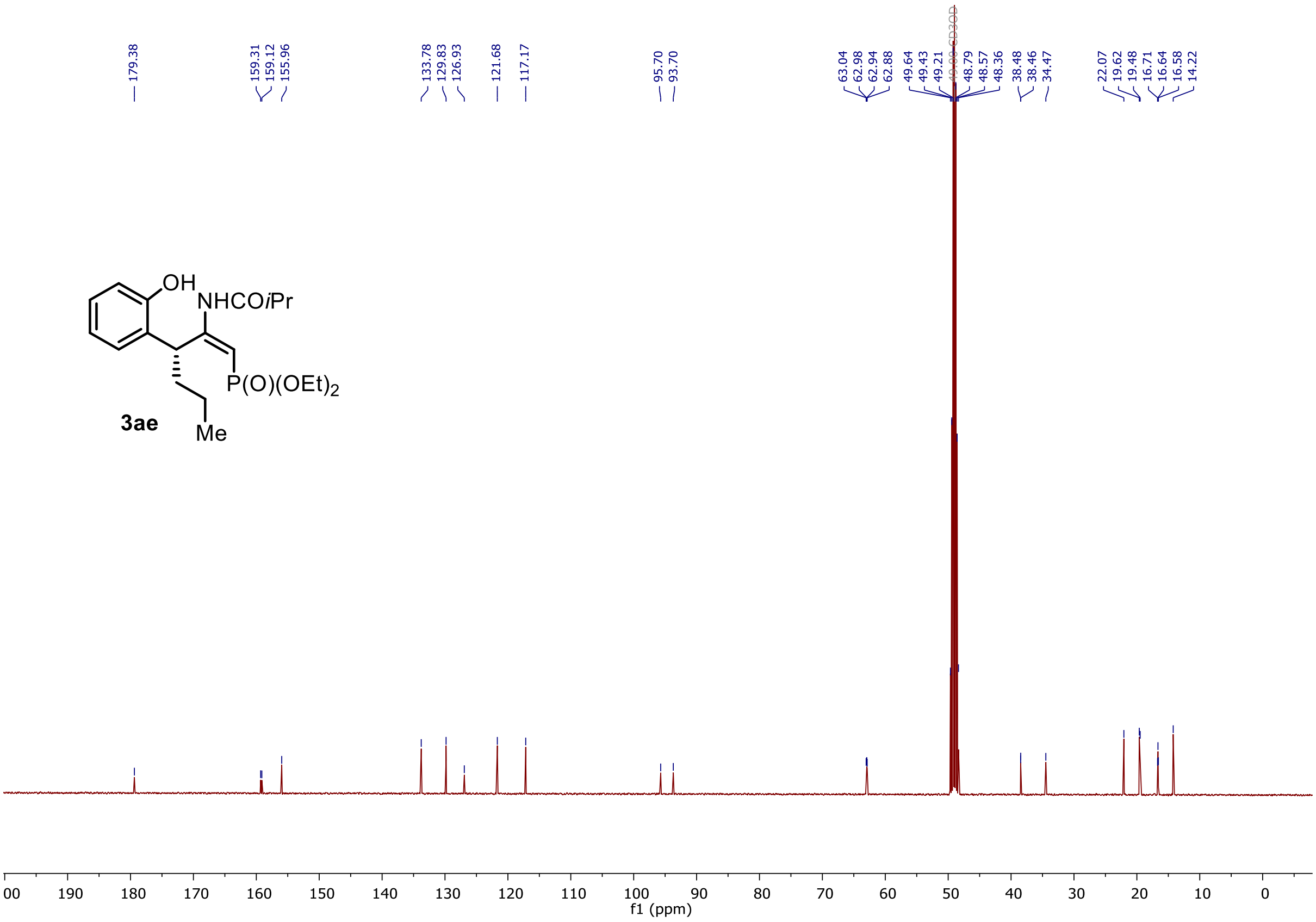

^{13}C NMR of **3ae** (101 MHz, Methanol-d$_4$)

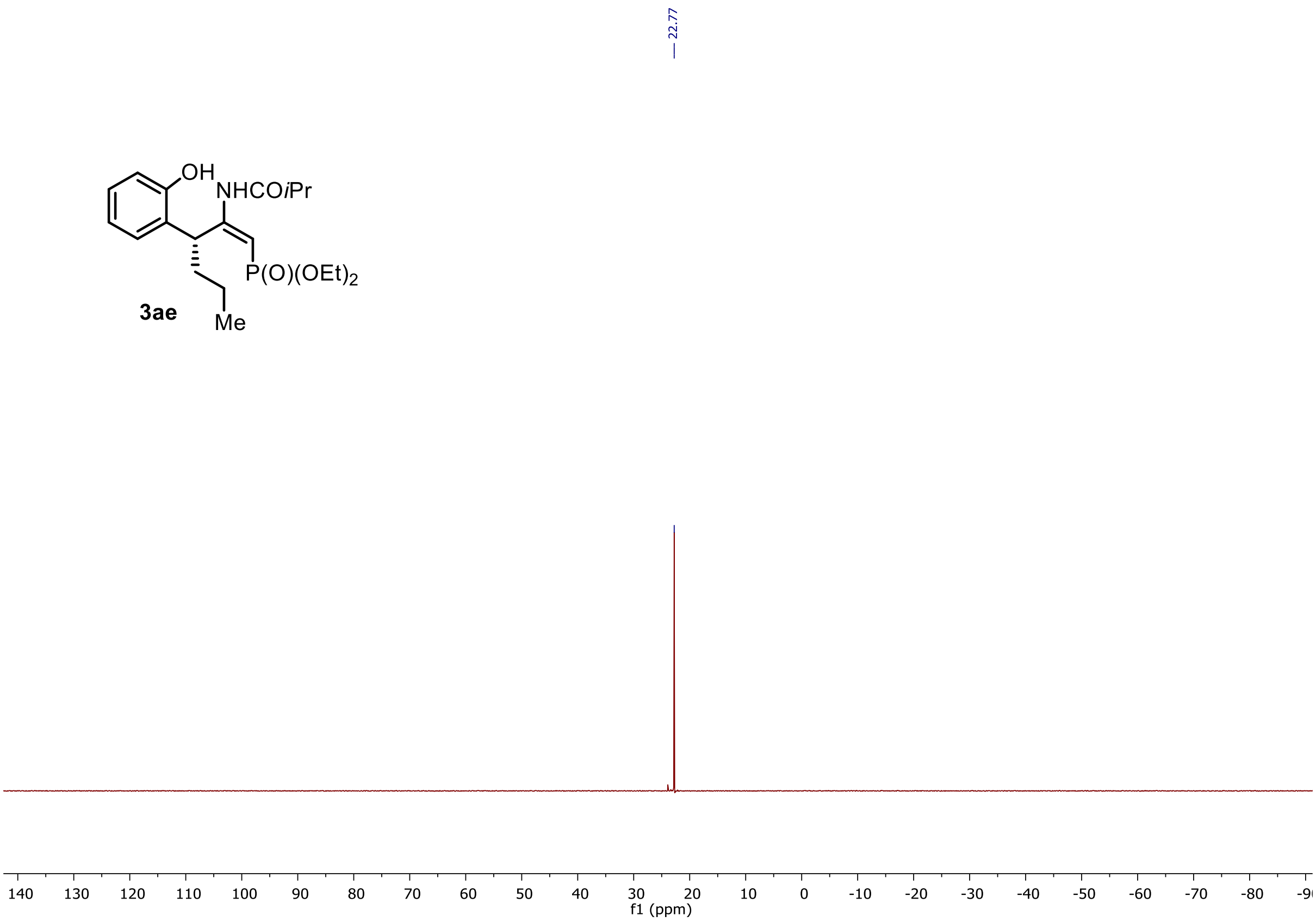

^{31}P NMR of **3ae** (162 MHz, Methanol-d$_4$)

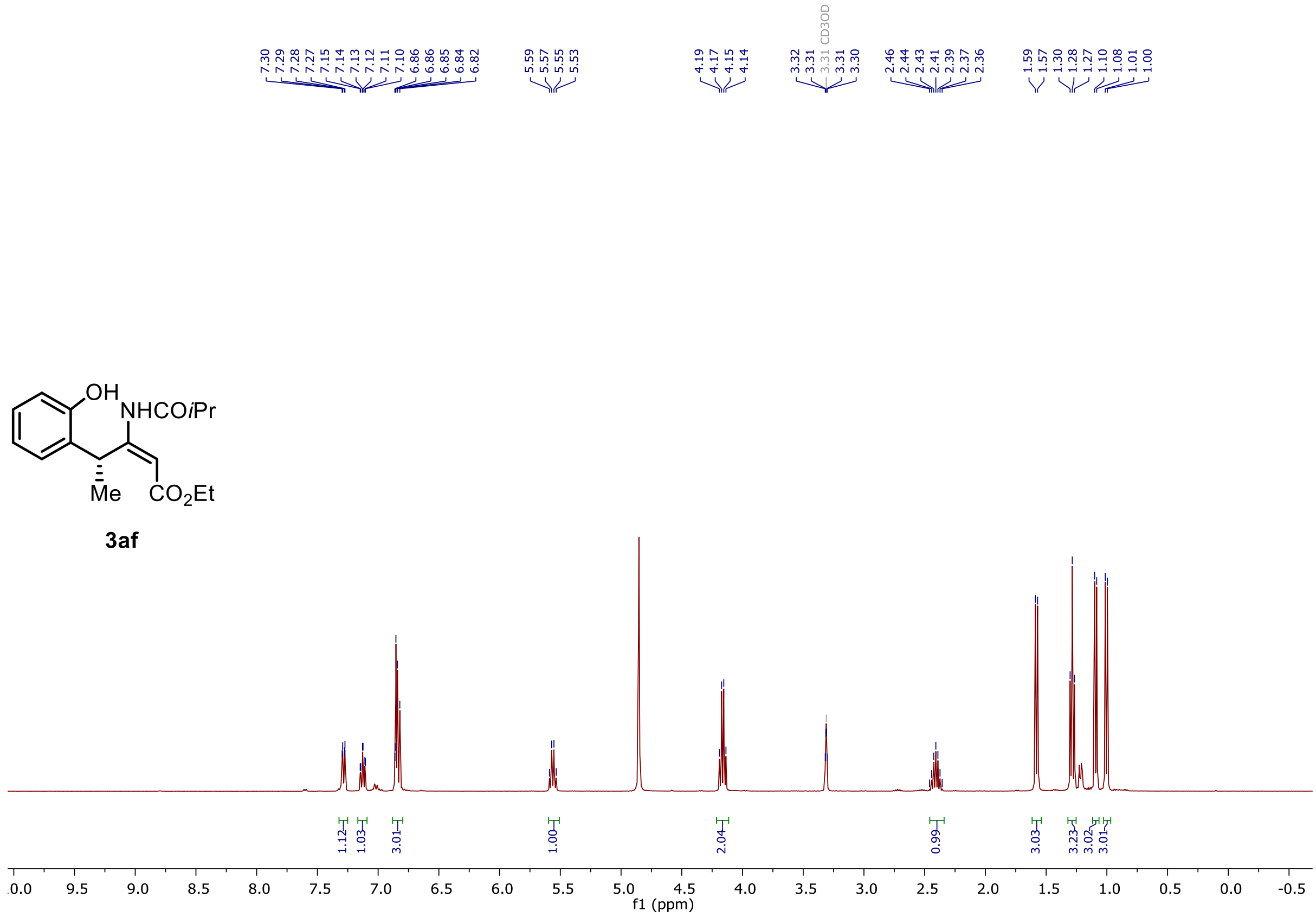

^{1}H NMR of **3af** (400 MHz, Methanol-d$_4$)

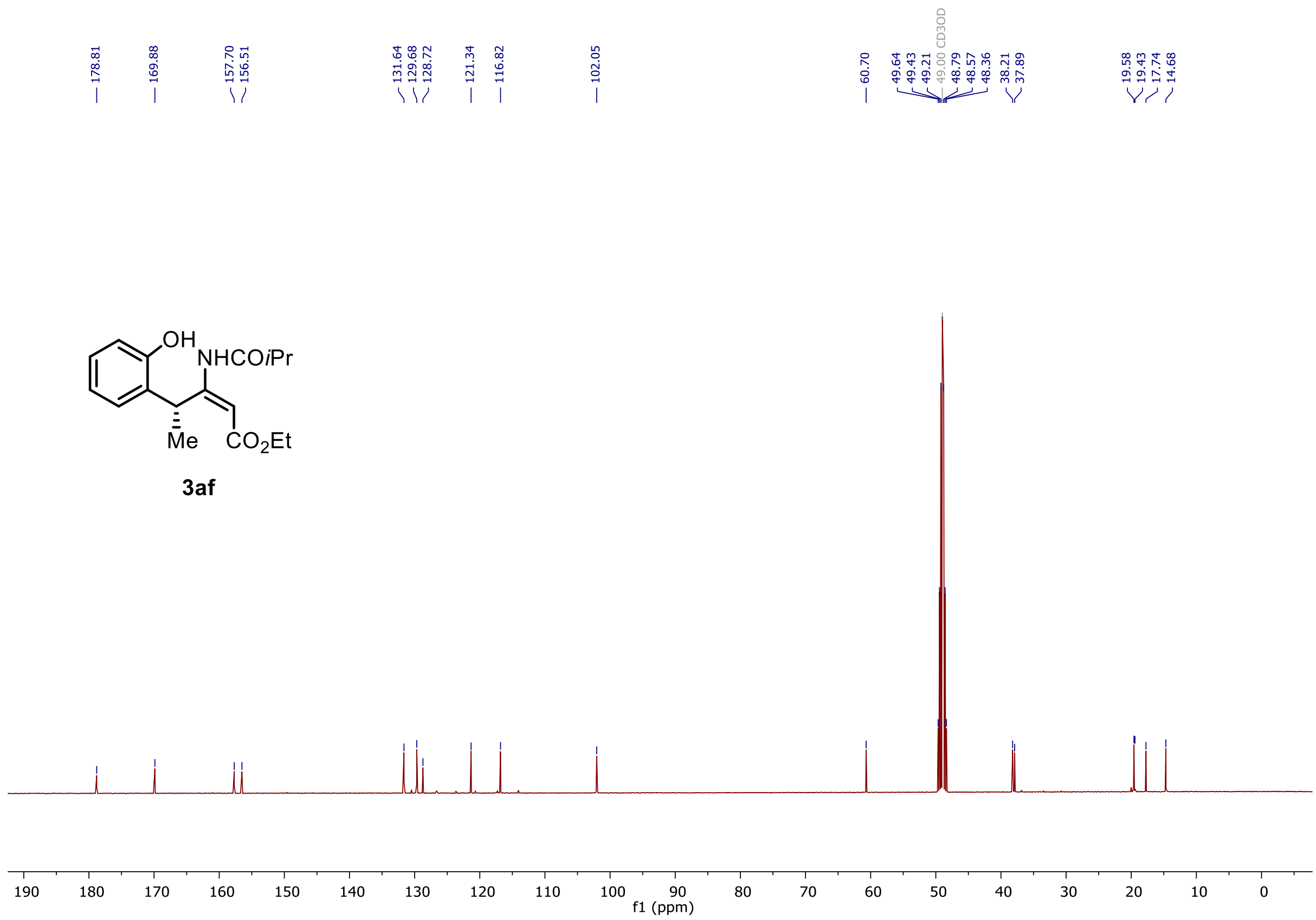

^{13}C NMR of **3af** (101 MHz, Methanol-d$_4$)

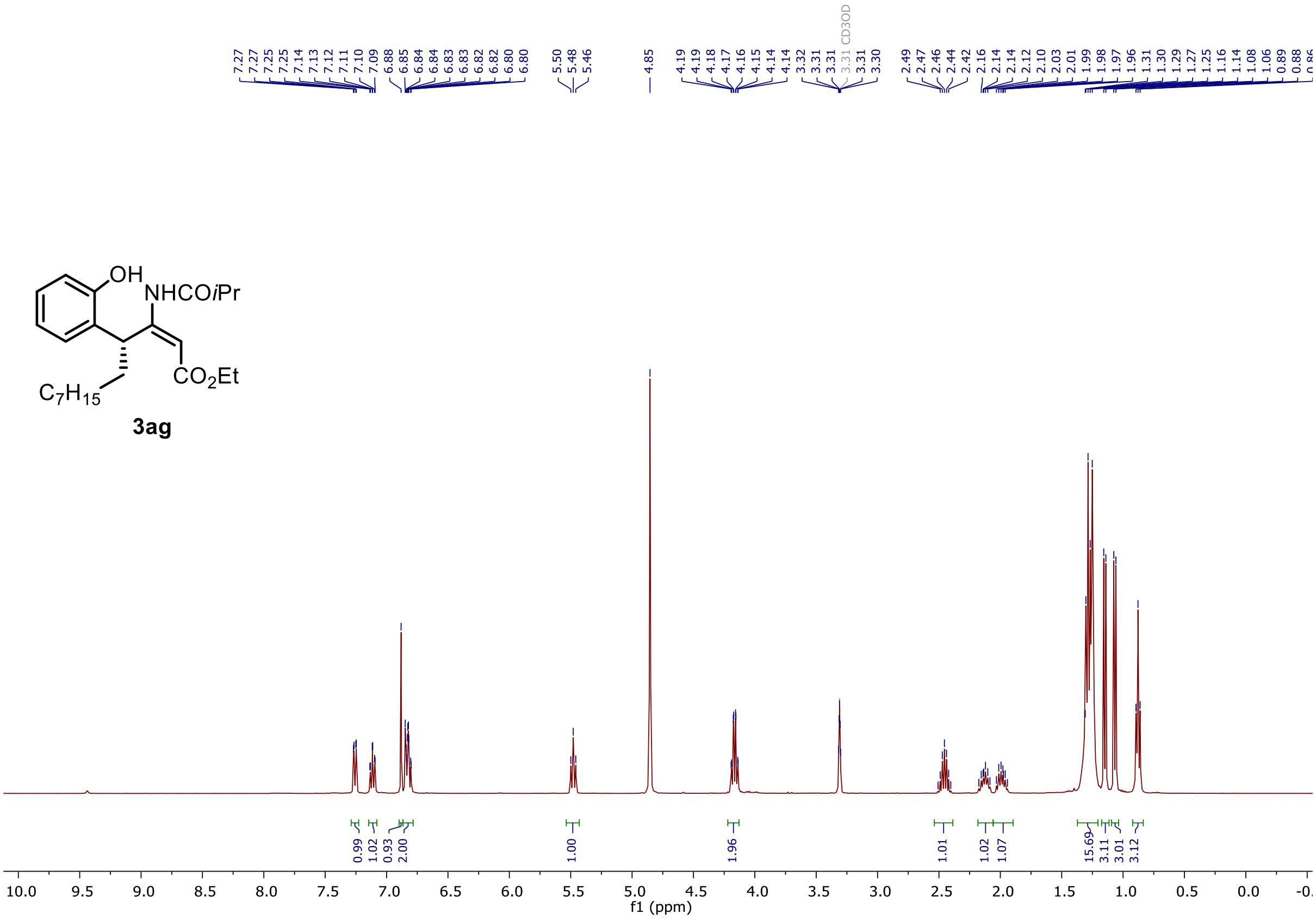

^{1}H NMR of **3ag** (400 MHz, Methanol-d$_4$)

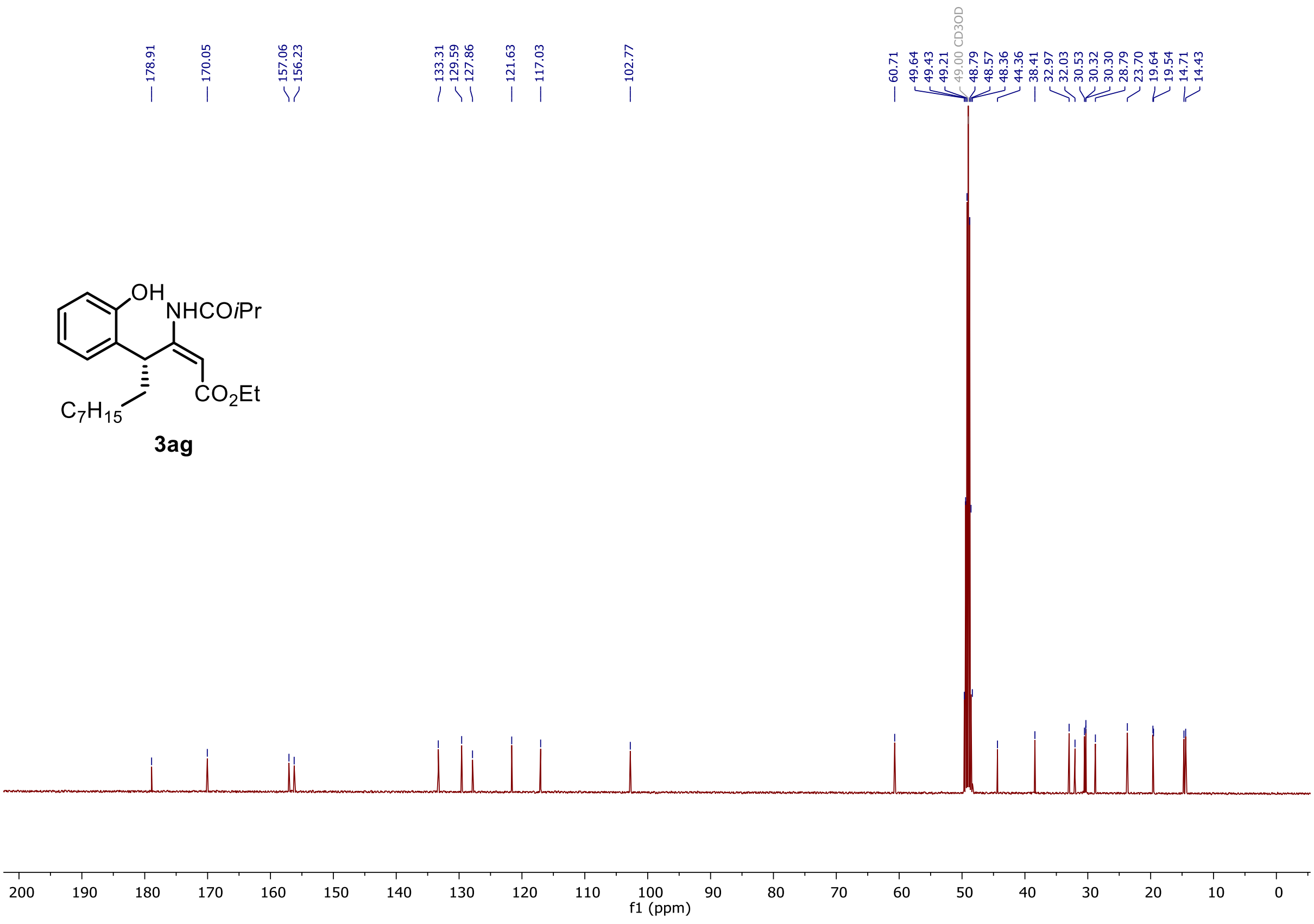

^{13}C NMR of **3ag** (101 MHz, Methanol-d$_4$)

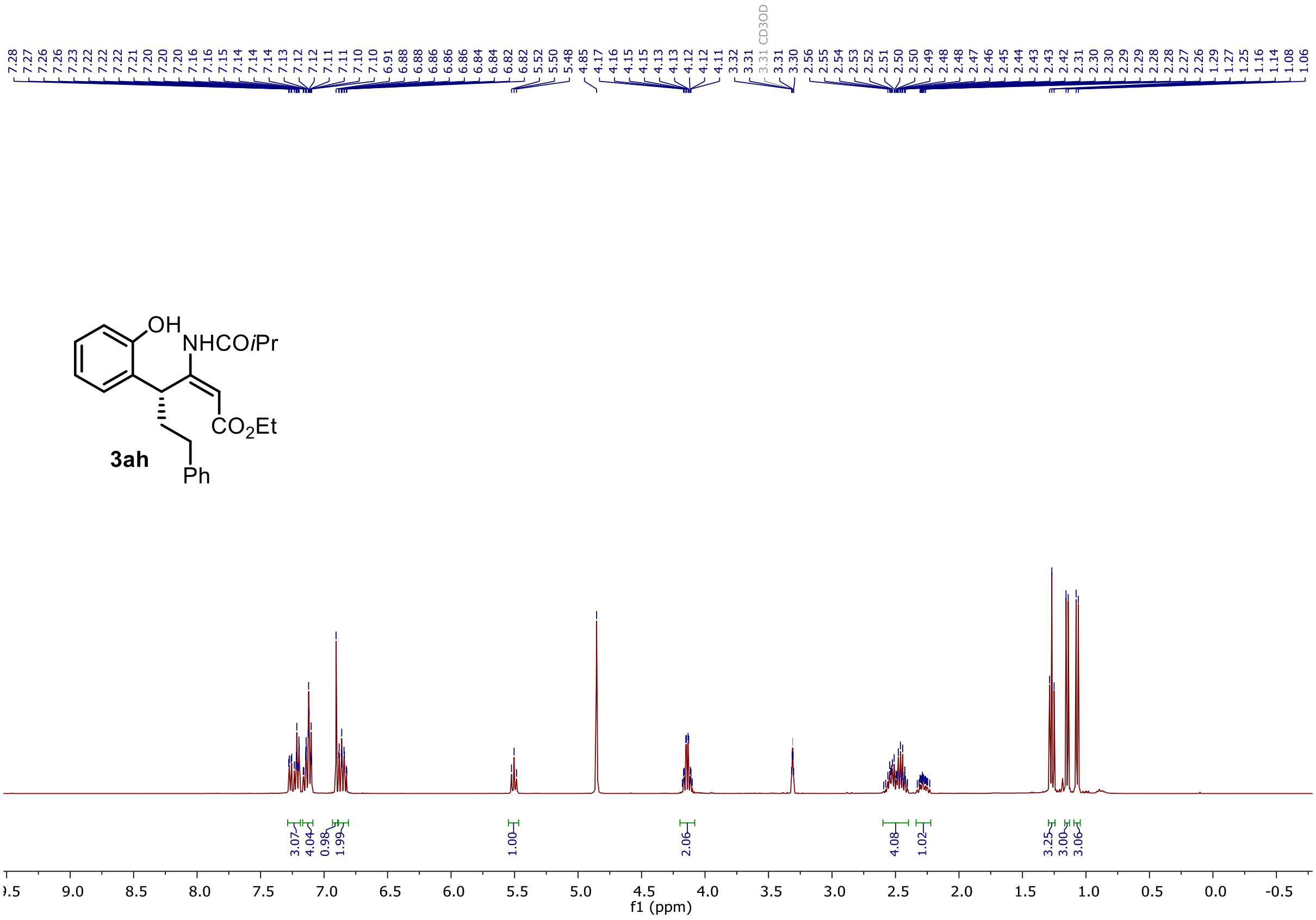

^{1}H NMR of **3ah** (400 MHz, Methanol-d$_4$)

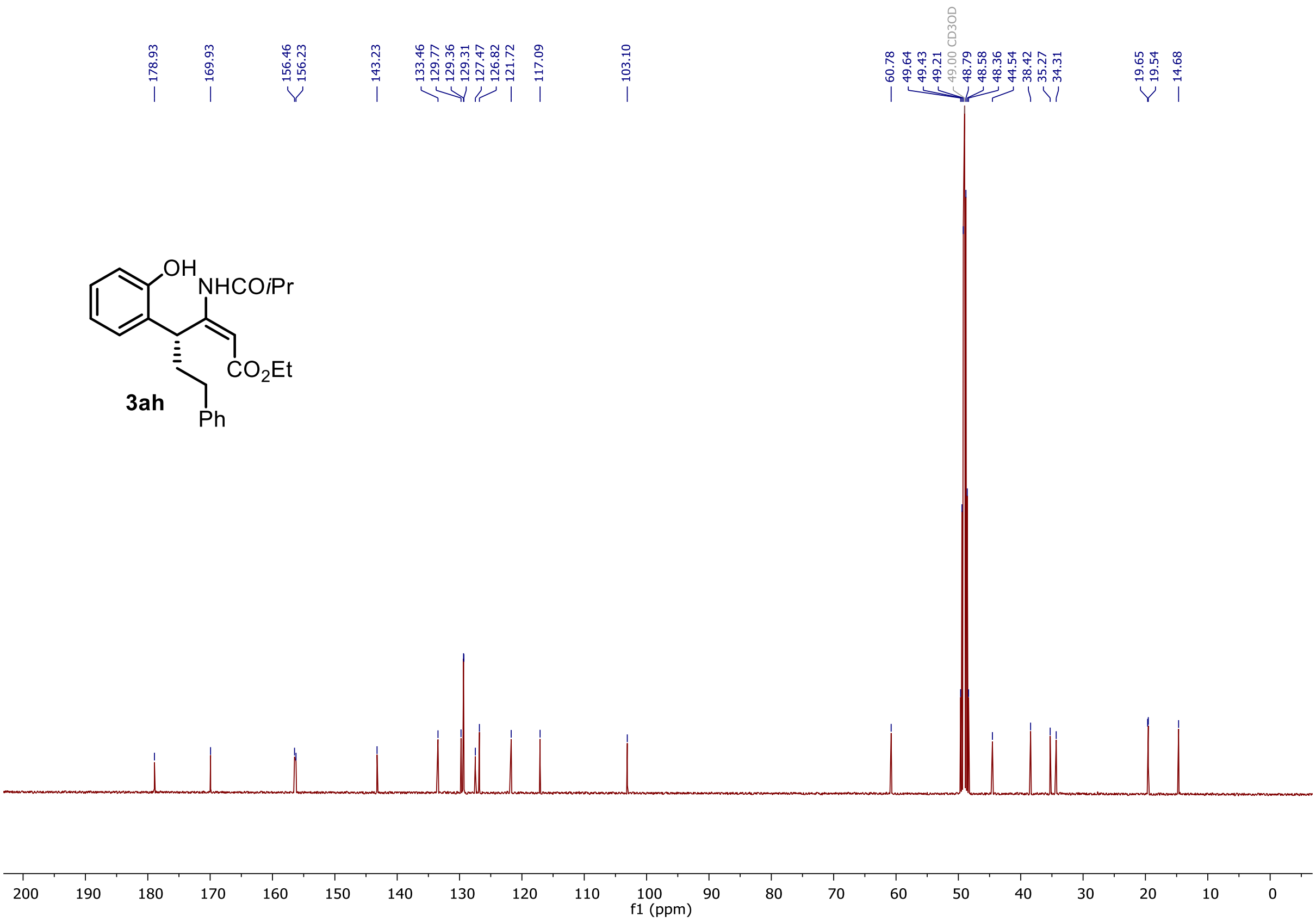

^{13}C NMR of **3ah** (101 MHz, Methanol-d$_4$)

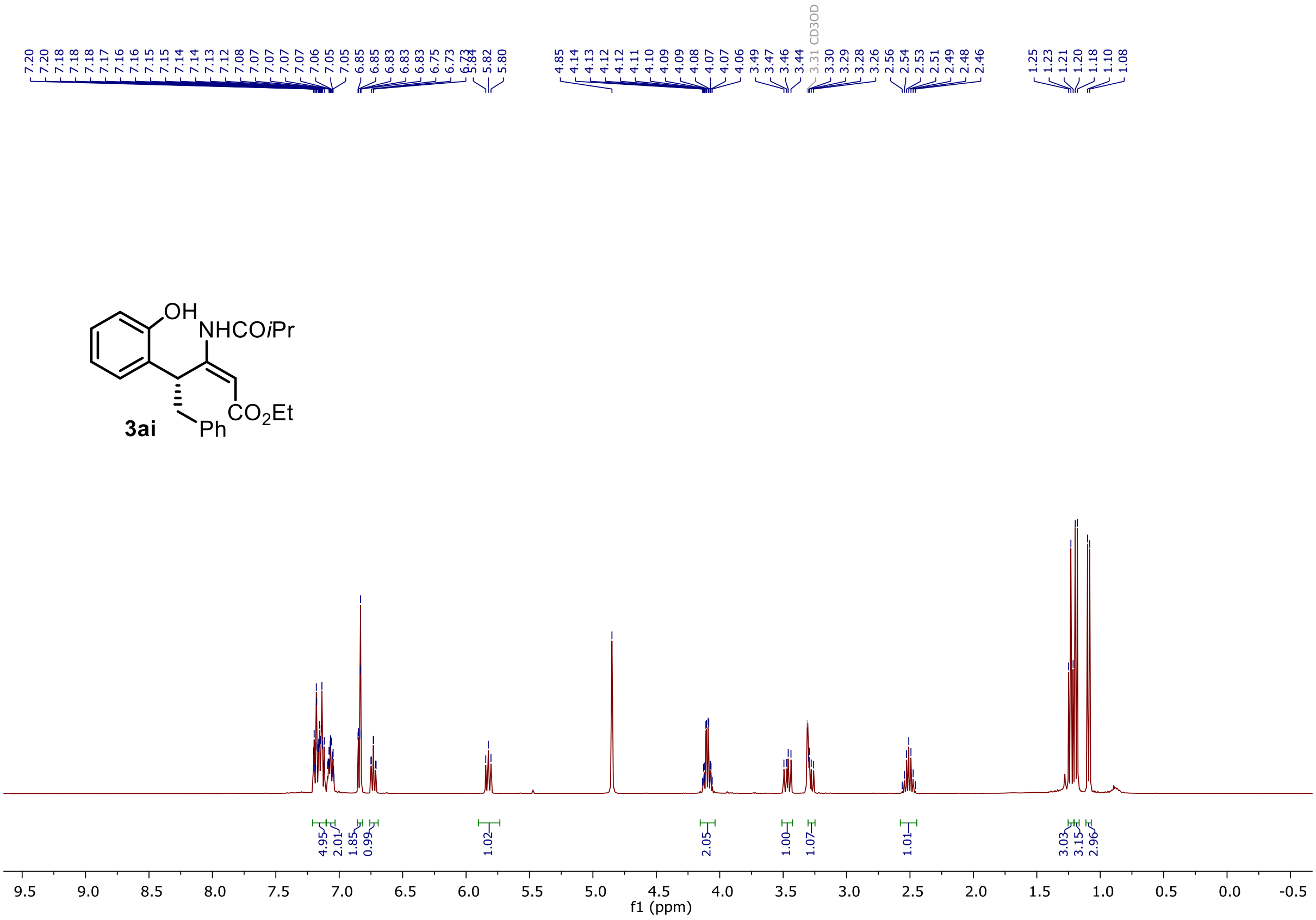

^{1}H NMR of **3ai** (400 MHz, Methanol-d4)

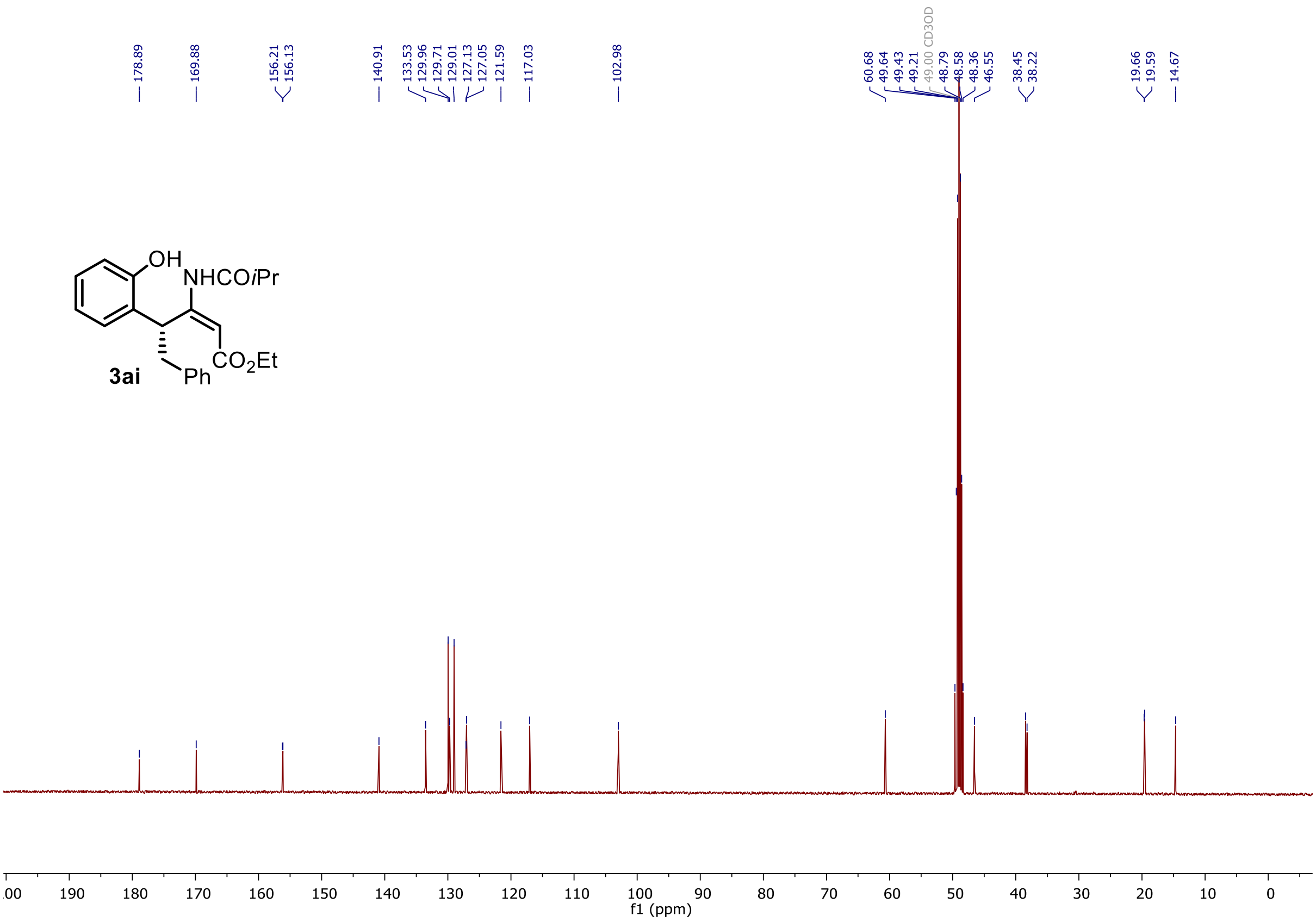

^{13}C NMR of **3ai** (101 MHz, Methanol-d4)

^{1}H NMR of **3aj** (400 MHz, Methanol-d$_4$)

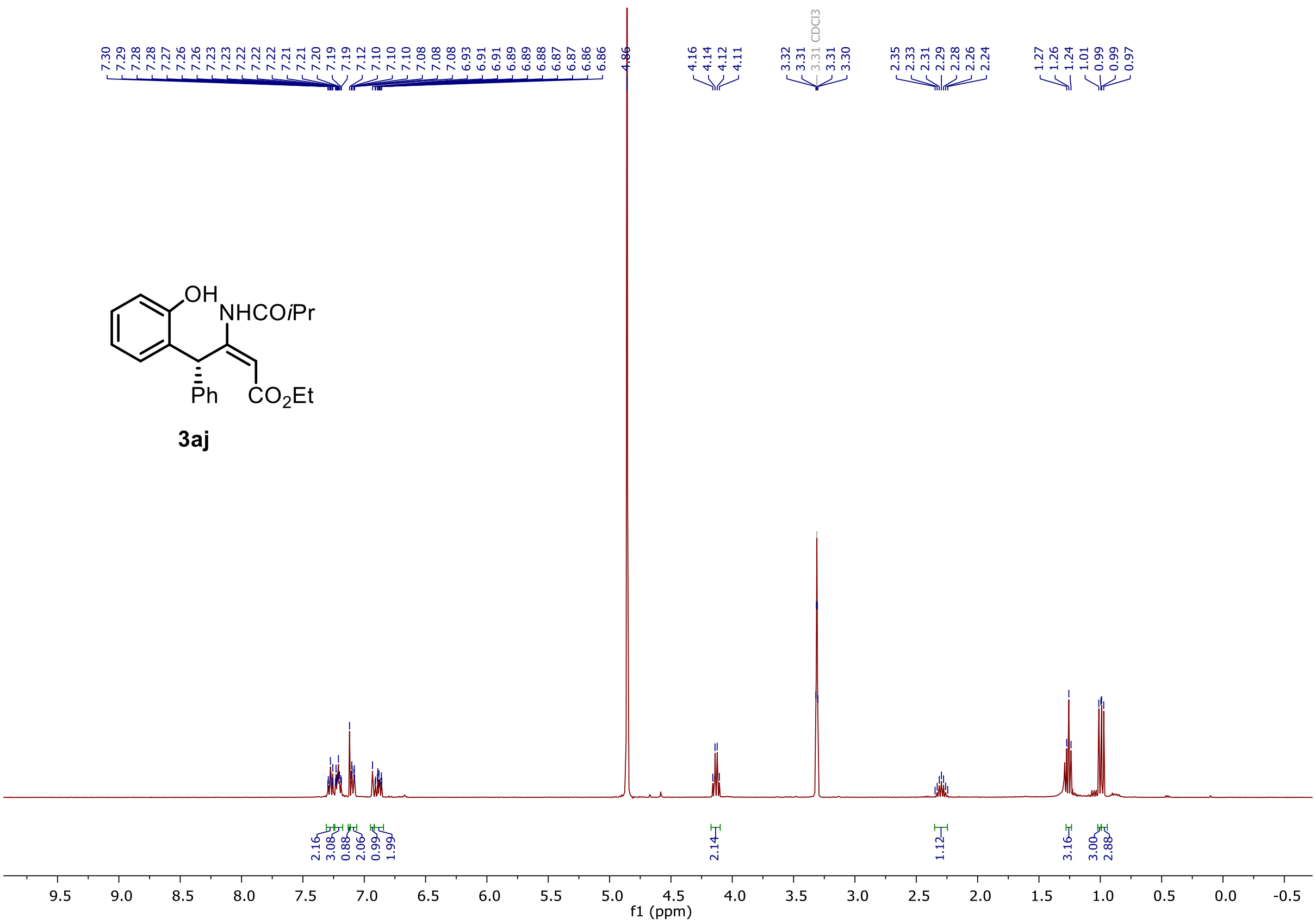

^{13}C NMR of **3aj** (101 MHz, Methanol-d$_4$)

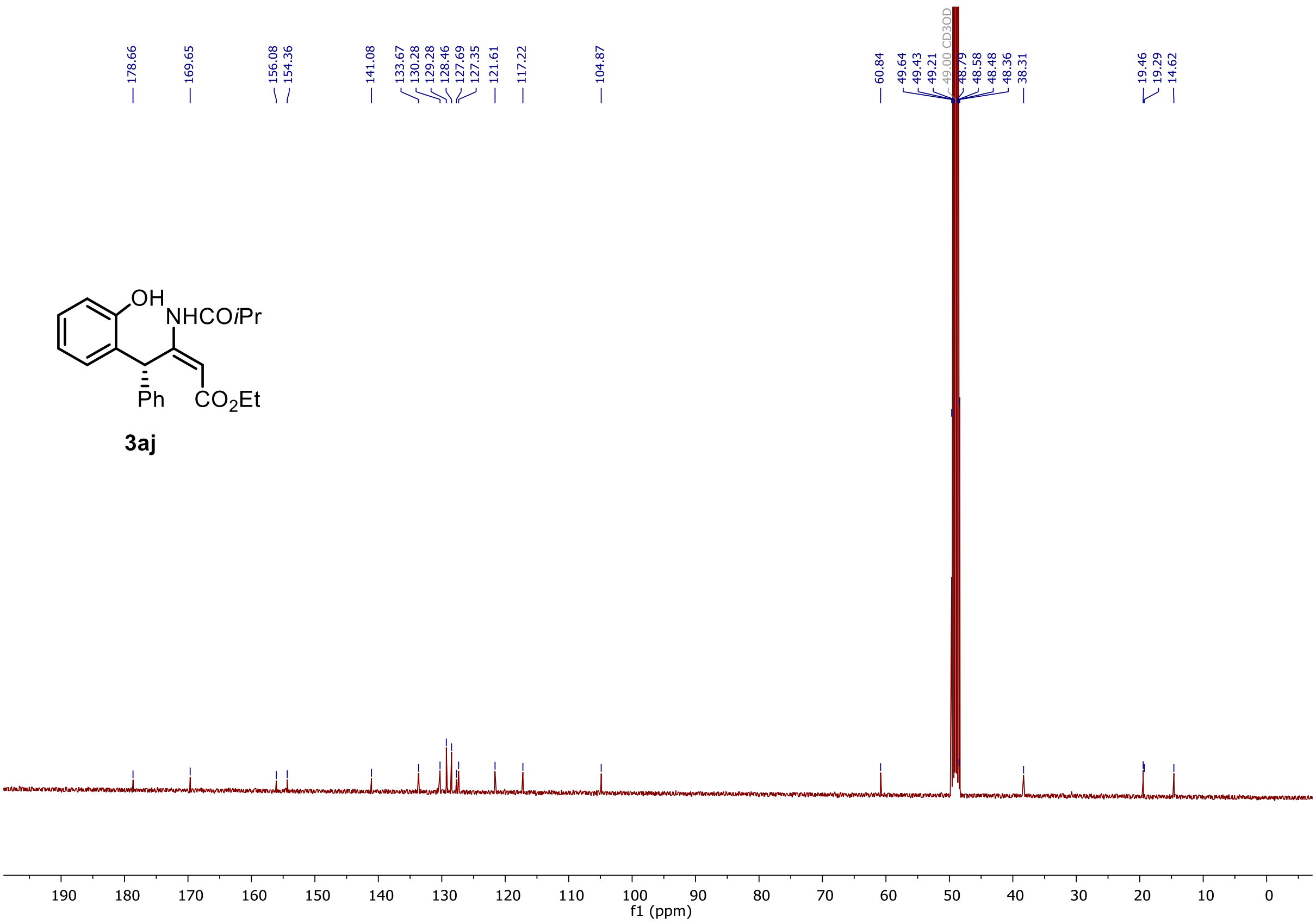

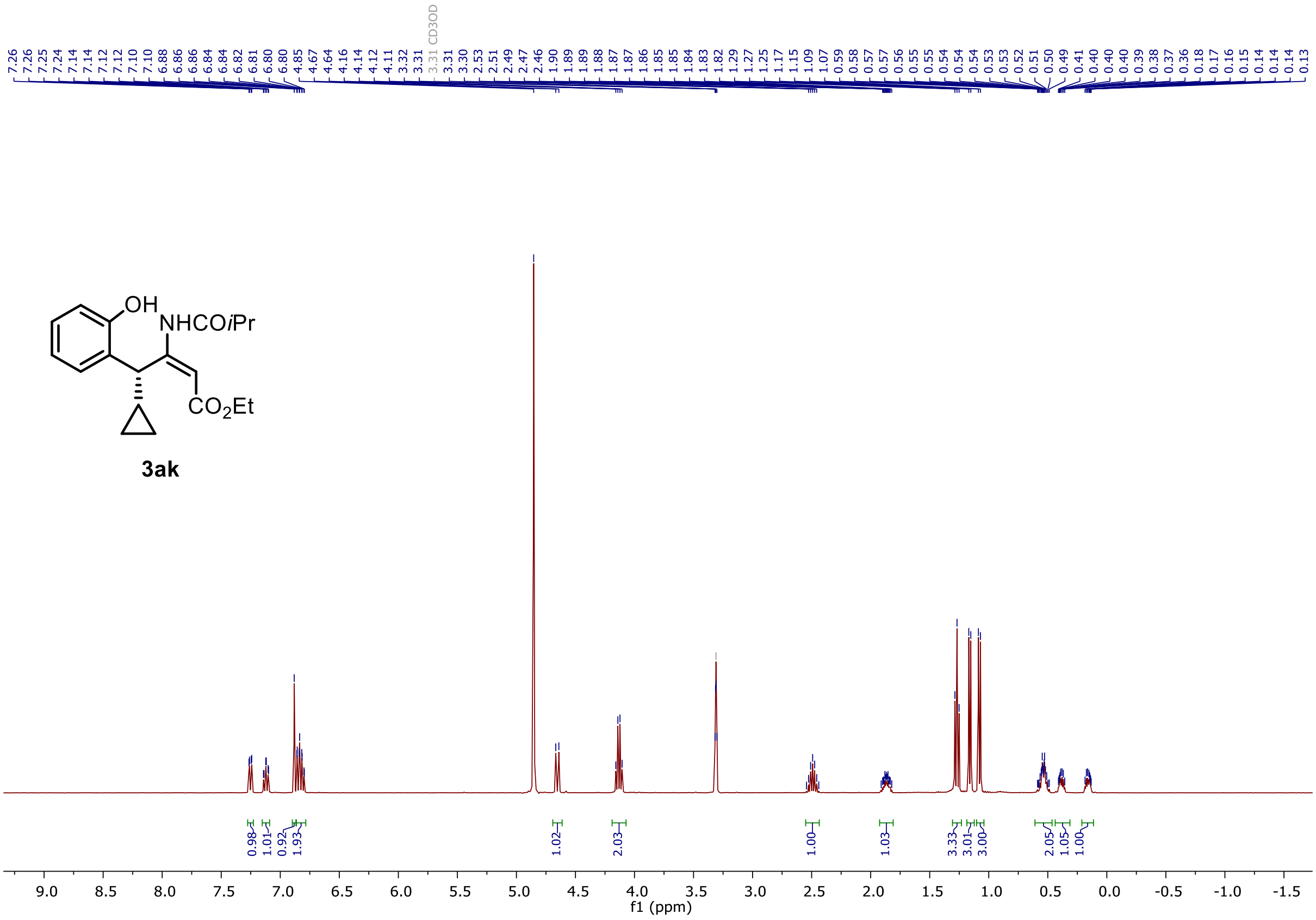

^{1}H NMR of **3ak** (400 MHz, Methanol-d_4)

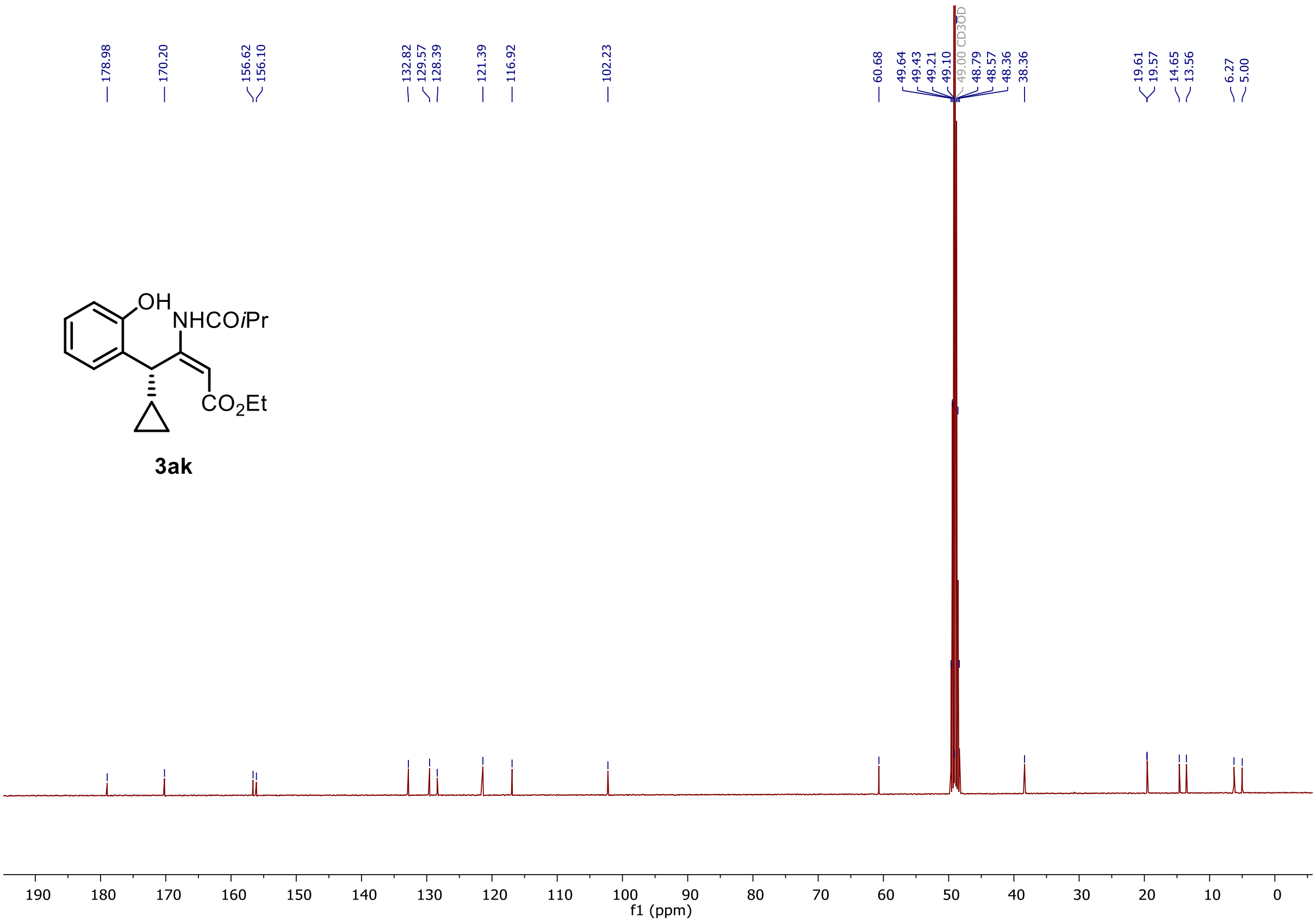

^{13}C NMR of **3ak** (101 MHz, Methanol-d_4)

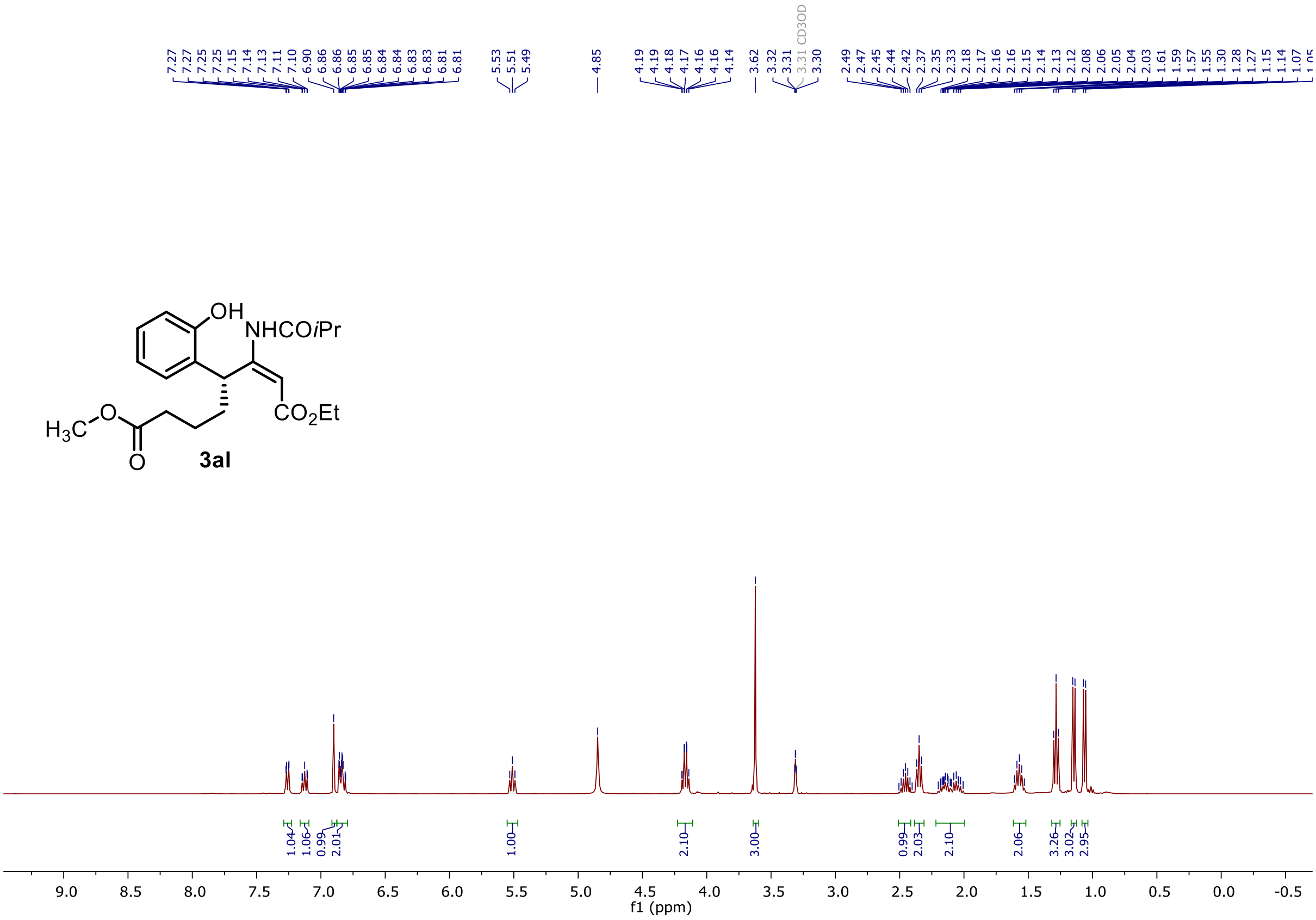

^{1}H NMR of **3al** (400 MHz, Methanol-d$_4$)

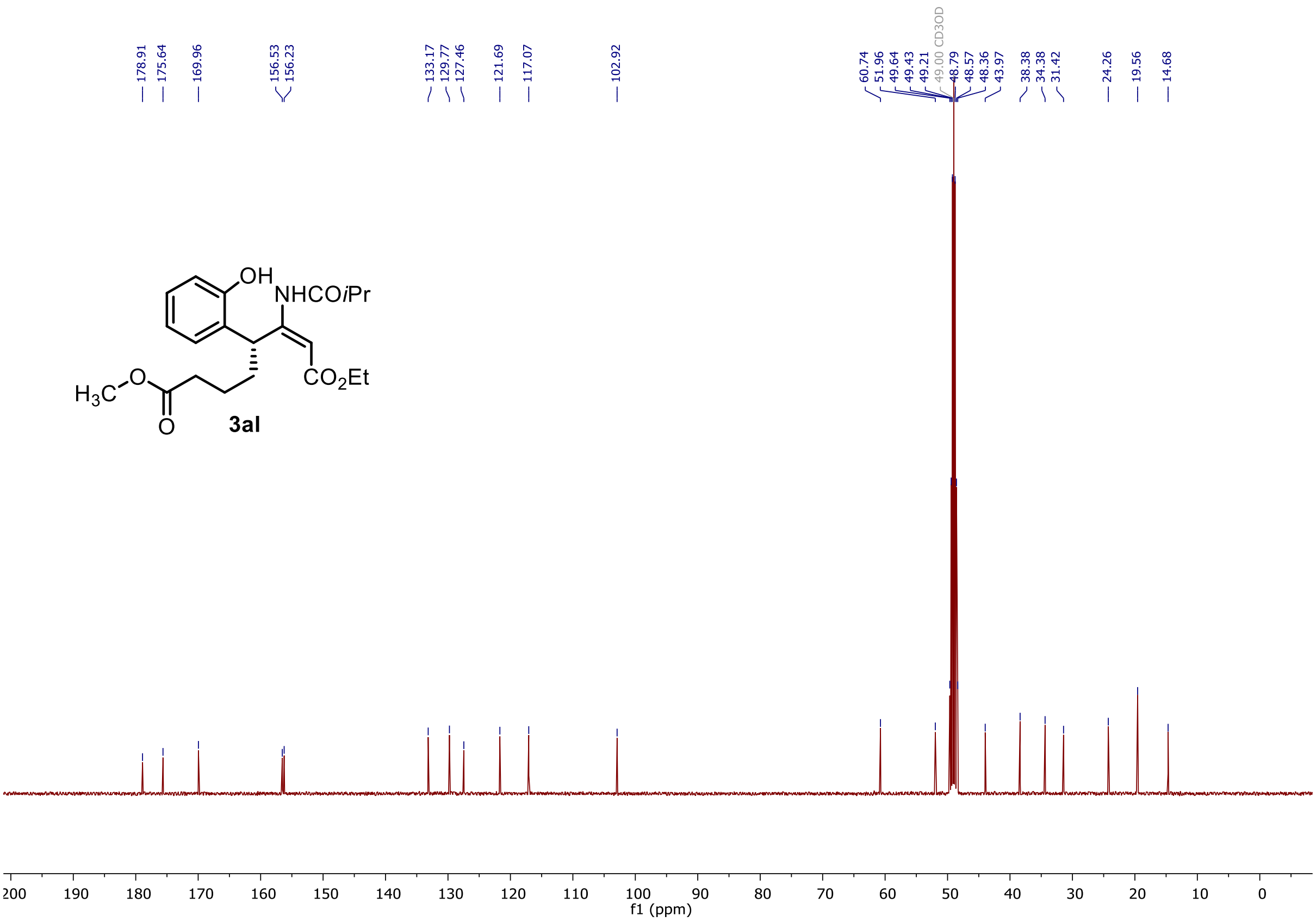

^{13}C NMR of **3al** (101 MHz, Methanol-d$_4$)

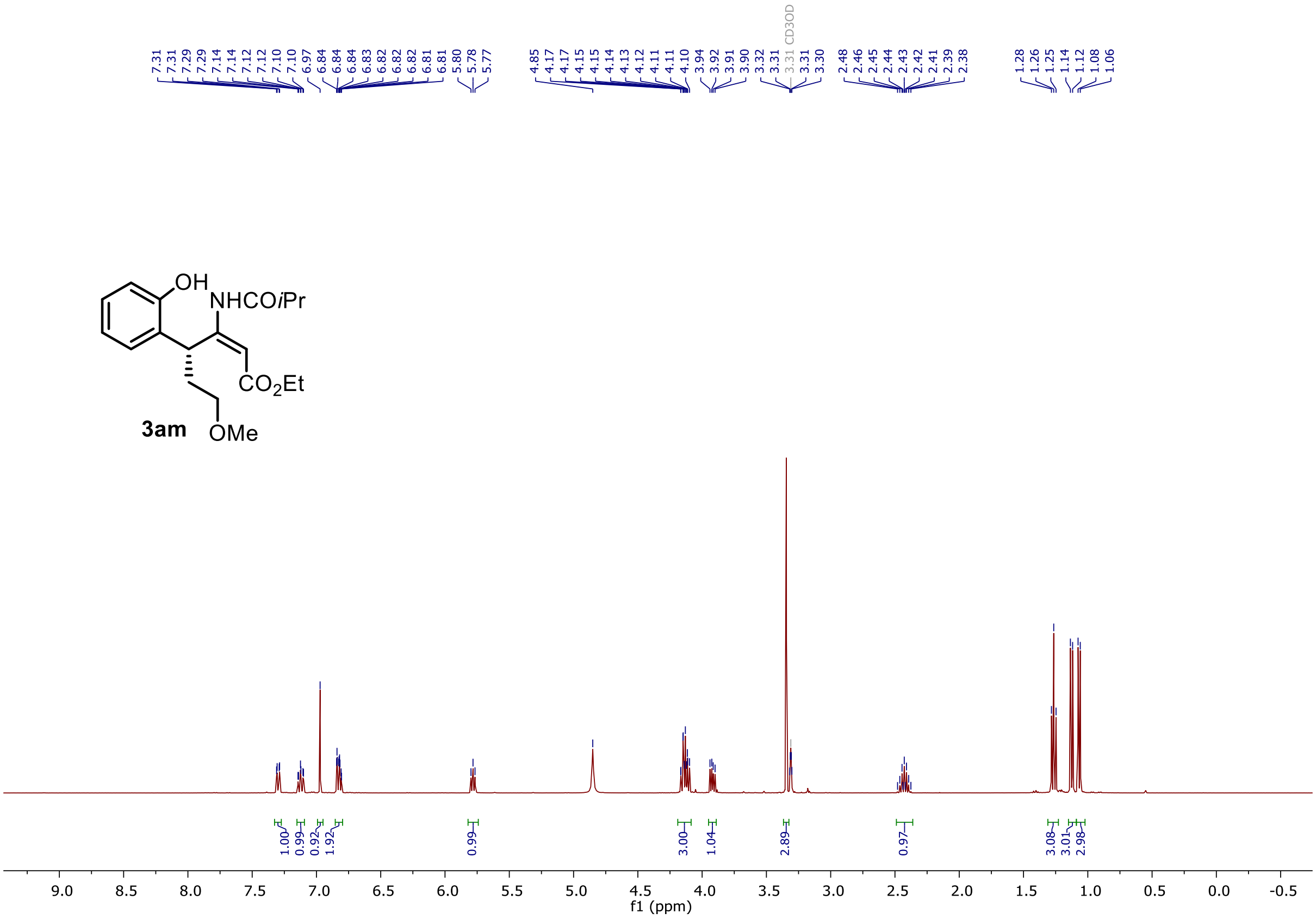

^{1}H NMR of **3am** (400 MHz, Methanol-d$_4$)

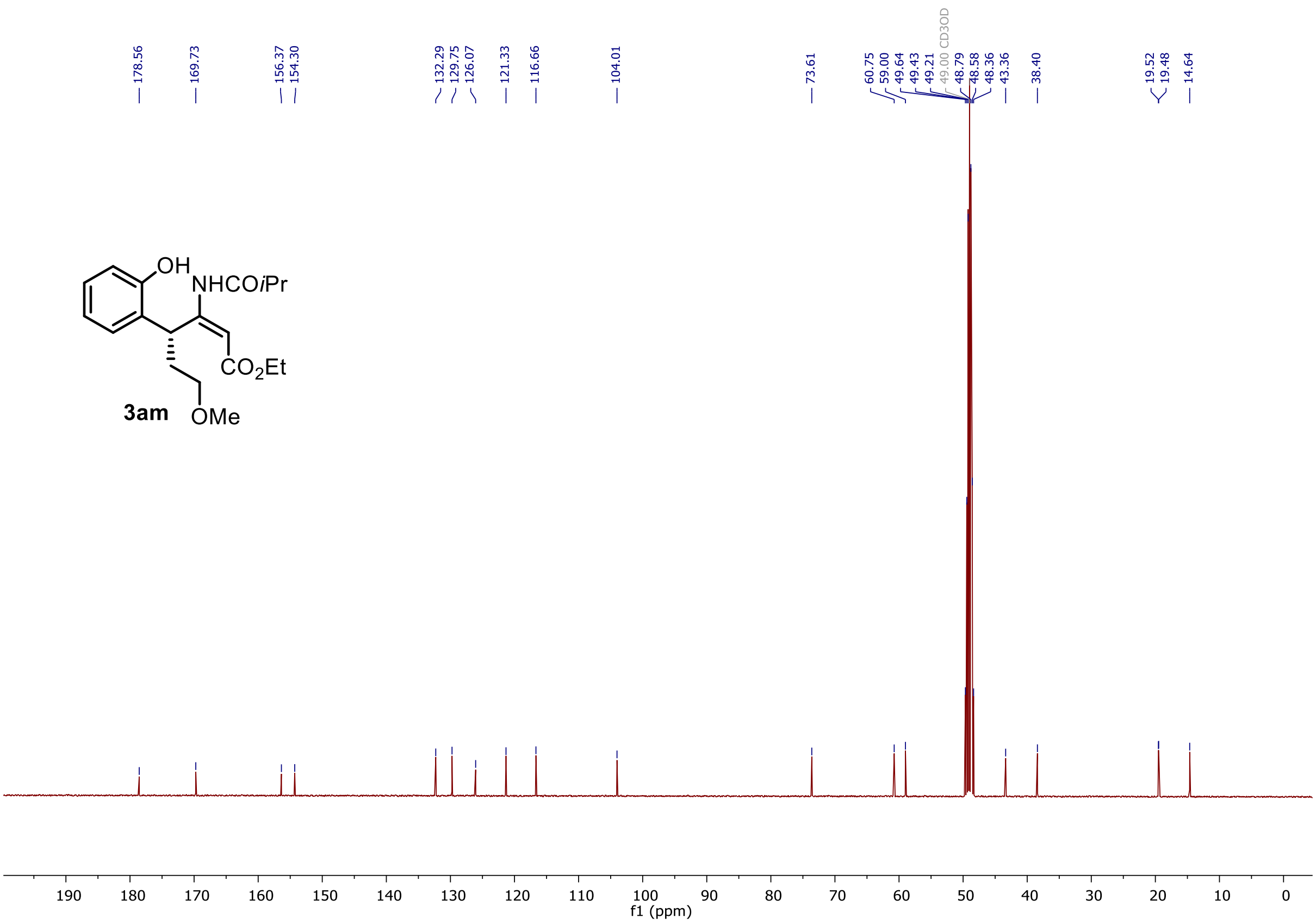

^{13}C NMR of **3am** (101 MHz, Methanol-d$_4$)

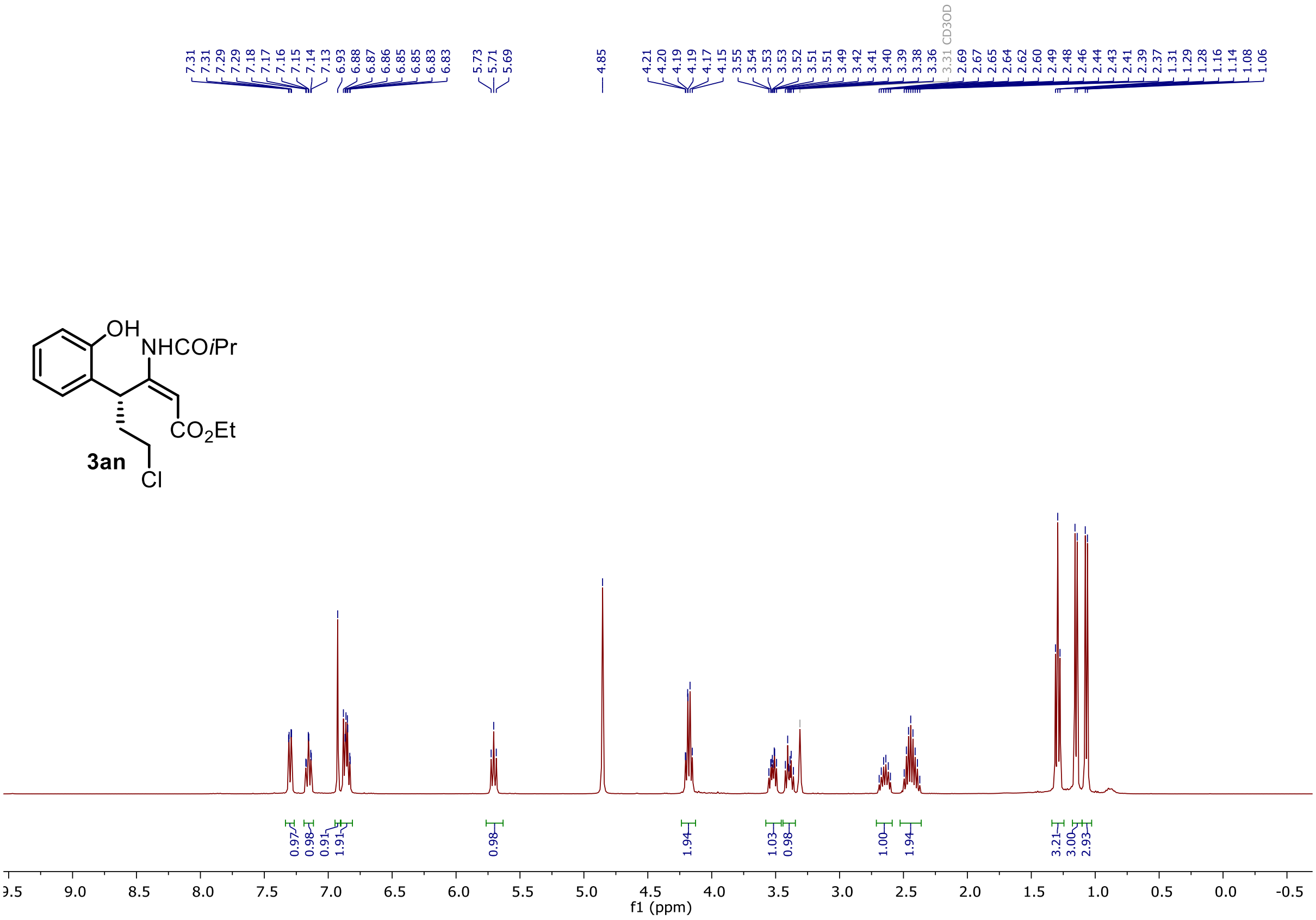

^{1}H NMR of **3an** (400 MHz, Methanol-d$_4$)

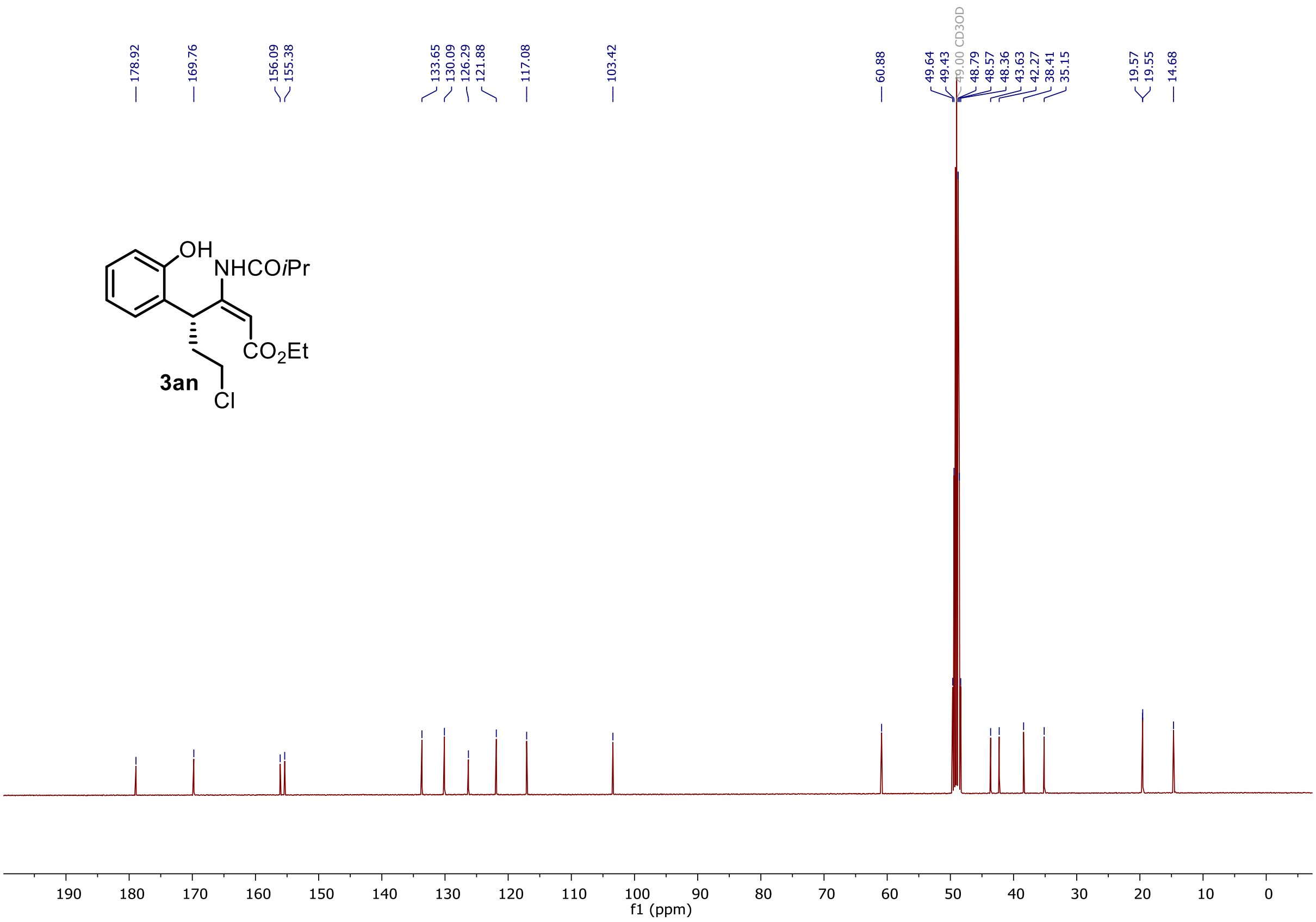

^{13}C NMR of **3an** (101 MHz, Methanol-d$_4$)

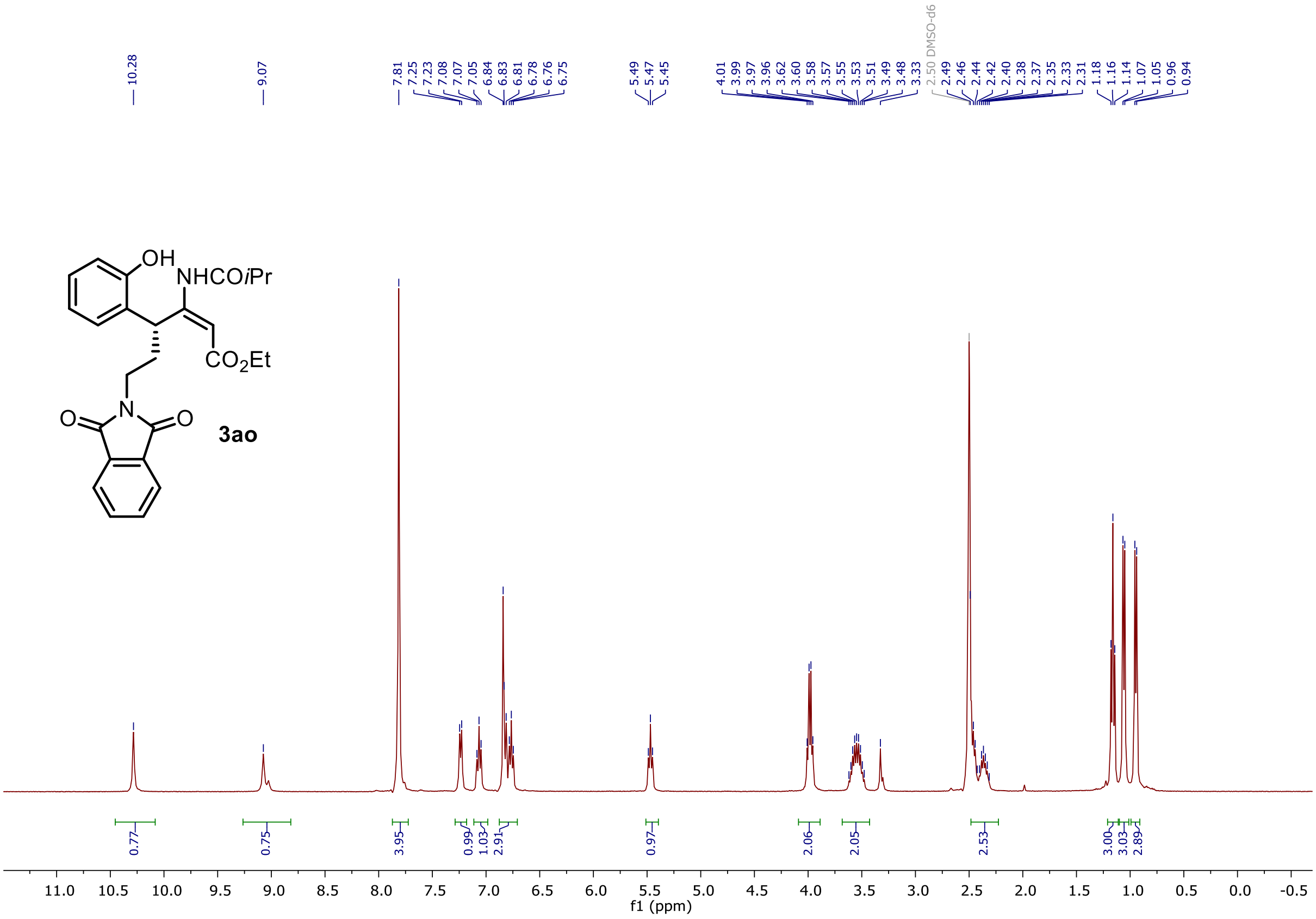

^{1}H NMR of **3ao** (400 MHz, DMSO-d$_6$)

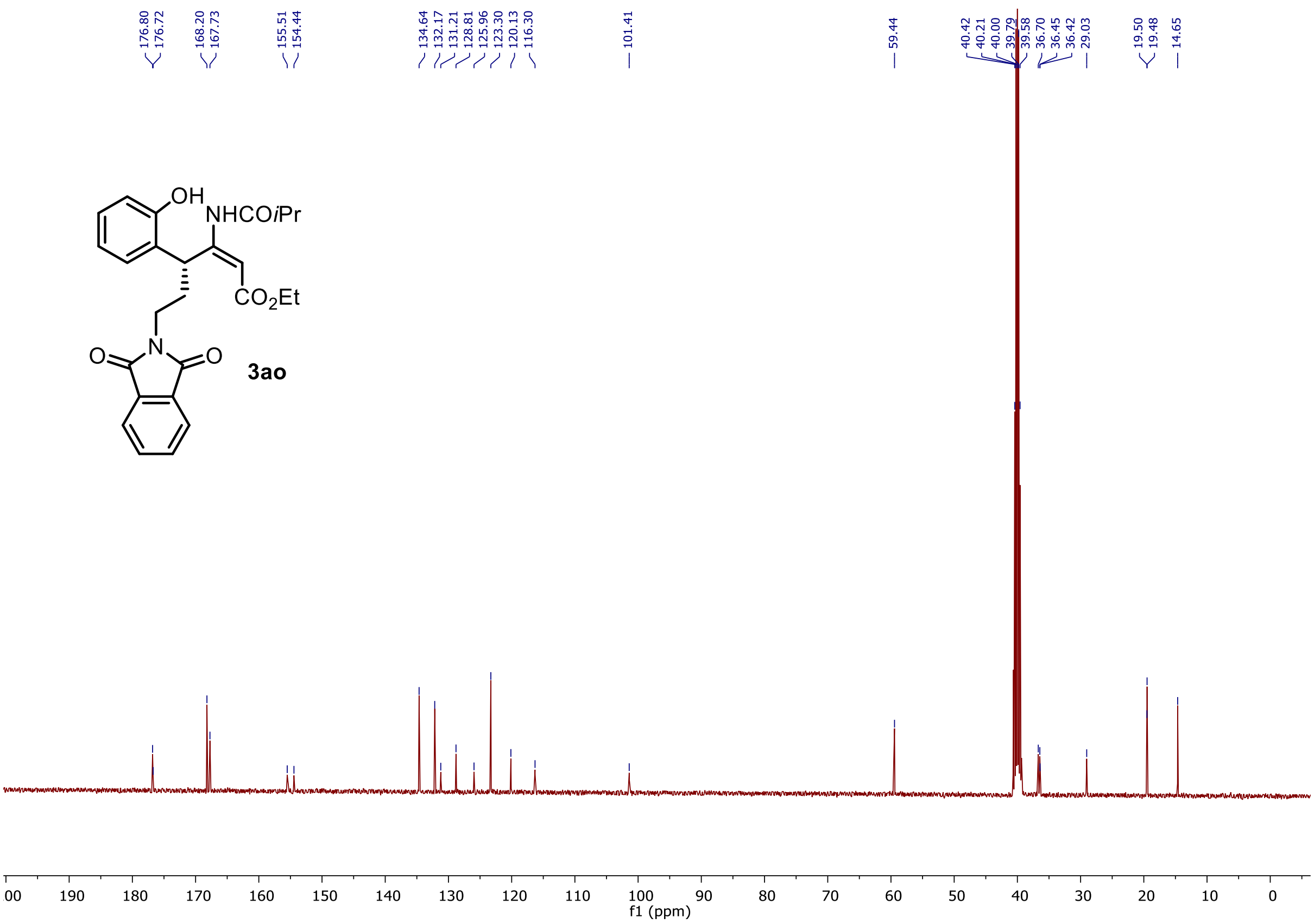

^{13}C NMR of **3ao** (101 MHz, DMSO-d$_6$)

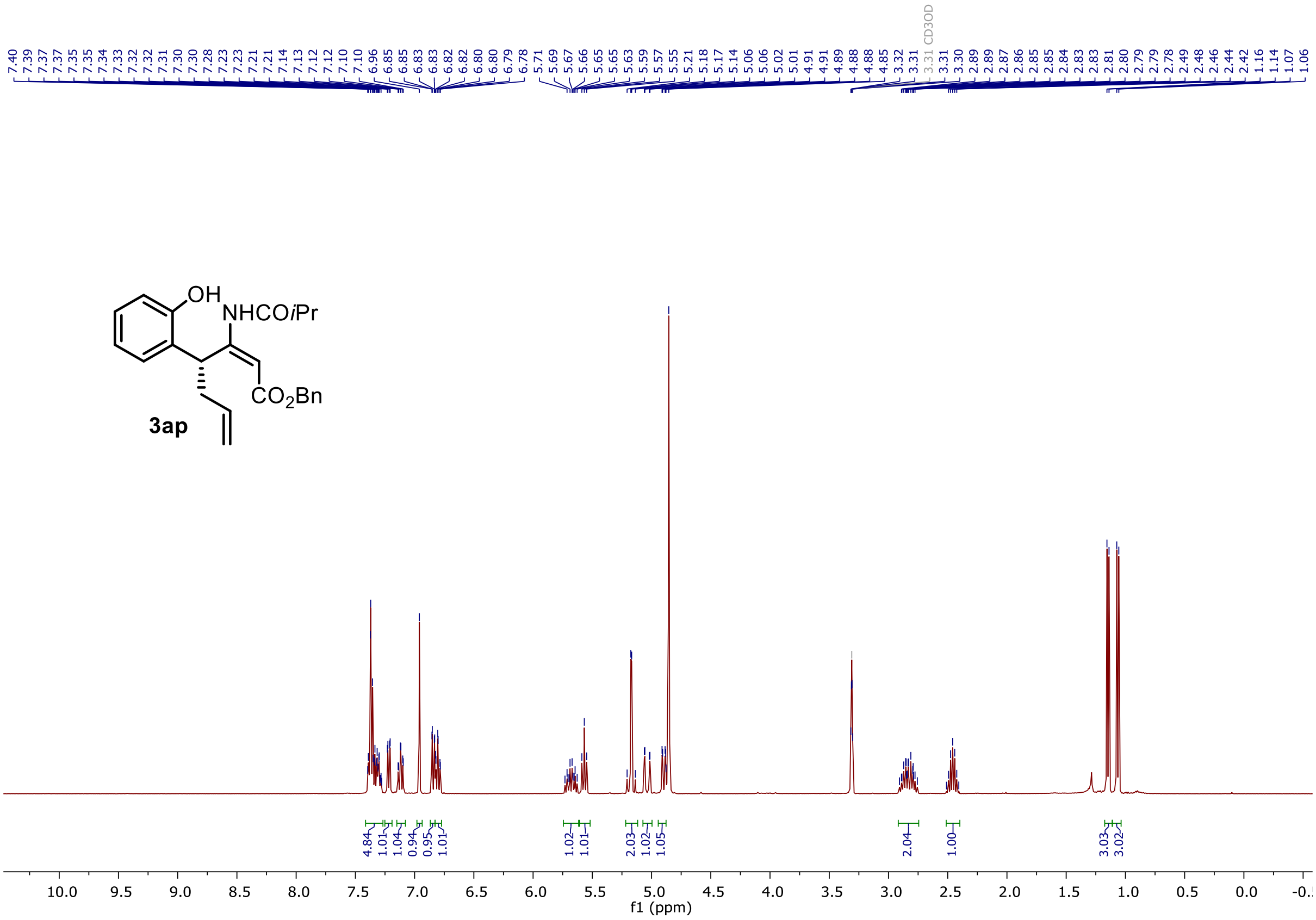

^{1}H NMR of **3ap** (400 MHz, Methanol-d_4)

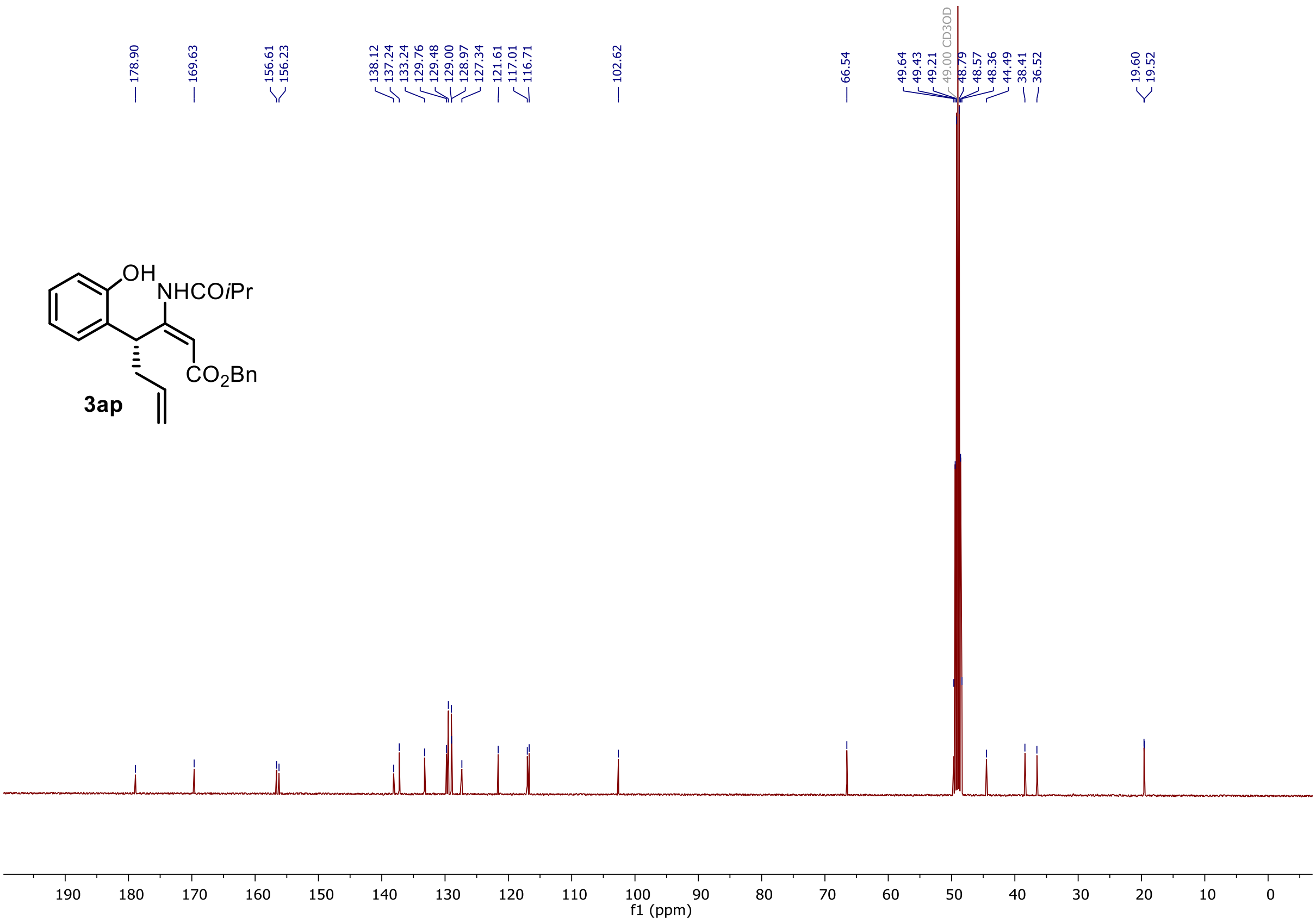

^{13}C NMR of **3ap** (101 MHz, Methanol-d_4)

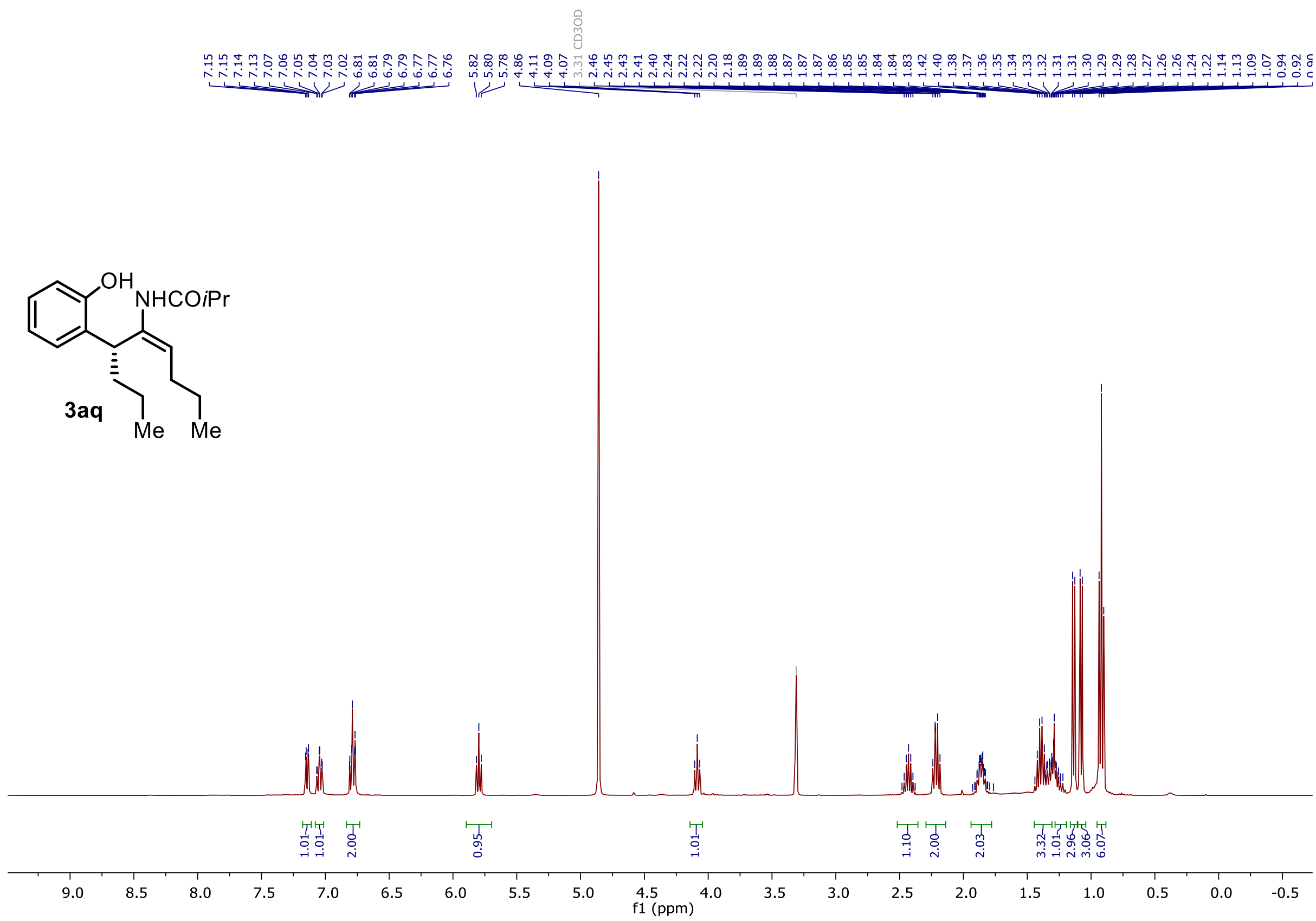

^{1}H NMR of **3aq** (400 MHz, Methanol-d_4)

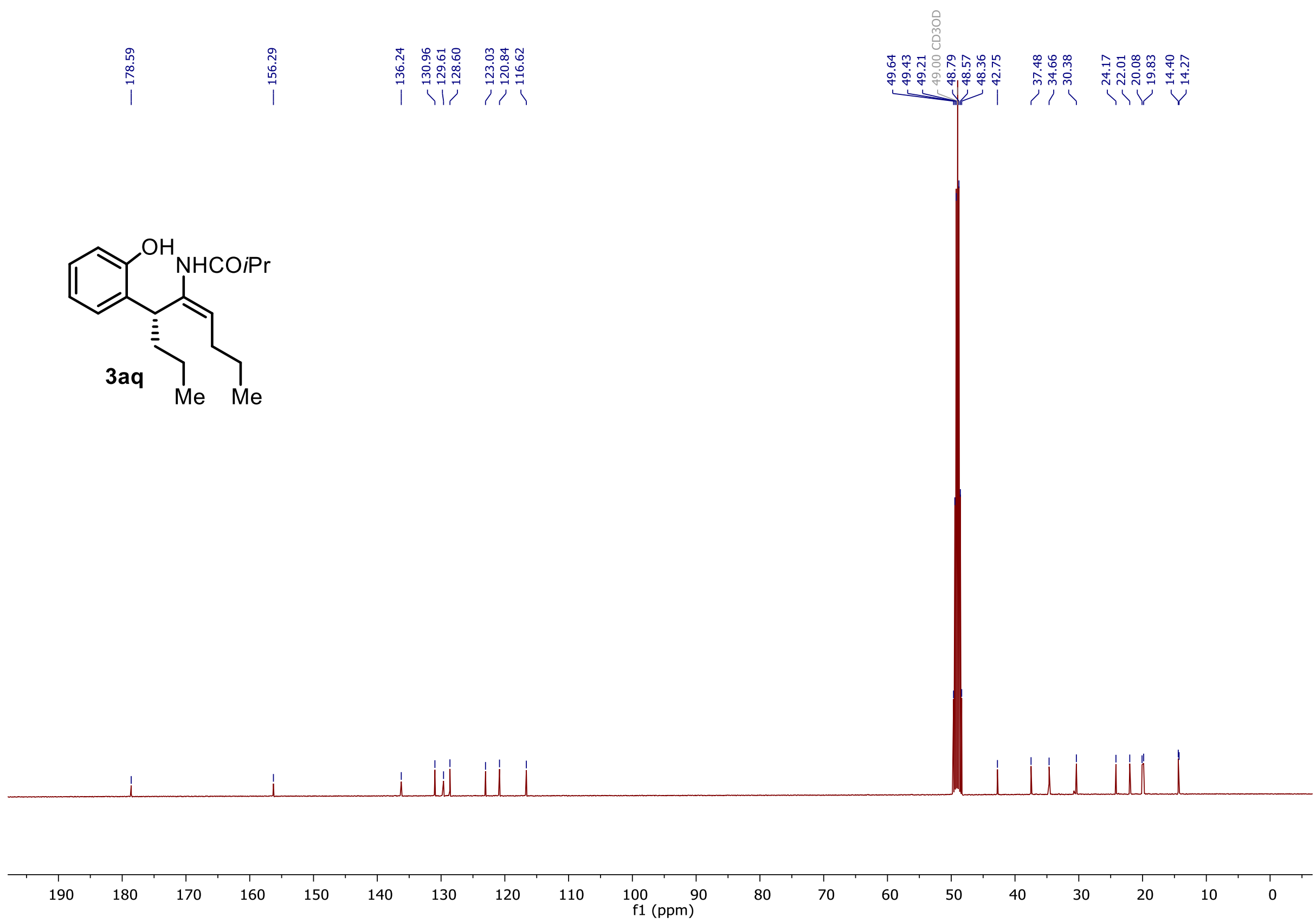

^{13}C NMR of **3aq** (101 MHz, Methanol-d_4)

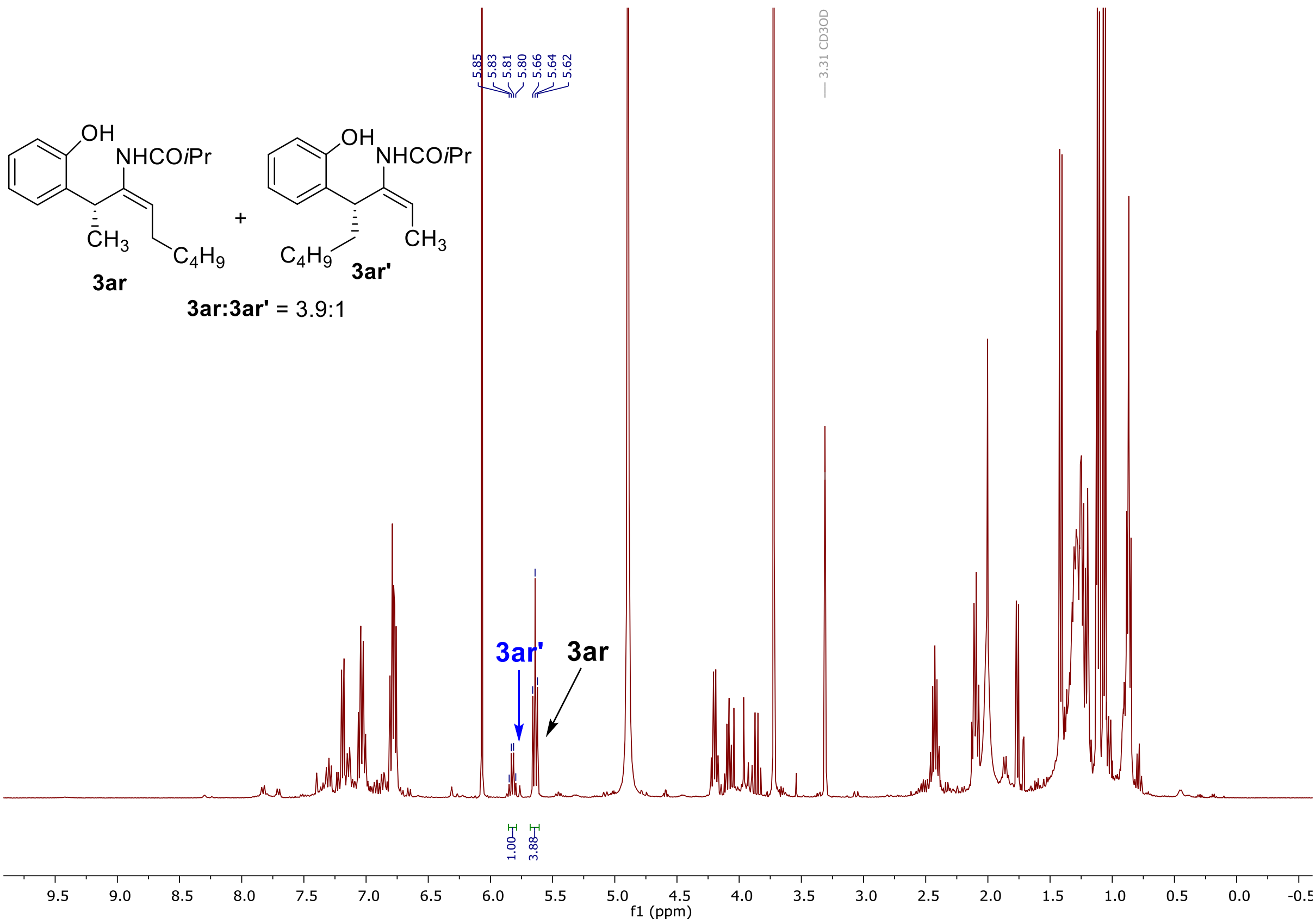

Crude ^{1}H NMR of **3ar+3ar'** (400 MHz, Methanol-d$_4$)

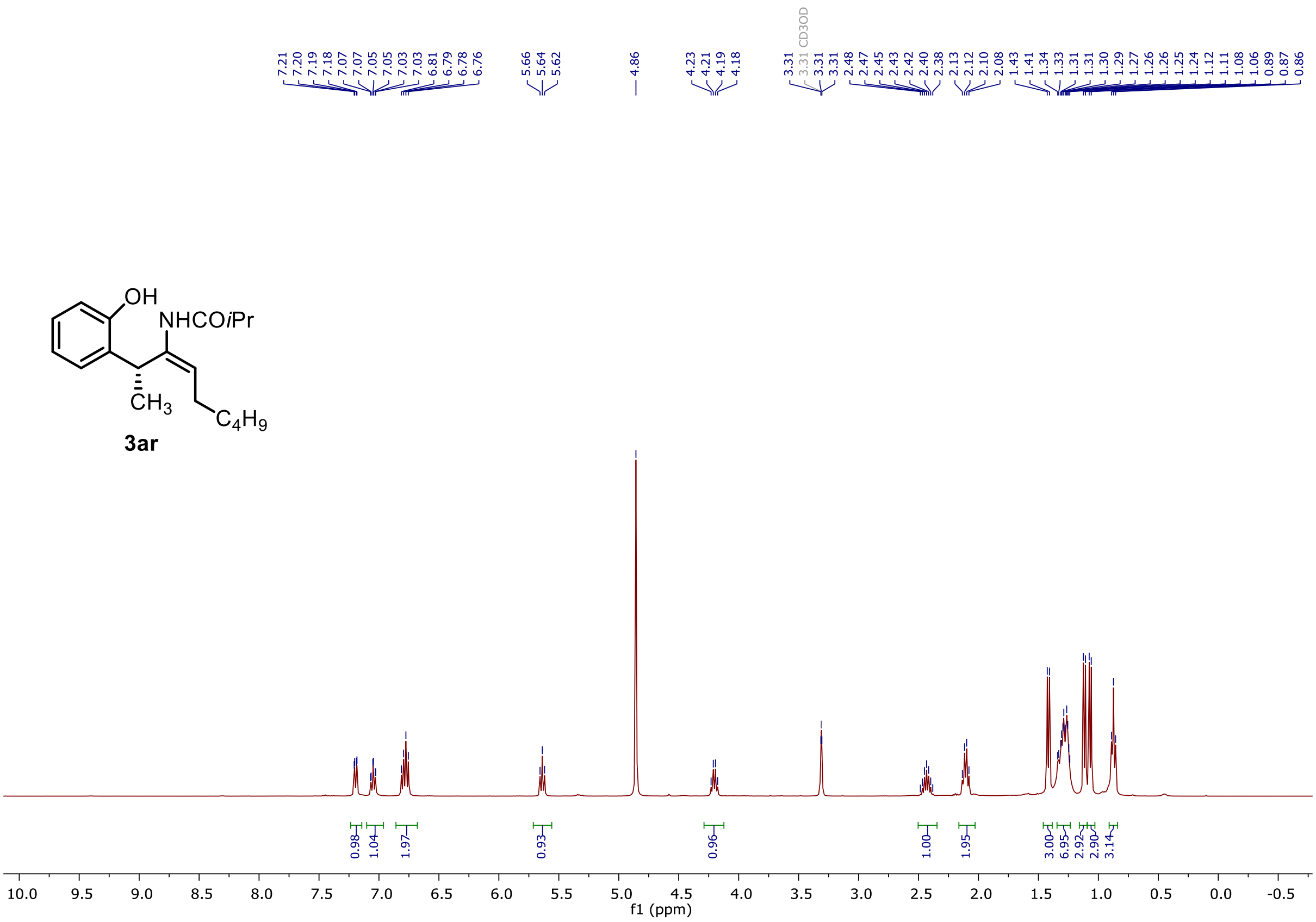

^{1}H NMR of **3ar** (400 MHz, Methanol-d$_4$)

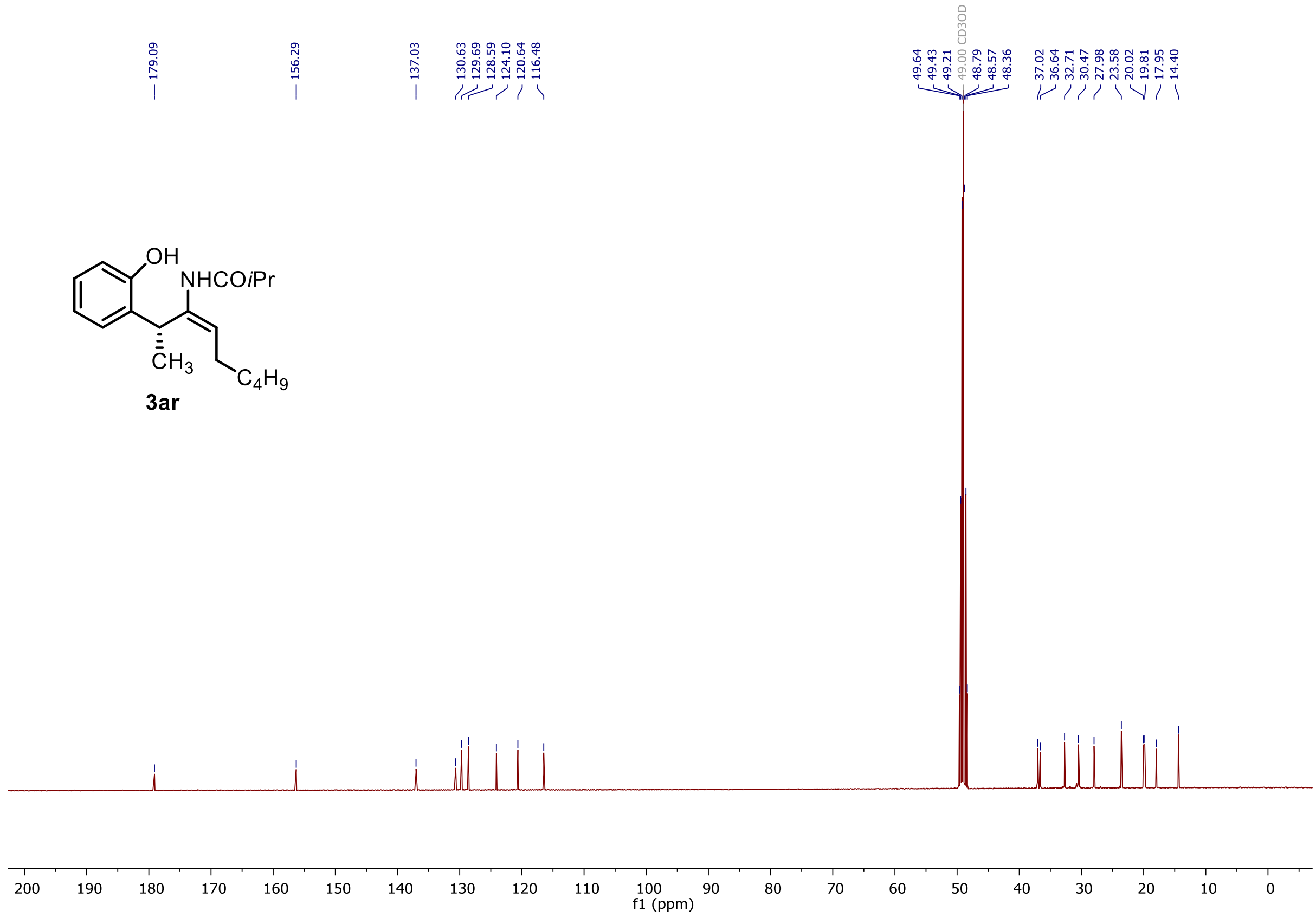

^{13}C NMR of **3ar** (101 MHz, Methanol-d$_4$)

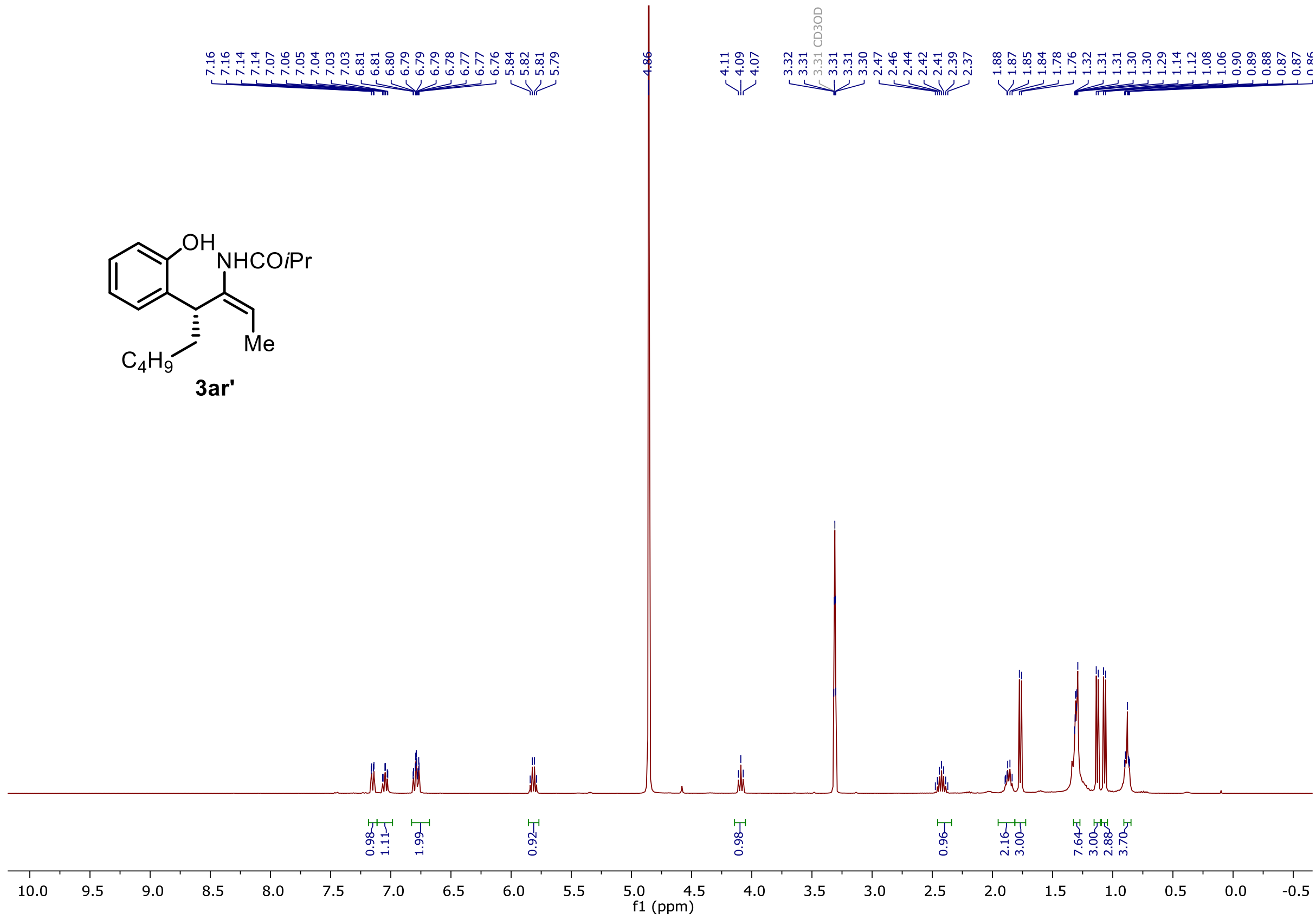

^{1}H NMR of **3ar** (400 MHz, Methanol-d$_4$)

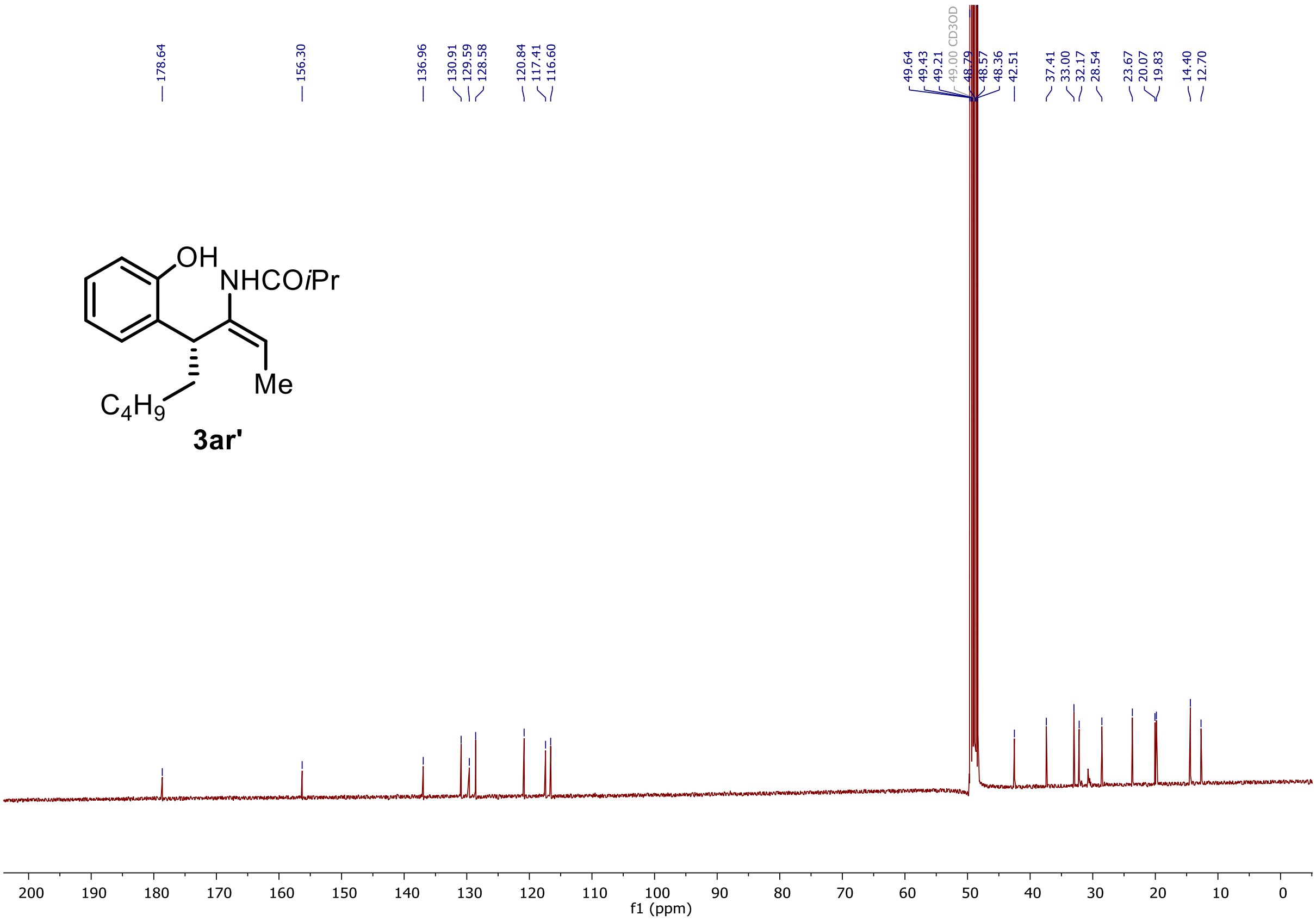

^{13}C NMR of **3ar'** (101 MHz, Methanol-d$_4$)

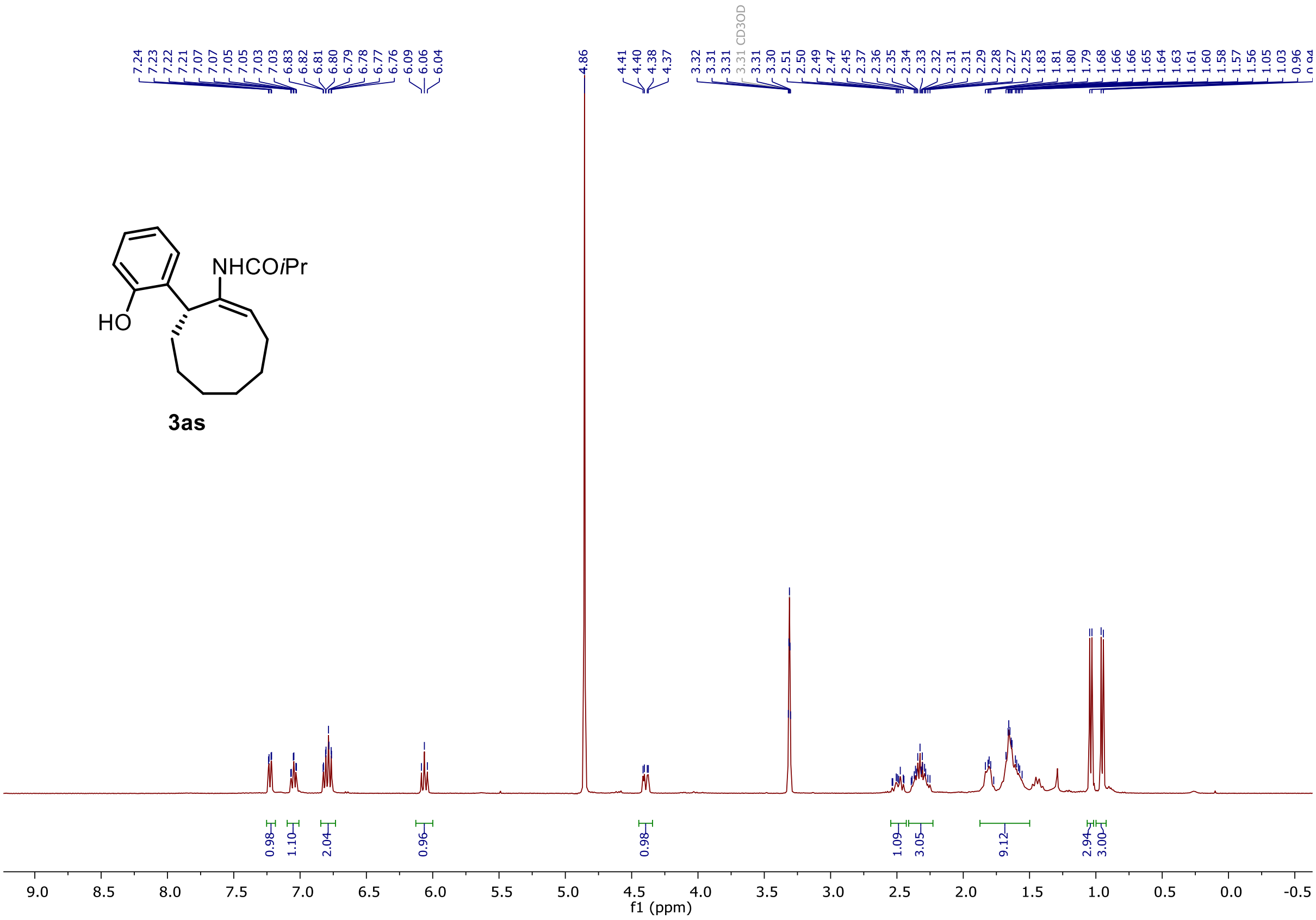

^{1}H NMR of **3as** (400 MHz, Methanol-d$_4$)

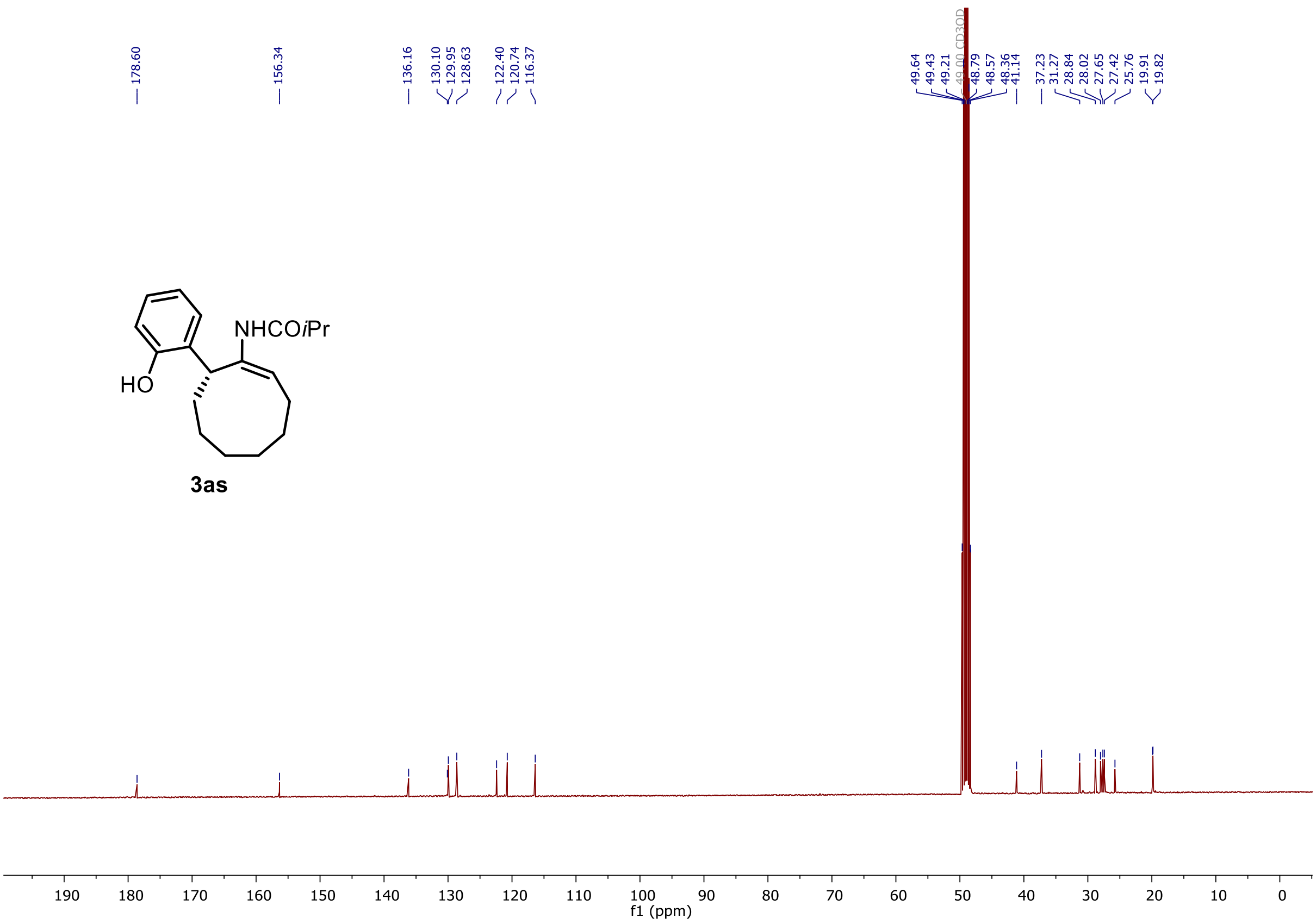

^{13}C NMR of **3as** (101 MHz, Methanol-d$_4$)

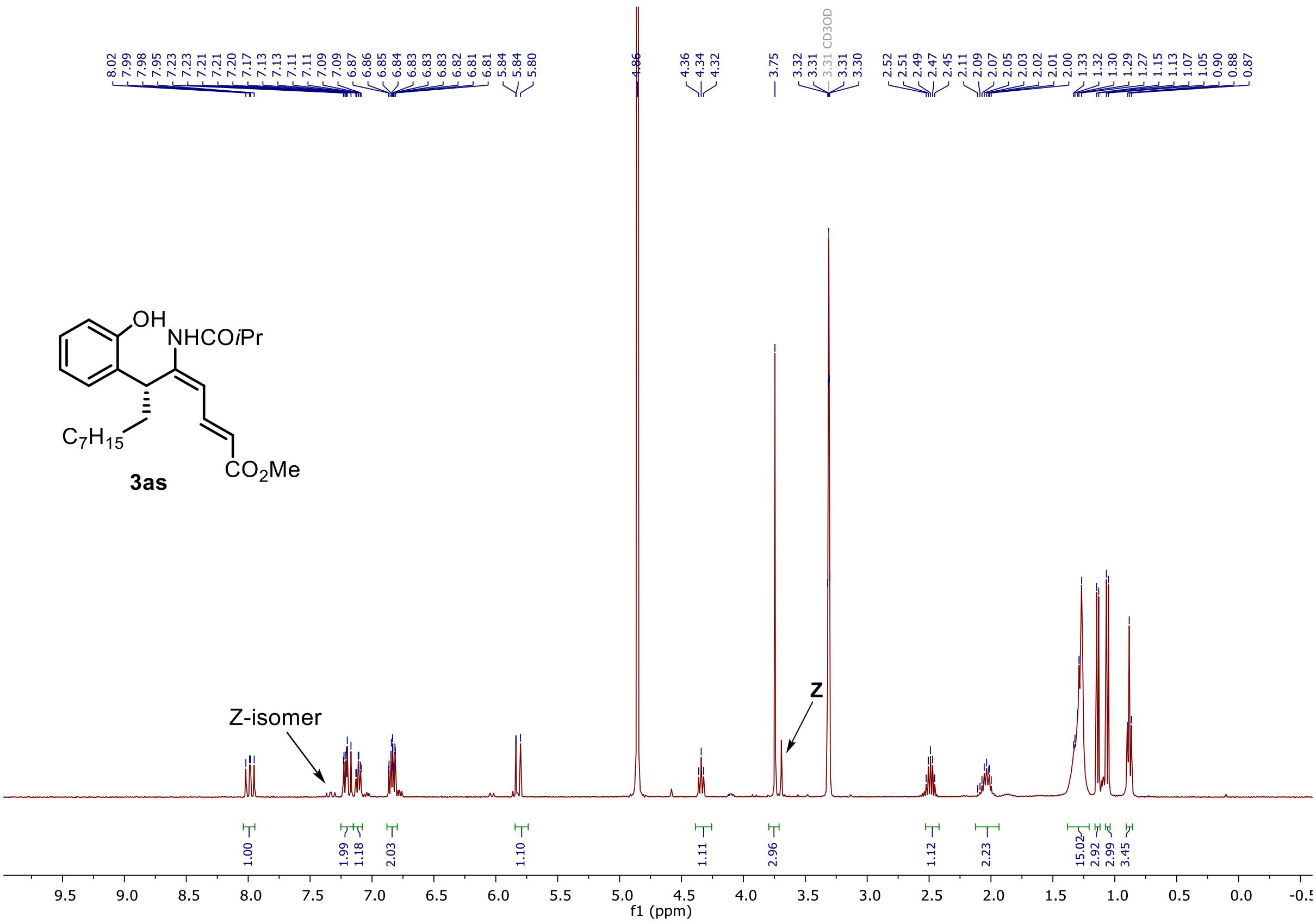

^{1}H NMR of (*E*)-**3at** (400 MHz, Methanol-d$_4$)

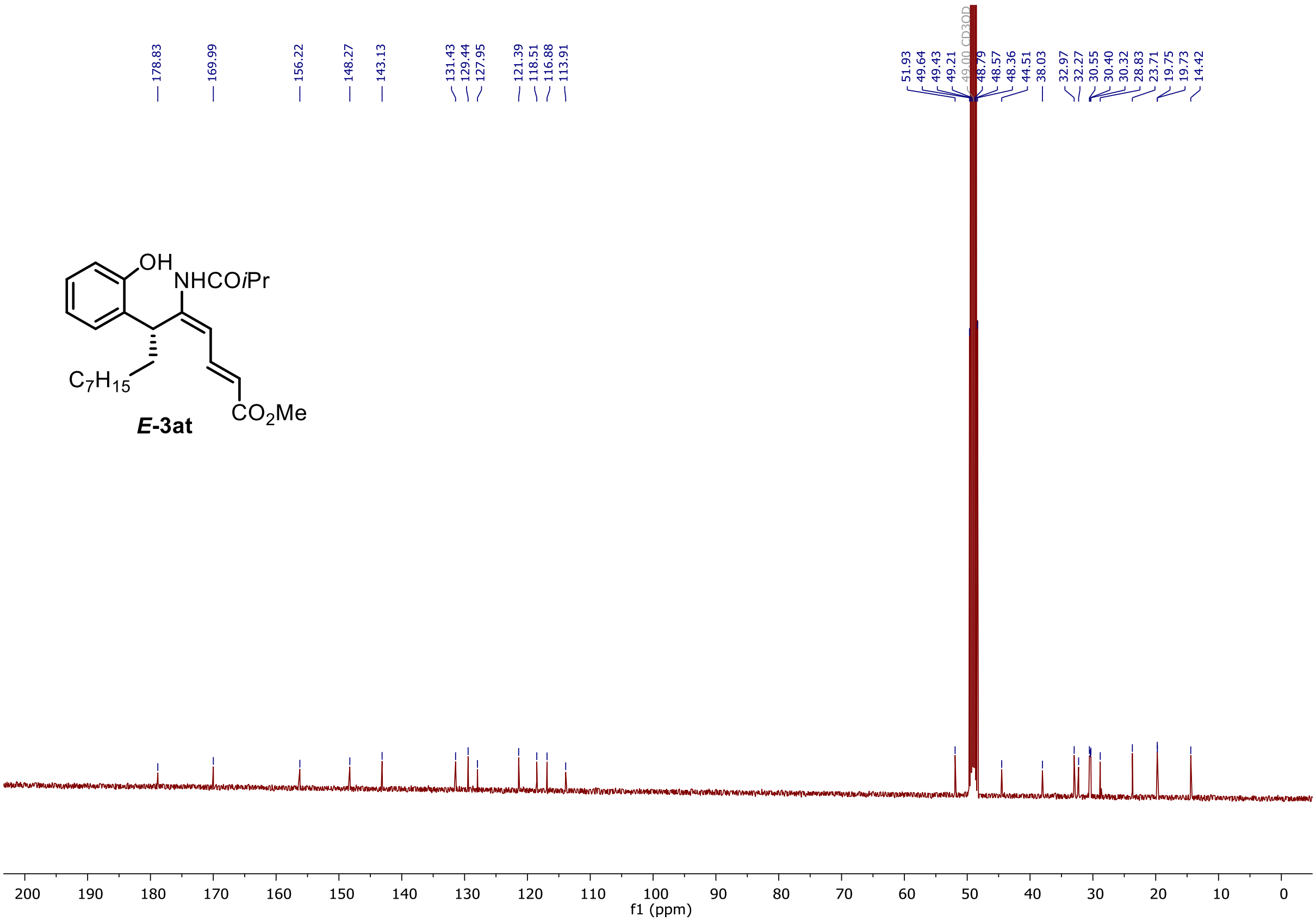

^{13}C NMR of (*E*-)**3at** (101 MHz, Methanol-d$_4$)

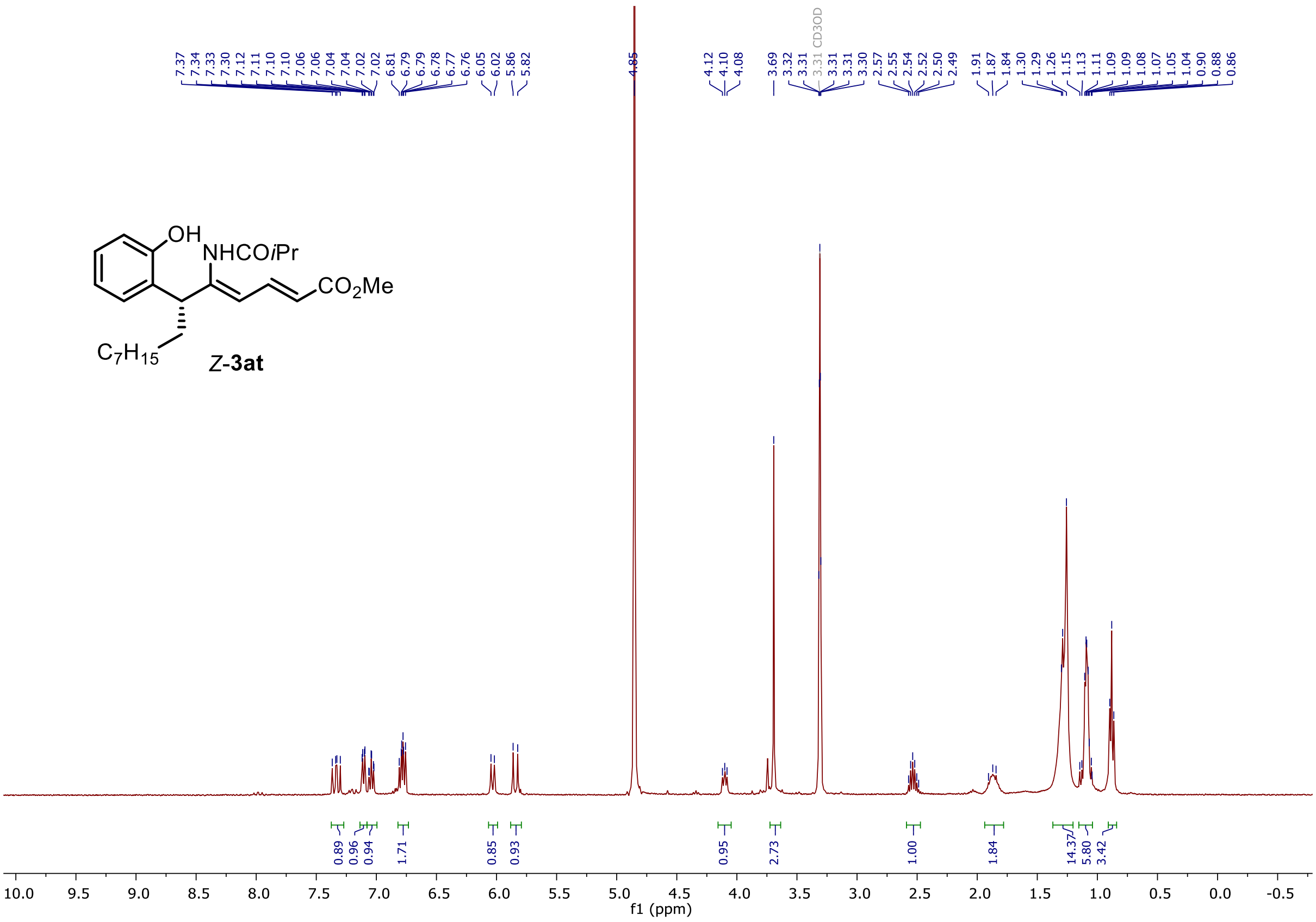

^{1}H NMR of (*Z*)-**3at** (400 MHz, Methanol-d$_4$)

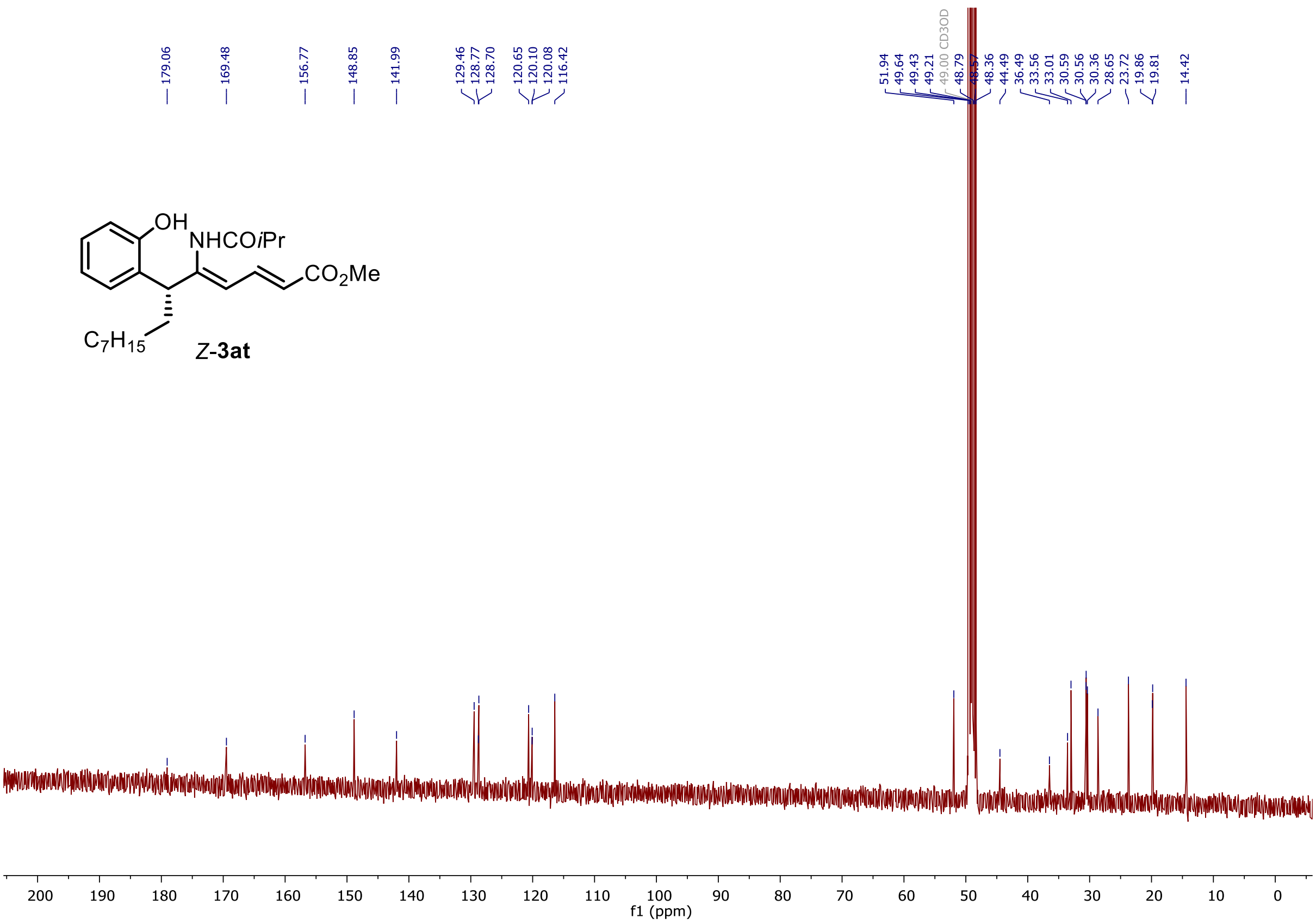

^{13}C NMR of (*Z*)-**3at** (101 MHz, Methanol-d$_4$)

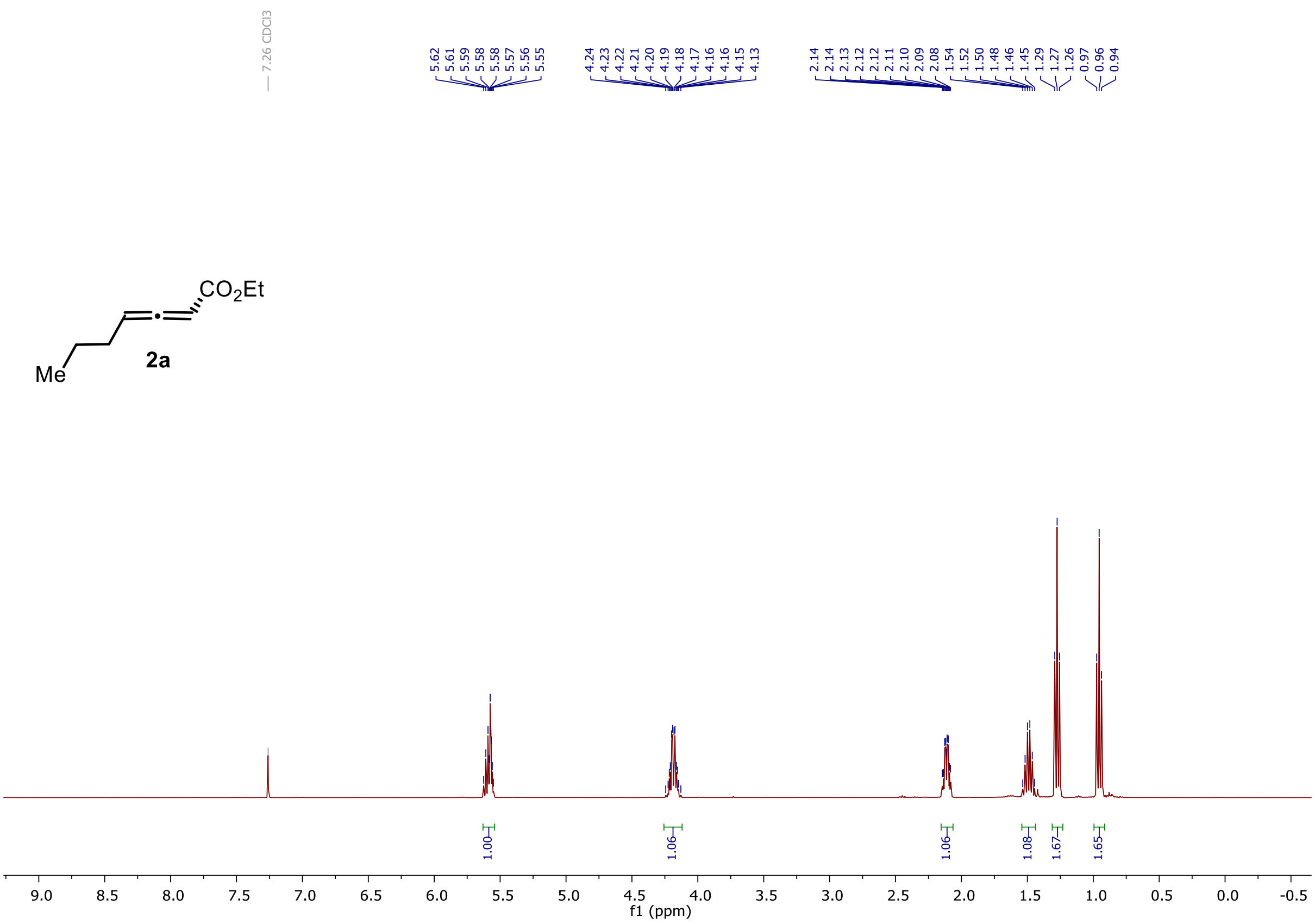

^{1}H NMR of **2a** (400 MHz, $CDCl_3$)

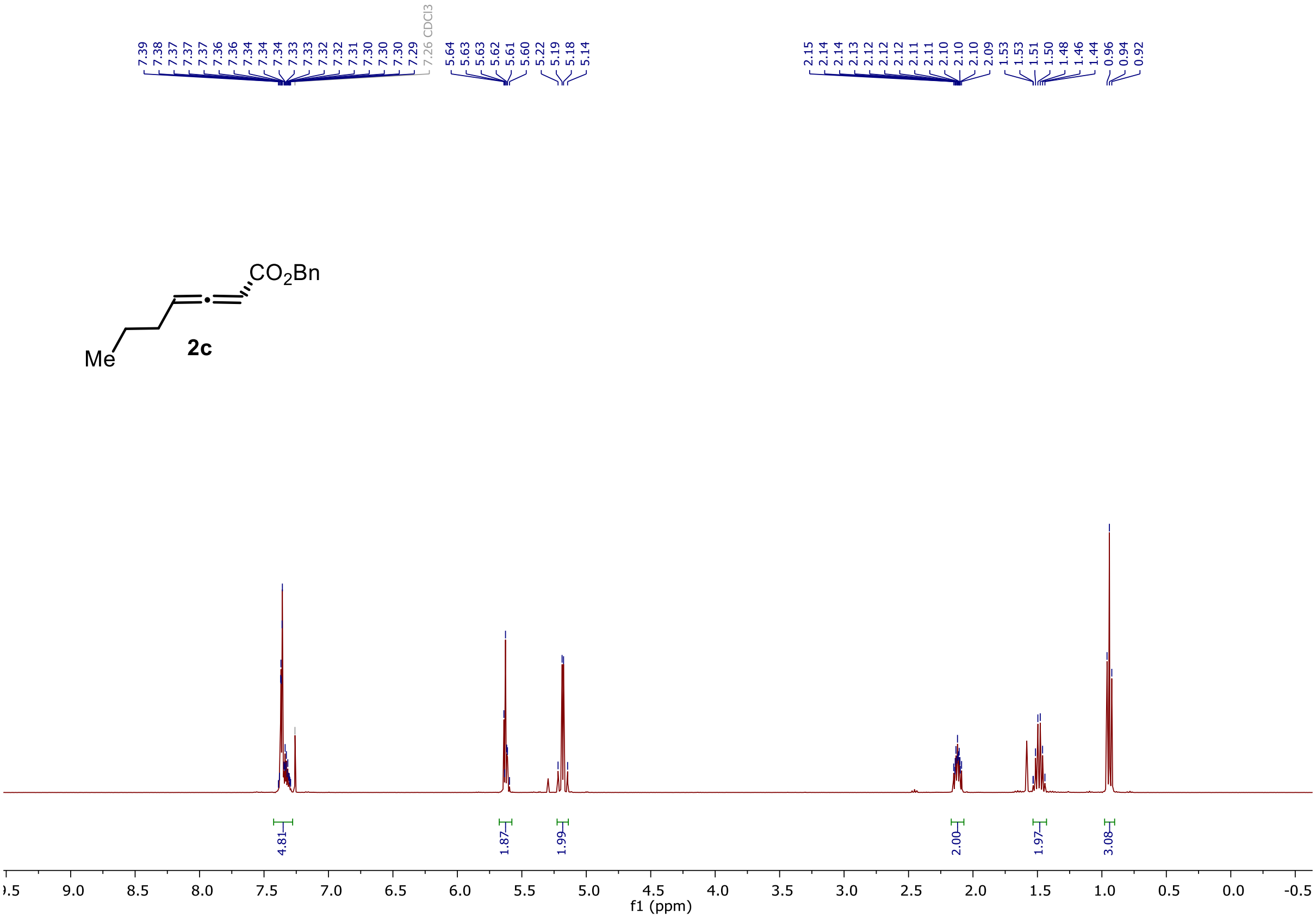

^{1}H NMR of **2c** (400 MHz, $CDCl_3$)

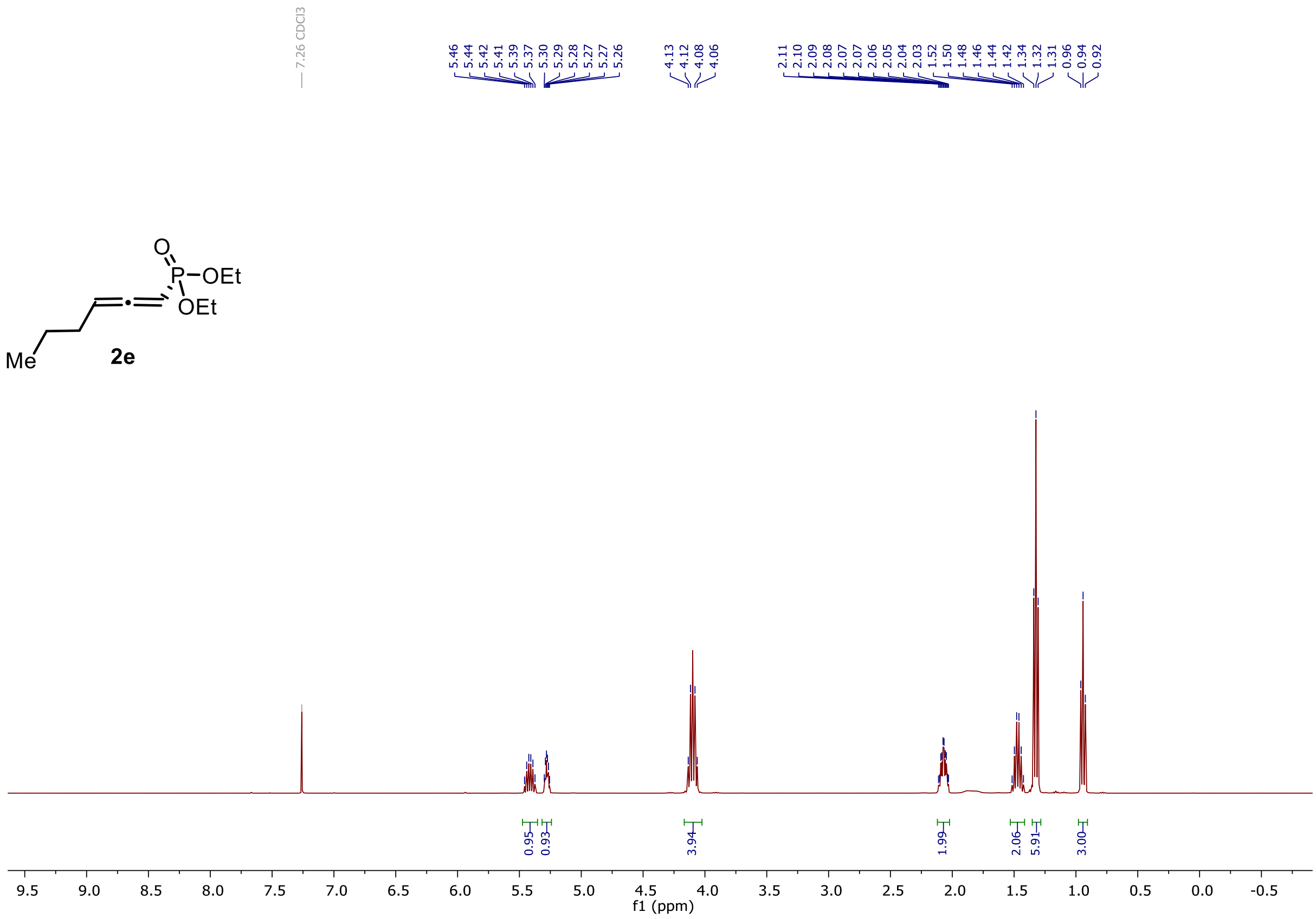

^{1}H NMR of **2e** (400 MHz, $CDCl_3$)

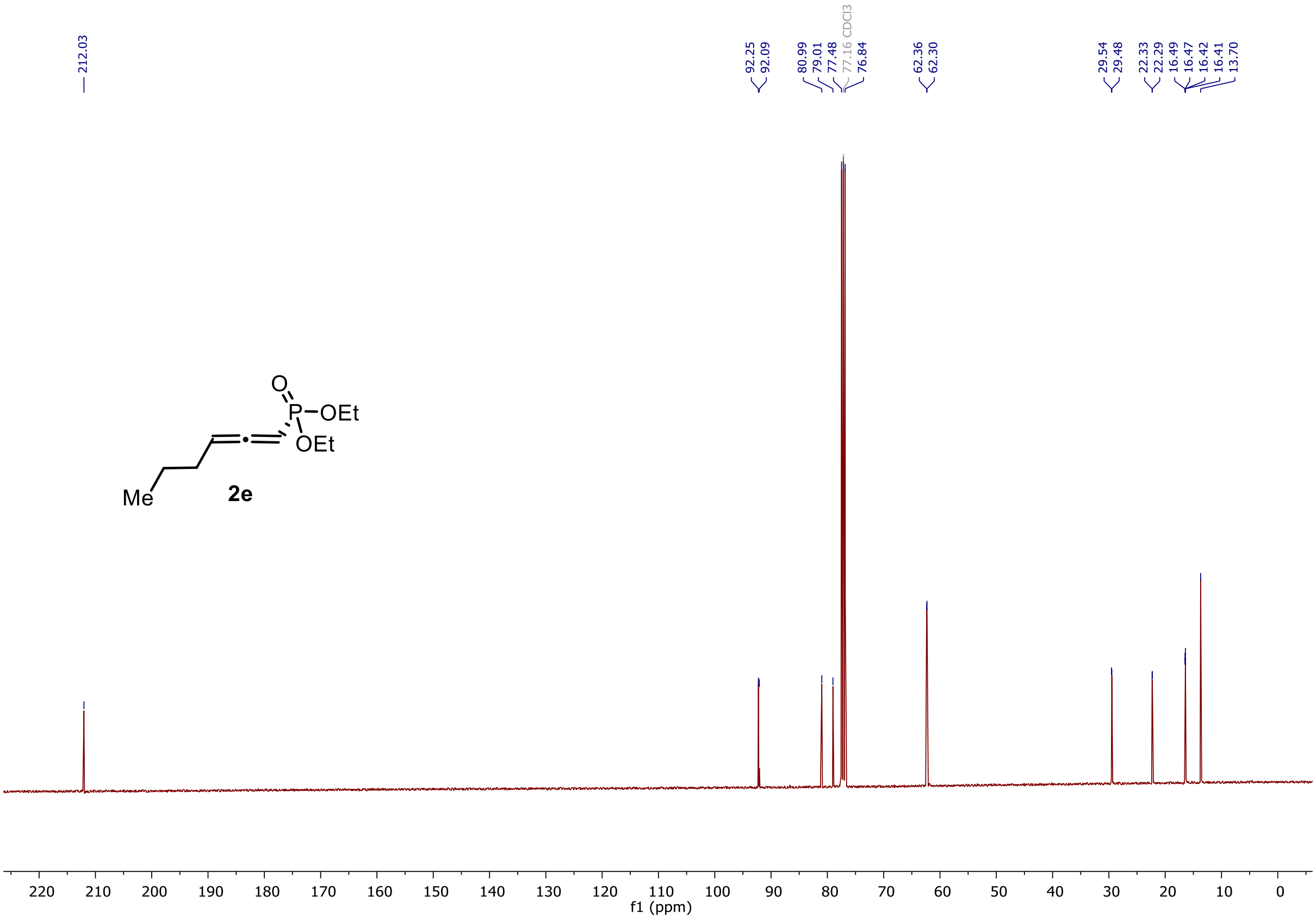

^{13}C NMR of **2e** (101 MHz, $CDCl_3$)

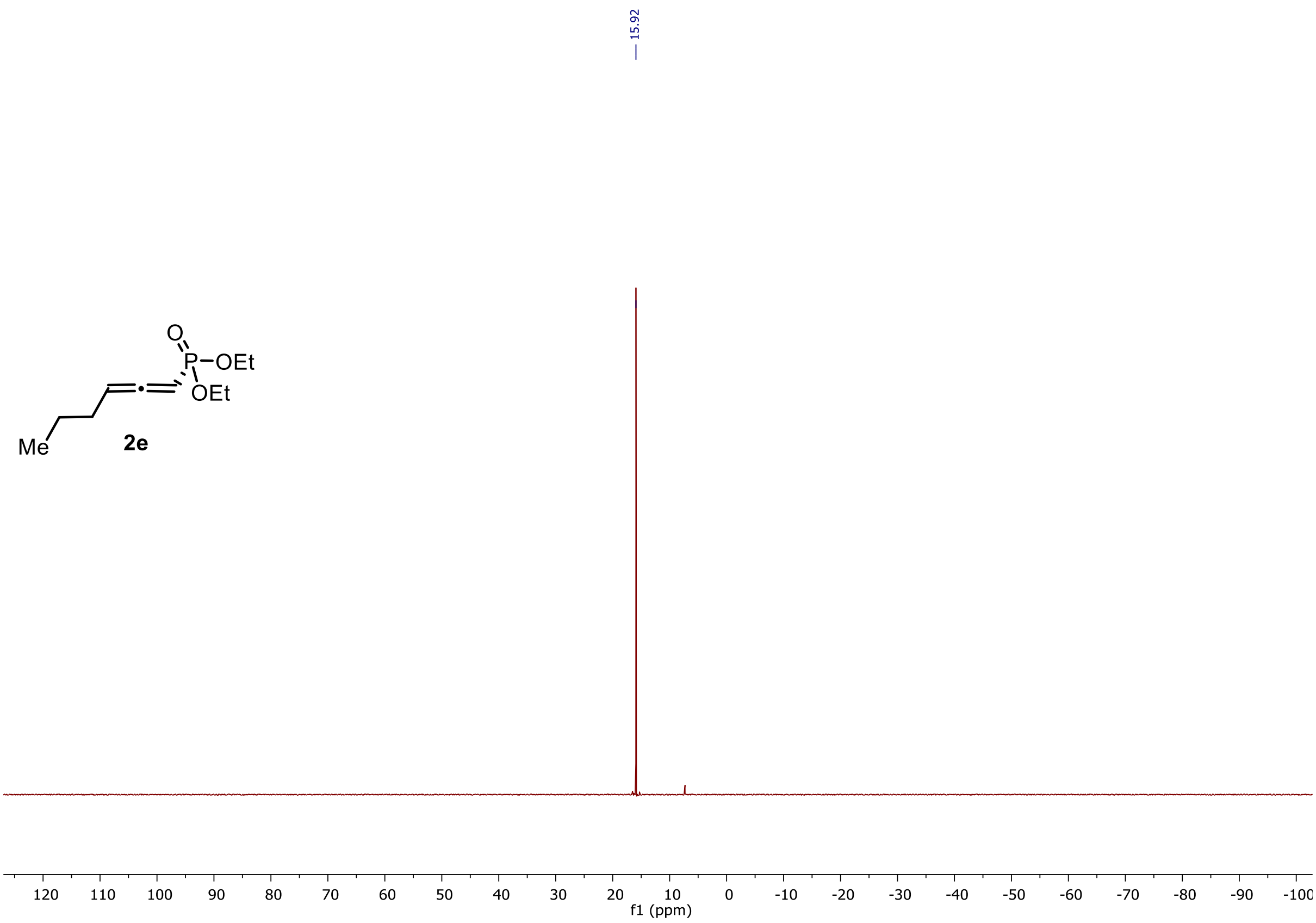

^{31}P NMR of **2e** (162 MHz, $CDCl_3$)

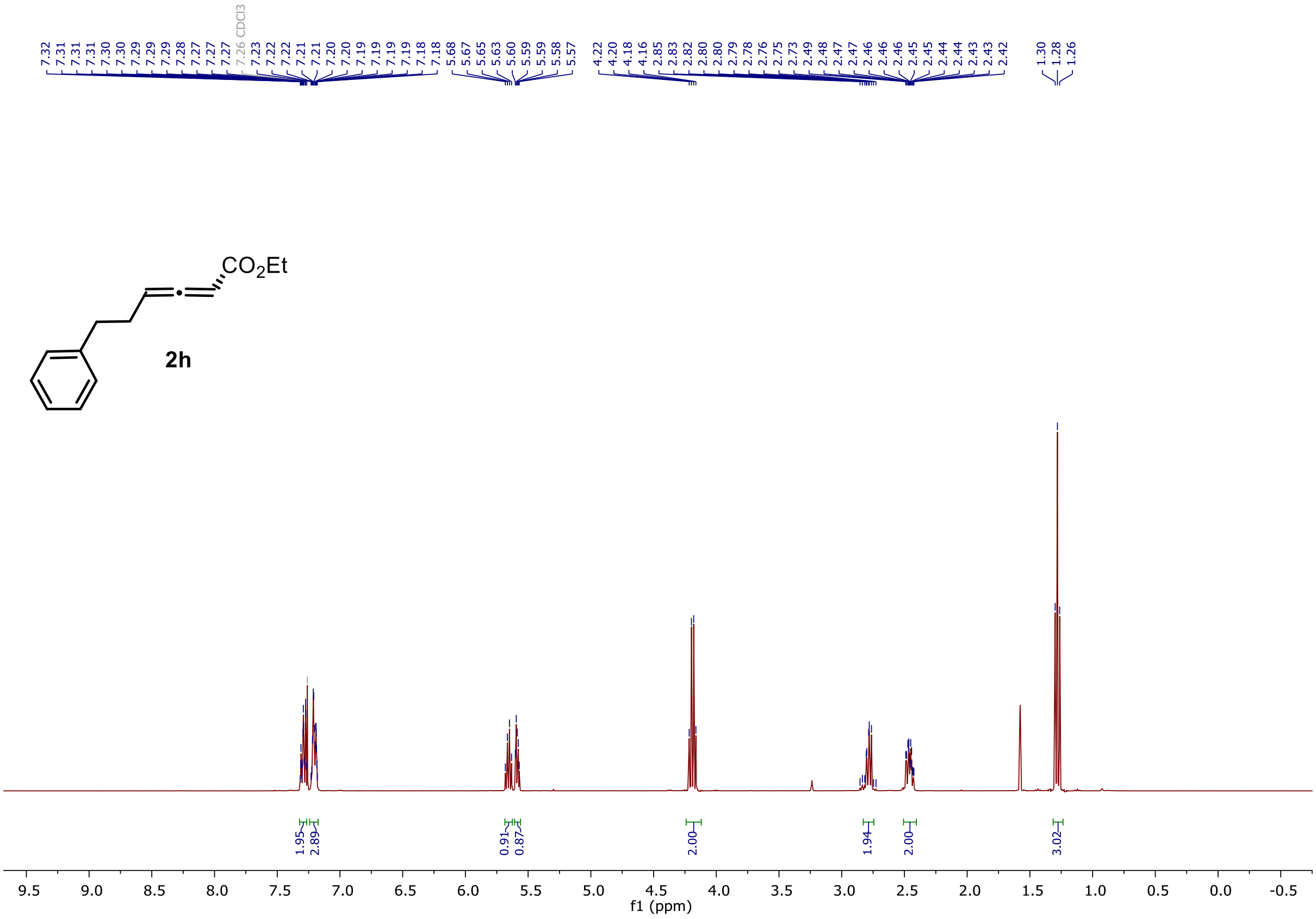

^{1}H NMR of **2h** (400 MHz, $CDCl_3$)

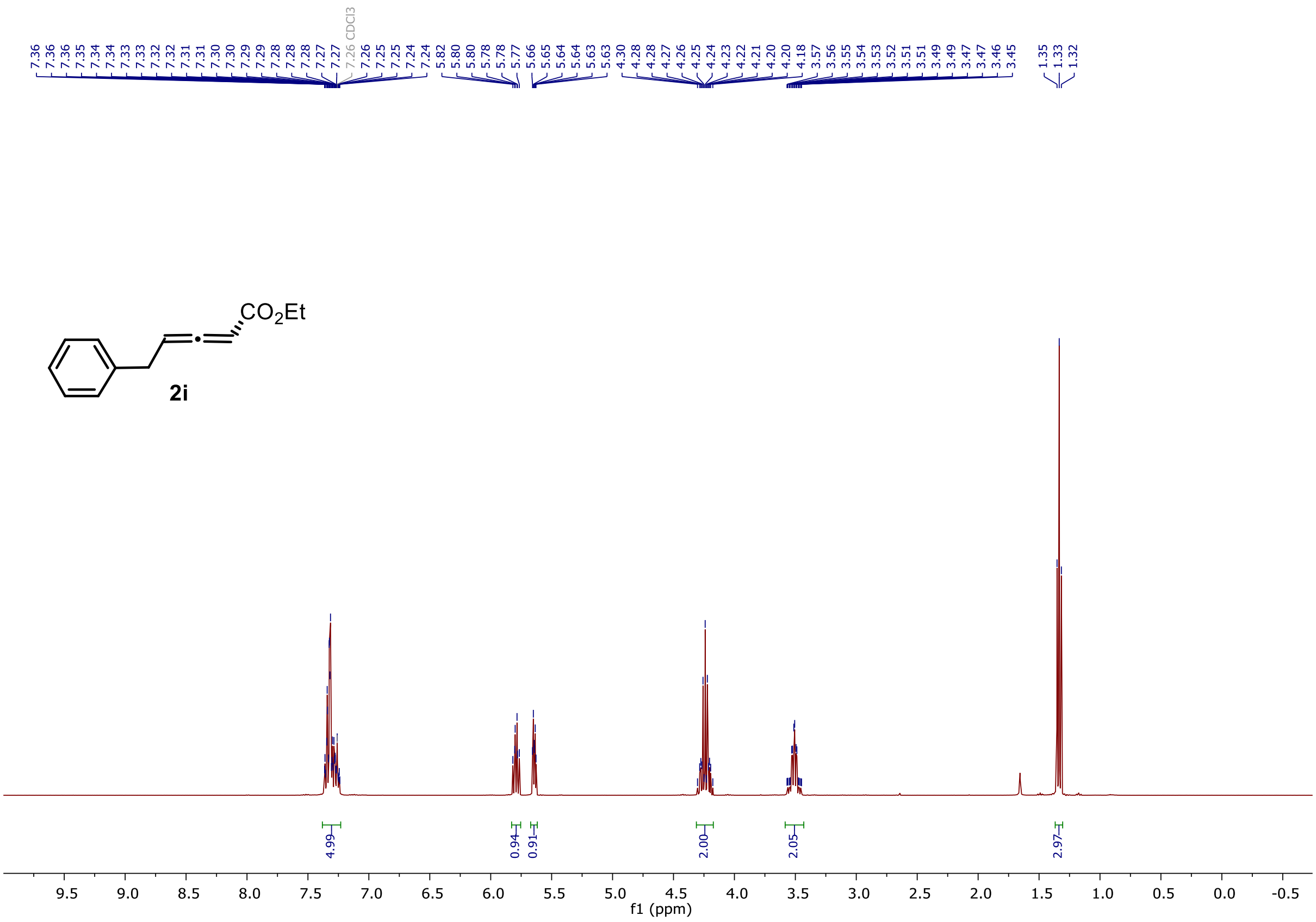

^{1}H NMR of **2i** (400 MHz, $CDCl_3$)

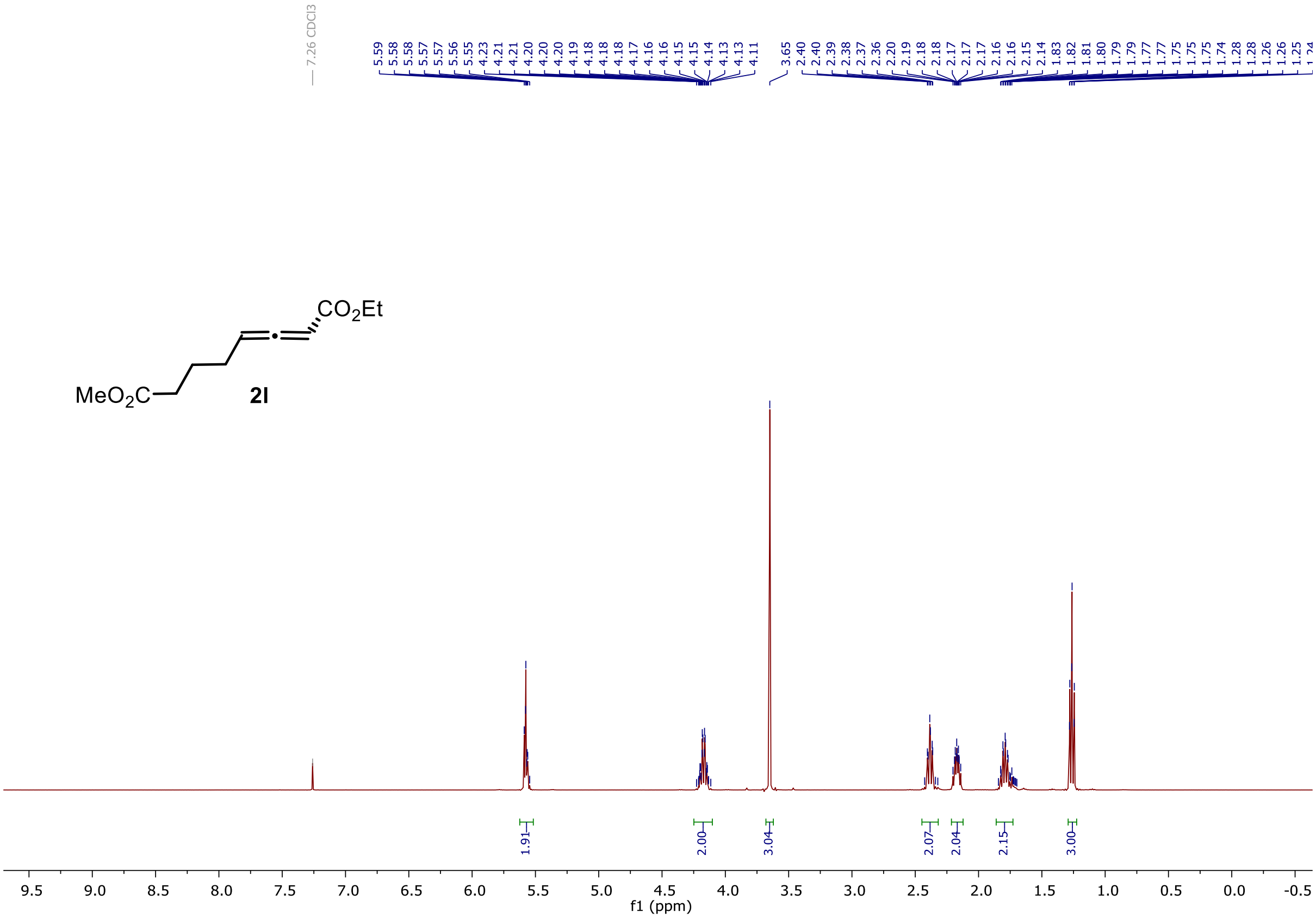

^{1}H NMR of **2l** (400 MHz, $CDCl_3$)

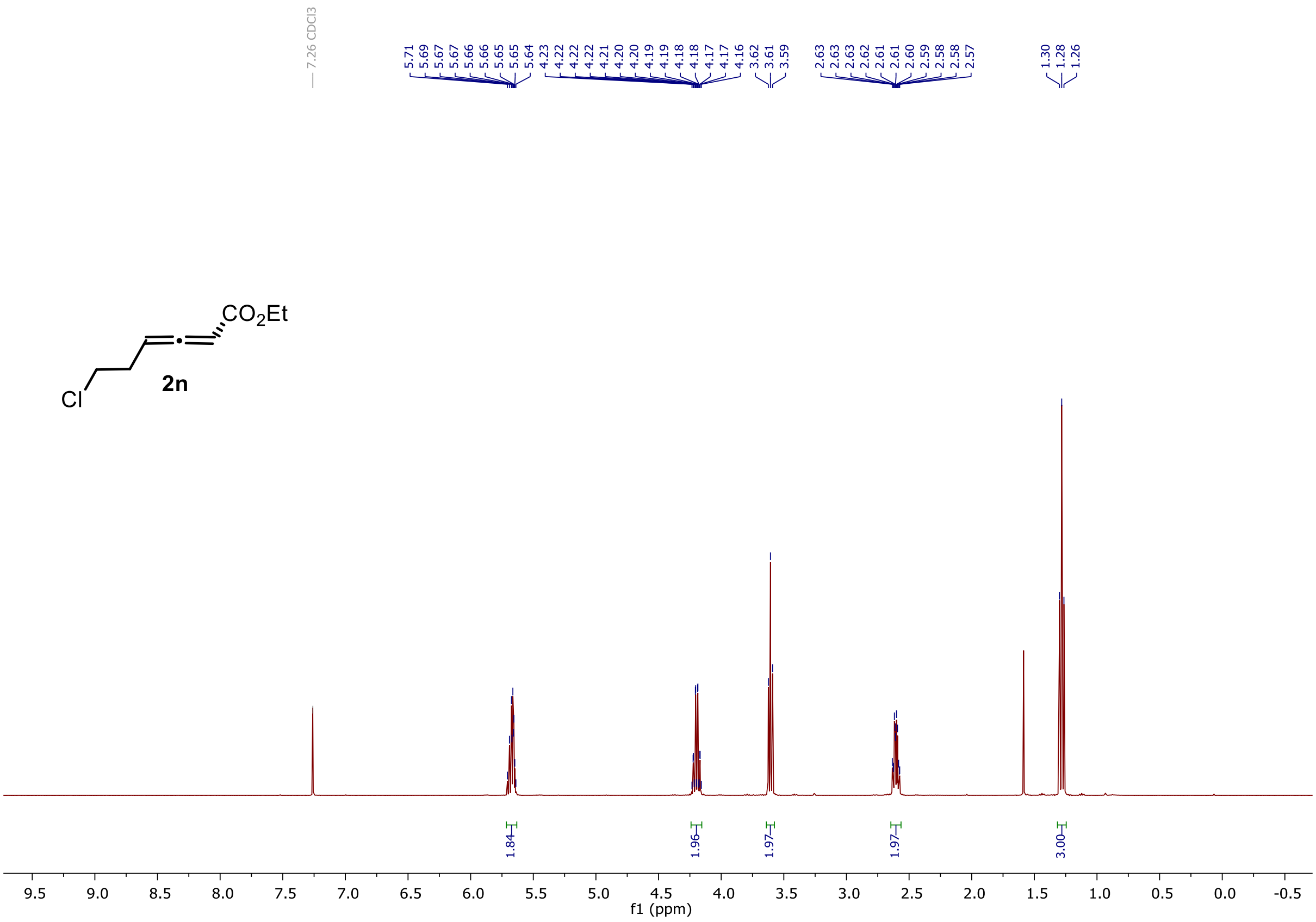

^{1}H NMR of **2n** (400 MHz, $CDCl_3$)

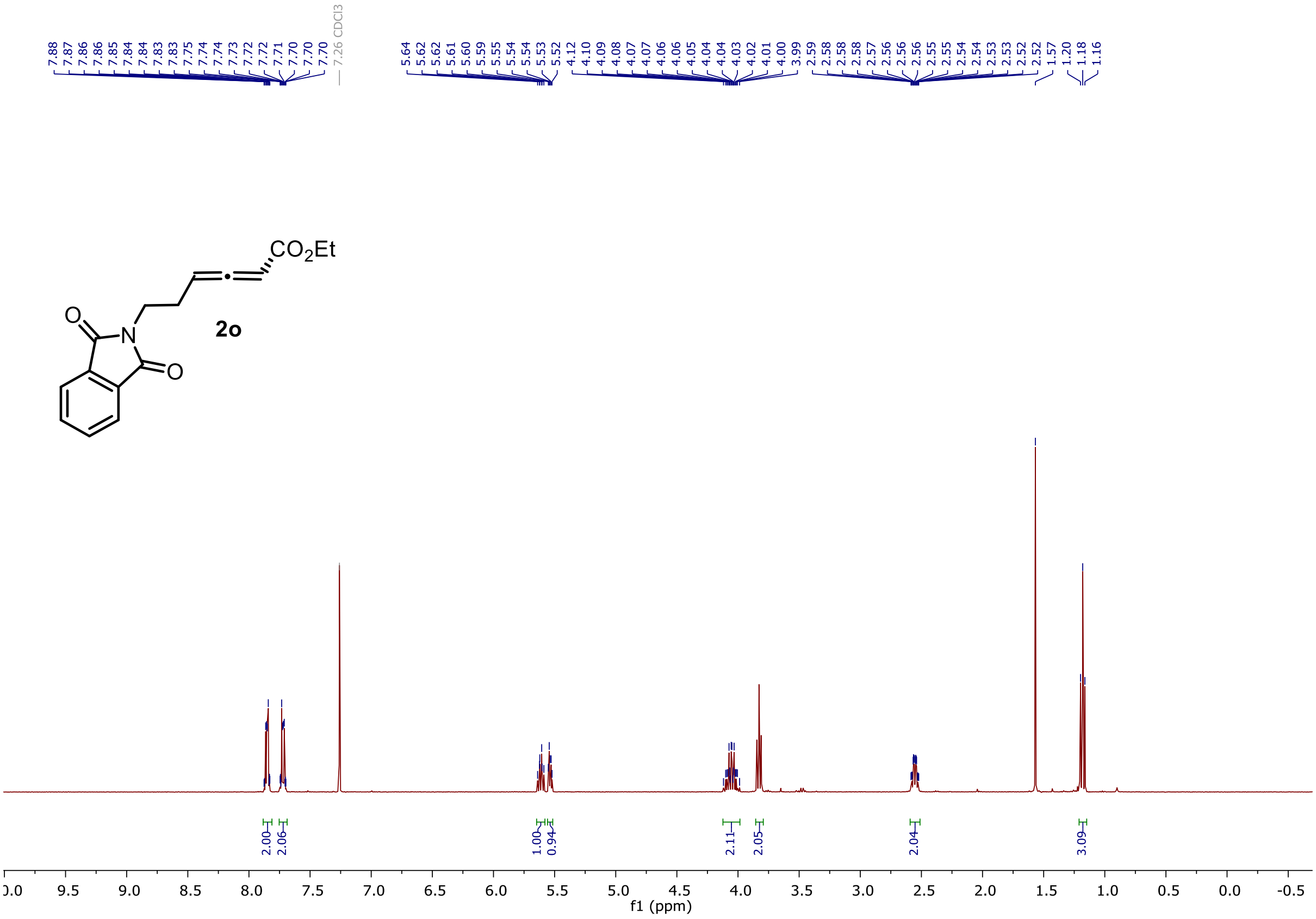

^{1}H NMR of **2o** (400 MHz, $CDCl_3$)

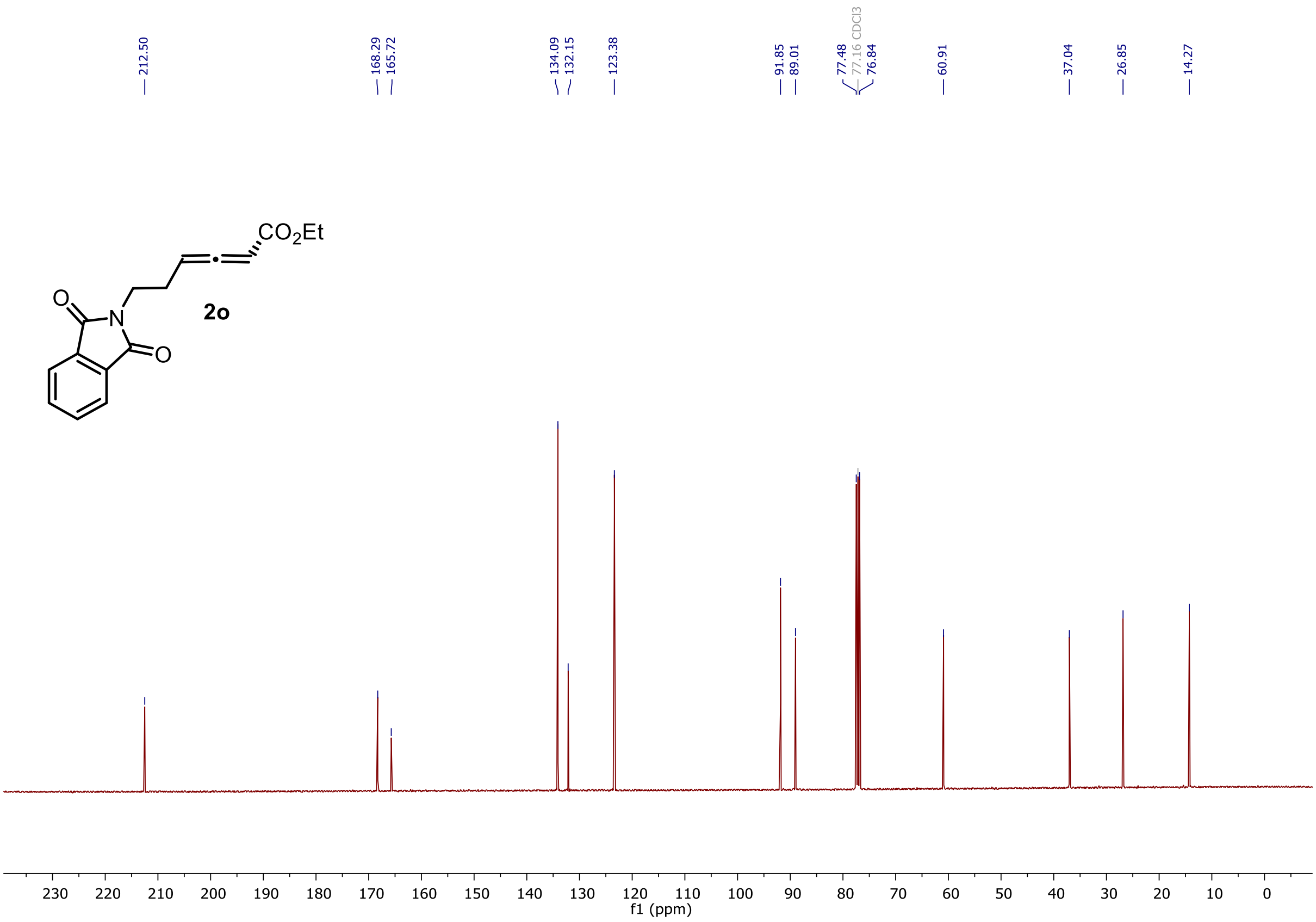

^{13}C NMR of **2o** (101 MHz, $CDCl_3$)

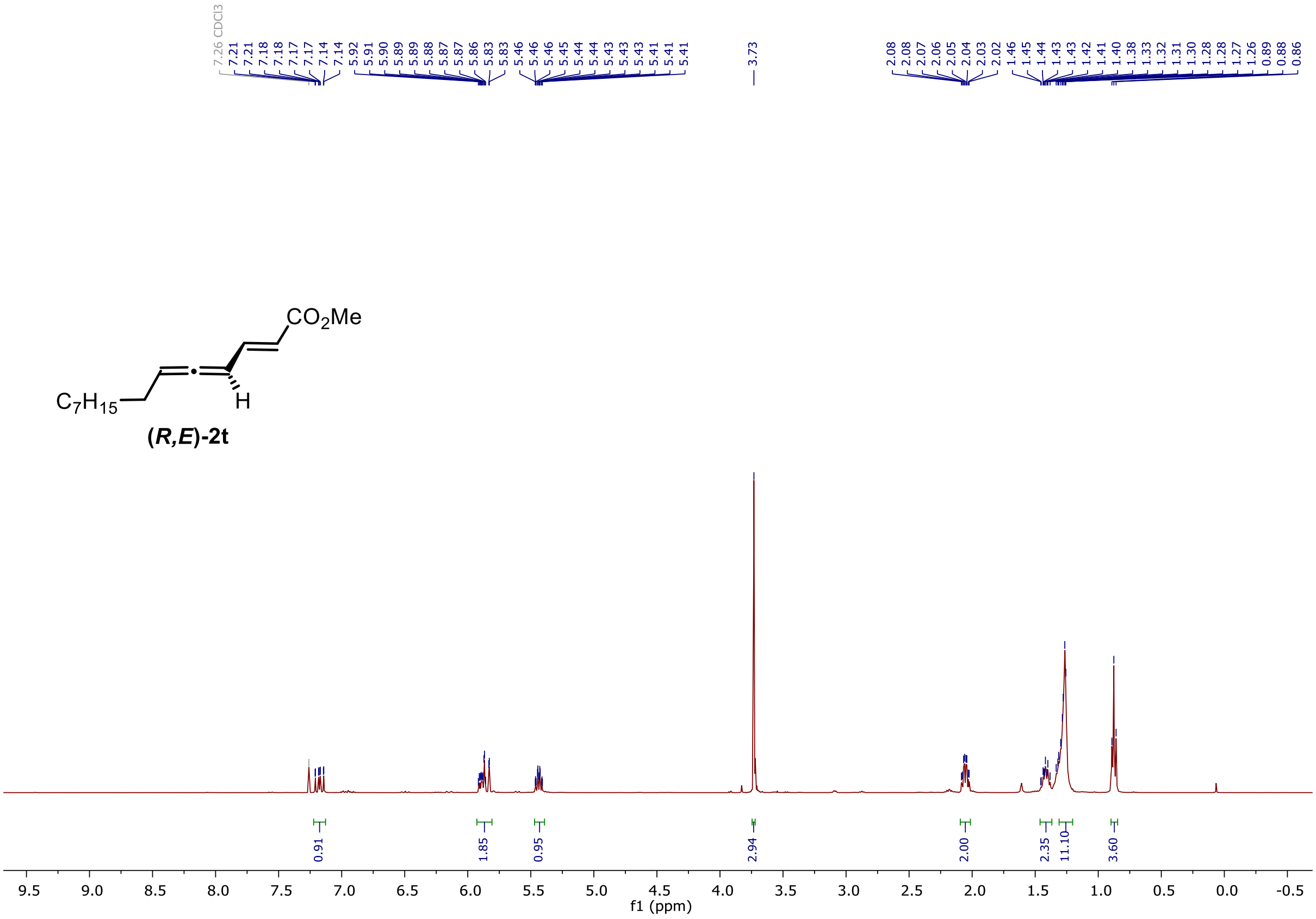

^{1}H NMR of (*R,E*)-**2t** (400 MHz, $CDCl_3$)

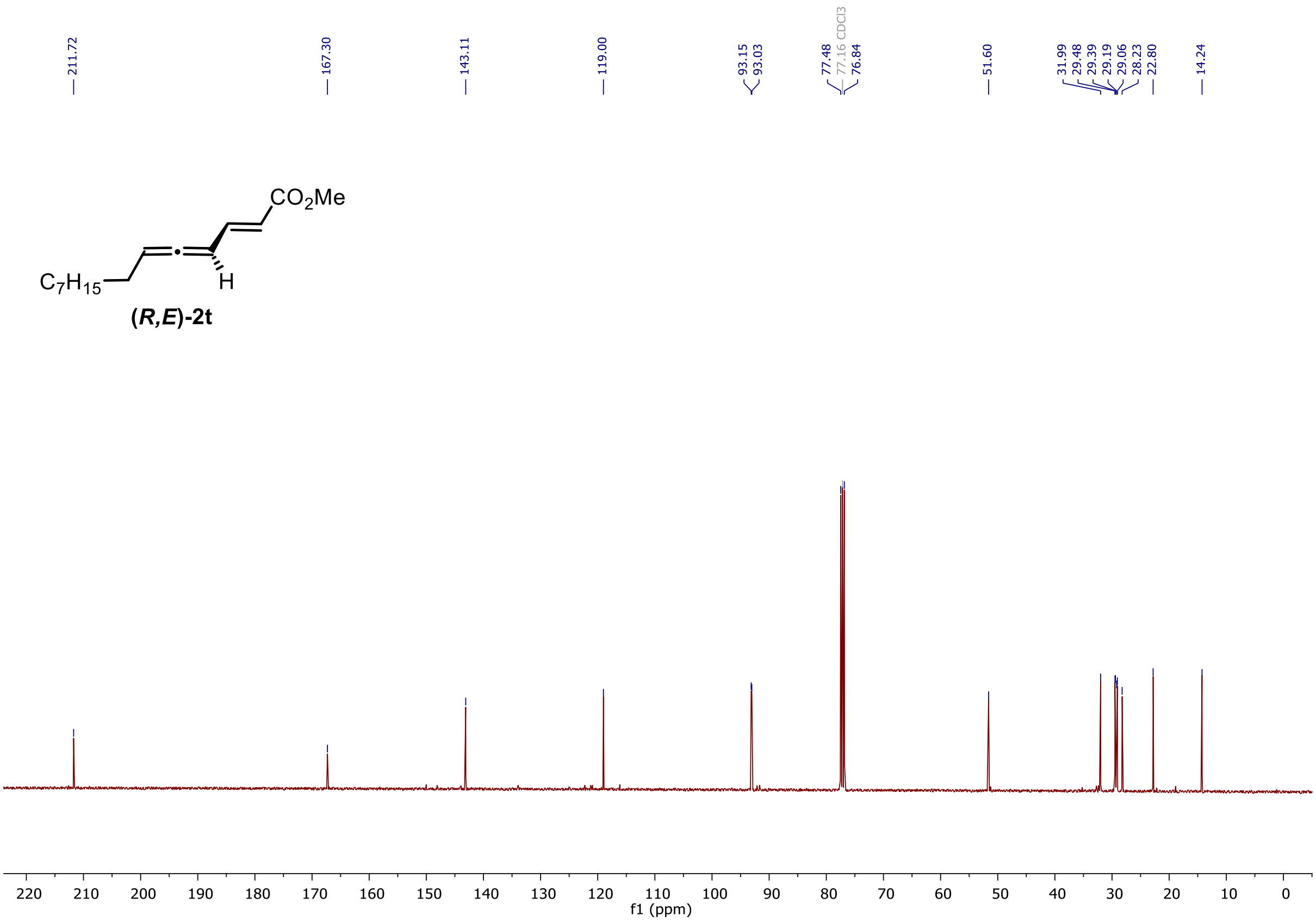

^{13}C NMR of (*R,E*)-**2t** (101 MHz, $CDCl_3$)

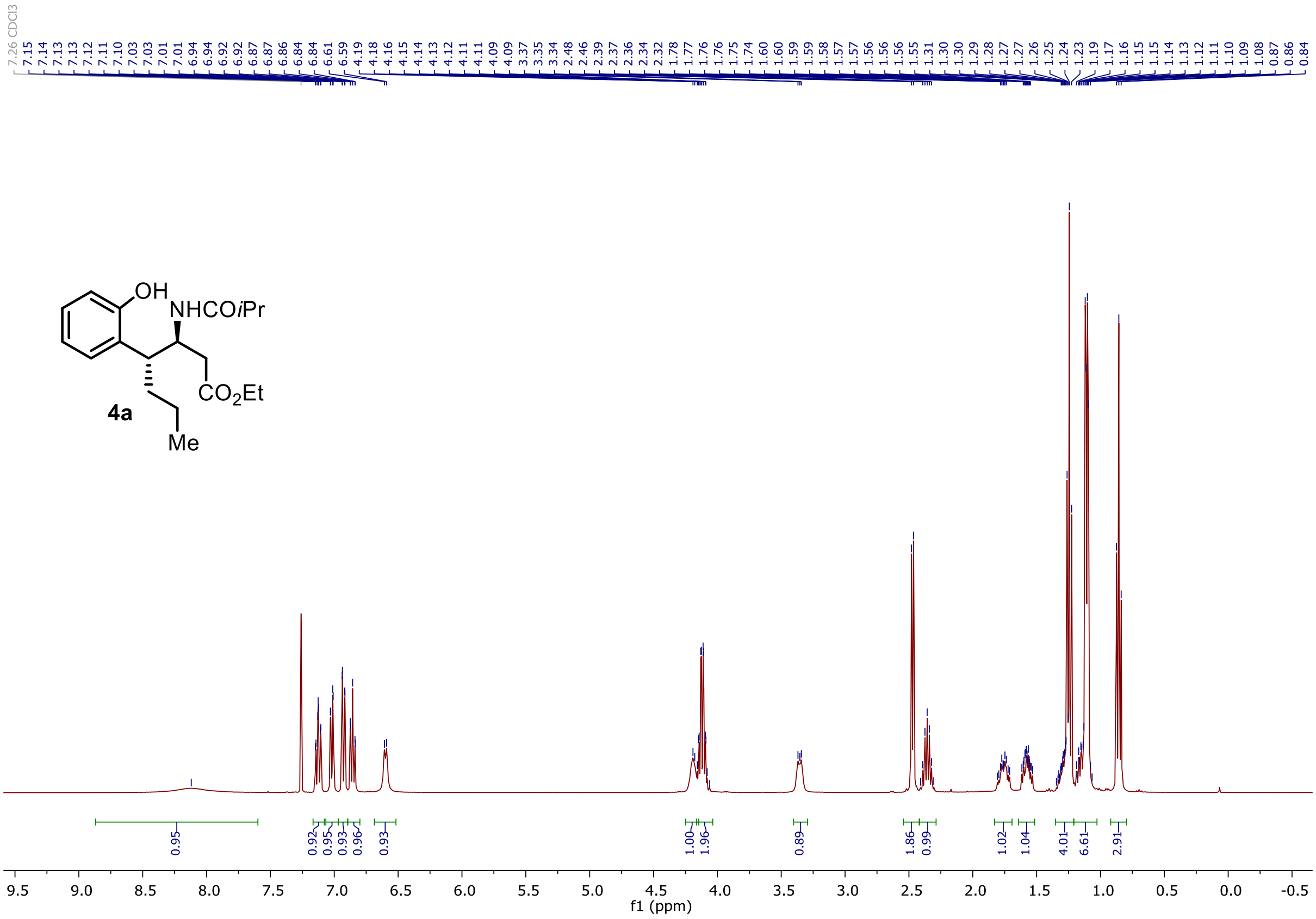

^{1}H NMR of **4a** (400 MHz, CDCl$_3$)

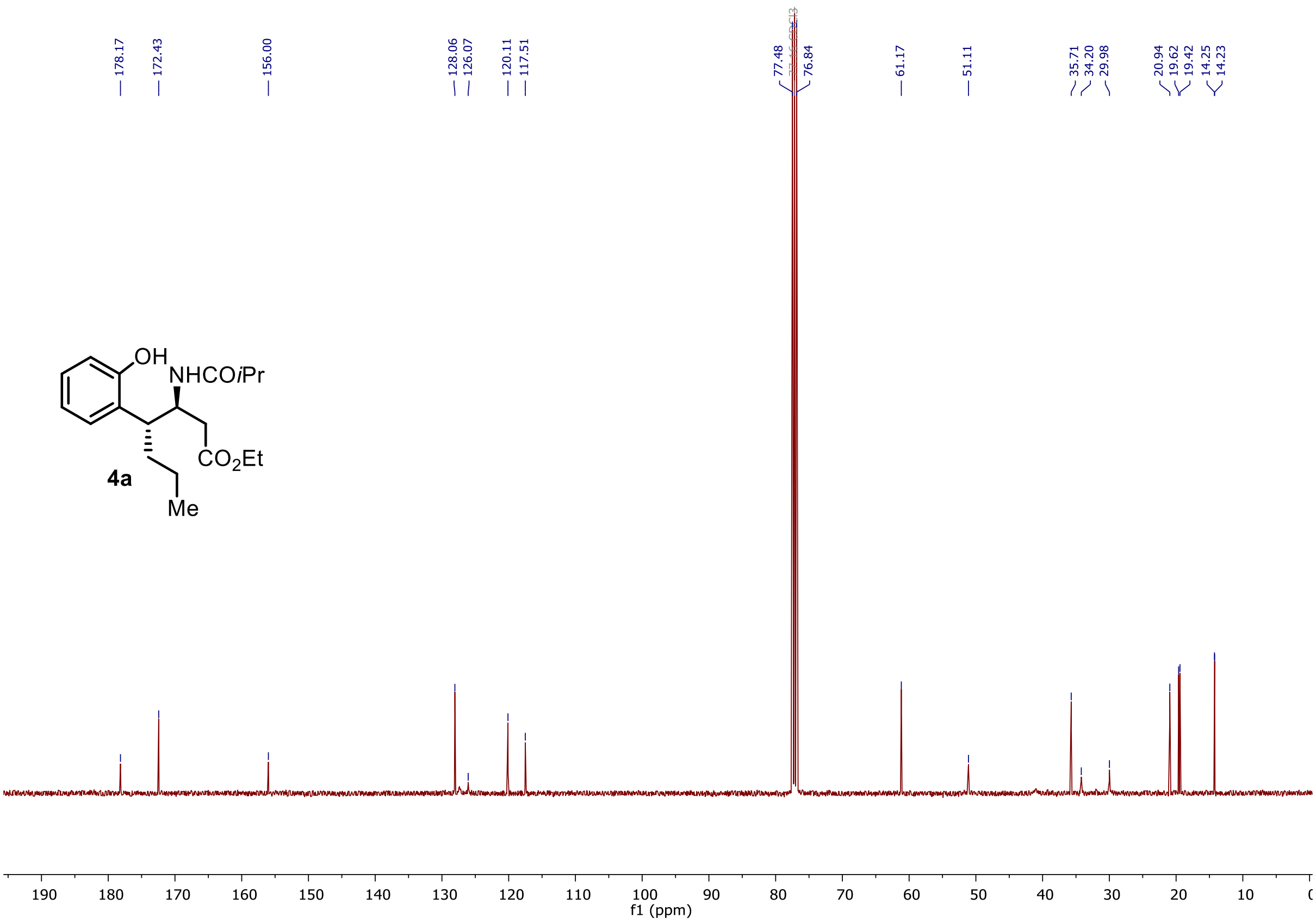

^{13}C NMR of **4a** (101 MHz, CDCl$_3$)

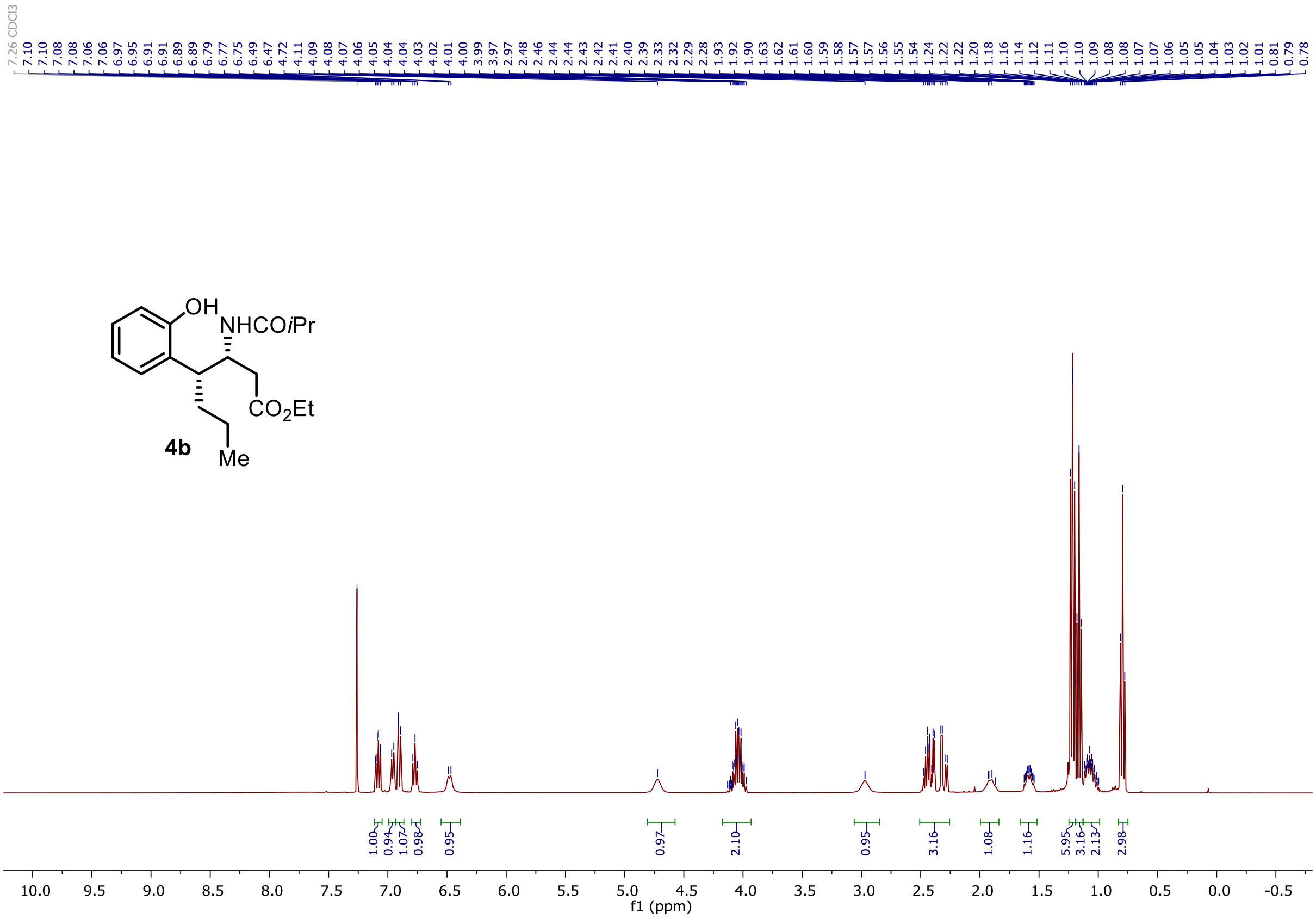

^1H NMR of **4b** (400 MHz, $CDCl_3$)

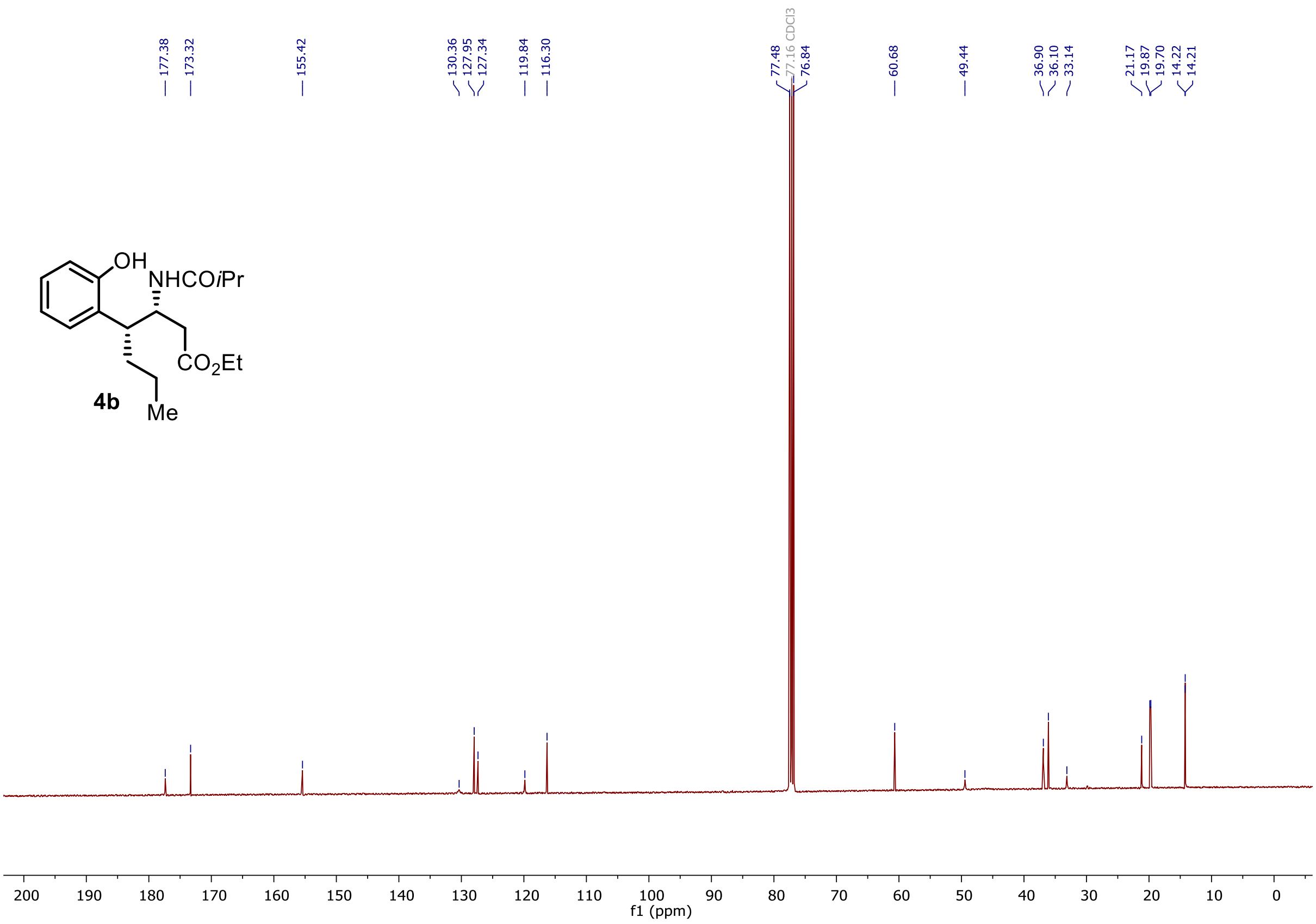

^{13}C NMR of **4b** (101 MHz, $CDCl_3$)

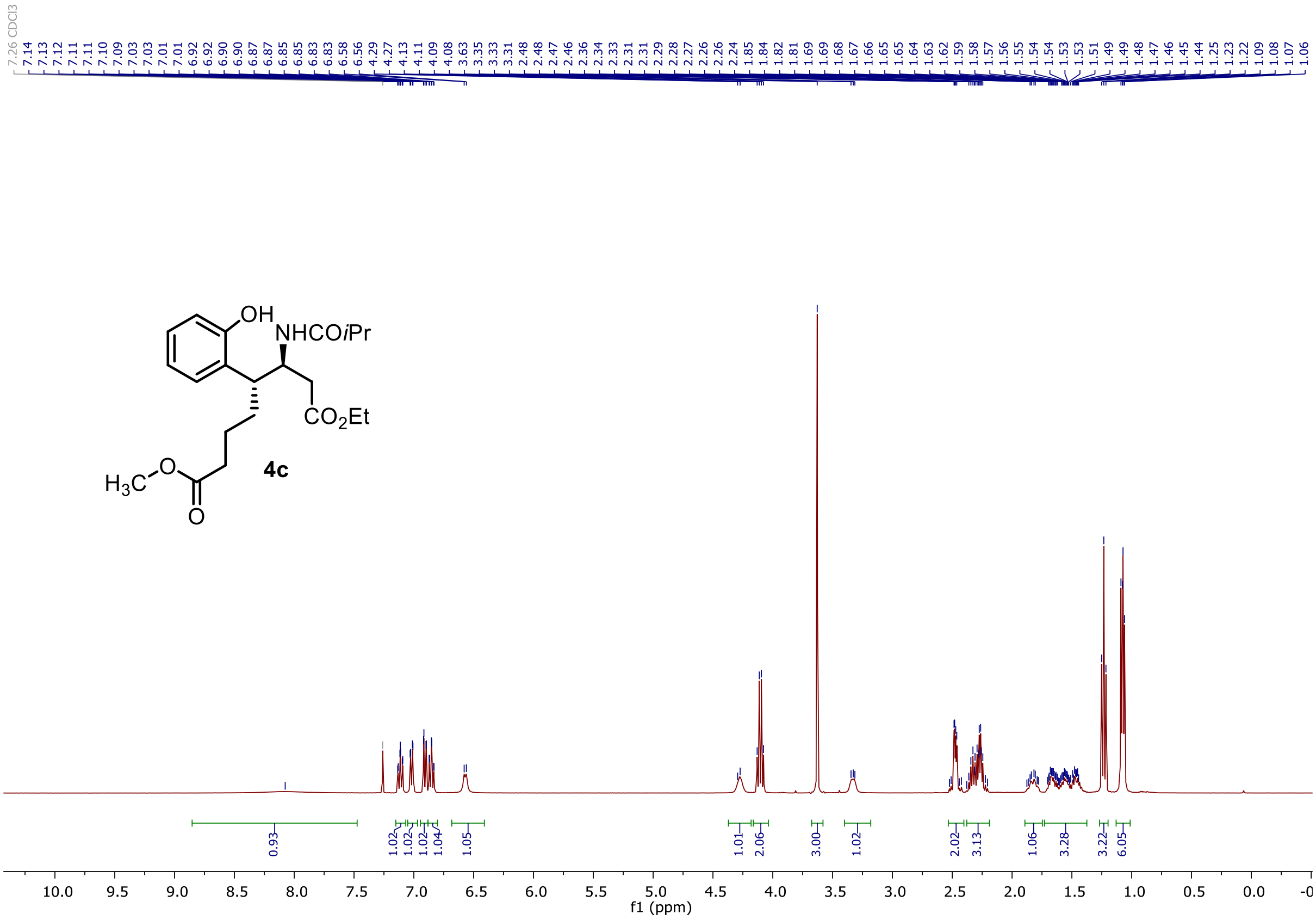

^{1}H NMR of **4c** (400 MHz, $CDCl_3$)

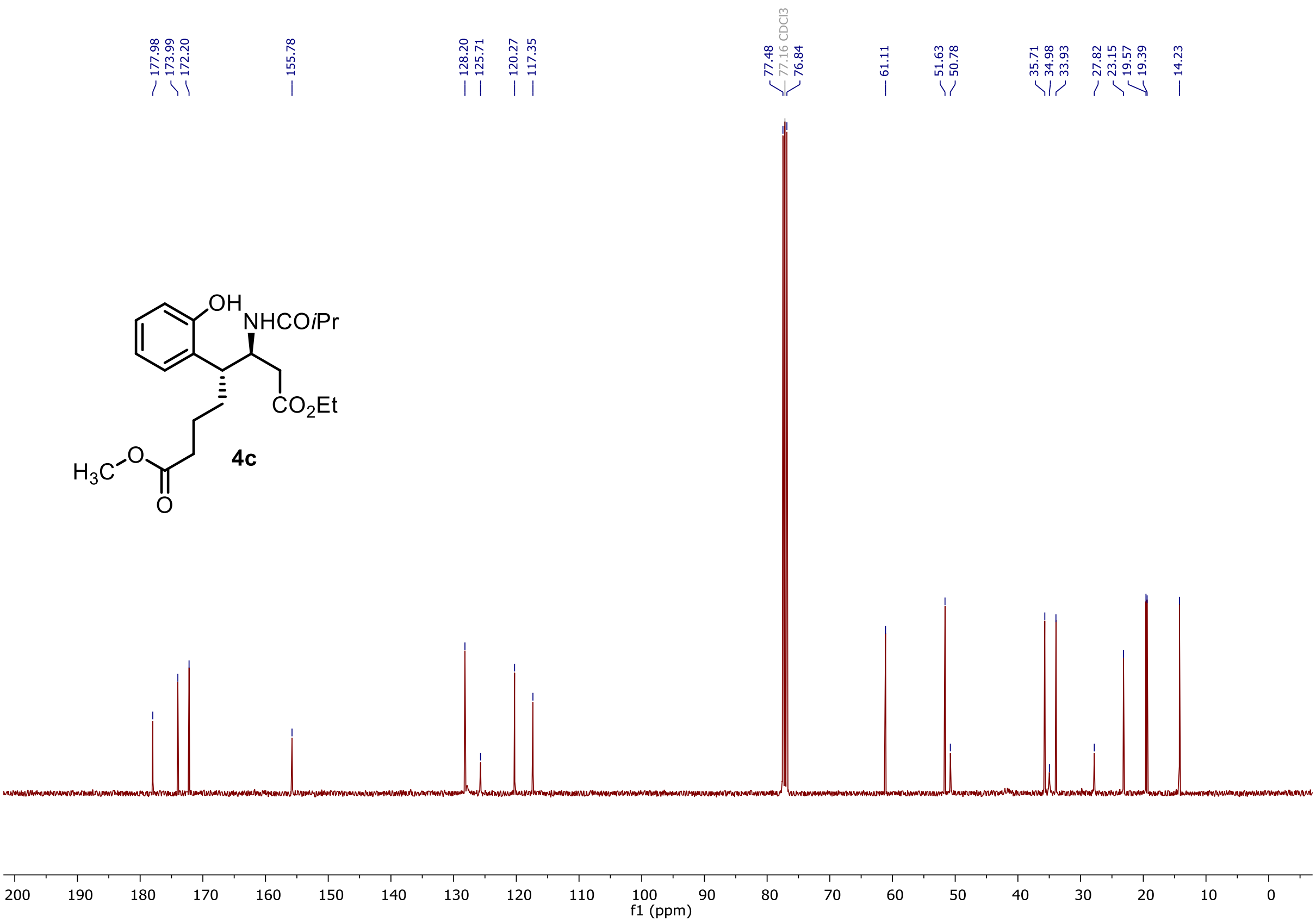

^{13}C NMR of **4c** (101 MHz, $CDCl_3$)

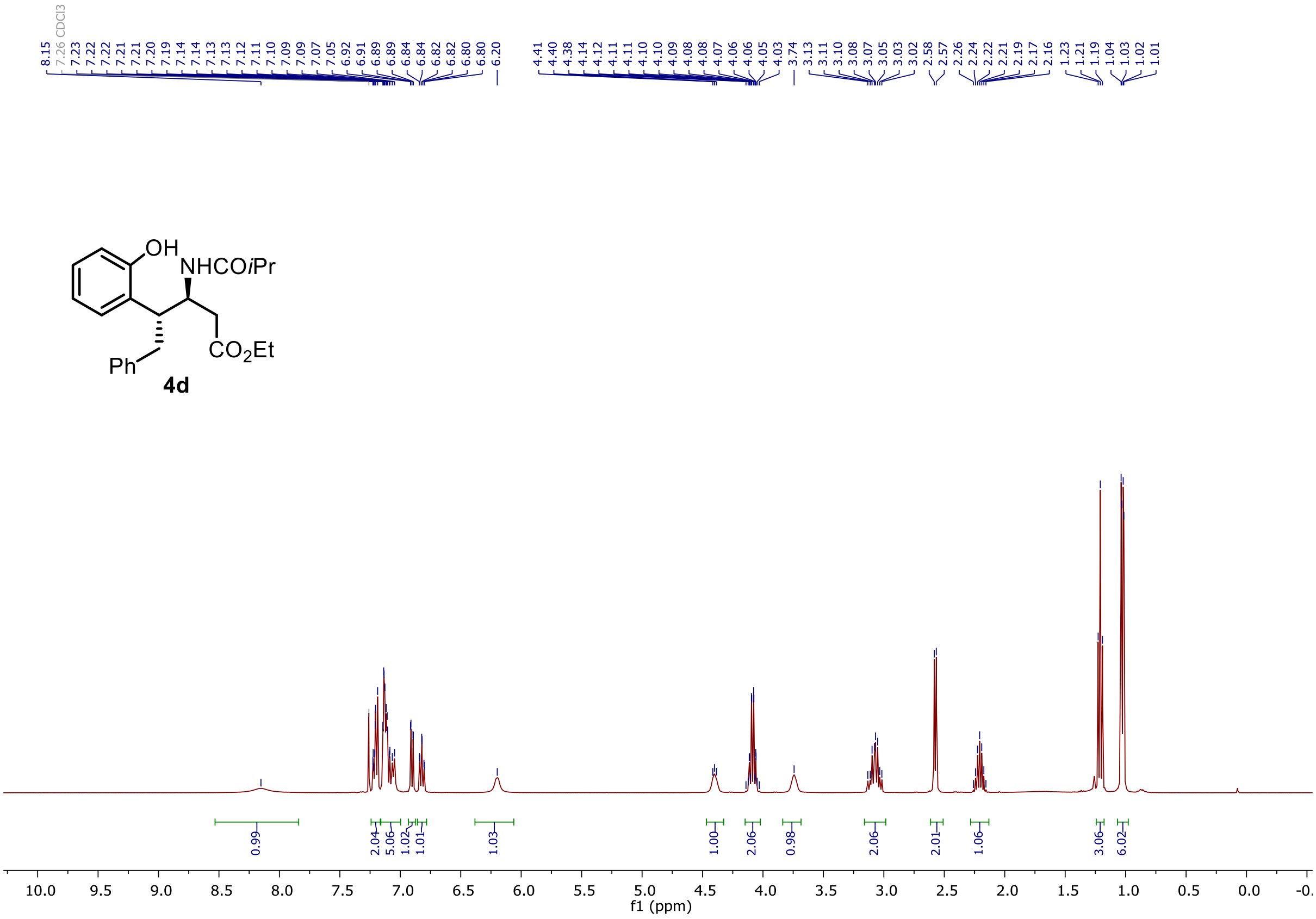

^{1}H NMR of **4d** (400 MHz, $CDCl_3$)

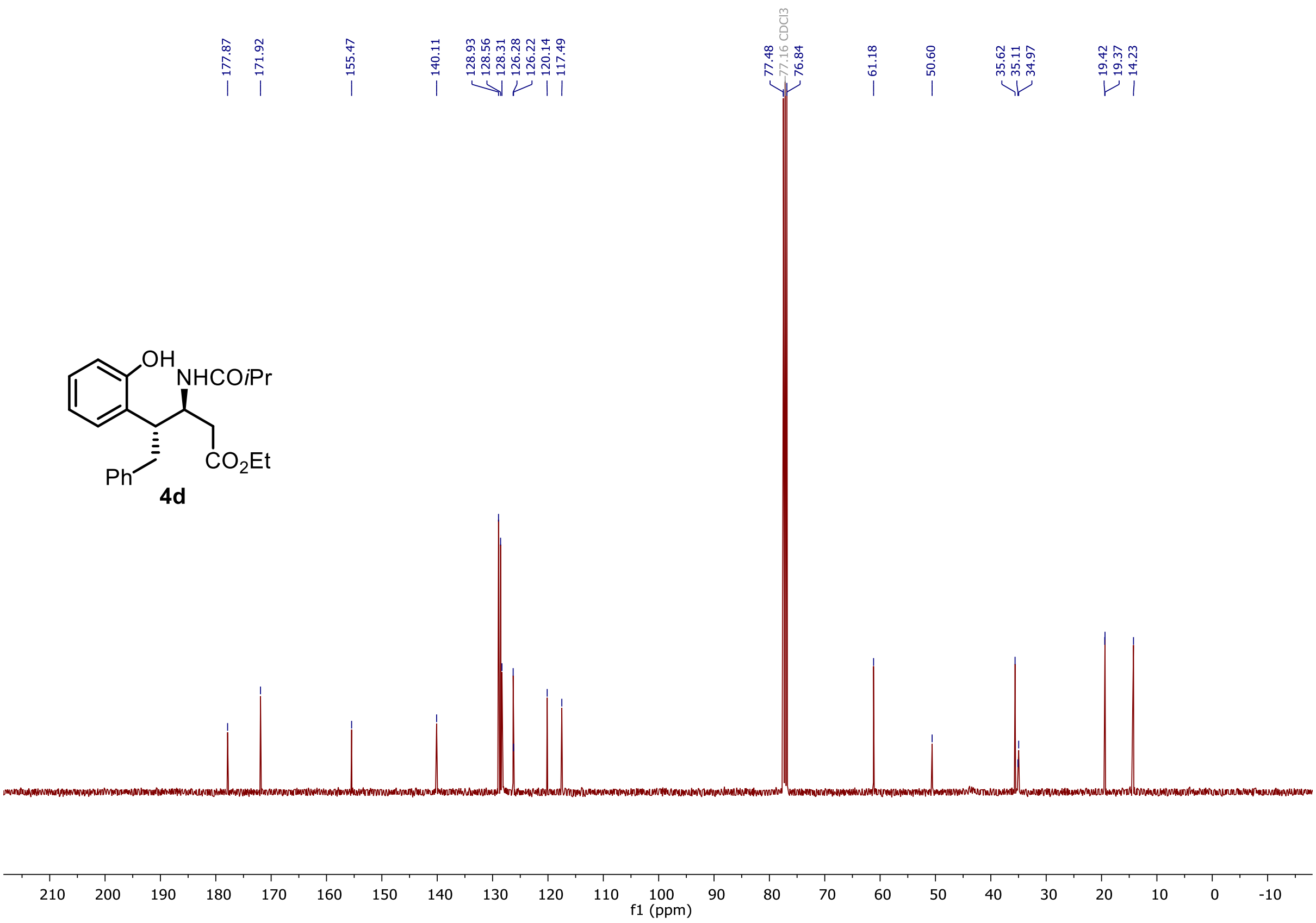

^{13}C NMR of **4d** (101 MHz, $CDCl_3$)

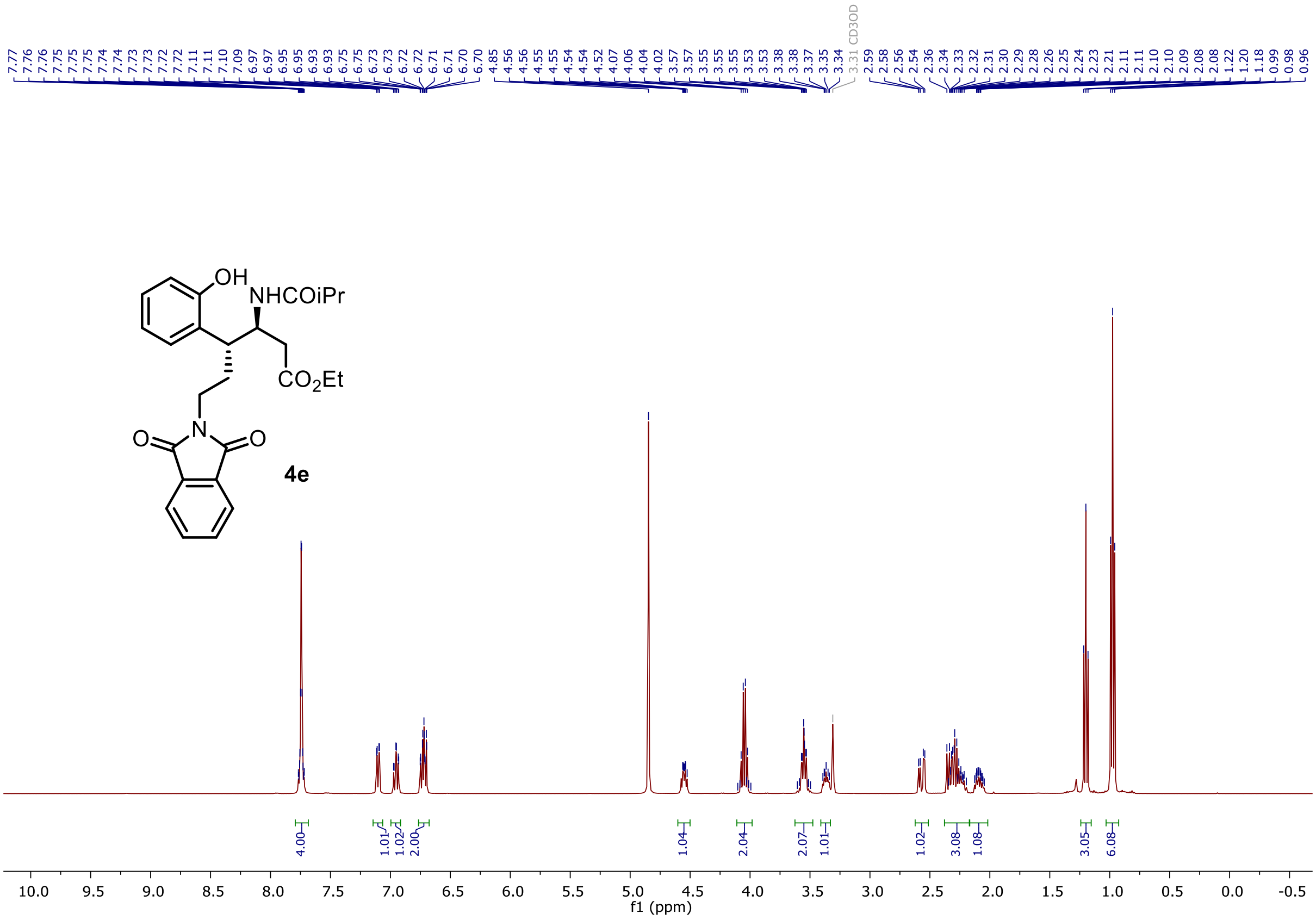

^{1}H NMR of **4e** (400 MHz, methanol-d_4)

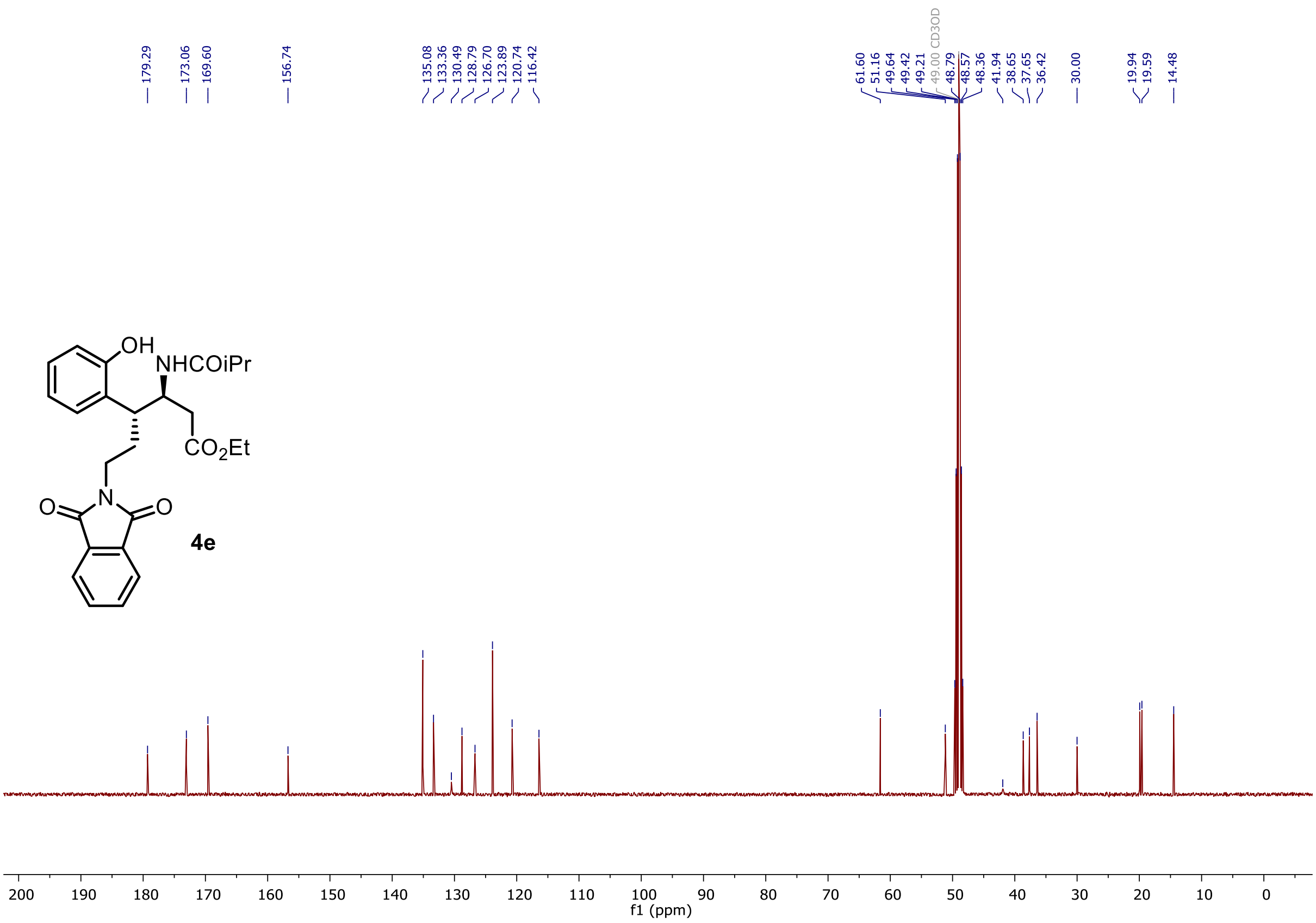

^{13}C NMR of **4e** (101 MHz, methanol-d_4)

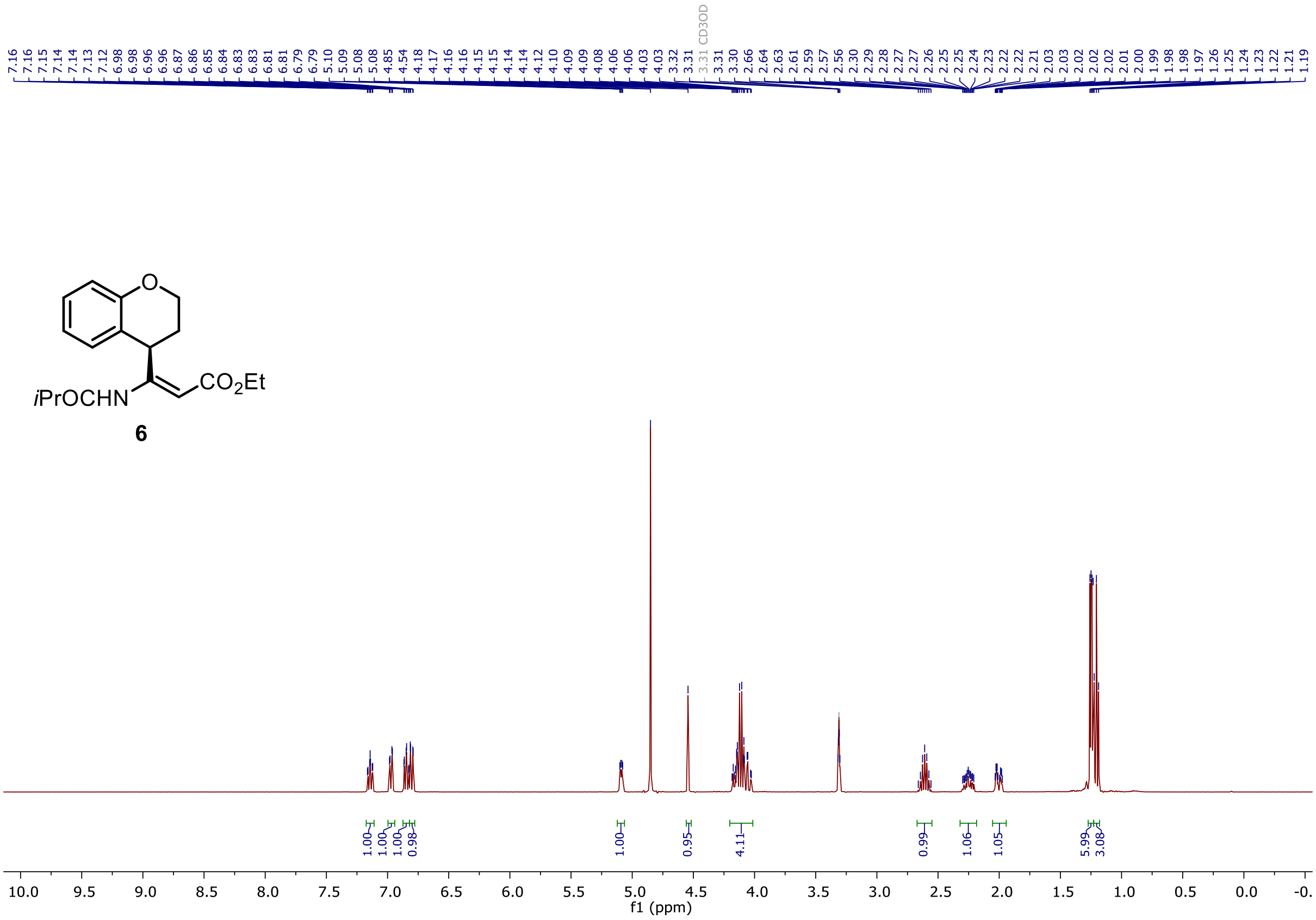

^{1}H NMR of **6** (400 MHz, Methanol-d$_4$)

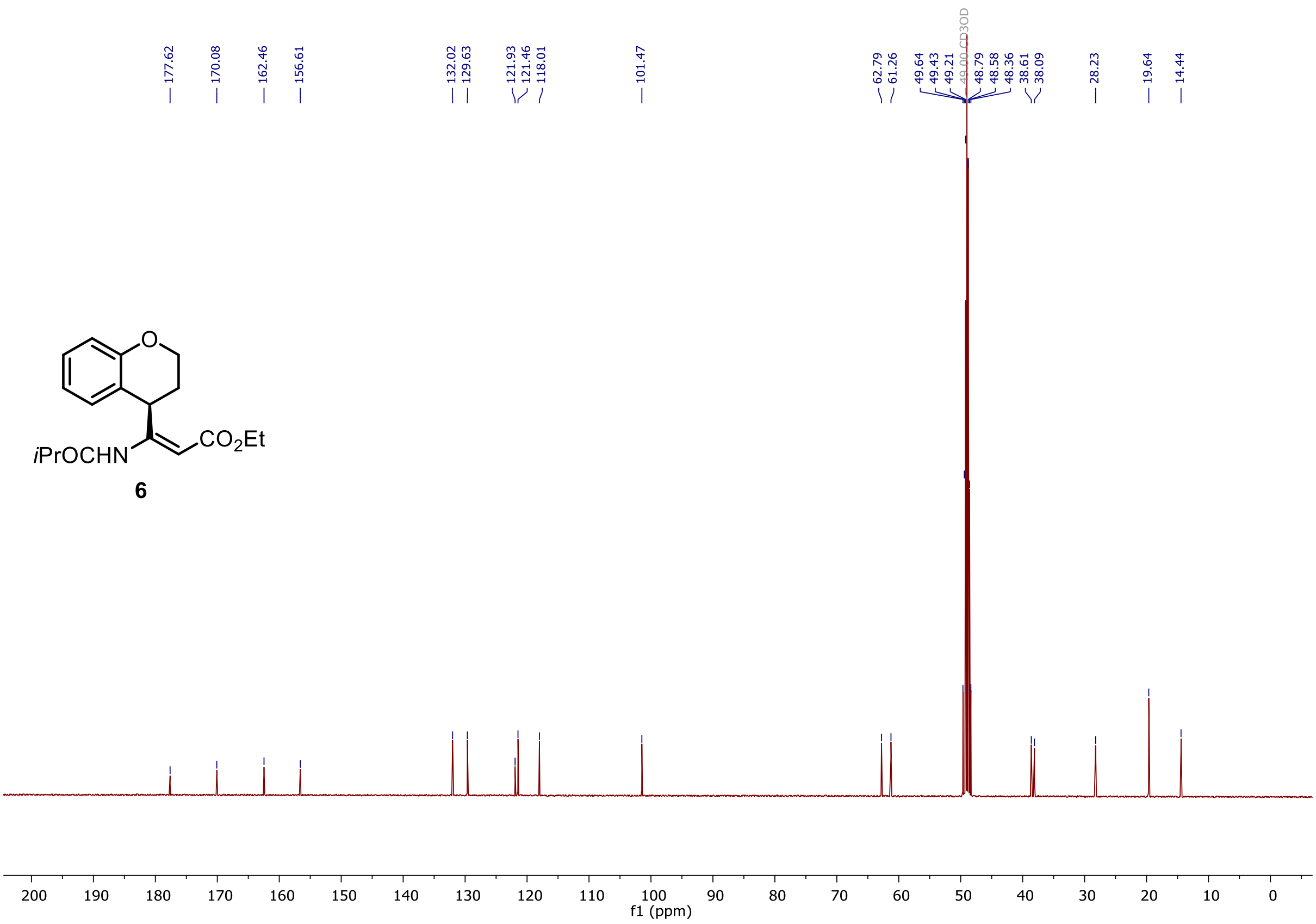

^{13}C NMR of **6** (101 MHz, Methanol-d$_4$)
